# A systematic review of skin ageing genes: gene pleiotropy and genes on the chromosomal band 16q24.3 may drive skin ageing

**DOI:** 10.1038/s41598-022-17443-1

**Published:** 2022-07-30

**Authors:** Jun Yan Ng, Fook Tim Chew

**Affiliations:** Allergy and Molecular Immunology Laboratory, Lee Hiok Kwee Functional Genomics Laboratories, Department of Biological Sciences, Faculty of Science, National University of Singapore, Block S2, Level 5, 14 Science Drive 4, Lower Kent Ridge Road, Singapore, 117543 Singapore

**Keywords:** Genome-wide association studies, Genetic variation

## Abstract

Skin ageing is the result of intrinsic genetic and extrinsic lifestyle factors. However, there is no consensus on skin ageing phenotypes and ways to quantify them. In this systematic review, we first carefully identified 56 skin ageing phenotypes from multiple literature sources and sought the best photo-numeric grading scales to evaluate them. Next, we conducted a systematic review on all 44 Genome-wide Association Studies (GWAS) on skin ageing published to date and identified genetic risk factors (2349 SNPs and 366 genes) associated with skin ageing. We identified 19 promising SNPs found to be significantly (p-Value < 1E−05) associated with skin ageing phenotypes in two or more independent studies. Here we show, using enrichment analyses strategies and gene expression data, that (1) pleiotropy is a recurring theme among skin ageing genes, (2) SNPs associated with skin ageing phenotypes are mostly located in a small handful of 44 pleiotropic and hub genes (mostly on the chromosome band 16q24.3) and 32 skin colour genes. Since numerous genes on the chromosome band 16q24.3 and skin colour genes show pleiotropy, we propose that (1) genes traditionally identified to contribute to skin colour have more than just skin pigmentation roles, and (2) further progress towards understand the development of skin pigmentation requires understanding the contributions of genes on the chromosomal band 16q24.3. We anticipate our systematic review to serve as a hub to locate primary literature sources pertaining to the genetics of skin ageing and to be a starting point for more sophisticated work examining pleiotropic genes, hub genes, and skin ageing phenotypes.

## Introduction

### Definition of skin ageing

Skin ageing is defined as changes to the skin that occur due to ageing. Paying attention to the phrases ‘changes’ and ‘ageing’, ‘changes’ to the skin include but is not limited to histological, morphological, and physiological changes. ‘Ageing’ can be understood as intrinsic ageing from chronological and genetic factors, and extrinsic ageing from environmental factors. Skin ageing is therefore a superimposition of benign skin phenotypes indicative of histological and morphological changes which are both continuous and inevitable, caused by both intrinsic and extrinsic factors, wherein genetic and chronological influences constitute the former, and environmental influences constitute the latter. Building on a previous work by Wong and Chew^1^, in this review, we focus on the intrinsic factors driving skin ageing.

### Background

Skin ageing is an inevitable process which manifests as different morphological phenotypes on the skin, such as wrinkles and pigment spots. Various studies agree that the skin ages differently in different genders, ethnicities, and at different chronological ages.

Skin ageing phenotypes differs between both genders of the same ethnicity. When stratified for gender, Korean women have a greater risk of developing wrinkles compared to Korean men after controlling for chronological age and environmental factors—Sun exposure and smoking^2^. The reverse is true for the Japanese; Japanese men develop more severe forehead wrinkles than Japanese women except in Japanese aged 65 and above^3^. While both genders develop pigment spots on the skin with age, the type of pigmentation differs; hyperpigmented macules are more prominent in Korean women and seborrheic keratosis (i.e. benign growths) are more prominent in Korean men^2^. The higher susceptibility of Korean men to seborrheic keratosis is independent of Sun exposure although a greater lifetime cumulative sunlight exposure (> 6 h/day) further increases the risk of seborrheic keratosis by 2.28-fold than lifetime cumulative sunlight exposure < 3 h/day^4^.

Skin ageing phenotypes differ across ethnicities. Here, we primarily compare Caucasian skin against Asian skin as they are the top two well-studied skin types. As Caucasians have relatively lower melanin content than Asians, Caucasian skin is less protected from photodamage by the Sun and wrinkles and sags more than other races^5^. In contrast, Asian skin has higher melanin content; while this pigment protects the Asian skin from photodamage, the aged Asian skin often has pigment spots and abnormal pigmentation^6^. Specifically, 30-to-60 years old Asians (Chinese and Japanese) have pigment spots on cheeks more often than age-matched Germans. In comparison, the Germans have wrinkles under the eyes more often than their Asian counterparts^7^. Other factors besides photodamage may still account for wrinkles on Asian faces^2^ in which Asians have coarser, thicker and deeper wrinkles, often on the forehead, perioral and Crow’s Feet area while Caucasians have relatively fine wrinkles on their cheeks and the Crow’s Feet Area^8^. Attempts to study the skin of other races (e.g., Hispanics^9^ and people with black skin) are much more limited in the literature. Nonetheless, people with black skin^10^ resemble Asians^11^ in that abnormal pigmentation occurs more frequently in these two races when compared with Caucasian skin. When Caucasians do have abnormal pigmentation, this tends to occur at an earlier age as compared to people with black skin^12^. One paper speculates that the lifestyle, eating habits, and living environment all play a part in contributing to these differences in the diversity of skin ageing phenotypes in people of colour^6^.

The pace of skin ageing differs at different chronological ages. Chinese females develop wrinkles rapidly between the ages of 40 and 50 while French women develop wrinkles at a relatively linear rate from ages 20 to 60^13^. Hispanics resemble Caucasians in that wrinkles develop early in life; fine wrinkles have been observed in Latin Americans with a mean age of 24.0^14^.

Skin ageing features may develop in a sequential manner. Solar lentigines, a type of pigment spot, occur at a younger age and are more conspicuous on Asian skin than Caucasian skin^15^. In the Japanese, women are more likely to develop pigment spots such as solar lentigines in early adulthood whereas facial wrinkles develop mostly between the ages of 30 and 50^15^.

All these differences in the presentation of skin ageing features across age, gender, and ethnicities despite controlling for environmental factors such as Sun exposure supports the idea that genetics influence the type, intensity, and developmental pace of different skin ageing phenotypes.

Indeed, it has been widely reported in numerous Genome-wide Association Studies (GWAS) that Single Nucleotide Polymorphisms (SNPs) are significantly associated (p-Value < 1E−05) with skin ageing phenotypes.

### Aim of review

This review has two aims. First, this review aims to deliver a comprehensive and detailed look into the genetics of skin ageing and all skin ageing phenotypes ever collected and measured by the skin ageing field. We report that it is common practice in the skin ageing field to assess skin ageing phenotypes visually and to study genetics through Genome-wide Association Studies (GWAS) using the additive allele model as opposed to candidate gene studies or other allele models.

Second, this review aims to illuminate the underlying genetic structure (e.g., pleiotropy) and biological processes of skin ageing through bioinformatics methodologies such as enrichment analyses. Multiple lines of evidence (e.g., SNP, gene expression levels, pleiotropy, enrichment) are used to support the association of a given gene (e.g., SPIRE2) and skin ageing. We also identified hub proteins: highly interconnected proteins in the gene network whose frequent interactions could illuminate relevant pathways driving the development of key skin ageing phenotypes.

## Methodology

### Search strategy for skin ageing phenotypes

#### Identifying skin ageing phenotypes

In this section, we identify and compile a list of changes that occur to the skin which are also widely accepted to be changes that occur due to ageing. We consulted skin ageing scales because they are frequently used to identify and quantify skin ageing phenotypes.

We searched the Web of Science for high quality validated skin ageing scales using the following search term: ((skin ageing OR skin aging) AND (scale) AND (validat*)).

We first included five scales that meet the criteria set by the Consensus-based Standards for the selection of health Measurement Instruments (COSMIN) because they are multidimensional assessment scales determined to have high methodological quality for all skin types. These are the Skin Ageing Score (SAS)^16^, Score of Intrinsic and Extrinsic skin Ageing (SCINEXA)^17^, Merz Aesthetics Scale (MAS)^18^, an unnamed scale by Allerhand et al*.*^19^ and an unnamed scale by Flament et al*.*^20^. These five scales identified 30 skin ageing phenotypes.

Previously, Dobos et al.^9^ have addressed the hundreds of available skin ageing scales that grade one specific phenotype each. Their team performed a systematic review on 100 scales from 130 studies and shortlisted several scales with high methodology, high performance, and focused on interrater reliability. We built upon their findings by studying each shortlisted scale independently and checking the way each phenotype is defined and comparing them against the definitions of the 30 phenotypes in COSMIN. This yielded several duplicates which indicate a degree of agreement across the scales. We including ‘forehead wrinkles’ and ‘low brow positioning’ from the upper face ageing scale by Flynn et al*.*^21^, ‘glabellar wrinkles’ from the scale by Honeck et al*.*^22^, ‘upper lip fullness’, ‘lower lip fullness’, and ‘sagging of jawline’ from the lower face ageing scale by Narins et al.^23^, ‘melomental folds’ from the scale by Carruthers et al*.*^24^, and ‘neck sagging’ from the scale by Sattler et al*.*^25^. Altogether, these five scales added eight more skin ageing phenotypes.

We consulted the Skin Ageing Atlas^26^ and added two phenotypes, namely ‘interocular wrinkles horizontal’ and ‘cheek skin pores appear larger’.

We also consulted two reputable medical books—Dermatology 3rd Ed.^27^ and Fitzpatrick’s Dermatology in General Medicine, 8th Ed.^28^. These two books added four more skin ageing phenotypes, namely ‘guttate hypomelanosis’, ‘venous lakes’, ‘senile purpura’, and ‘sebaceous hyperplasia’.

Finally, we searched three databases—PubMed (https://pubmed.ncbi.nlm.nih.gov/)^29^, Web of Science (http://www.webofknowledge.com), and Embase (http://www.embase.com)—for existing skin ageing GWAS literature (Table 1) and included relevant phenotypes.

**Table 1:** Search terms used to search for SNPs associated with skin ageing phenotypes.

Database	Results	Search term
**For the whole world**		
PubMed	25	(skin ageing OR skin aging) AND (GWAS) AND (genotype), all time
Web of science, all databases	71	(skin ageing OR skin aging) AND (GWAS) AND (genotype), all time
Embase	46	(skin ageing OR skin aging) AND (GWAS) AND (genotype), all time
**For Han Chinese**		
PubMed	6	(skin ageing OR skin aging) AND (Chinese) AND (SNP), all time
Web of science, all databases	66	(skin ageing OR skin aging) AND (Chinese) AND (SNP), all time
Embase	28	(skin ageing OR skin aging) AND (Chinese) AND (SNP), all time

Altogether, these sources identified 56 skin ageing phenotypes (Table 2), which we use as our definition of skin ageing phenotypes in all subsequent discussions. The 56 skin ageing phenotypes are discussed in detail in “Results” “Skin ageing phenotypes” section. Briefly, the 56 skin ageing phenotypes can be broadly grouped into four key phenotype categories by their morphology—Category A Phenotype are skin wrinkling and sagging-related phenotypes; Category B Phenotype are skin colour-related phenotypes; Category C Phenotype are skin cancer-related phenotypes; and Category D Phenotype are skin global impression phenotypes.

**Table 2:** The 56 skin ageing phenotypes.

Phenotype ID	Skin ageing phenotype
A1	Absence of fat tissue/reduced fat tissue
A2	Cheek skin pores appear larger
A3	Coarse wrinkles on cheek/coarse cheek folds
A4	Cris-cross wrinkles/perioral wrinkles/perioral lines/lip wrinkles
A5	Cutis rhomboidalis nuchae
A6	Deep wrinkles/coarse wrinkles/lateral canthal lines/crow’s feet
A7	Droopy eye(s)/ptosis of eyelids
A8	Eyebags
A9	Favre-Racouchot syndrome
A10	Fine lines on cheek/fine cheek folds
A11	Forehead wrinkles/forehead lines
A12	Glabellar lines/forehead furrows
A13	Horizontal interocular wrinkles
A14	Lax appearance/tissue slacking
A15	Low brow positioning
A16	Lower lip fullness
A17	Melomental folds/Marionette lines/drooping of labial commissures
A18	Nasolabial folds
A19	Pseudoscar/pseudo scar/stellate pseudoscar
A20	Sagging of jawline
A21	Sagging or wrinkling of the neck skin or having an obtuse cervicomental angle
A22	Solar elastosis/actinic elastosis/elastosis senilis
A23	Superficial wrinkles/fine wrinkles/lateral canthal lines/crow’s feet
A24	Upper lip fullness
A25	Wrinkles (detected by shaded lines on the face)
A26	Wrinkles under eyes/periorbital wrinkles upper cheek area
A27	Xerosis/asteatotic dermatitis/higher TEWL
B1	Facial pigmented spots (detected by polarised light capturing the skin surface)
B2	Facial pigmented spots (detected by UV light capturing the inside of the epidermis)
B3	Freckles/sunburn freckles/ephelides
B4	Guttate hypomelanosis
B5	Melasma
B6	Milia
B7	Perceived skin darkness
B8	Permanent erythema/skin flushing
B9	Sebaceous hyperplasia
B10	Seborrheic keratosis/benign skin tumours
B11	Senile comedone/senile comedo/solar comedo/periorbital comedone
B12	Senile purpura
B13	Skin colour/skin colour (extent of darkness)/lower reflectance skin (darker skin than normal, higher melanin content)
B14	Skin type—sun sensitivity/skin sensitivity to the Sun
B15	Skin type—tanning type/inability to tan/burn rather than tan/sunburn type
B16	Solar lentigines/solar lentigo/senile lentigo/lentigo senilis/age spot/liver spot/pigment spots (darkness)
B17	Telangiectasias
B18	Uneven pigment/uneven pigmentation/pigment spot
B19	Venous lakes
B20	Yellowish decolouration/yellowness
C1	Actinic keratosis/actinic precancerosis/solar keratosis
C2	Basal cell carcinoma/cutaneous basal cell carcinoma
C3	Malignant melanoma/cutaneous malignant melanoma/melanoma
C4	Non-melanoma skin cancer
C5	Squamous cell carcinoma/cutaneous squamous cell carcinoma of the skin
D1	Different gene expression in young skin compared to aged skin, the influence of this SNP changes with age (increase/decrease)
D2	Facial skin is more youthful than normal
D3	Global facial photoageing
D4	Perceived age is different from actual age (older than/younger than)

Varying terminology used by different studies for identical phenotypes are separated by a slash (/).

### Search strategy for skin ageing genes

#### Identifying skin ageing genes containing SNPs associated with skin ageing phenotypes

**Figure 1:**
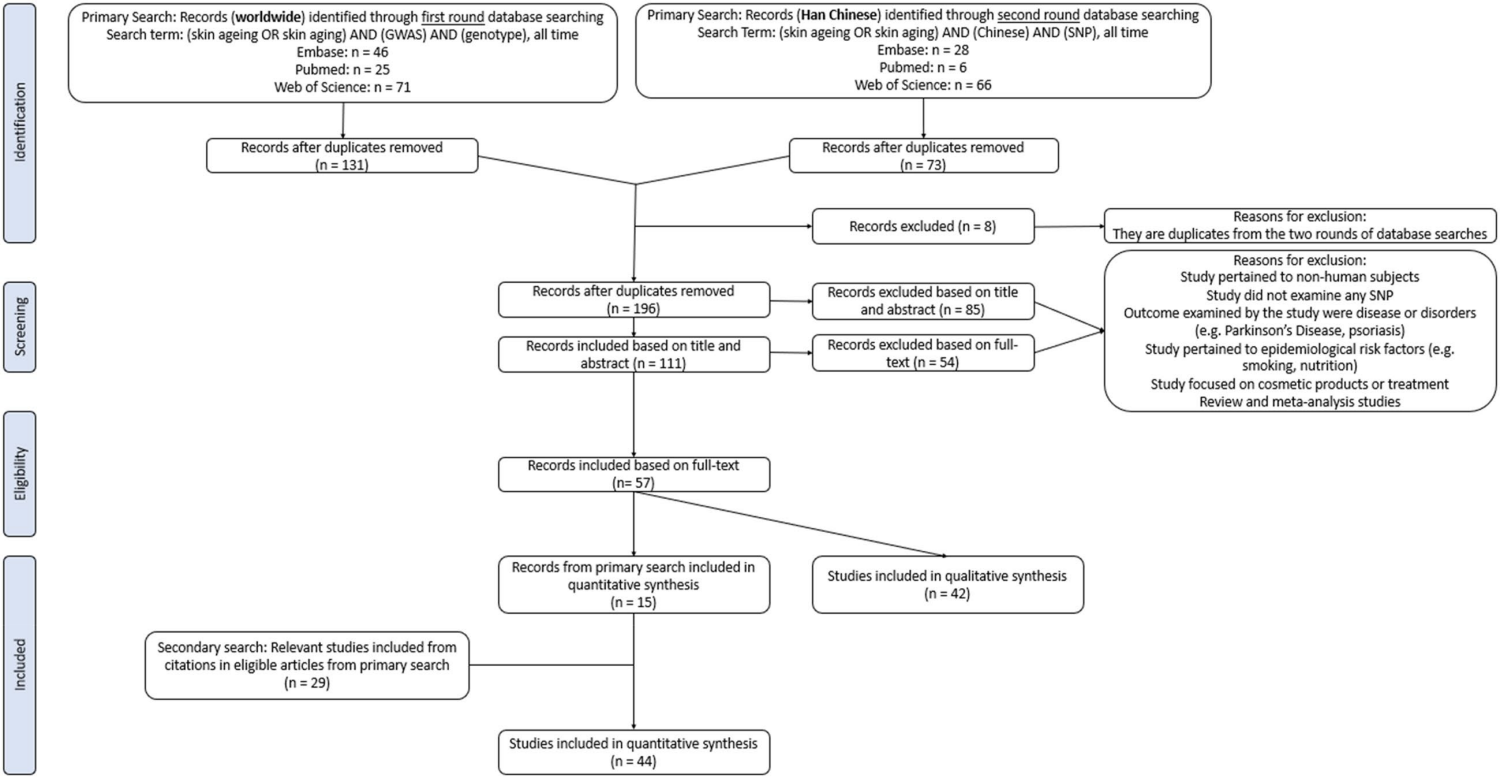
Workflow for screening through publications to include in the review.

This review was conducted in accordance with the Preferred Reporting Item for Systematic Review and Meta-Analyses (PRISMA) guidelines (Supplementary Table S1). A search specifically for candidate gene papers as opposed to GWAS did not return records for wrinkles, and only returned records on skin cancer^30–32^ and pigmentation changes^7,33,34^. All records on wrinkles are from GWAS. Therefore, a primary literature search on GWAS was performed using the Embase, PubMed and Web of Science databases in July 2021. Search results were restricted to English journal articles published any time before and including 2021. The search term for all databases included ‘skin aging’ or ‘skin ageing’ in the title or abstract, and ‘GWAS’ and ‘genotype’ in all index fields. To be exhaustive for East Asian results, we performed another primary literature search. The search term for all databases included ‘skin aging’ or ‘skin ageing’ in the title or abstract, and ‘Chinese’ and ‘SNP’ in all index fields. Full search terms for their respective databases are summarised in Table 1. To remain current, searches were reperformed in November 2021. Eligible articles from the primary search were determined using pre-defined eligibility criteria. A secondary search was conducted by manually searching references cited by the eligible articles from the primary search. Results obtained in the secondary search were deduplicated and screened using the same eligibility criteria as that applied in the primary search. The manual search process was repeated for results from the secondary search to ensure a thorough record was obtained (Fig. 1). A final search using specific skin ageing phenotypes as keywords only returned records that were duplicates of previous results or irrelevant to this review’s aims, ensuring that the literature search illustrated in Fig. 1 was sufficiently thorough.

#### Eligibility criteria

We included articles into this review in accordance with our definition of skin ageing and stated aims. The included articles examined association of SNPs with skin ageing phenotypes, both intrinsic and extrinsic, in any human subject assessed via non-invasive methods, by means of a non-experimental observational study. All full-text journal articles available in English were included. Studies excluded from the review met at least one of the following criteria: study pertained to non-human subjects (e.g., swine or dog experiments); study did not examine any SNP; phenotypes examined by the study were disease or disorders (e.g., Parkinson’s Disease, psoriasis); study pertained to epidemiological risk factors (e.g., smoking, nutrition); study focused on cosmetic products or treatments; studies were reviews or meta-analyses. Phenotypes quantified using digital image analysis were included. Using this eligibility criteria, a total of 240 records were screened. Screening was performed in two stages. In the first stage, articles with titles and abstracts irrelevant to this review’s aims were discarded. In the second stage, full-length journal articles were retrieved for all remaining papers and each record was manually assessed for suitability. Full-text articles excluded from the review were either review papers or meta-analyses papers.

#### Data extraction

The following variables were obtained from the respective articles and supplementary materials: author(s) and year, country, sample size and subject demographics, genetic background (SNP rsID, effect allele, allele model), and phenotype details (phenotype, anatomical location, and definition if any). In several studies, participants were not recruited and only photographs of their faces were investigated; the number of photographs included in statistical analysis, if reported, was extracted, and considered as the sample size. In several studies, the number of recruited participants and the number of participants with genetic data available to the investigators differed; the number of participants included in statistical analysis, if reported, was extracted, and considered as the sample size. In several studies, the same SNP was tested for associations with multiple phenotypes and only one result has a significant p-Value (p-Value < 1E−05); to identify between phenotypes that have been tested for this SNP and phenotypes that have never been tested for this SNP in any literature, all results are retained regardless of their p-Value. To account for varying terminology used by different studies for identical phenotypes, we compared the definition of the phenotype in words or photographs, if any, across different papers and collapsed them into the same phenotype. For quantitative data, the following were extracted: effect allele frequency, p-Values, estimates of effect sizes, beta coefficient, odds ratios, confidence intervals, standard errors, whichever was reported. Where possible, beta coefficients and standard errors were converted to odds ratios and confidence intervals respectively. Some SNPs were reported in SNP coordinates from an old human assembly; to obtain the most recent information, all SNP coordinates were lifted over from their original assembly to the hg38 human assembly using UCSC LiftOver, and both rsID and genome coordinates are reported for every SNP to maximise usability of information. Some discrepancy exists between our cleaned dataset and the genes reported in primary literature because new knowledge indicated that the reported SNP belongs to a different gene instead of what had been reported in the primary literature. To ensure that the reassignment of SNPs to genes is sufficiently thorough, all published SNPs were converted to genomic coordinates using UCSC Table Browser, loaded onto a custom track and matched to the most updated gene positions using UCSC Genome Browser on the hg38 human assembly. Some papers report ssID instead of rsID; the genome coordinate of the ssID SNP is first tracked in the old human assembly, lifted over to the hg38 human assembly, then, the new genome coordinate is tracked in the hg38 human assembly to identify the rsID. Both the rsID and hg38 human assembly genome coordinate are reported instead of the ssID and old genome coordinates. Some SNPs are intergenic; the genome coordinate of the SNP is reported together with the closest landmark (e.g., protein coding gene, non-coding gene, splice variant, pseudogene) upstream and downstream of the SNP. Some SNPs are in uncharacterised genes; the Ensembl State ID (ENSG) is reported as no gene symbol has been assigned yet. Some gene symbols change over time; to maximise usability of information, both the current gene symbol and Ensembl Stable ID (ENSG) are reported for every gene.

#### Statistical analysis

SNP-phenotype associations reported in only one cohort were excluded from pooling; the results were still grouped at the phenotype level and reported. The random-effect meta-analysis was performed by first extracting the Odds Ratio (OR) and 95% Confidence Interval (CI) reported in all the studies of interest. For papers with no OR and CI reported, we calculate them using the reported statistical beta and Standard Error (SE) respectively. As most associations in the field were reported based on the additive model (i.e., each copy of the allele changes the OR by a certain amount), in this review, we estimated the effect size based on the additive model; all results from other models (e.g., dominant model, allele model) are not included in the figures as the results from different allele models cannot be directly compared. However, all results from other models are still reported for completeness of information. Meta-analyses were conducted on R with the RStudio interface, using the metafor package^35^. Meta-analysis for each risk factor (i.e., each SNP) was conducted when at least two independent studies or cohorts report an effect size estimate for an association between a skin ageing phenotype of interest and the said SNP. We used the DerSimonian and Laird procedure for random effects meta-analysis^36^ to account for between-study heterogeneity, as what had previously been done^1^. Pooled odds ratios (pOR) were calculated for meta-analysis of ≥ 2 studies or cohorts. To avoid a possible overlap of study populations and to avoid including duplicates in the meta-analytic models, each study was only represented once for a given SNP unless the study reported effect sizes after stratifying their populations. We also avoided including the same entry twice (i.e., duplication) such as when two studies investigated the same phenotype in the same study cohort. In such cases where there could be overlapping study subjects, only the larger study was considered. To assess heterogeneity presented in the pooled risk estimate, we computed the inconsistency index (the I^2^ index). An I^2^ value ≥ 50% and a p-Value < 0.05 was considered statistically significant for heterogeneity. Begg’s funnel plots and Egger’s test were used to assess publication bias; Begg’s funnel plots were only drawn for meta-analysis of ≥ 2 studies or cohorts and Egger’s test was only performed for meta-analysis of ≥ 3 studies or cohorts to estimate asymmetry of data; an Egger’s test p-Value < 0.05 was considered as that publication bias possibly exist.

#### Identifying pleiotropic genes

Pleiotropic genes are genes associated with two or more skin ageing phenotypes. We defined pleiotropy strictly such that a gene is pleiotropic if it is associated with morphologically distinct skin ageing phenotypes (e.g., forehead wrinkles and pigment spots) but not pleiotropic if the associated phenotypes are morphologically similar (e.g., forehead wrinkles and Crow’s feet wrinkles). There are 3703 SNP-phenotype associations that pass GWAS-suggestive significant threshold or better (p-Value < 1E−05). We mapped them to 366 genes and intergenic regions on the hg38 human assembly. Genes containing SNPs associated with two or more morphologically distinct skin ageing phenotypes are treated as pleiotropic genes.

### Gene enrichment analyses

As described in “Identifying skin ageing phenotypes” section, We grouped 56 skin ageing phenotypes into four key phenotypic categories (A, B, C, and D) based on their morphology. Skin ageing genes were stratified into the four categories according to gene-phenotype associations.

Next, we subjected (1) all skin ageing genes, (2) Category A genes, (3) Category B genes, (4) Category C genes, and (5) Category D genes to three different gene enrichment strategies (general enrichment, functional enrichment, and network enrichment). This ensures that the gene enrichment analyses were performed sufficiently thoroughly.

#### General enrichment

General enrichment was performed using the functional profiling feature (g:GOSt) on g:Profiler. Lists of genes identified by their Ensembl Stable ID (ENSG) were queried for enrichment from multiple databases—Gene Ontology (GO), Biological Pathways (KEGG, Reactome, WikiPathways), regulatory motifs in DNA (TRANSFAC (TF), miRTarBase), protein databases (Human Protein Atlas, CORUM), and Human phenotype ontology (HP). Detailed results were retrieved and all enrichments between two or more genes were reported in Supplementary Table S9.

#### Functional enrichment

Functional enrichment was performed using Version 11.5 of the Search Tool for Retrieval of Interacting Genes/Proteins (STRING) database (https://string-db.org/) (Figs. 3, 5, 6). Lists of genes identified by their Ensembl Stable ID (ENSG) were queried for enrichment in *Homo sapiens* with interaction confidence score > 0.4. Proteins that may not physically bind to each other but jointly contribute to a shared function were identified from curated databases and co-expression experiments. Other sources, namely text-mined data (i.e., co-mentions in publications) and co-occurrence of the proteins in different genomes (if any) were also reported. The network is further clustered using the Markov Cluster (MCL) Algorithm—a fast and scalable unsupervised cluster algorithm—with a MCL inflation parameter of 3.

#### Network enrichment

Network enrichment identifies common nodes with high connectivity in the network (i.e., hub proteins). High connectivity among proteins could illuminate key pathways driving the development of key skin ageing phenotypes such as wrinkling and sagging skin.

Network enrichment was performed using Version 2.0.0 of Molecular Complex Detection (MCODE) available as a plug-in of Version 3.9.0 of Cytoscape (National Institute of General Medical Sciences, Bethesda, MD, USA) (Fig. 4). The network data was first exported from STRING to Cytoscape and MCODE plug-in of Cytoscape was employed to identify the functional modules in the system. Locally dense protein–protein interaction (PPI) networks are scored according to their graphical interconnectivity^37^. The parameters used to identify these networks are a degree cutoff of 2, a node density cutoff of 0.2, a K-Core of 2, a maximum depth of 100, ‘Haircut’ turned off, and ‘Fluff’ turned off. A sensitivity analysis was conducted to determine the best node score cutoff among 0.1, 0.2, and 0.3. Results with the best Network Scores are shown and the corresponding node score cutoff is reported. Modules with a Network Score ≥ 4 and nodes ≥ 6 are further analysed for general enrichment. Hub proteins were queried for enrichment from multiple databases—Kyoto Encyclopedia of Genes and Genomes (KEGG) pathway, Gene Ontology (GO), and Transfac (TF) through the g:Profiler gene set analysis toolkit (g:Profiler; https://biit.cs.ut.ee/g:Profiler/gost). The full set of network and general enrichment results including the results at different node score cutoffs and results with Network Scores below 4 are reported in Supplementary Table S10.

### Gene expression omnibus (GEO) repository analysis

GEO Datasets are publicly available gene expression datasets. GEO Datasets in NCBI were retrieved using the following search terms: wrinkle AND Homo sapiens; lentigines AND Homo sapiens; skin cancer AND Homo sapiens; Sun exposure AND skin AND Homo sapiens.

Seven GEO Datasets are retrieved. Four GEO Datasets on changes in aged skin are retrieved; they are GSE85358: Metabolic alterations in aged human skin in vivo (Supplementary Fig. S1)^38^, GSE108674: Expression data in human young and old primary keratinocytes (Supplementary Fig. S2)^39^, GSE44805: Capturing the biological impact of the melanoma susceptibility genes CDKN2A and MC1R in cocultured keratinocytes and melanocytes (Supplementary Fig. S3)^40^, and GSE109778: Comparing gene expression of Lentigo to perilesional normal (Supplementary Fig. S4)^41^. Three GEO Datasets on the risk factors of ageing are retrieved; they are GSE181022: New-born infant skin gene expression: Remarkable differences versus adults (Supplementary Fig. S5)^42^, GSE102676: Transcription factors and stress response gene alterations in human keratinocytes following Solar Simulated Ultraviolet Radiation (Supplementary Fig. S6)^43^, and GSE17046: Analysis of the Infrared-A radiation induced transcriptome of human skin fibroblasts (Supplementary Fig. S7)^44^. We further stratified the ‘chronological age’ and ‘UVR exposure’ risk factors to improve the accuracy of our associations. Specifically, ‘chronological age’ was stratified into ‘aged sun-protected skin’, ‘young adult sun-protected skin’, and ‘infant sun-protected skin’, while ‘UVR’ was stratified into ‘single exposure’, ‘repeated exposure’, and ‘repeated exposure followed by recovery’.

We extracted the raw data and made comparisons across the GEO datasets according to our aims. Comparisons were made between the expression levels in aged skin tissues from older participants and expression levels of the same gene in young skin tissues from younger participants for three datasets (GSE85358, GSE108674 and GSE181022). Comparisons were made between the expression levels in skin tissue exhibiting a particular skin ageing phenotype (e.g., melanoma skin or lentigo skin) and the adjacent normal skin of the same participant for two datasets (GSE44805 and GSE109778). Comparisons were made between cells exposed to a treatment (e.g., UV radiation or infrared radiation) and untreated cells for two datasets (GSE102676 and GSE17046).

The compared data were reported as a ratio of the average expression level in the cases (i.e., aged skin tissue or skin tissue exhibiting skin ageing phenotypes or cells exposed to a treatment) against the average expression level of the reference group (i.e., young skin tissue or adjacent normal skin or untreated cells). Ratios above 1 indicate higher expression levels in the cases group while ratios below 1 indicate lower expression levels in the cases group. Significant (p-Value < 0.05) expression ratios were retrieved for further analysis.

### Consent for publication

All authors have read and consented to the publication of this manuscript.

## Results

### SNPs associated with skin ageing phenotypes: literature search

Forty-four eligible publications were identified during the primary search, secondary search, and the subsequent screening process (Supplementary References and Fig. 1). As the shape of the Beggs’ funnel plots are mostly symmetrical, and Egger’s test p-Value are mostly > 0.05, there does not appear to be obvious publication bias across all phenotypes.

The risk factor studied in this paper is genetics, specifically, we compare the association between the effect allele of a given SNP on a given skin ageing phenotype against the association between the non-effect allele of that SNP on the same phenotype. Pleiotropic SNPs exist (i.e., the effect allele of a SNP (e.g., rs258322) is significantly associated with multiple different skin ageing phenotypes (p-Value < 1E−05). There are 2349 effect SNPs making 3703 SNP-phenotype associations with skin ageing phenotypes at the GWAS-suggestive significance level (p-Value < 1E−05).

### Overview of study characteristics

Study populations originated from 16 countries (Supplementary Table S2). Sample sizes and subject characteristics varied between studies, with samples sizes ranging from 87 females from Japan and Germany only^45^, to 287,137 genetic samples from both genders from the 23andme database^46^. The studies varied in scope, by examining specific races (e.g. Ashkenazi Jews in New York^47^), genders (e.g. Nurses' Health Study and Health Professionals Follow-Up Study^48–50^), countries (e.g. selected towns in Germany—Dortmund, Duisburg, Essen, Gelsenkirchen, Herne, Dülmen, Borken^51,52^) or regions (e.g. selected countries in Latin America—Colombia, Brazil, Chile, Mexico, Peru^14^). The narrowest sample age range reported was 54–55 years^51,52^ and the widest was 18–79 years^53^, with the youngest subject aged 18^53–55^ and the oldest aged 98 years^56^.

### Skin ageing phenotype definition

Most publications investigated one phenotype (e.g., forehead wrinkles). Publications which investigated multiple phenotypes often use different methods to identify different phenotypes. For example, Liu et al*.*^57^ detects three skin ageing phenotypes—wrinkles, facial pigmented spots, and perceived age. ‘Wrinkles’ are detected both by image analysis algorithms and manually assessed using photonumeric scales. ‘Facial pigmented spots’ are detected by image analysis algorithms. ‘Perceived age’ data is collected from manually assessing the facial appearance of participants. The detailed breakdown and definitions used are in Supplementary Table S3. Briefly, nineteen papers defined the phenotype by describing the appearance of the feature^15,33,48,49,52–54,57–68^, three papers employed image analysis algorithms to identify the feature^56,57,69^, fourteen papers identified the feature using photonumeric scales^14,45,47,51,52,57,69–76^, seven papers detected the feature through self-reporting via questionnaires^46,50,67,76–79^, three papers detected the feature through equipment^55,80,81^, two papers studied genomic data collected for a different condition (e.g. the inability to tan) and attempted to use it for their phenotype (e.g., carcinoma)^54,82^, and the way that the feature was defined was not reported in five papers^30,33,61,83,84^.

Overall, most methods of identifying skin ageing phenotypes required visual assessment of the appearance of the skin feature either by one or more dermatologists or the research subject. The presence/absence of the skin feature and its severity are reported through text-based questions (e.g., ‘What type of skin cancer did you have? Please check all that apply.’^50^) or photonumeric grading scales. A few studies^56,57,69^ performed digital image analyses to obtain more objective measures (e.g., wrinkle length, area). There is agreement with Wong and Chew^1^ that even though SCINEXA was validated as a scale which calculates an overall skin aging score from the individual scores of 23 skin ageing signs, papers reported individual phenotype scores, instead of overall skin aging score. As skin ageing is almost always assessed as individual phenotypes, in this review, we examine individual skin ageing phenotypes as proxy measures of overall skin ageing.

### Skin ageing phenotypes

There is currently no complete list of skin ageing phenotypes; the most recent attempt is the Skin Ageing Atlas^26^ a decade ago. Using the method detailed in “Identifying skin ageing phenotypes” section, we are the first to bring together the findings from the Skin Ageing Atlas and multiple other sources (e.g., the 44 GWAS publications in the skin ageing field) to create a comprehensive list of all the ways that skin ageing phenotypes have ever been collected and measured in the skin ageing field (Table 2) across ethnicities worldwide.

Fifty-six skin ageing phenotypes were identified using methods detailed in “Identifying skin ageing phenotypes” section. Next, we group the 56 skin ageing phenotypes into four key phenotypes categories based on their morphology—Category A Phenotype are skin wrinkling and sagging-related phenotypes; Category B Phenotype are skin colour-related phenotypes; Category C Phenotype are skin cancer-related phenotypes; and Category D Phenotype are skin global impression phenotypes. We therefore treat skin ageing as the result of changes to the skin in these four key areas. While the appearance of all 56 phenotypes have been described in literature, SNP-phenotype associations have not been explored for all 56 phenotypes. Supplementary Table S4 contains a detailed breakdown of the Table 2 phenotypes which also appeared in SNP literature.

We observed that while the field has an abundance of SNP-phenotype associations that reach GWAS-suggestive significance level (p-Value < 1E−05) or better in one publication, only 19 SNP-phenotype associations are corroborated by other studies at the same significance level or better. A few publications performed internal validation by having a discovery cohort and validation cohort(s); often with much weaker p-Values in the validation cohort(s).

For instance, rs4785704 is significantly associated with four skin ageing phenotypes—solar elastosis (OR: 1.17 [1.10–1.26], p = 6.46E−06)^72^, perceived skin darkness (OR: 0.79 [0.74–0.86], p = 3.96E−09)^67^, an older perceived age (OR: 2.72 [1.86–3.97], p = 2.64E−07)^57^, and facial pigmented spots—solar lentigines, seborrheic keratosis (OR: 1.15 [1.13–1.17], p = 7.8E−12)^69^. Each of the four studies conducted internal validation in one or more validation cohorts, but none of the validation cohorts show p-Value < 1E−05 statistical significance (solar elastosis—OR: 1.11 [1.00,1.23], p = 0.046. Perceived skin darkness—OR: 0.94 [0.91,0.97], p = 8.91E−05. Older perceived age—OR: 1.58 [0.78–3.21], p = 0.200. Facial pigmented spots—OR: 1.23 [0.97–1.56], p = 0.077). For the former three phenotypes, checks against the three databases (Web of Science, PubMed, Ensembl) also retrieved no GWAS papers on solar elastosis, perceived skin darkness, and an older perceived age other than the three papers cited in this illustration^57,67,72^. For the fourth phenotype, while our literature search retrieved GWAS papers on facial pigmented spots, solar lentigines, and seborrheic keratosis, none of the retrieved papers^14,15,45,51,58,60,61,64–66,70,73^, reported a significant association with rs4785704 other than the sole paper cited in this illustration^69^.

This illustration shows that there is some uncertainty in establishing whether a given SNP is truly associated with skin ageing. The associations made in the discovery cohort are often either less significant in the replication cohort or absent in other studies investigating the same phenotype. We therefore hypothesise that skin ageing phenotypes are not so much the result of individual SNPs but rather, is the overall effect of multiple SNPs on the same gene or intergenic region. We therefore focus on studying the genes associated with each skin ageing phenotype (i.e., Category A, B, C, and D Phenotypes) and how the genes and proteins are interrelated, through enrichment studies and differential gene expression studies.

We quantify the association between the SNPs and the phenotype using Odds Ratios. The additive allele model is the preferred model in the field; out of the 2349 SNPs making 3703 SNP-phenotype associations with skin ageing phenotypes at the GWAS-suggestive significance level (p-Value < 1E−05), the additive allele model accounts for 2073 of these SNPs and 3135 of these SNP-phenotype associations. Hence, the Odds Ratios for all SNPs illustrated in the figures follow the additive allele model.

**Figure 2:**
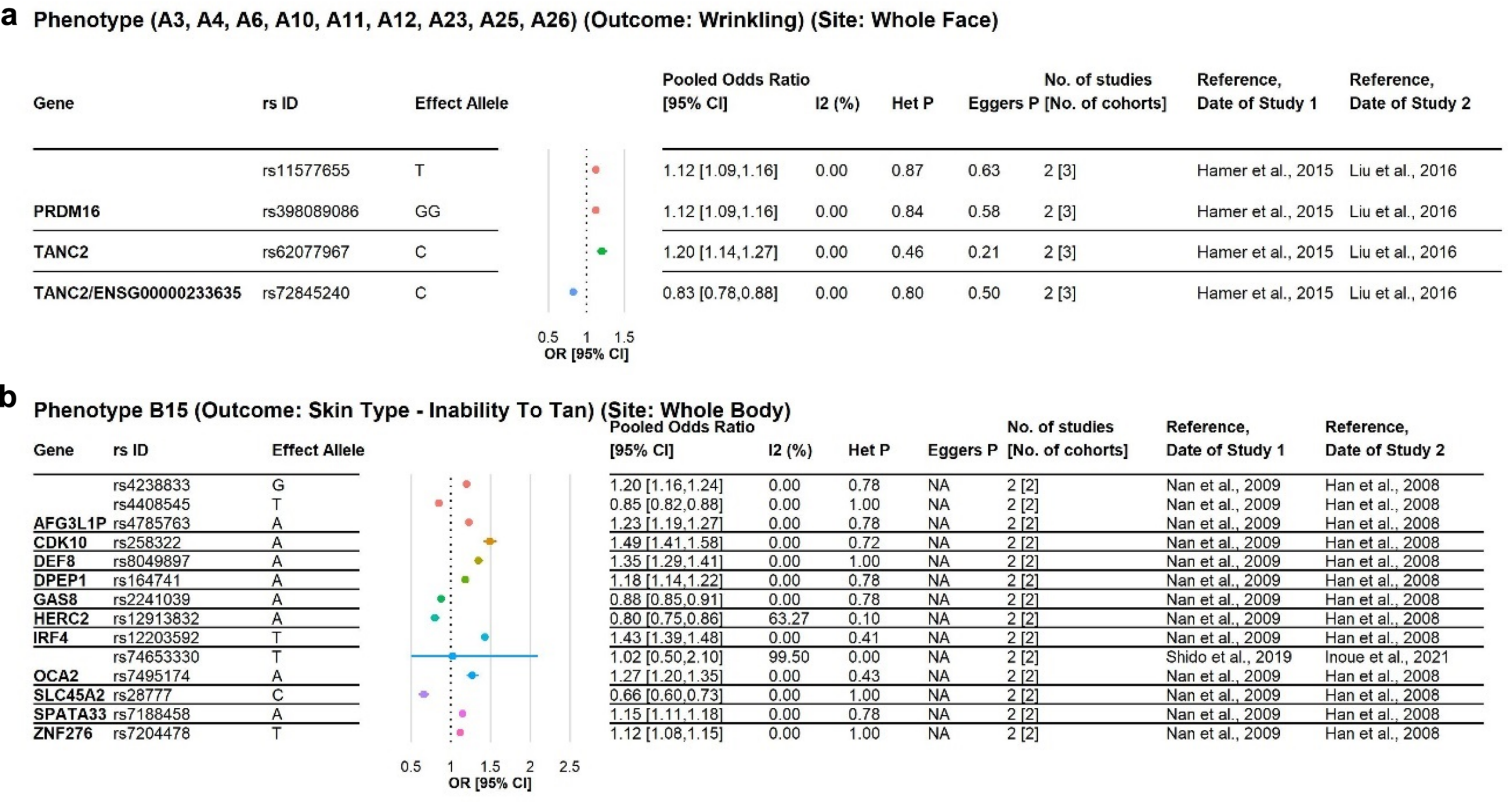

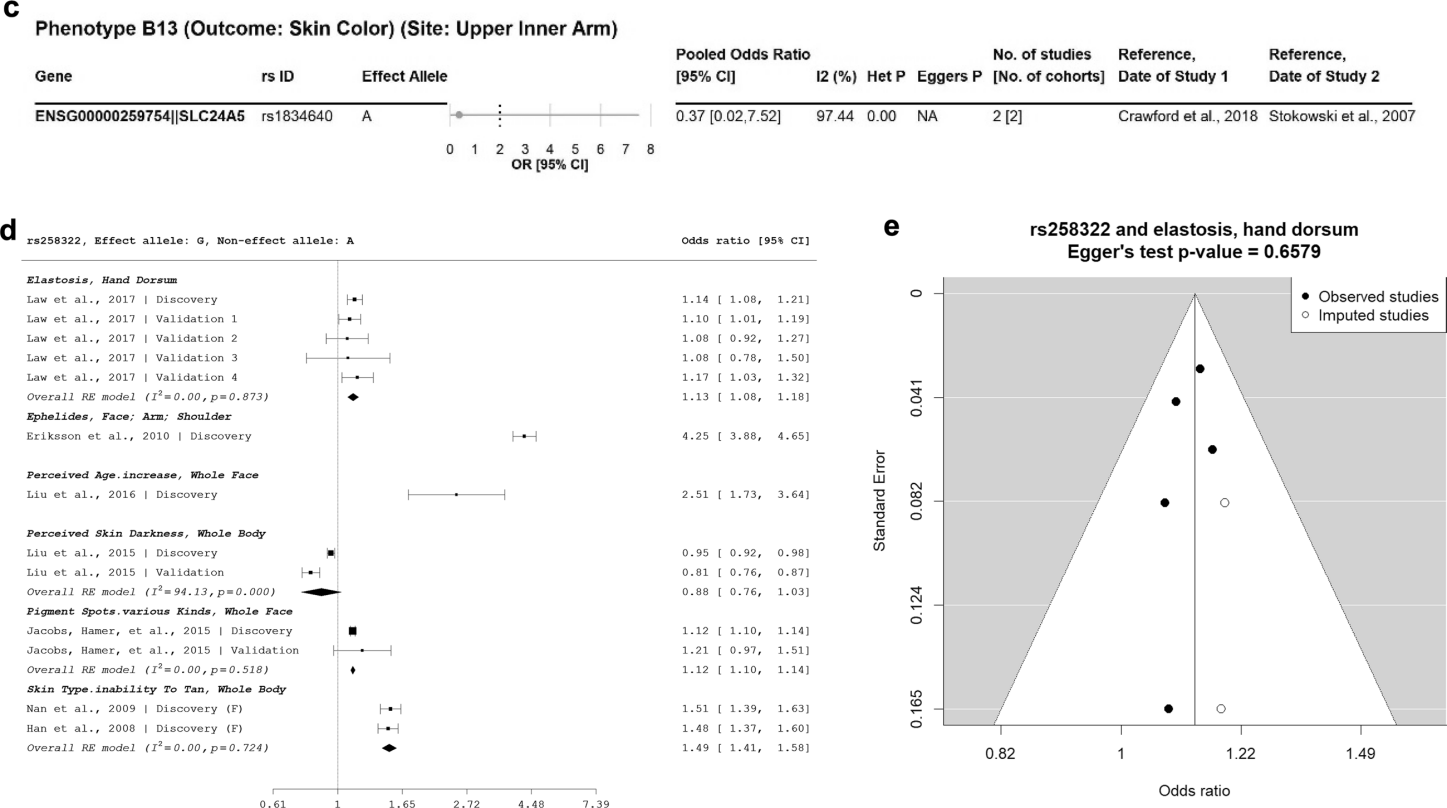
(**a**) SNPs associated with the phenotype: wrinkles, site: whole face. This is a phenotype under Category A Phenotype—skin wrinkling and sagging-related phenotypes. Pooled Odds Ratio and the 95% Confidence Interval are calculated from both studies which reported significant associations between the SNP and the phenotype. An inconsistency index (I^2^ index) value ≥ 50% and a Het p-Value < 0.05 are considered statistically significant for heterogeneity. Begg’s funnel plots and Egger’s test were used to assess publication bias; Begg’s funnel plots were only drawn for meta-analysis of ≥ 2 studies or cohorts and Egger’s test was only performed for meta-analysis of ≥ 3 studies or cohorts to estimate asymmetry of data; an Egger’s test p-Value < 0.05 was considered as that publication bias possibly exist. (**b**) SNPs associated with the phenotype: skin type—inability to tan, site: whole body. This is a phenotype under Category B Phenotype—skin pigmentation-related phenotypes. Pooled Odds Ratio and the 95% Confidence Interval are calculated from both studies which reported significant associations between the SNP and the phenotype. An inconsistency index (I^2^ index) value ≥ 50% and a Het p-Value < 0.05 are considered statistically significant for heterogeneity. Begg’s funnel plots and Egger’s test were used to assess publication bias; Begg’s funnel plots were only drawn for meta-analysis of ≥ 2 studies or cohorts and Egger’s test was only performed for meta-analysis of ≥ 3 studies or cohorts to estimate asymmetry of data; an Egger’s test p-Value < 0.05 was considered as that publication bias possibly exist. (**c**) SNPs associated with the phenotype: skin colour, site: upper inner arm. This is a phenotype under Category B Phenotype—skin pigmentation-related phenotypes. Pooled Odds Ratio and the 95% Confidence Interval are calculated from both studies which reported significant associations between the SNP and the phenotype. An inconsistency index (I^2^ index) value ≥ 50% and a Het p-Value < 0.05 are considered statistically significant for heterogeneity. Begg’s funnel plots and Egger’s test were used to assess publication bias; Begg’s funnel plots were only drawn for meta-analysis of ≥ 2 studies or cohorts and Egger’s test was only performed for meta-analysis of ≥ 3 studies or cohorts to estimate asymmetry of data; an Egger’s test p-Value < 0.05 was considered as that publication bias possibly exist. (**d**) Forest plot for all skin ageing phenotypes associated with rs258322 under the additive allele model. Pooled Odds Ratios (OR) and 95% Confidence Intervals (CI) under the random effects model were computed for each phenotype with ≥ 2 studies or cohorts. Effect alleles were streamlined as allele G; in cases where the primary literature reports the effect allele to be allele A, reciprocals of the OR and CI reported in the primary literature were taken where appropriate to ensure compatibility. (**e**) Begg’s funnel plots and Egger’s test p-Values for rs258322 and skin ageing.

We report, in Fig. 2a–c, the 19 SNP-phenotype associations which are corroborated by other studies at the GWAS-suggestive significance level (p-Value < 1E−05) or better. Other than these 19 SNP-phenotype associations, there are another 1,060 SNP-phenotype associations from publications which performed internal validation by having a discovery cohort and validation cohort(s) (Supplementary Dataset S1) and another 2056 SNP-phenotype associations found at the GWAS-suggestive significance level (p-Value < 1E−05) in only a single publication (Supplementary Dataset S2). All SNP-phenotype associations, regardless of allele model, are reported in Supplementary Table S5. Pooled odds ratios (pOR) were calculated for random effects meta-analysis of ≥ 2 studies or cohorts where appropriate; Fig. 2d illustrates the forest plot for one of the 19 SNPs (rs258322), the rest are reported in Supplementary Dataset S3. Figure 2e illustrates the Begg’s funnel plot for rs258322 and a skin ageing phenotype (solar elastosis on the hand dorsum); the rest are reported in Supplementary Dataset S4.

### Skin ageing genes are pleiotropic

We define pleiotropic skin ageing genes as genes associated with multiple morphologically distinct skin ageing phenotypes (i.e., Phenotypes A, B, C, and D). To the best of our knowledge, we are the first to put forth the idea that pleiotropic skin ageing genes account for the bulk of the genetic associations with skin ageing.

We are also the first to bring together the findings from all 44 GWAS publications in the skin ageing field to create a comprehensive list of genes found to be associated with skin ageing phenotypes across ethnicities worldwide. Being the first to have a comprehensive picture of the skin ageing gene landscape, we seized the opportunity to find relationships and patterns among the skin ageing genes that could not have been discovered by researchers who lack this comprehensive list.

**Figure 3:**
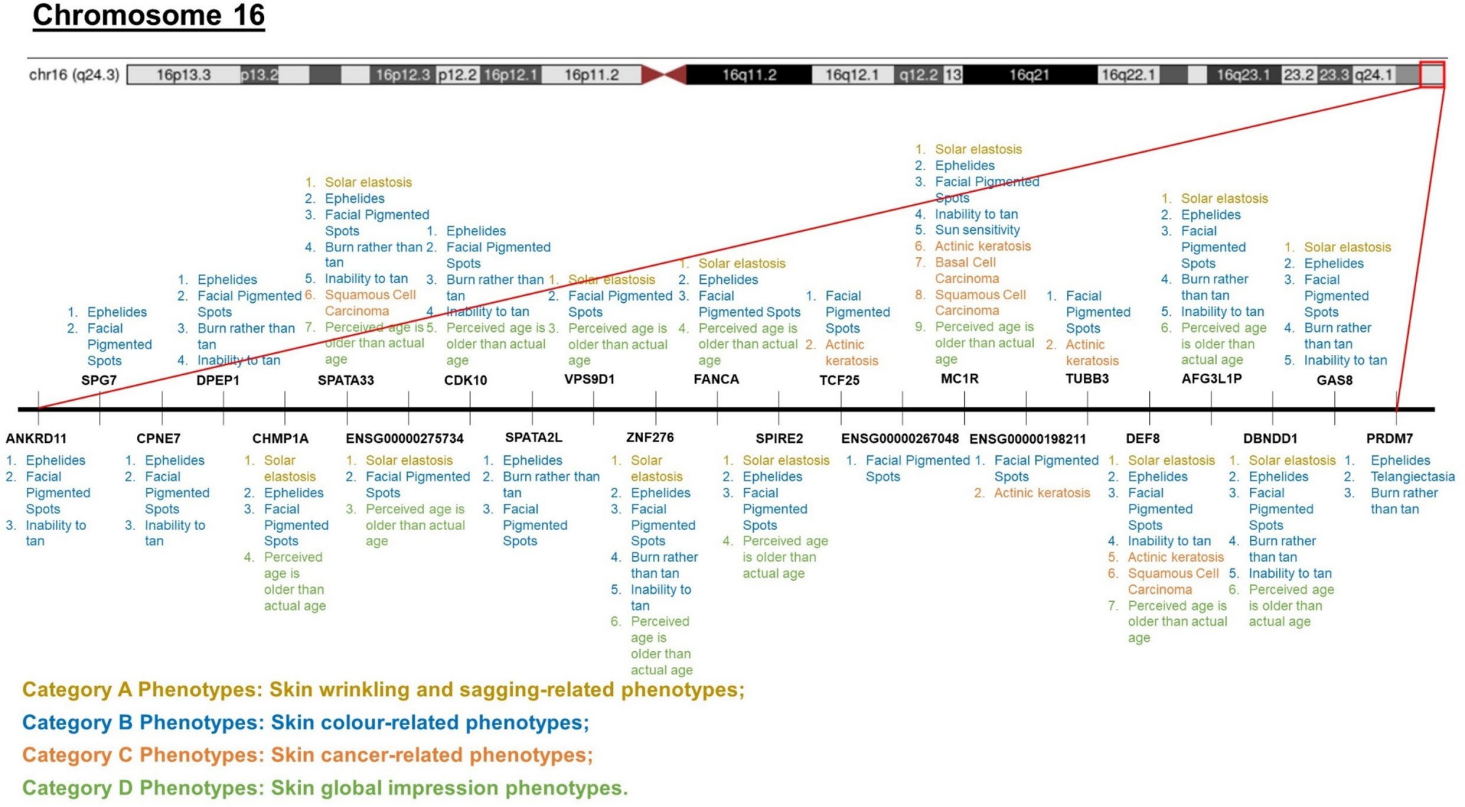
Visual representation of the pleiotropic diversity of genes on the chromosomal band 16q24.3. Each gene shown in this figure is associated with morphologically distinct phenotypes from different phenotypic categories (e.g., Phenotype Category A, B, C, and D colour coded in yellow, blue, orange, and green respectively).

We report that most of the pleiotropic skin ageing genes are skin colour genes, genes on the chromosomal band 16q24.3, and their immediate intergenic neighbourhood (Fig. 3 and Supplementary Table S6). Specifically, the intergenic region between the IRF4 skin colour gene and the gene directly downstream (i.e., ENSG00000286364) is highly pleiotropic. Similarly, the intergenic region between SPATA2L and VPS9D1 on the chromosomal band 16q24.3 is highly pleiotropic. Details on the extent of pleiotropy of skin ageing genes and SNPs can be found in the Supplementary Tables S7 and S8 respectively.

We earlier proposed that (1) genes traditionally identified to contribute to skin colour have more than just skin pigmentation roles, and (2) further progress towards understand the development of skin pigmentation requires understanding the contributions of genes on the chromosomal band 16q24.3. The first proposal is well-illustrated by how the MC1R gene (a skin colour gene) could have more than just skin pigmentation roles (Fig. 3). The second proposal is well-illustrated by the fact that the majority of the 23 genes on the chromosomal band 16q24.3 are associated with skin colour-related phenotypes (colour-coded in blue in Fig. 3) despite only one gene (MC1R) being traditionally identified as a skin colour gene.

Skin colour genes are genes first described for their role in human skin pigmentation. While our understanding of the genetics of skin pigmentation is relatively clear, the genetics for skin wrinkling and sagging is still largely unknown. Recent discoveries propose that skin colour genes are associated with more than just skin colour-related phenotypes^14,63^ and may drive the development of these phenotypes through melanogenesis-independent pathways which are still poorly understood^63^. Our list of pleiotropic skin ageing genes includes many skin colour genes and can guide future work in characterising the melanogenesis-independent pathways in which skin ageing genes operate, which may ultimately explain their association with pigmentation-independent phenotypes such as facial wrinkles.

We report that many skin colour genes are pleiotropic for skin ageing phenotypes: SHC4 is pleiotropic for Category A Phenotypes (skin wrinkling and sagging phenotypes) and Category B Phenotypes (skin colour phenotypes). BNC2, HERC2, OCA2, RALY, and TYR are pleiotropic for Category B Phenotypes (skin colour phenotypes) and Category C Phenotypes (skin cancer phenotypes). IRF4 and SLC45A2 are pleiotropic for Category A Phenotypes (skin wrinkling and sagging phenotypes), Category B Phenotypes (skin colour phenotypes), and Category C Phenotypes (skin cancer phenotypes). MC1R is pleiotropic for Category A Phenotypes (skin wrinkling and sagging phenotype), Category B Phenotypes (skin colour phenotype), Category C Phenotypes (skin cancer phenotype), and Category D Phenotypes (skin global impression phenotypes).

Skin colour and Category B Phenotypes (skin colour phenotypes) are dependent on melanin synthesis by tyrosinase. One form of melanin, phaeomelanin, generates free radicals in response to ultraviolet radiation that in turn, damages the skin^85^. It is possible that some non-skin colour phenotypes (e.g., skin wrinkling and sagging) may result from the skin damage sustained through this process.

### Enrichment analyses

We scrutinised the dataset using three different enrichment analyses—general enrichment (Supplementary Table S9), functional enrichment, and network enrichment (Supplementary Table S10). All three enrichment strategies revealed several novel and coherent results which offer different perspectives to the same story.

**Figure 4:**
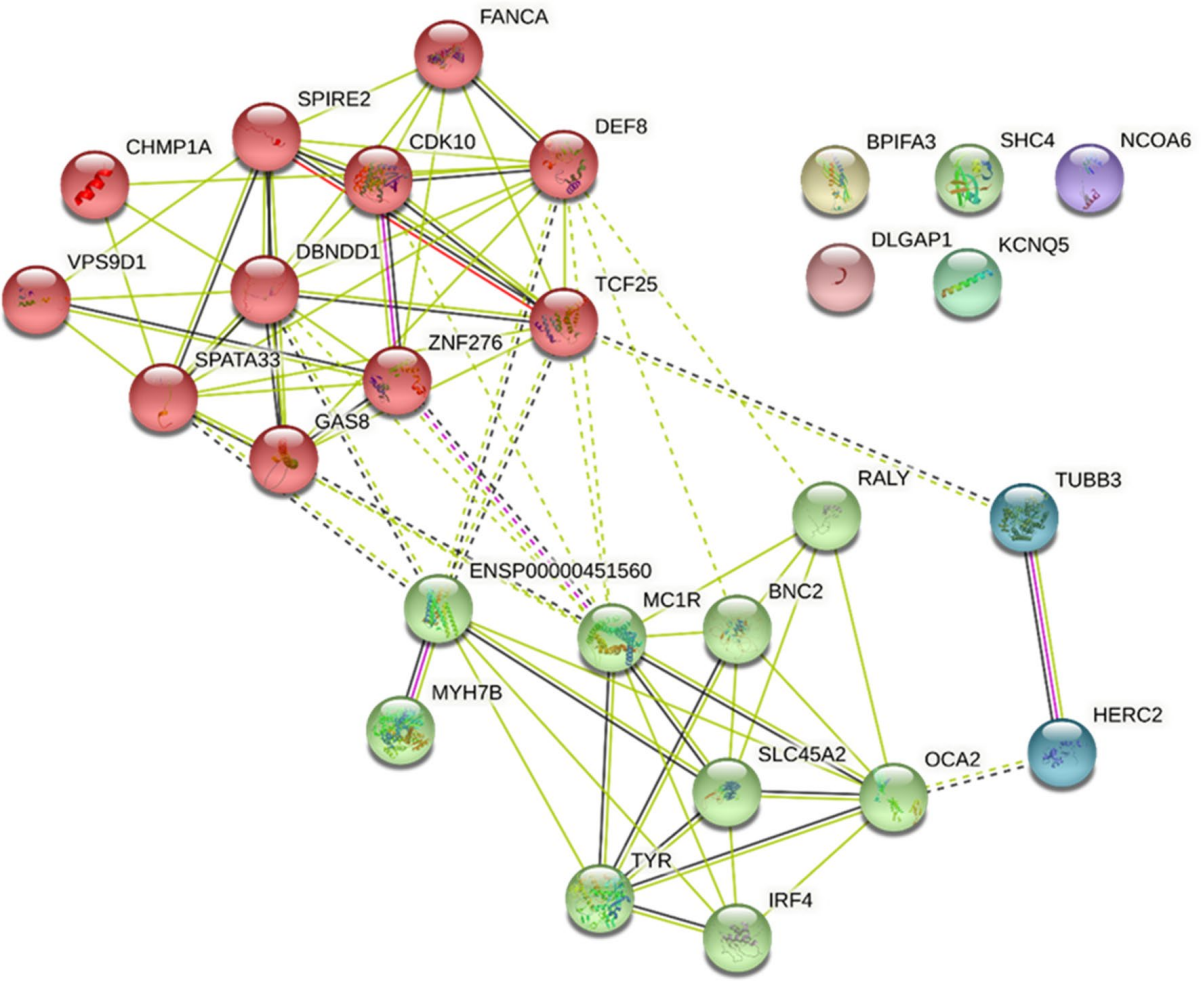
Functional enrichment analysis of skin ageing genes. Each gene shown in this figure is associated with two or more morphologically distinct phenotypes from different phenotypic categories (e.g., Phenotype Category A, B, C, and D). Nodes coloured red represent genes on the chromosomal band 16q24.3 and nodes coloured green represent skin colour genes. Edges (i.e., lines) linking the node indicate evidence of enrichment between the two connected nodes.

**Figure 5:**
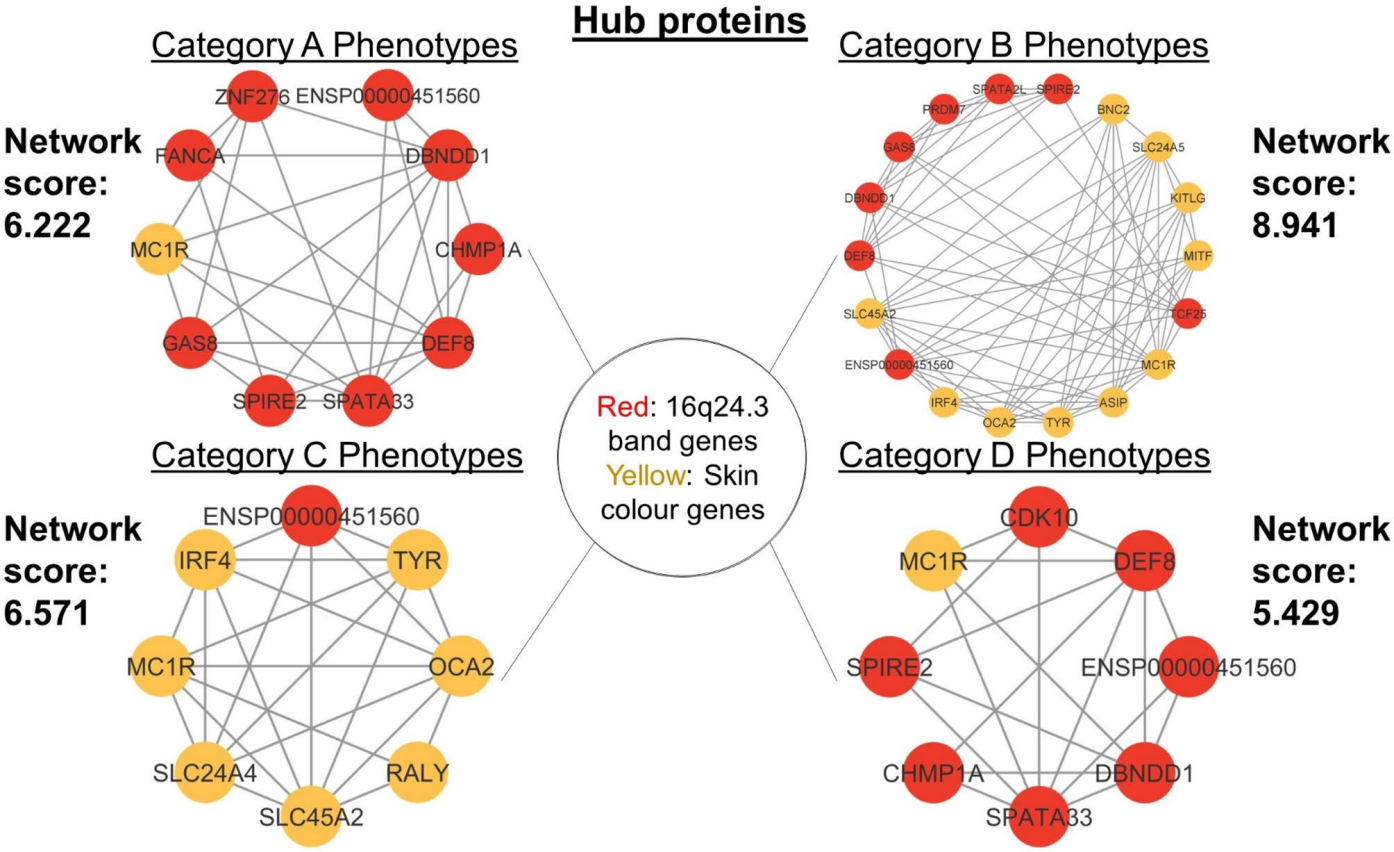
Network enrichment of skin ageing hub proteins. Only one strong network (i.e., Network score ≥ 4) is identified for each key skin ageing phenotype. Red gene nodes are genes on the chromosomal band 16q24.3 and yellow gene nodes are skin colour genes. Edges linking the node indicate evidence of enrichment between the two connected nodes.

Firstly, many skin ageing phenotypes are associated with a small handful of 44 genes. These 44 genes are (1) associated with morphologically diverse skin ageing phenotypes (i.e., each of them is a pleiotropic skin ageing gene) (Fig. 4 and Supplementary Table S6) or (2) code for hub proteins: highly interconnected proteins in the gene network whose frequent interactions could illuminate relevant pathways driving the development of key skin ageing phenotypes (Fig. 5), or (3) are pleiotropic genes coding for hub proteins (Supplementary Table S11).

**Figure 6:**
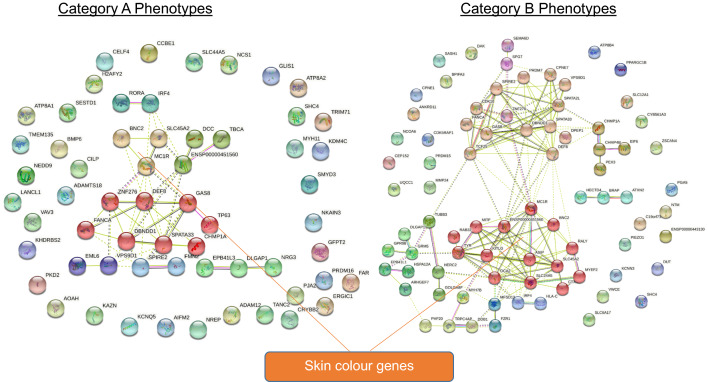
Functional enrichment of skin ageing genes reveals that skin colour genes are pleiotropic for morphologically diverse (Category A and B) skin ageing phenotypes.

**Figure 7:**
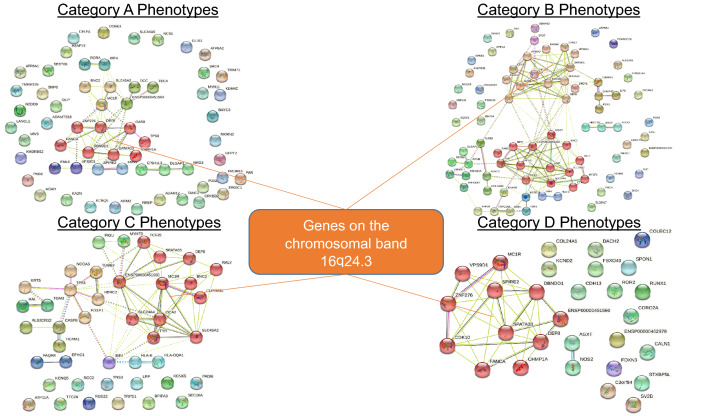
Functional enrichment of skin ageing genes reveals that genes on the chromosomal band 16q24.3 are pleiotropic for morphologically diverse (Category A, B, C, and D) skin ageing phenotypes.

Secondly, most of the pleiotropic and hub genes fall into one of two categories—skin colour genes (Fig. 6), or genes on the chromosomal band 16q24.3 (Fig. 7). This gives two immediate implications: First, genes traditionally identified to contribute to skin colour take on more than just skin pigmentation roles, and second, to truly understand the development of skin pigmentation, we must also account for the contributions by genes on the chromosomal band 16q24.3.

Realising the important role that skin colour genes and genes on the chromosomal band 16q24.3 contribute to understanding many diverse skin ageing phenotypes, we searched the literature to obtain a thorough list of 32 skin colour genes (Supplementary Table S12). This list contains traditionally well-studied skin colour genes (e.g., BNC2), and less well-studied skin colour genes (e.g., KIAA0930)^52^. However, we expect that there will be even more skin colour genes because we discovered that many genes on the chromosomal band 16q24.3 are associated with skin pigmentation phenotypes but are not yet classified under skin colour genes by the larger community. Hence, to be as thorough as possible, we paid close attention to our established list of 44 pleiotropic skin ageing genes and 32 skin colour genes.

### Gene expression levels change with age

#### Defining case and control groups

Aged skin and young skin have different expression levels in the pleiotropic skin ageing genes, hub genes, and skin colour genes. We retrieve this expression data from the Gene Expression Omnibus (GEO) repository and represent the data as a ratio of the average expression level in the cases (e.g., aged skin tissue or skin tissue exhibiting skin ageing phenotypes or cells exposed to a treatment) against the average expression level of the reference group (i.e., young skin tissue or adjacent normal skin or untreated cells). Ratios above 1 indicate higher expression levels in the cases group while ratios below 1 indicate lower expression levels in the cases group. Expression ratios of pleiotropic skin ageing genes, hub genes, and skin colour genes are reported in Tables 3, 4, 5, S11, S12, and Supplementary Figs. S1–S7.

**Table 3:** Differentially expressed pleiotropic and hub genes associated with chronological age.

Pleiotropic and/or hub gene	GEO dataset study ID^a^	Ratio definition^b^	Expression ratio	p-value
BNC2	GSE181022	Non-infant/infant	0.90	3.58E−03
	GSE85358	Naturally aged skin/young skin	0.98	5.57E−03
ENSG00000198211	GSE181022	Old age/young adult	0.93	3.18E−02
	GSE85358	Naturally aged skin/young skin	0.96	8.10E−03
MC1R	GSE181022	Old age/young adult	0.96	1.23E−02
	GSE44805	Melanoma skin/non-melanoma skin	0.89	1.13E−02
SHC4	GSE181022	Non-infant/infant	0.90	4.58E−08
	GSE44805	Melanoma skin/non-melanoma skin	0.70	2.07E−03
SLC24A5	GSE181022	Non-infant/infant	0.91	1.53E−02
	GSE44805	Melanoma skin/non-melanoma skin	0.37	3.77E−03
SPIRE2	GSE181022	Non-infant/infant	0.98	5.69E−03
	GSE85358	Naturally aged skin/young skin	0.96	1.82E−02
	GSE44805	Melanoma skin/non-melanoma skin	0.77	4.84E−02
TYR	GSE181022	Non-infant/infant	0.74	6.63E−03
	GSE44805	Melanoma skin/non-melanoma skin	0.39	1.65E−08

^a^GSE181022 controls for Sun exposure by using only sun-protected skin. The skin tissue in GSE85358 is skin aged normally, which will typically be exposed concurrently to numerous factors (e.g., chronological age and sun exposure).

^b^The expression ratio is the average expression level in the aged tissue or tissue with skin ageing phenotypes (i.e., cases) divided by (/) the average expression level in the young tissue or tissue without skin ageing phenotypes (i.e., reference). Ratios above 1 indicate higher expression levels in the cases group while ratios below 1 indicate lower expression levels in the cases group.

**Table 4:** Differentially expressed skin colour genes associated with chronological age.

Skin ageing gene	GEO dataset study ID^a^	Ratio definition^b^	Expression ratio	p-Value
AGR3	GSE181022	Non-infant/infant	1.17	8.71E−09
	GSE85358	Naturally aged skin/young skin	1.15	3.47E−04
BNC2	GSE181022	Non-infant/infant	0.90	3.58E−03
	GSE85358	Naturally aged skin/young skin	0.98	5.57E−03
DSTYK	GSE181022	Non-infant/infant	1.21	3.75E−05
	GSE102676	Solar stimulated UV radiation/untreated	1.12	5.78E−03
	GSE85358	Naturally aged skin/young skin	1.03	2.86E−02
EDNRB	GSE181022	Non-infant/infant	0.69	6.27E−16
	GSE44805	Melanoma skin/non-melanoma skin	0.56	6.94E−03
MC1R	GSE181022	Old age/young adult	0.96	1.23E−02
	GSE44805	Melanoma skin/non-melanoma skin	0.89	1.13E−02
SHC4	GSE181022	Non-infant/infant	0.90	4.58E−08
	GSE44805	Melanoma skin/non-melanoma skin	0.70	2.07E−03
TPCN2	GSE181022	Non-infant/infant	1.05	1.24E−05
	GSE102676	Solar stimulated UV radiation/untreated	1.21	3.98E−02
	GSE85358	Naturally aged skin/young skin	1.02	1.89E−02
TYR	GSE181022	Non-infant/infant	0.74	6.63E−03
	GSE44805	Melanoma skin/non-melanoma skin	0.39	1.65E−08

^a^GSE181022 controls for sun exposure by using only sun-protected skin. The skin tissue in GSE85358 is skin aged normally, which will typically be exposed concurrently to numerous factors (e.g., chronological age and sun exposure).

^b^The expression ratio is the average expression level in the aged tissue or tissue with skin ageing phenotypes (i.e., cases) divided by (/) the average expression level in the young tissue or tissue without skin ageing phenotypes (i.e., reference). Ratios above 1 indicate higher expression levels in the cases group while ratios below 1 indicate lower expression levels in the cases group.

**Table 5:** Differentially expressed pleiotropic and hub genes associated with UV radiation exposure.

Pleiotropic and hub gene	GEO dataset study ID^a^	Ratio definition^b^	Expression ratio	p-value^c^
DBNDD1	GSE102676	Five repeat exposures to solar stimulated UV radiation/untreated	0.65	**4.10E−02**
	GSE44805	Melanoma skin/non-melanoma skin	0.84	**2.10E−02**
	GSE181022	Non-infant/infant	1.02	7.82E−02
	GSE181022	Old age/young adult	0.99	4.64E−01

^a^GSE181022 controls for sun exposure by using only Sun-protected skin. The skin tissue in GSE85358 is skin aged normally, which will typically be exposed concurrently to numerous factors (e.g., chronological age and sun exposure).

^b^The expression ratio is the average expression level in the aged tissue or tissue with skin ageing phenotypes (i.e., cases) divided by (/) the average expression level in the young tissue or tissue without skin ageing phenotypes (i.e., reference). Ratios above 1 indicate higher expression levels in the cases group while ratios below 1 indicate lower expression levels in the cases group.

^c^Significant values (p-Value < 0.05) are bolded.

As described in “Gene expression omnibus (GEO) repository analysis” section, we extracted the raw data and made relevant comparisons across the GEO datasets. Firstly, among the four GEO datasets on skin ageing phenotypes, three of them (GSE85358, GSE108674, and GSE44805) have significantly different expression levels between aged skin and young skin and these results will be further discussed. Secondly, among the three GEO datasets used to study various risk factors of skin ageing, two of them (GSE181022 and GSE102676) have significantly different expression levels between skin exposed to the risk factor and skin unexposed to the risk factor and these results will be further discussed in “Chronological ageing is associated with the downregulation of BNC2, ENSG00000198211, MC1R, SHC4, SLC24A5, SPIRE2, and TYR in the aged skin”, “Chronological ageing is associated with the upregulation of AGR3, DSTYK, and TPCN2, and the downregulation of BNC2, EDNRB, MC1R, SHC4, and TYR in the aged skin”, and “UV radiation exposure is associated with the downregulation of DBNDD1 in the aged skin” sections.

#### Chronological ageing is associated with the downregulation of BNC2, ENSG00000198211, MC1R, SHC4, SLC24A5, SPIRE2, and TYR in the aged skin

**Figure 8:**
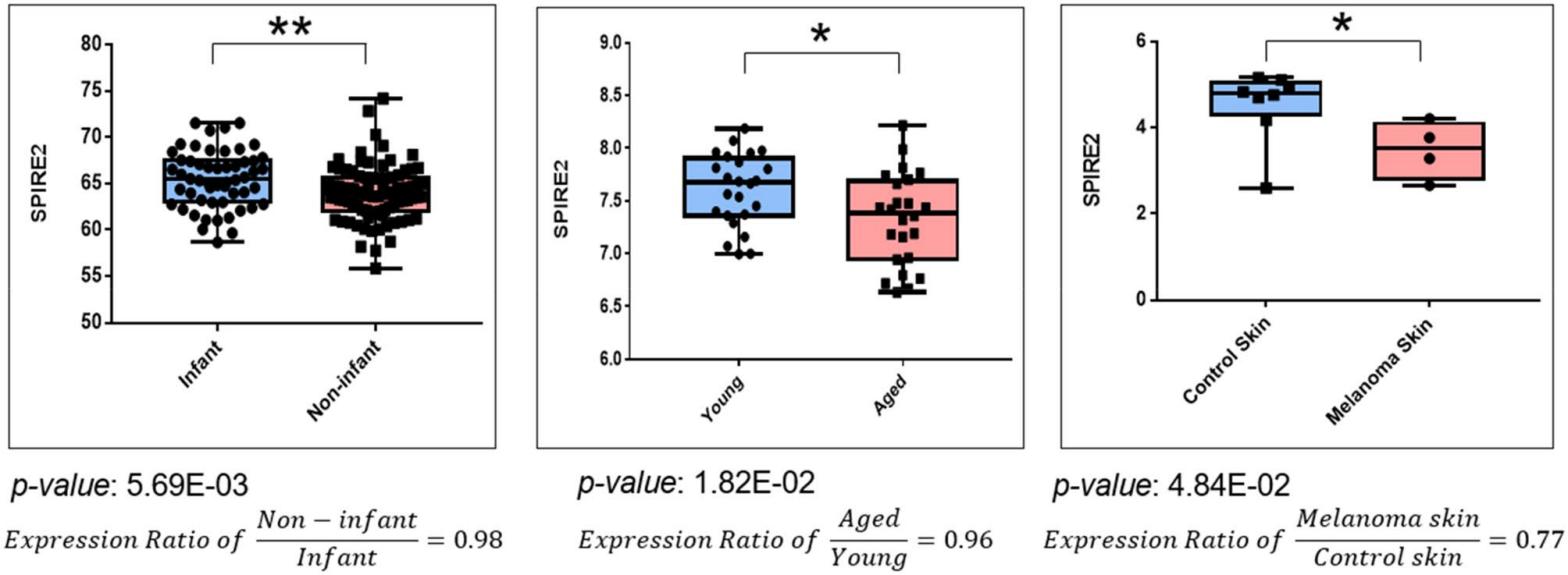
Gene expression of the pleiotropic and hub gene SPIRE2 is downregulated in non-infant skin, aged skin, and melanoma skin, when compared against expression levels in infant skin, young skin, and non-melanoma control skin respectively. Melanoma is a skin ageing phenotype. Expression ratio is the ratio of the average expression level in the cases (e.g., aged skin tissue) against the average expression level of the reference group (e.g., young skin tissue). Ratios above 1 indicate higher expression levels in the cases group while ratios below 1 indicate lower expression levels in the cases group.

In this section, we analyse gene expression level changes to our established list of 44 pleiotropic and hub genes. Seven GEO Datasets relevant to our study aims were consulted. Genes with significantly different (p-Value < 0.05) expression levels between case and control groups in ≥ 2 GEO Datasets are reported in Table 3 and illustrated using SPIRE2 in Fig. 8. The full set of results are in Supplementary Table S11.

BNC2 (GSE85358, Expression Ratio = 0.98, p-Value = 5.57E−03) and ENSG00000198211 (GSE85358, Expression Ratio = 0.96, p-Value = 8.10E−03) are downregulated in aged skin tissue. This finding is coherent with study GSE181022 which found that BNC2 (Expression Ratio = 0.90, p-Value = 3.58E−03) is downregulated in Sun-protected non-infant skin tissue as compared to infant skin tissue, and that ENSG00000198211 (Expression Ratio = 0.93, p-Value = 3.18E−02) is downregulated in Sun-protected young adult skin tissue as compared to aged skin tissue. This suggests that chronological ageing from infant to old age is associated with the downregulation of BNC2 observed in aged skin and that chronological ageing from young adult to old age is associated with the downregulation of ENSG00000198211 observed in aged skin.

Melanoma is a skin ageing phenotype. Study GSE44805 reports that SHC4 (Expression Ratio = 0.70, p-Value = 2.07E−03), SLC24A5 (Expression Ratio = 0.37, p-Value = 3.77E−03), TYR (Expression Ratio = 0.39, p-Value = 1.65E−08), and MC1R (Expression Ratio = 0.89, p-Value = 1.13E−02) are downregulated in melanoma skin relative to adjacent normal skin. This finding is coherent with study GSE181022 which found that SHC4 (Expression Ratio = 0.90, p-Value = 4.58E−08), SLC24A5 (Expression Ratio = 0.91, p-Value = 1.53E−02), and TYR (Expression Ratio = 0.74, p-Value = 6.63E−03) are also downregulated in Sun-protected non-infant skin tissue as compared to infant skin tissue and MC1R (Expression Ratio = 0.96, p-Value = 1.23E−02) is also downregulated in Sun-protected young adult skin tissue as compared to aged skin tissue. This suggests that chronological ageing from infant to old age is associated with the downregulation of SHC4, SLC24A5, and TYR observed in aged skin and that chronological ageing from young adult to old age is associated with the downregulation of MC1R observed in aged skin.

SPIRE2 (Fig. 8) is both downregulated in aged skin tissue (GSE85358, Expression Ratio = 0.96, p-Value = 1.82E−02) and in melanoma skin relative to adjacent normal skin (GSE44805, Expression Ratio = 0.77, p-Value = 4.84E−02). This finding is coherent with study GSE181022 which found that SPIRE2 is also downregulated in Sun-protected non-infant skin tissue as compared to infant skin tissue (Expression Ratio = 0.98, p-Value = 5.69E−03). This suggests that chronological ageing from infant to old age is associated with the downregulation of SPIRE2 observed in aged skin.

#### Chronological ageing is associated with the upregulation of AGR3, DSTYK, and TPCN2, and the downregulation of BNC2, EDNRB, MC1R, SHC4, and TYR in the aged skin

In this section, we analyse gene expression level changes to our established list of 32 skin colour genes. Seven GEO Datasets relevant to our study aims were consulted. Genes with significantly different (p-Value < 0.05) expression levels between case and control groups in ≥ 2 GEO Datasets are reported in Table 4 and the full set of results are in Supplementary Table S12.

The downregulation of BNC2, MC1R, SHC4, and TYR have been explored in the previous section as they are also pleiotropic genes and/or hub genes in addition to being skin colour genes. Melanoma is a skin ageing phenotype; EDNRB is downregulated in melanoma skin relative to adjacent normal skin (GSE44805, Expression Ratio = 0.56, p-Value = 6.94E−03). This finding is coherent with study GSE181022 which found that EDNRB is downregulated in Sun-protected non-infant skin tissue as compared to infant skin tissue (GSE181022, Expression Ratio = 0.69, p-Value = 6.27E−16). This suggests that chronological ageing from infant to old age is associated with the downregulation of EDNRB observed in aged skin.

AGR3 (GSE85358, Expression Ratio = 1.15, p-Value = 3.47E−04) is upregulated in aged skin tissue. This finding is coherent with study GSE181022 which found that AGR3 (Expression Ratio = 1.17, p-Value = 8.71E−09) is upregulated in Sun-protected non-infant skin tissue as compared to infant skin tissue. This suggests that chronological ageing from infant to old age is associated with the upregulation of AGR3 observed in aged skin.

DSTYK (GSE85358, Expression Ratio = 1.03, p-Value = 2.86E−02) and TPCN2 (GSE85358, Expression Ratio = 1.02, p-Value = 1.89E−02) are upregulated in aged skin tissue. These findings are coherent with study GSE181022 which found that DSTYK (Expression Ratio = 1.21, p-Value = 3.75E−05) and TPCN2 (Expression Ratio = 1.05, p-Value = 1.24E−05) are upregulated in Sun-protected non-infant skin tissue as compared to infant skin tissue. Additionally, study GSE102676 found that DSTYK (Expression Ratio = 1.12, p-Value = 5.78E−03) and TPCN2 (Expression Ratio = 1.21, p-Value = 3.98E−02) are also upregulated in HaCaT keratinocyte cells exposed to a single dose of 12 J/cm^2^ solar stimulated UVR. These suggest that both chronological ageing from infant to old age and extrinsic ageing from UV exposure from the Sun are associated with the upregulation of DSTYK and TPCN2 observed in aged skin.

#### UV radiation exposure is associated with the downregulation of DBNDD1 in the aged skin

Chronological age is not the only risk factor for skin ageing. Other risk factors include UV radiation exposure (Table 5). DBNDD1 is both a pleiotropic gene and a hub gene. DBNDD1 is significantly associated with UV radiation exposure, not chronological ageing (Table 5). Melanoma is a skin ageing phenotype; DBNDD1 is downregulated in melanoma skin relative to adjacent normal skin (GSE44805, Expression Ratio = 0.84, p-Value = 2.10E−02). This finding is coherent with study GSE102676 which found that DBNDD1 is also downregulated in HaCaT keratinocyte cells exposed to 5 doses of 12 J/cm^2^ solar stimulated UVR followed by a 1-week recovery period (Expression Ratio = 0.65, p-Value = 4.10E−02). There are no significant associations between Sun-protected infant, young adult, or aged skin tissue (p-Value for comparison between infant and non-infant = 7.82E−02, p-Value for comparison between young adult and old age = 4.64E−01). This suggests that even with a sufficient recovery period, short periods of frequent UVR exposure, not chronological age, is associated with the downregulation of DBNDD1 observed in aged skin.

## Limitations

While there is a diversity in assessment methods of skin ageing phenotypes across all papers, the bulk of them still rely on visual assessment methods.

On one hand, image analysis algorithms such as MATLAB used in some studies^57^ would provide superior analysis capabilities because skin ageing features will be indiscriminately detected and will have less bias. Nonetheless, these image analysis algorithms have technical challenges of its own; already, one paper reported difficulties in separating one type of facial pigmented lesions (solar lentigines) from many other types (seborrheic keratosis, melanocytic nevi, freckles, melasma) using their algorithm^69^. Furthermore, obtaining the high-resolution digital photographs required for an accurate analysis is challenging and may require specialised equipment, making it difficult to be recommended by default.

On the other hand, visual assessment methods are cost-effective but are inherently subjective and can be influenced by cultural perceptions, for instance, Chinese women tend to underestimate the age of Chinese faces while overestimating the age of Caucasian faces^86^.

Often, the association between a given SNP and a given skin ageing phenotype could not be replicated. This could be caused by several reasons, some of which are listed below: (1) many skin ageing phenotypes are understudied, or (2) the heritability of the SNP differs in different populations in different environments, or (3) skin ageing is the cumulative effect of multiple SNPs of the same gene.

## Conclusion

There is currently no consensus on the definition of skin ageing, no comprehensive list of skin ageing phenotypes, and no clear ways to identify them. Such a list is important for an overview of the morphologies in which skin ageing phenotypes manifest and to identify potential associations among different phenotypes. We are the first to create this comprehensive list of skin ageing phenotypes. Creating this list was challenging due to two reasons. Firstly, there are multiple discrepancies in the phenotypes that different researchers consider important to measure, and secondly, there are over 100 scales of different methodological qualities claiming to quantify the same item (e.g., forehead wrinkles). To achieve reliable data, we only selected the best photonumeric grading scales (i.e., the scales with high methodology, high performance, and focused on interrater reliability).

To the best of our knowledge, nobody in the field has compiled a list of skin ageing genes. We brought together the findings from all 44 GWAS publications in the field to create a comprehensive list of genes found to be associated with skin ageing phenotypes. Being the first to have a detailed picture of the skin ageing gene landscape, we found relationships and patterns among the skin ageing genes that could not have been discovered by researchers who lack this comprehensive list.

Through three different enrichment analyses (general enrichment, functional enrichment, and network enrichment) and gene expression data from GEO Datasets, we discovered four new findings: (1) pleiotropy is a recurring theme among skin ageing genes, (2) SNPs associated with skin ageing phenotypes are mostly found in a small handful of 44 pleiotropic and hub genes and 32 skin colour genes, (3) various gene expression studies paint a coherent picture that most of these genes are down- or up-regulated with chronological age, and (4) most skin ageing genes are spatially confined within chromosomal band 16q24.3, or are skin colour genes elsewhere in the genome.

As a result, we propose two important implications. First, genes traditionally identified to contribute to skin colour take on more than just skin pigmentation roles, and second, to truly understand the development of skin pigmentation, we must also account for the contributions by genes on the chromosomal band 16q24.3.

Specifically, through our systematic review we identified 19 promising SNPs which are found to be significantly (p-Value < 1E−05) associated with skin ageing phenotypes in two or more independent studies. Multiple lines of evidence (e.g., SNP, gene expression levels, pleiotropy, enrichment) were employed to support and strengthen the association between a named gene (e.g., SPIRE2) and skin ageing.

Finally, this systematic review acts as a hub to locate primary literature sources pertaining to the genetics of skin ageing and offers new perspectives (i.e., skin ageing genes are pleiotropic and most of which are either spatially confined within chromosomal band 16q24.3 or are skin colour genes elsewhere on the genome) to understanding skin ageing as a condition with multiple morphologically distinct but nonetheless related phenotypes.

Future work will examine skin ageing phenotypes more closely and explore environmental risk factors in Han Chinese skin. Moreover, since many skin colour genes are pleiotropic, future work in characterising the melanogenesis-independent pathways in which skin ageing genes operate may ultimately explain their association with pigmentation-independent phenotypes such as facial wrinkles.

## Supplementary Information

Supplementary Information 1.

Supplementary Information 2.

## Data Availability

Supplementary Information is available for this paper. Correspondence and requests for materials should be addressed to F.T.C. Reprints and permissions information is available at http://www.nature.com/reprints. The forty-four publications selected for the review were identified through the primary search or the secondary search and remained relevant to the aims of this review after a subsequent screening process. The publications are reported in Supplementary References. All 3703 SNP-phenotype associations with skin ageing phenotypes at the GWAS-suggestive significance level (p-Value < 1E−05), 1,079 Begg’s funnel plots, and Egger’s test used to assess publication bias, and Odds Ratios are documented in Supplementary Table S5, Supplementary Datasets S1, and S2 respectively. A review protocol was not prepared prior to the writing of this manuscript but the review methodology follows what was described in “Methodology” section.
