# Supplementary figures and images for: A systematic review of skin ageing genes: gene pleiotropy and genes on the chromosomal band 16q24.3 may drive skin ageing (part 2 of 3)
Source: Sci Rep. 2022 Jul 30;12:13099. doi: 10.1038/s41598-022-17443-1 (PMC9338925; doi:10.1038/s41598-022-17443-1)

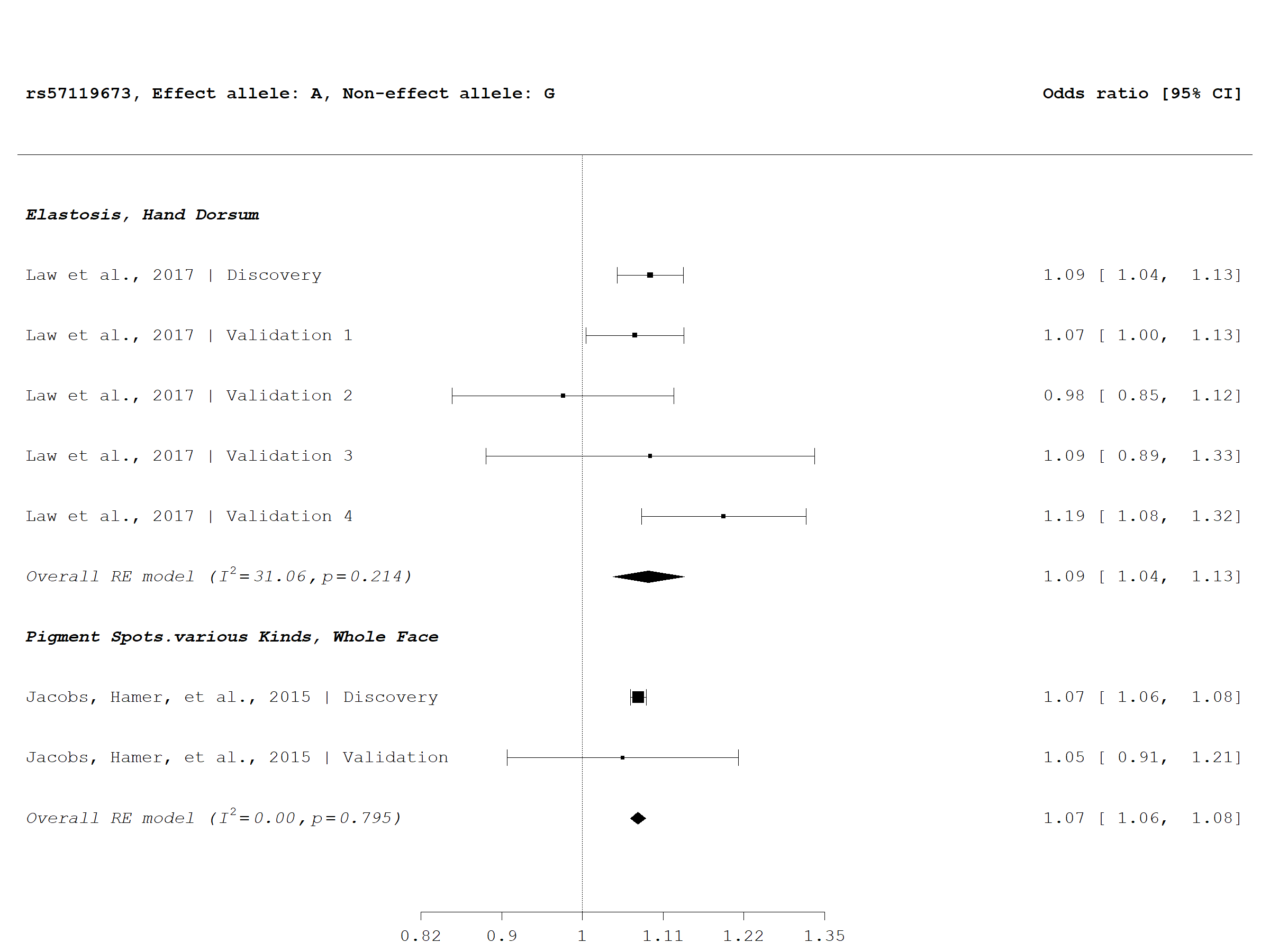

Supplement: Supplementary file 1 — Supplementary Information 1. [file 41598_2022_17443_MOESM1_ESM.zip › Supplementary Datasets/Dataset S3 - Forest Plots/fp116_rs57119673.png]

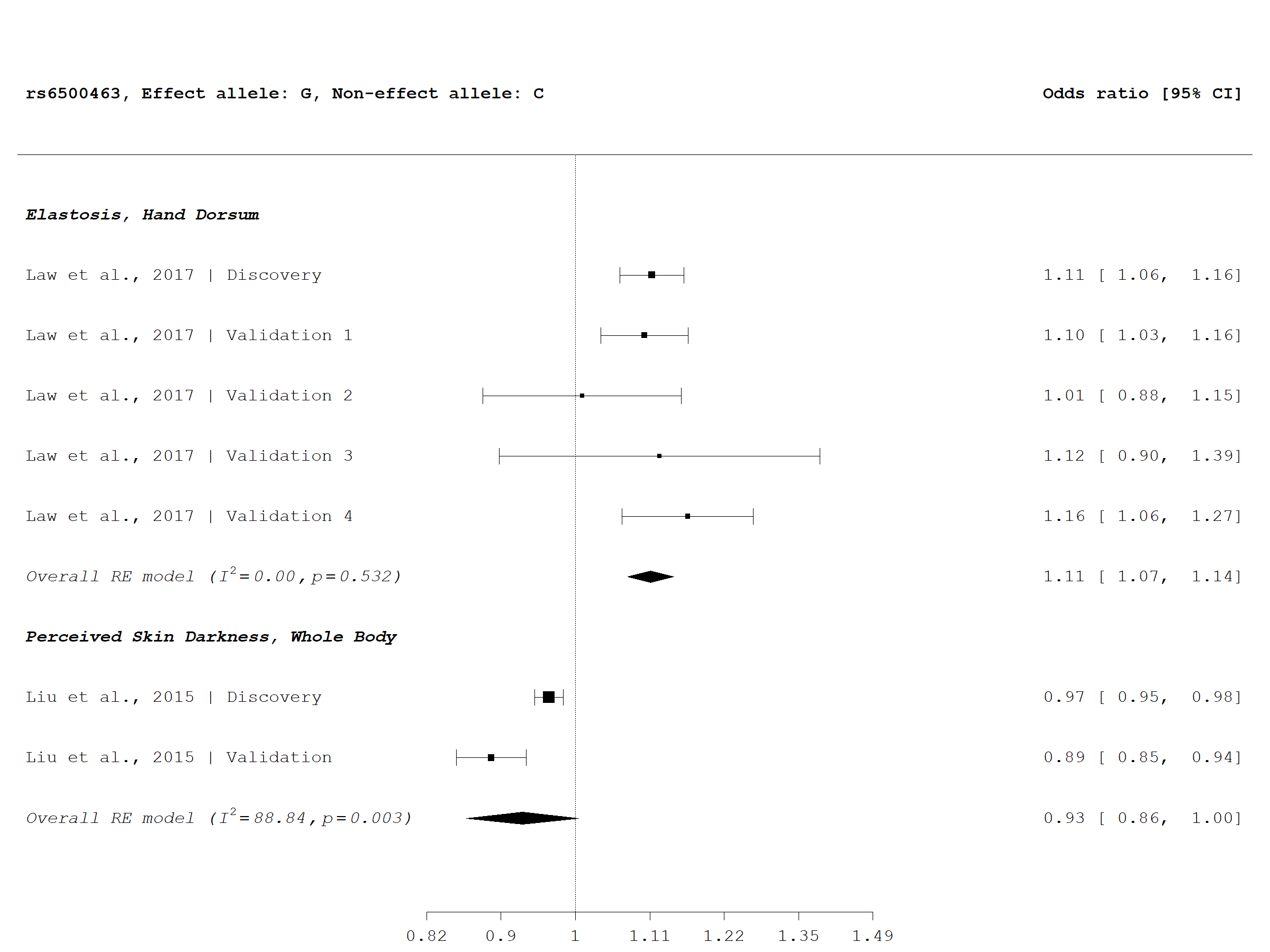

Supplement: Supplementary file 1 — Supplementary Information 1. [file 41598_2022_17443_MOESM1_ESM.zip › Supplementary Datasets/Dataset S3 - Forest Plots/fp117_rs6500463.png]

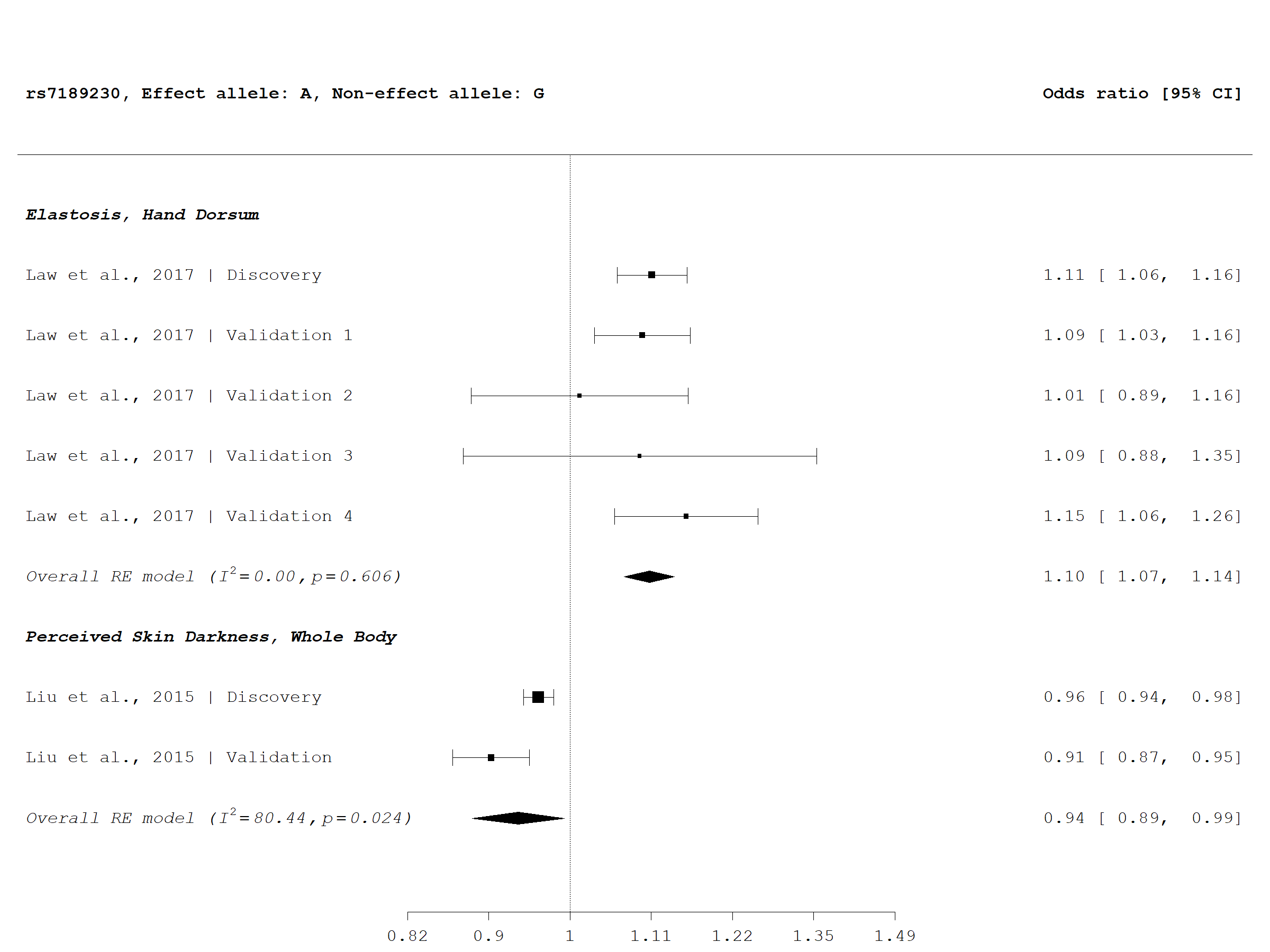

Supplement: Supplementary file 1 — Supplementary Information 1. [file 41598_2022_17443_MOESM1_ESM.zip › Supplementary Datasets/Dataset S3 - Forest Plots/fp118_rs7189230.png]

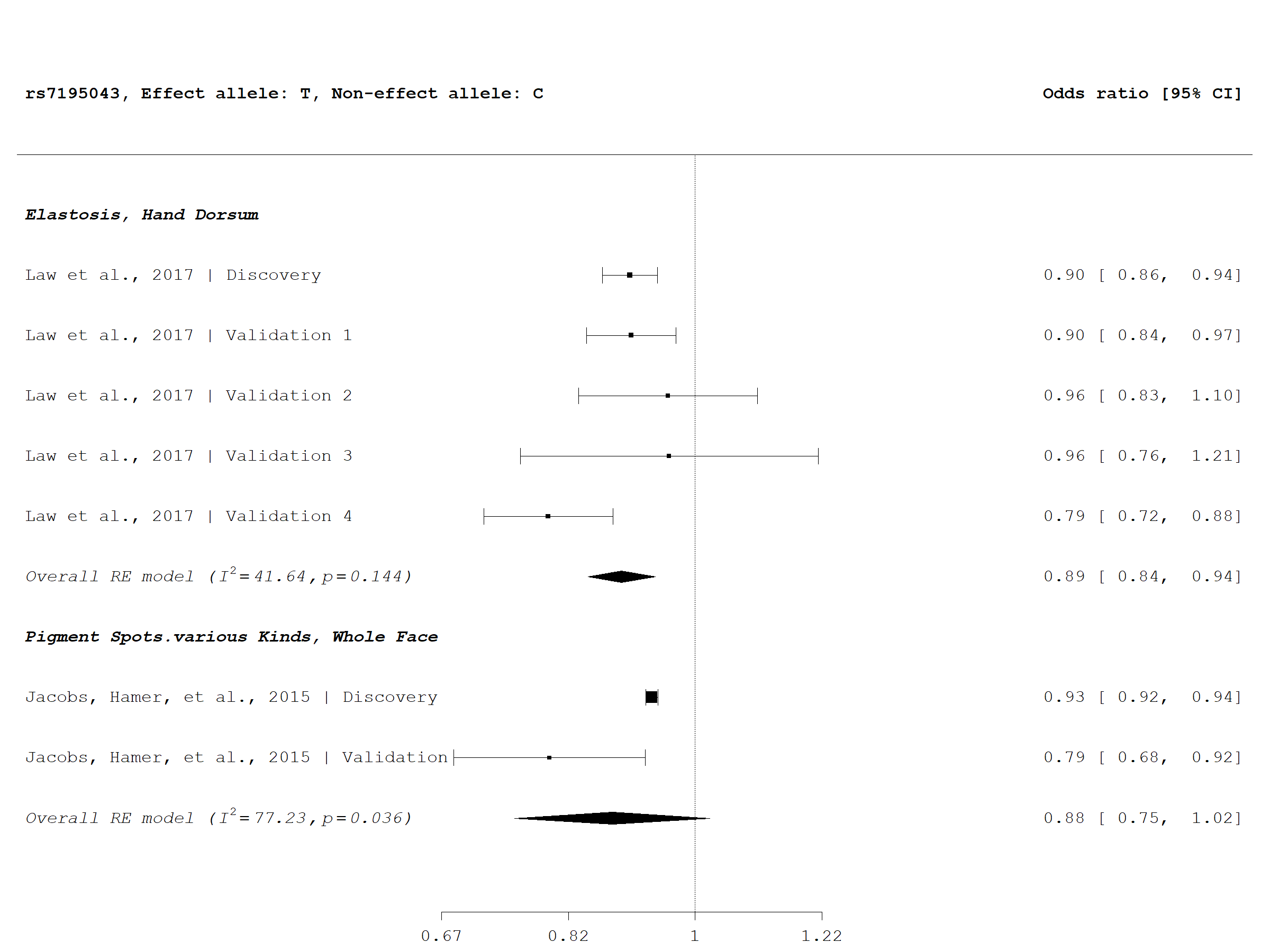

Supplement: Supplementary file 1 — Supplementary Information 1. [file 41598_2022_17443_MOESM1_ESM.zip › Supplementary Datasets/Dataset S3 - Forest Plots/fp119_rs7195043.png]

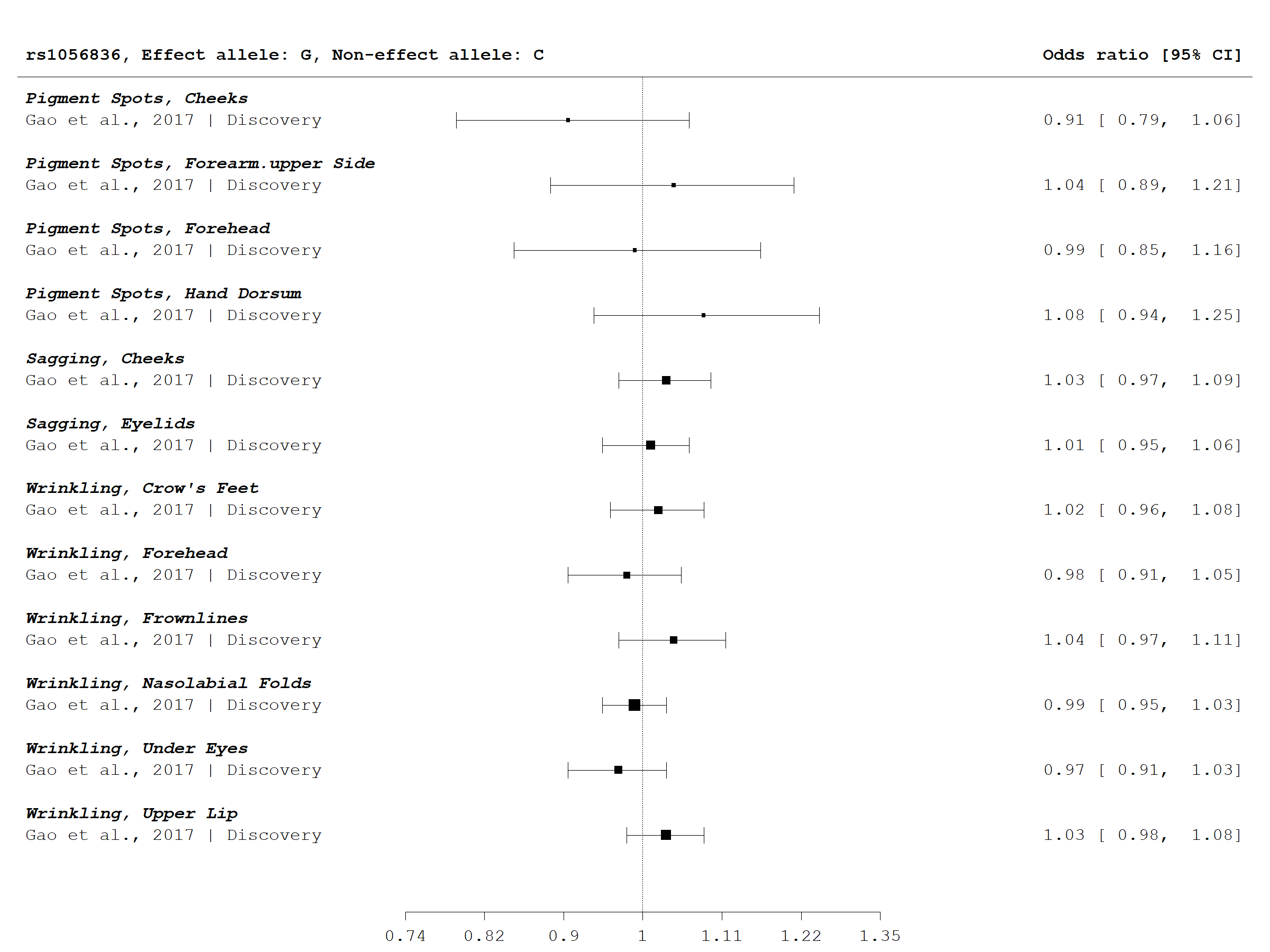

Supplement: Supplementary file 1 — Supplementary Information 1. [file 41598_2022_17443_MOESM1_ESM.zip › Supplementary Datasets/Dataset S3 - Forest Plots/fp11_rs1056836.png]

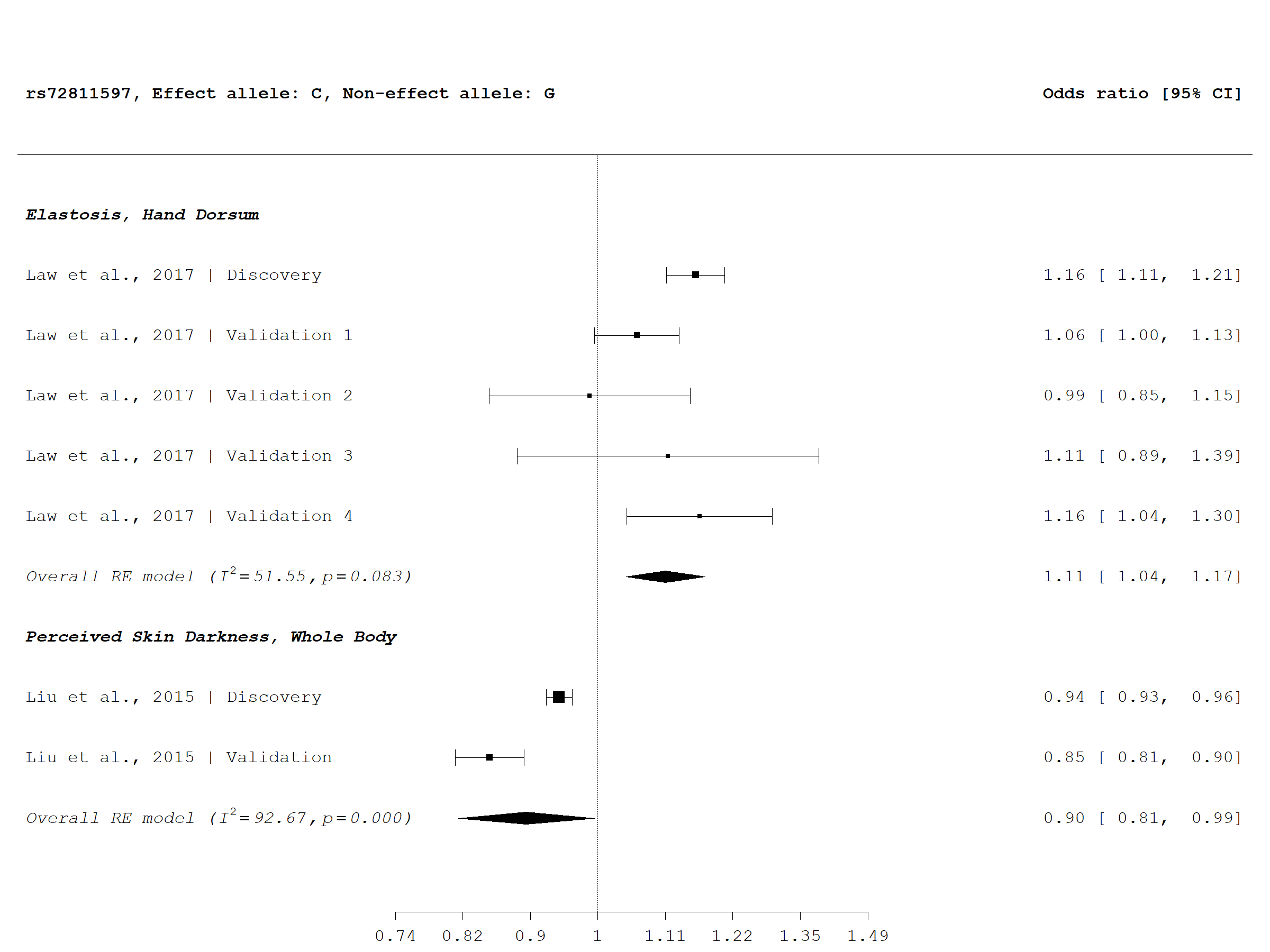

Supplement: Supplementary file 1 — Supplementary Information 1. [file 41598_2022_17443_MOESM1_ESM.zip › Supplementary Datasets/Dataset S3 - Forest Plots/fp120_rs72811597.png]

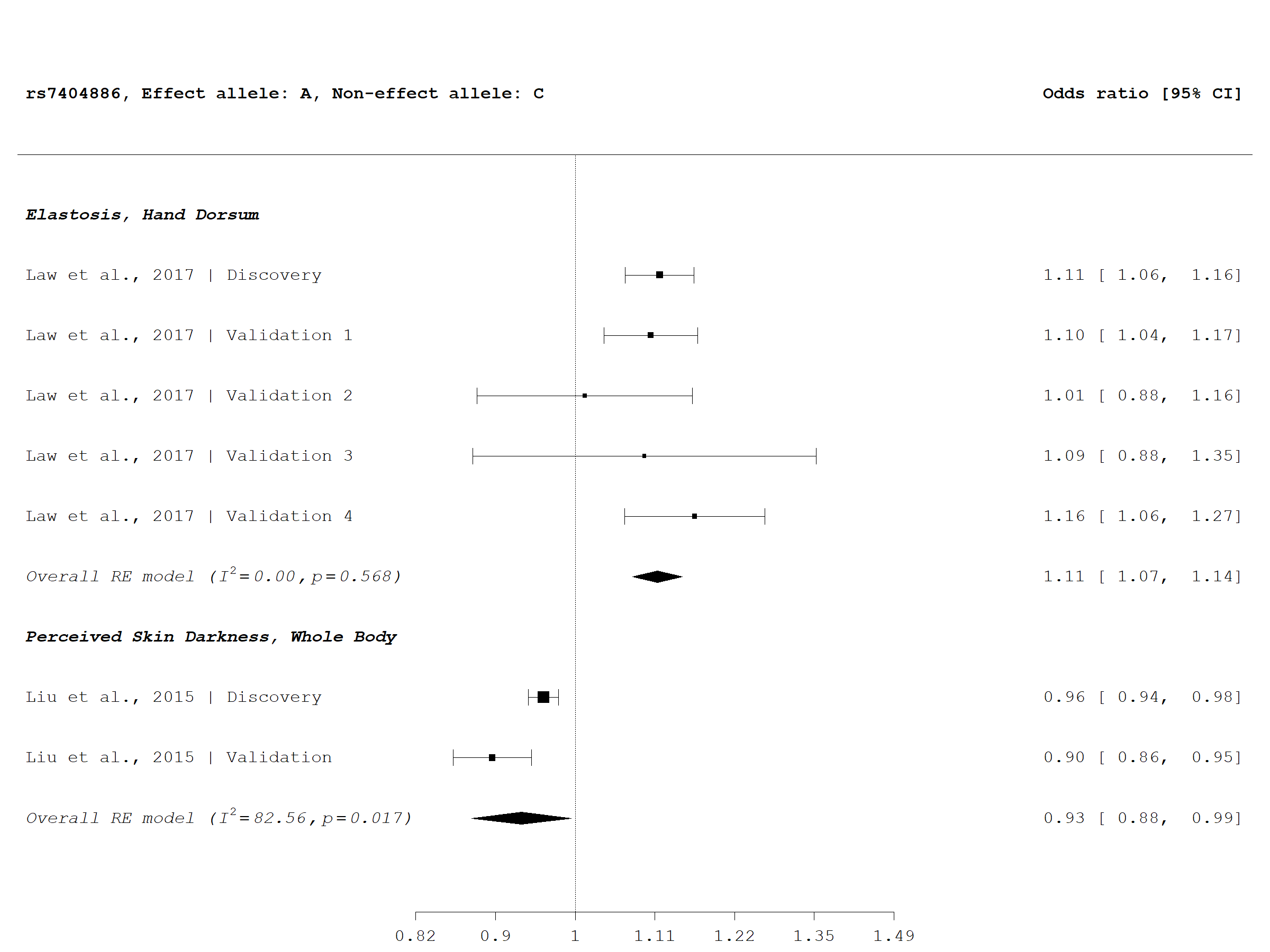

Supplement: Supplementary file 1 — Supplementary Information 1. [file 41598_2022_17443_MOESM1_ESM.zip › Supplementary Datasets/Dataset S3 - Forest Plots/fp121_rs7404886.png]

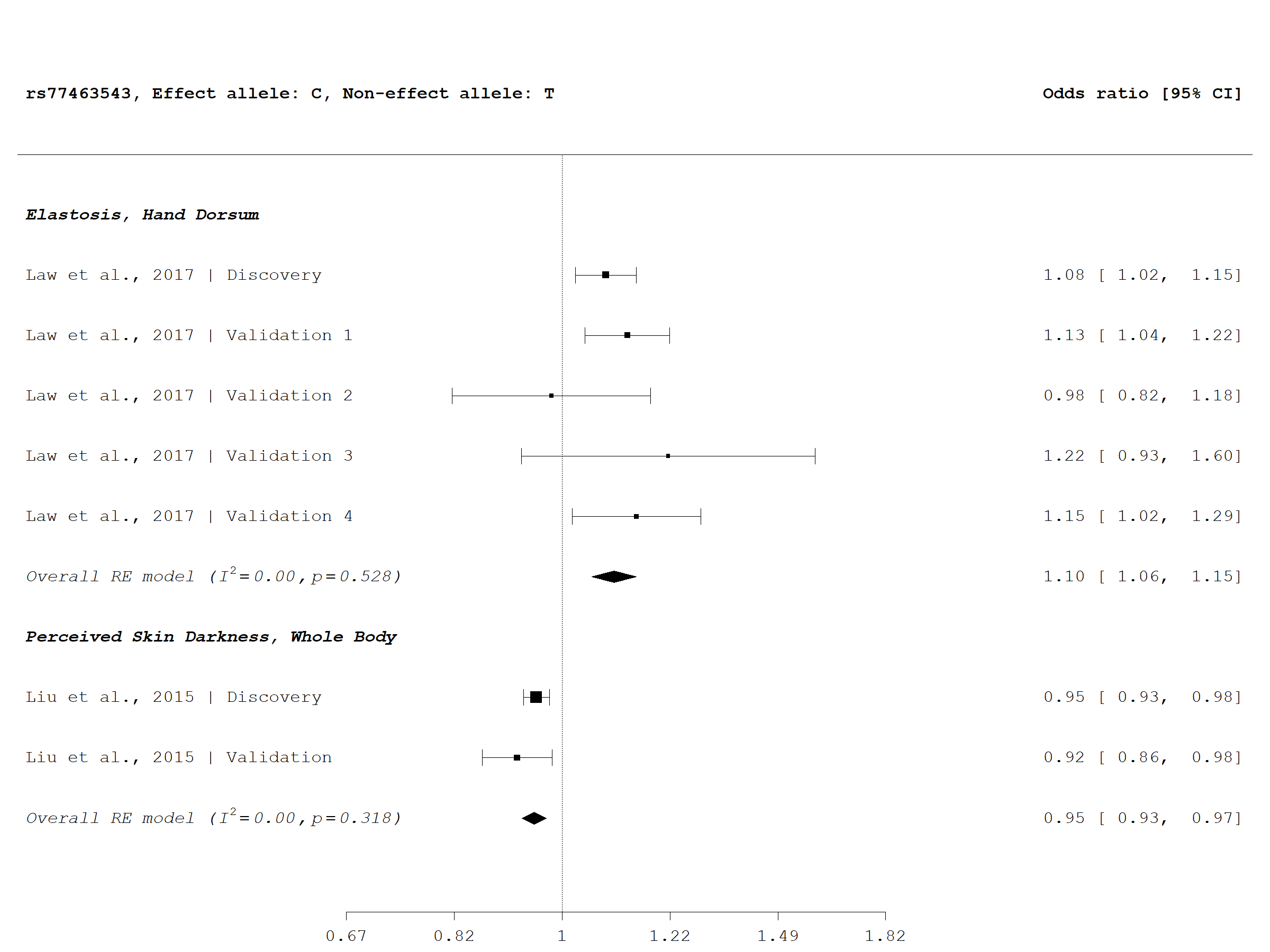

Supplement: Supplementary file 1 — Supplementary Information 1. [file 41598_2022_17443_MOESM1_ESM.zip › Supplementary Datasets/Dataset S3 - Forest Plots/fp122_rs77463543.png]

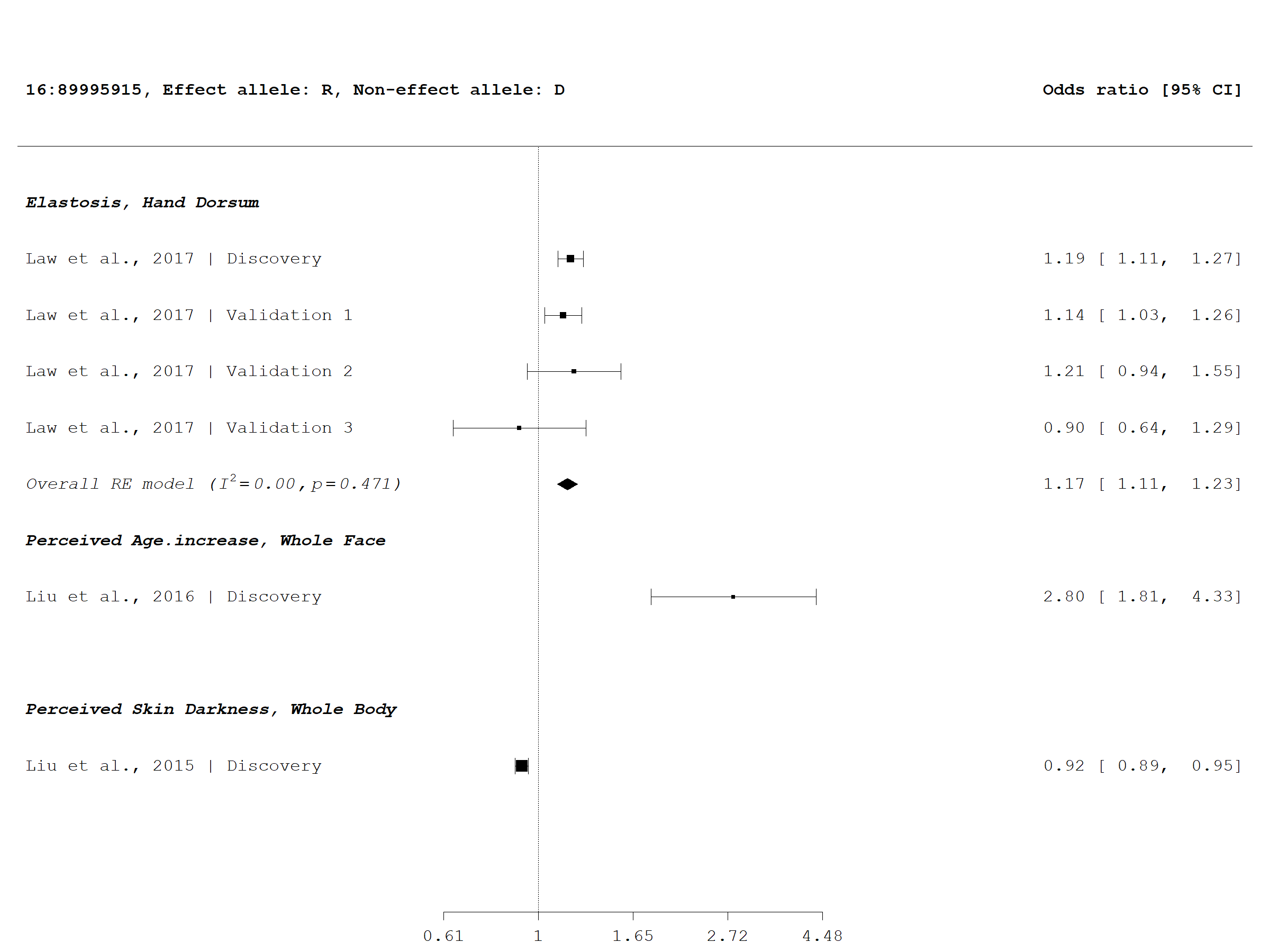

Supplement: Supplementary file 1 — Supplementary Information 1. [file 41598_2022_17443_MOESM1_ESM.zip › Supplementary Datasets/Dataset S3 - Forest Plots/fp123_16_89995915.png]

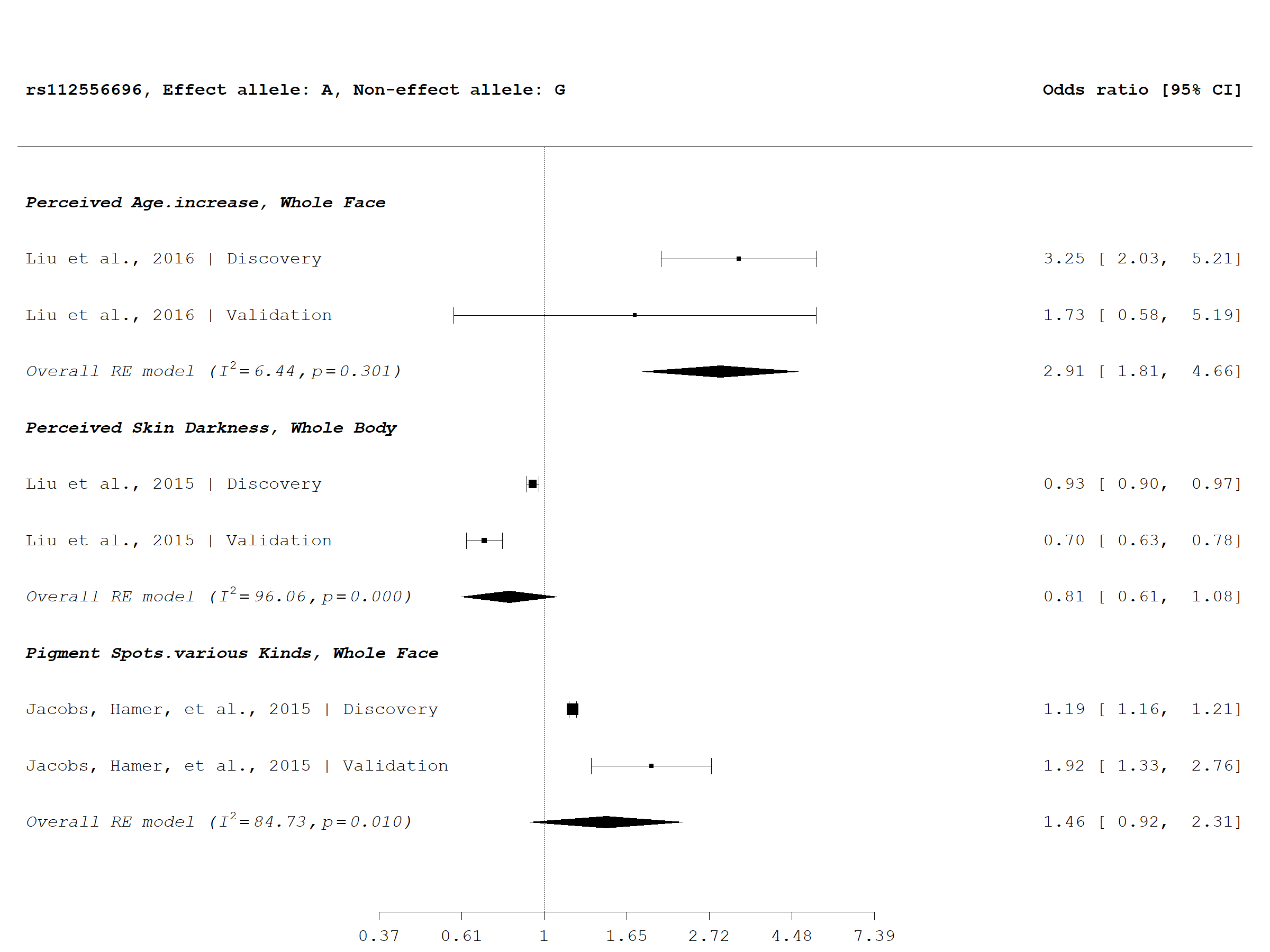

Supplement: Supplementary file 1 — Supplementary Information 1. [file 41598_2022_17443_MOESM1_ESM.zip › Supplementary Datasets/Dataset S3 - Forest Plots/fp124_rs112556696.png]

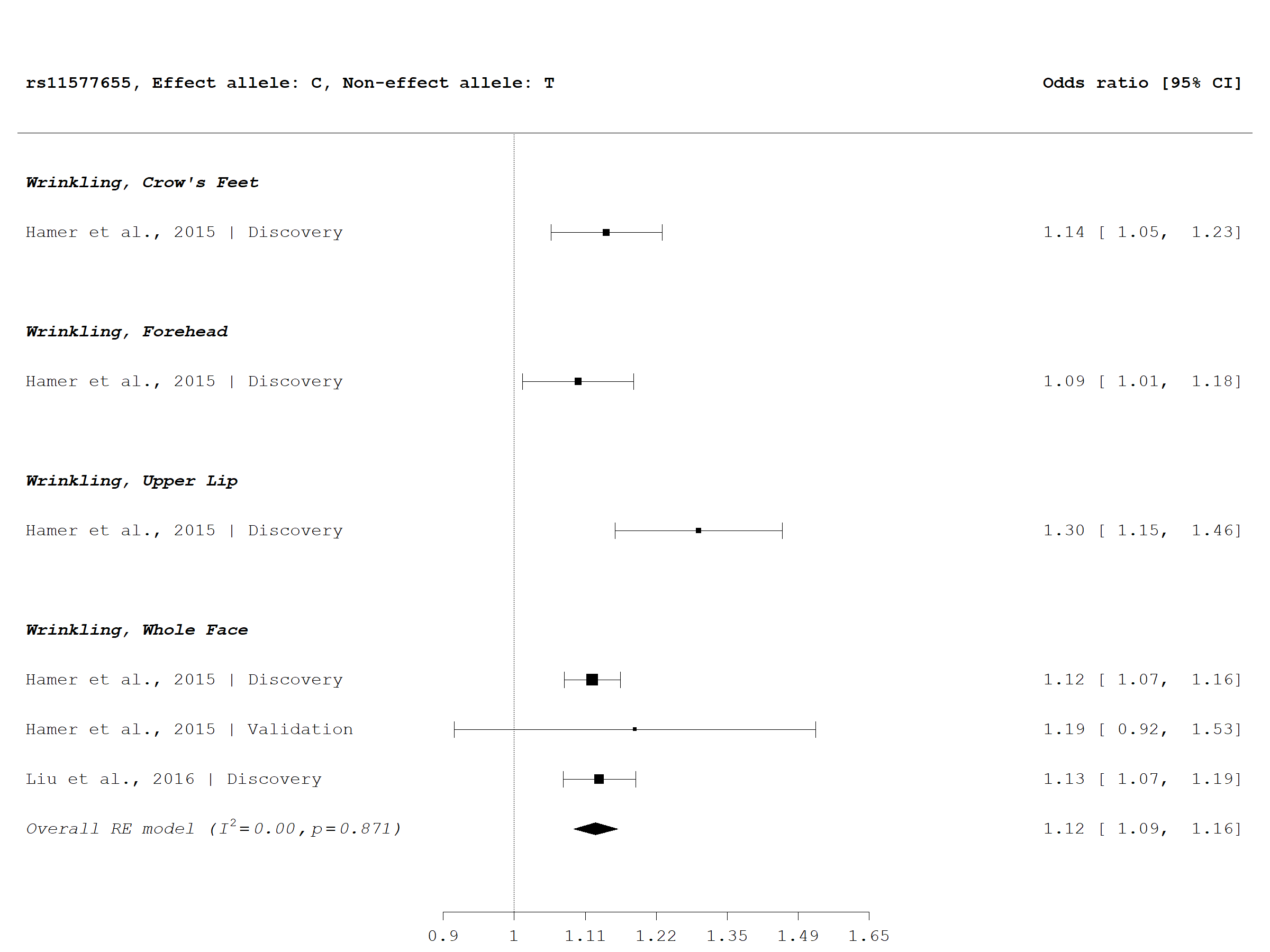

Supplement: Supplementary file 1 — Supplementary Information 1. [file 41598_2022_17443_MOESM1_ESM.zip › Supplementary Datasets/Dataset S3 - Forest Plots/fp125_rs11577655.png]

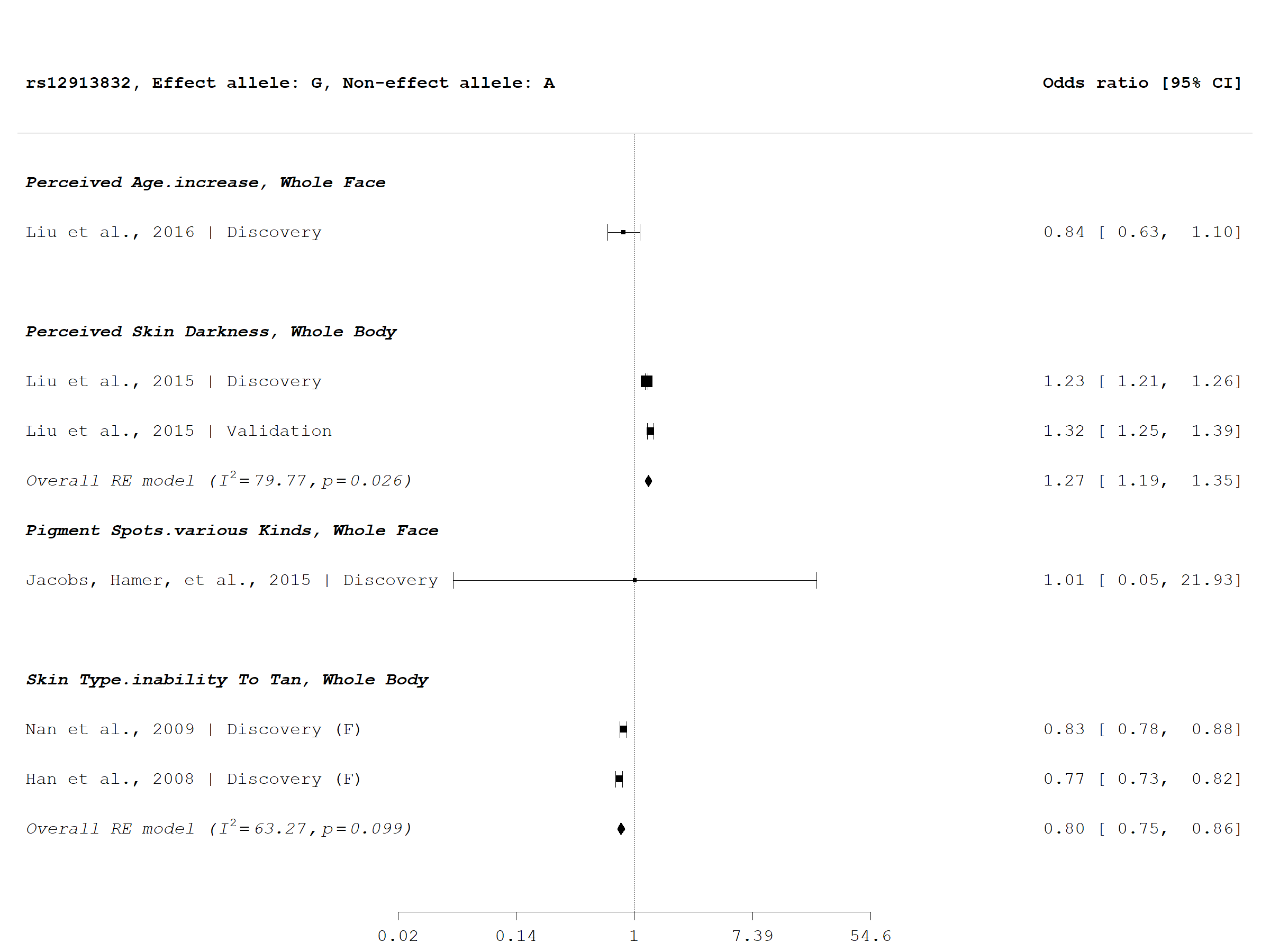

Supplement: Supplementary file 1 — Supplementary Information 1. [file 41598_2022_17443_MOESM1_ESM.zip › Supplementary Datasets/Dataset S3 - Forest Plots/fp126_rs12913832.png]

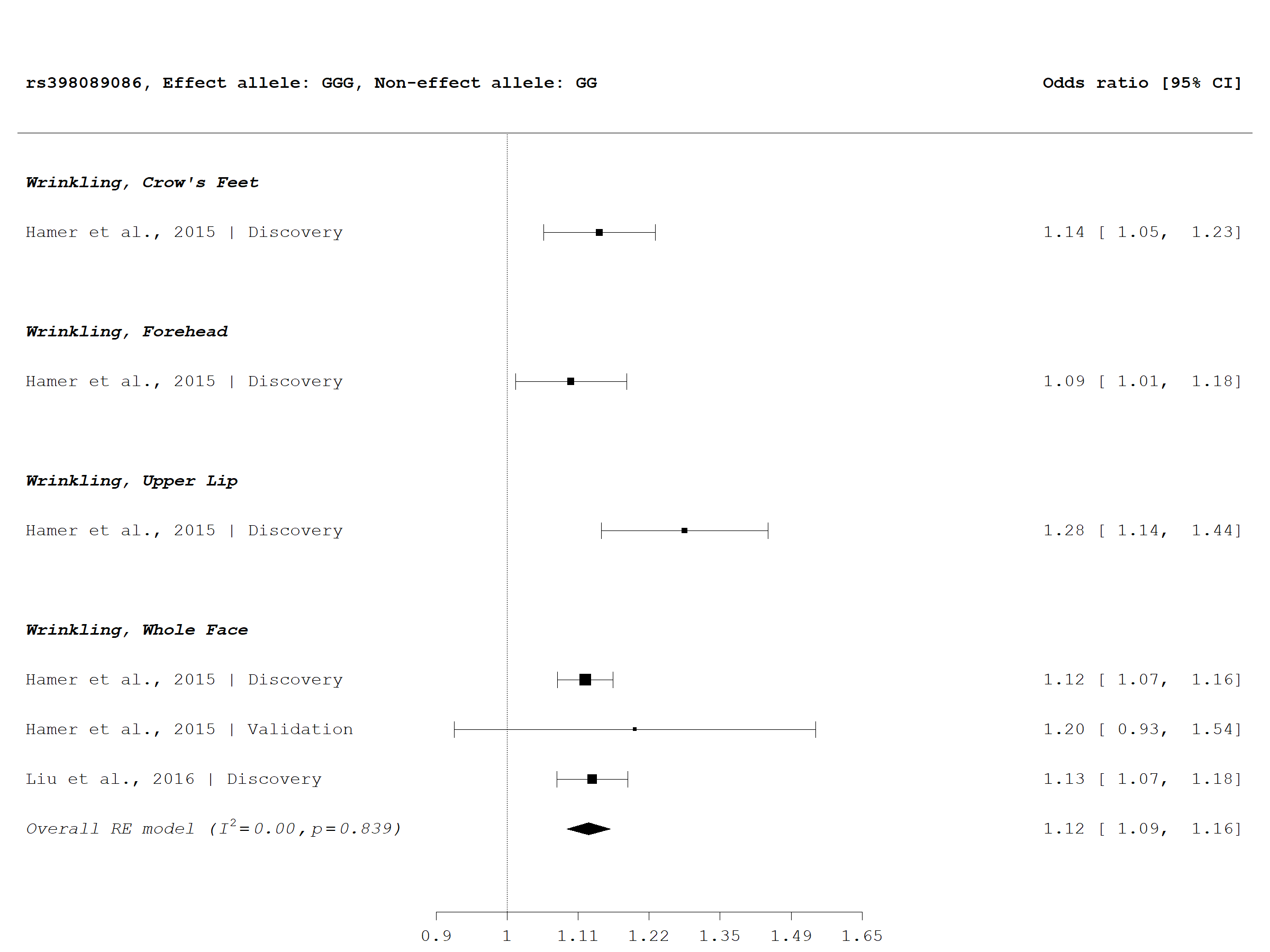

Supplement: Supplementary file 1 — Supplementary Information 1. [file 41598_2022_17443_MOESM1_ESM.zip › Supplementary Datasets/Dataset S3 - Forest Plots/fp127_rs398089086.png]

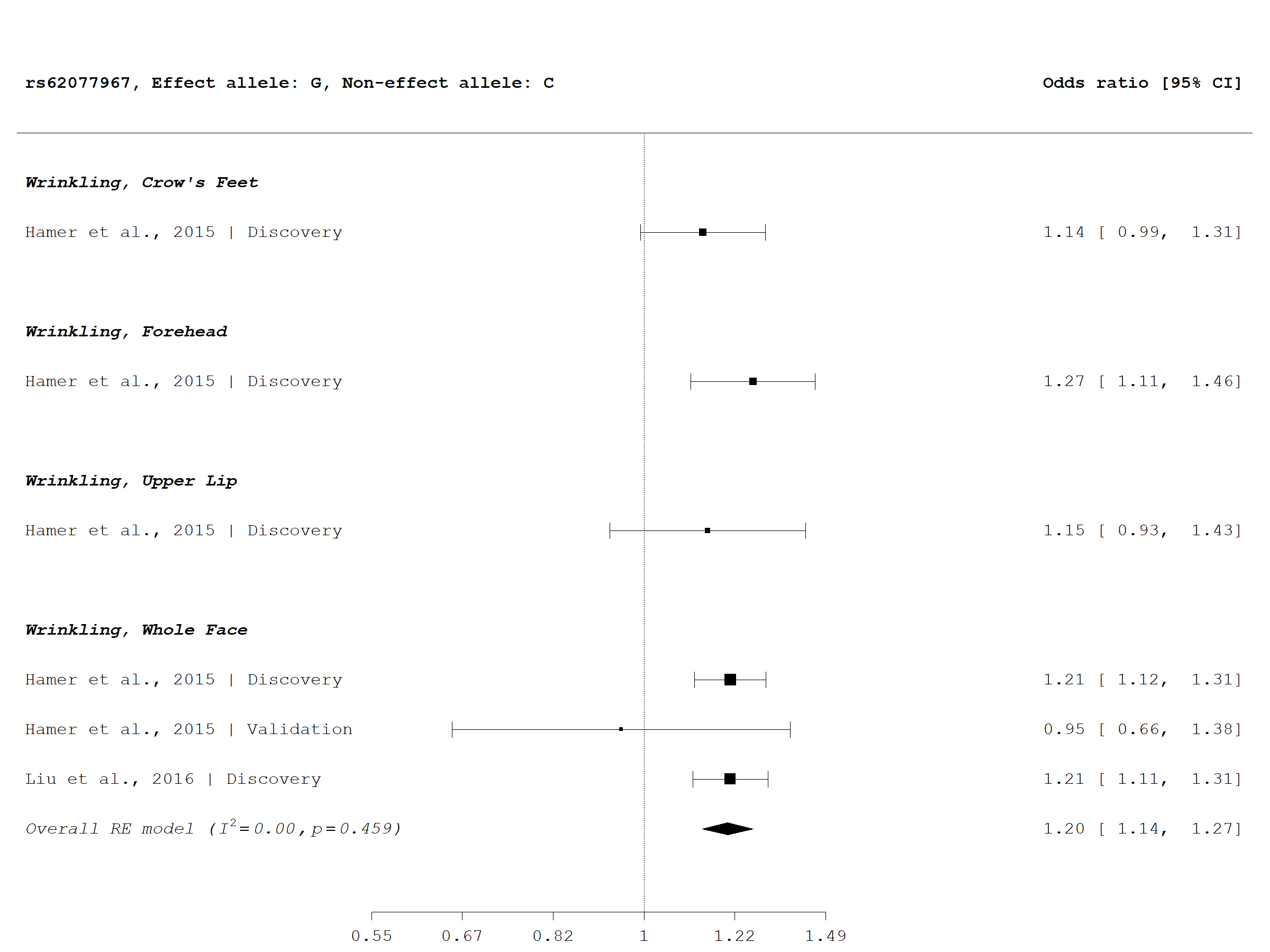

Supplement: Supplementary file 1 — Supplementary Information 1. [file 41598_2022_17443_MOESM1_ESM.zip › Supplementary Datasets/Dataset S3 - Forest Plots/fp128_rs62077967.png]

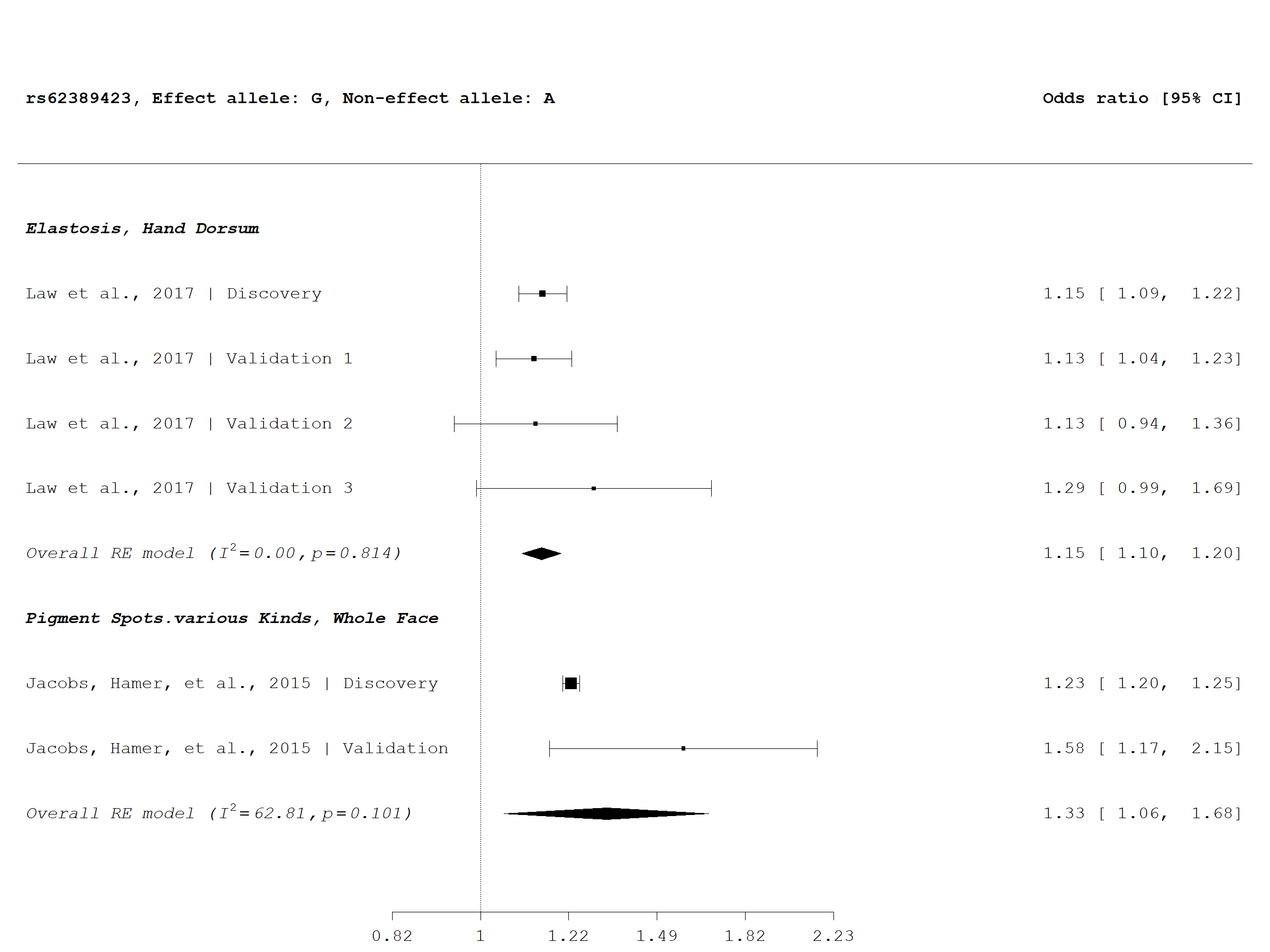

Supplement: Supplementary file 1 — Supplementary Information 1. [file 41598_2022_17443_MOESM1_ESM.zip › Supplementary Datasets/Dataset S3 - Forest Plots/fp129_rs62389423.png]

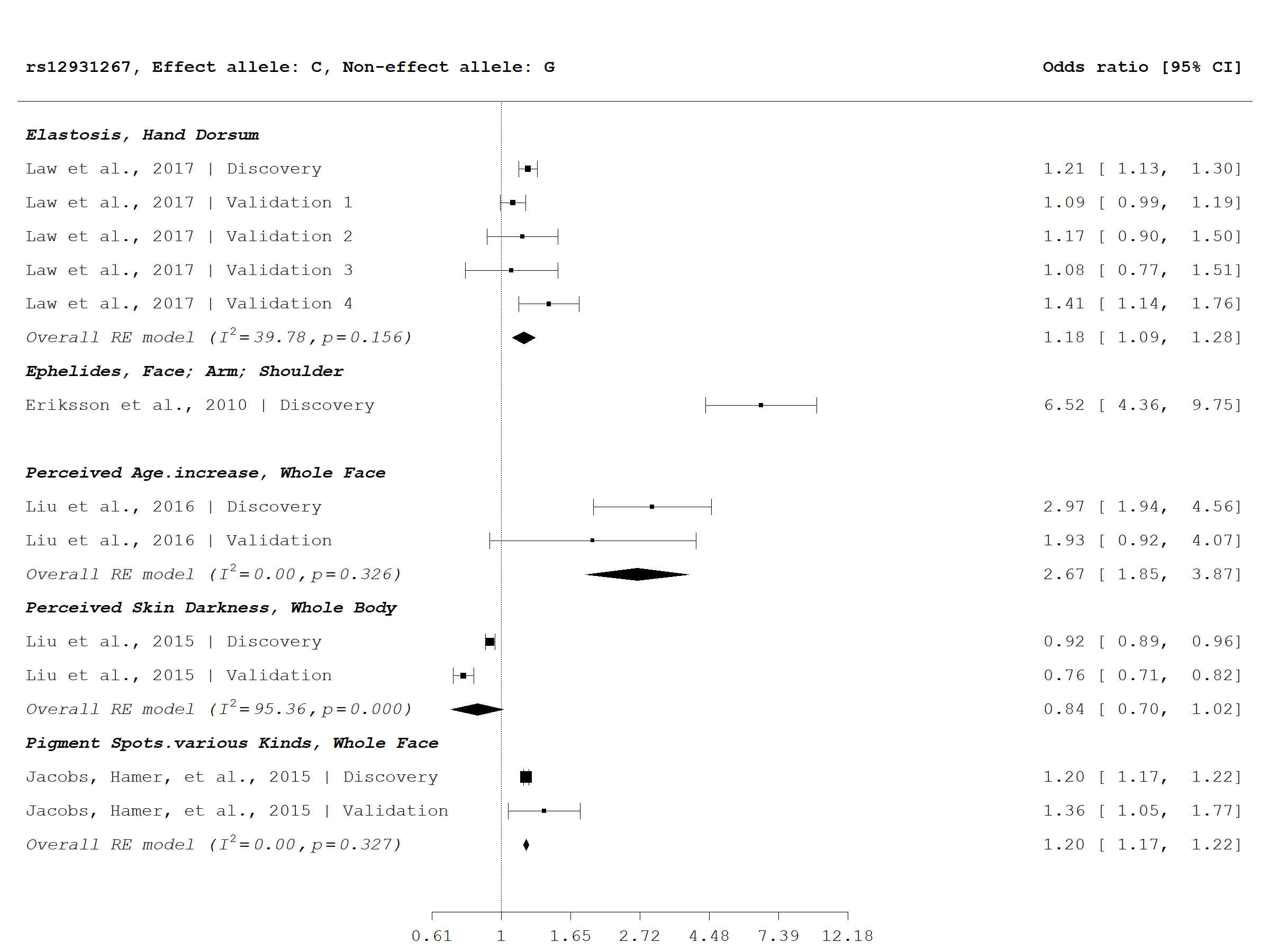

Supplement: Supplementary file 1 — Supplementary Information 1. [file 41598_2022_17443_MOESM1_ESM.zip › Supplementary Datasets/Dataset S3 - Forest Plots/fp12_rs12931267.png]

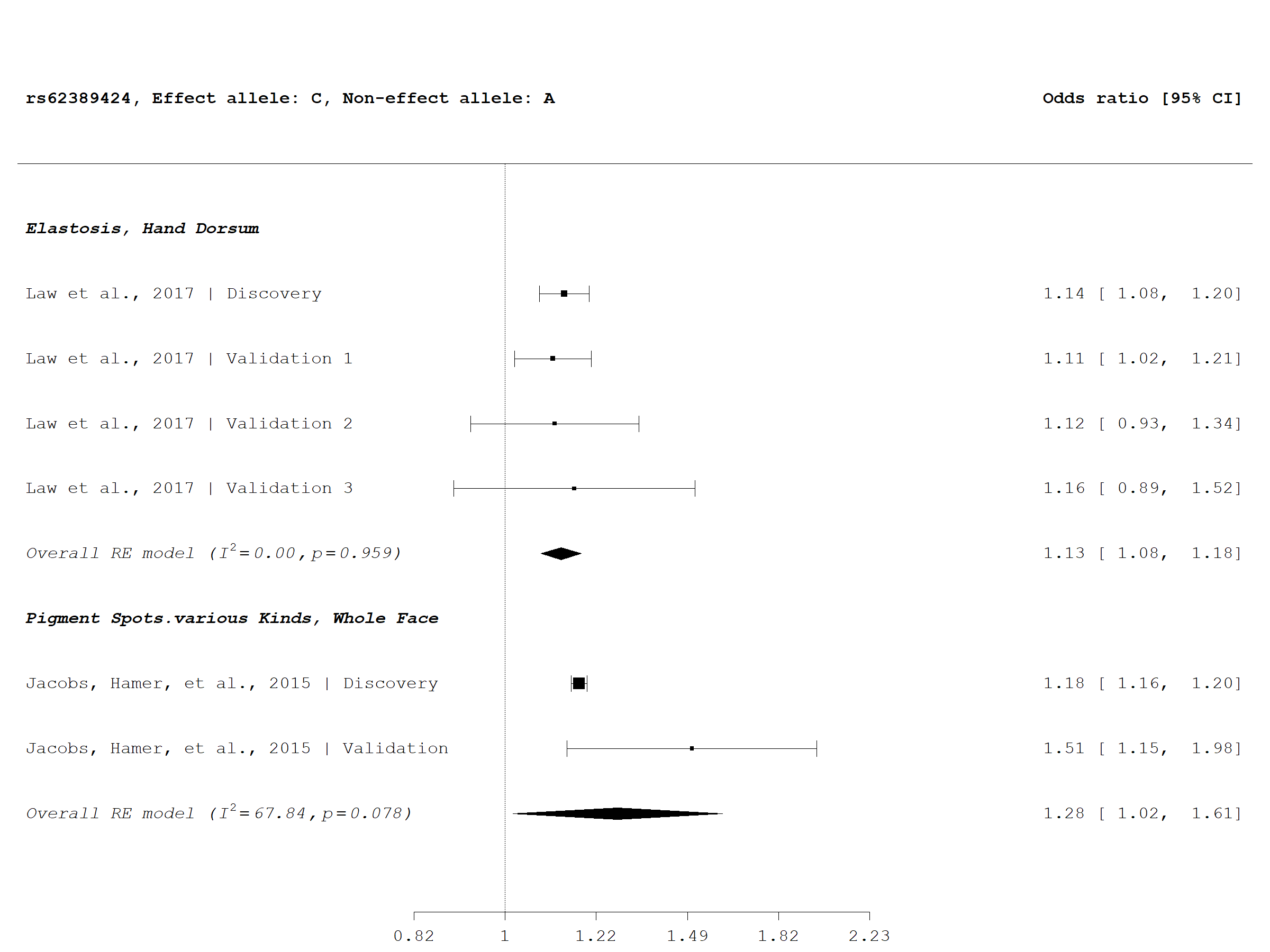

Supplement: Supplementary file 1 — Supplementary Information 1. [file 41598_2022_17443_MOESM1_ESM.zip › Supplementary Datasets/Dataset S3 - Forest Plots/fp130_rs62389424.png]

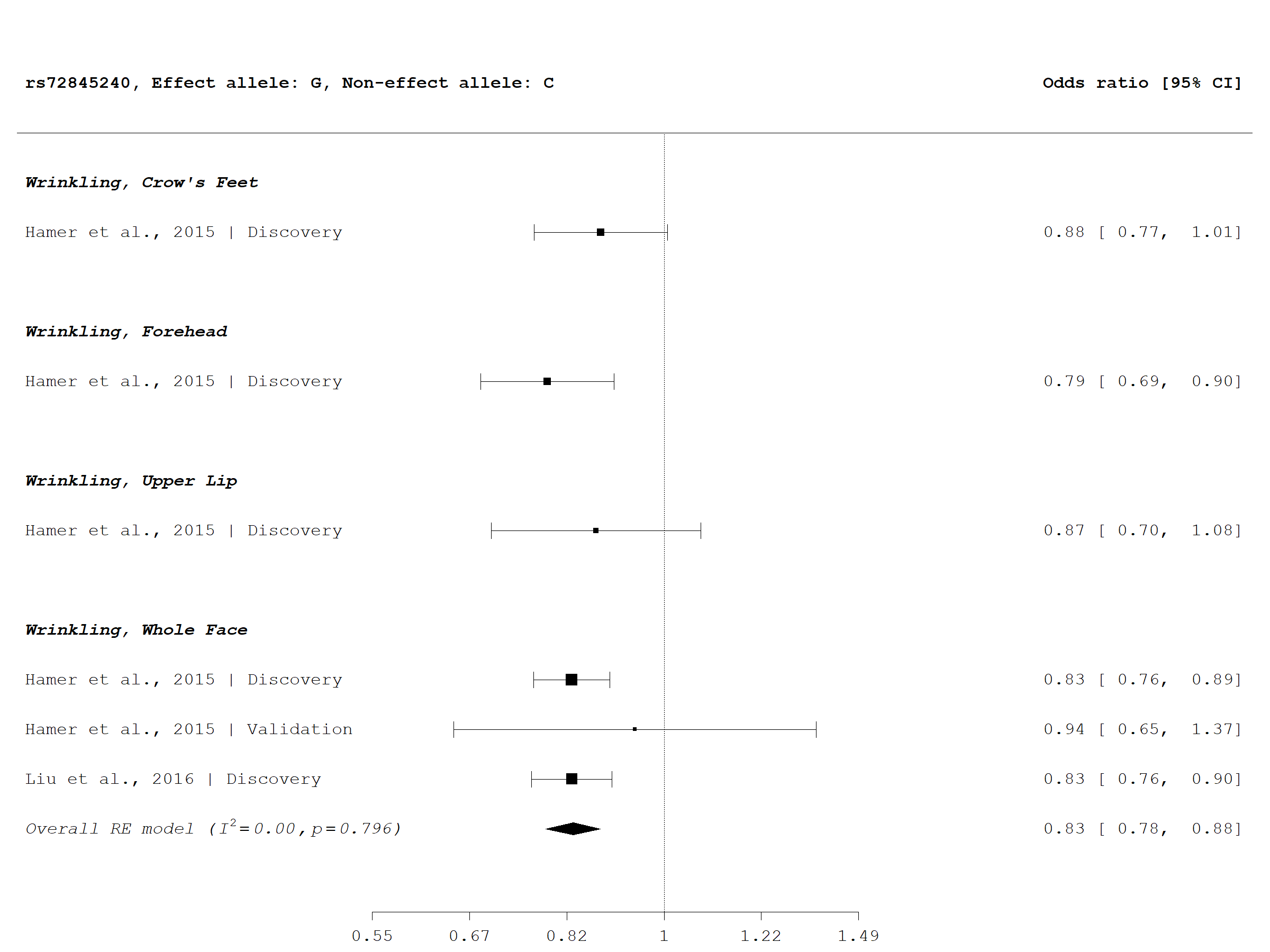

Supplement: Supplementary file 1 — Supplementary Information 1. [file 41598_2022_17443_MOESM1_ESM.zip › Supplementary Datasets/Dataset S3 - Forest Plots/fp131_rs72845240.png]

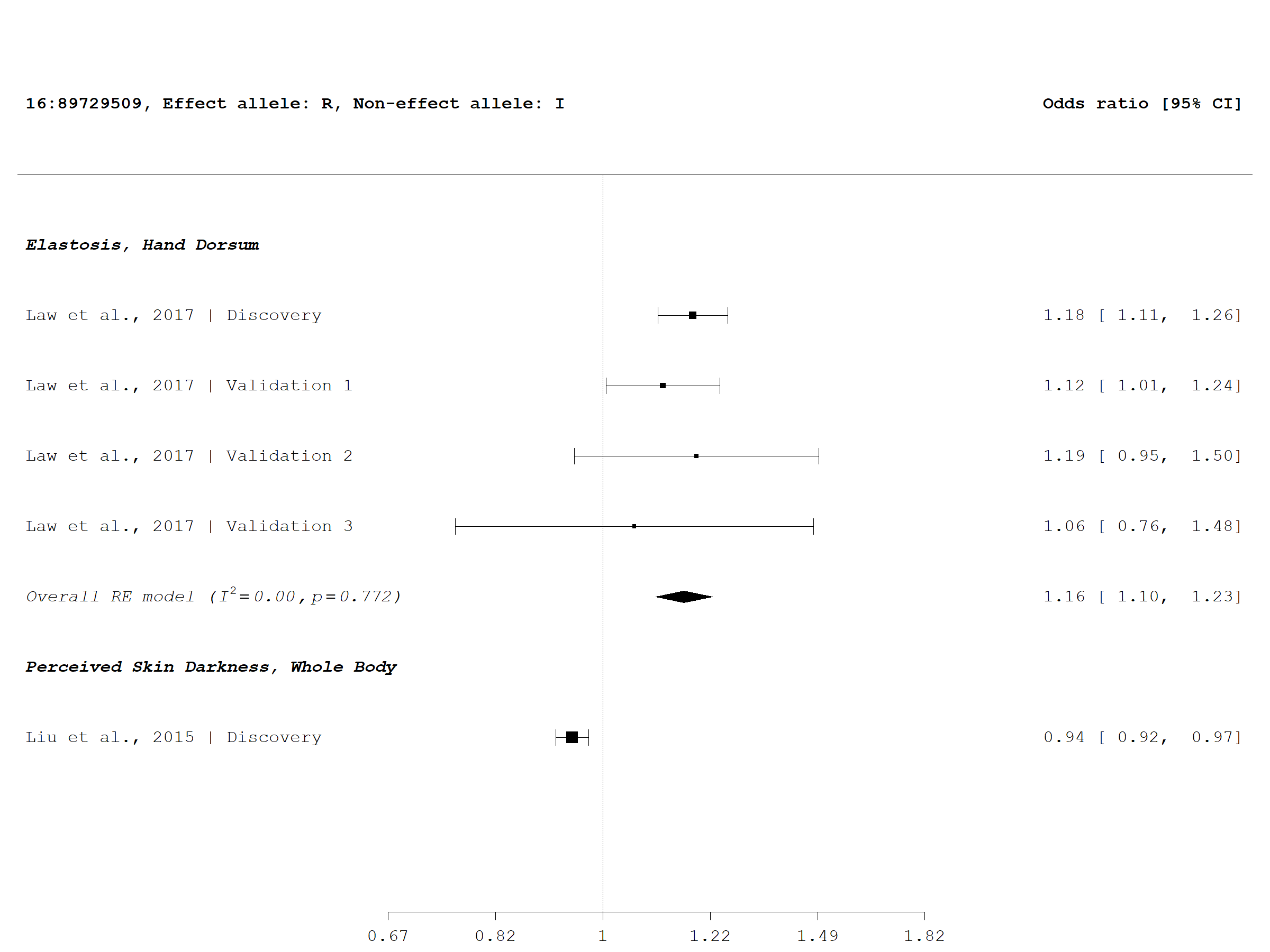

Supplement: Supplementary file 1 — Supplementary Information 1. [file 41598_2022_17443_MOESM1_ESM.zip › Supplementary Datasets/Dataset S3 - Forest Plots/fp132_16_89729509.png]

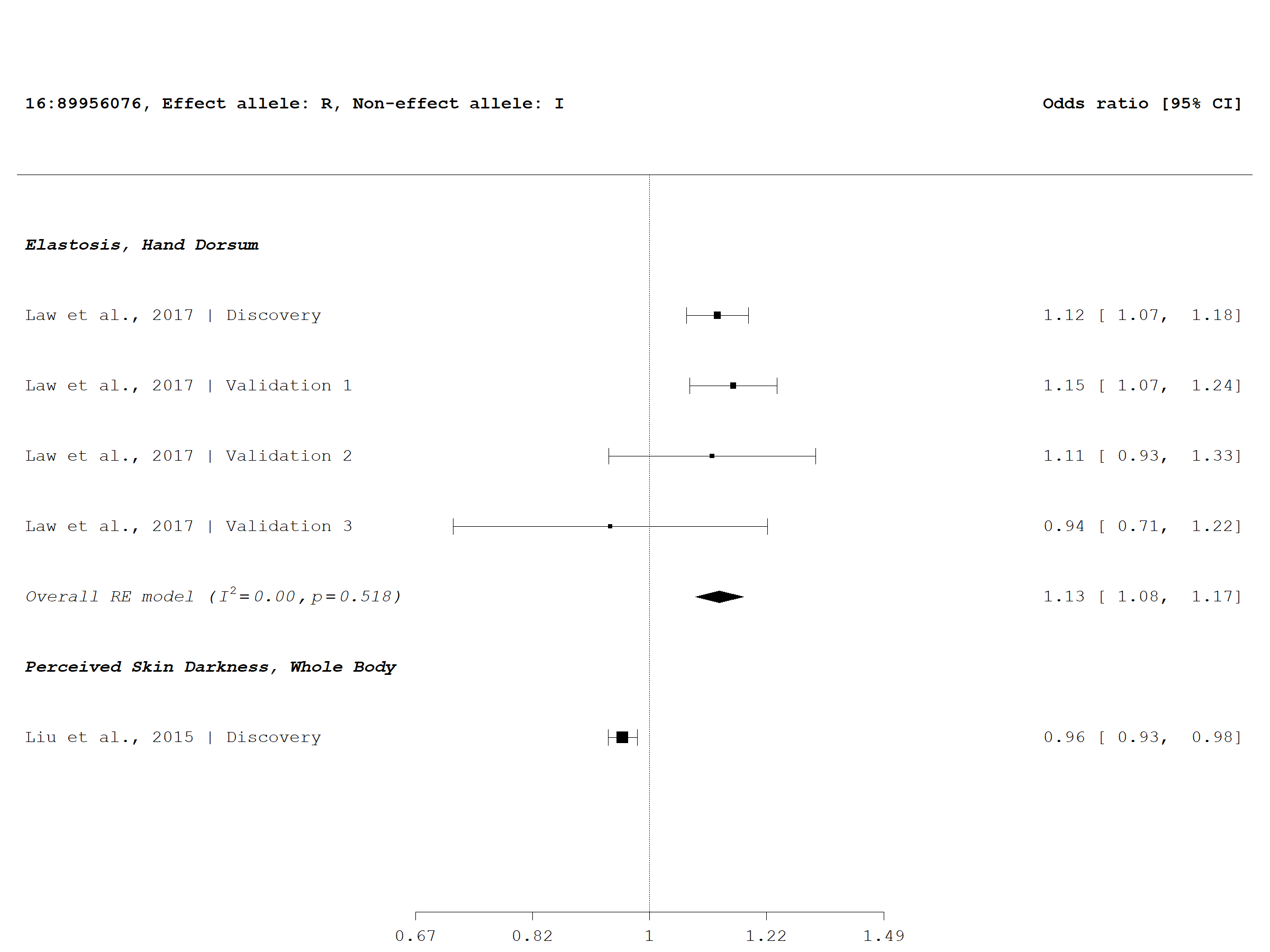

Supplement: Supplementary file 1 — Supplementary Information 1. [file 41598_2022_17443_MOESM1_ESM.zip › Supplementary Datasets/Dataset S3 - Forest Plots/fp133_16_89956076.png]

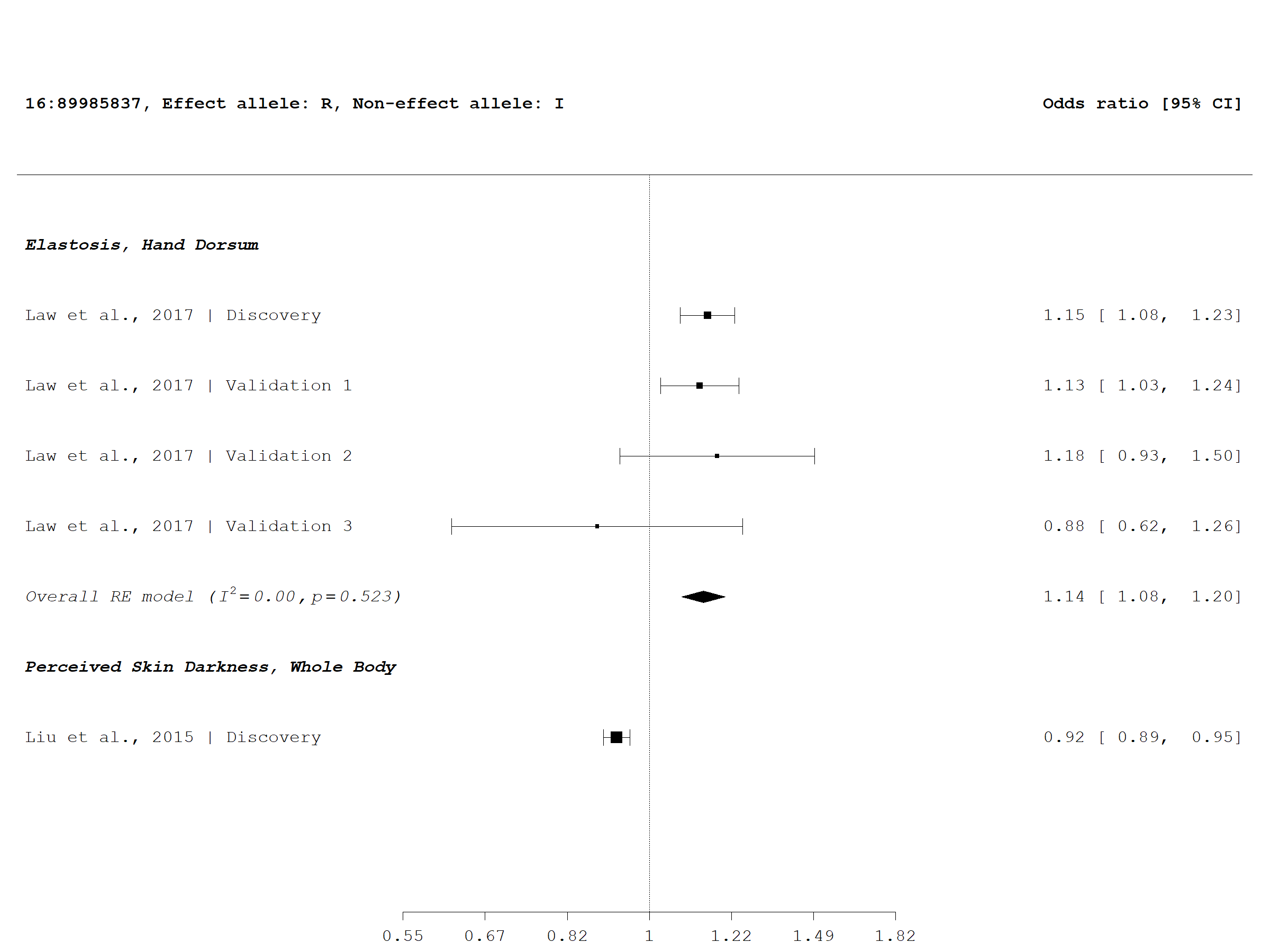

Supplement: Supplementary file 1 — Supplementary Information 1. [file 41598_2022_17443_MOESM1_ESM.zip › Supplementary Datasets/Dataset S3 - Forest Plots/fp134_16_89985837.png]

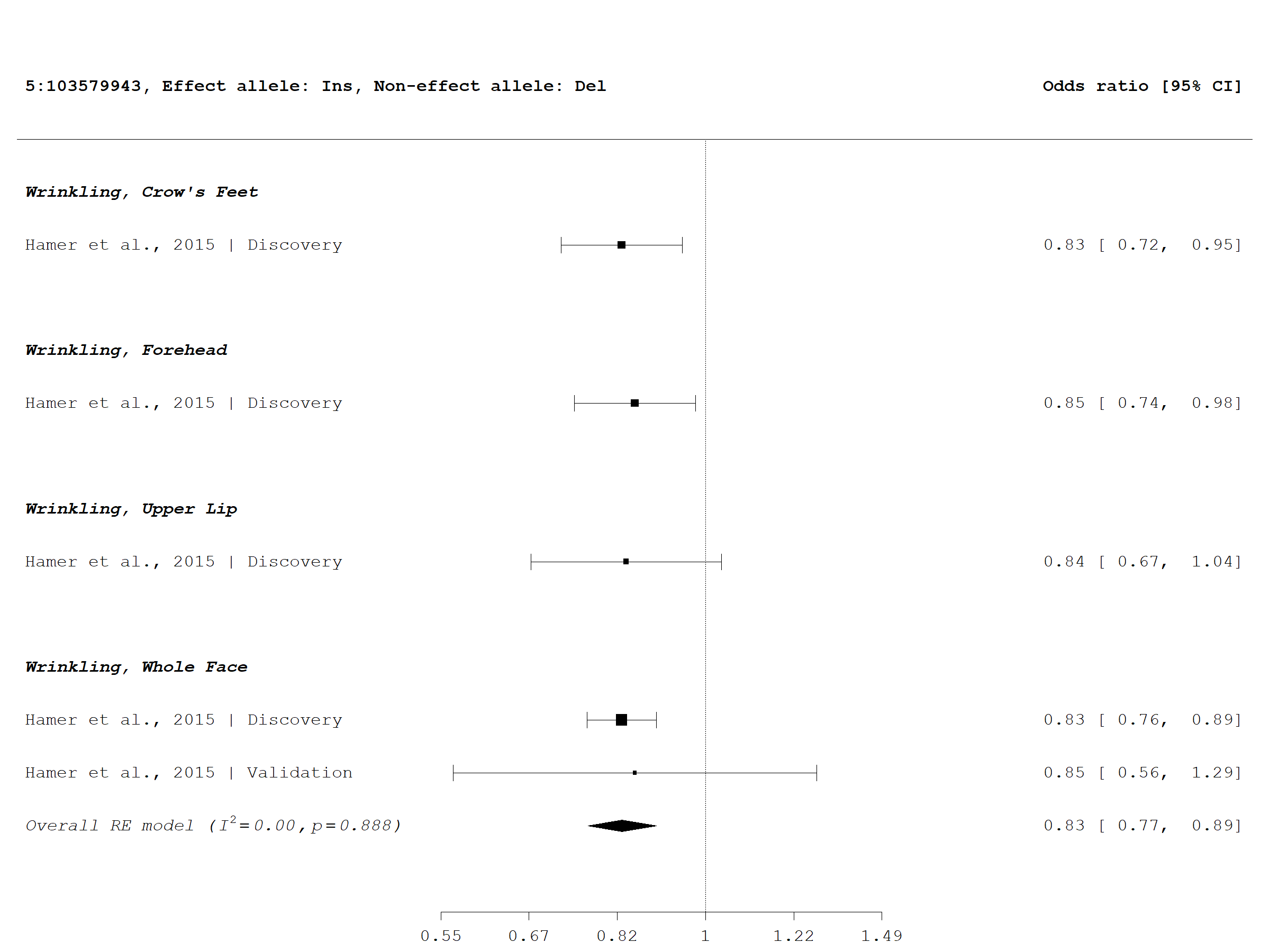

Supplement: Supplementary file 1 — Supplementary Information 1. [file 41598_2022_17443_MOESM1_ESM.zip › Supplementary Datasets/Dataset S3 - Forest Plots/fp135_5_103579943.png]

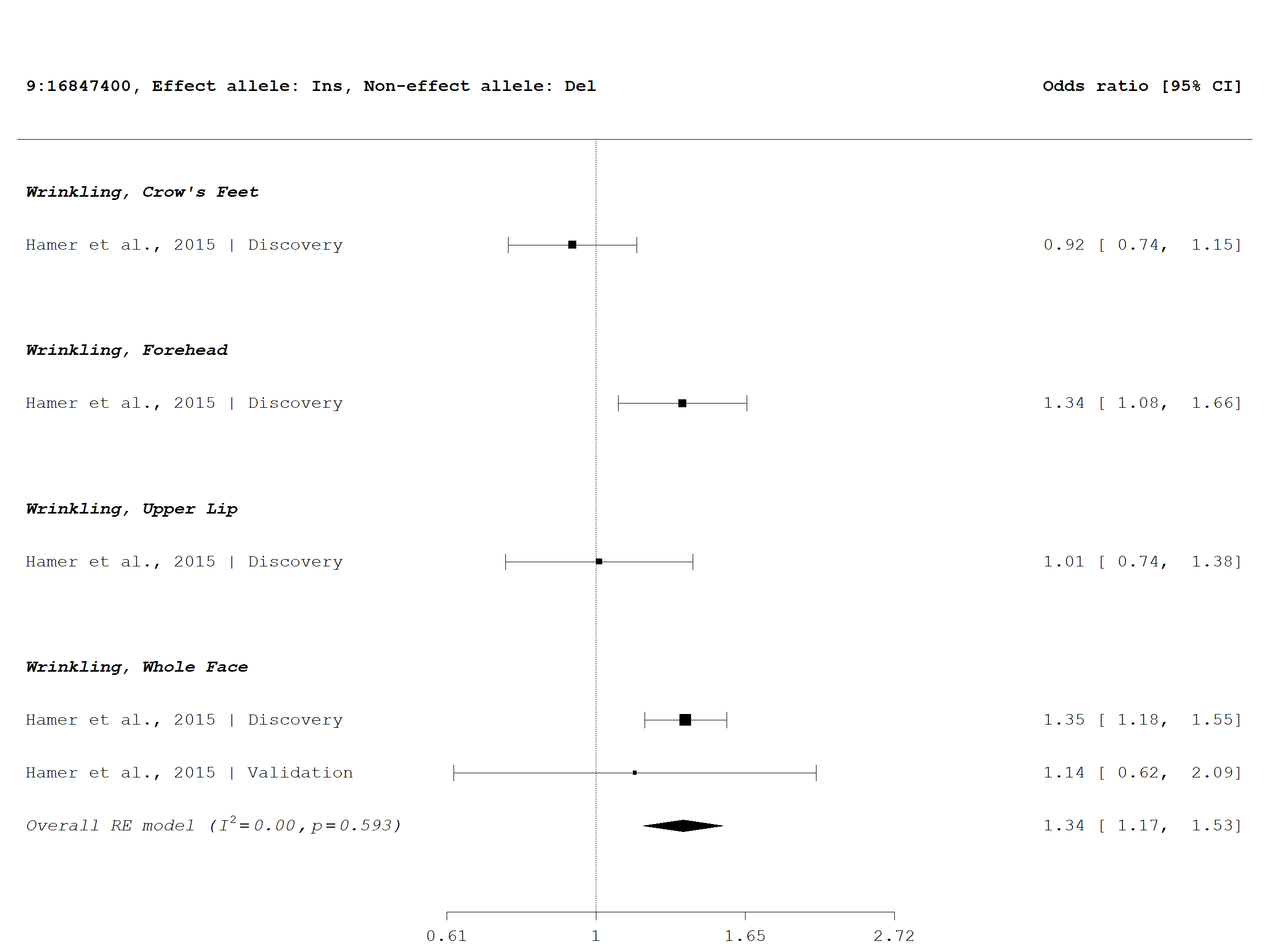

Supplement: Supplementary file 1 — Supplementary Information 1. [file 41598_2022_17443_MOESM1_ESM.zip › Supplementary Datasets/Dataset S3 - Forest Plots/fp136_9_16847400.png]

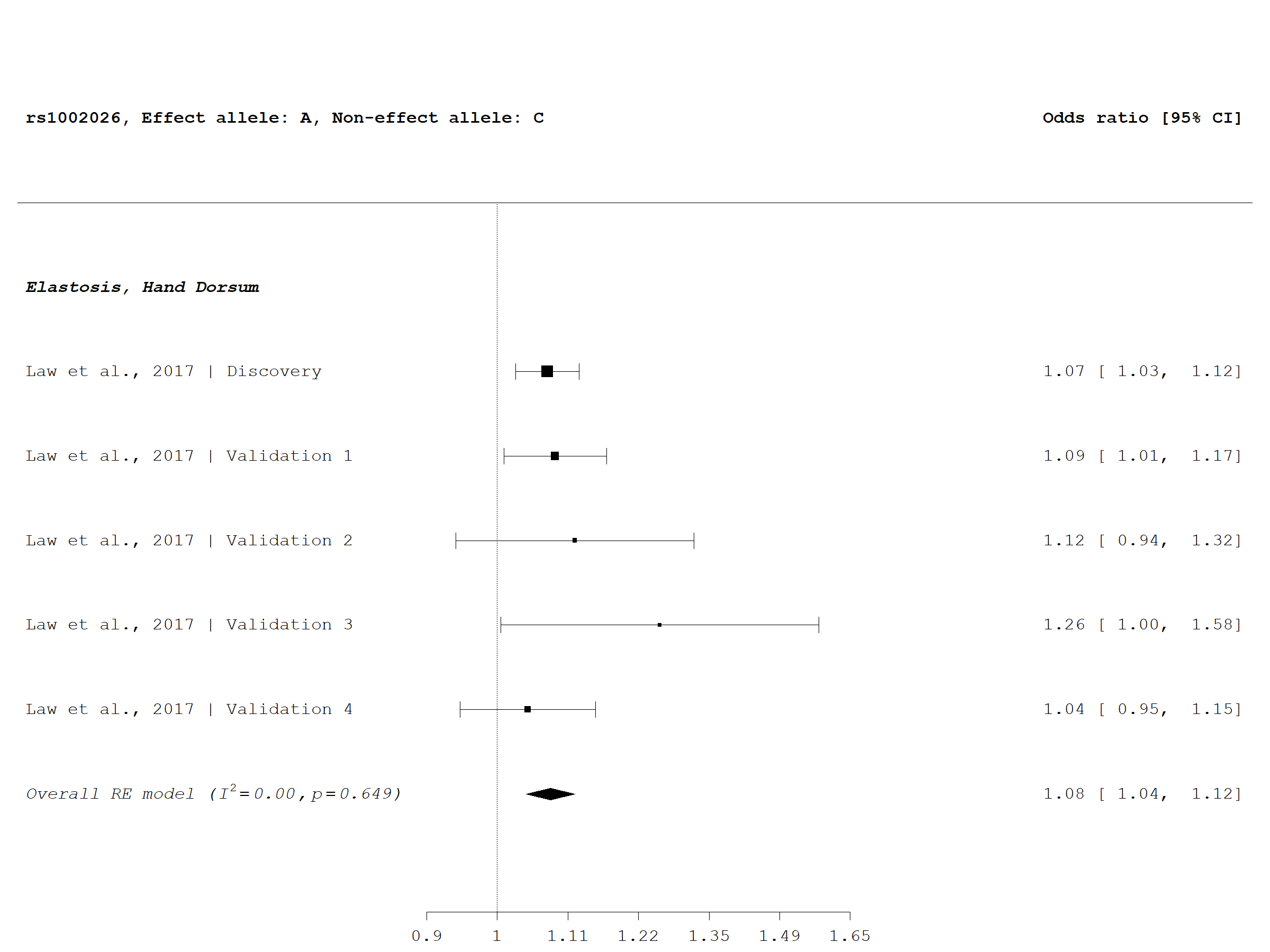

Supplement: Supplementary file 1 — Supplementary Information 1. [file 41598_2022_17443_MOESM1_ESM.zip › Supplementary Datasets/Dataset S3 - Forest Plots/fp137_rs1002026.png]

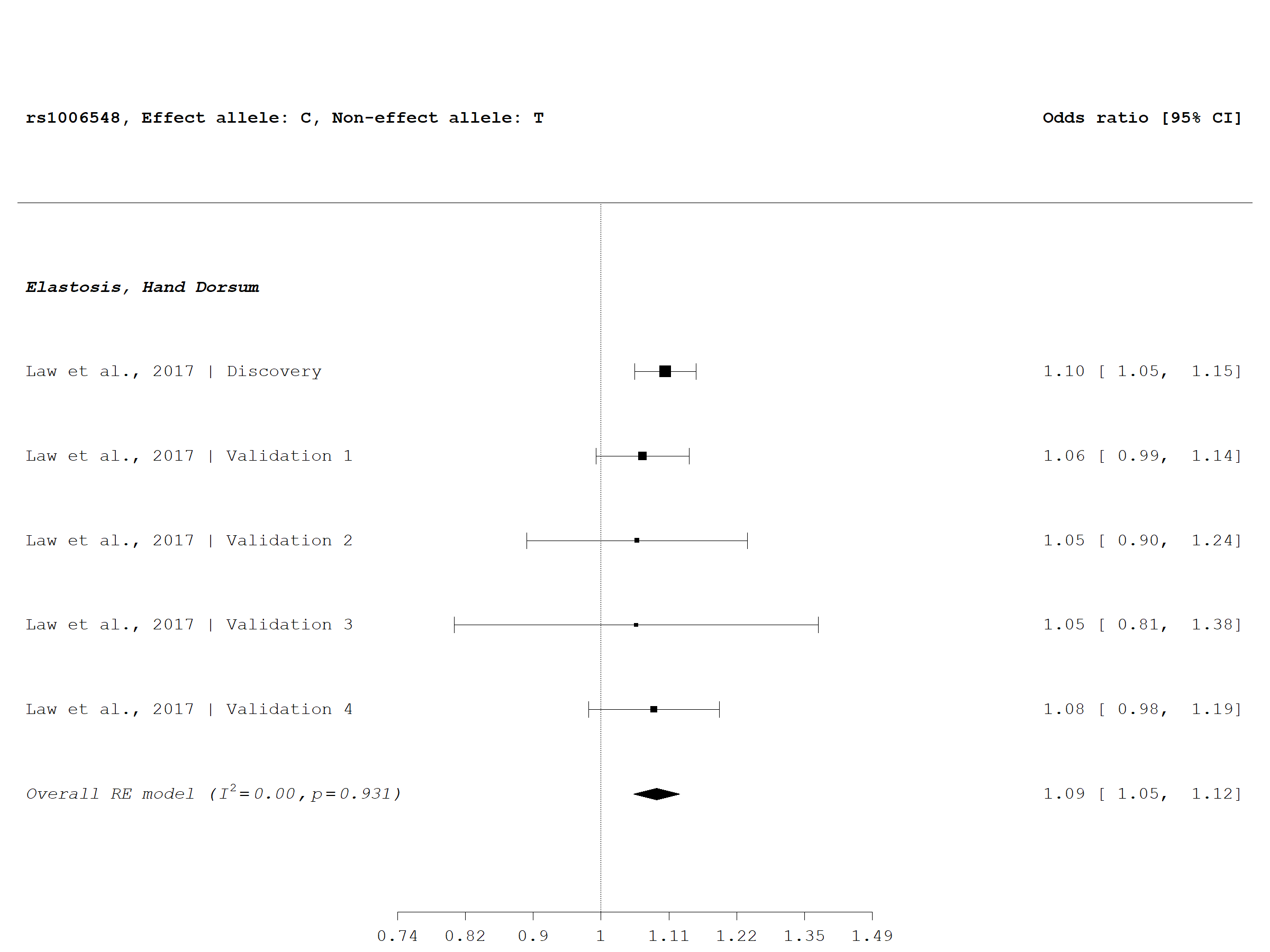

Supplement: Supplementary file 1 — Supplementary Information 1. [file 41598_2022_17443_MOESM1_ESM.zip › Supplementary Datasets/Dataset S3 - Forest Plots/fp138_rs1006548.png]

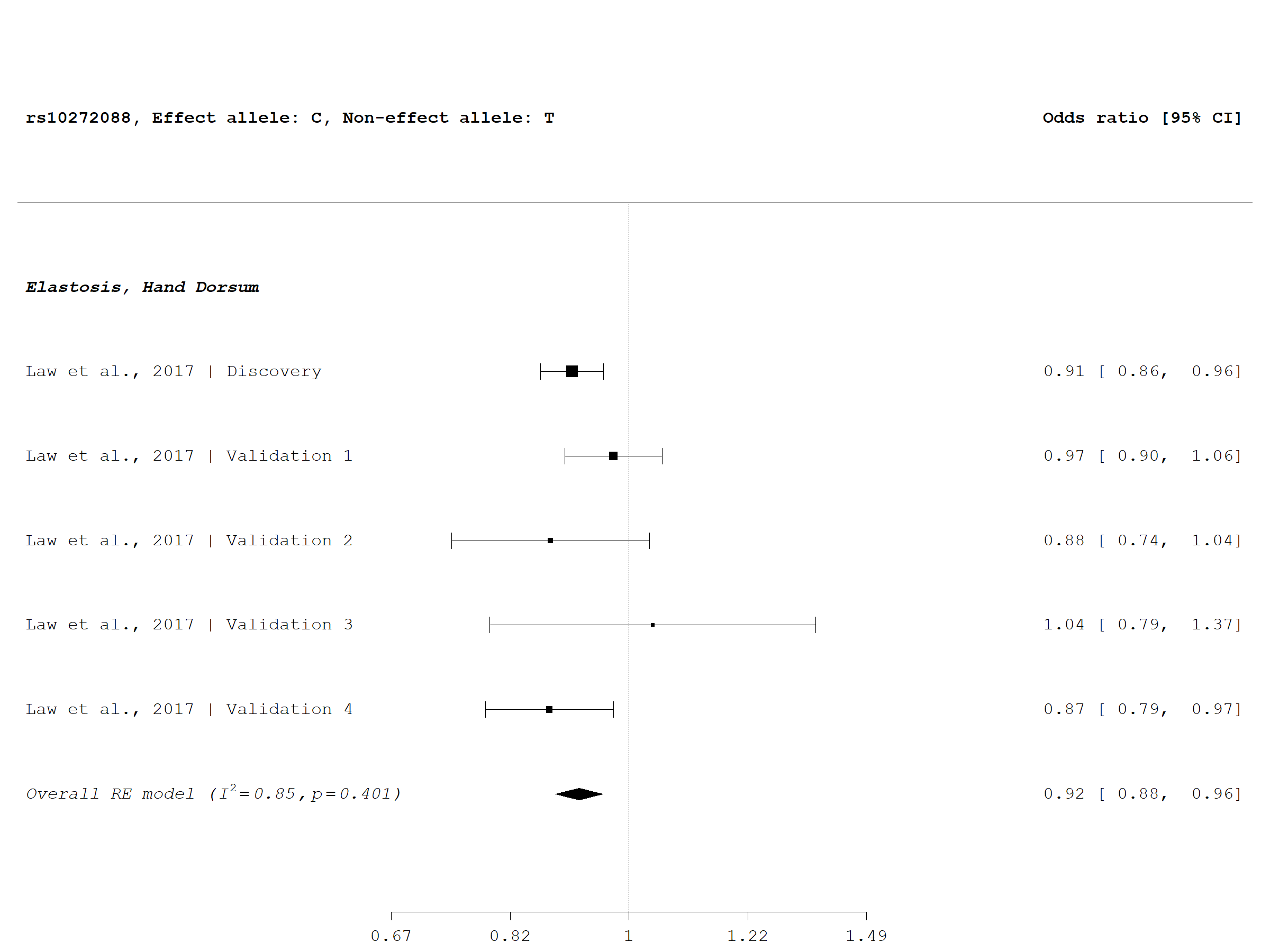

Supplement: Supplementary file 1 — Supplementary Information 1. [file 41598_2022_17443_MOESM1_ESM.zip › Supplementary Datasets/Dataset S3 - Forest Plots/fp139_rs10272088.png]

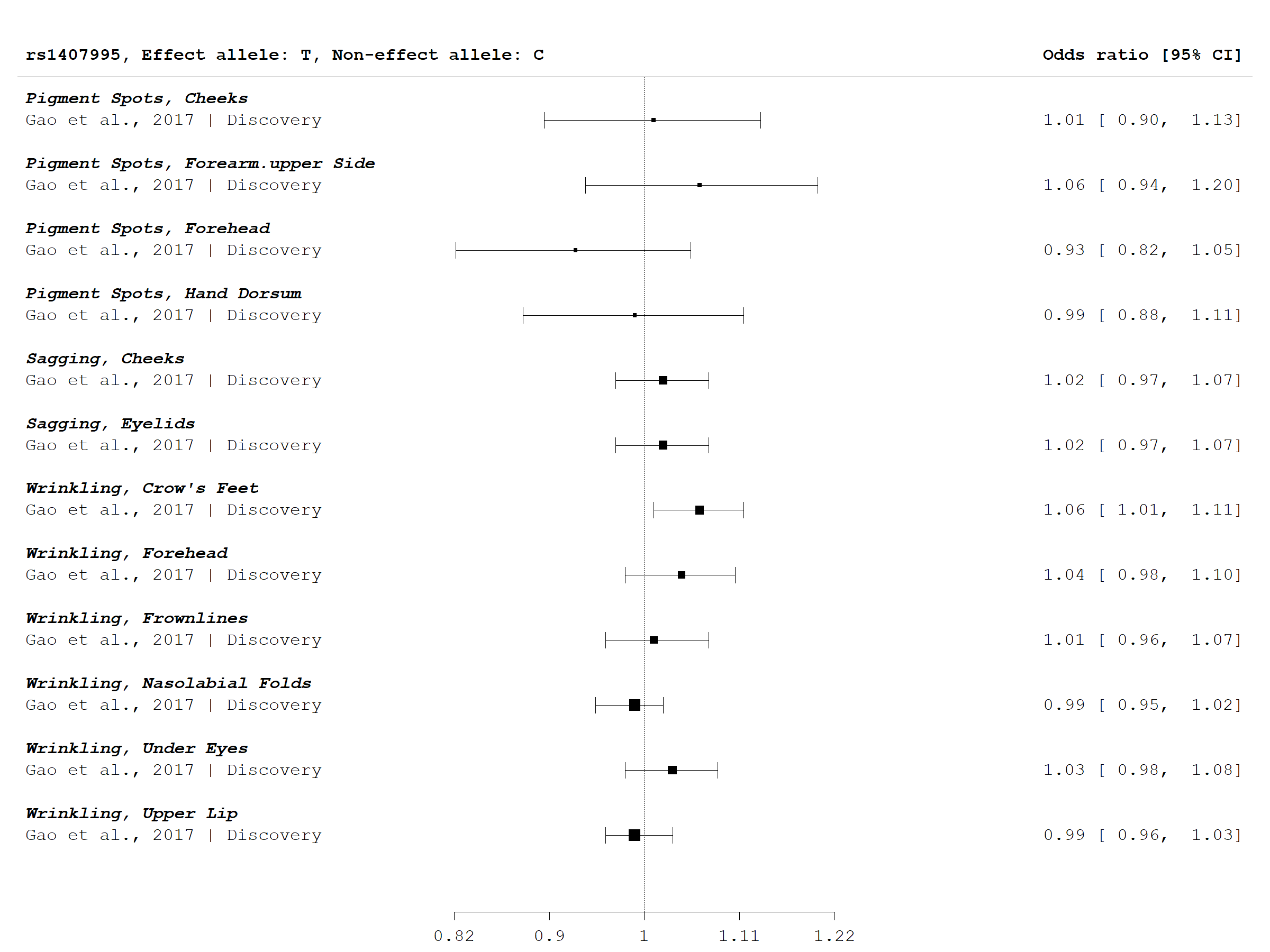

Supplement: Supplementary file 1 — Supplementary Information 1. [file 41598_2022_17443_MOESM1_ESM.zip › Supplementary Datasets/Dataset S3 - Forest Plots/fp13_rs1407995.png]

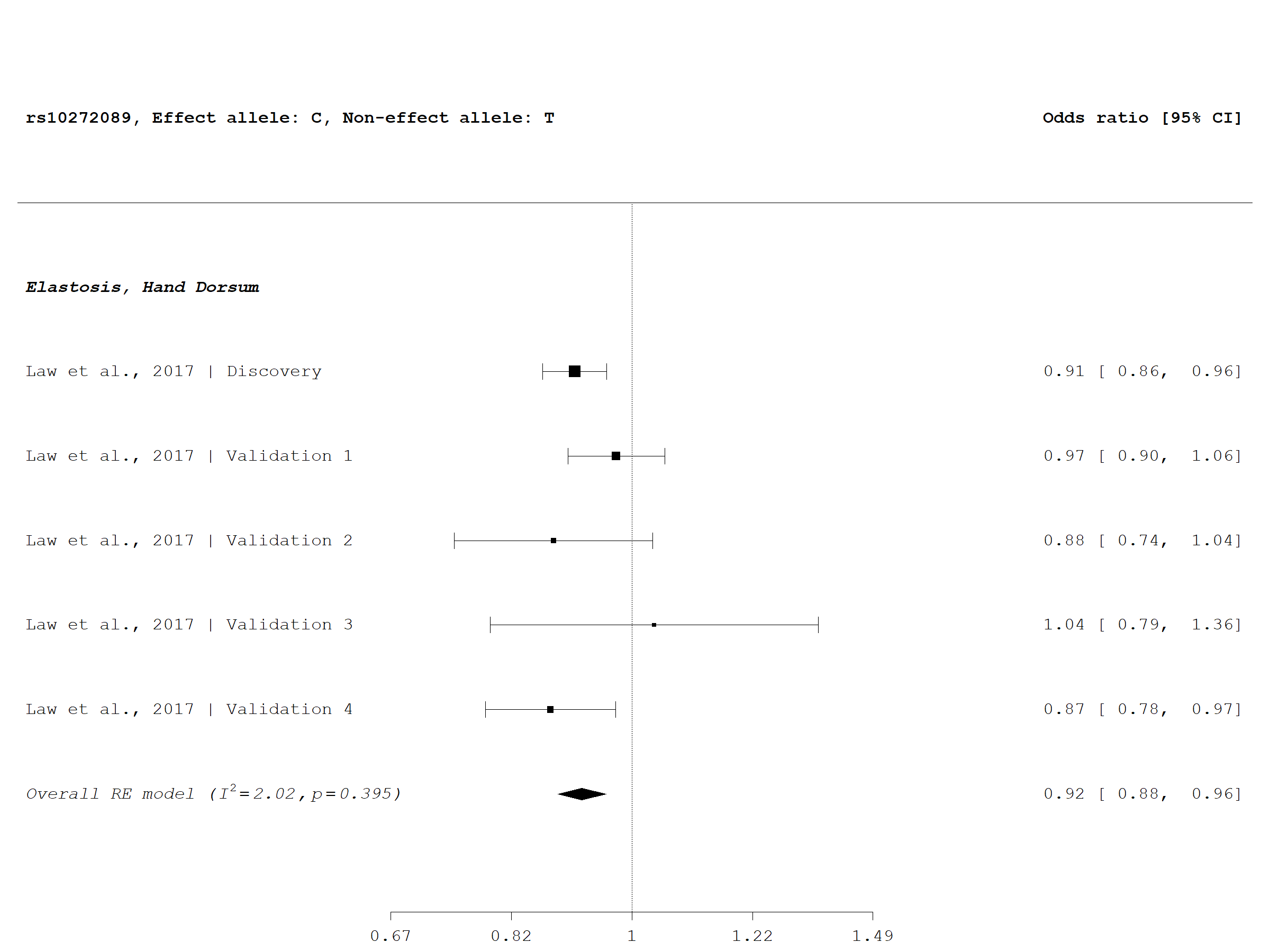

Supplement: Supplementary file 1 — Supplementary Information 1. [file 41598_2022_17443_MOESM1_ESM.zip › Supplementary Datasets/Dataset S3 - Forest Plots/fp140_rs10272089.png]

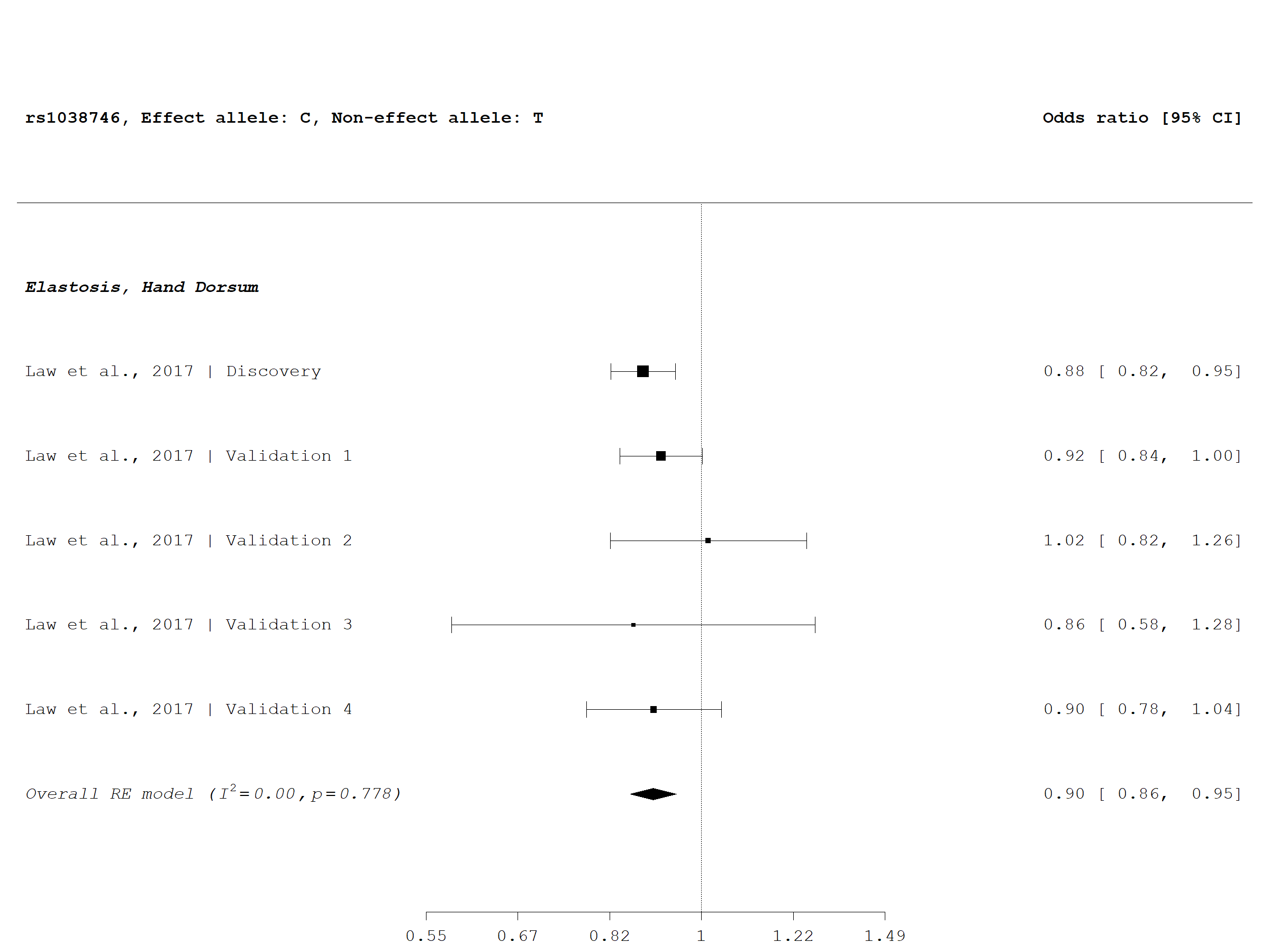

Supplement: Supplementary file 1 — Supplementary Information 1. [file 41598_2022_17443_MOESM1_ESM.zip › Supplementary Datasets/Dataset S3 - Forest Plots/fp141_rs1038746.png]

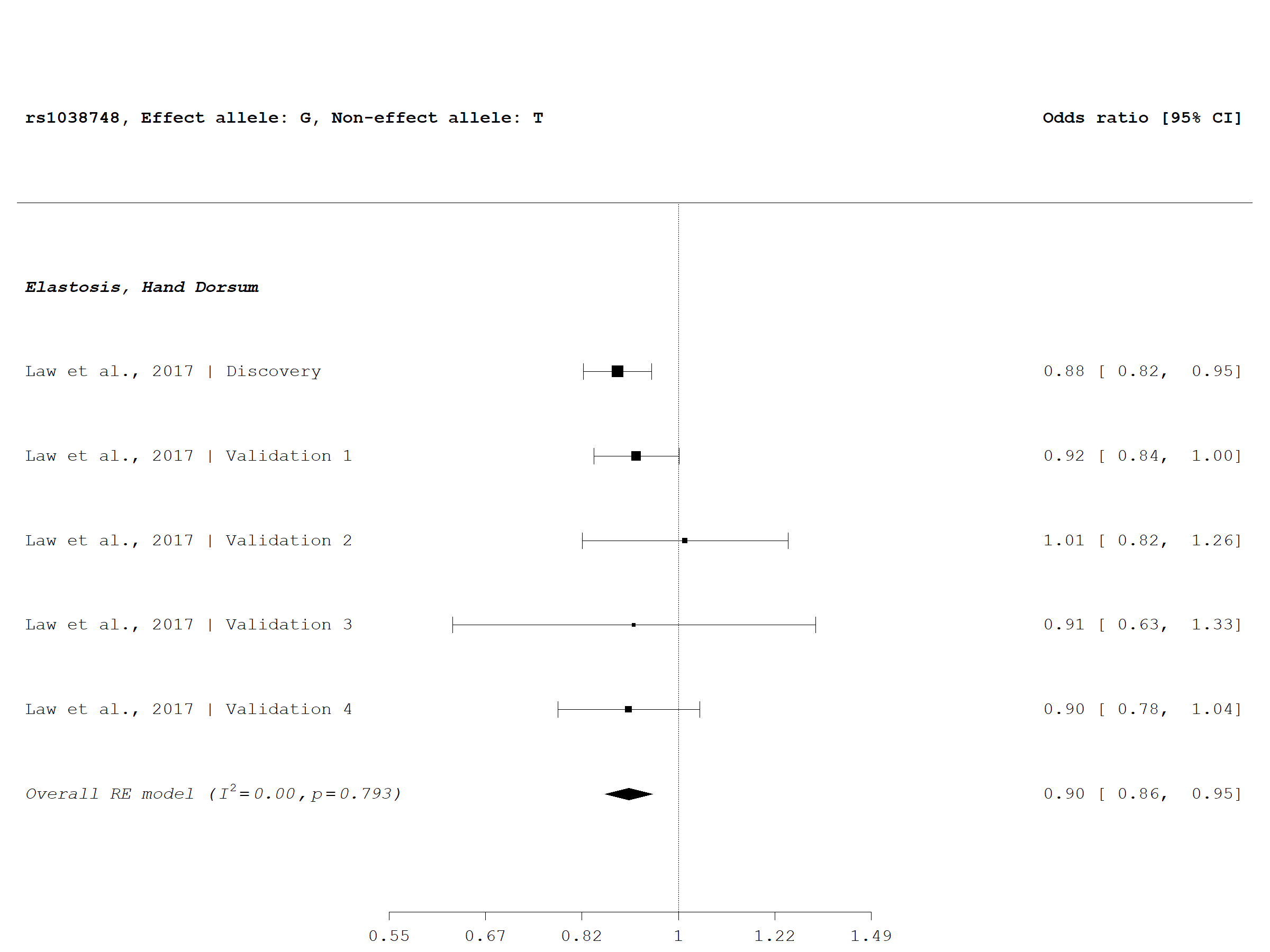

Supplement: Supplementary file 1 — Supplementary Information 1. [file 41598_2022_17443_MOESM1_ESM.zip › Supplementary Datasets/Dataset S3 - Forest Plots/fp142_rs1038748.png]

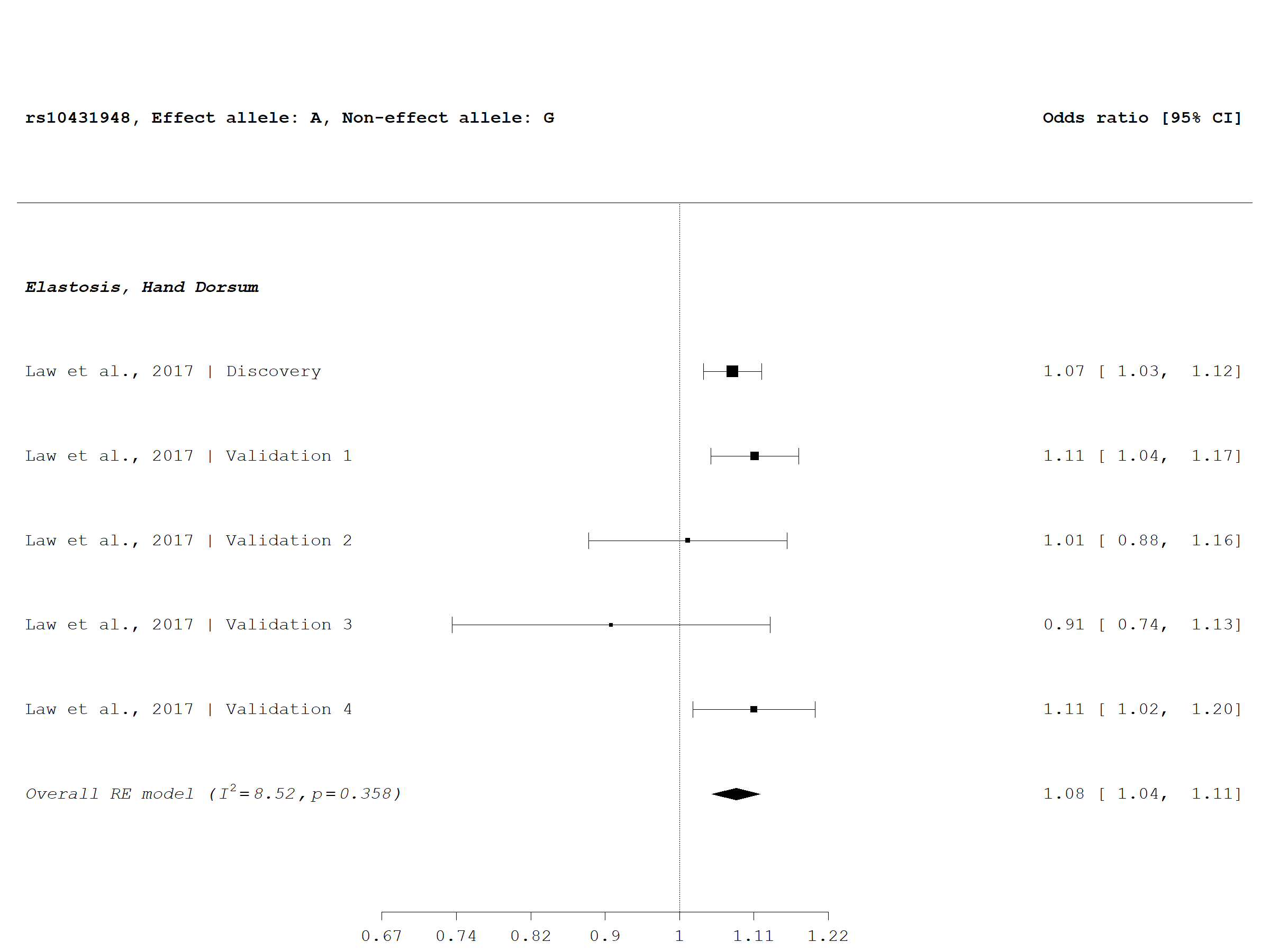

Supplement: Supplementary file 1 — Supplementary Information 1. [file 41598_2022_17443_MOESM1_ESM.zip › Supplementary Datasets/Dataset S3 - Forest Plots/fp143_rs10431948.png]

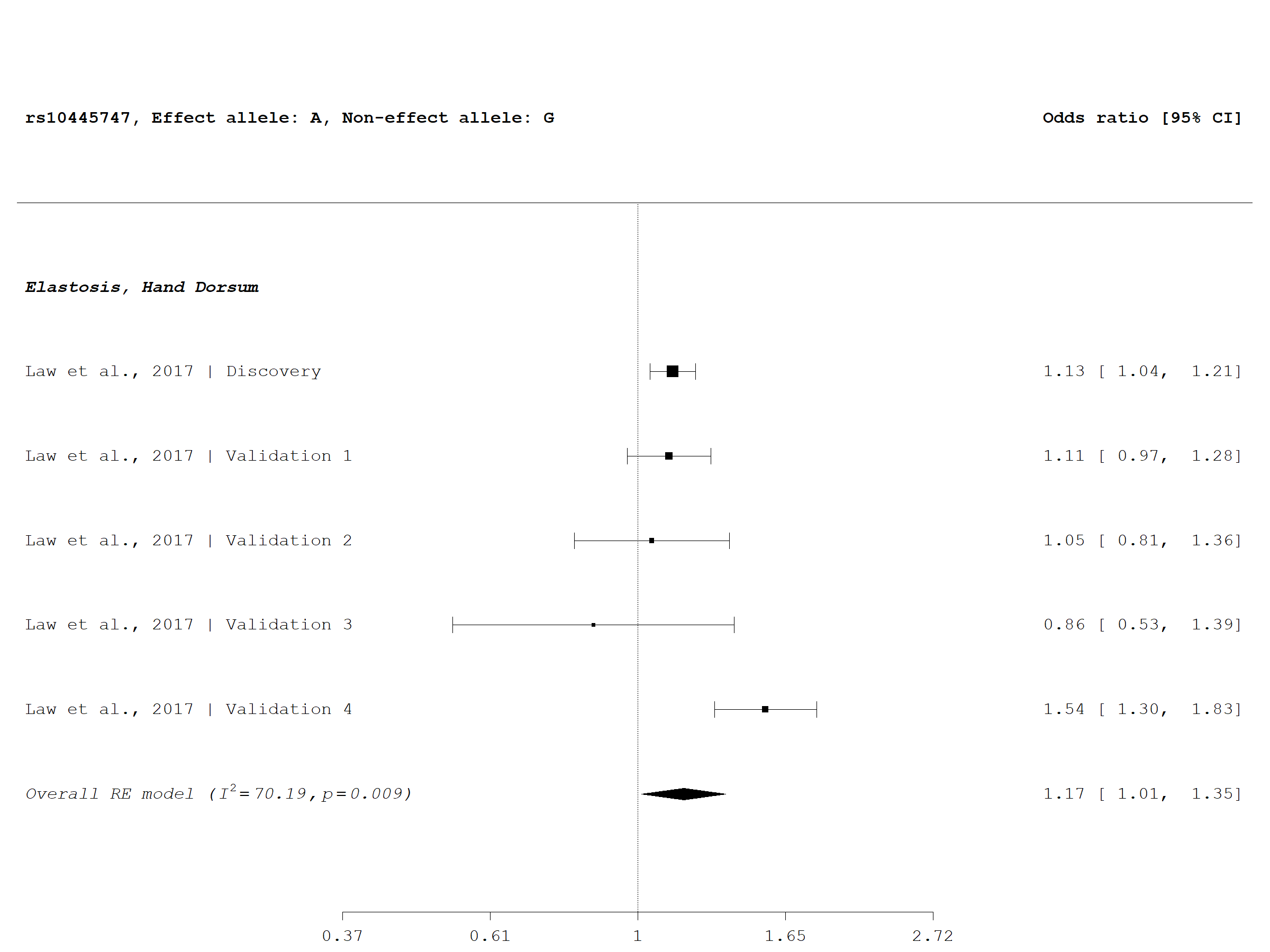

Supplement: Supplementary file 1 — Supplementary Information 1. [file 41598_2022_17443_MOESM1_ESM.zip › Supplementary Datasets/Dataset S3 - Forest Plots/fp144_rs10445747.png]

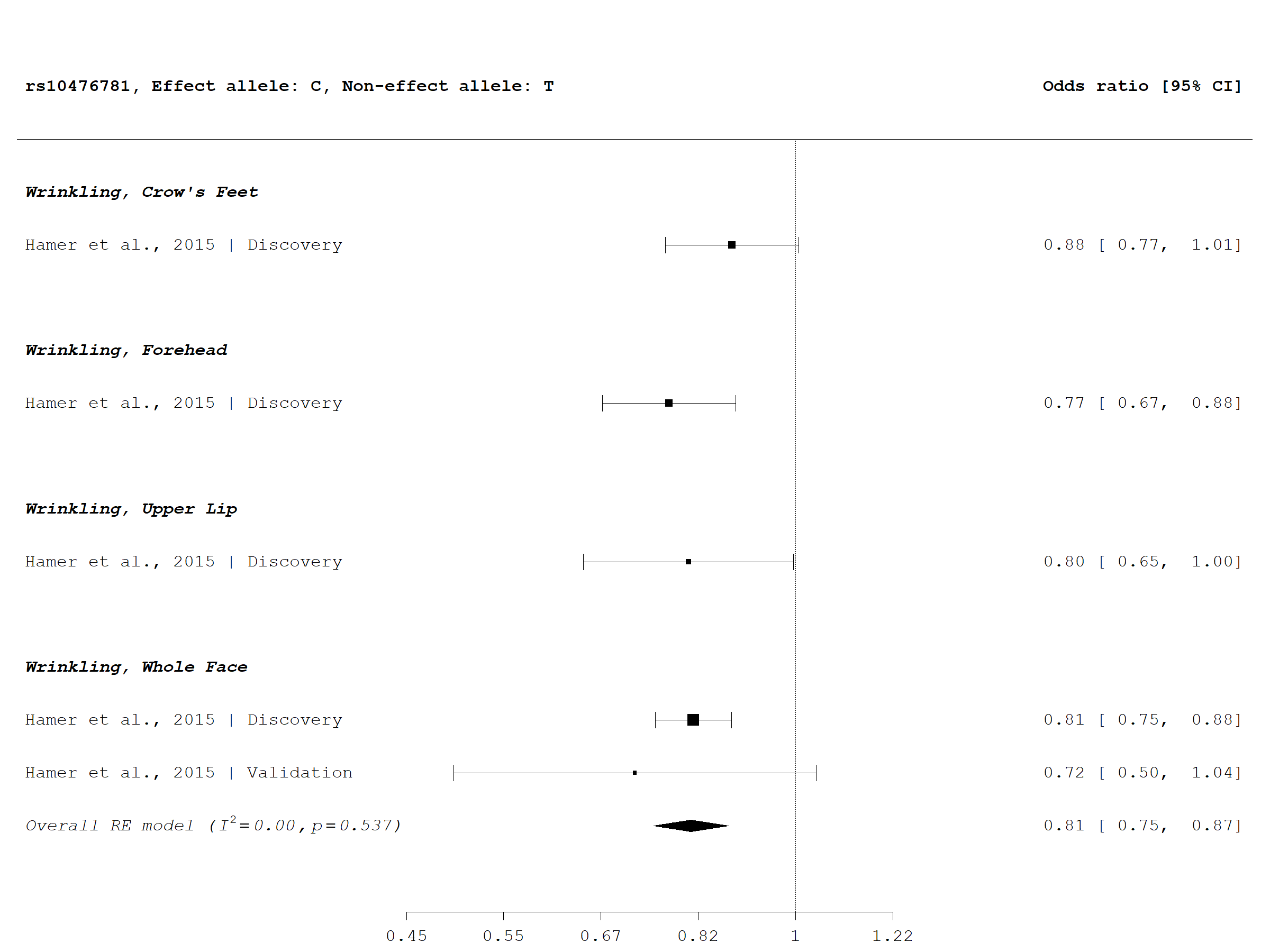

Supplement: Supplementary file 1 — Supplementary Information 1. [file 41598_2022_17443_MOESM1_ESM.zip › Supplementary Datasets/Dataset S3 - Forest Plots/fp145_rs10476781.png]

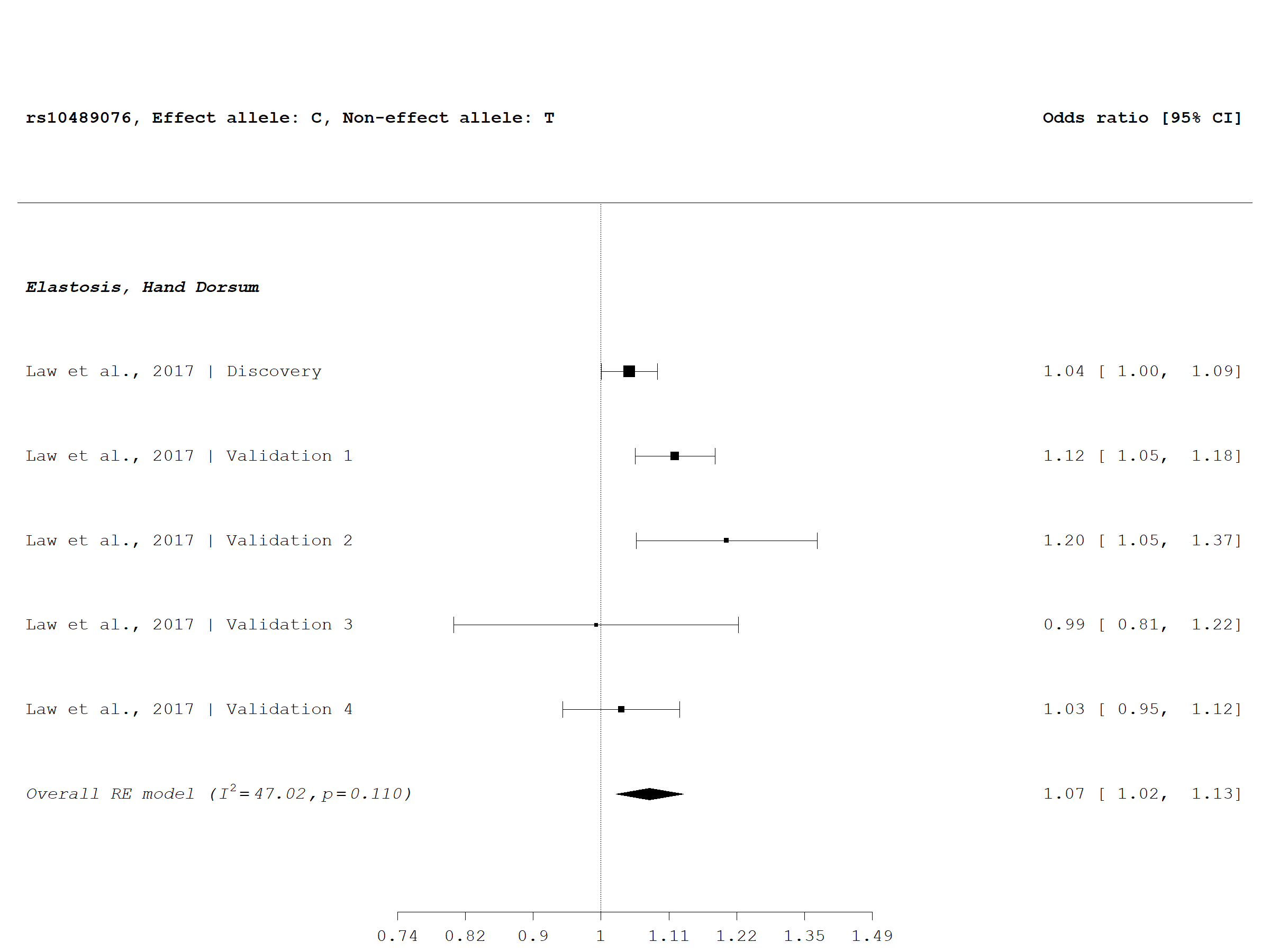

Supplement: Supplementary file 1 — Supplementary Information 1. [file 41598_2022_17443_MOESM1_ESM.zip › Supplementary Datasets/Dataset S3 - Forest Plots/fp146_rs10489076.png]

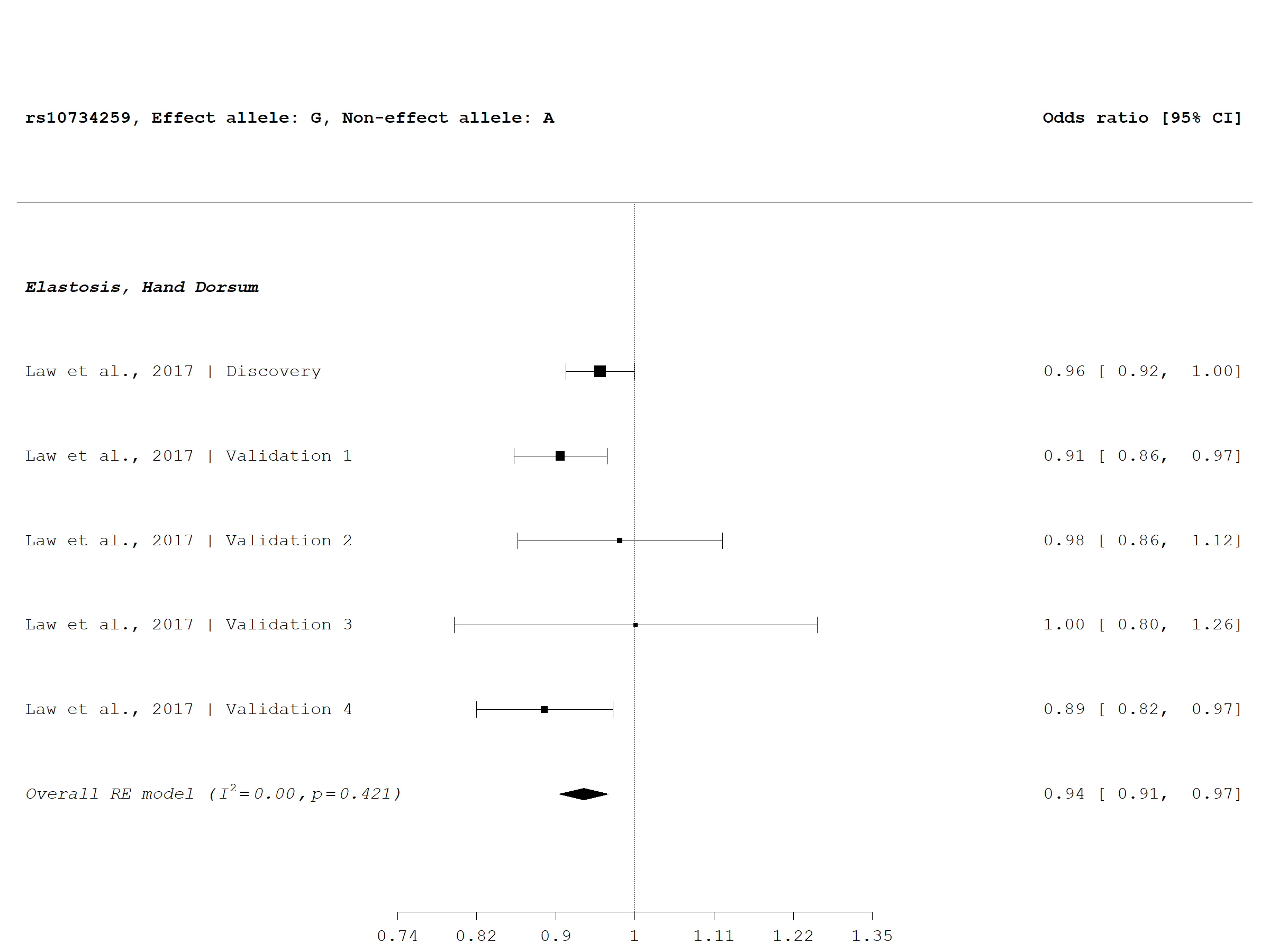

Supplement: Supplementary file 1 — Supplementary Information 1. [file 41598_2022_17443_MOESM1_ESM.zip › Supplementary Datasets/Dataset S3 - Forest Plots/fp147_rs10734259.png]

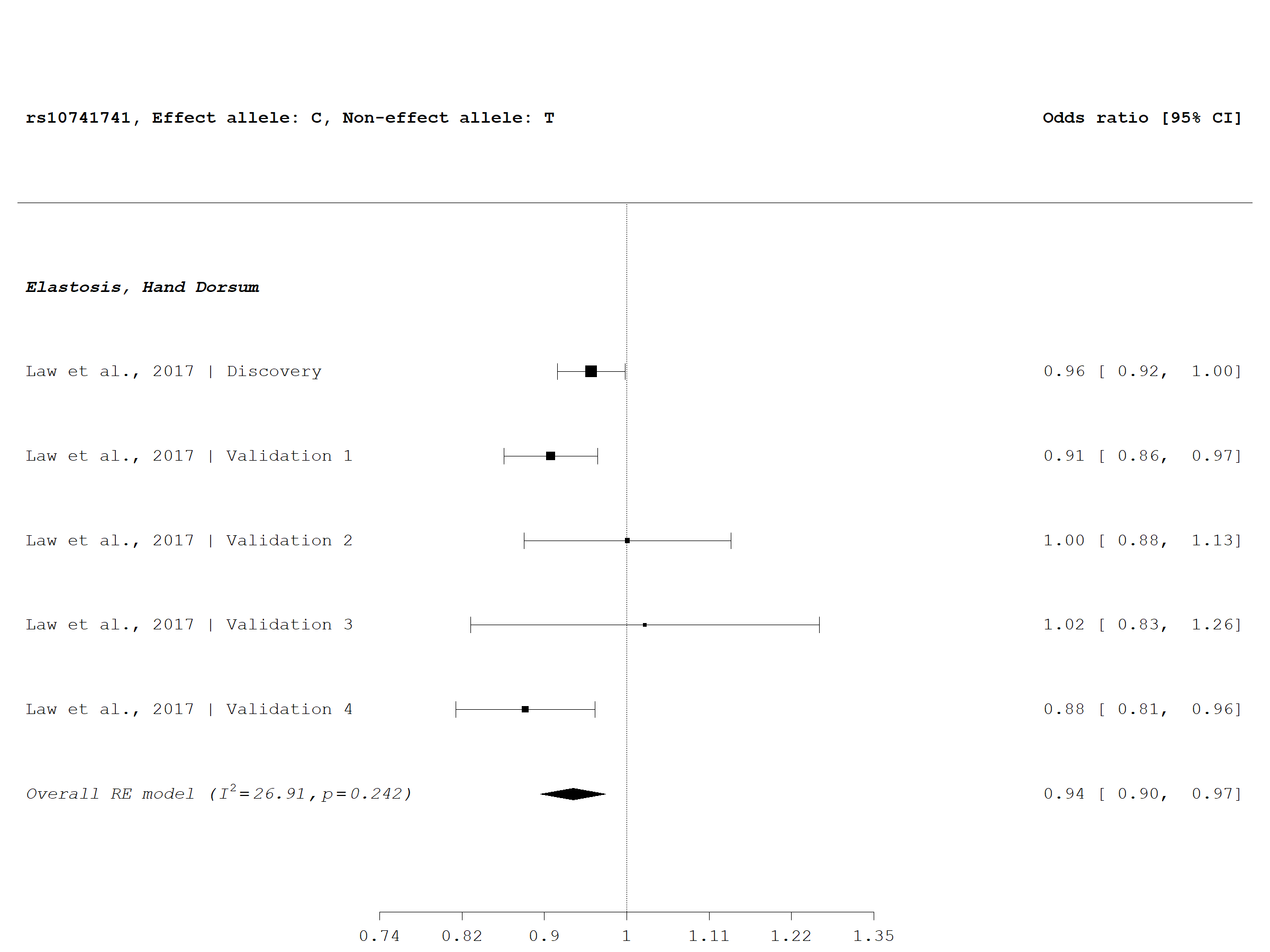

Supplement: Supplementary file 1 — Supplementary Information 1. [file 41598_2022_17443_MOESM1_ESM.zip › Supplementary Datasets/Dataset S3 - Forest Plots/fp148_rs10741741.png]

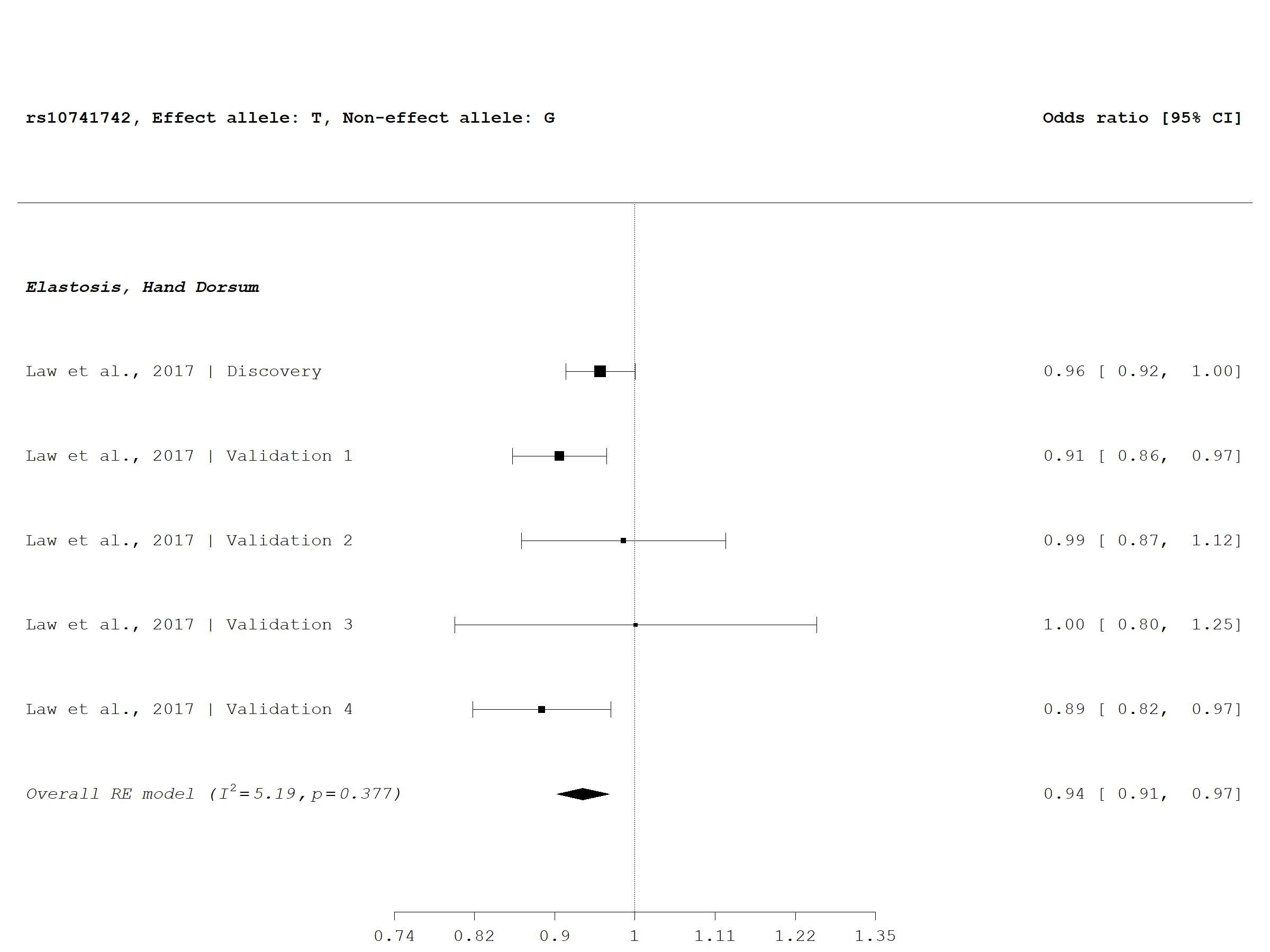

Supplement: Supplementary file 1 — Supplementary Information 1. [file 41598_2022_17443_MOESM1_ESM.zip › Supplementary Datasets/Dataset S3 - Forest Plots/fp149_rs10741742.png]

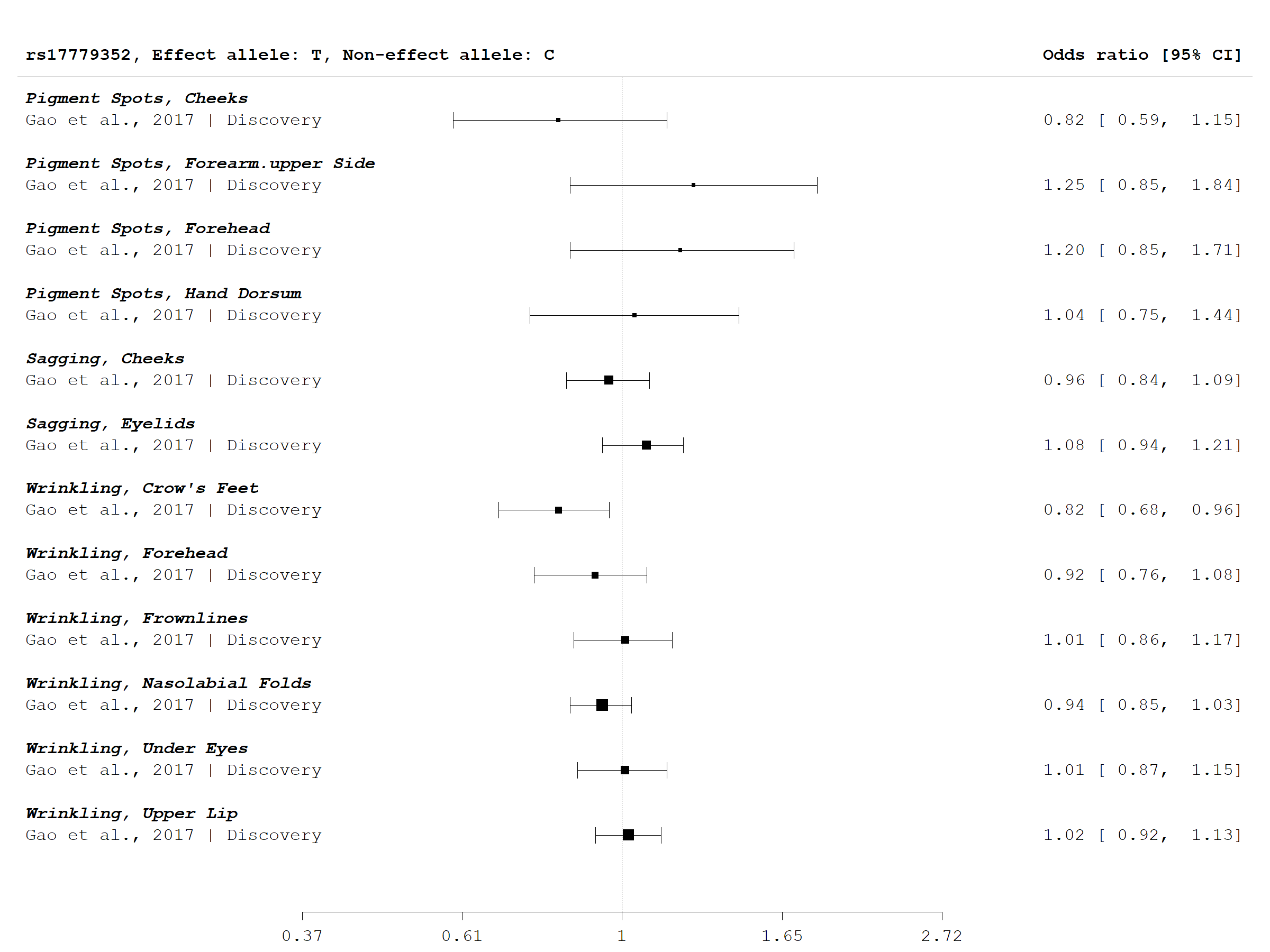

Supplement: Supplementary file 1 — Supplementary Information 1. [file 41598_2022_17443_MOESM1_ESM.zip › Supplementary Datasets/Dataset S3 - Forest Plots/fp14_rs17779352.png]

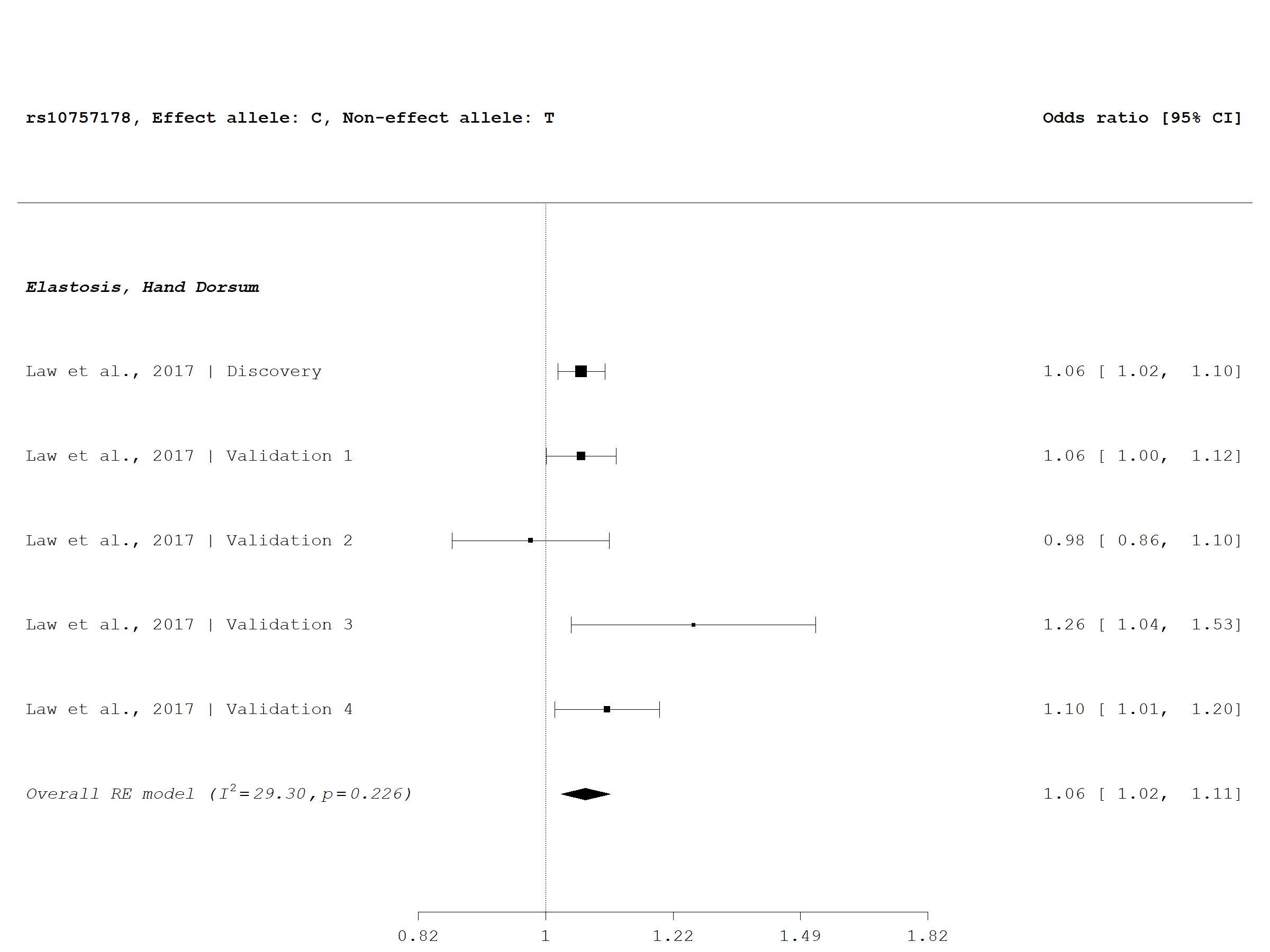

Supplement: Supplementary file 1 — Supplementary Information 1. [file 41598_2022_17443_MOESM1_ESM.zip › Supplementary Datasets/Dataset S3 - Forest Plots/fp150_rs10757178.png]

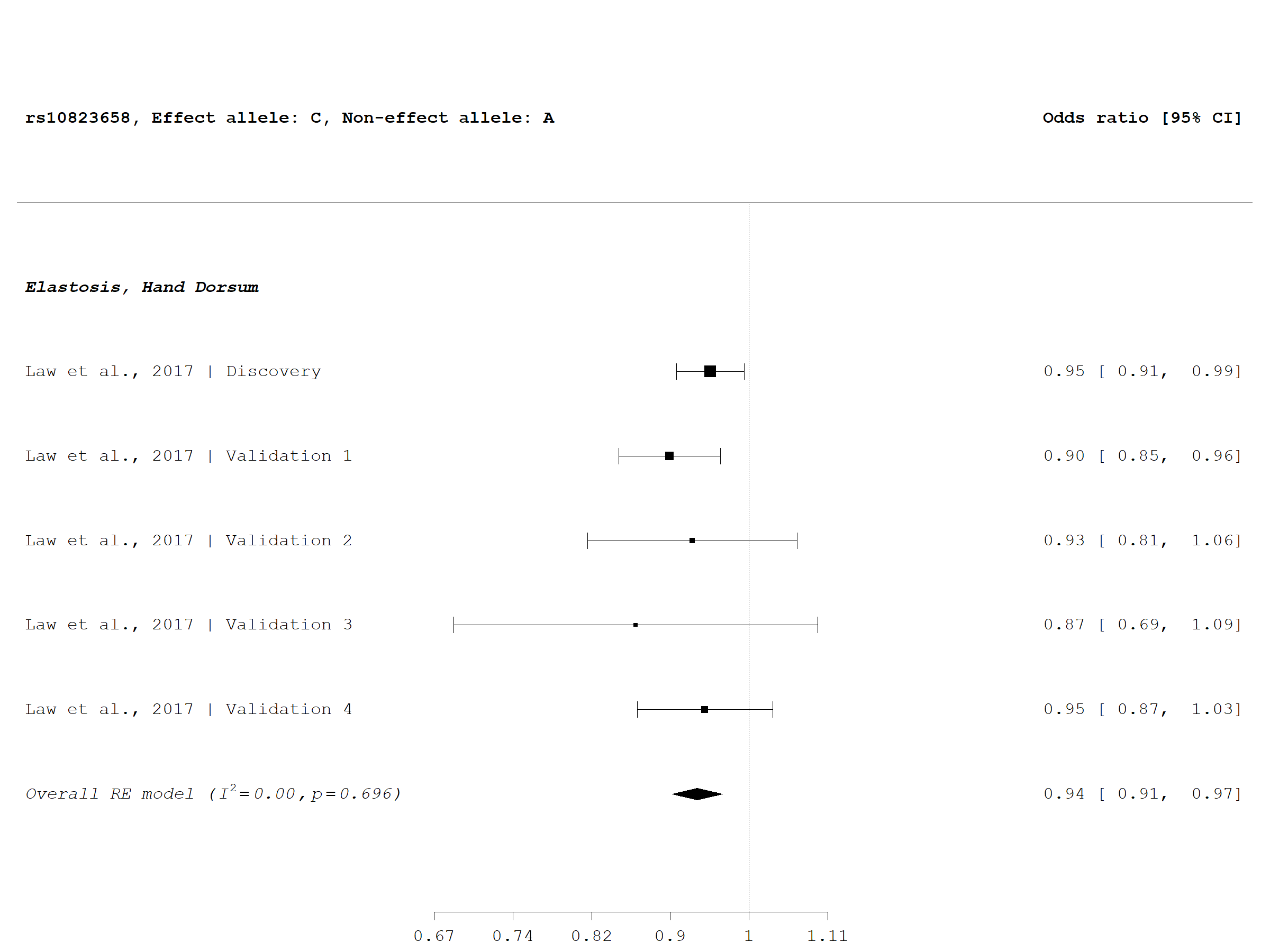

Supplement: Supplementary file 1 — Supplementary Information 1. [file 41598_2022_17443_MOESM1_ESM.zip › Supplementary Datasets/Dataset S3 - Forest Plots/fp151_rs10823658.png]

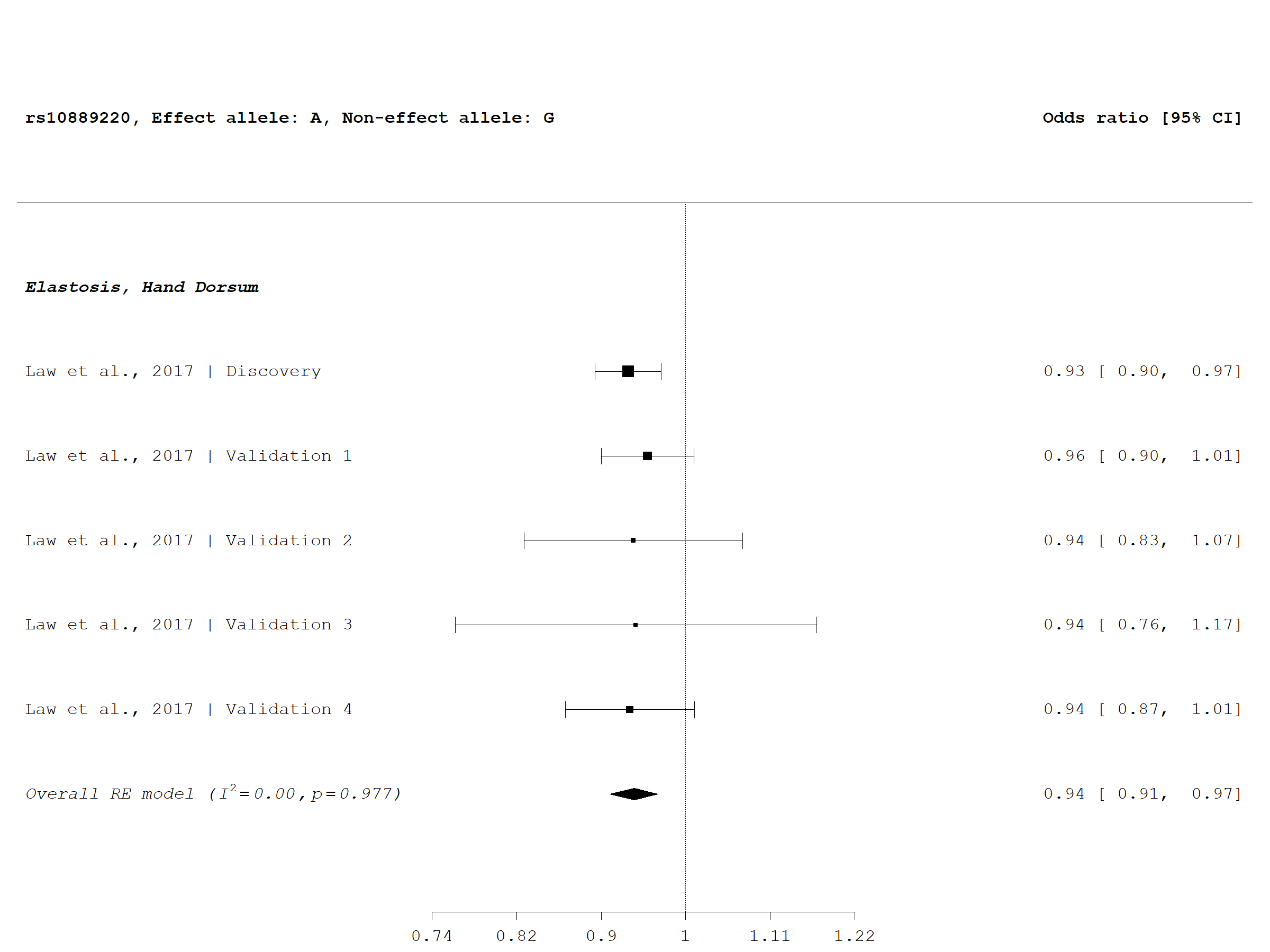

Supplement: Supplementary file 1 — Supplementary Information 1. [file 41598_2022_17443_MOESM1_ESM.zip › Supplementary Datasets/Dataset S3 - Forest Plots/fp152_rs10889220.png]

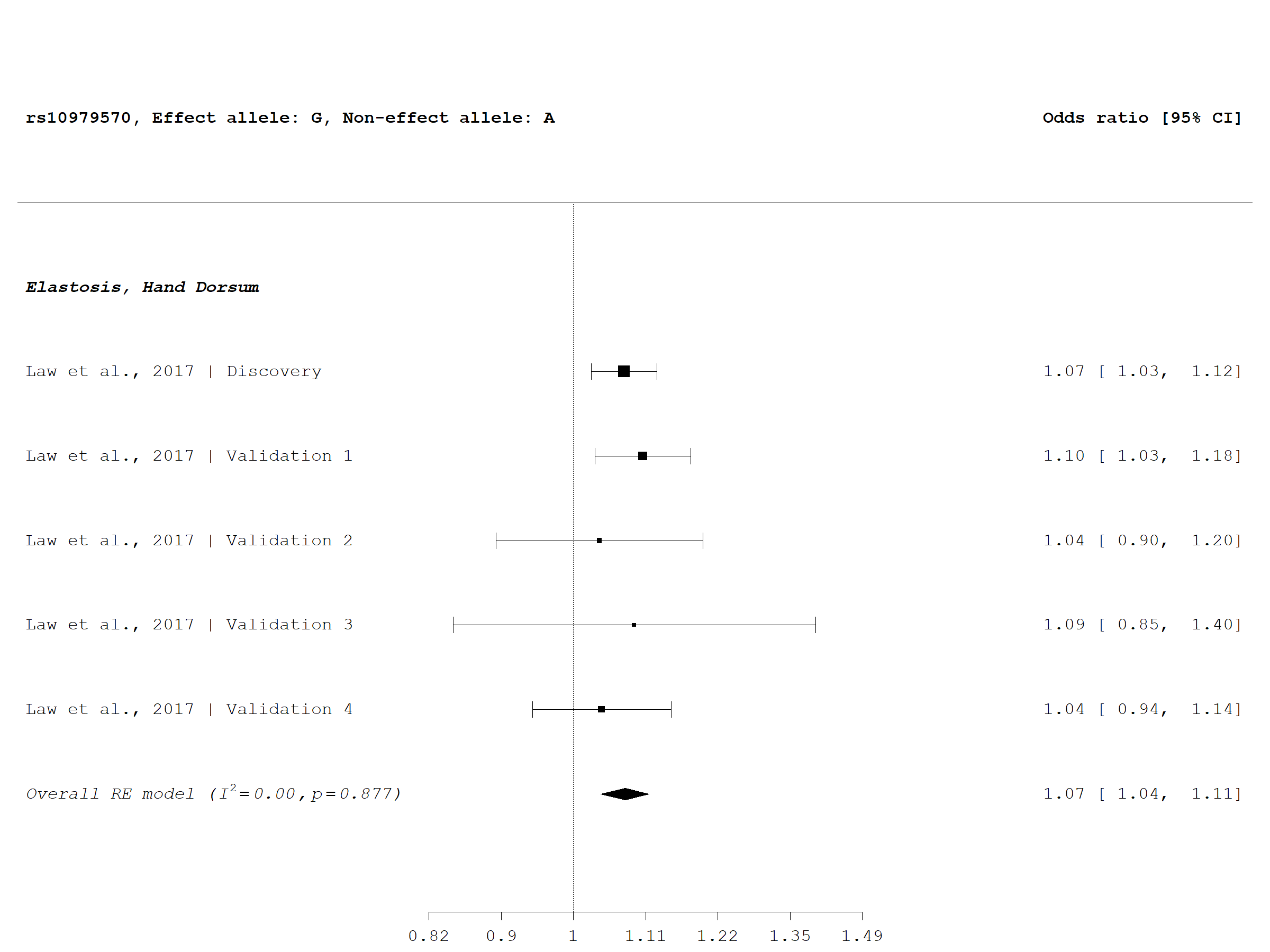

Supplement: Supplementary file 1 — Supplementary Information 1. [file 41598_2022_17443_MOESM1_ESM.zip › Supplementary Datasets/Dataset S3 - Forest Plots/fp153_rs10979570.png]

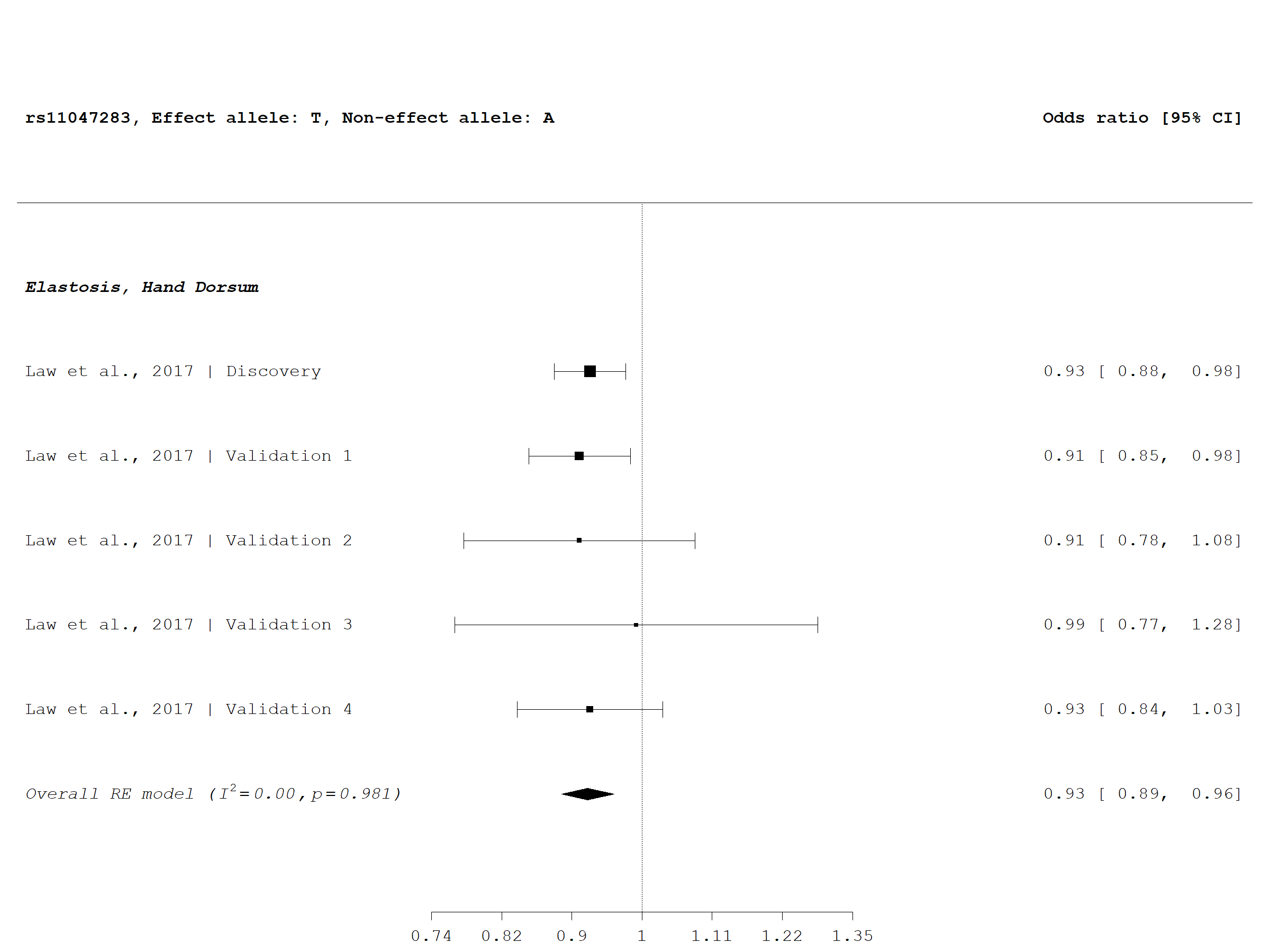

Supplement: Supplementary file 1 — Supplementary Information 1. [file 41598_2022_17443_MOESM1_ESM.zip › Supplementary Datasets/Dataset S3 - Forest Plots/fp154_rs11047283.png]

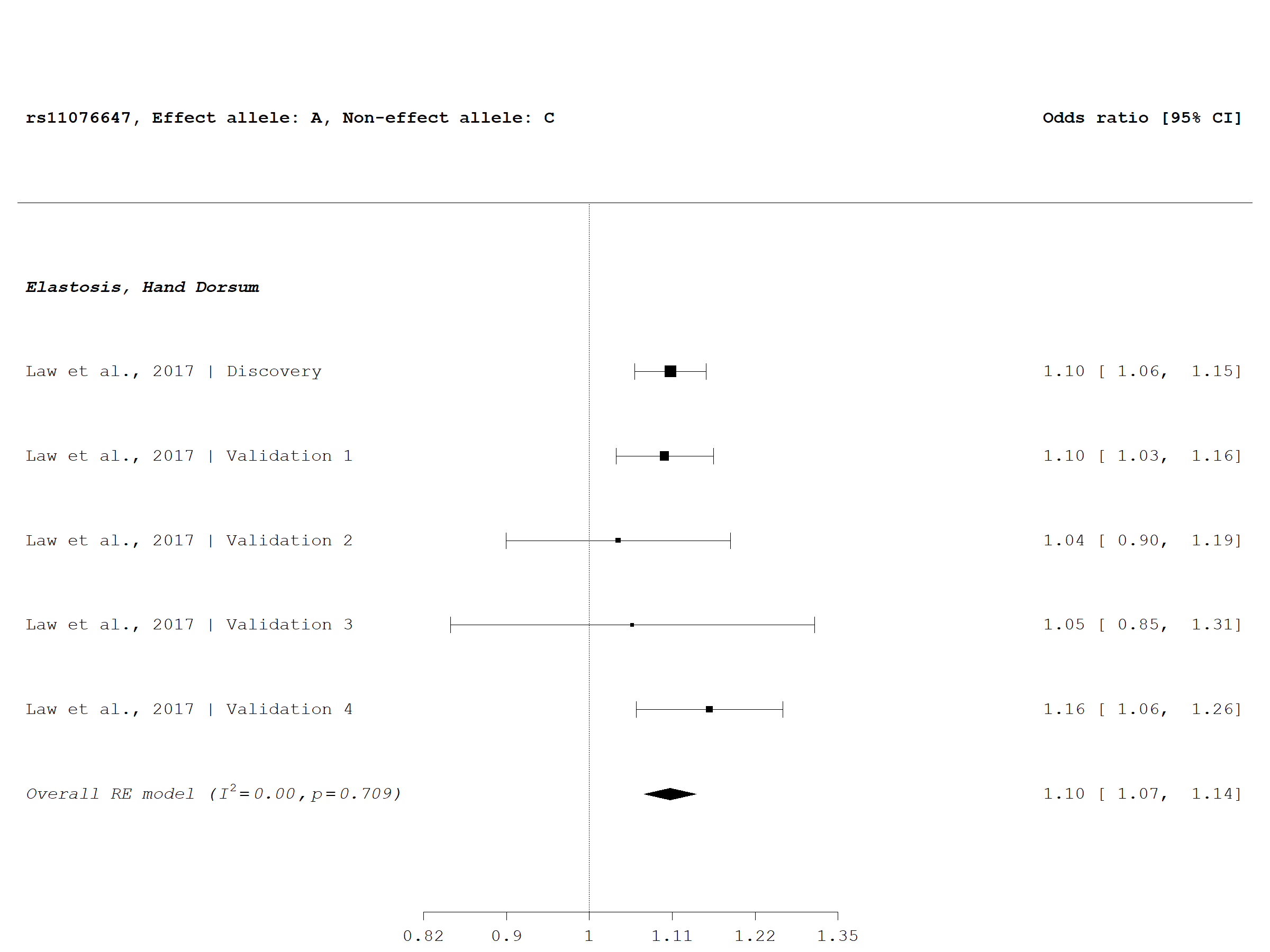

Supplement: Supplementary file 1 — Supplementary Information 1. [file 41598_2022_17443_MOESM1_ESM.zip › Supplementary Datasets/Dataset S3 - Forest Plots/fp155_rs11076647.png]

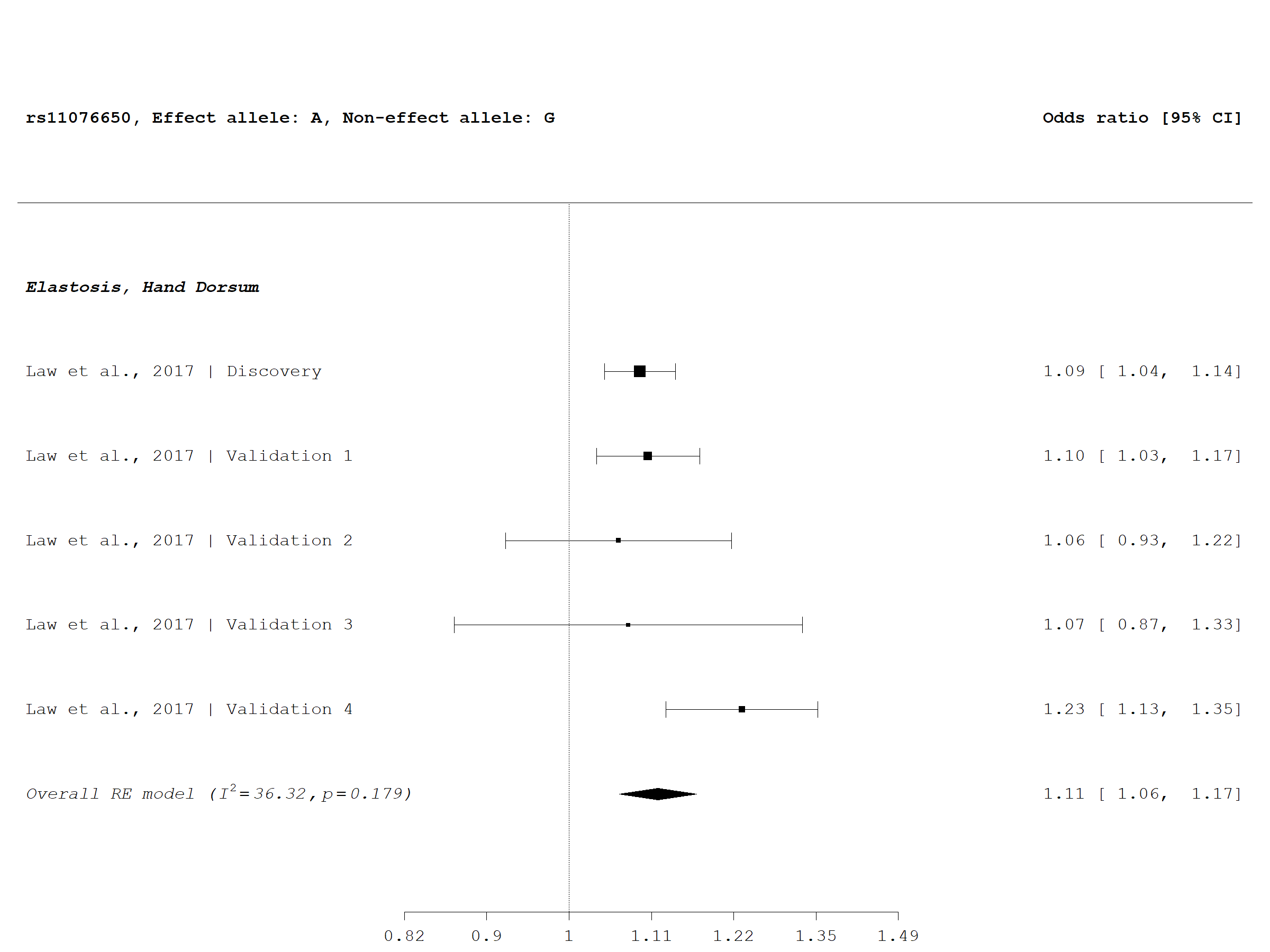

Supplement: Supplementary file 1 — Supplementary Information 1. [file 41598_2022_17443_MOESM1_ESM.zip › Supplementary Datasets/Dataset S3 - Forest Plots/fp156_rs11076650.png]

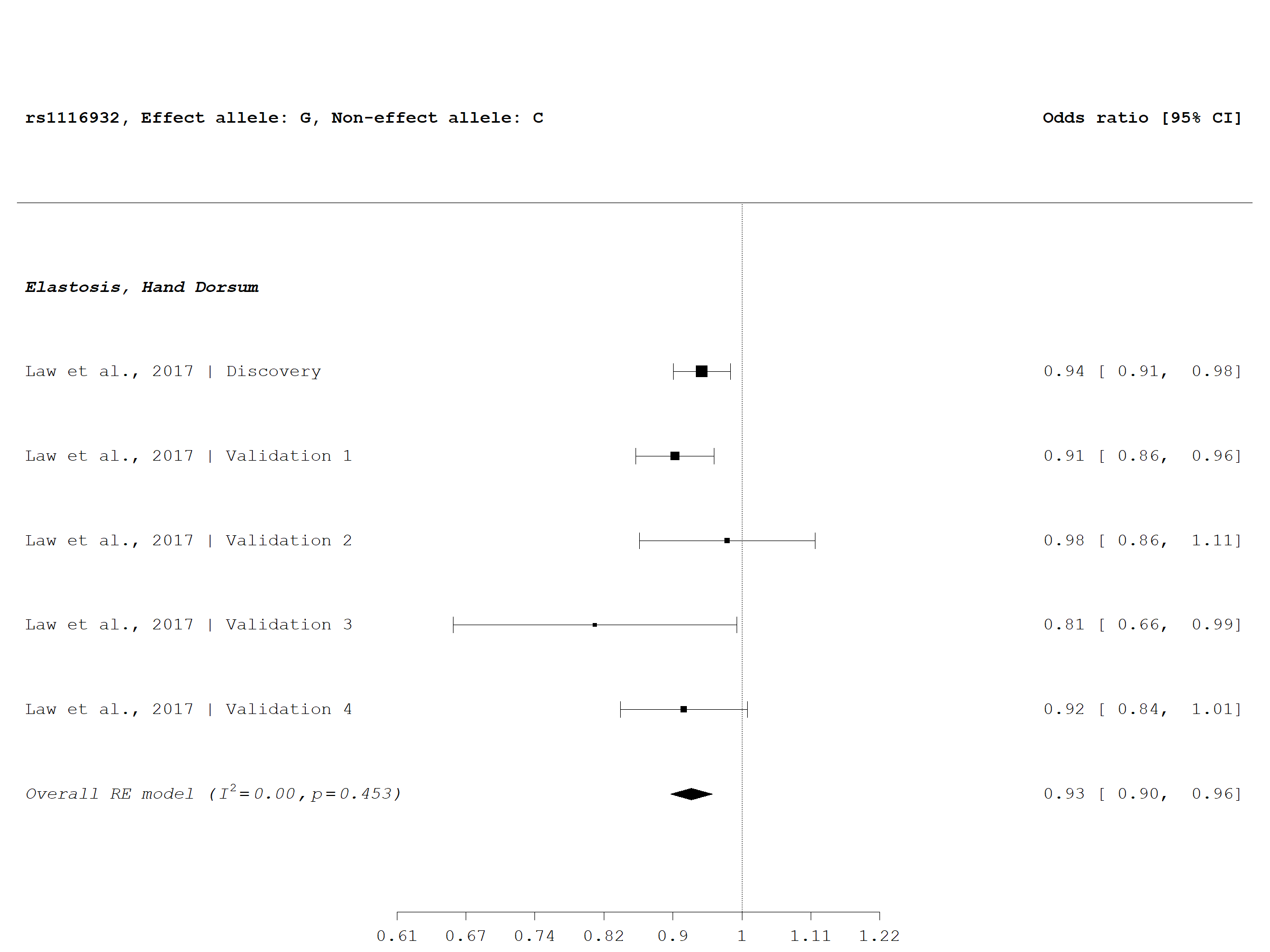

Supplement: Supplementary file 1 — Supplementary Information 1. [file 41598_2022_17443_MOESM1_ESM.zip › Supplementary Datasets/Dataset S3 - Forest Plots/fp157_rs1116932.png]

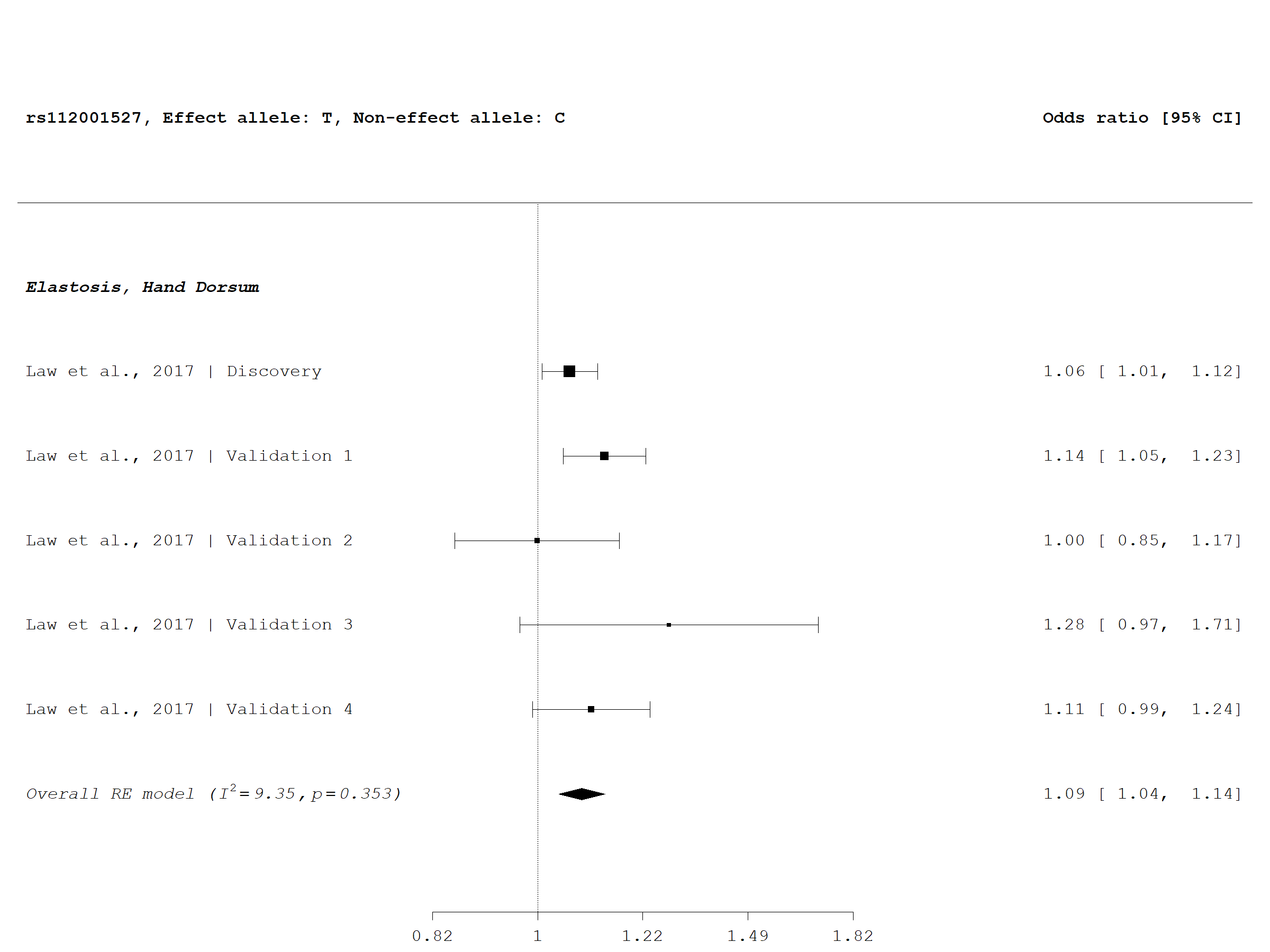

Supplement: Supplementary file 1 — Supplementary Information 1. [file 41598_2022_17443_MOESM1_ESM.zip › Supplementary Datasets/Dataset S3 - Forest Plots/fp158_rs112001527.png]

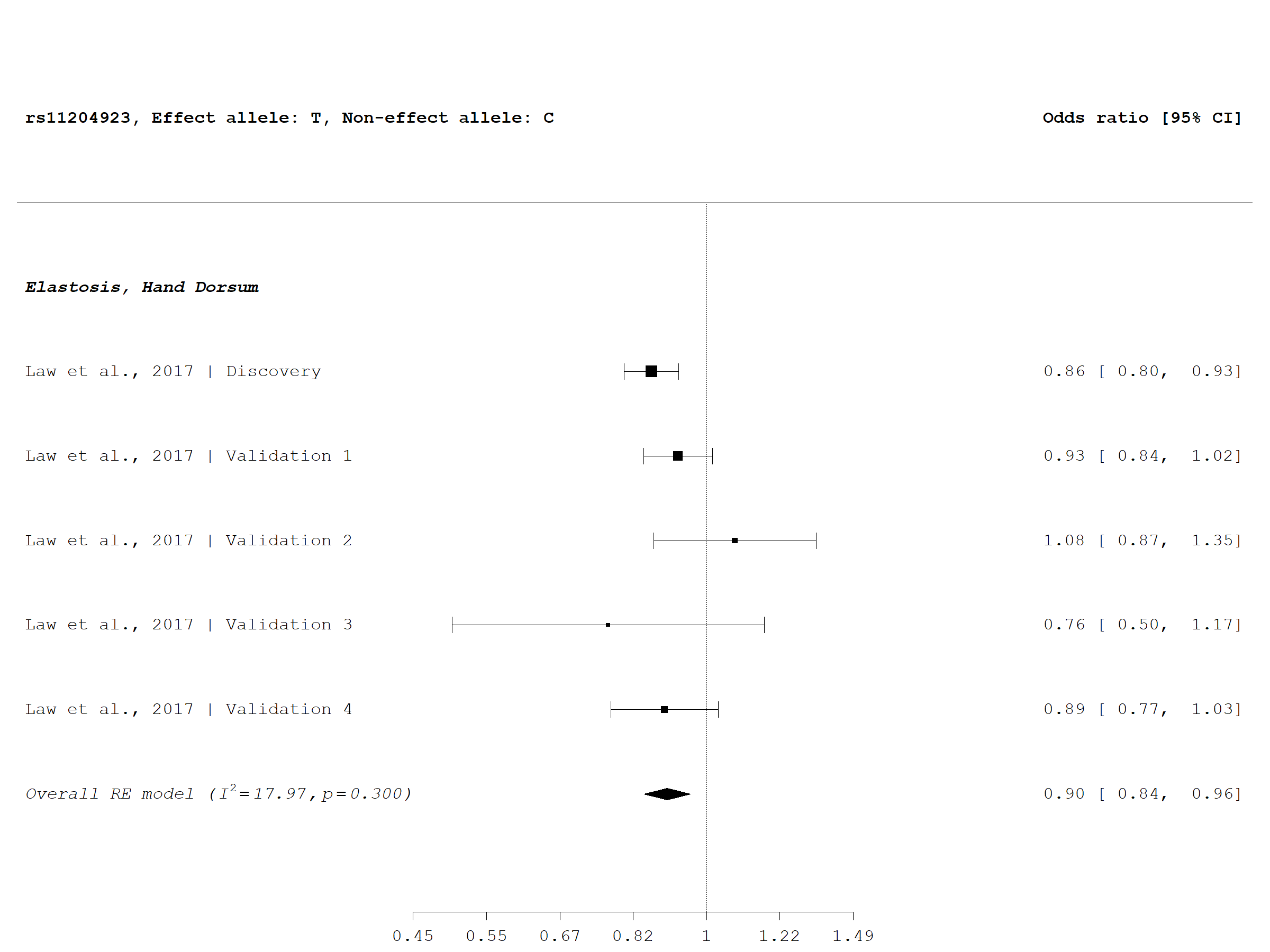

Supplement: Supplementary file 1 — Supplementary Information 1. [file 41598_2022_17443_MOESM1_ESM.zip › Supplementary Datasets/Dataset S3 - Forest Plots/fp159_rs11204923.png]

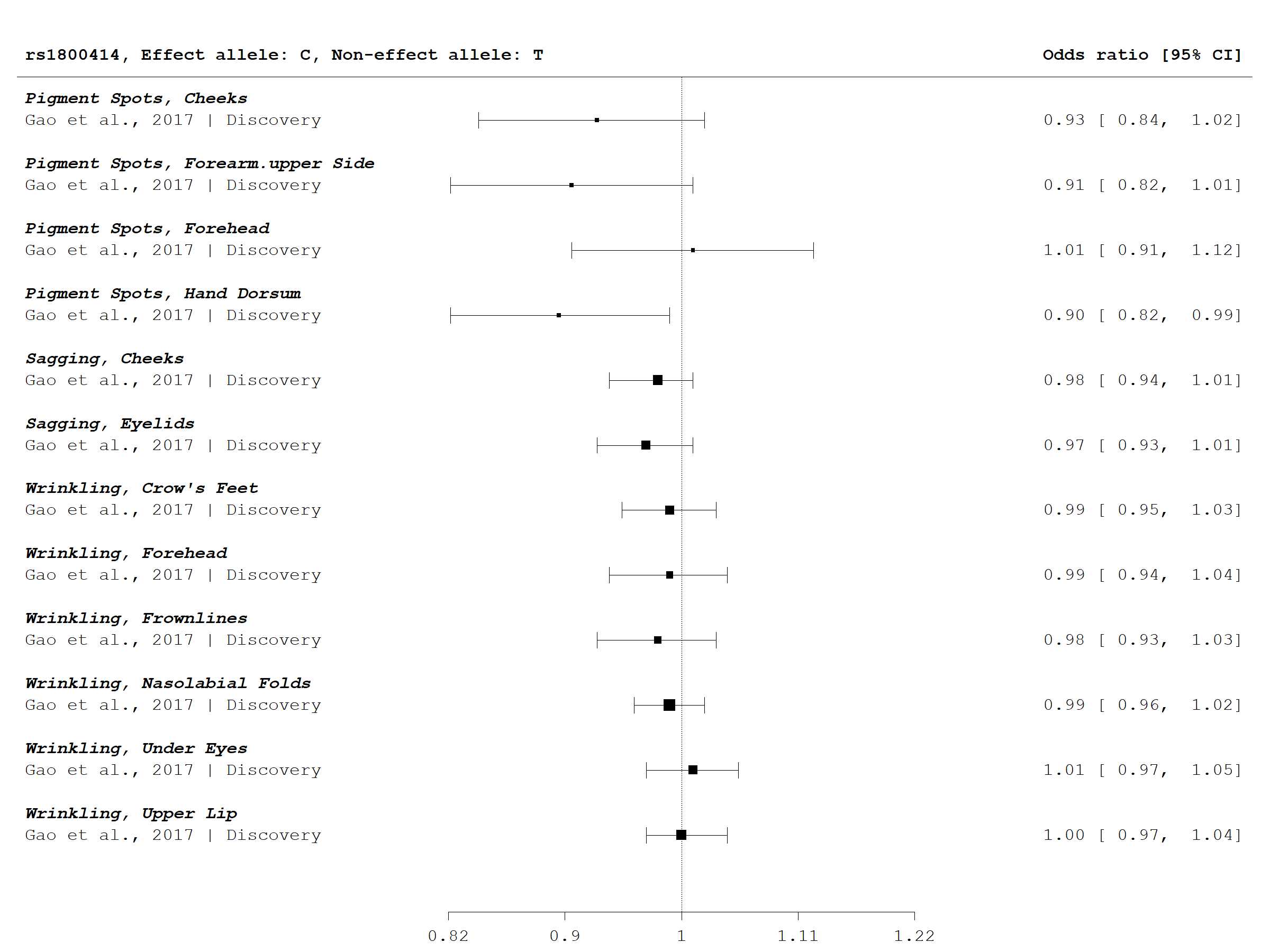

Supplement: Supplementary file 1 — Supplementary Information 1. [file 41598_2022_17443_MOESM1_ESM.zip › Supplementary Datasets/Dataset S3 - Forest Plots/fp15_rs1800414.png]

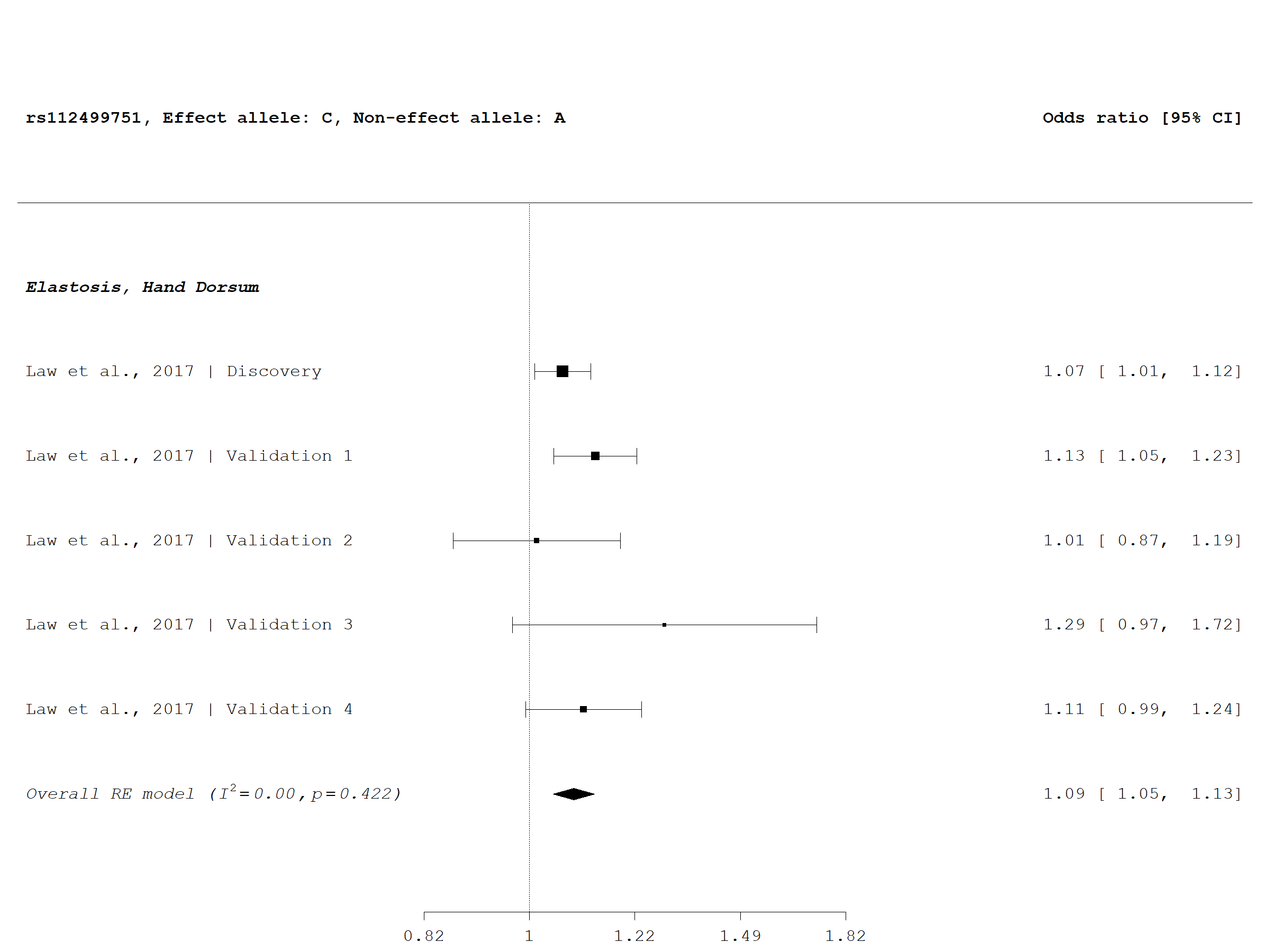

Supplement: Supplementary file 1 — Supplementary Information 1. [file 41598_2022_17443_MOESM1_ESM.zip › Supplementary Datasets/Dataset S3 - Forest Plots/fp160_rs112499751.png]

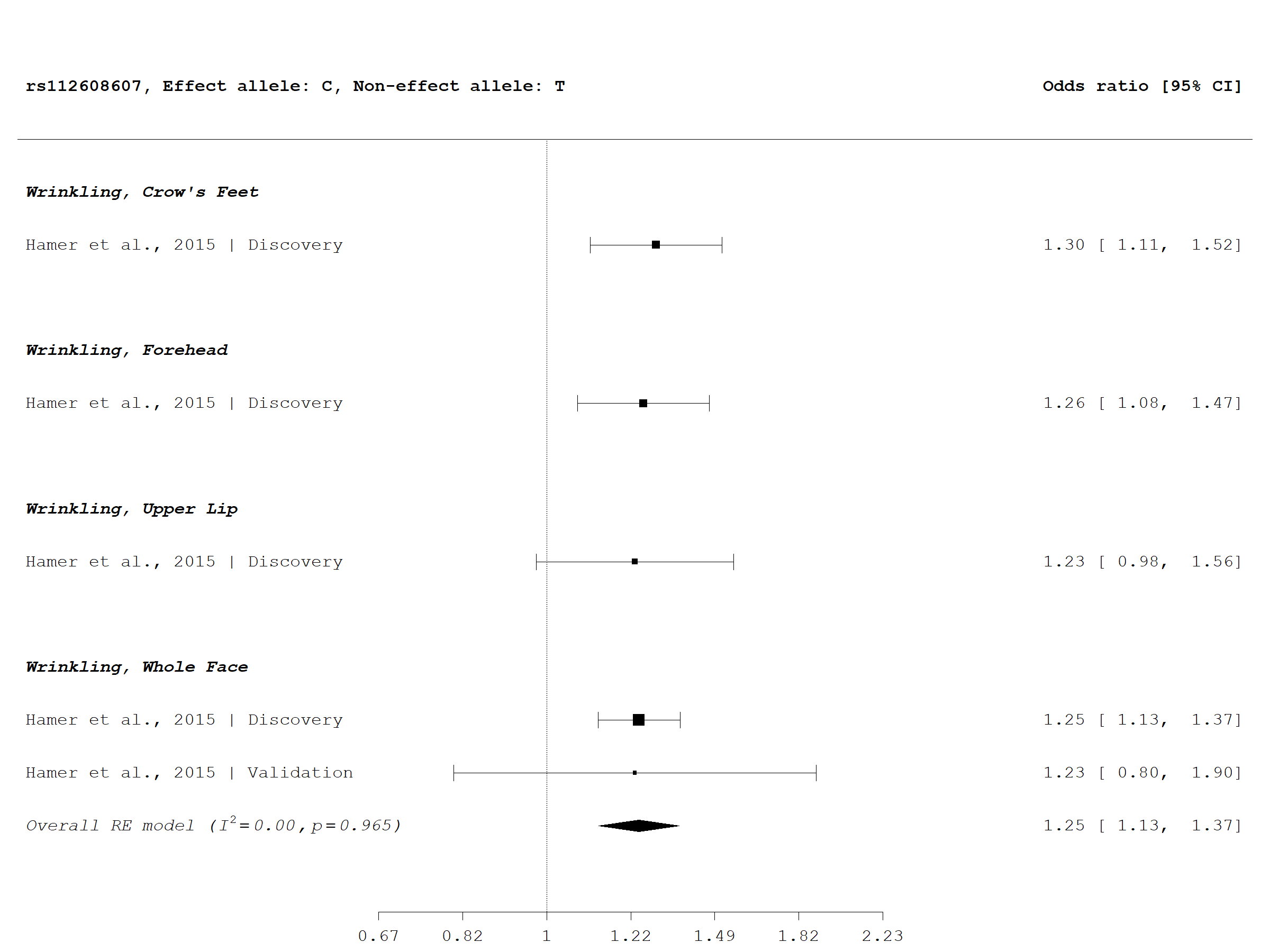

Supplement: Supplementary file 1 — Supplementary Information 1. [file 41598_2022_17443_MOESM1_ESM.zip › Supplementary Datasets/Dataset S3 - Forest Plots/fp161_rs112608607.png]

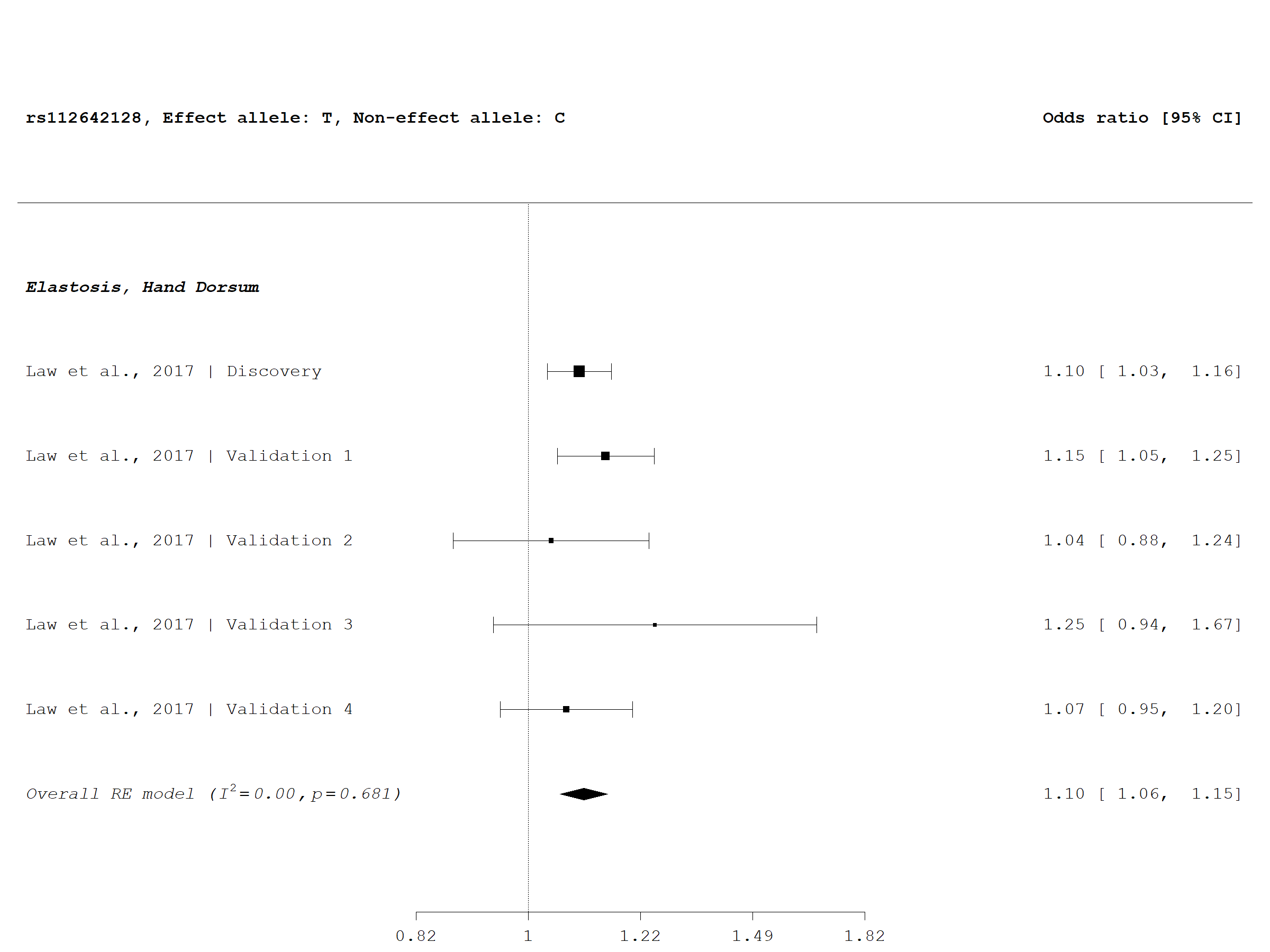

Supplement: Supplementary file 1 — Supplementary Information 1. [file 41598_2022_17443_MOESM1_ESM.zip › Supplementary Datasets/Dataset S3 - Forest Plots/fp162_rs112642128.png]

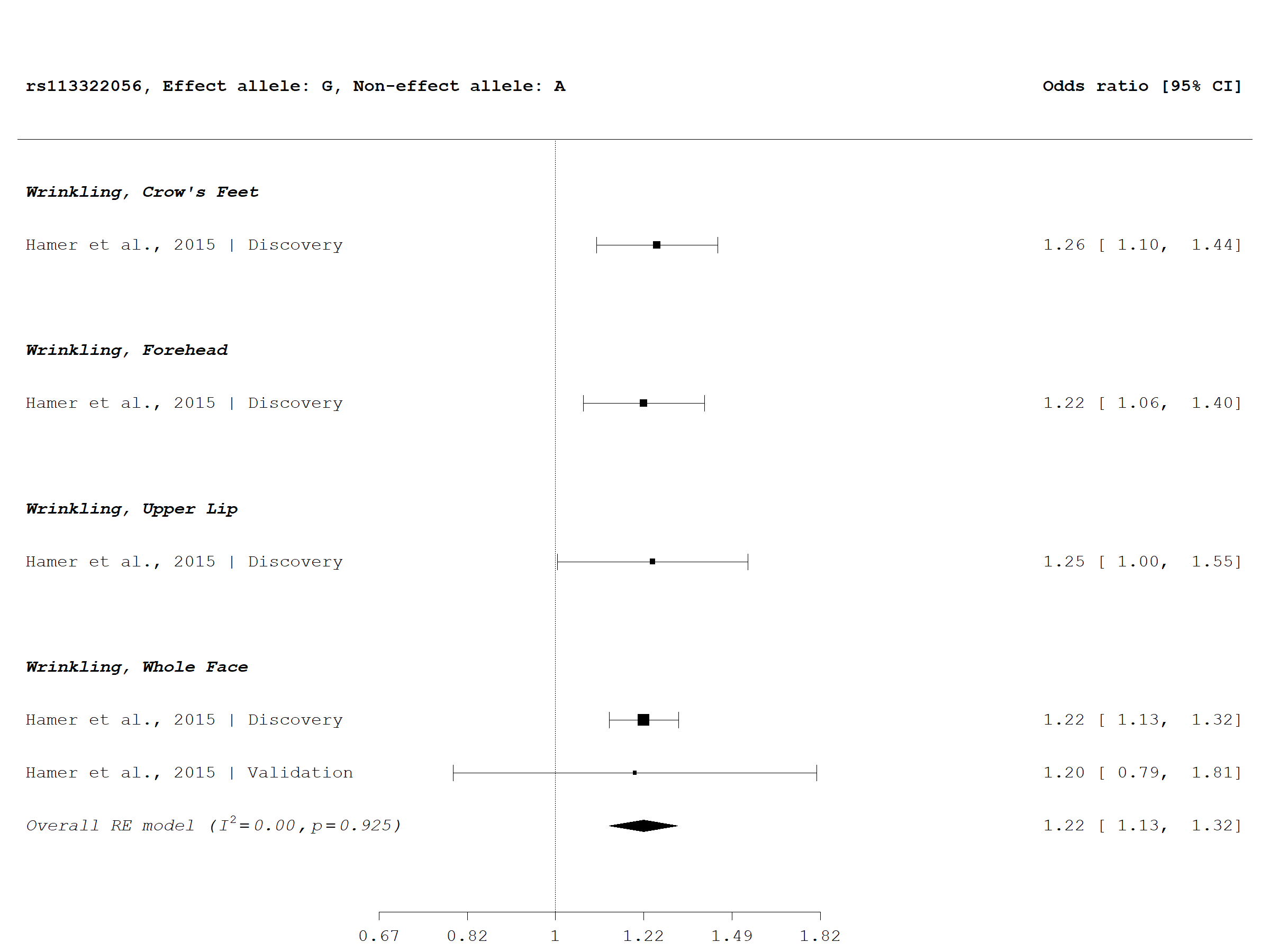

Supplement: Supplementary file 1 — Supplementary Information 1. [file 41598_2022_17443_MOESM1_ESM.zip › Supplementary Datasets/Dataset S3 - Forest Plots/fp163_rs113322056.png]

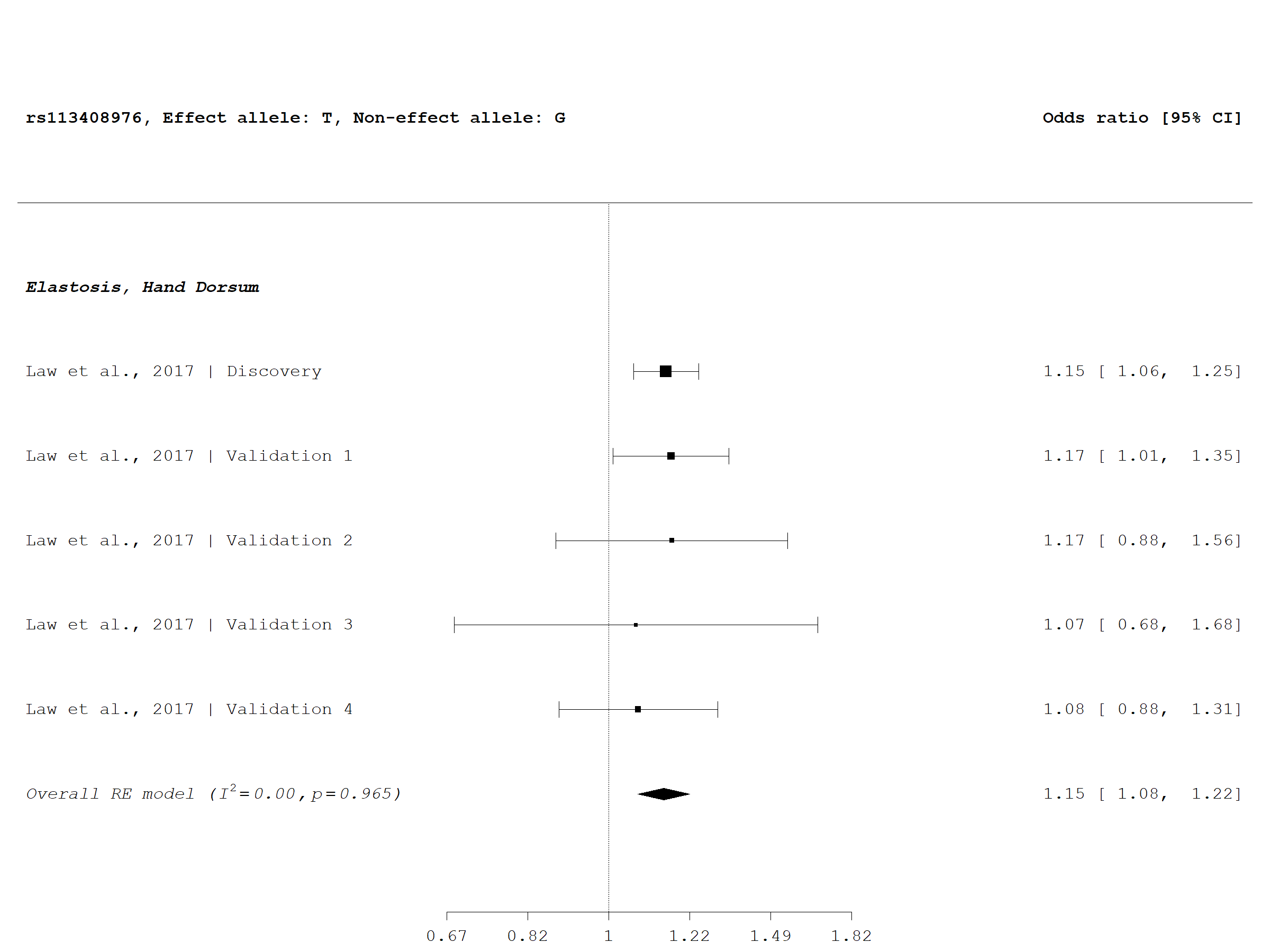

Supplement: Supplementary file 1 — Supplementary Information 1. [file 41598_2022_17443_MOESM1_ESM.zip › Supplementary Datasets/Dataset S3 - Forest Plots/fp164_rs113408976.png]

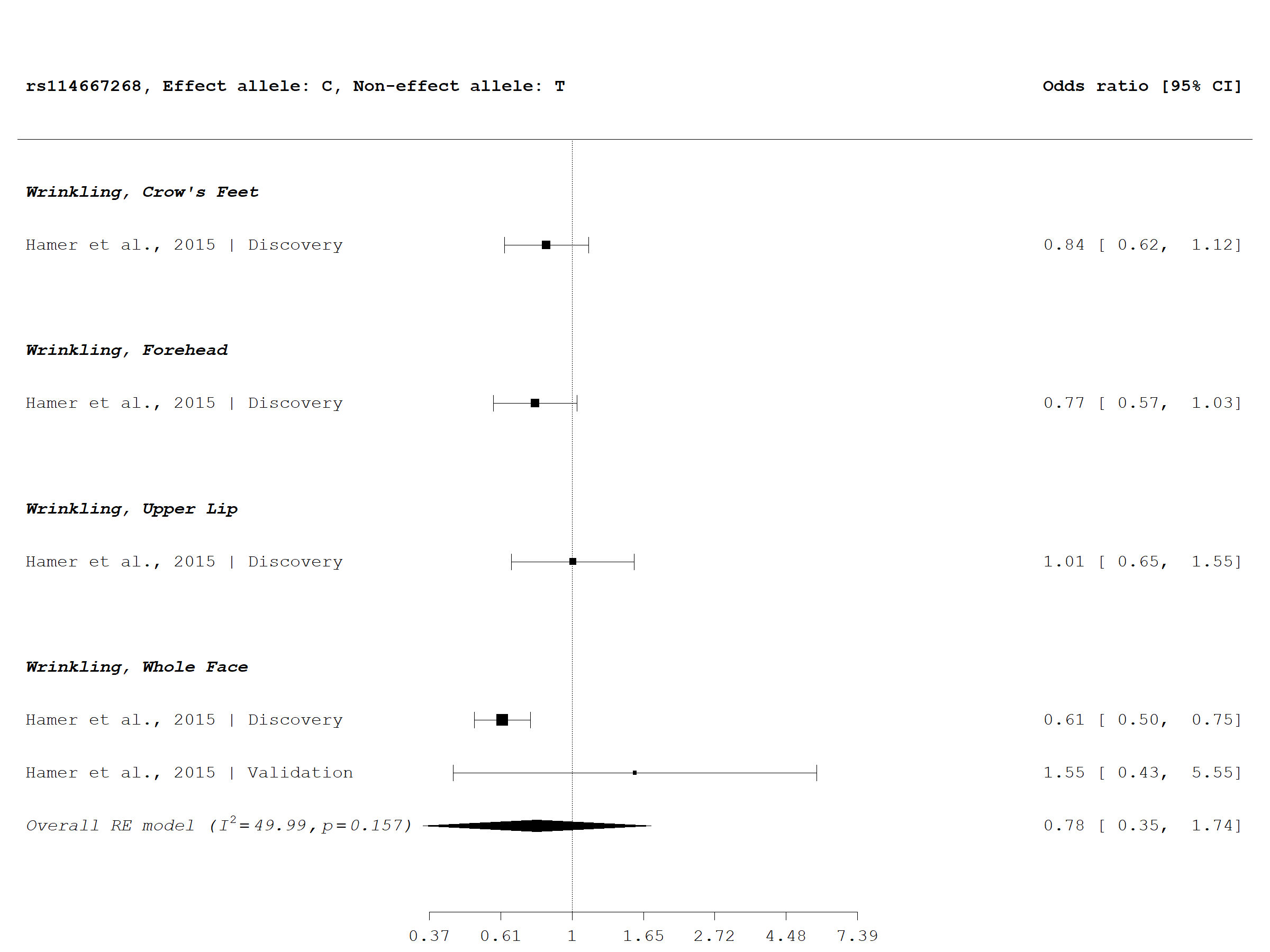

Supplement: Supplementary file 1 — Supplementary Information 1. [file 41598_2022_17443_MOESM1_ESM.zip › Supplementary Datasets/Dataset S3 - Forest Plots/fp165_rs114667268.png]

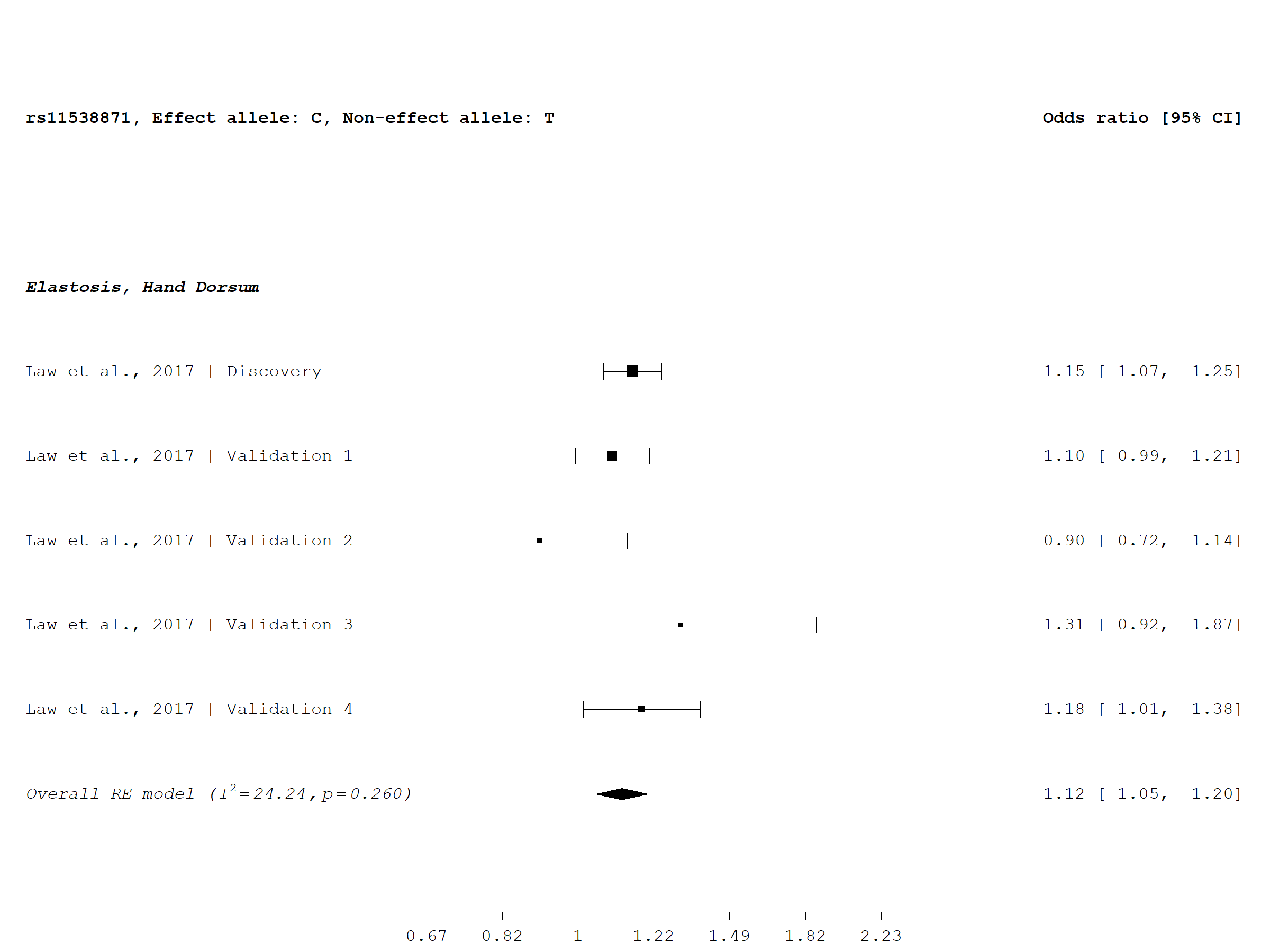

Supplement: Supplementary file 1 — Supplementary Information 1. [file 41598_2022_17443_MOESM1_ESM.zip › Supplementary Datasets/Dataset S3 - Forest Plots/fp166_rs11538871.png]

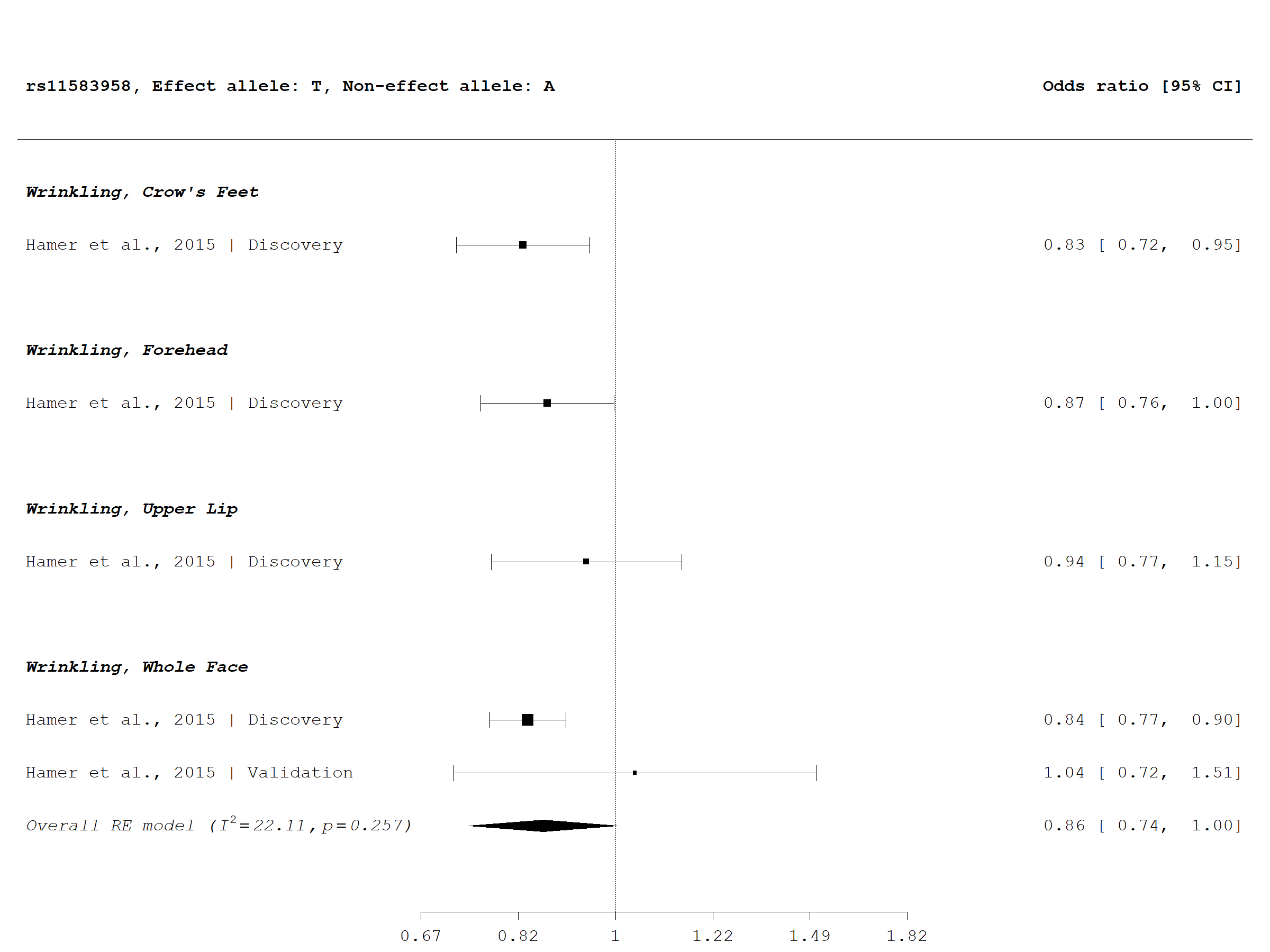

Supplement: Supplementary file 1 — Supplementary Information 1. [file 41598_2022_17443_MOESM1_ESM.zip › Supplementary Datasets/Dataset S3 - Forest Plots/fp167_rs11583958.png]

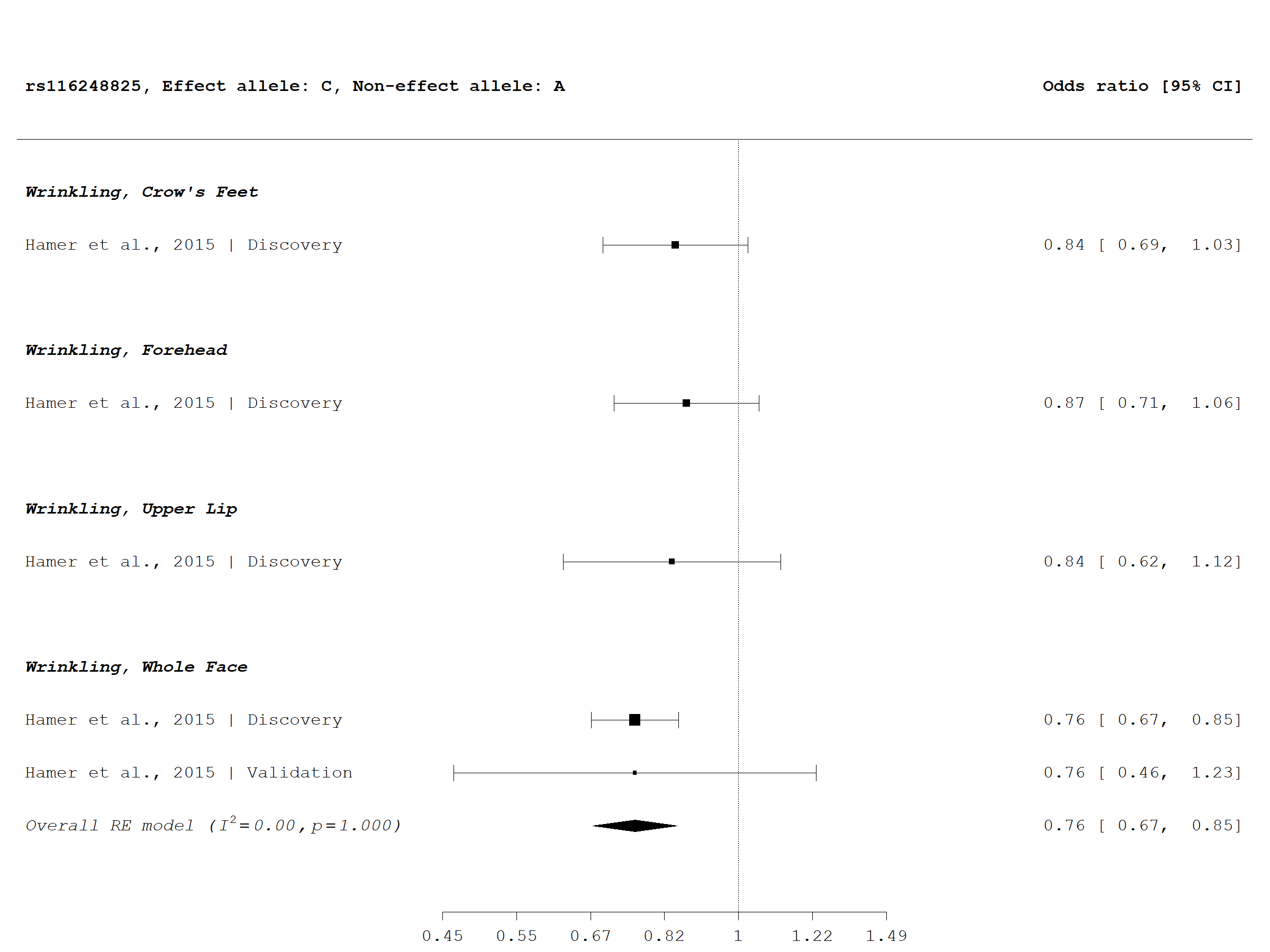

Supplement: Supplementary file 1 — Supplementary Information 1. [file 41598_2022_17443_MOESM1_ESM.zip › Supplementary Datasets/Dataset S3 - Forest Plots/fp168_rs116248825.png]

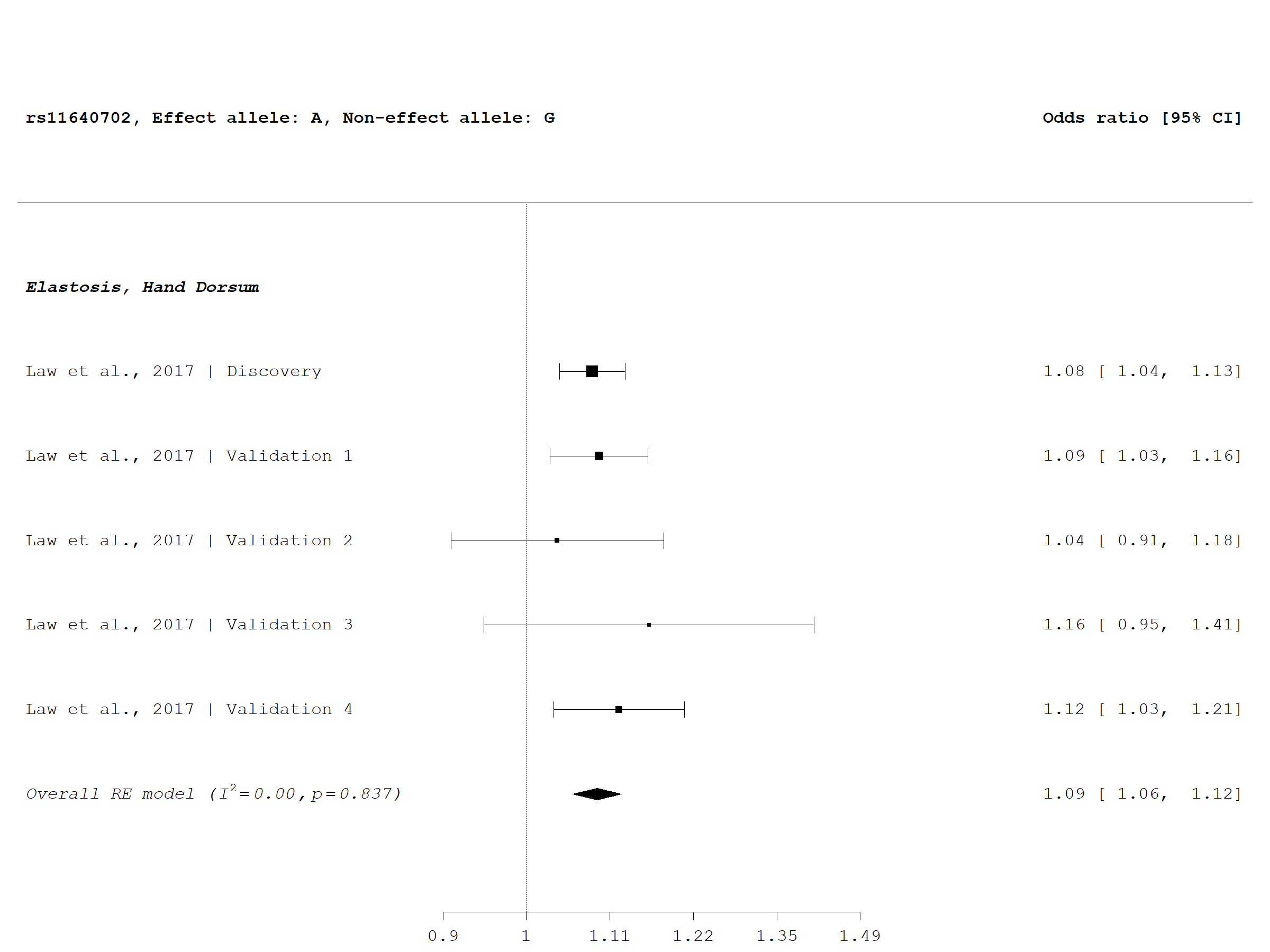

Supplement: Supplementary file 1 — Supplementary Information 1. [file 41598_2022_17443_MOESM1_ESM.zip › Supplementary Datasets/Dataset S3 - Forest Plots/fp169_rs11640702.png]

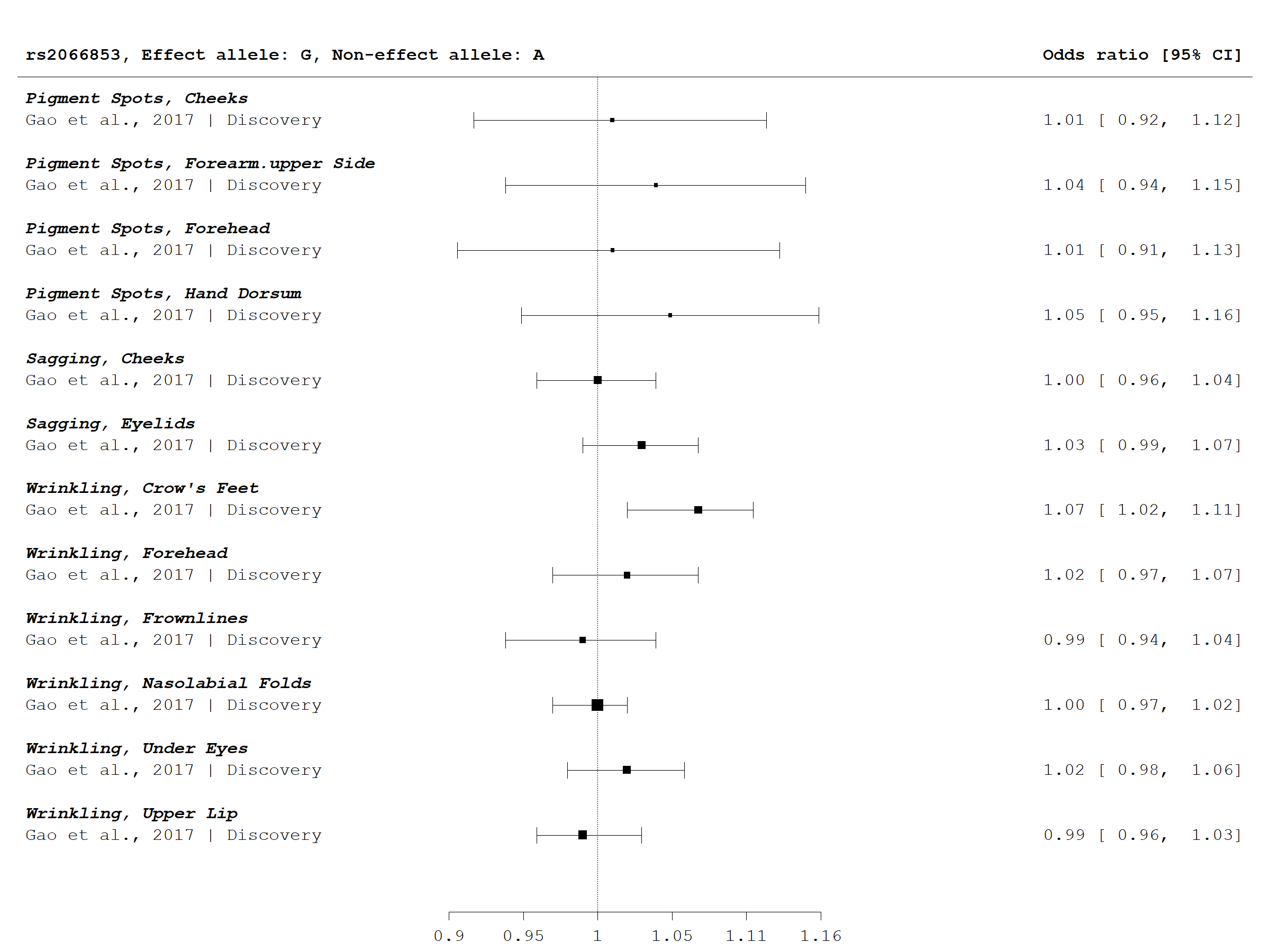

Supplement: Supplementary file 1 — Supplementary Information 1. [file 41598_2022_17443_MOESM1_ESM.zip › Supplementary Datasets/Dataset S3 - Forest Plots/fp16_rs2066853.png]

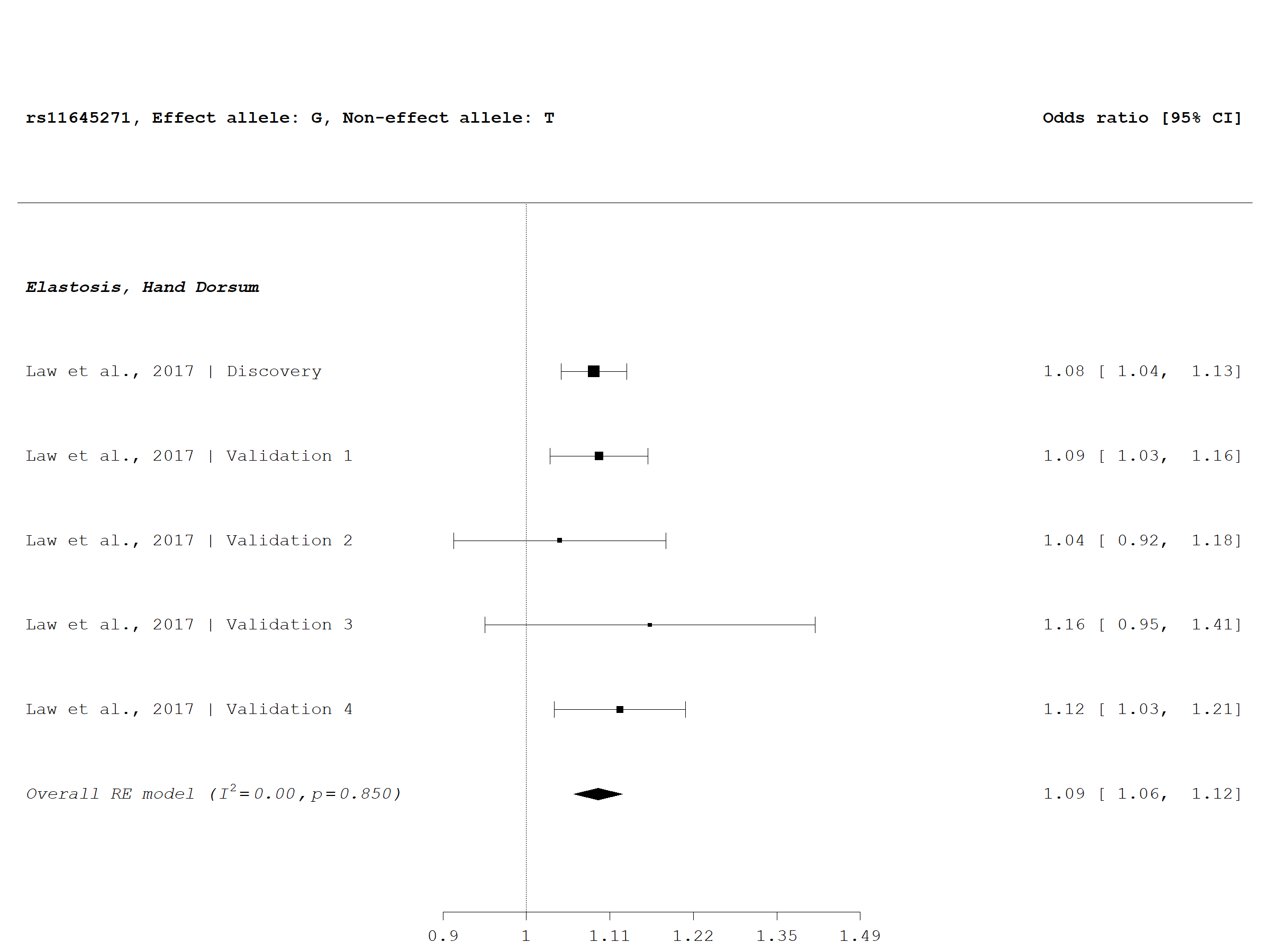

Supplement: Supplementary file 1 — Supplementary Information 1. [file 41598_2022_17443_MOESM1_ESM.zip › Supplementary Datasets/Dataset S3 - Forest Plots/fp170_rs11645271.png]

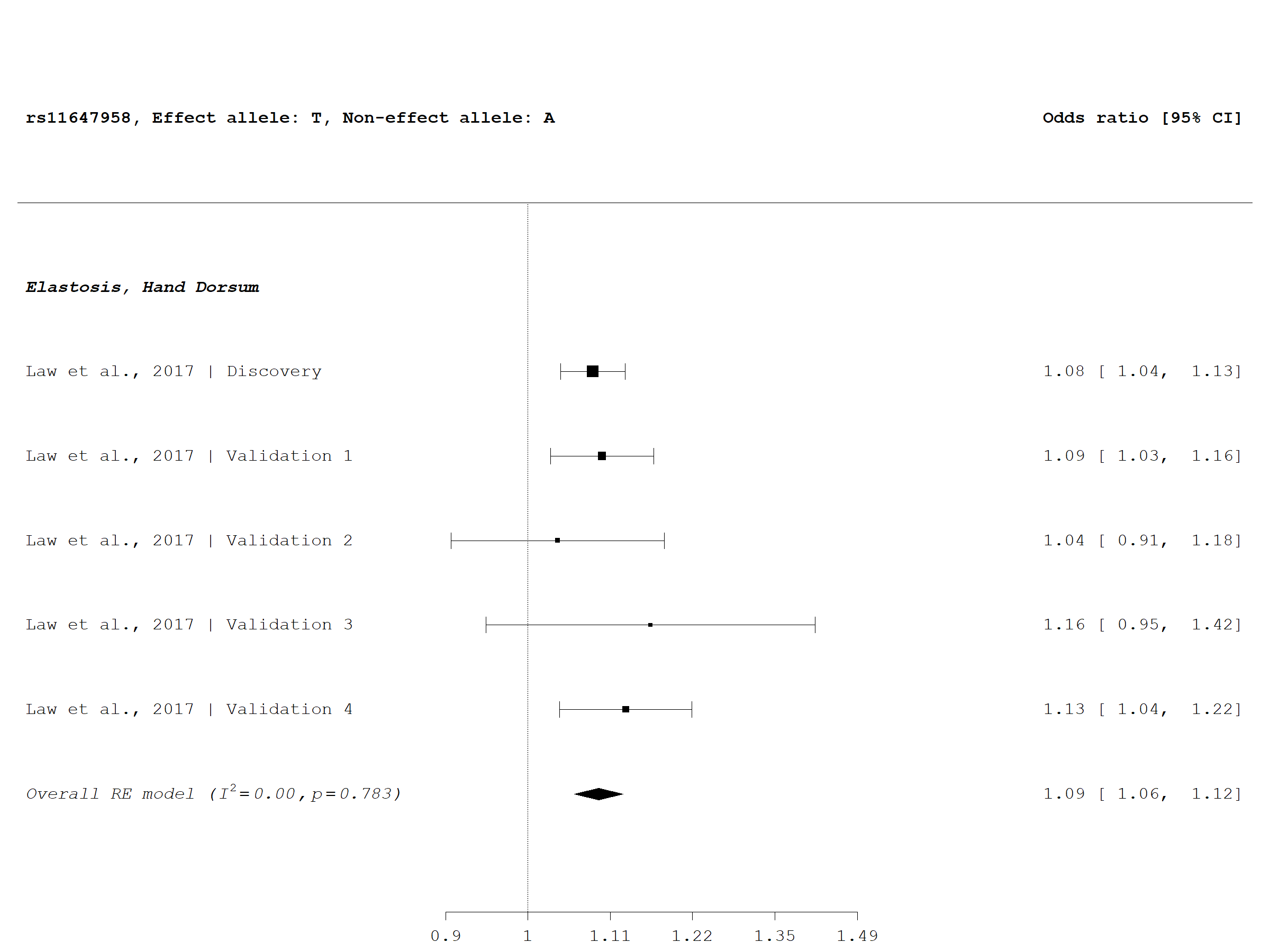

Supplement: Supplementary file 1 — Supplementary Information 1. [file 41598_2022_17443_MOESM1_ESM.zip › Supplementary Datasets/Dataset S3 - Forest Plots/fp171_rs11647958.png]

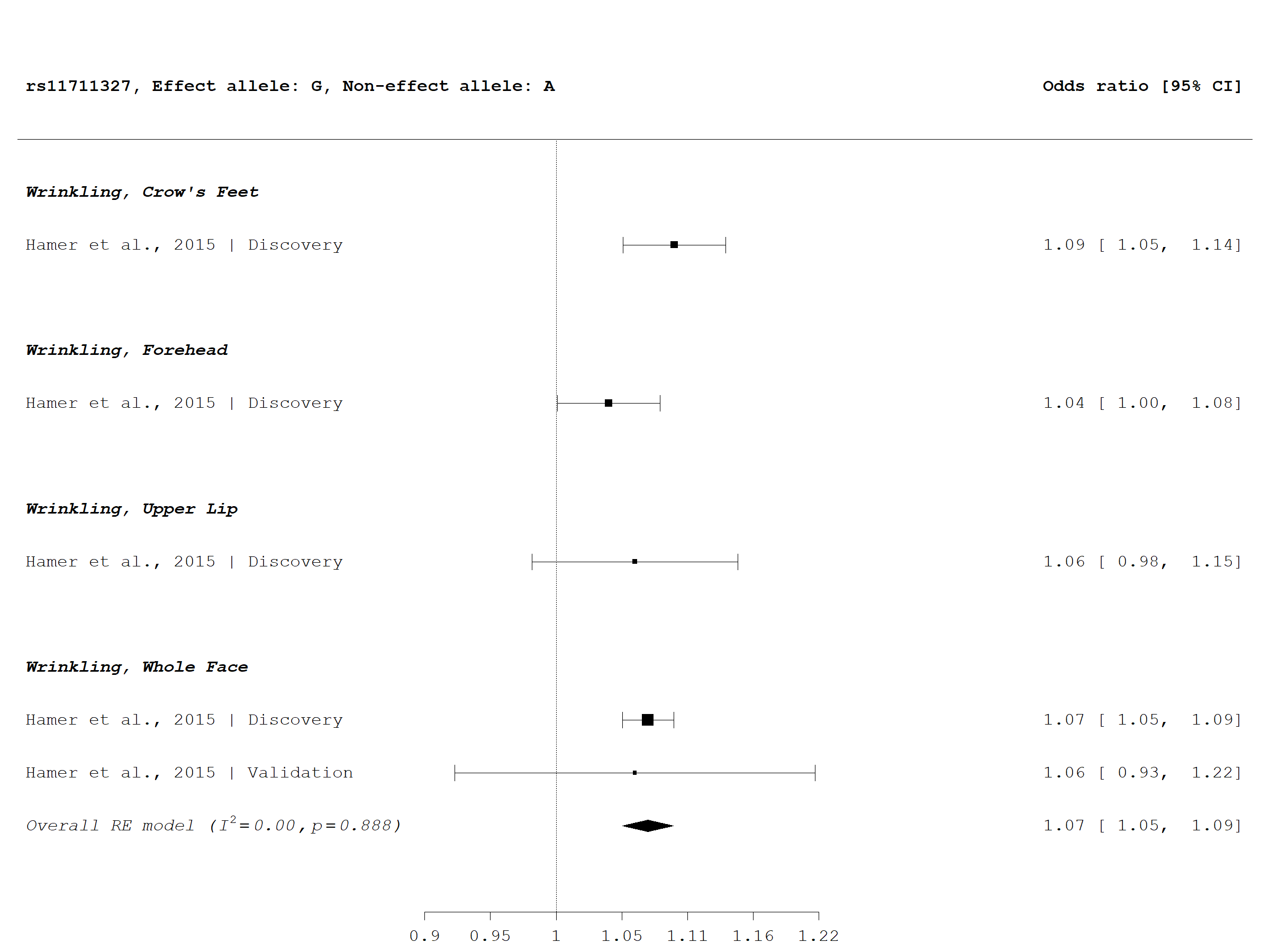

Supplement: Supplementary file 1 — Supplementary Information 1. [file 41598_2022_17443_MOESM1_ESM.zip › Supplementary Datasets/Dataset S3 - Forest Plots/fp172_rs11711327.png]

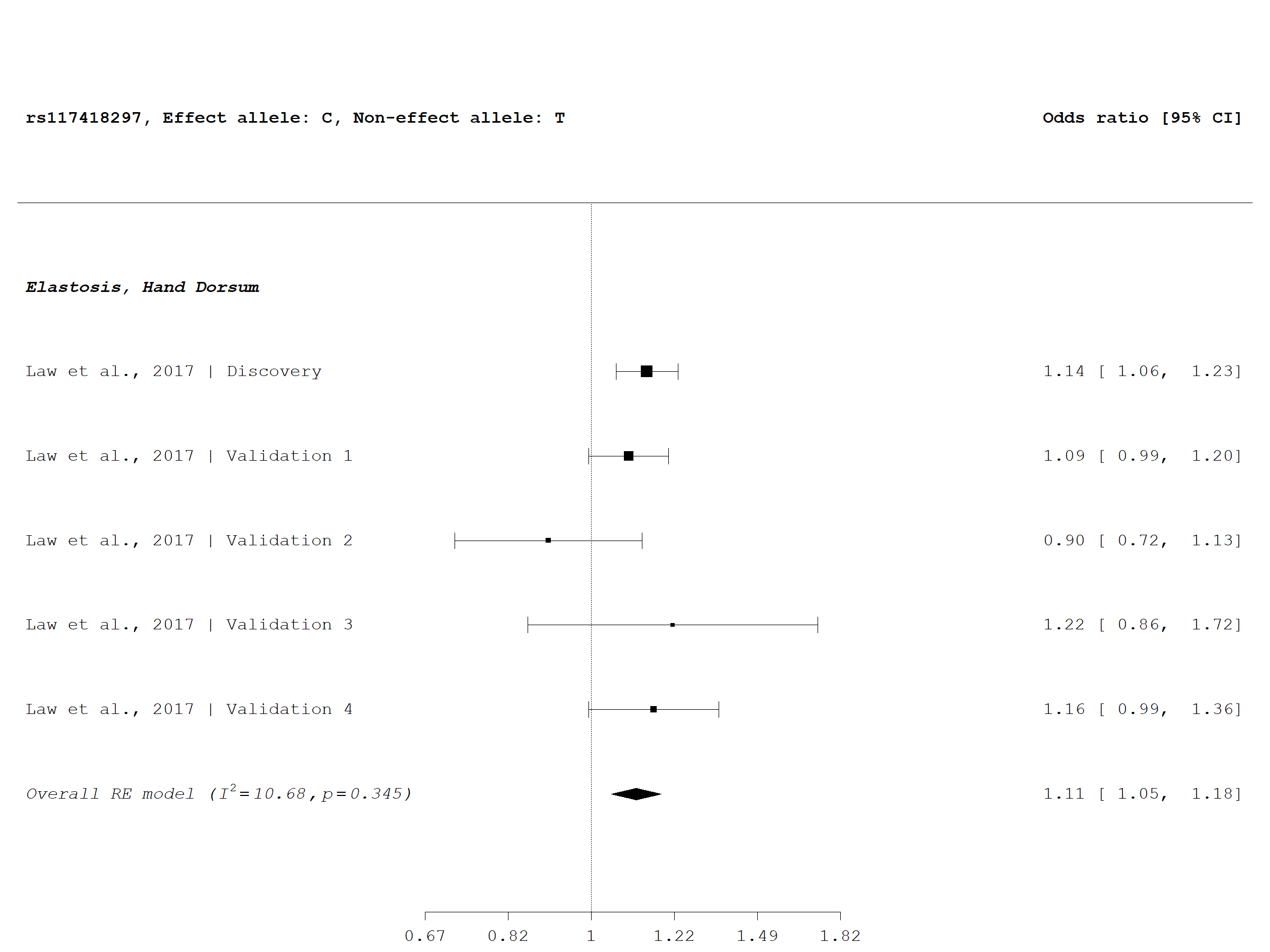

Supplement: Supplementary file 1 — Supplementary Information 1. [file 41598_2022_17443_MOESM1_ESM.zip › Supplementary Datasets/Dataset S3 - Forest Plots/fp173_rs117418297.png]

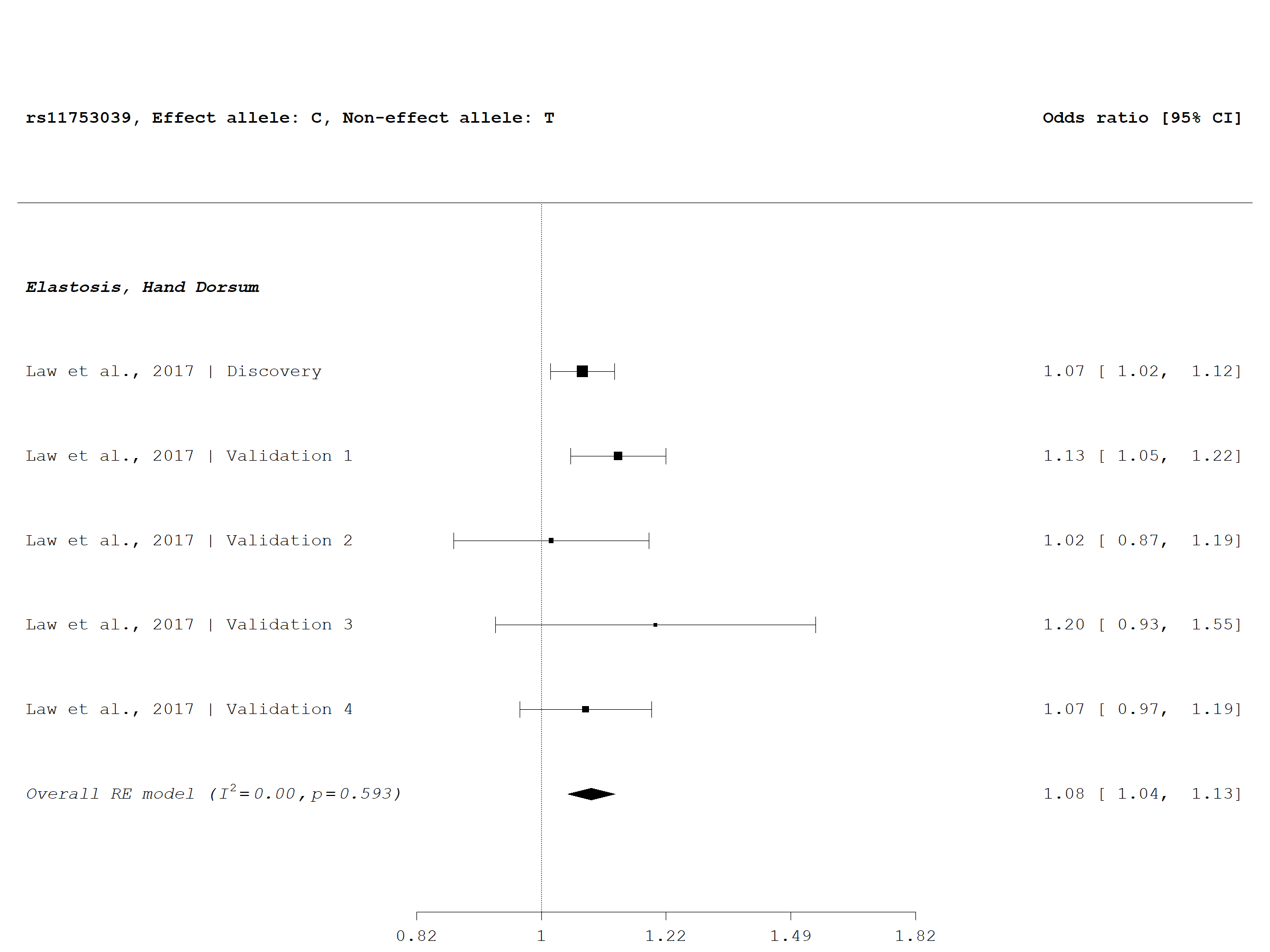

Supplement: Supplementary file 1 — Supplementary Information 1. [file 41598_2022_17443_MOESM1_ESM.zip › Supplementary Datasets/Dataset S3 - Forest Plots/fp174_rs11753039.png]

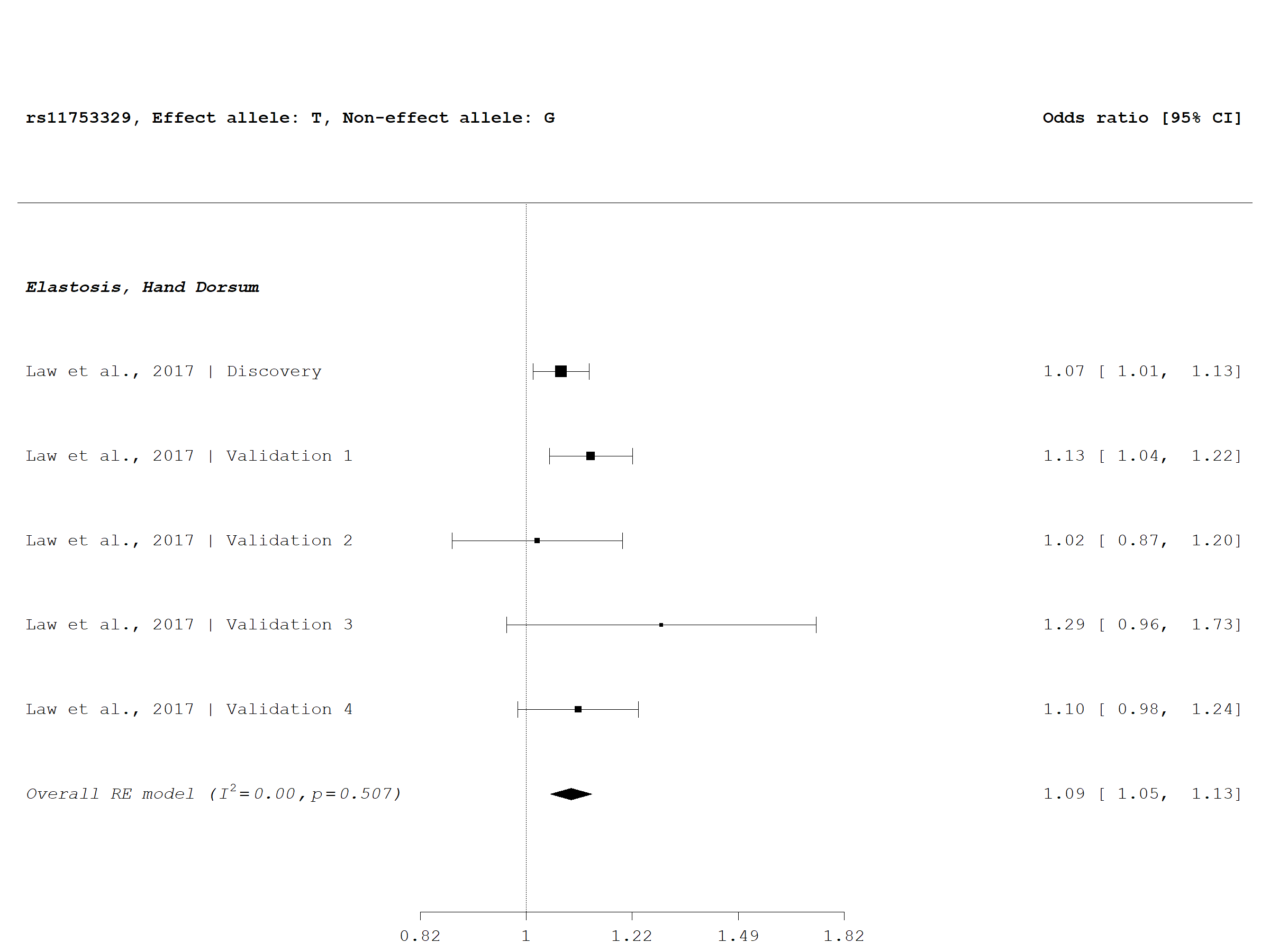

Supplement: Supplementary file 1 — Supplementary Information 1. [file 41598_2022_17443_MOESM1_ESM.zip › Supplementary Datasets/Dataset S3 - Forest Plots/fp175_rs11753329.png]

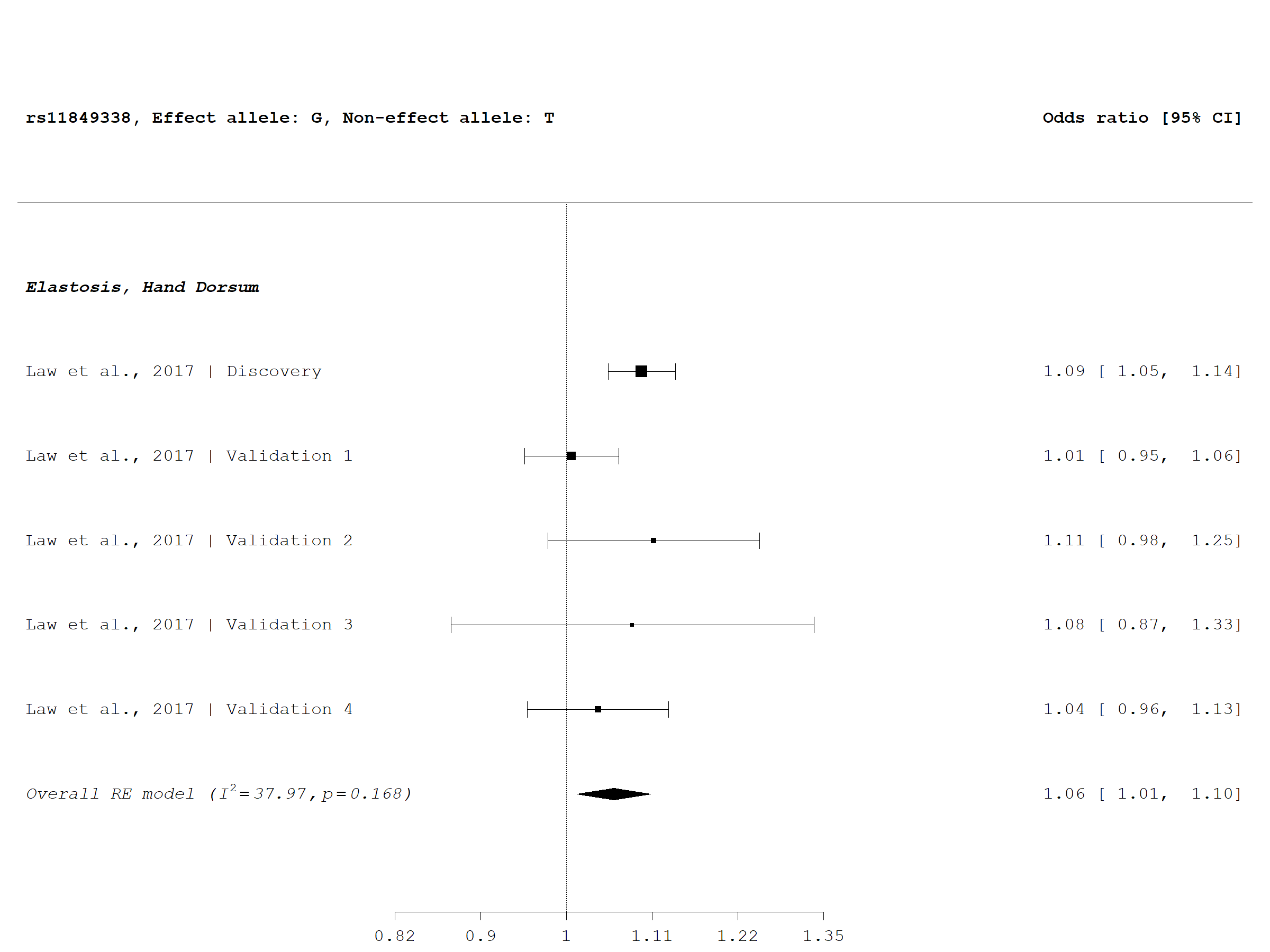

Supplement: Supplementary file 1 — Supplementary Information 1. [file 41598_2022_17443_MOESM1_ESM.zip › Supplementary Datasets/Dataset S3 - Forest Plots/fp176_rs11849338.png]

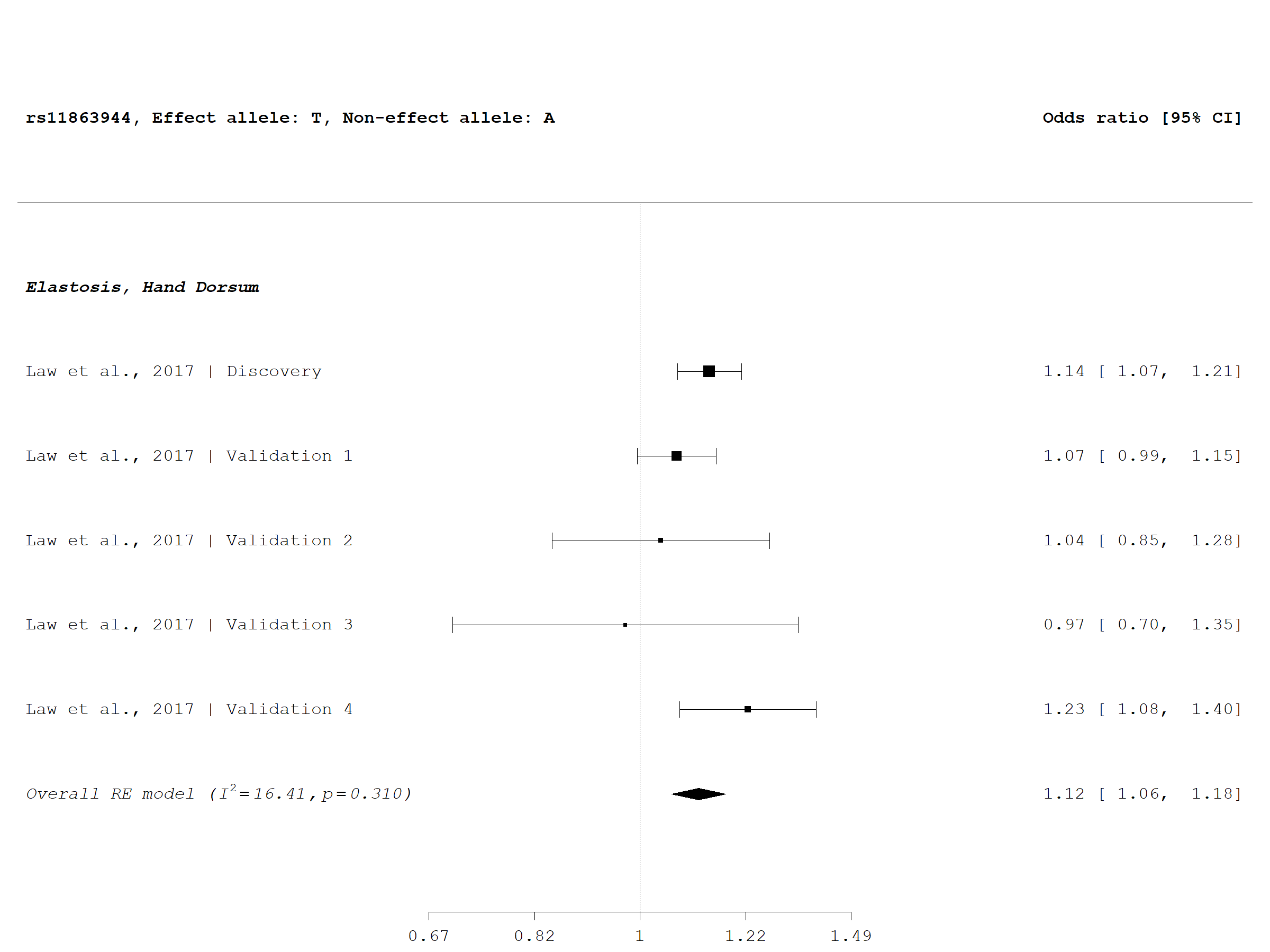

Supplement: Supplementary file 1 — Supplementary Information 1. [file 41598_2022_17443_MOESM1_ESM.zip › Supplementary Datasets/Dataset S3 - Forest Plots/fp177_rs11863944.png]

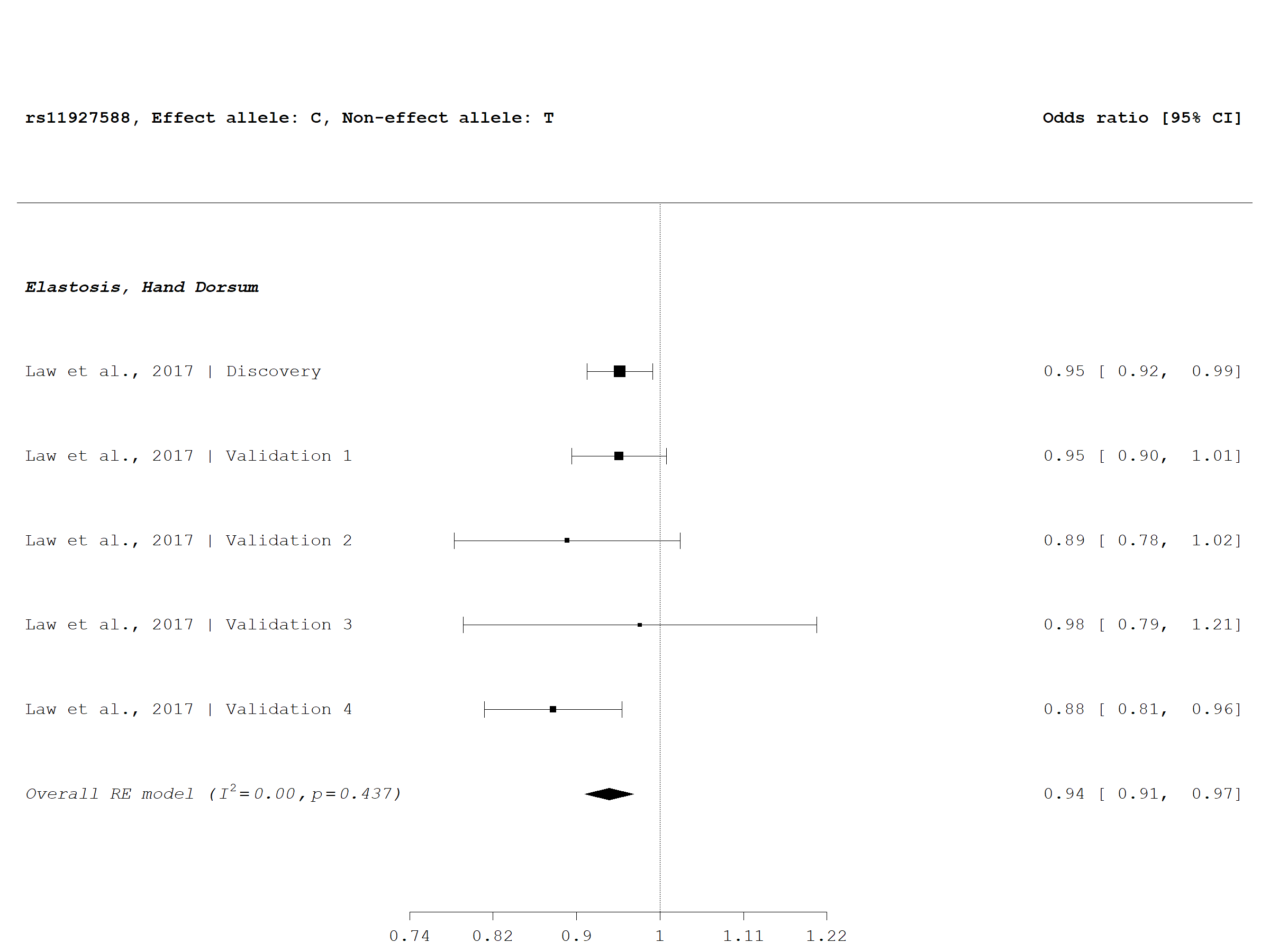

Supplement: Supplementary file 1 — Supplementary Information 1. [file 41598_2022_17443_MOESM1_ESM.zip › Supplementary Datasets/Dataset S3 - Forest Plots/fp178_rs11927588.png]

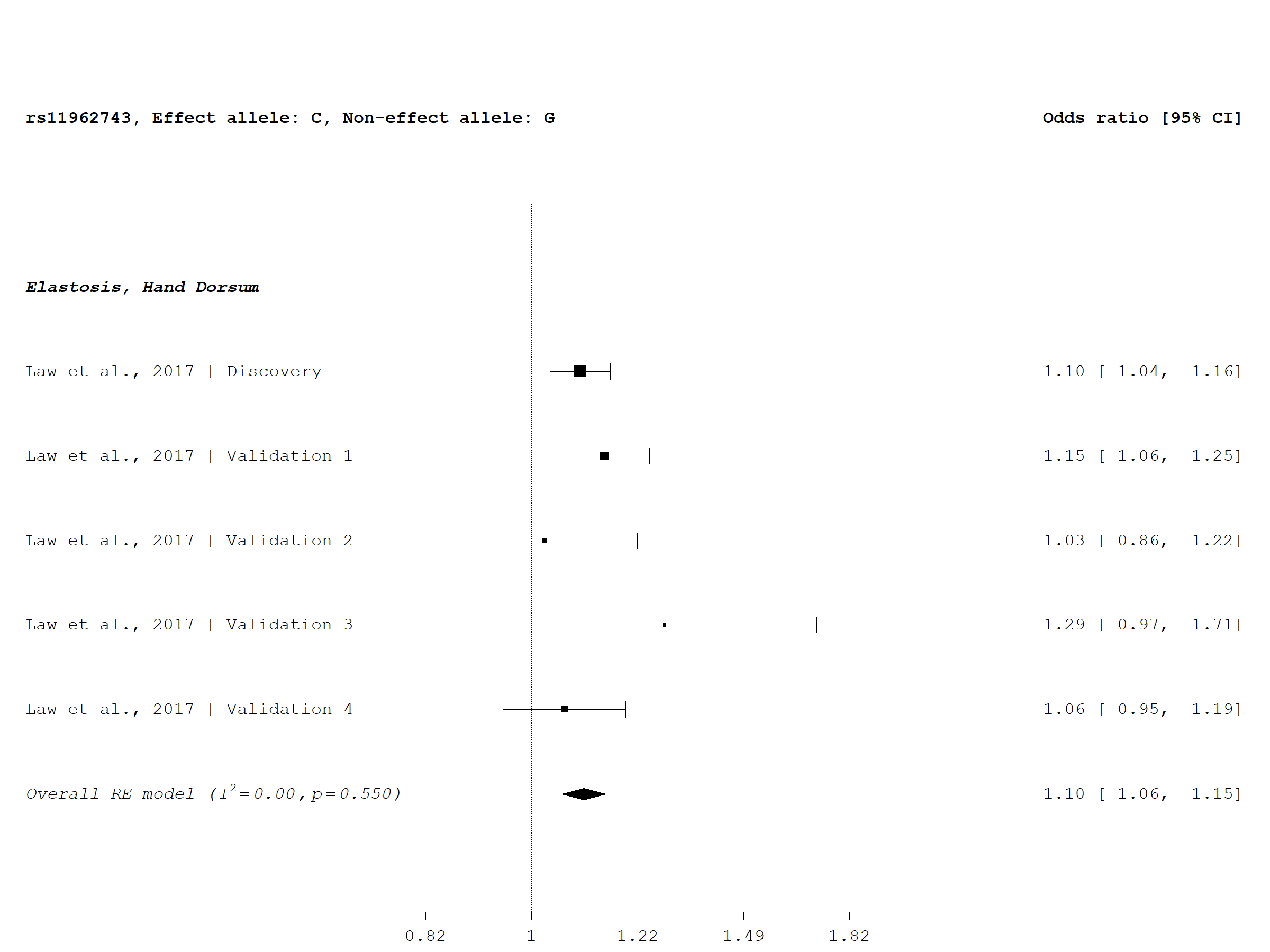

Supplement: Supplementary file 1 — Supplementary Information 1. [file 41598_2022_17443_MOESM1_ESM.zip › Supplementary Datasets/Dataset S3 - Forest Plots/fp179_rs11962743.png]

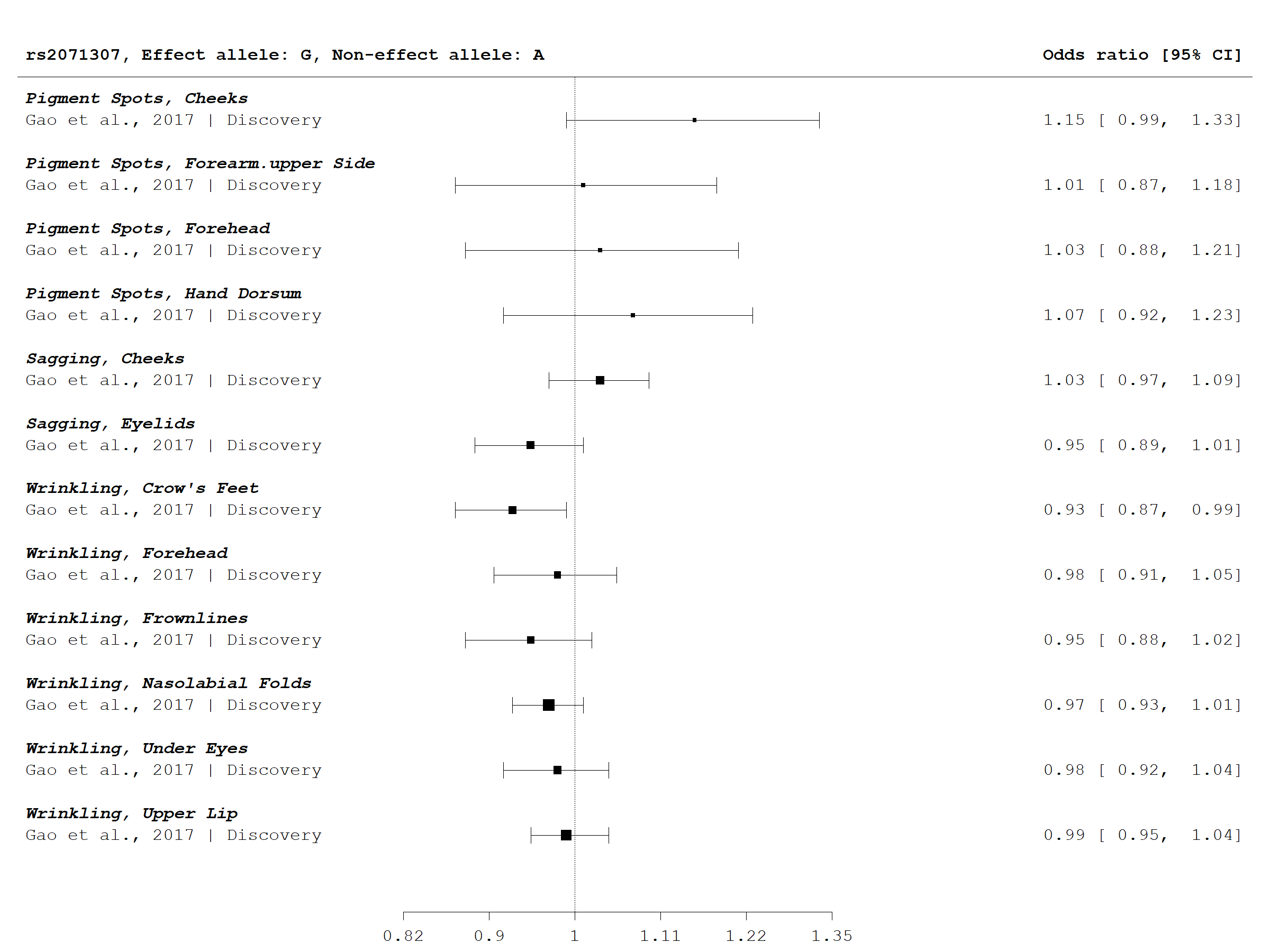

Supplement: Supplementary file 1 — Supplementary Information 1. [file 41598_2022_17443_MOESM1_ESM.zip › Supplementary Datasets/Dataset S3 - Forest Plots/fp17_rs2071307.png]

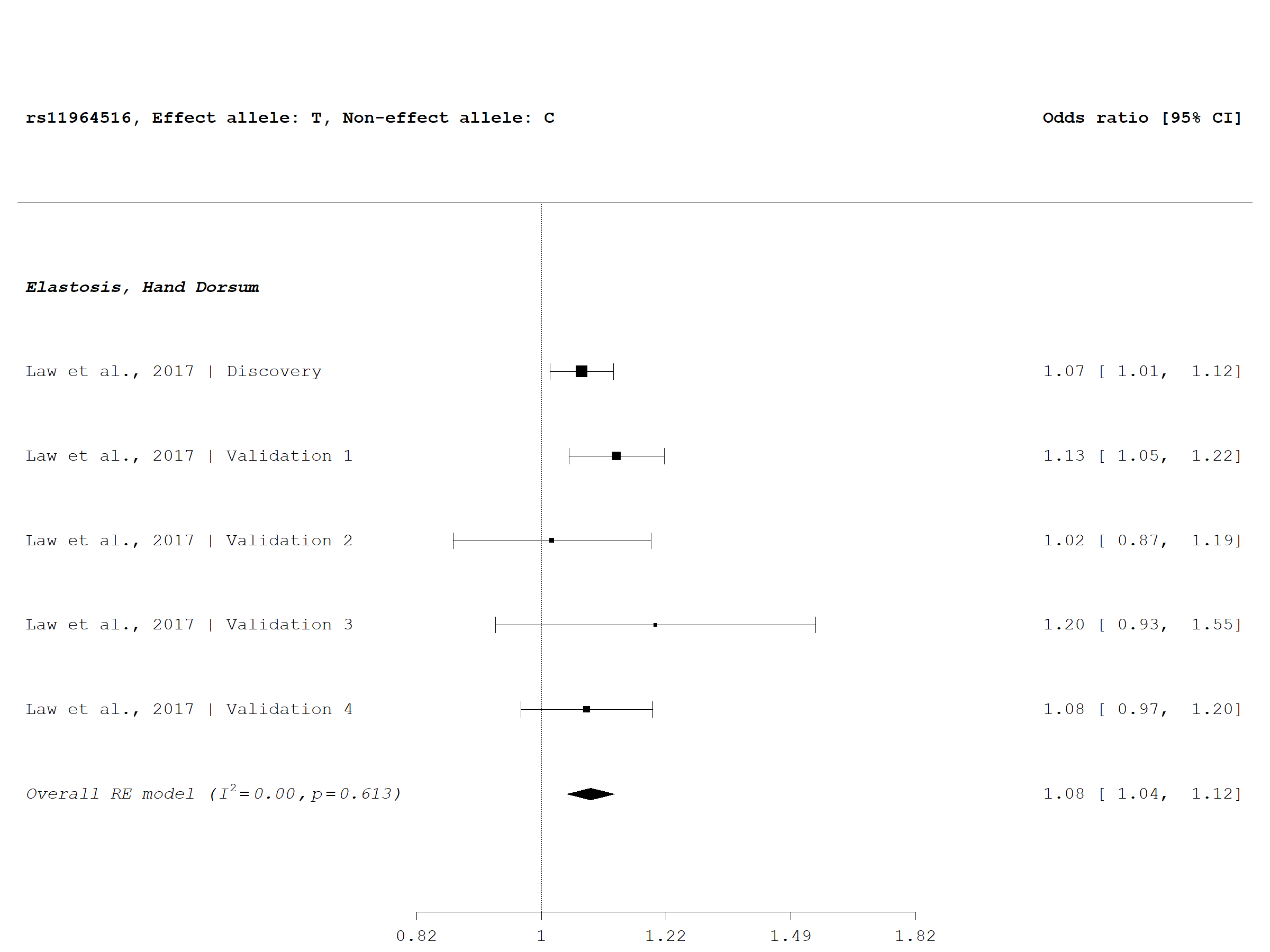

Supplement: Supplementary file 1 — Supplementary Information 1. [file 41598_2022_17443_MOESM1_ESM.zip › Supplementary Datasets/Dataset S3 - Forest Plots/fp180_rs11964516.png]

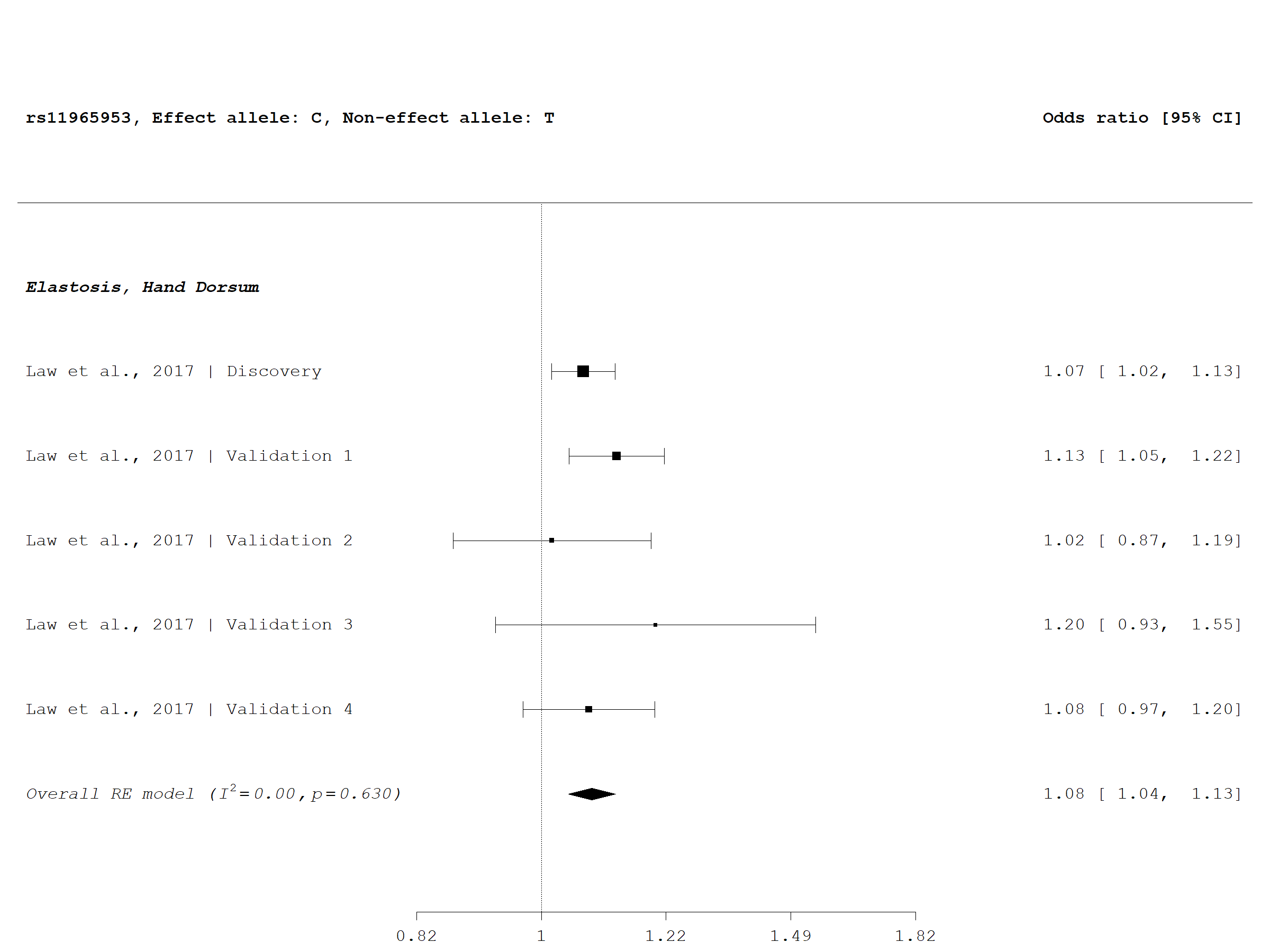

Supplement: Supplementary file 1 — Supplementary Information 1. [file 41598_2022_17443_MOESM1_ESM.zip › Supplementary Datasets/Dataset S3 - Forest Plots/fp181_rs11965953.png]

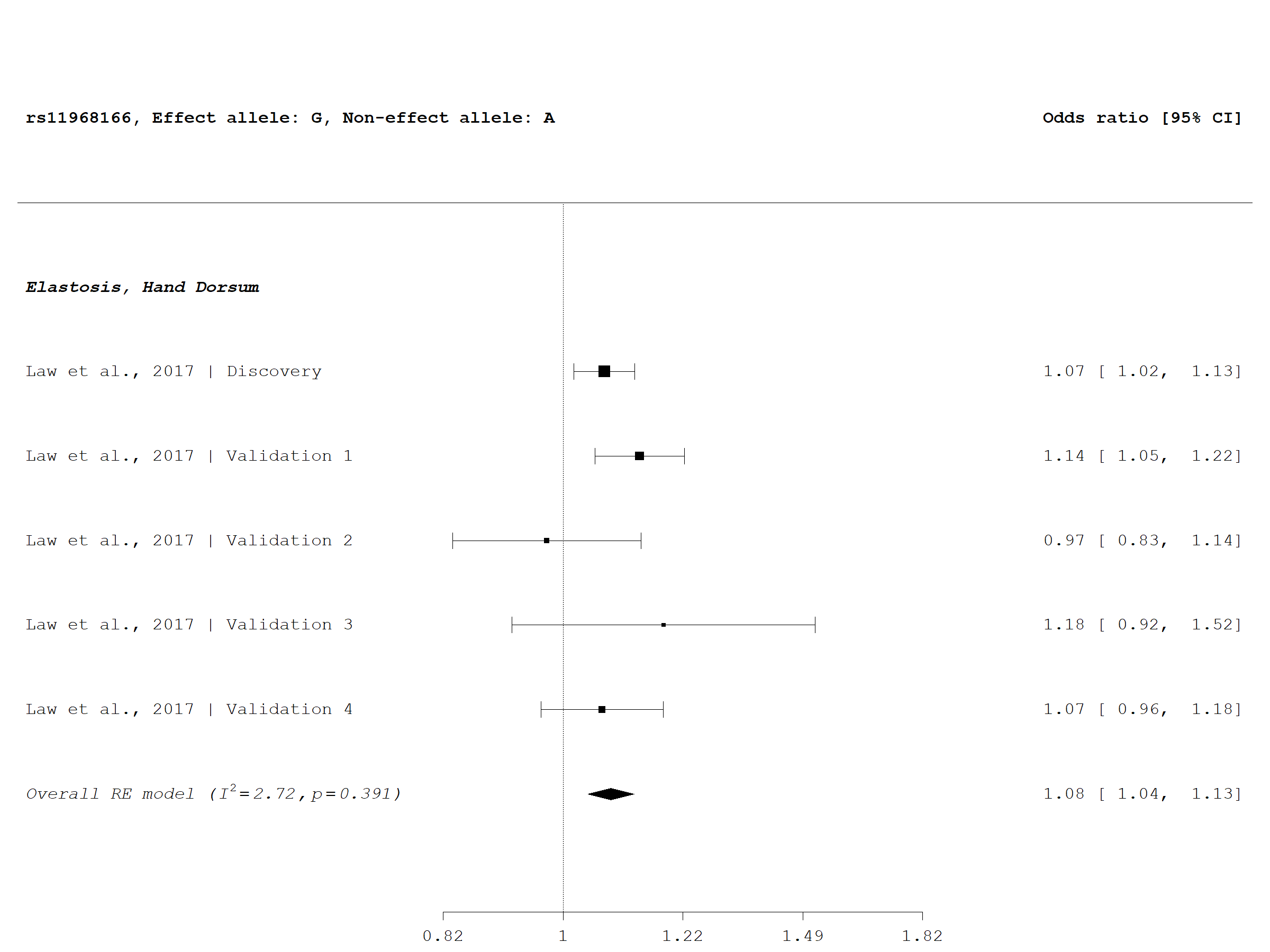

Supplement: Supplementary file 1 — Supplementary Information 1. [file 41598_2022_17443_MOESM1_ESM.zip › Supplementary Datasets/Dataset S3 - Forest Plots/fp182_rs11968166.png]

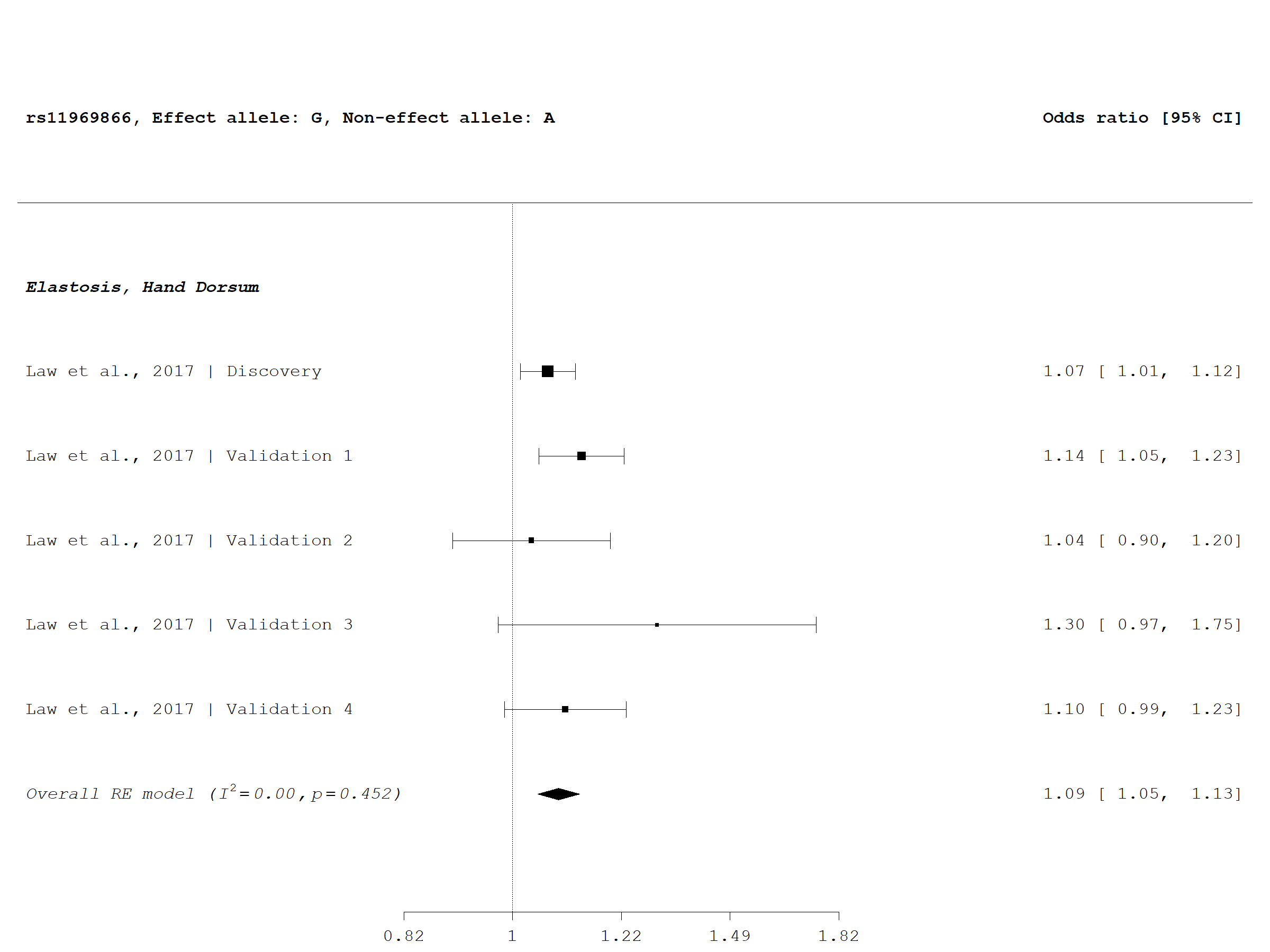

Supplement: Supplementary file 1 — Supplementary Information 1. [file 41598_2022_17443_MOESM1_ESM.zip › Supplementary Datasets/Dataset S3 - Forest Plots/fp183_rs11969866.png]

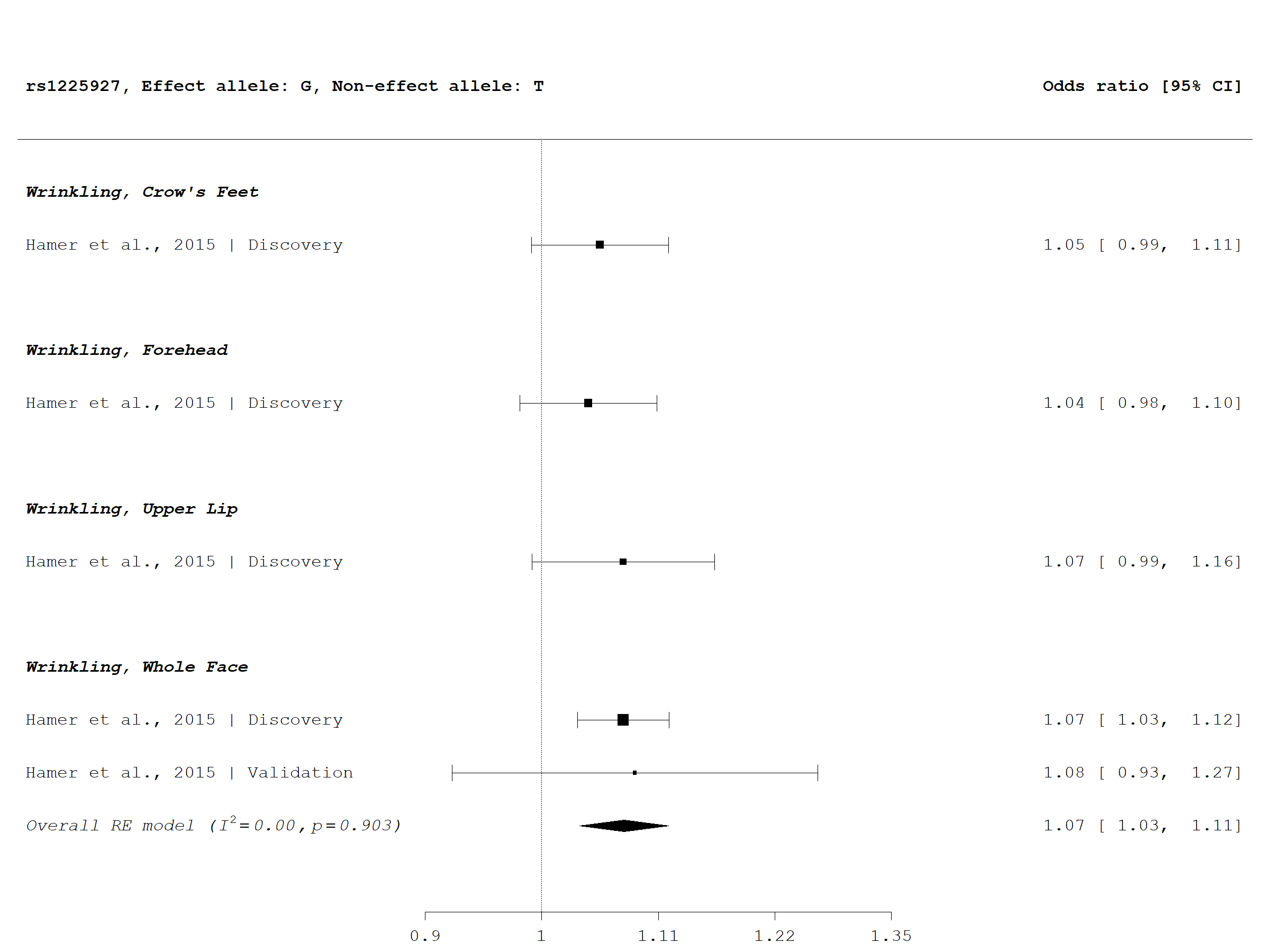

Supplement: Supplementary file 1 — Supplementary Information 1. [file 41598_2022_17443_MOESM1_ESM.zip › Supplementary Datasets/Dataset S3 - Forest Plots/fp184_rs1225927.png]

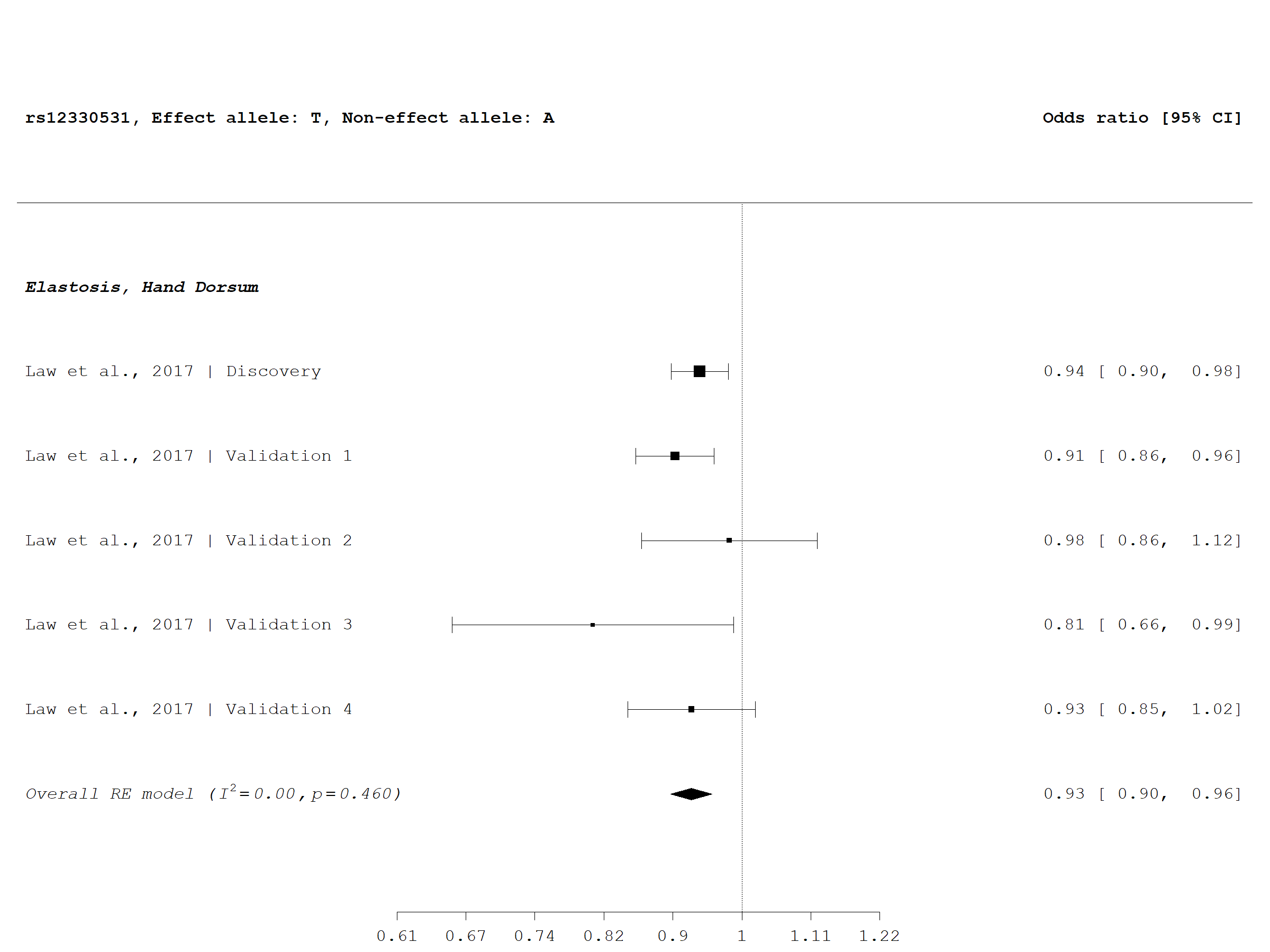

Supplement: Supplementary file 1 — Supplementary Information 1. [file 41598_2022_17443_MOESM1_ESM.zip › Supplementary Datasets/Dataset S3 - Forest Plots/fp185_rs12330531.png]

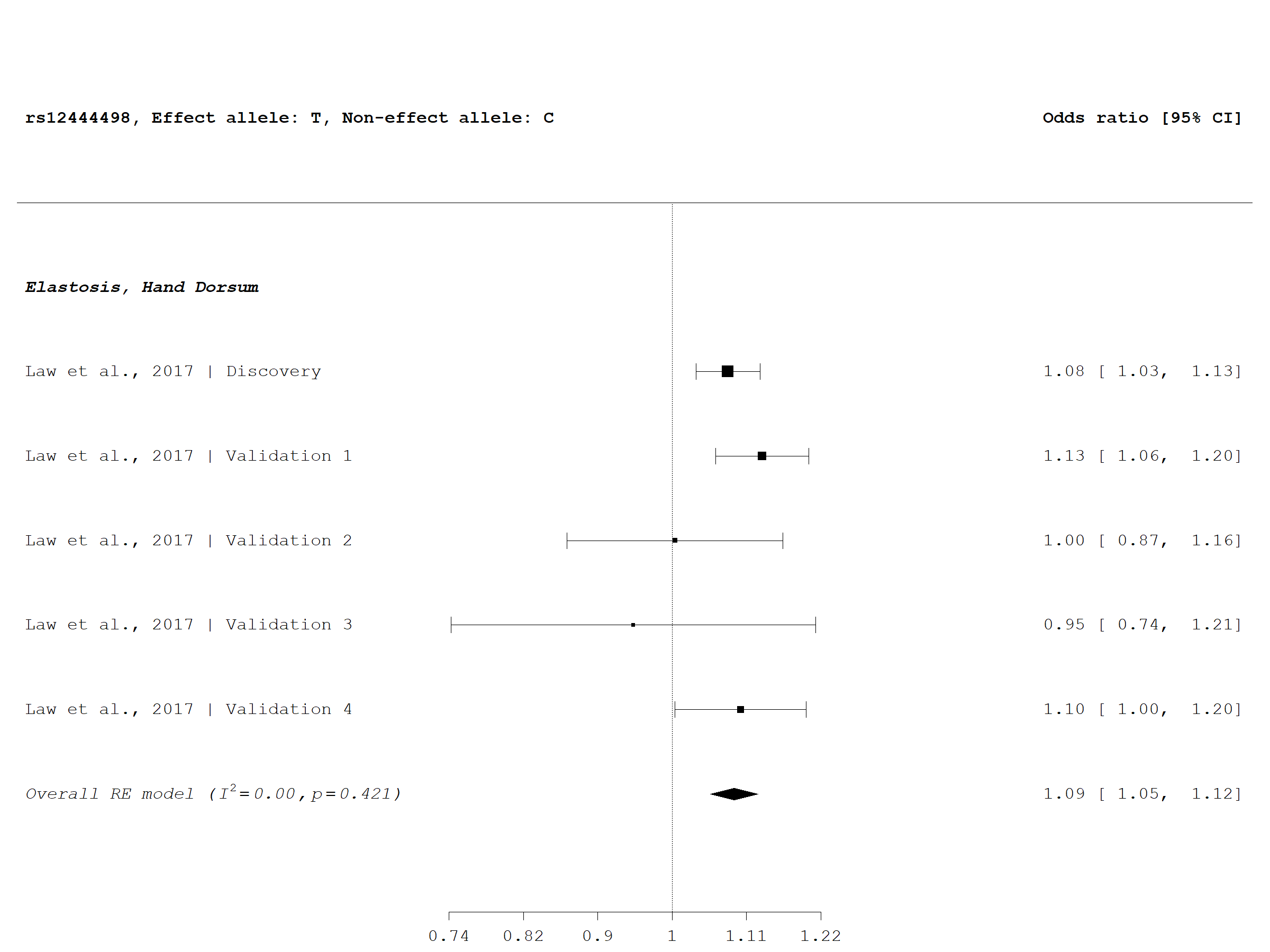

Supplement: Supplementary file 1 — Supplementary Information 1. [file 41598_2022_17443_MOESM1_ESM.zip › Supplementary Datasets/Dataset S3 - Forest Plots/fp186_rs12444498.png]

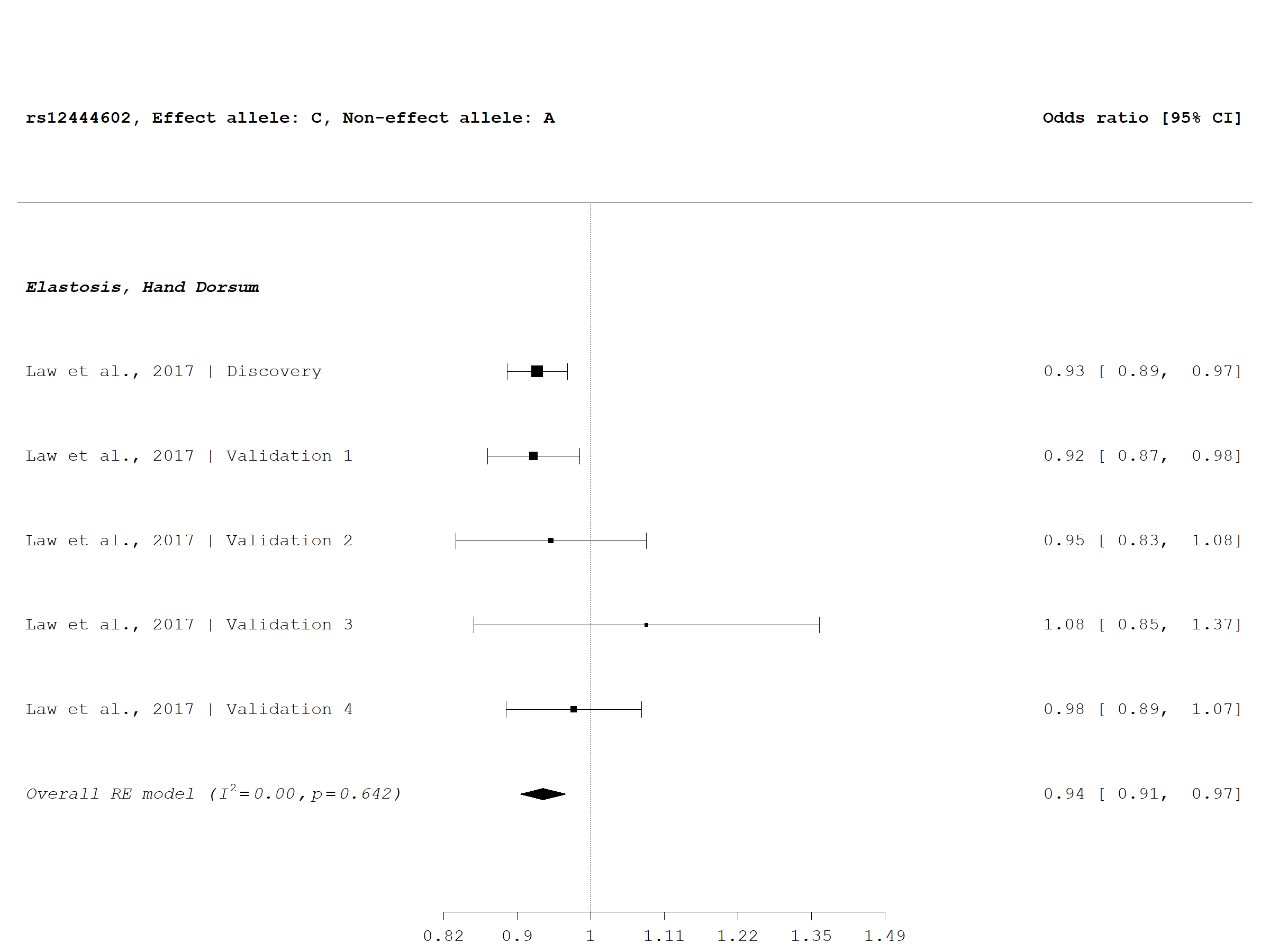

Supplement: Supplementary file 1 — Supplementary Information 1. [file 41598_2022_17443_MOESM1_ESM.zip › Supplementary Datasets/Dataset S3 - Forest Plots/fp187_rs12444602.png]

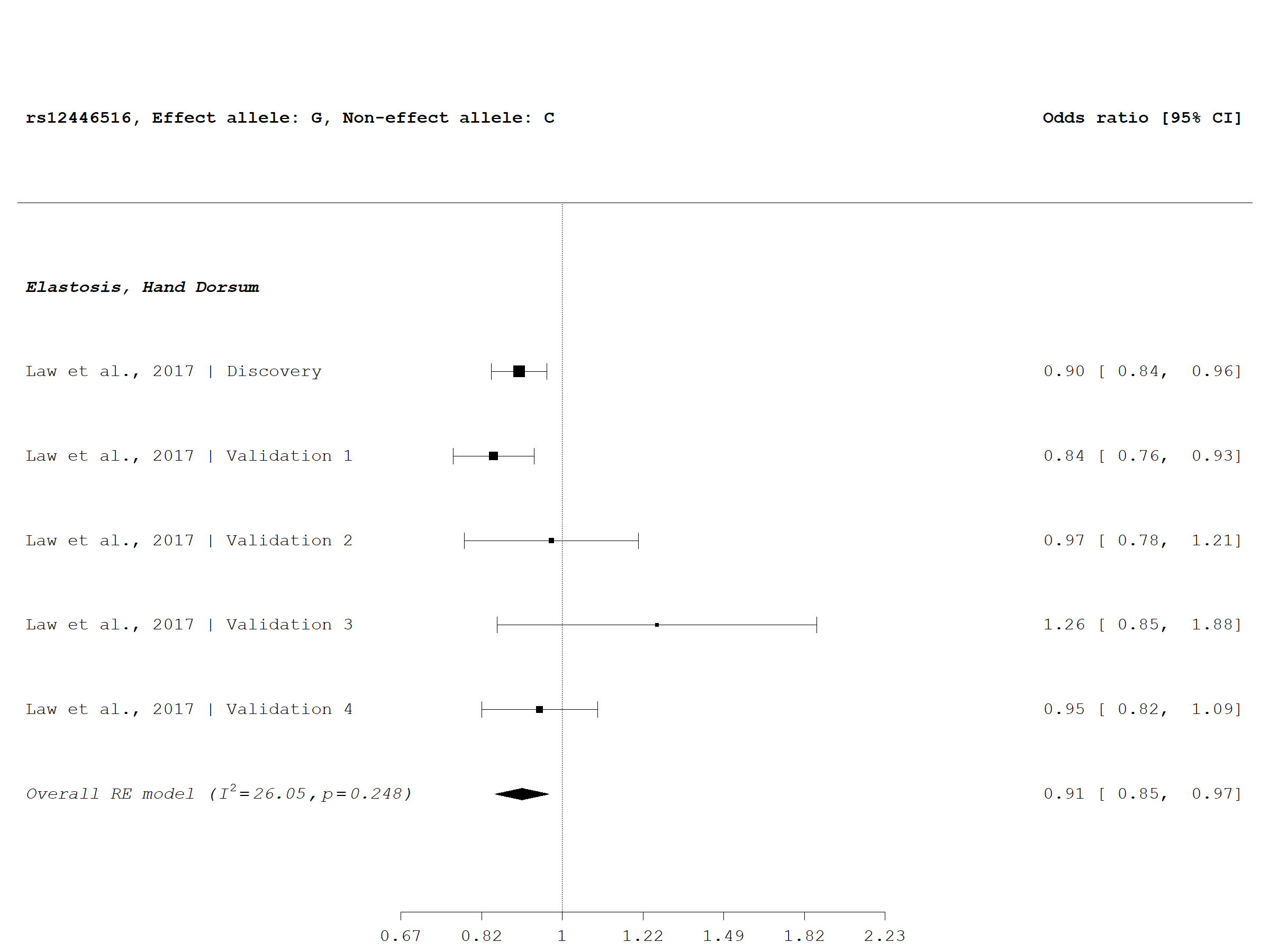

Supplement: Supplementary file 1 — Supplementary Information 1. [file 41598_2022_17443_MOESM1_ESM.zip › Supplementary Datasets/Dataset S3 - Forest Plots/fp188_rs12446516.png]

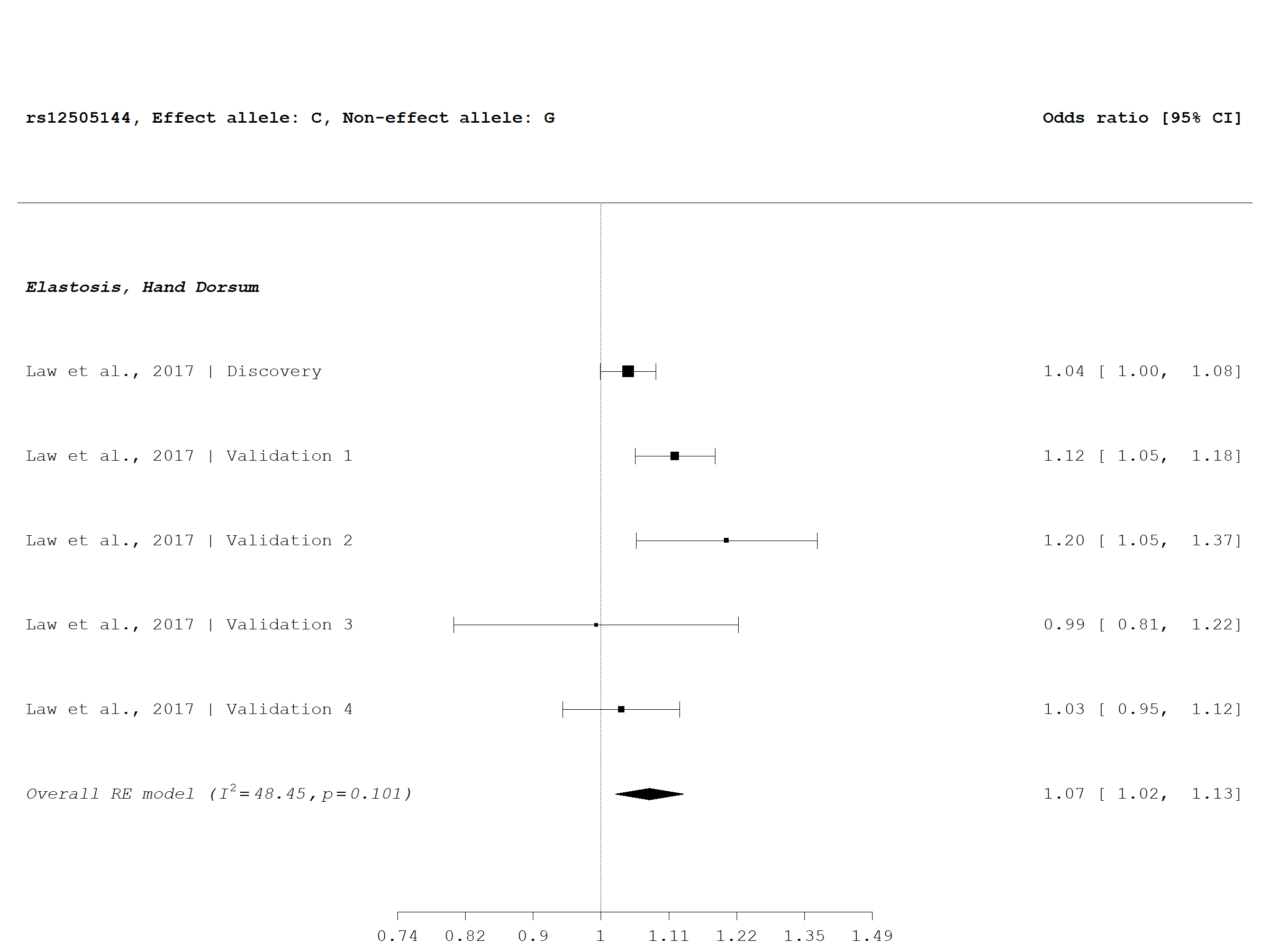

Supplement: Supplementary file 1 — Supplementary Information 1. [file 41598_2022_17443_MOESM1_ESM.zip › Supplementary Datasets/Dataset S3 - Forest Plots/fp189_rs12505144.png]

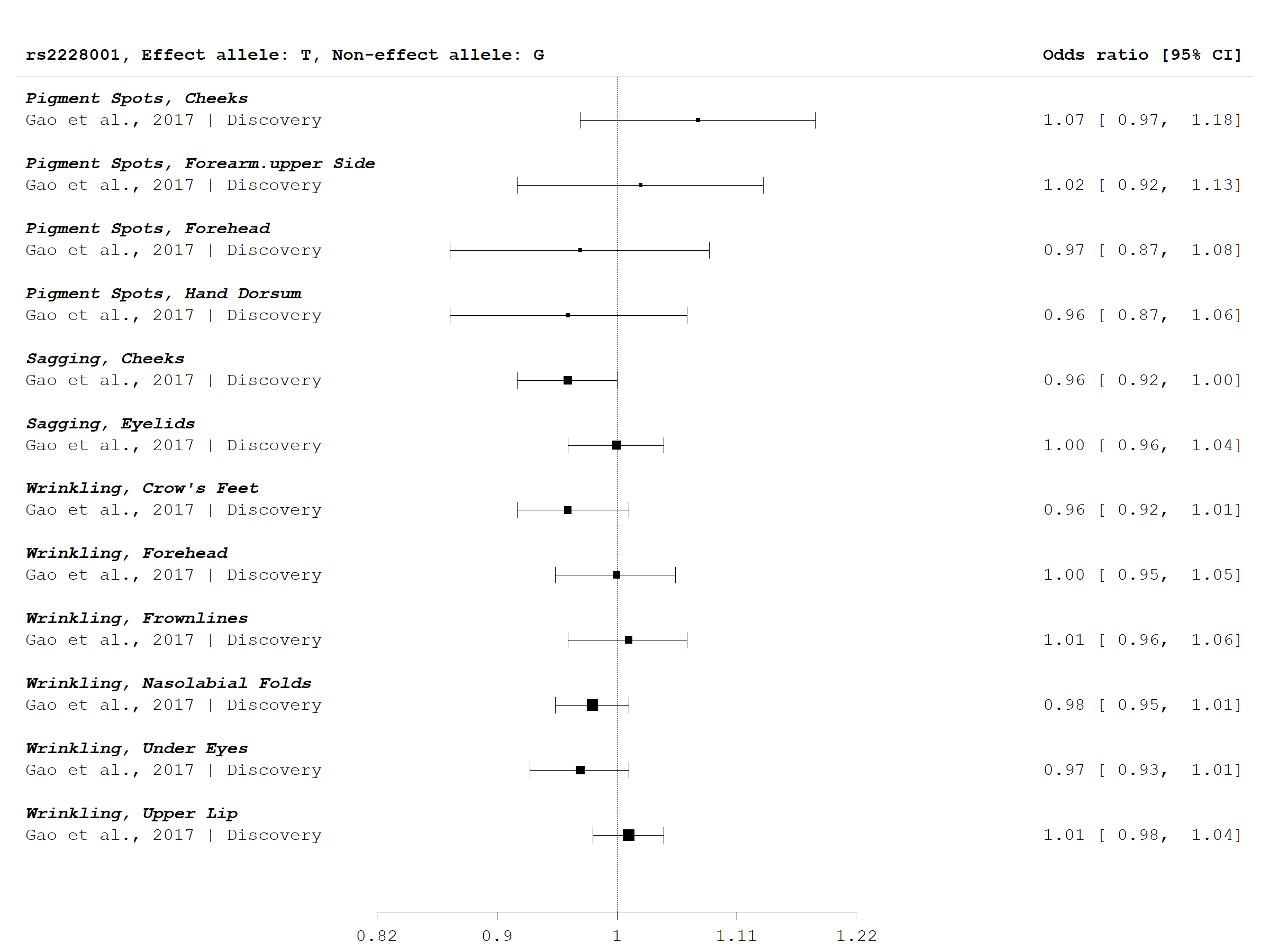

Supplement: Supplementary file 1 — Supplementary Information 1. [file 41598_2022_17443_MOESM1_ESM.zip › Supplementary Datasets/Dataset S3 - Forest Plots/fp18_rs2228001.png]

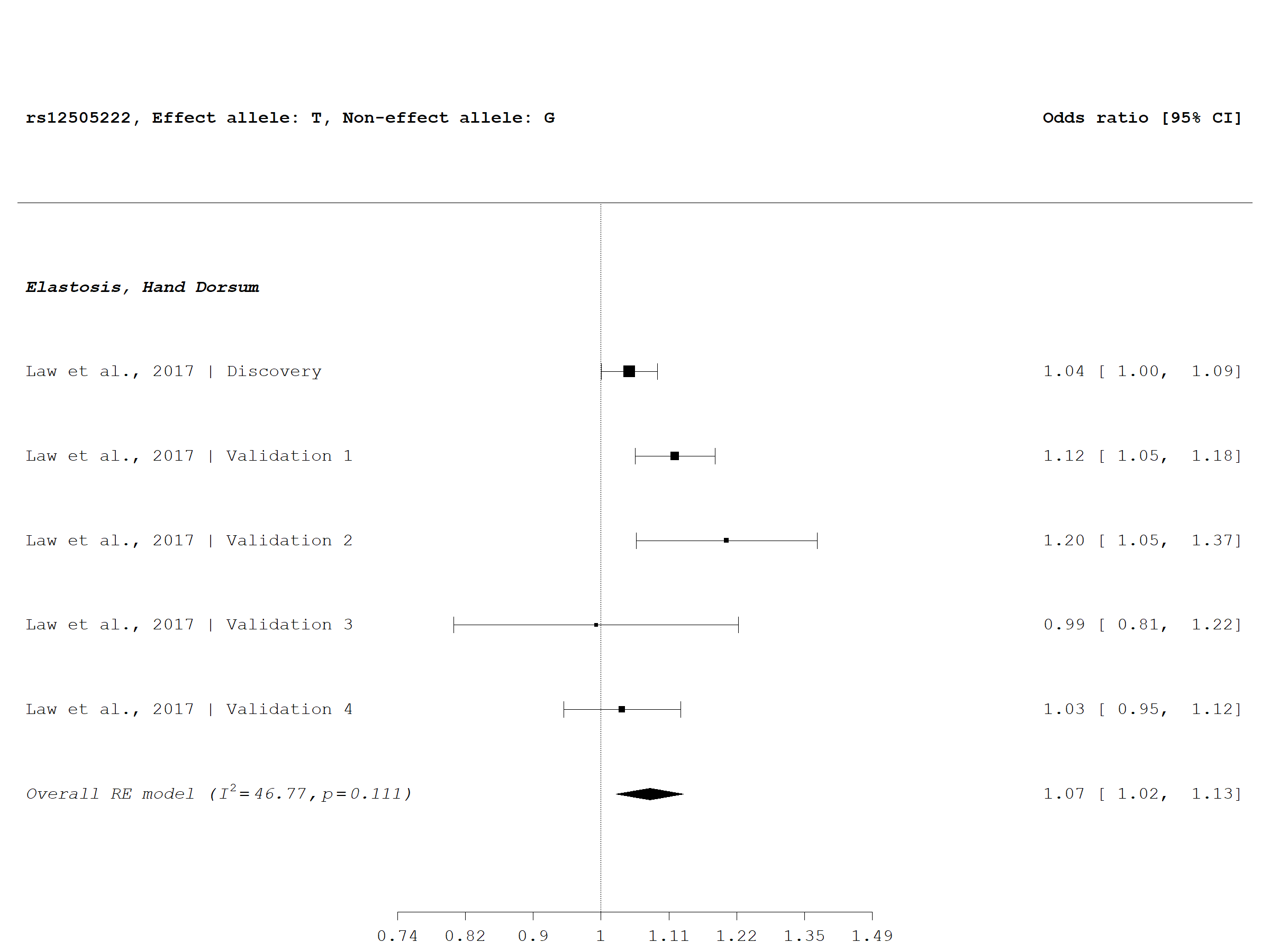

Supplement: Supplementary file 1 — Supplementary Information 1. [file 41598_2022_17443_MOESM1_ESM.zip › Supplementary Datasets/Dataset S3 - Forest Plots/fp190_rs12505222.png]

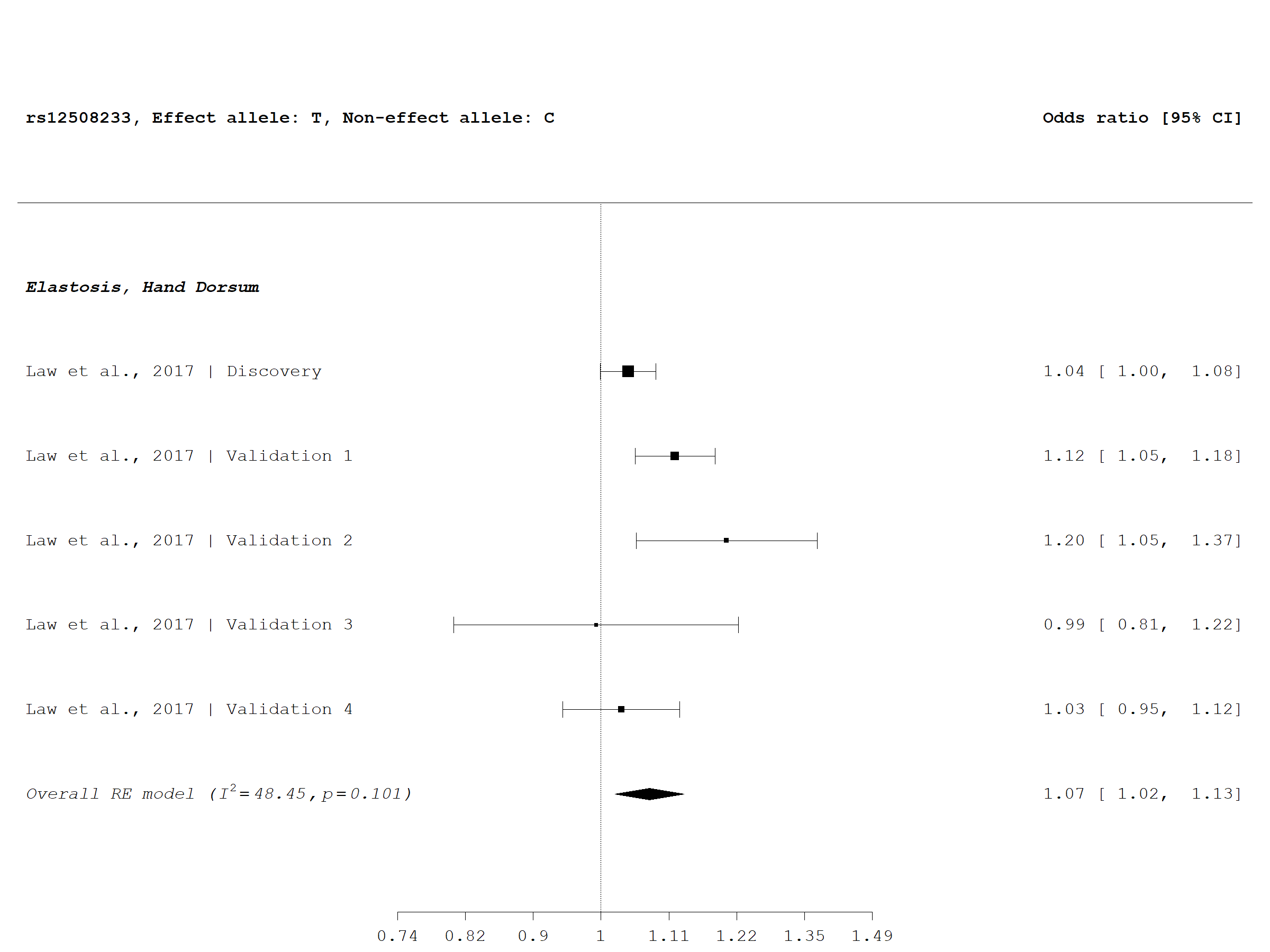

Supplement: Supplementary file 1 — Supplementary Information 1. [file 41598_2022_17443_MOESM1_ESM.zip › Supplementary Datasets/Dataset S3 - Forest Plots/fp191_rs12508233.png]

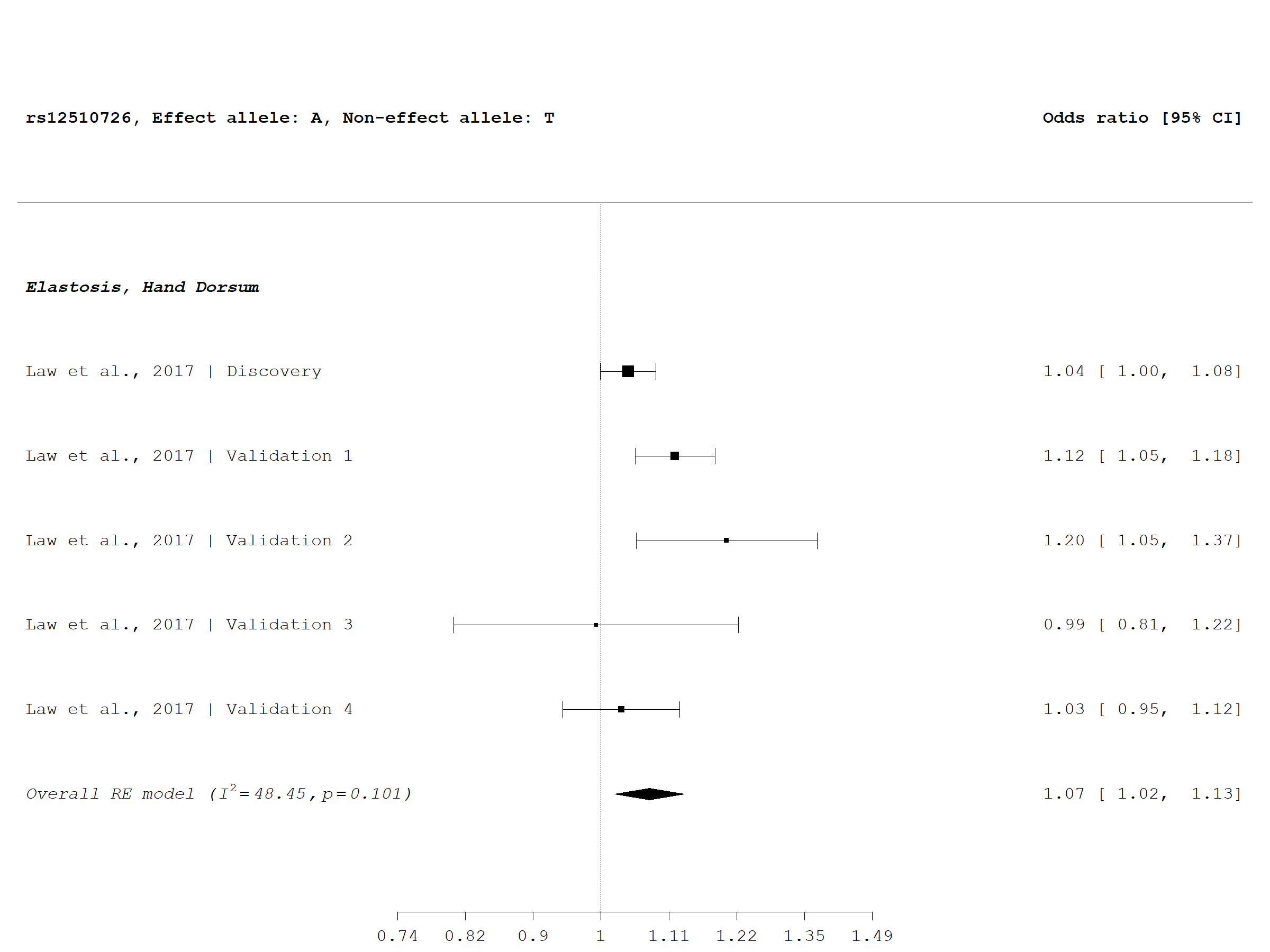

Supplement: Supplementary file 1 — Supplementary Information 1. [file 41598_2022_17443_MOESM1_ESM.zip › Supplementary Datasets/Dataset S3 - Forest Plots/fp192_rs12510726.png]

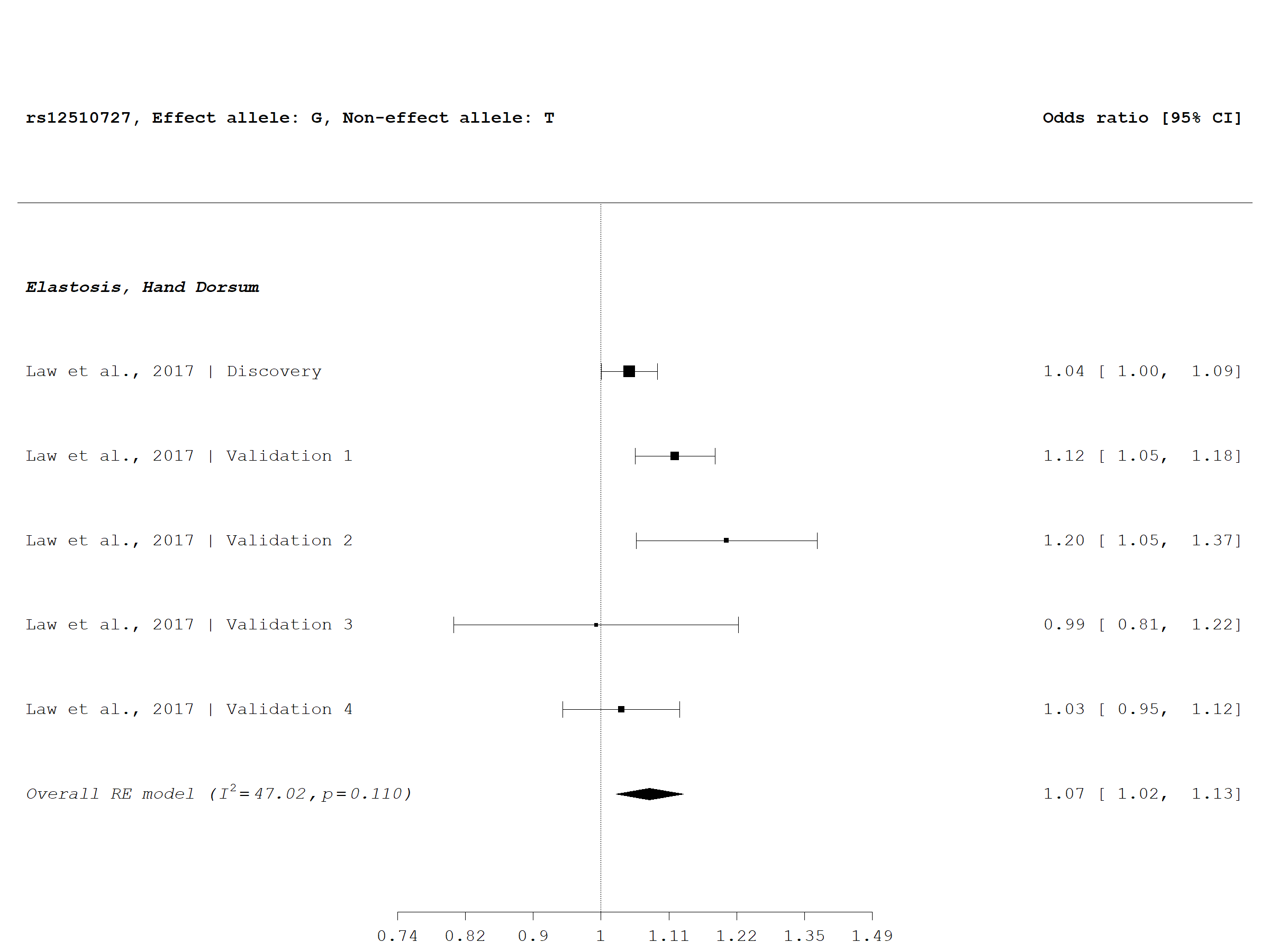

Supplement: Supplementary file 1 — Supplementary Information 1. [file 41598_2022_17443_MOESM1_ESM.zip › Supplementary Datasets/Dataset S3 - Forest Plots/fp193_rs12510727.png]

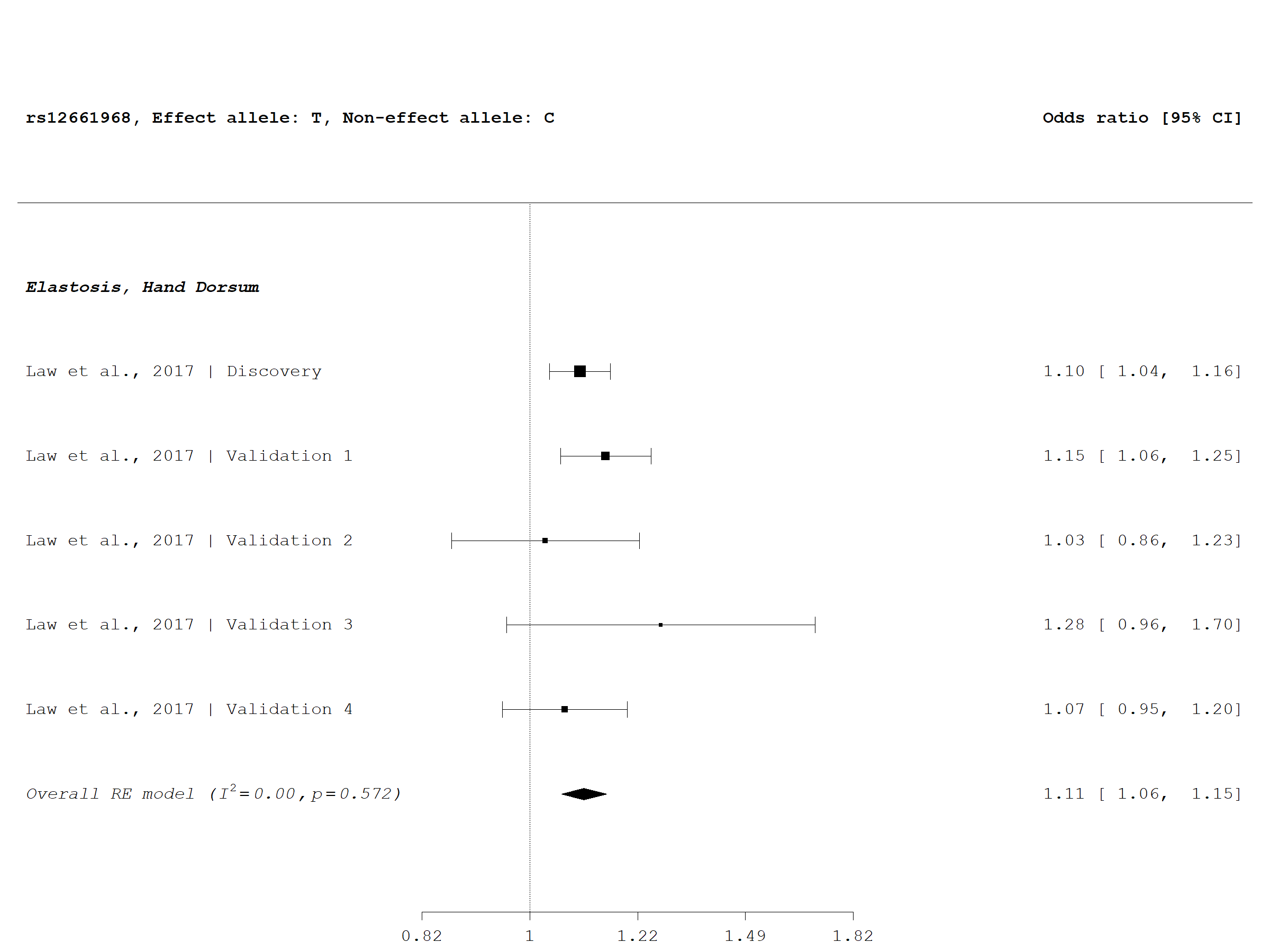

Supplement: Supplementary file 1 — Supplementary Information 1. [file 41598_2022_17443_MOESM1_ESM.zip › Supplementary Datasets/Dataset S3 - Forest Plots/fp194_rs12661968.png]

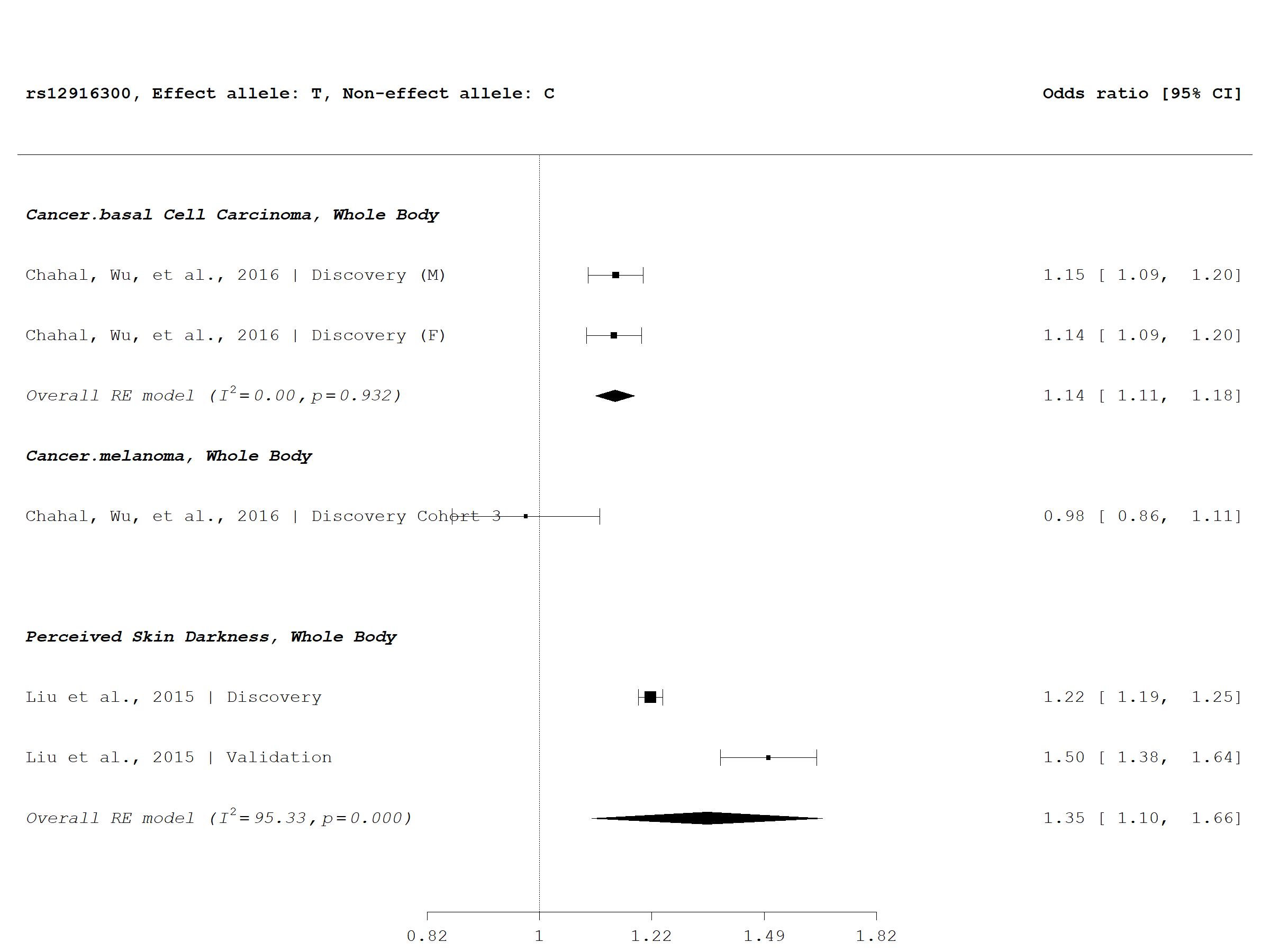

Supplement: Supplementary file 1 — Supplementary Information 1. [file 41598_2022_17443_MOESM1_ESM.zip › Supplementary Datasets/Dataset S3 - Forest Plots/fp195_rs12916300.png]

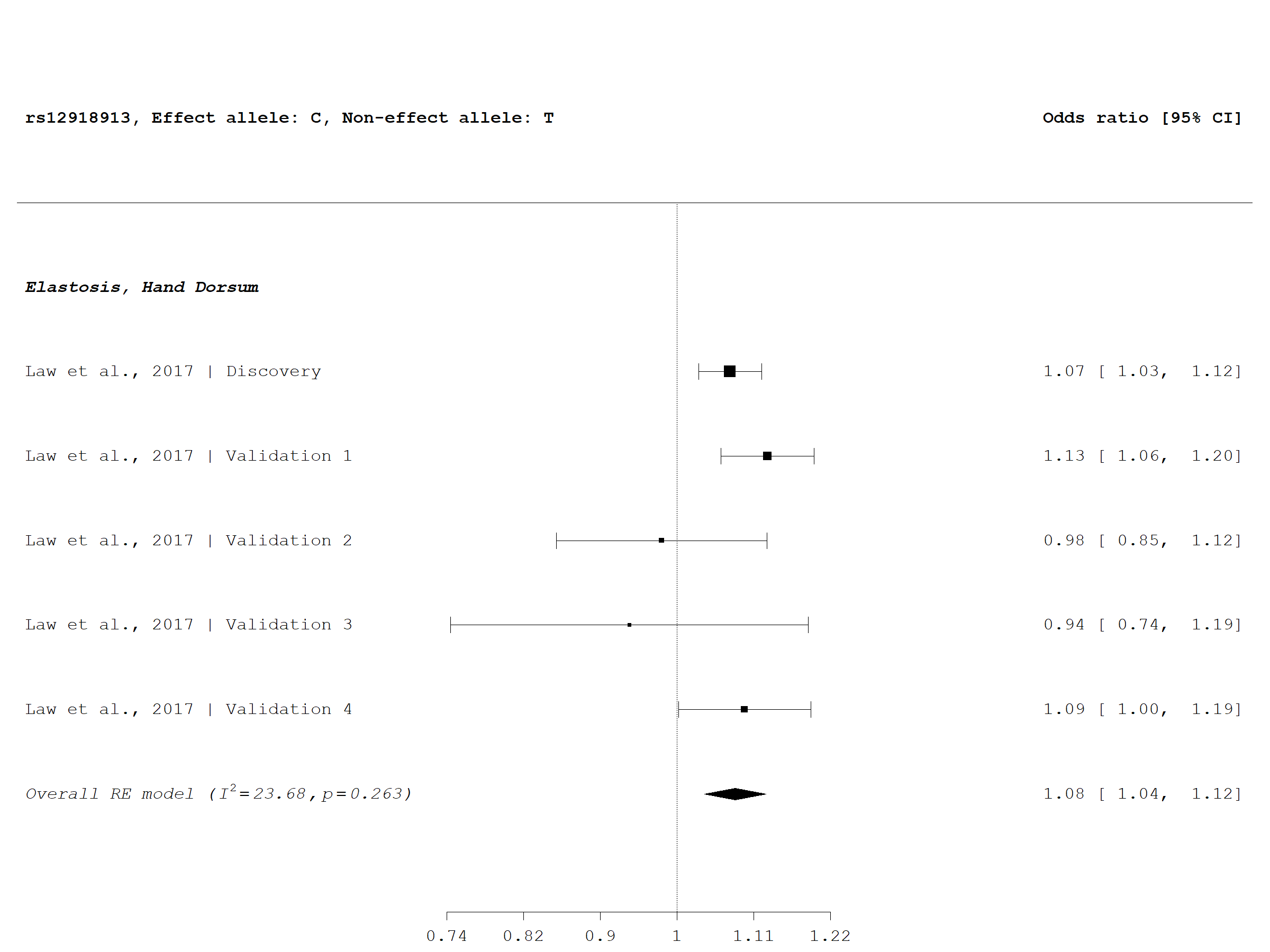

Supplement: Supplementary file 1 — Supplementary Information 1. [file 41598_2022_17443_MOESM1_ESM.zip › Supplementary Datasets/Dataset S3 - Forest Plots/fp196_rs12918913.png]

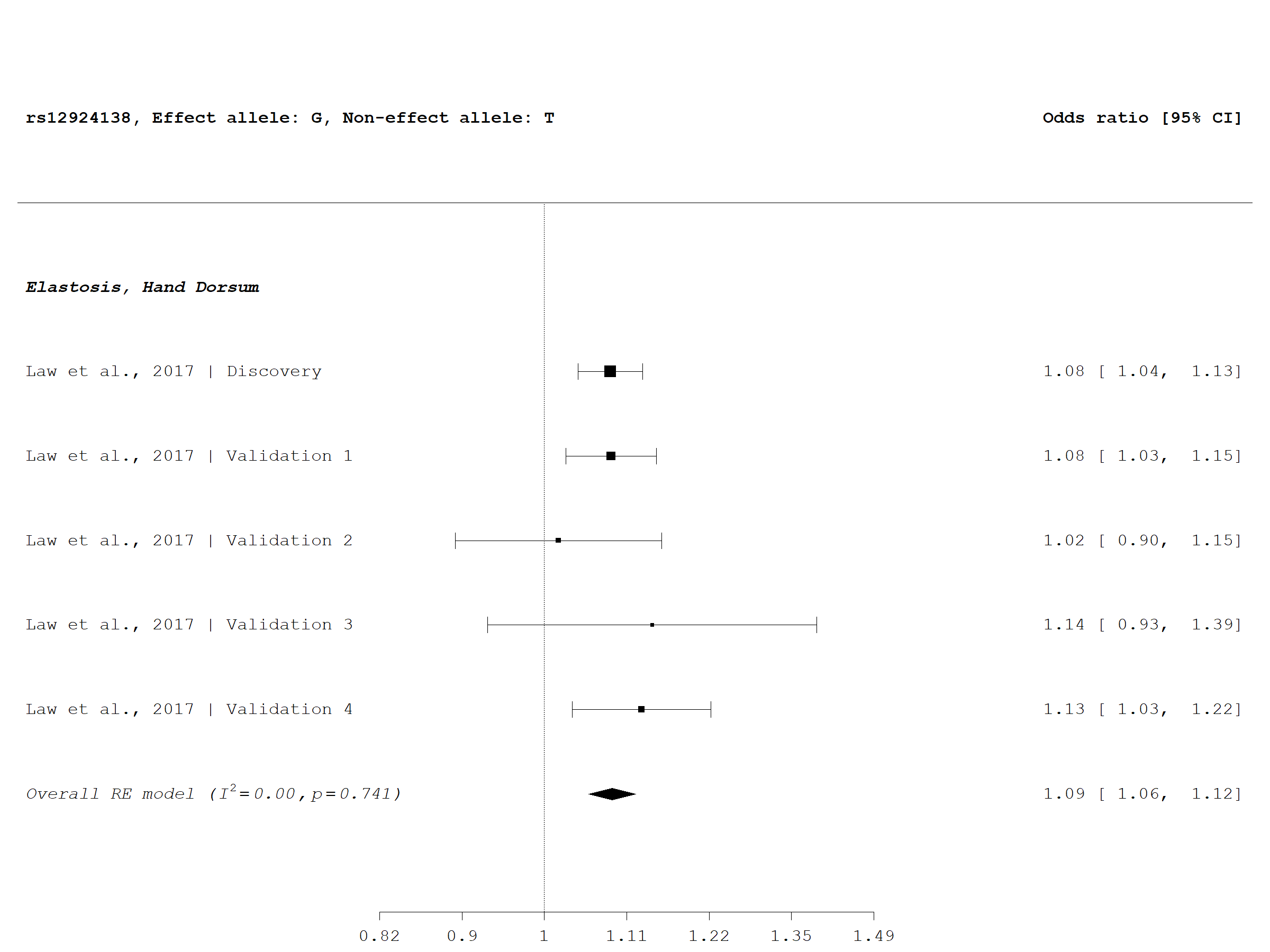

Supplement: Supplementary file 1 — Supplementary Information 1. [file 41598_2022_17443_MOESM1_ESM.zip › Supplementary Datasets/Dataset S3 - Forest Plots/fp197_rs12924138.png]

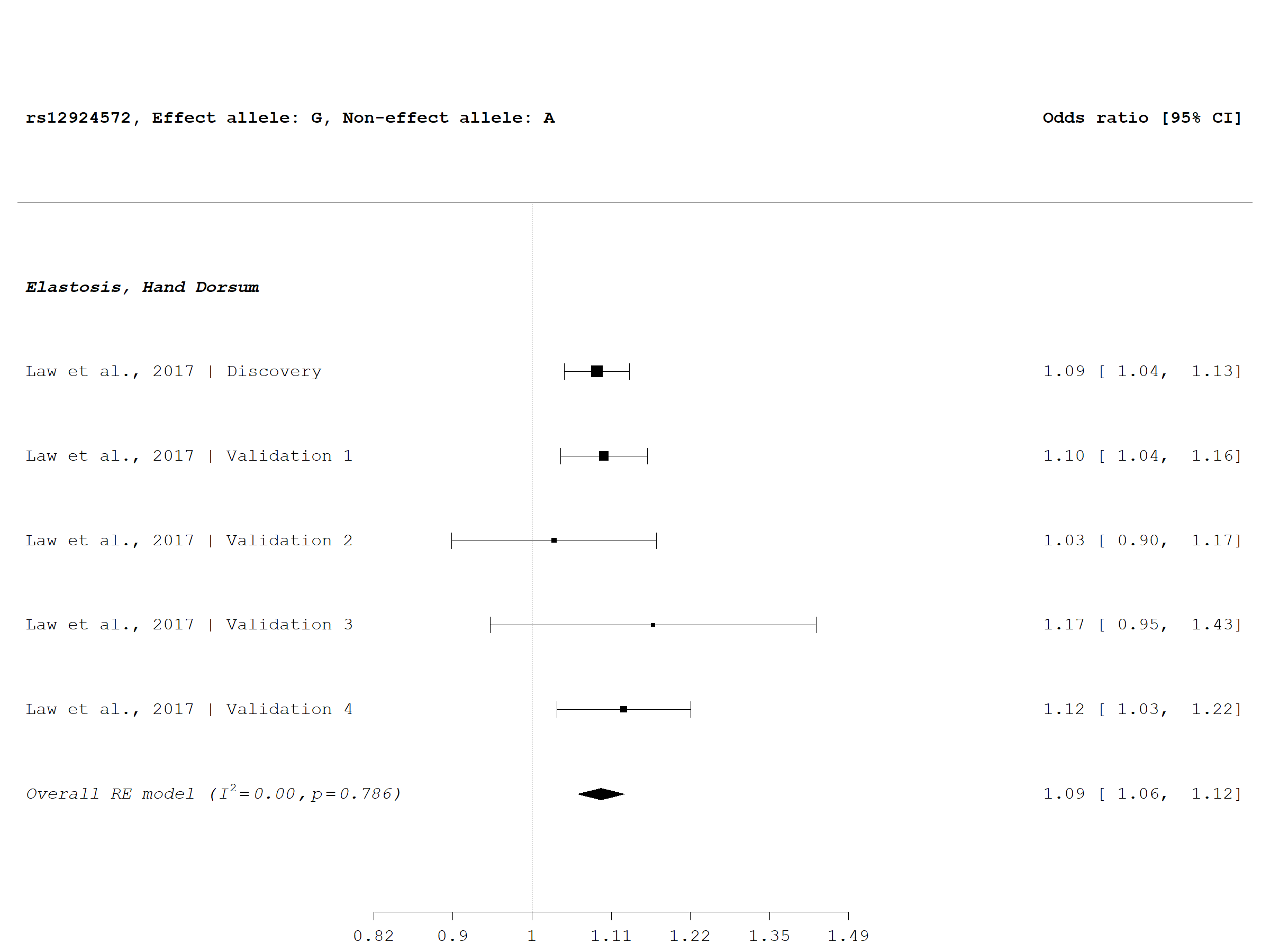

Supplement: Supplementary file 1 — Supplementary Information 1. [file 41598_2022_17443_MOESM1_ESM.zip › Supplementary Datasets/Dataset S3 - Forest Plots/fp198_rs12924572.png]

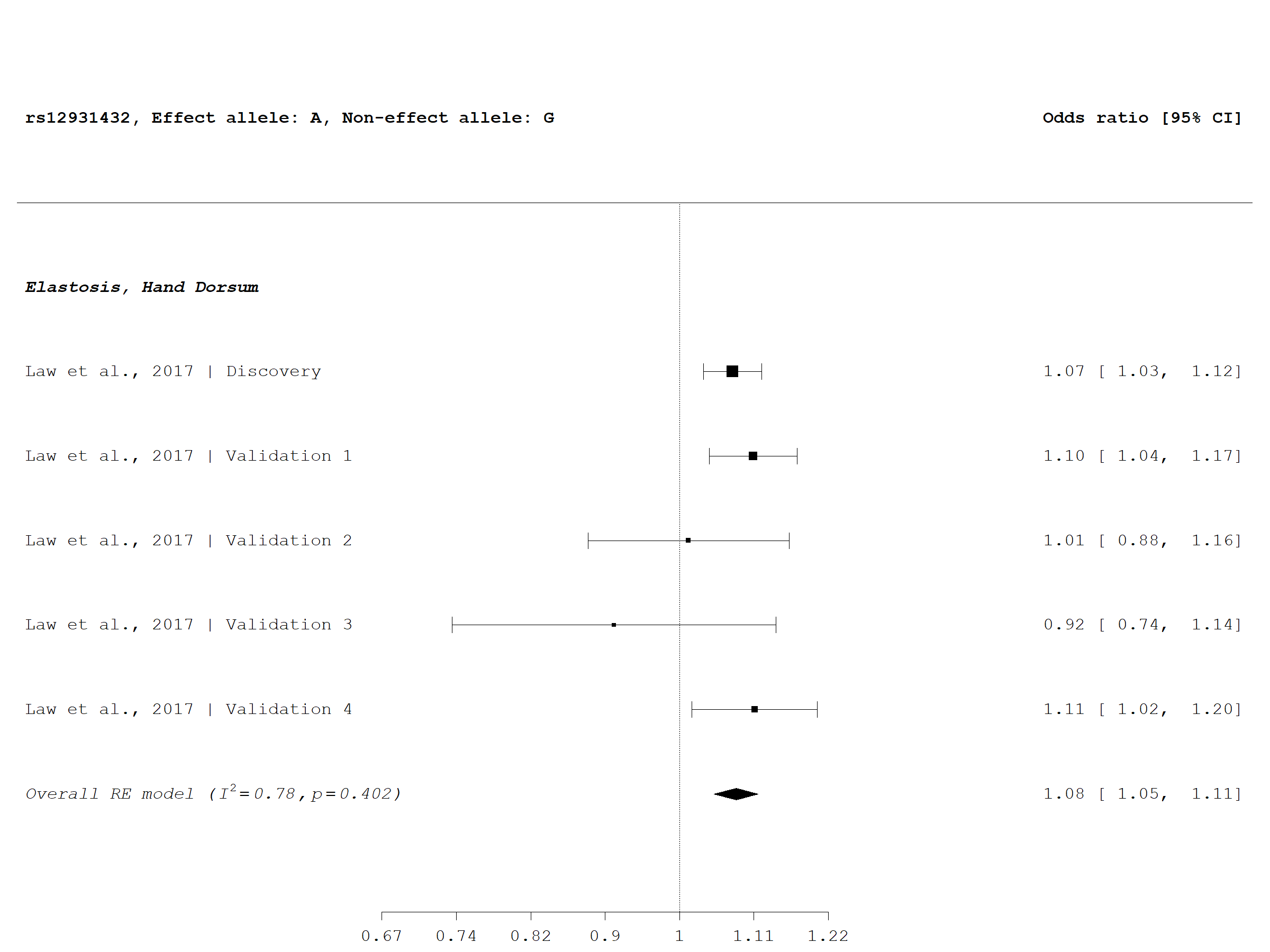

Supplement: Supplementary file 1 — Supplementary Information 1. [file 41598_2022_17443_MOESM1_ESM.zip › Supplementary Datasets/Dataset S3 - Forest Plots/fp199_rs12931432.png]

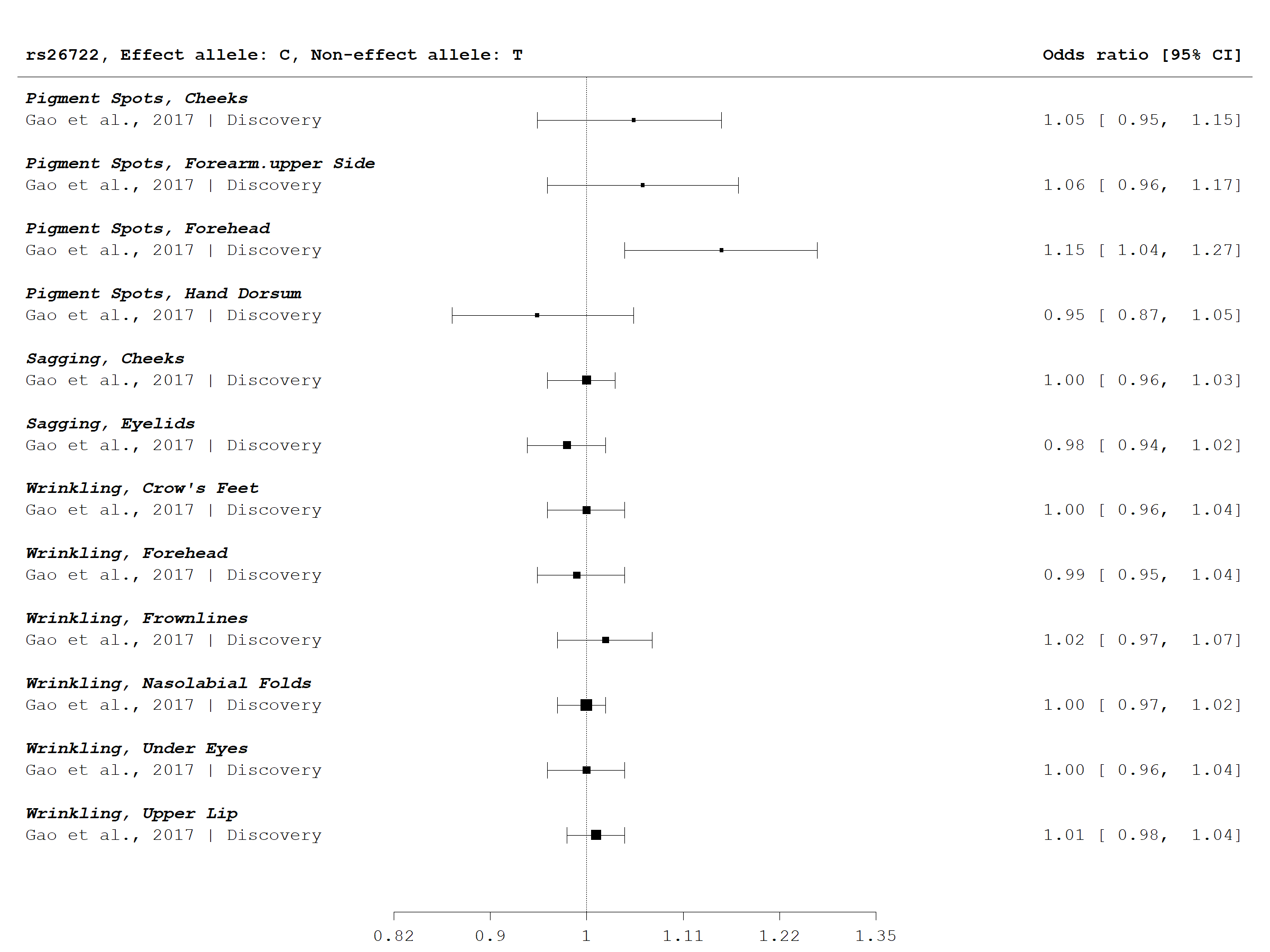

Supplement: Supplementary file 1 — Supplementary Information 1. [file 41598_2022_17443_MOESM1_ESM.zip › Supplementary Datasets/Dataset S3 - Forest Plots/fp19_rs26722.png]

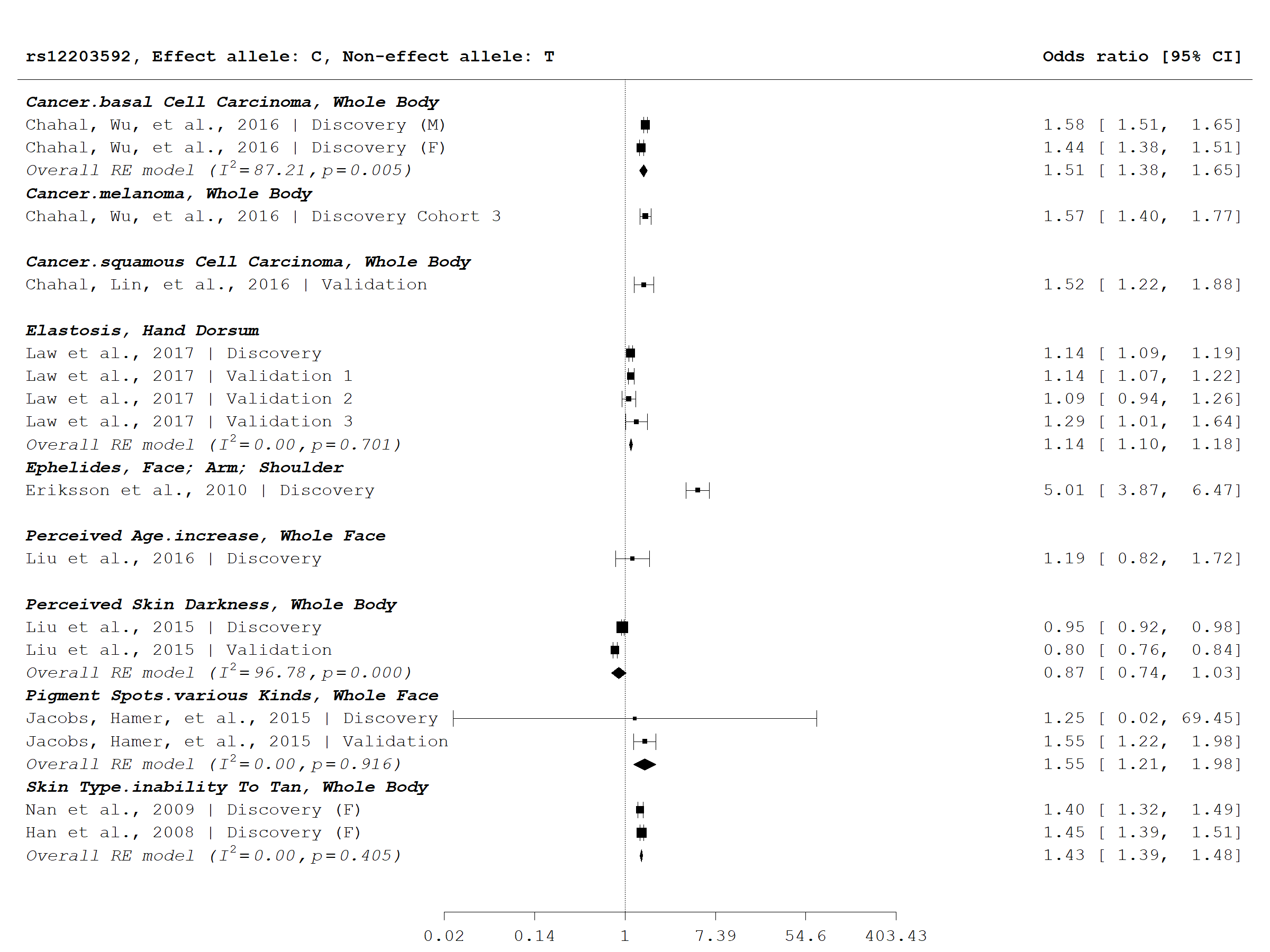

Supplement: Supplementary file 1 — Supplementary Information 1. [file 41598_2022_17443_MOESM1_ESM.zip › Supplementary Datasets/Dataset S3 - Forest Plots/fp1_rs12203592.png]

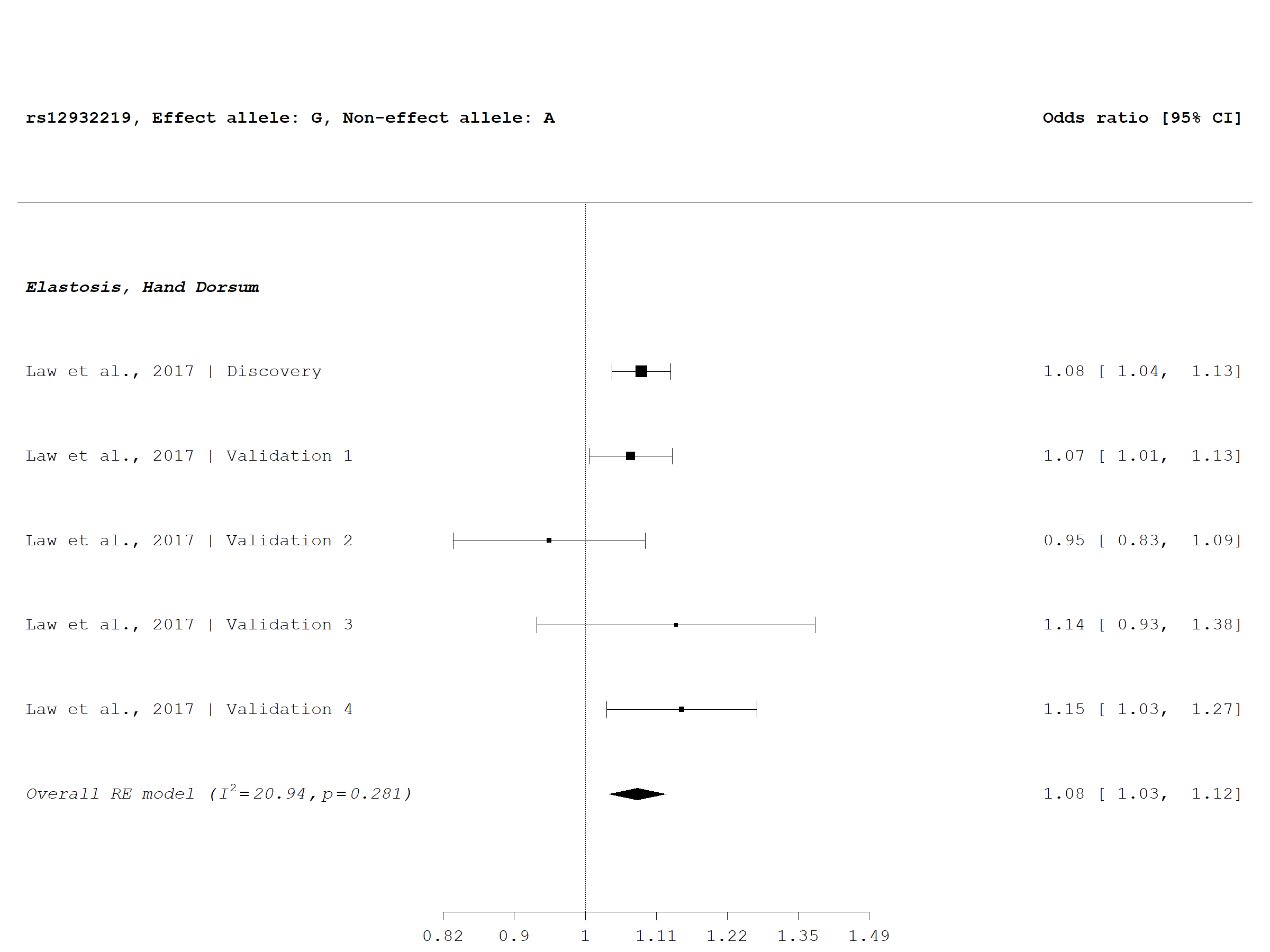

Supplement: Supplementary file 1 — Supplementary Information 1. [file 41598_2022_17443_MOESM1_ESM.zip › Supplementary Datasets/Dataset S3 - Forest Plots/fp200_rs12932219.png]

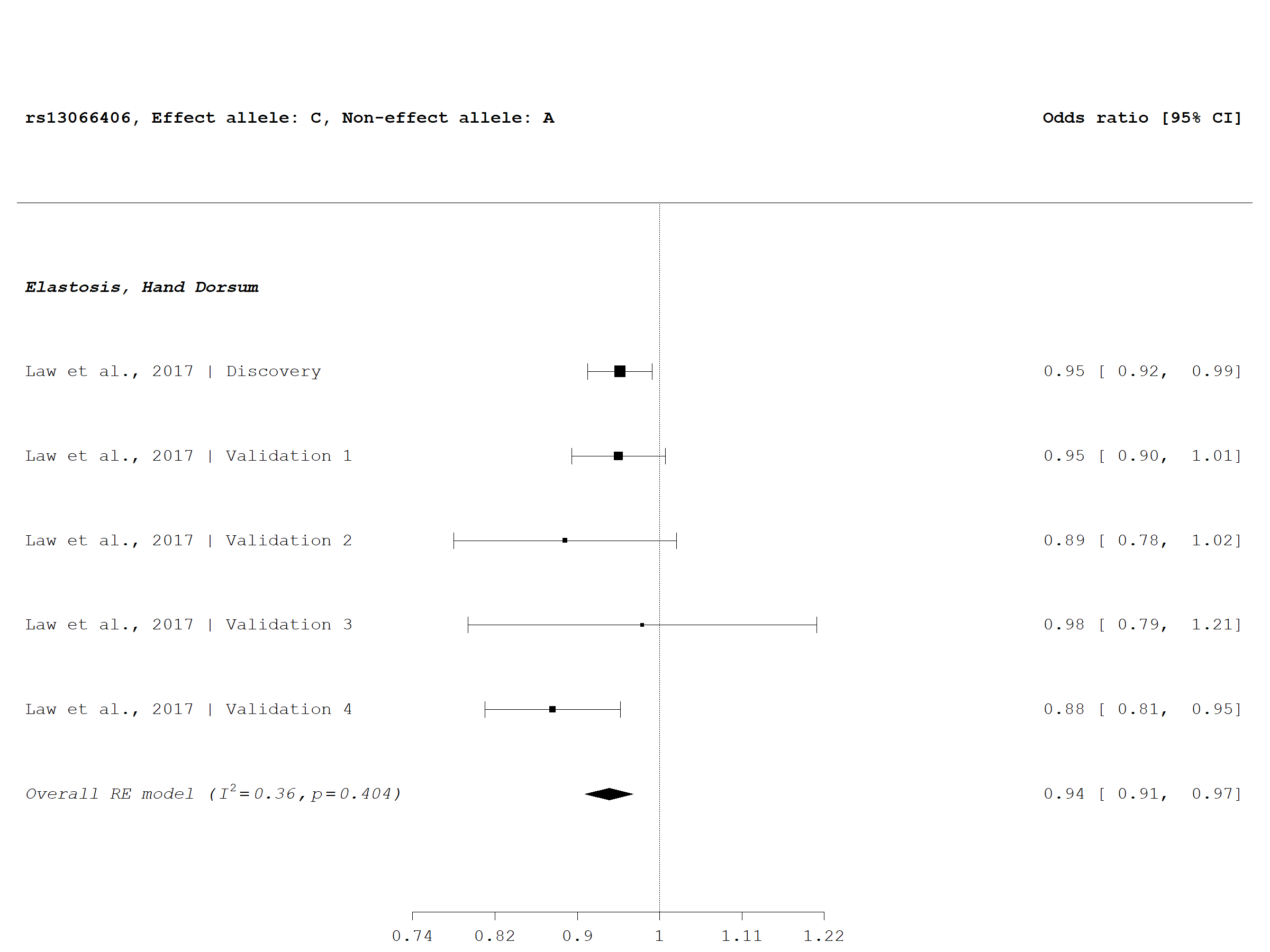

Supplement: Supplementary file 1 — Supplementary Information 1. [file 41598_2022_17443_MOESM1_ESM.zip › Supplementary Datasets/Dataset S3 - Forest Plots/fp201_rs13066406.png]

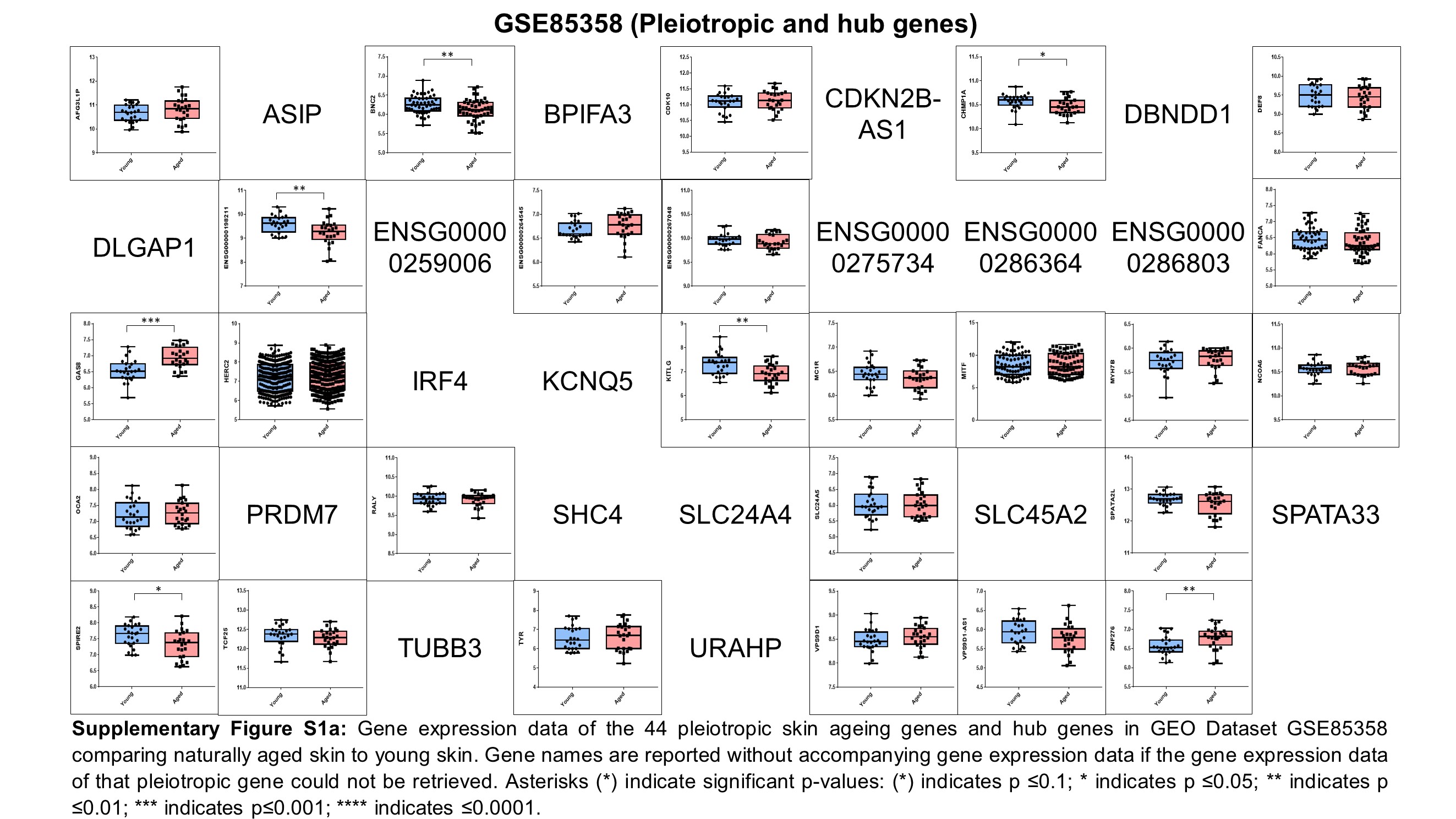

Supplement: Supplementary file 2 — Supplementary Information 2. [file 41598_2022_17443_MOESM2_ESM.zip › Supplementary Information/Figure S1 - GEO Dataset GSE85358/Supplementary Figure S1a.JPG]

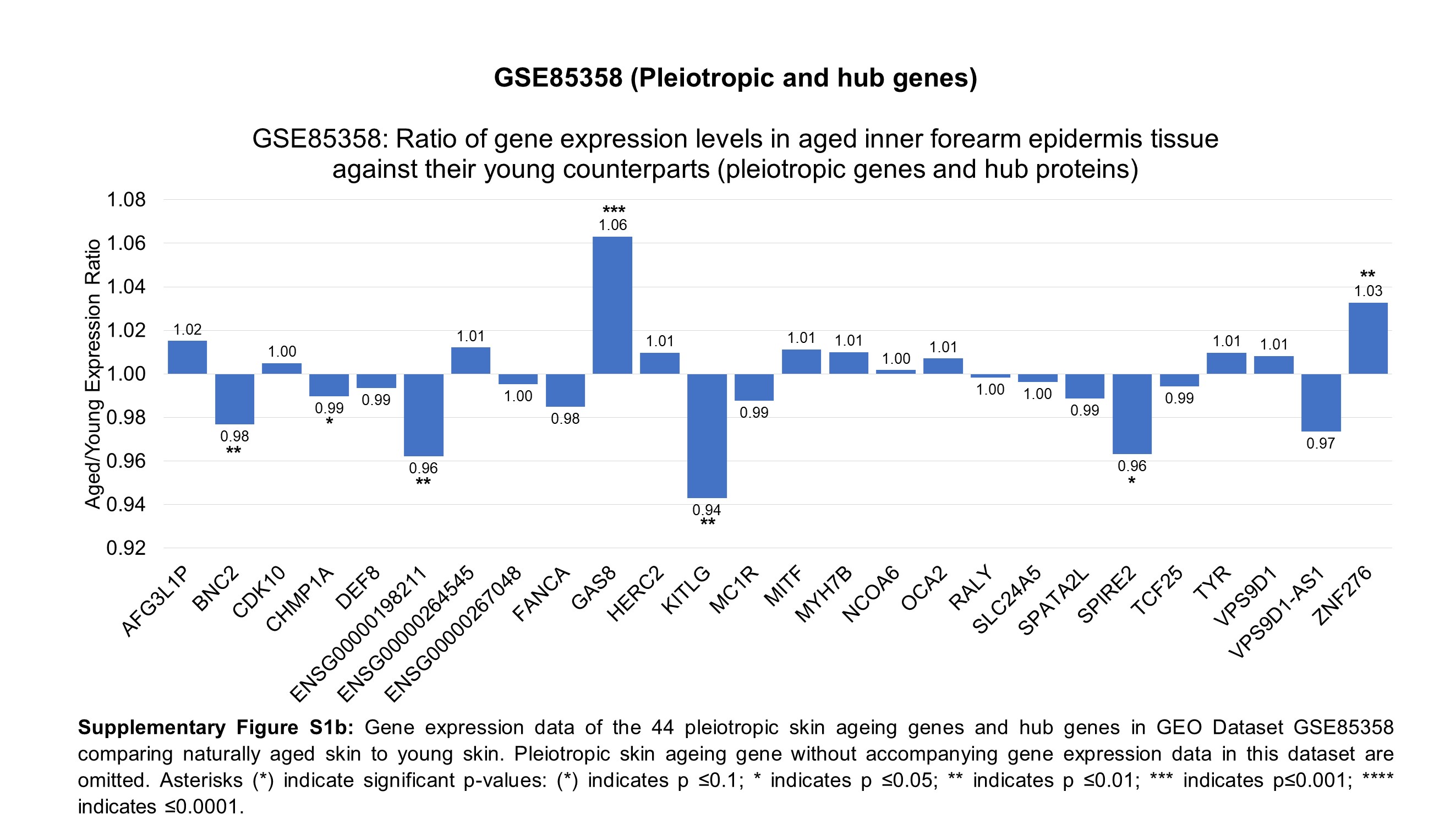

Supplement: Supplementary file 2 — Supplementary Information 2. [file 41598_2022_17443_MOESM2_ESM.zip › Supplementary Information/Figure S1 - GEO Dataset GSE85358/Supplementary Figure S1b.JPG]

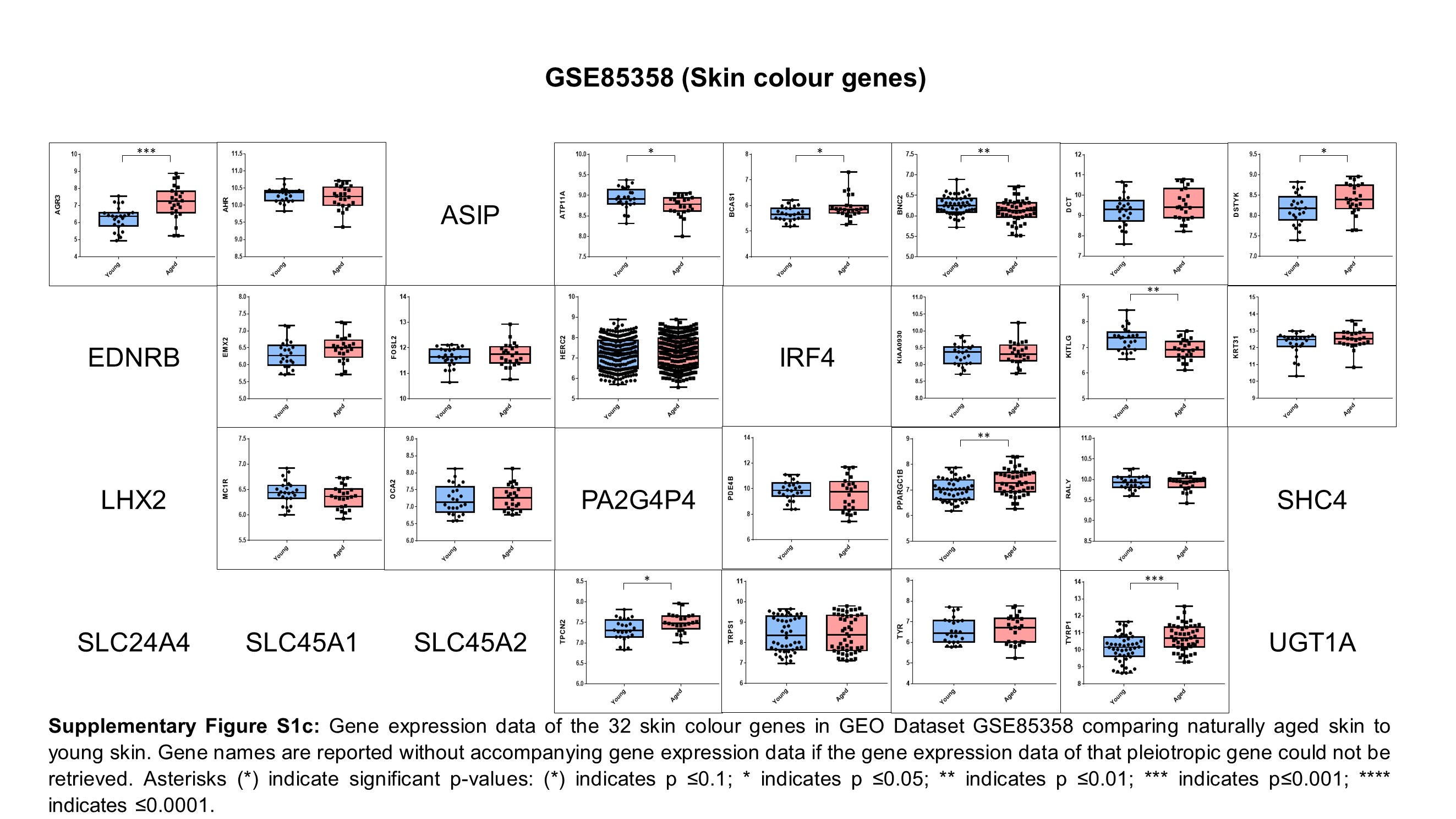

Supplement: Supplementary file 2 — Supplementary Information 2. [file 41598_2022_17443_MOESM2_ESM.zip › Supplementary Information/Figure S1 - GEO Dataset GSE85358/Supplementary Figure S1c.JPG]

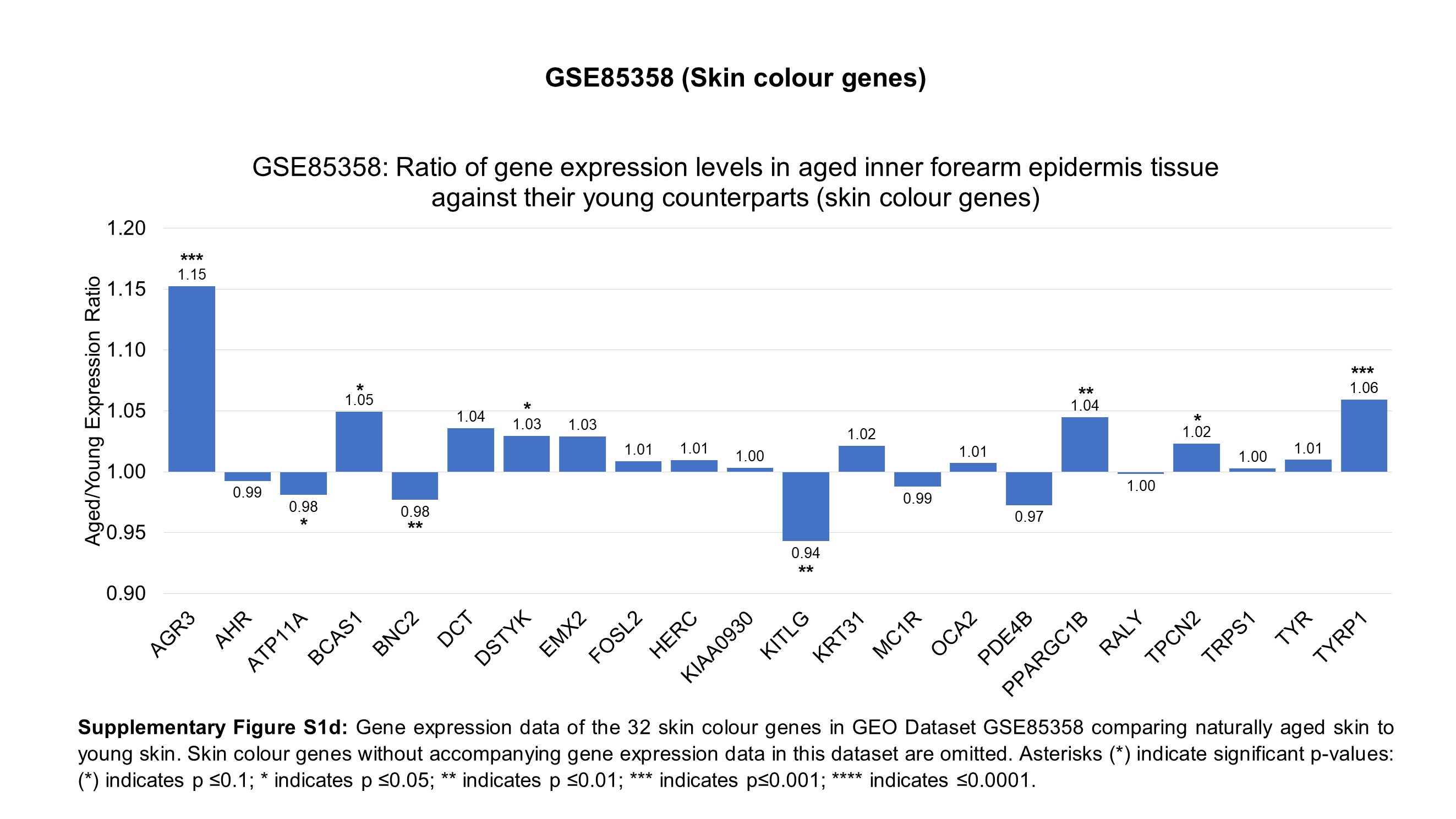

Supplement: Supplementary file 2 — Supplementary Information 2. [file 41598_2022_17443_MOESM2_ESM.zip › Supplementary Information/Figure S1 - GEO Dataset GSE85358/Supplementary Figure S1d.JPG]
