# Supplementary figures and images for: A systematic review of skin ageing genes: gene pleiotropy and genes on the chromosomal band 16q24.3 may drive skin ageing (part 1 of 3)
Source: Sci Rep. 2022 Jul 30;12:13099. doi: 10.1038/s41598-022-17443-1 (PMC9338925; doi:10.1038/s41598-022-17443-1)

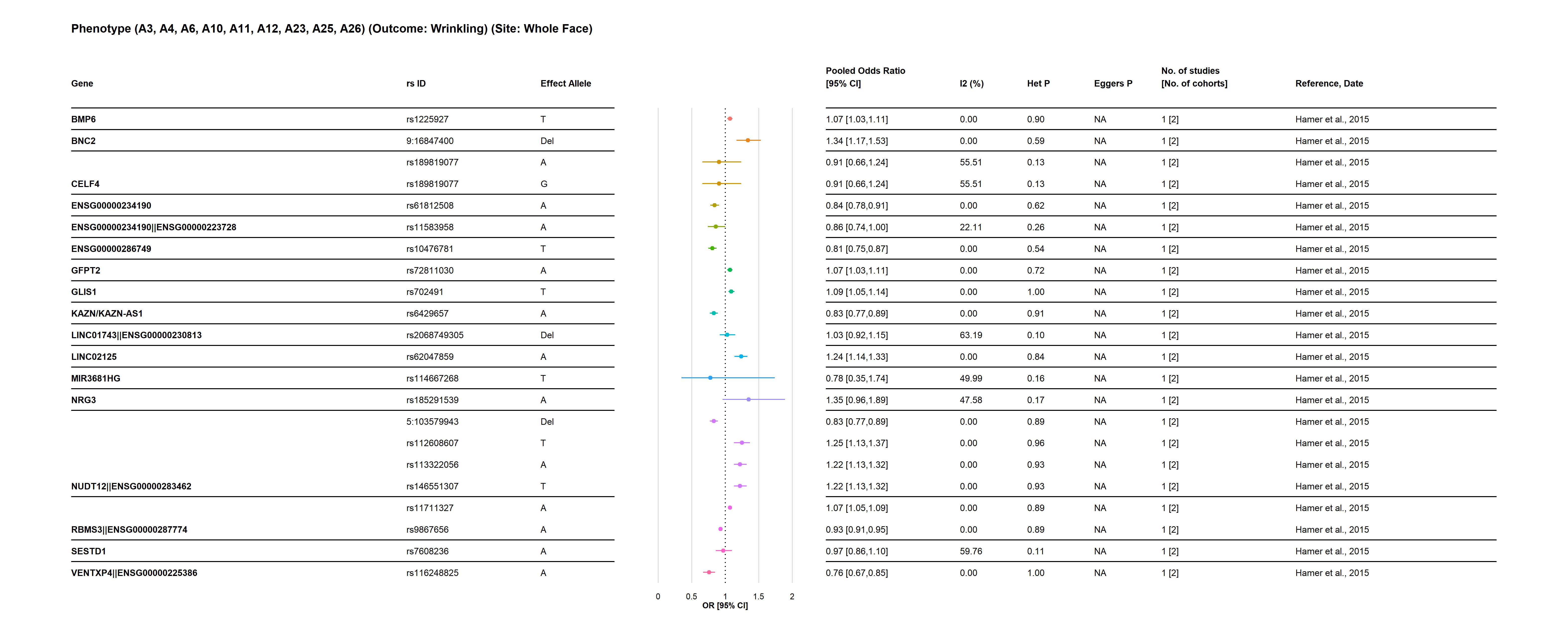

Supplement: Supplementary file 1 — Supplementary Information 1. [file 41598_2022_17443_MOESM1_ESM.zip › Supplementary Datasets/Dataset S1 - SNP-Phenotype Associations with Discovery and Validation Cohorts/1 study 2 cohorts Phenotype (A3, A4, A6, A10, A11, A12, A23, A25, A26) (Outcome_Wrinkling) (Site_Whole Face).jpg]

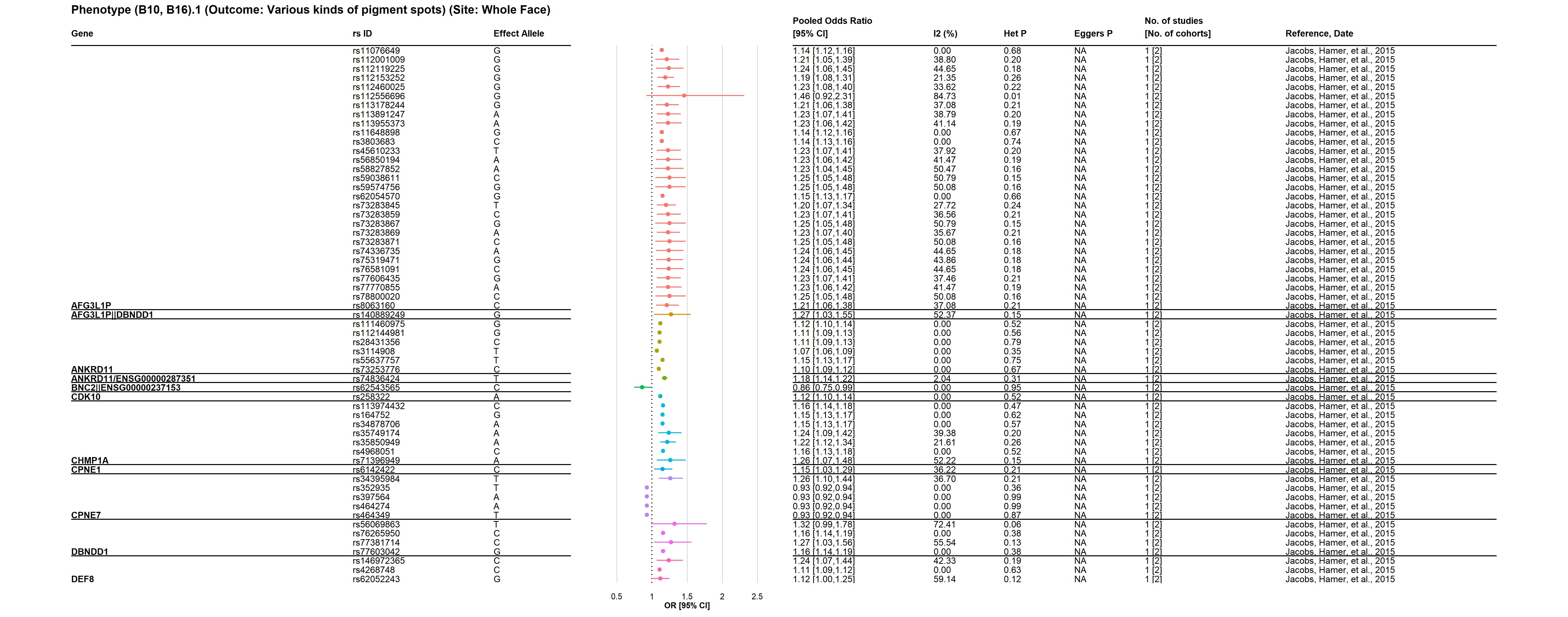

Supplement: Supplementary file 1 — Supplementary Information 1. [file 41598_2022_17443_MOESM1_ESM.zip › Supplementary Datasets/Dataset S1 - SNP-Phenotype Associations with Discovery and Validation Cohorts/1 study 2 cohorts Phenotype (B10, B16).1 (Outcome_Various kinds of pigment spots) (Site_Whole Face).jpg]

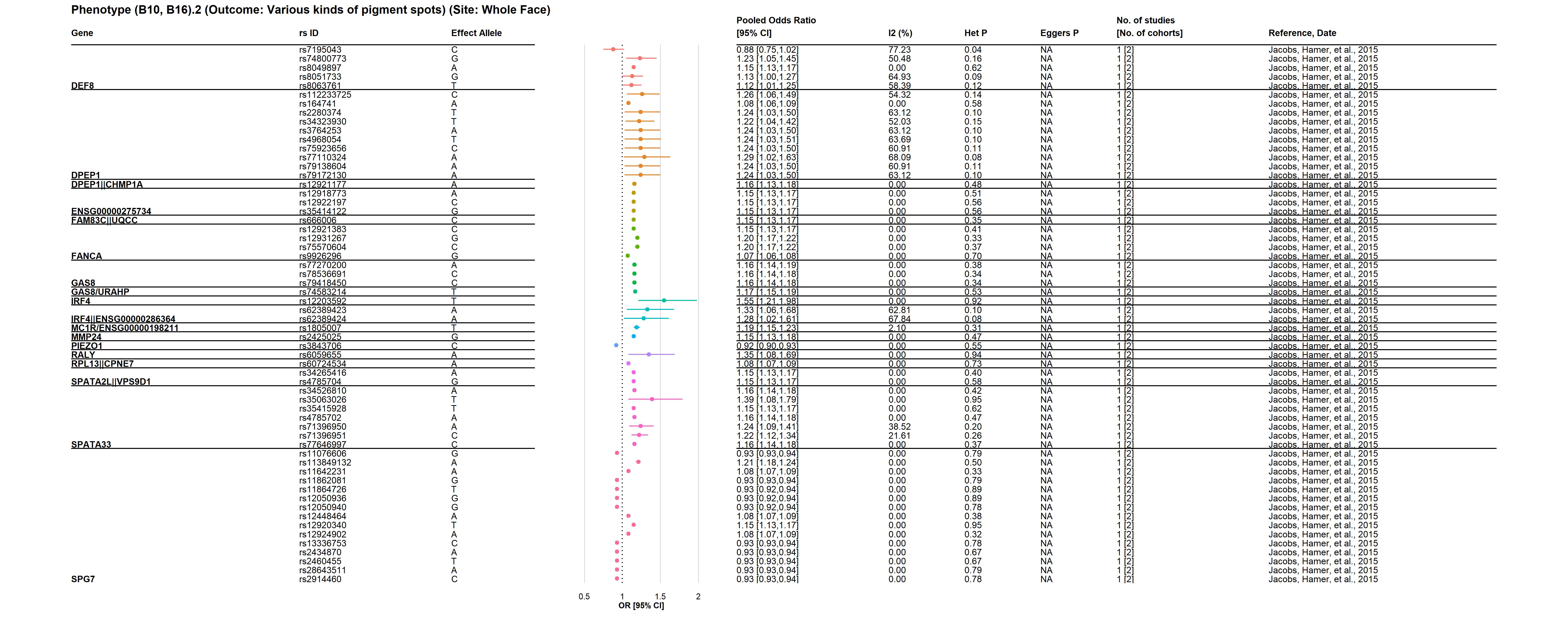

Supplement: Supplementary file 1 — Supplementary Information 1. [file 41598_2022_17443_MOESM1_ESM.zip › Supplementary Datasets/Dataset S1 - SNP-Phenotype Associations with Discovery and Validation Cohorts/1 study 2 cohorts Phenotype (B10, B16).2 (Outcome_Various kinds of pigment spots) (Site_Whole Face).jpg]

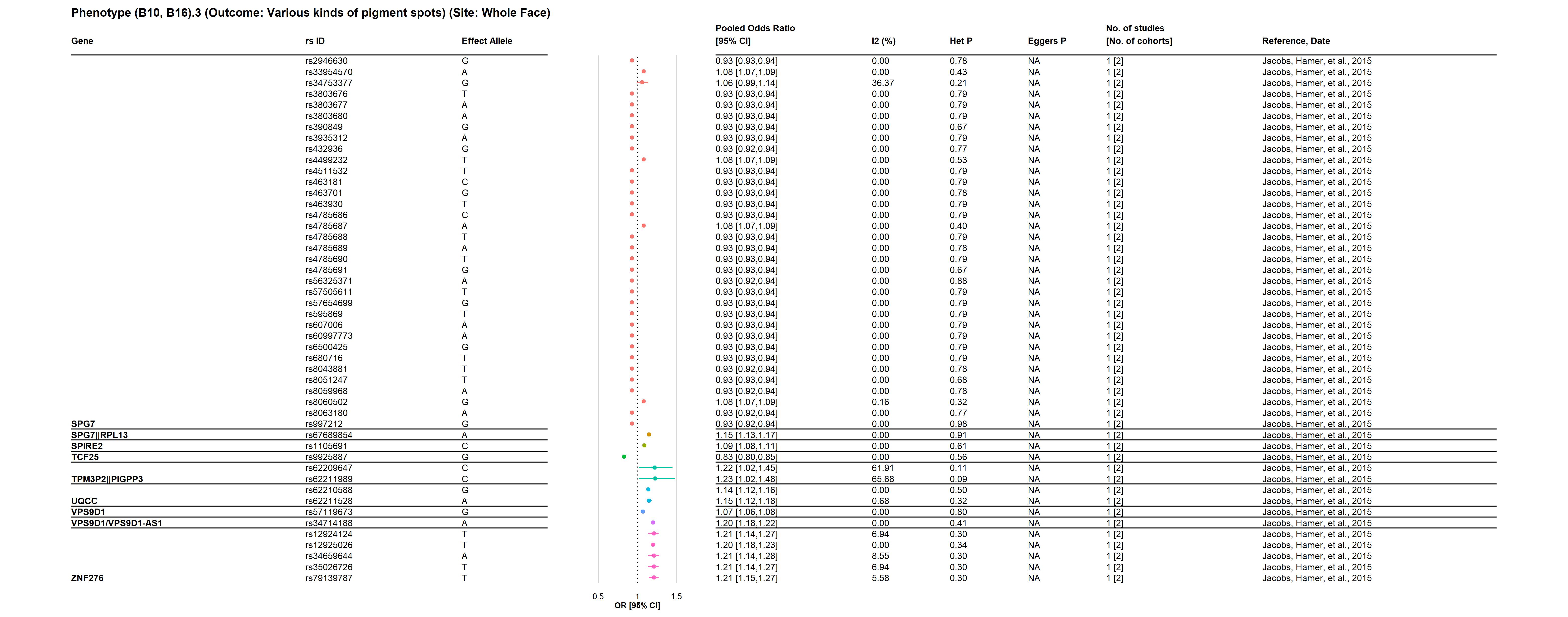

Supplement: Supplementary file 1 — Supplementary Information 1. [file 41598_2022_17443_MOESM1_ESM.zip › Supplementary Datasets/Dataset S1 - SNP-Phenotype Associations with Discovery and Validation Cohorts/1 study 2 cohorts Phenotype (B10, B16).3 (Outcome_Various kinds of pigment spots) (Site_Whole Face).jpg]

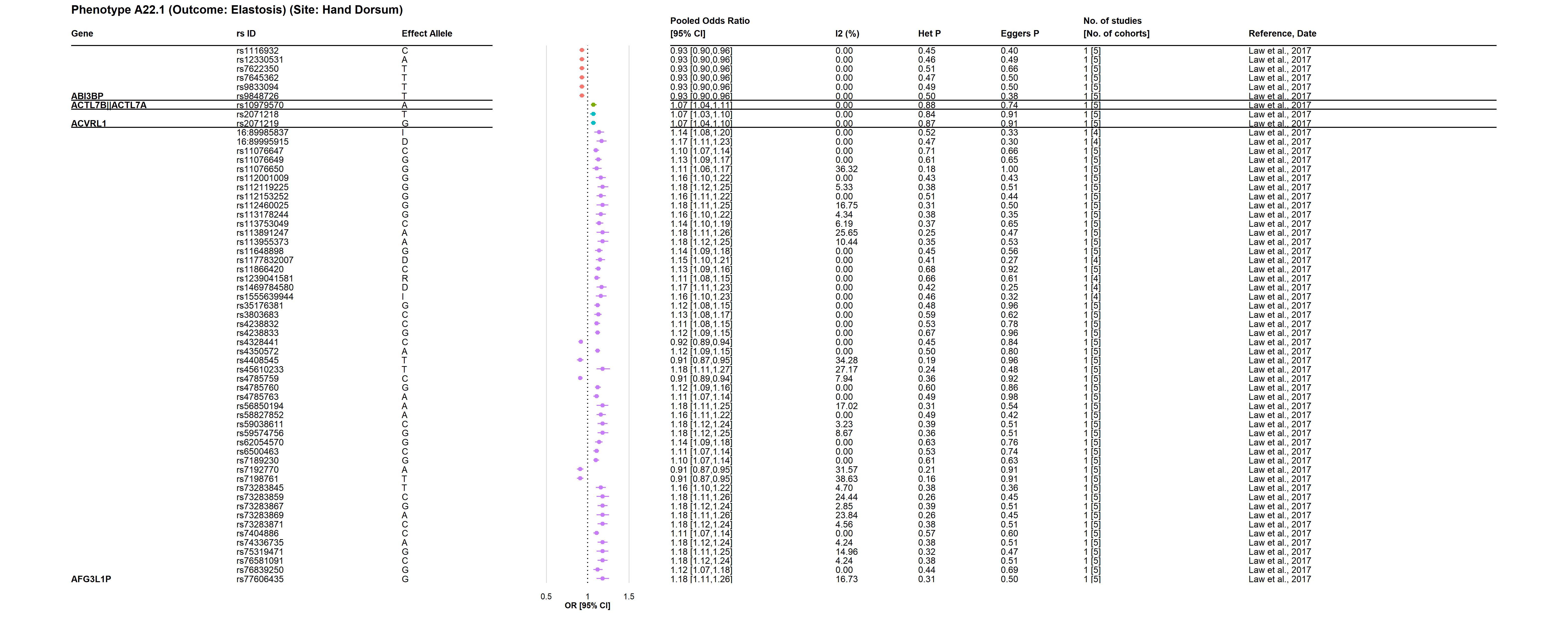

Supplement: Supplementary file 1 — Supplementary Information 1. [file 41598_2022_17443_MOESM1_ESM.zip › Supplementary Datasets/Dataset S1 - SNP-Phenotype Associations with Discovery and Validation Cohorts/1 study 2 cohorts Phenotype A22.1 (Outcome_Elastosis) (Site_Hand Dorsum).jpg]

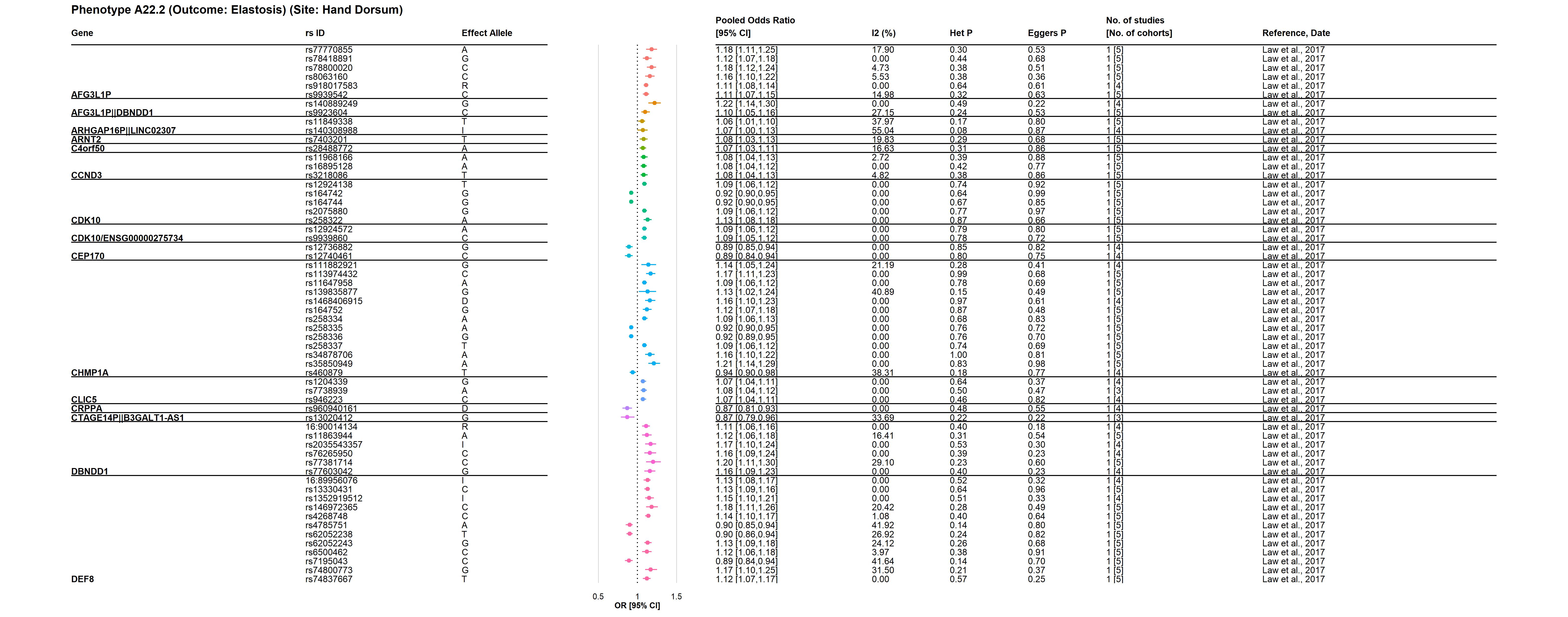

Supplement: Supplementary file 1 — Supplementary Information 1. [file 41598_2022_17443_MOESM1_ESM.zip › Supplementary Datasets/Dataset S1 - SNP-Phenotype Associations with Discovery and Validation Cohorts/1 study 2 cohorts Phenotype A22.2 (Outcome_Elastosis) (Site_Hand Dorsum).jpg]

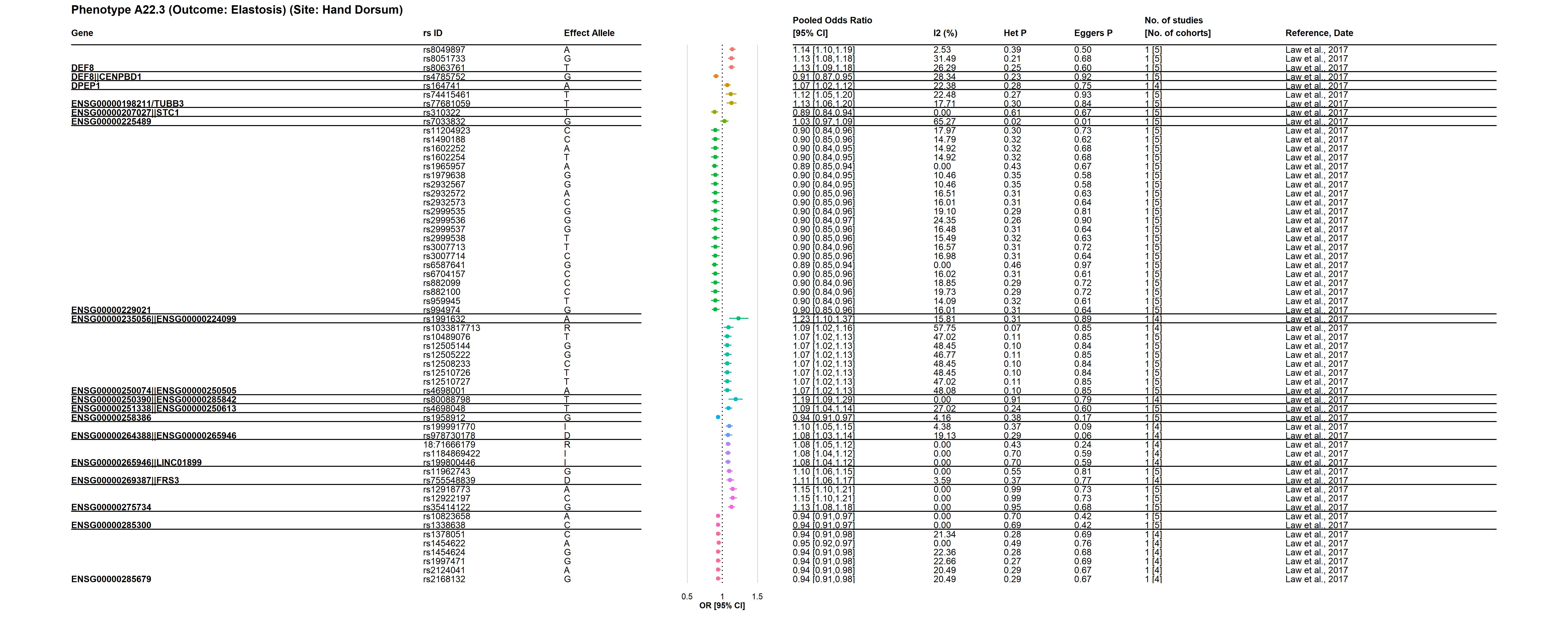

Supplement: Supplementary file 1 — Supplementary Information 1. [file 41598_2022_17443_MOESM1_ESM.zip › Supplementary Datasets/Dataset S1 - SNP-Phenotype Associations with Discovery and Validation Cohorts/1 study 2 cohorts Phenotype A22.3 (Outcome_Elastosis) (Site_Hand Dorsum).jpg]

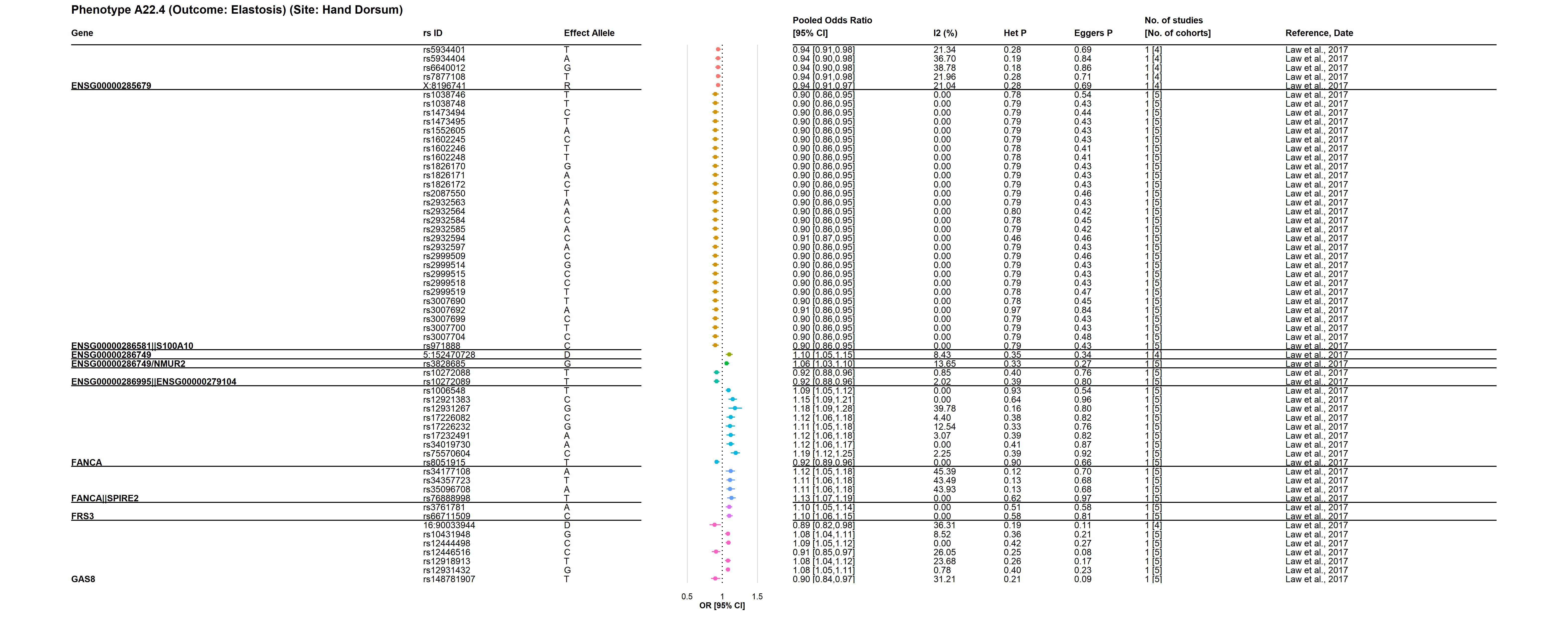

Supplement: Supplementary file 1 — Supplementary Information 1. [file 41598_2022_17443_MOESM1_ESM.zip › Supplementary Datasets/Dataset S1 - SNP-Phenotype Associations with Discovery and Validation Cohorts/1 study 2 cohorts Phenotype A22.4 (Outcome_Elastosis) (Site_Hand Dorsum).jpg]

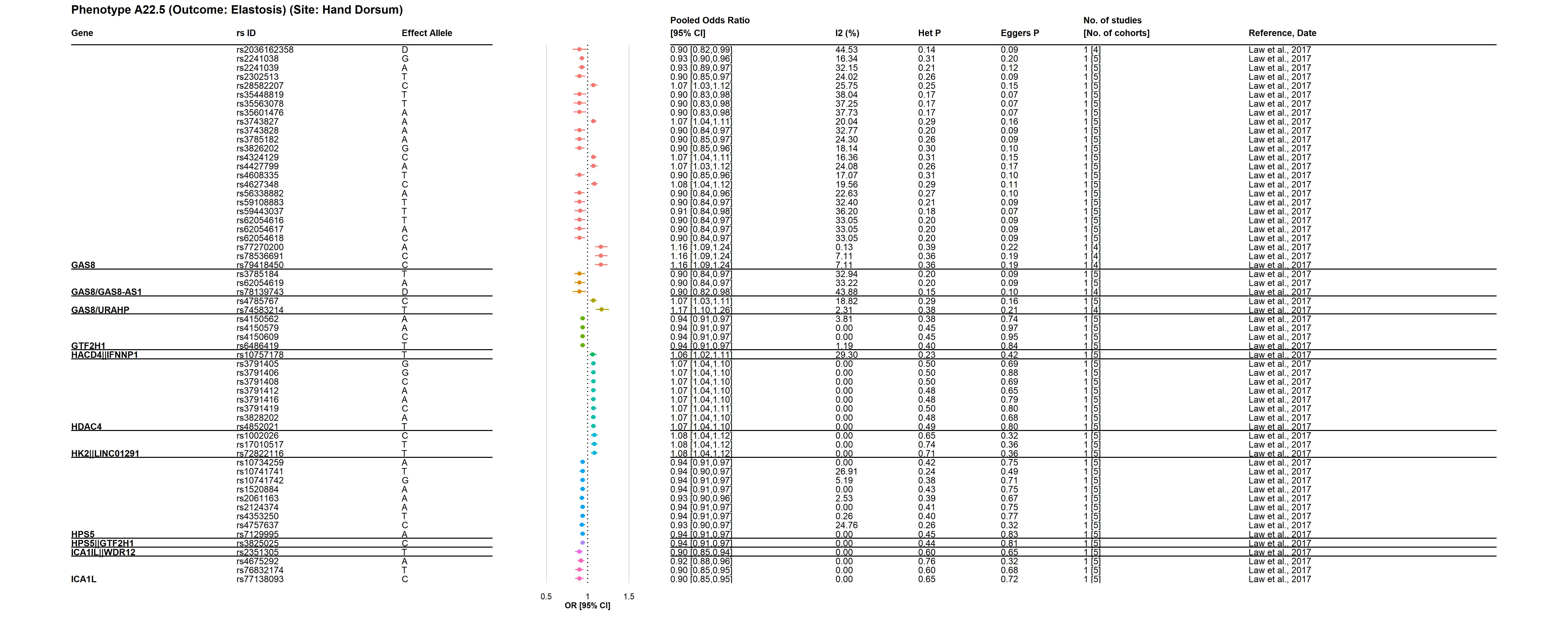

Supplement: Supplementary file 1 — Supplementary Information 1. [file 41598_2022_17443_MOESM1_ESM.zip › Supplementary Datasets/Dataset S1 - SNP-Phenotype Associations with Discovery and Validation Cohorts/1 study 2 cohorts Phenotype A22.5 (Outcome_Elastosis) (Site_Hand Dorsum).jpg]

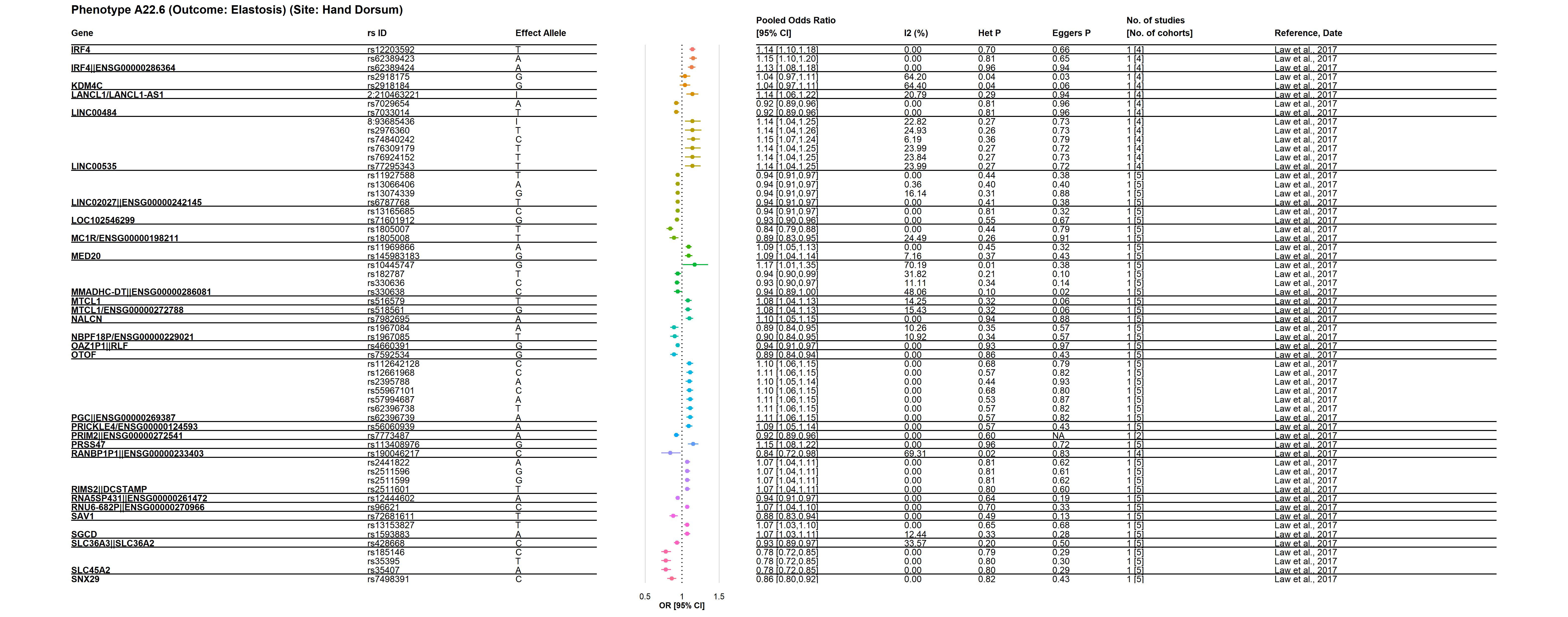

Supplement: Supplementary file 1 — Supplementary Information 1. [file 41598_2022_17443_MOESM1_ESM.zip › Supplementary Datasets/Dataset S1 - SNP-Phenotype Associations with Discovery and Validation Cohorts/1 study 2 cohorts Phenotype A22.6 (Outcome_Elastosis) (Site_Hand Dorsum).jpg]

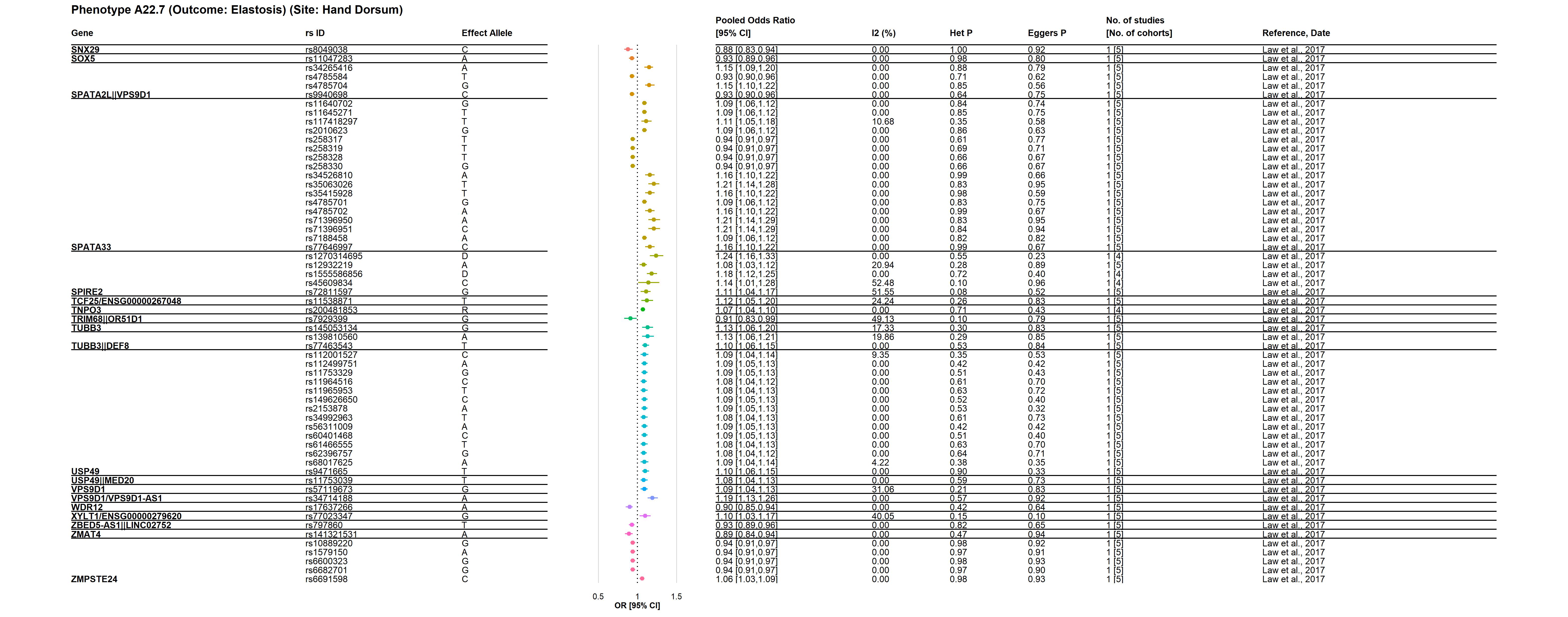

Supplement: Supplementary file 1 — Supplementary Information 1. [file 41598_2022_17443_MOESM1_ESM.zip › Supplementary Datasets/Dataset S1 - SNP-Phenotype Associations with Discovery and Validation Cohorts/1 study 2 cohorts Phenotype A22.7 (Outcome_Elastosis) (Site_Hand Dorsum).jpg]

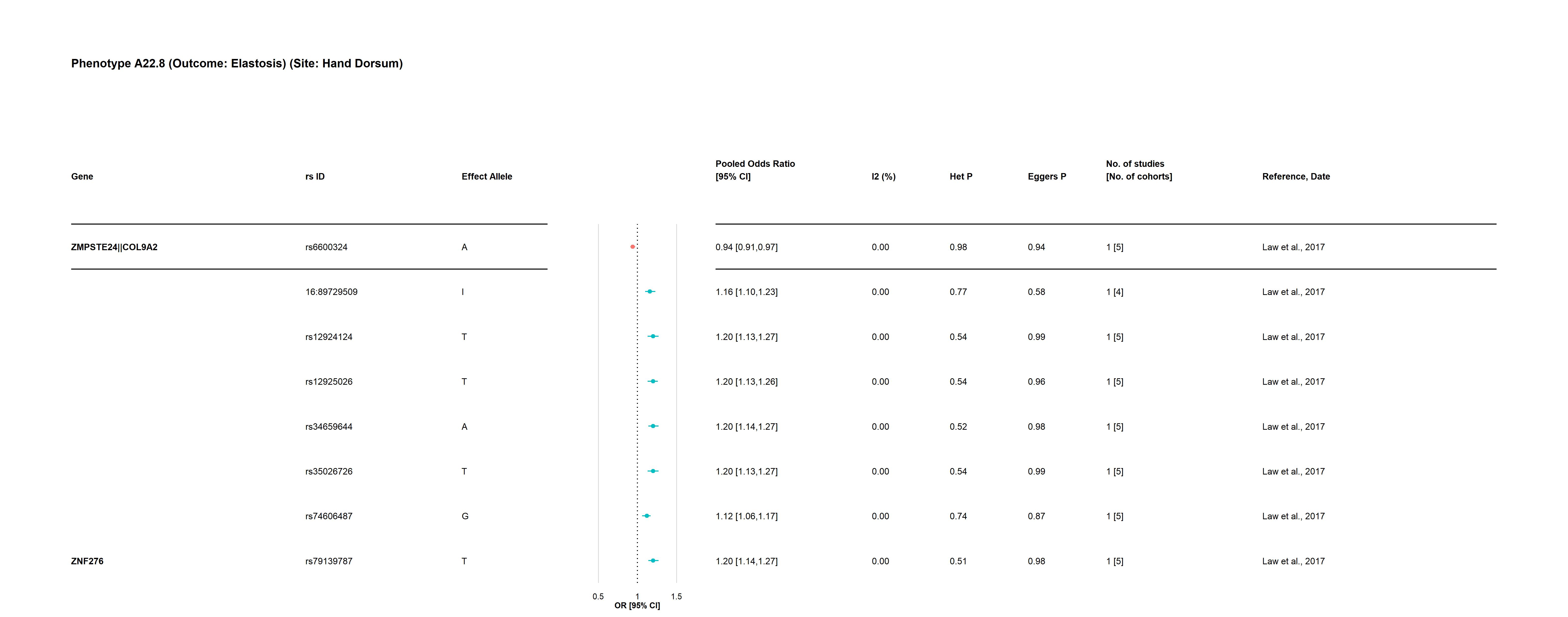

Supplement: Supplementary file 1 — Supplementary Information 1. [file 41598_2022_17443_MOESM1_ESM.zip › Supplementary Datasets/Dataset S1 - SNP-Phenotype Associations with Discovery and Validation Cohorts/1 study 2 cohorts Phenotype A22.8 (Outcome_Elastosis) (Site_Hand Dorsum).jpg]

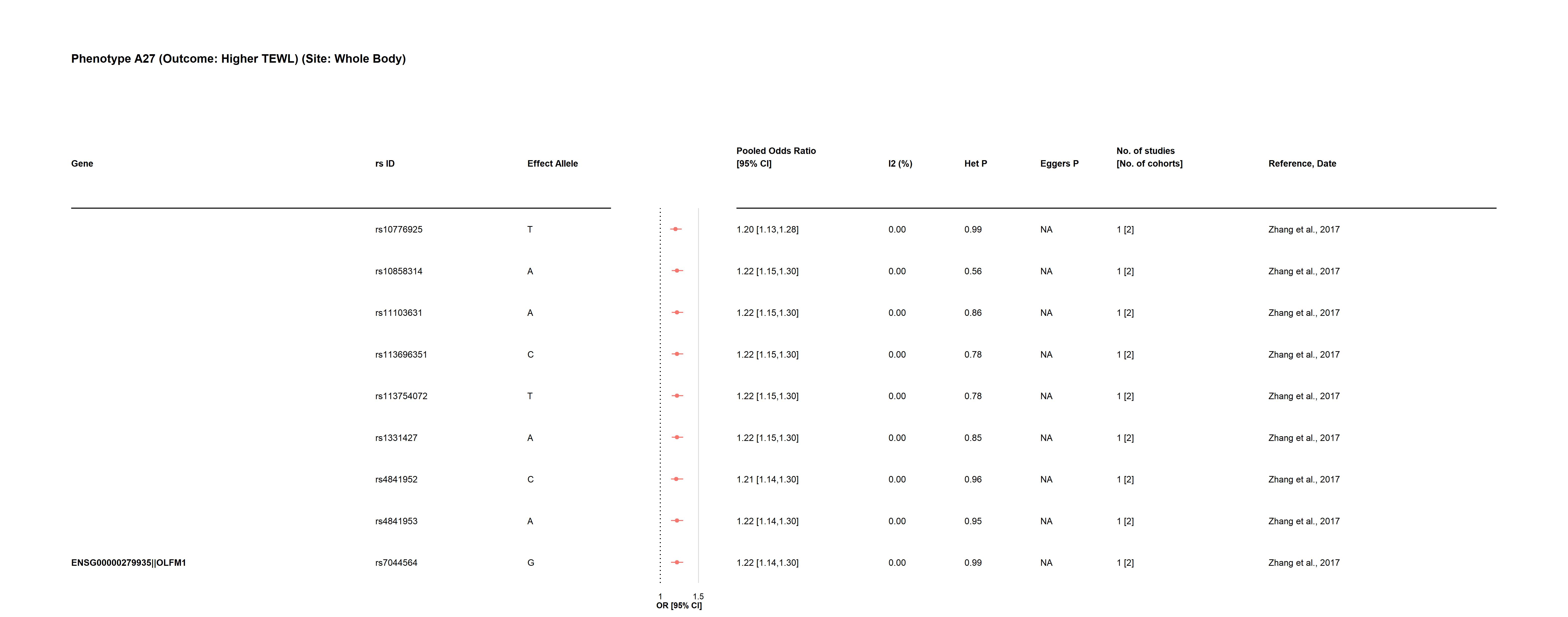

Supplement: Supplementary file 1 — Supplementary Information 1. [file 41598_2022_17443_MOESM1_ESM.zip › Supplementary Datasets/Dataset S1 - SNP-Phenotype Associations with Discovery and Validation Cohorts/1 study 2 cohorts Phenotype A27 (Outcome_Higher TEWL) (Site_Whole Body).jpg]

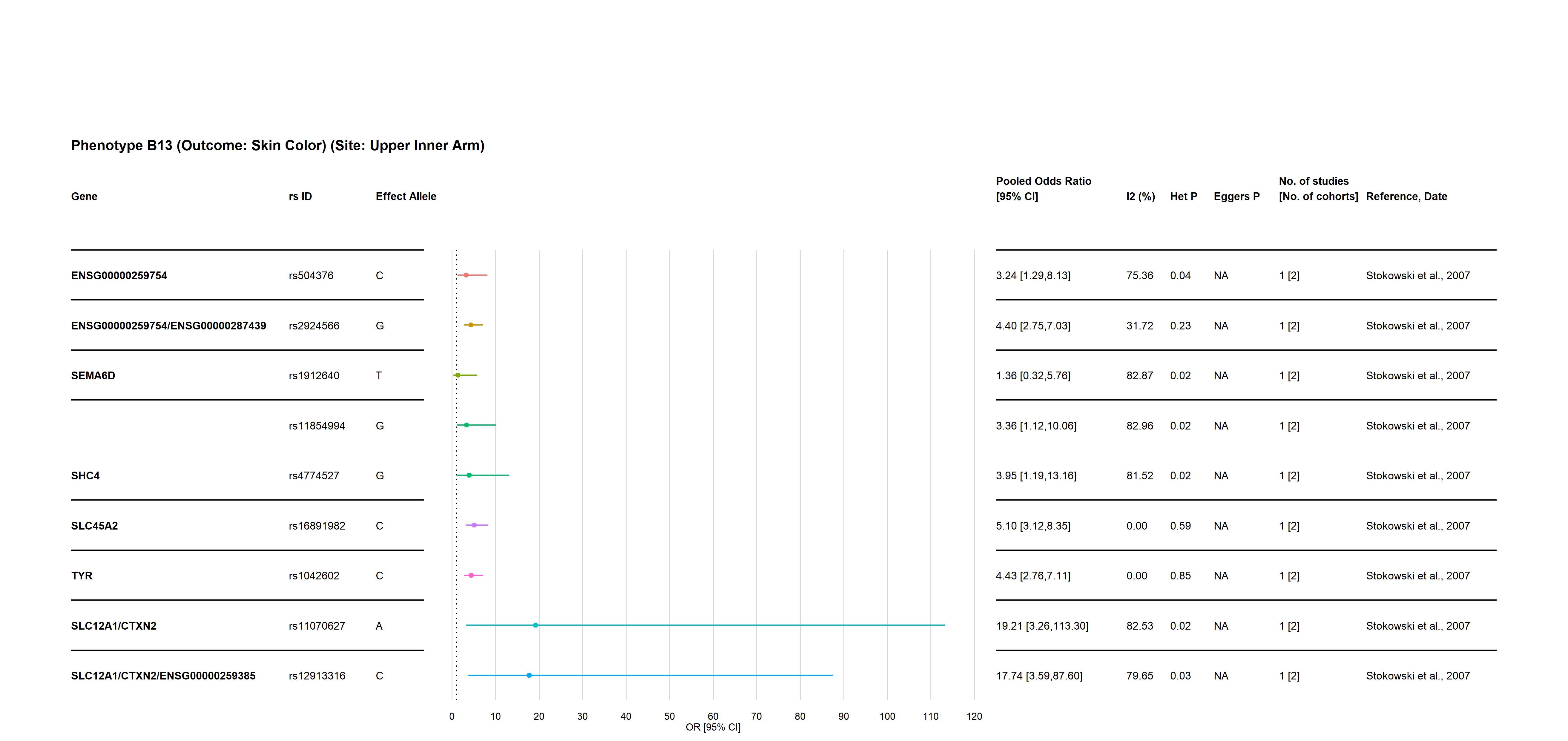

Supplement: Supplementary file 1 — Supplementary Information 1. [file 41598_2022_17443_MOESM1_ESM.zip › Supplementary Datasets/Dataset S1 - SNP-Phenotype Associations with Discovery and Validation Cohorts/1 study 2 cohorts Phenotype B13 (Outcome_Skin Color) (Site_Upper Inner Arm).jpg]

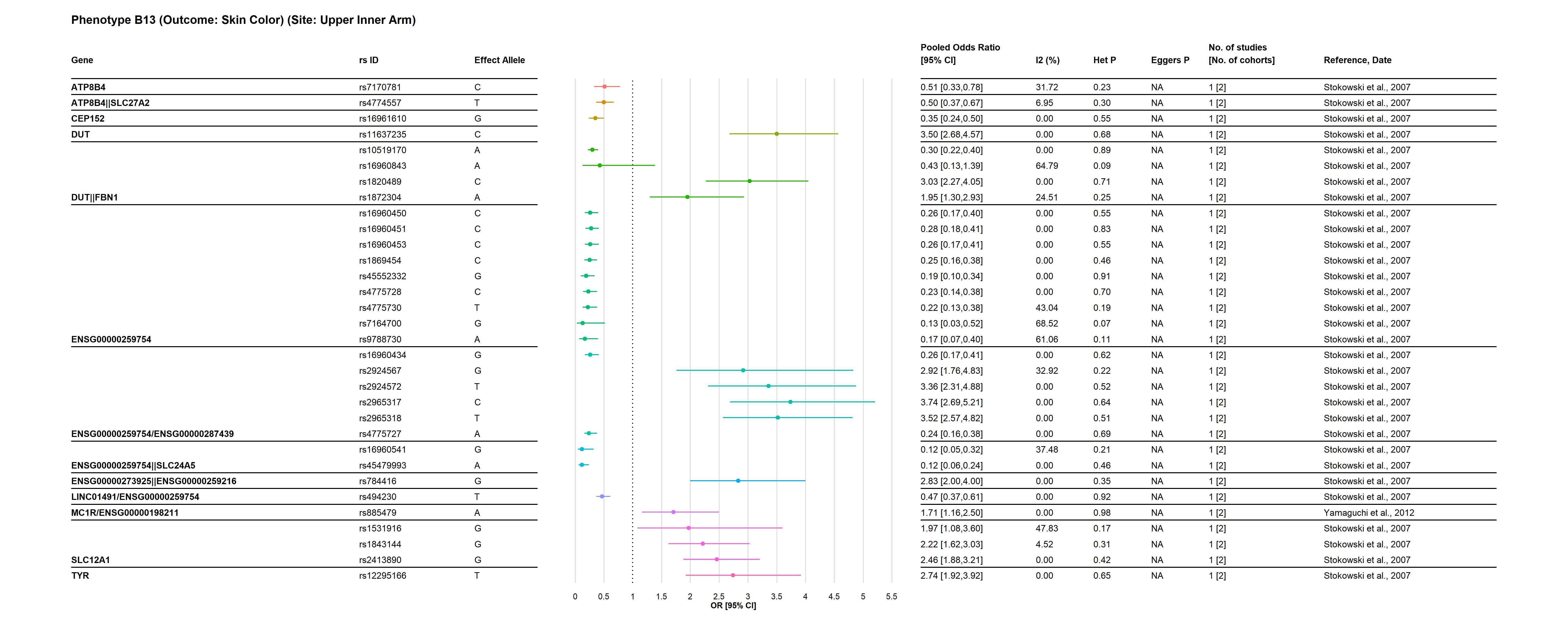

Supplement: Supplementary file 1 — Supplementary Information 1. [file 41598_2022_17443_MOESM1_ESM.zip › Supplementary Datasets/Dataset S1 - SNP-Phenotype Associations with Discovery and Validation Cohorts/1 study 2 cohorts Phenotype B13.1 (Outcome_Skin Color) (Site_Upper Inner Arm).jpg]

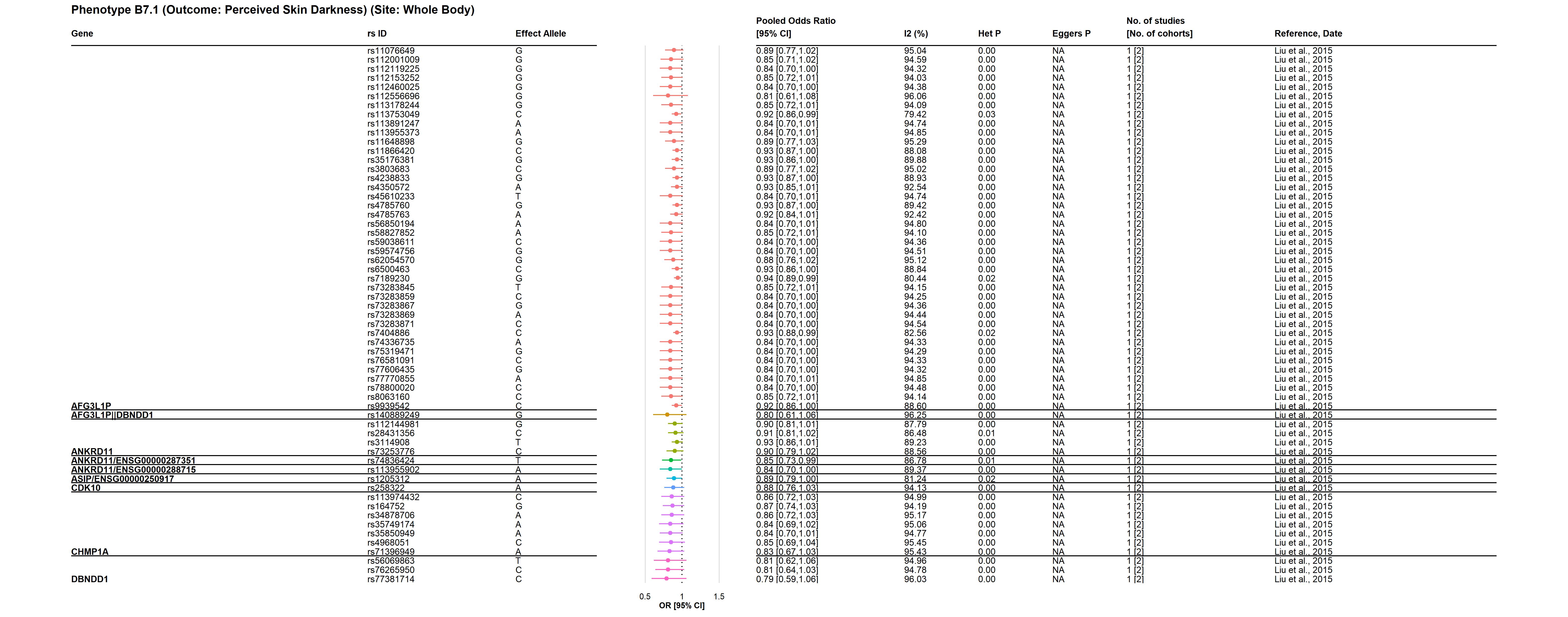

Supplement: Supplementary file 1 — Supplementary Information 1. [file 41598_2022_17443_MOESM1_ESM.zip › Supplementary Datasets/Dataset S1 - SNP-Phenotype Associations with Discovery and Validation Cohorts/1 study 2 cohorts Phenotype B7.1 (Outcome_Perceived Skin Darkness) (Site_Whole Body).jpg]

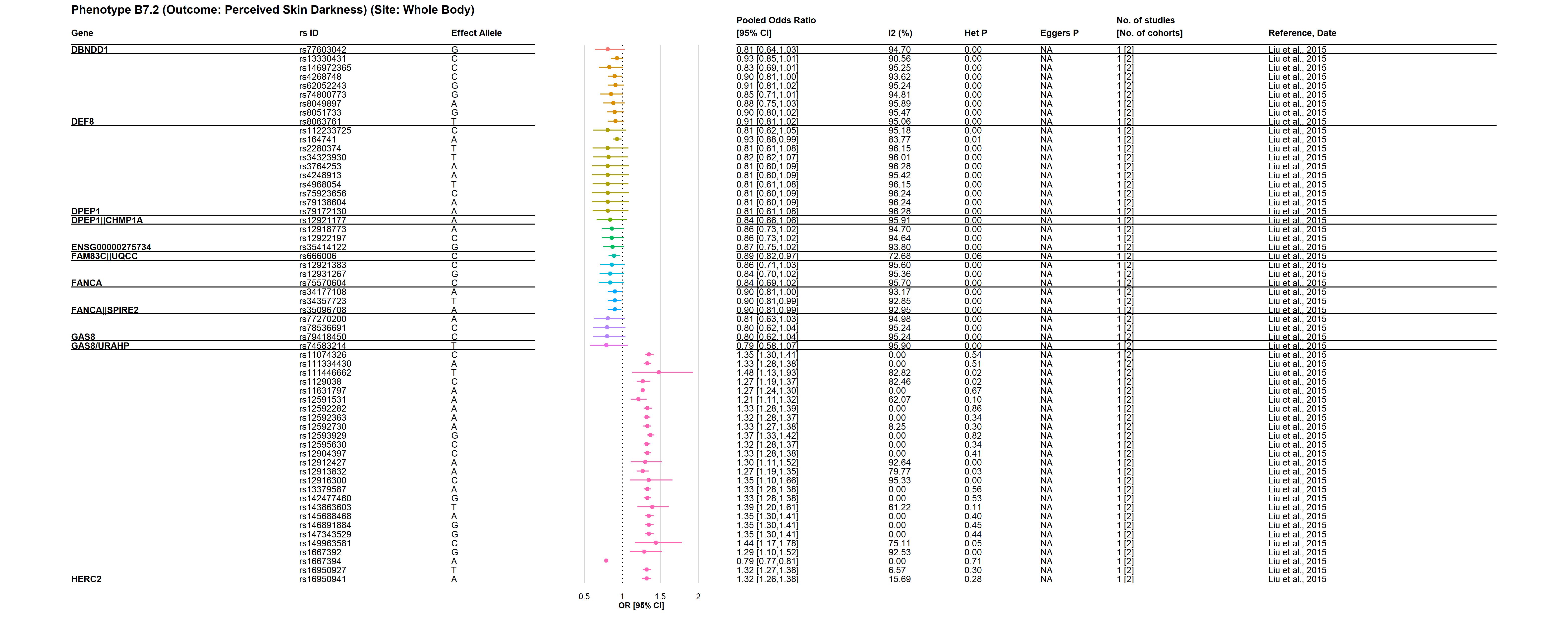

Supplement: Supplementary file 1 — Supplementary Information 1. [file 41598_2022_17443_MOESM1_ESM.zip › Supplementary Datasets/Dataset S1 - SNP-Phenotype Associations with Discovery and Validation Cohorts/1 study 2 cohorts Phenotype B7.2 (Outcome_Perceived Skin Darkness) (Site_Whole Body).jpg]

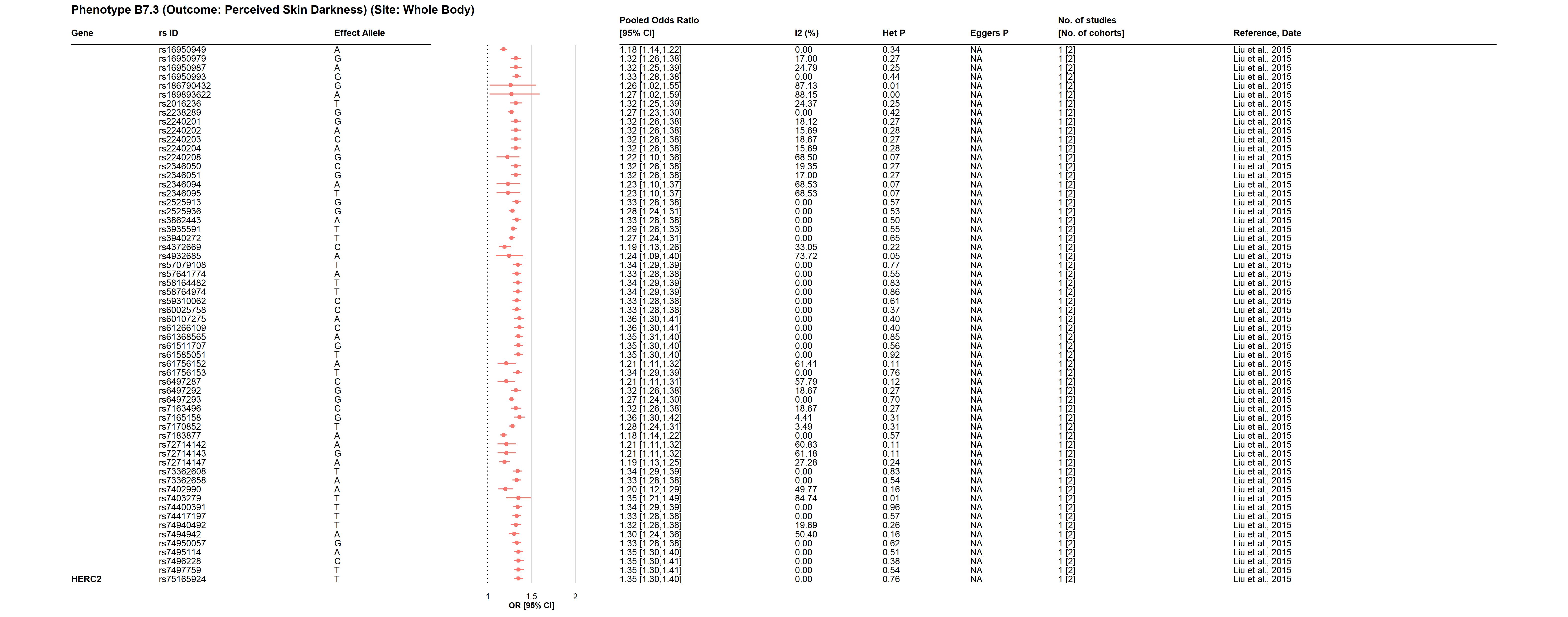

Supplement: Supplementary file 1 — Supplementary Information 1. [file 41598_2022_17443_MOESM1_ESM.zip › Supplementary Datasets/Dataset S1 - SNP-Phenotype Associations with Discovery and Validation Cohorts/1 study 2 cohorts Phenotype B7.3 (Outcome_Perceived Skin Darkness) (Site_Whole Body).jpg]

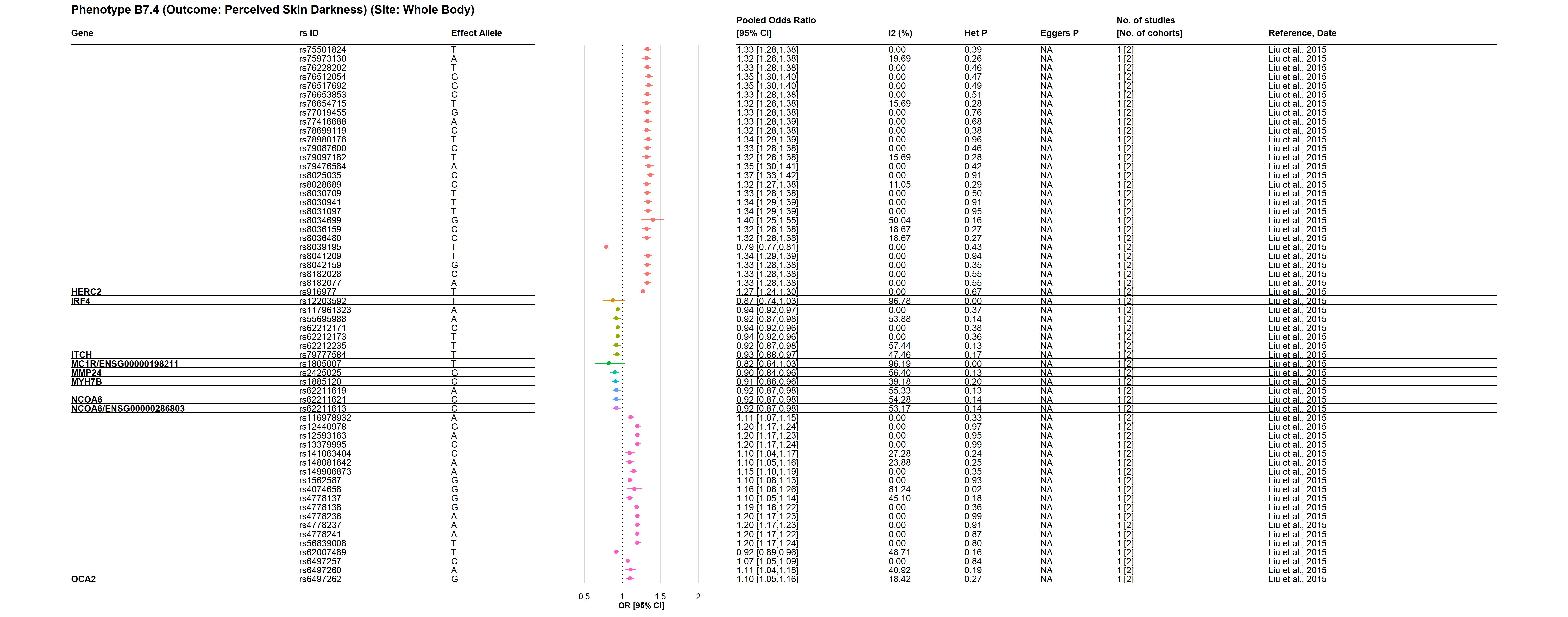

Supplement: Supplementary file 1 — Supplementary Information 1. [file 41598_2022_17443_MOESM1_ESM.zip › Supplementary Datasets/Dataset S1 - SNP-Phenotype Associations with Discovery and Validation Cohorts/1 study 2 cohorts Phenotype B7.4 (Outcome_Perceived Skin Darkness) (Site_Whole Body).jpg]

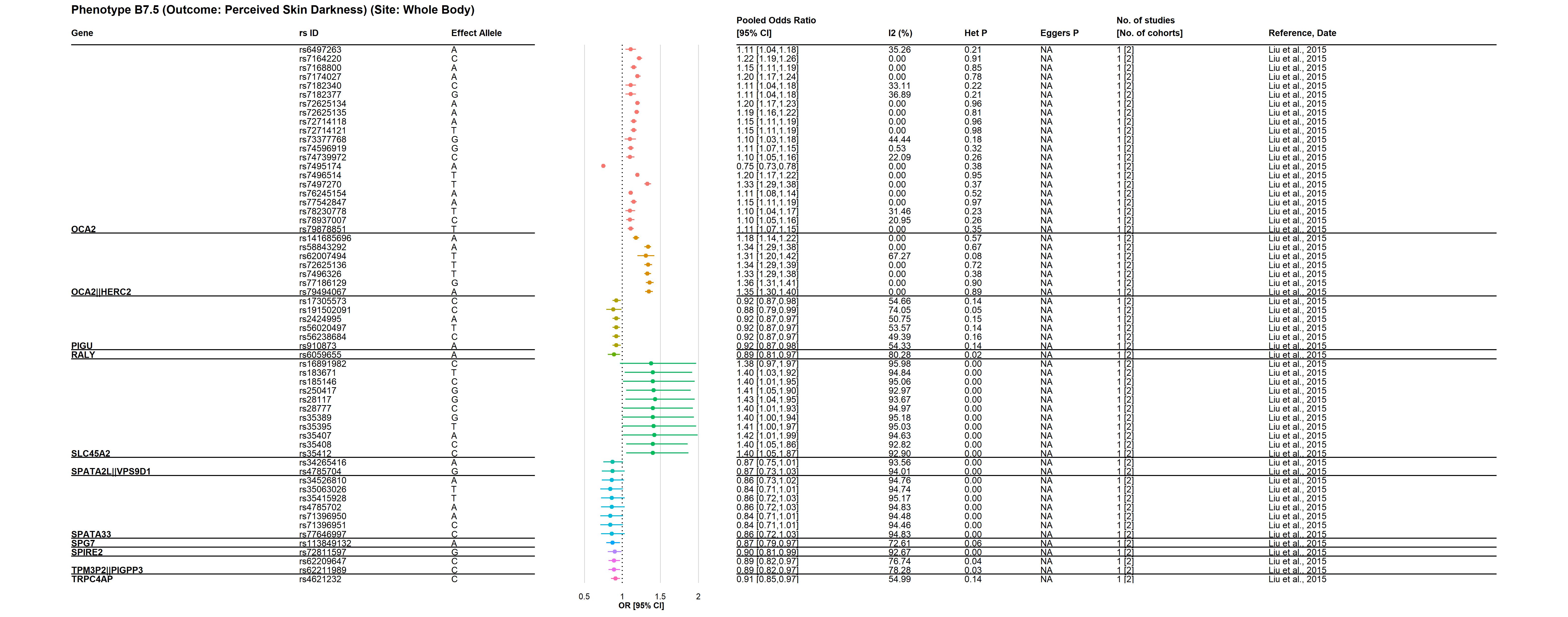

Supplement: Supplementary file 1 — Supplementary Information 1. [file 41598_2022_17443_MOESM1_ESM.zip › Supplementary Datasets/Dataset S1 - SNP-Phenotype Associations with Discovery and Validation Cohorts/1 study 2 cohorts Phenotype B7.5 (Outcome_Perceived Skin Darkness) (Site_Whole Body).jpg]

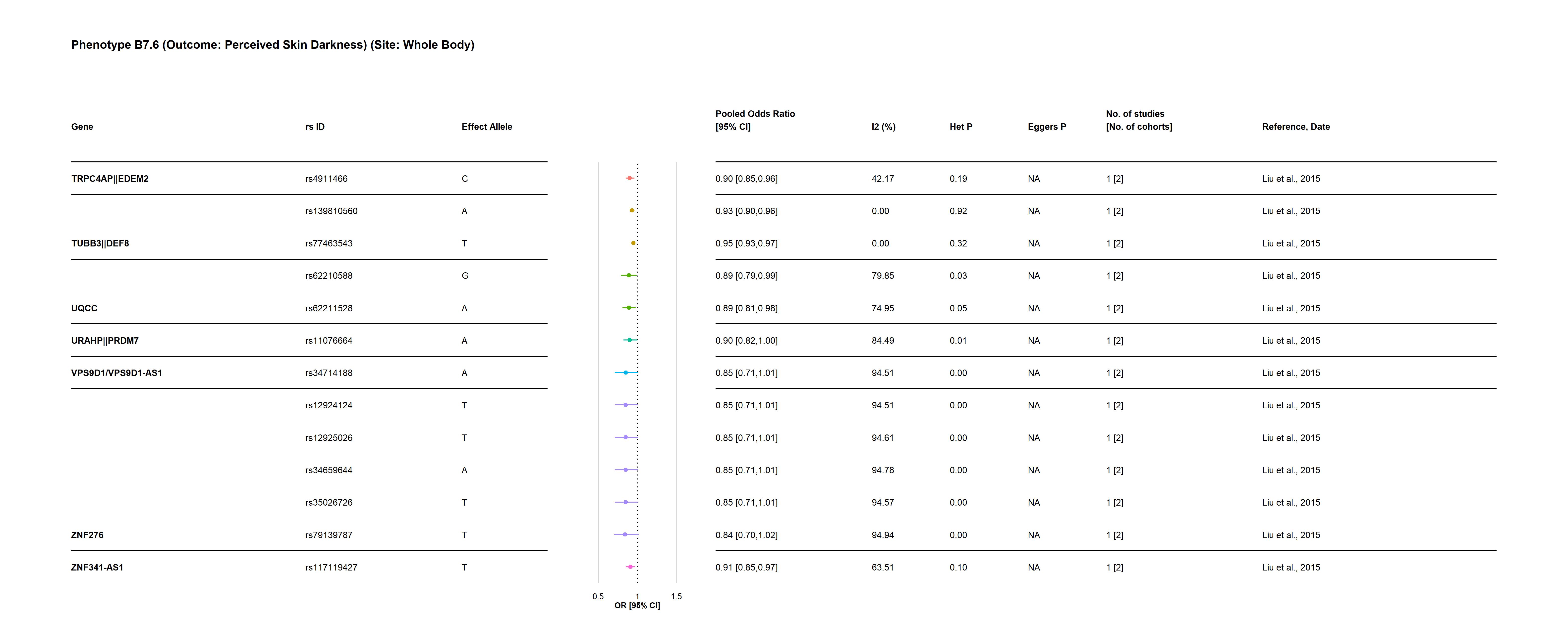

Supplement: Supplementary file 1 — Supplementary Information 1. [file 41598_2022_17443_MOESM1_ESM.zip › Supplementary Datasets/Dataset S1 - SNP-Phenotype Associations with Discovery and Validation Cohorts/1 study 2 cohorts Phenotype B7.6 (Outcome_Perceived Skin Darkness) (Site_Whole Body).jpg]

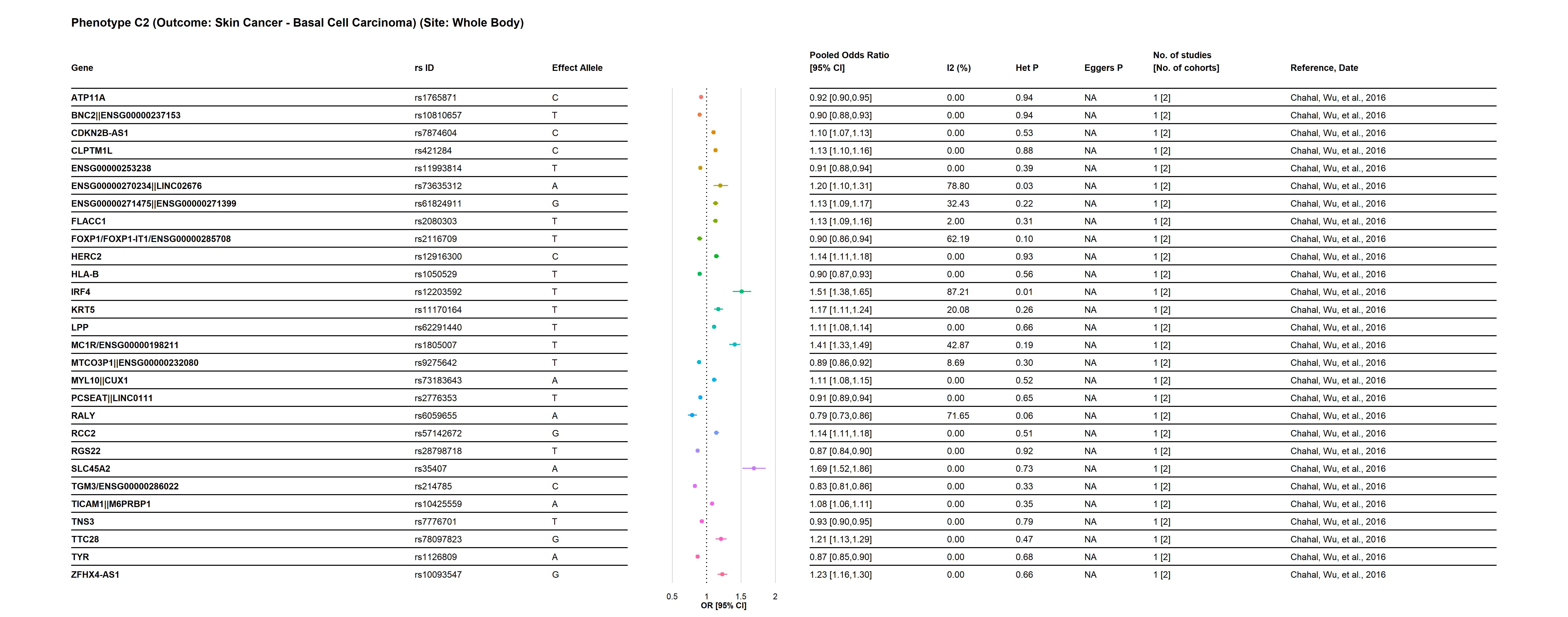

Supplement: Supplementary file 1 — Supplementary Information 1. [file 41598_2022_17443_MOESM1_ESM.zip › Supplementary Datasets/Dataset S1 - SNP-Phenotype Associations with Discovery and Validation Cohorts/1 study 2 cohorts Phenotype C2 (Outcome_Skin Cancer - Basal Cell Carcinoma) (Site_Whole Body).jpg]

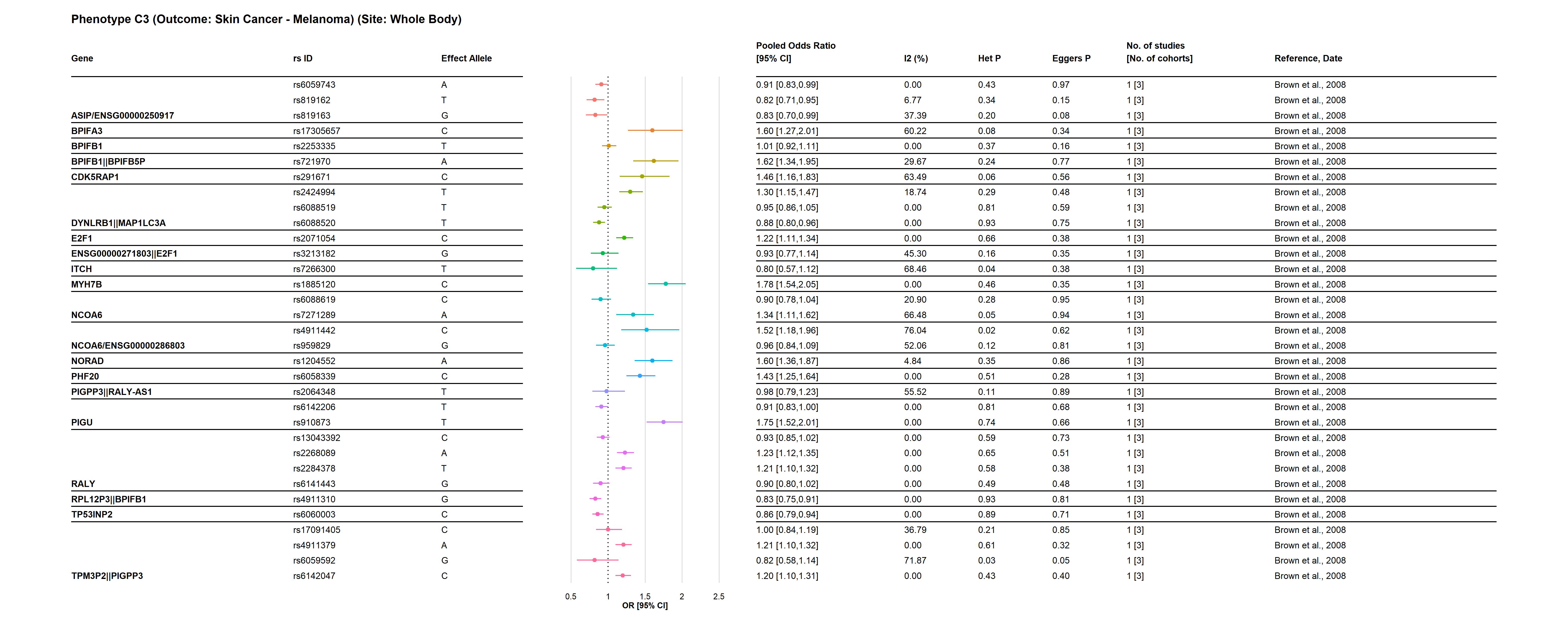

Supplement: Supplementary file 1 — Supplementary Information 1. [file 41598_2022_17443_MOESM1_ESM.zip › Supplementary Datasets/Dataset S1 - SNP-Phenotype Associations with Discovery and Validation Cohorts/1 study 2 cohorts Phenotype C3 (Outcome_Skin Cancer - Melanoma) (Site_Whole Body).jpg]

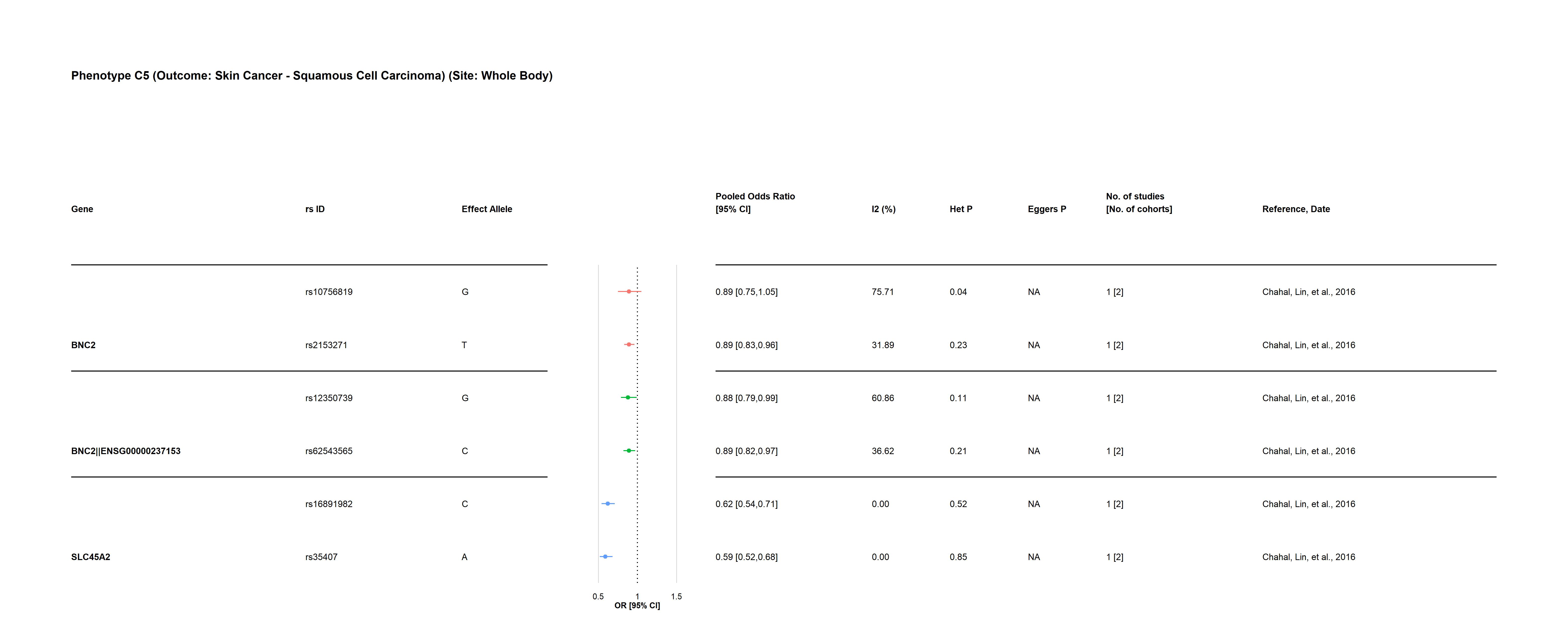

Supplement: Supplementary file 1 — Supplementary Information 1. [file 41598_2022_17443_MOESM1_ESM.zip › Supplementary Datasets/Dataset S1 - SNP-Phenotype Associations with Discovery and Validation Cohorts/1 study 2 cohorts Phenotype C5 (Outcome_Skin Cancer - Squamous Cell Carcinoma) (Site_Whole Body).jpg]

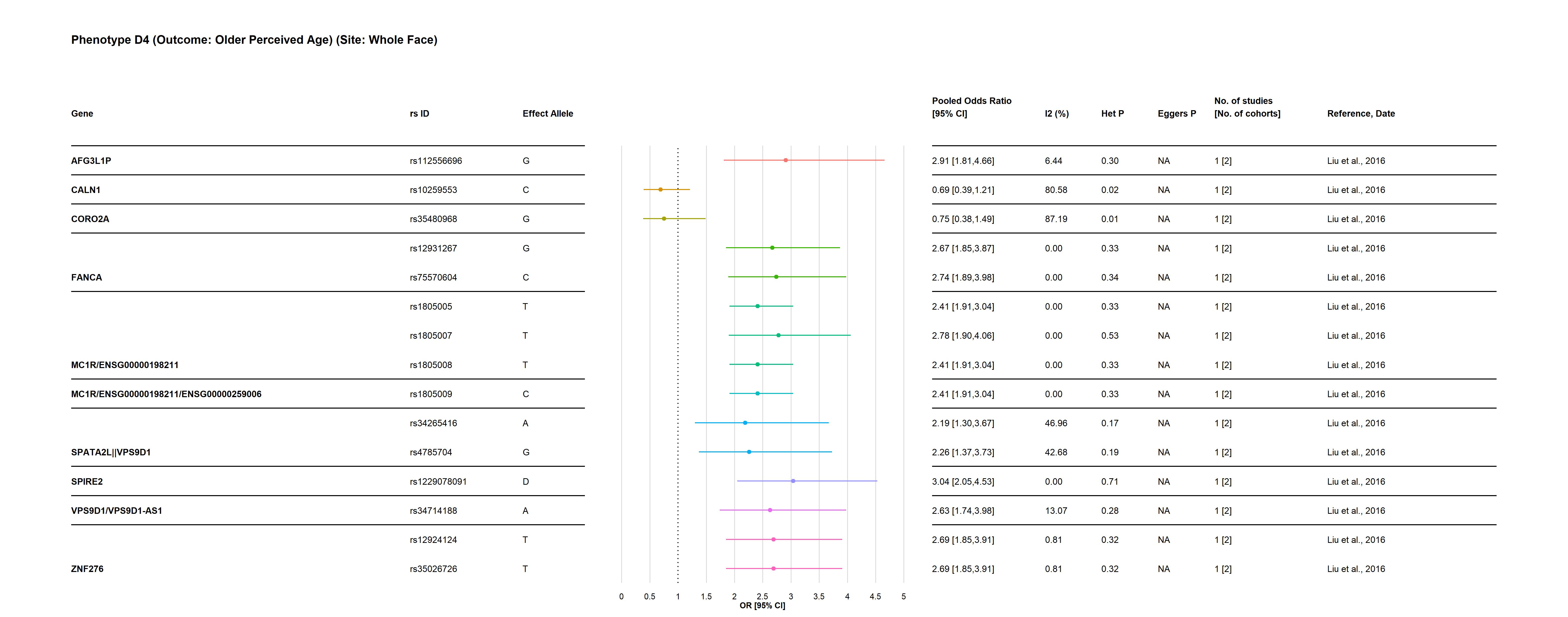

Supplement: Supplementary file 1 — Supplementary Information 1. [file 41598_2022_17443_MOESM1_ESM.zip › Supplementary Datasets/Dataset S1 - SNP-Phenotype Associations with Discovery and Validation Cohorts/1 study 2 cohorts Phenotype D4 (Outcome_Older Perceived Age) (Site_Whole Face).jpg]

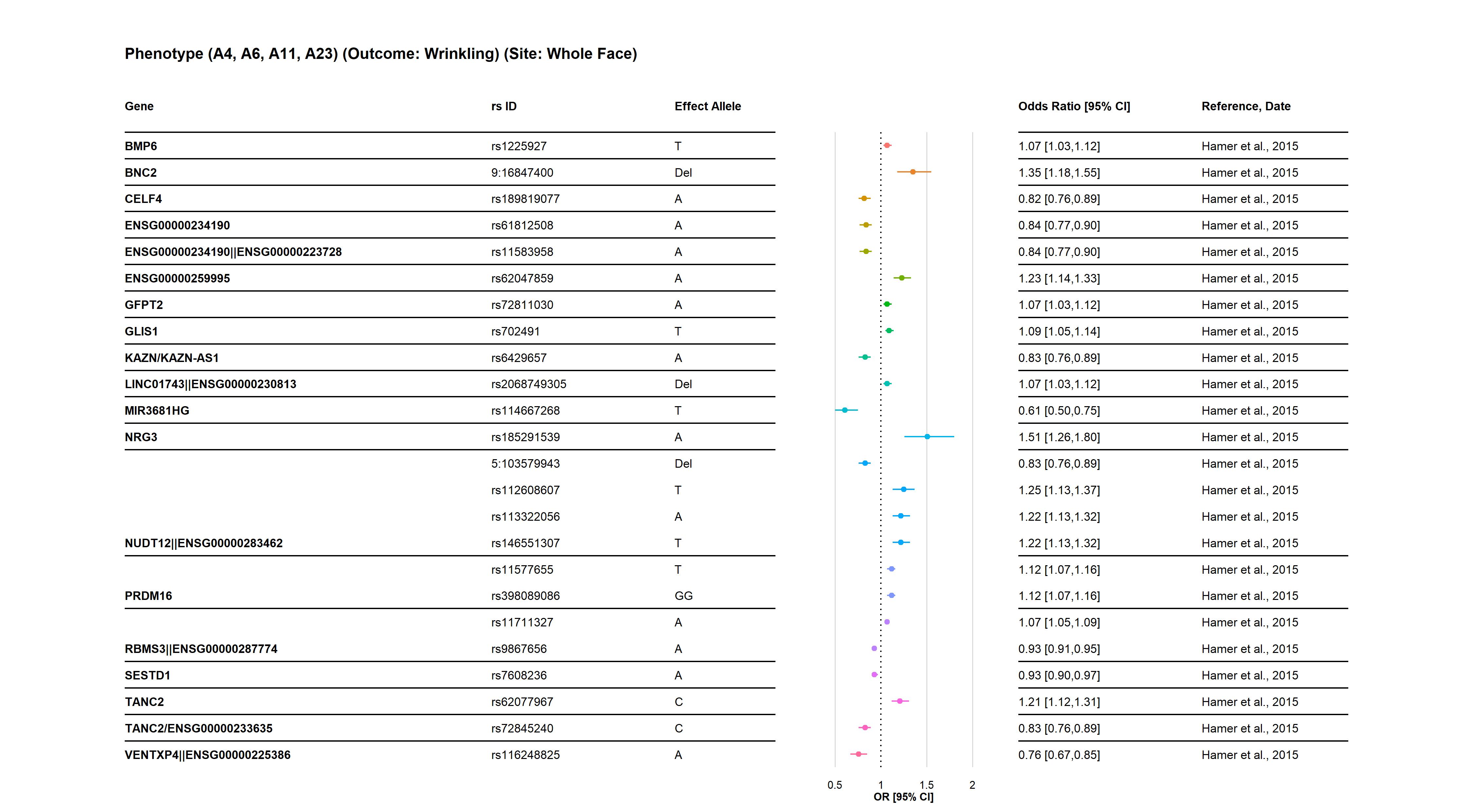

Supplement: Supplementary file 1 — Supplementary Information 1. [file 41598_2022_17443_MOESM1_ESM.zip › Supplementary Datasets/Dataset S2 - SNP-Phenotype Associations with 1 Study 1 Cohort/1 study 1 cohort Phenotype (A4, A6, A11, A23).1 (Outcome_Wrinkling) (Site_Whole Face).jpg]

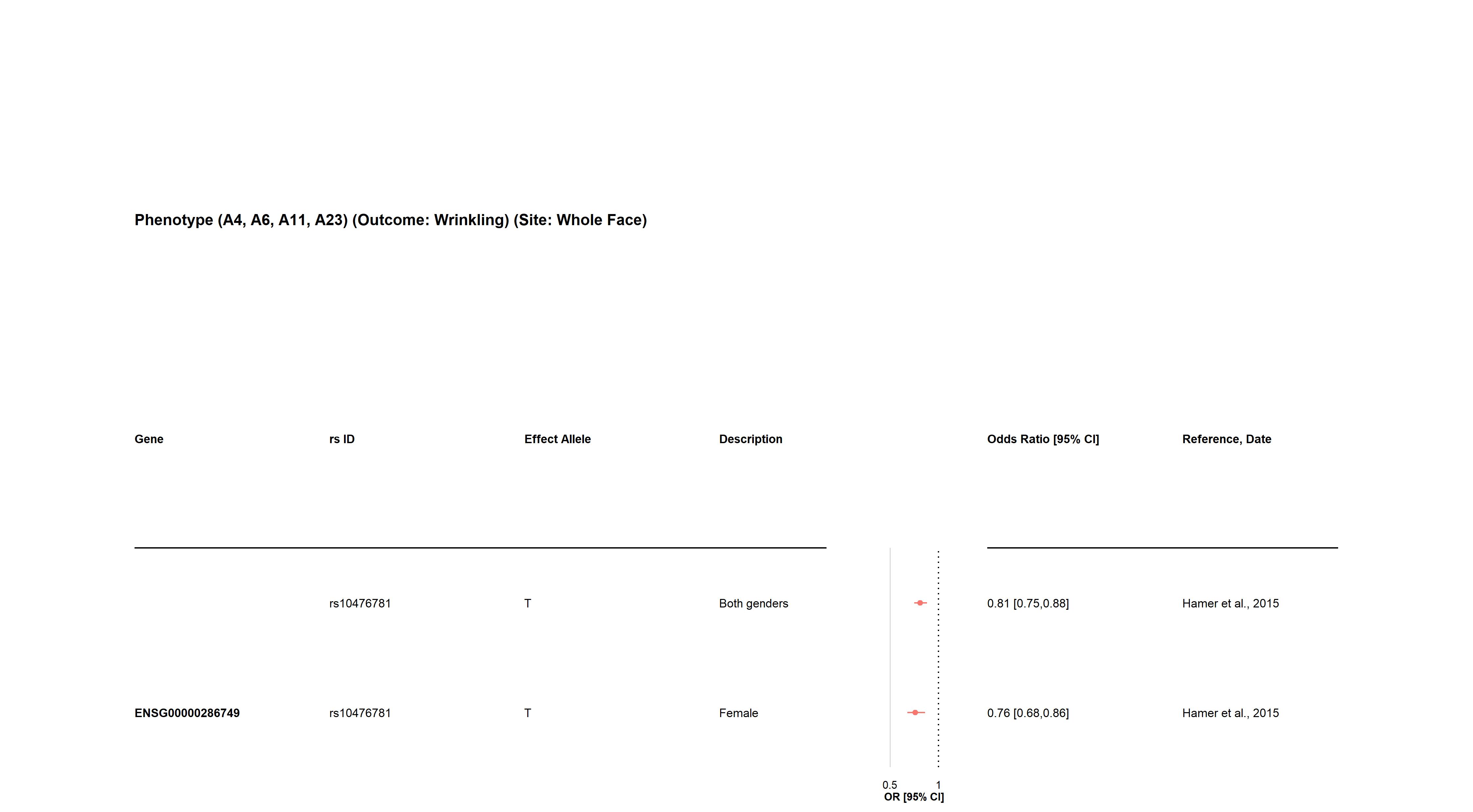

Supplement: Supplementary file 1 — Supplementary Information 1. [file 41598_2022_17443_MOESM1_ESM.zip › Supplementary Datasets/Dataset S2 - SNP-Phenotype Associations with 1 Study 1 Cohort/1 study 1 cohort Phenotype (A4, A6, A11, A23).2 (Outcome_Wrinkling) (Site_Whole Face).jpg]

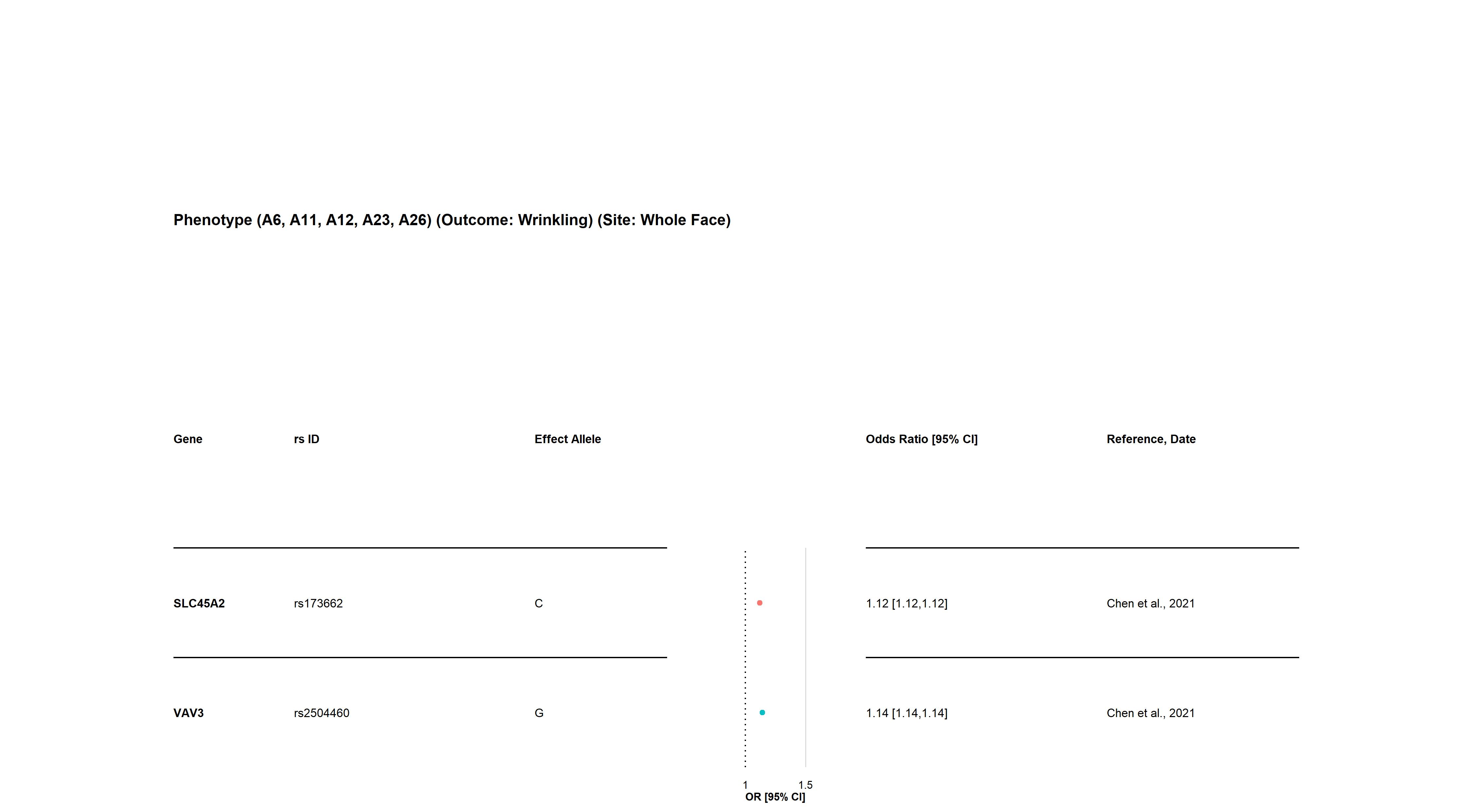

Supplement: Supplementary file 1 — Supplementary Information 1. [file 41598_2022_17443_MOESM1_ESM.zip › Supplementary Datasets/Dataset S2 - SNP-Phenotype Associations with 1 Study 1 Cohort/1 study 1 cohort Phenotype (A6, A11, A12, A23, A26) (Outcome_Wrinkling) (Site_Whole Face).jpg]

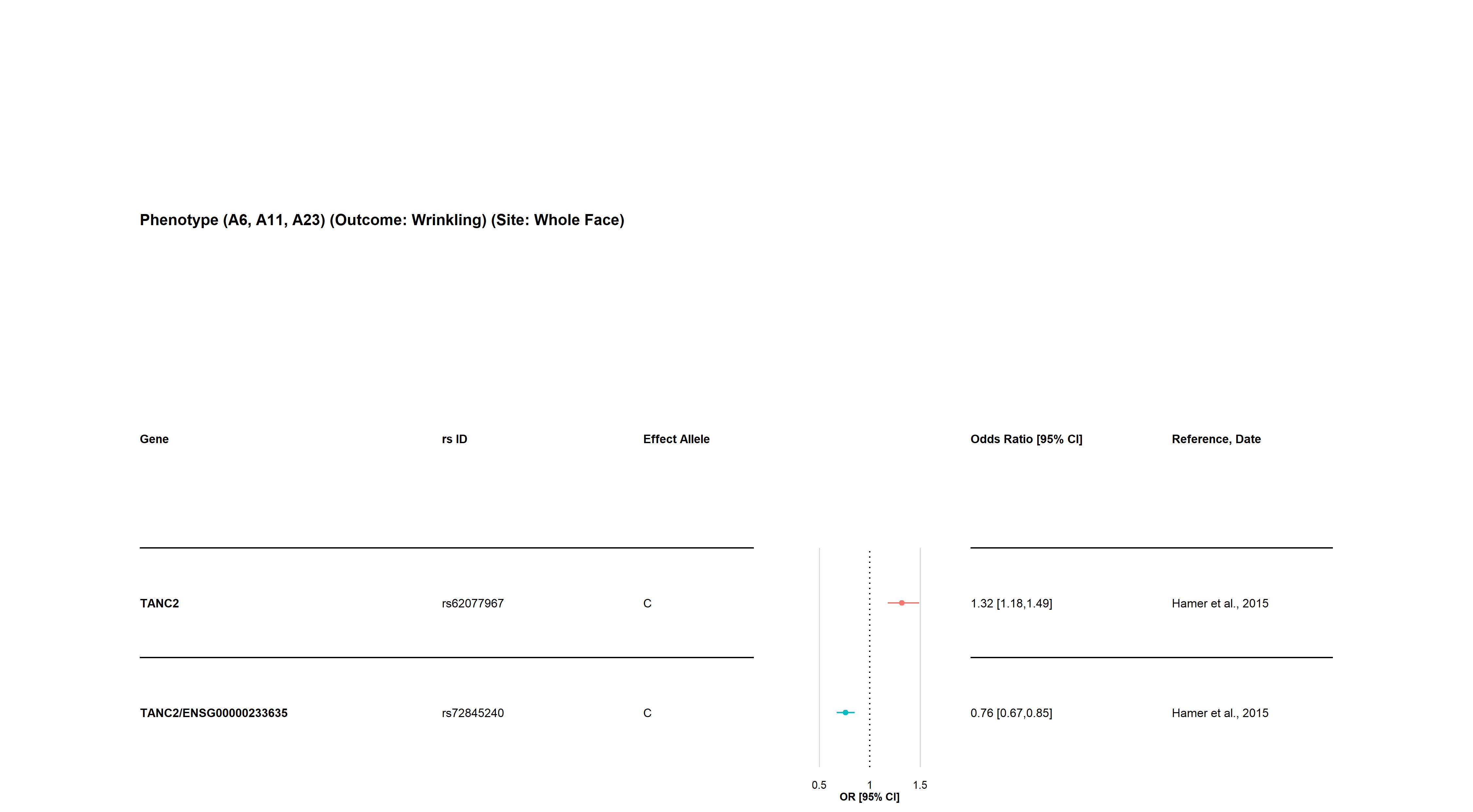

Supplement: Supplementary file 1 — Supplementary Information 1. [file 41598_2022_17443_MOESM1_ESM.zip › Supplementary Datasets/Dataset S2 - SNP-Phenotype Associations with 1 Study 1 Cohort/1 study 1 cohort Phenotype (A6, A11, A23) (Outcome_Wrinkling) (Site_Whole Face).jpg]

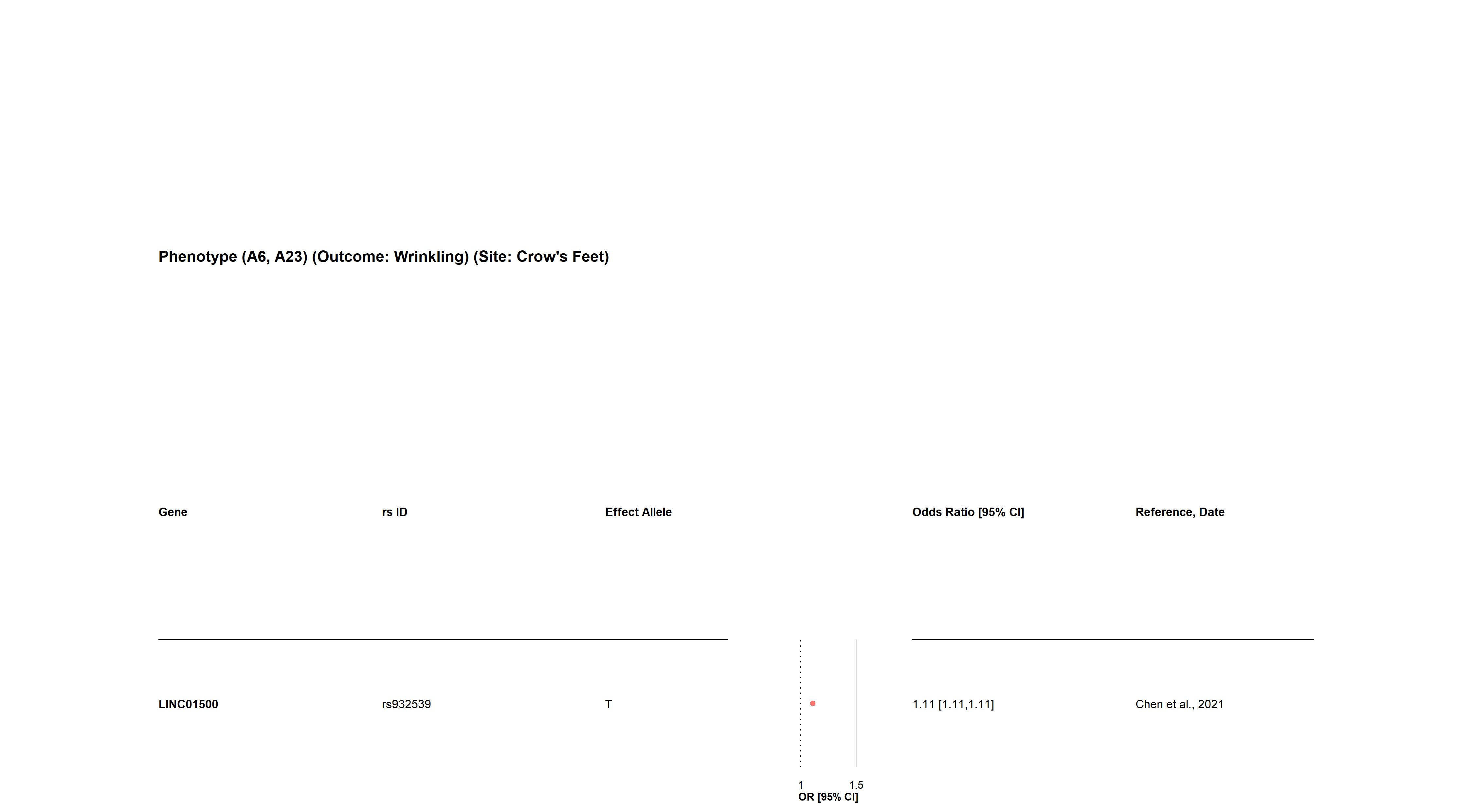

Supplement: Supplementary file 1 — Supplementary Information 1. [file 41598_2022_17443_MOESM1_ESM.zip › Supplementary Datasets/Dataset S2 - SNP-Phenotype Associations with 1 Study 1 Cohort/1 study 1 cohort Phenotype (A6, A23) (Outcome_Wrinkling) (Site_Crow_s Feet).jpg]

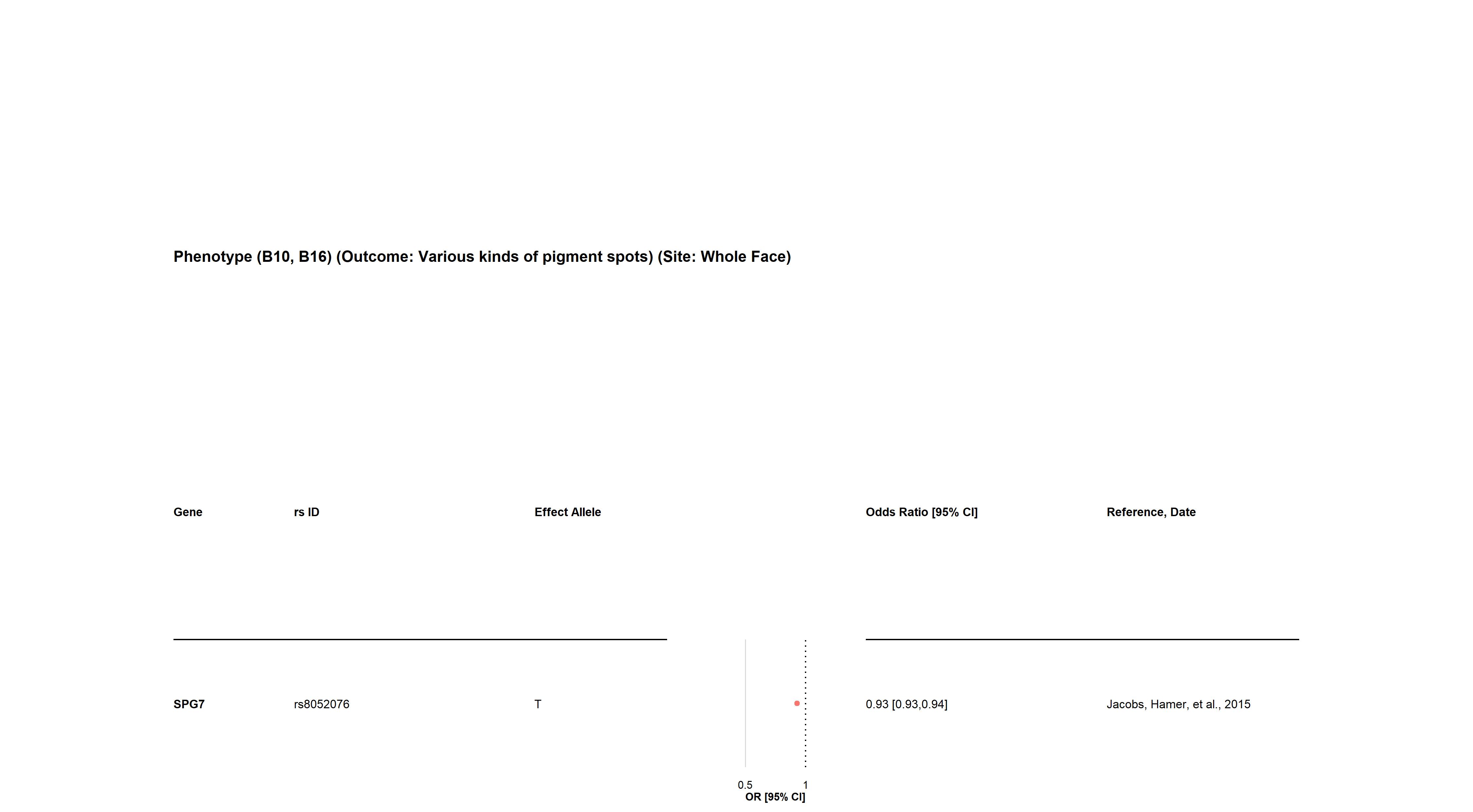

Supplement: Supplementary file 1 — Supplementary Information 1. [file 41598_2022_17443_MOESM1_ESM.zip › Supplementary Datasets/Dataset S2 - SNP-Phenotype Associations with 1 Study 1 Cohort/1 study 1 cohort Phenotype (B10, B16).1 (Outcome_Various kinds of pigment spots) (Site_Whole Face).jpg]

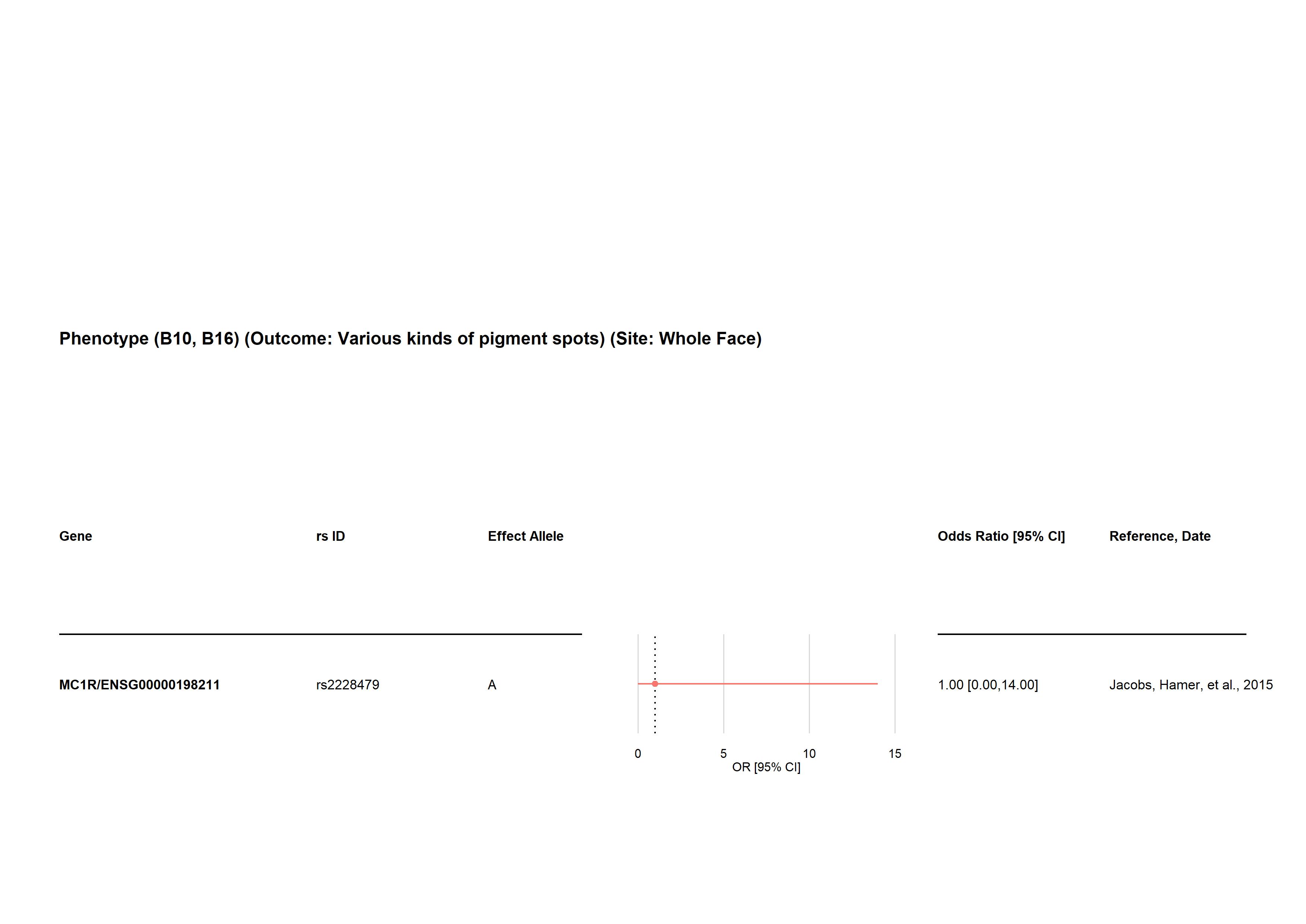

Supplement: Supplementary file 1 — Supplementary Information 1. [file 41598_2022_17443_MOESM1_ESM.zip › Supplementary Datasets/Dataset S2 - SNP-Phenotype Associations with 1 Study 1 Cohort/1 study 1 cohort Phenotype (B10, B16).2 (Outcome_Various kinds of pigment spots) (Site_Whole Face).jpg]

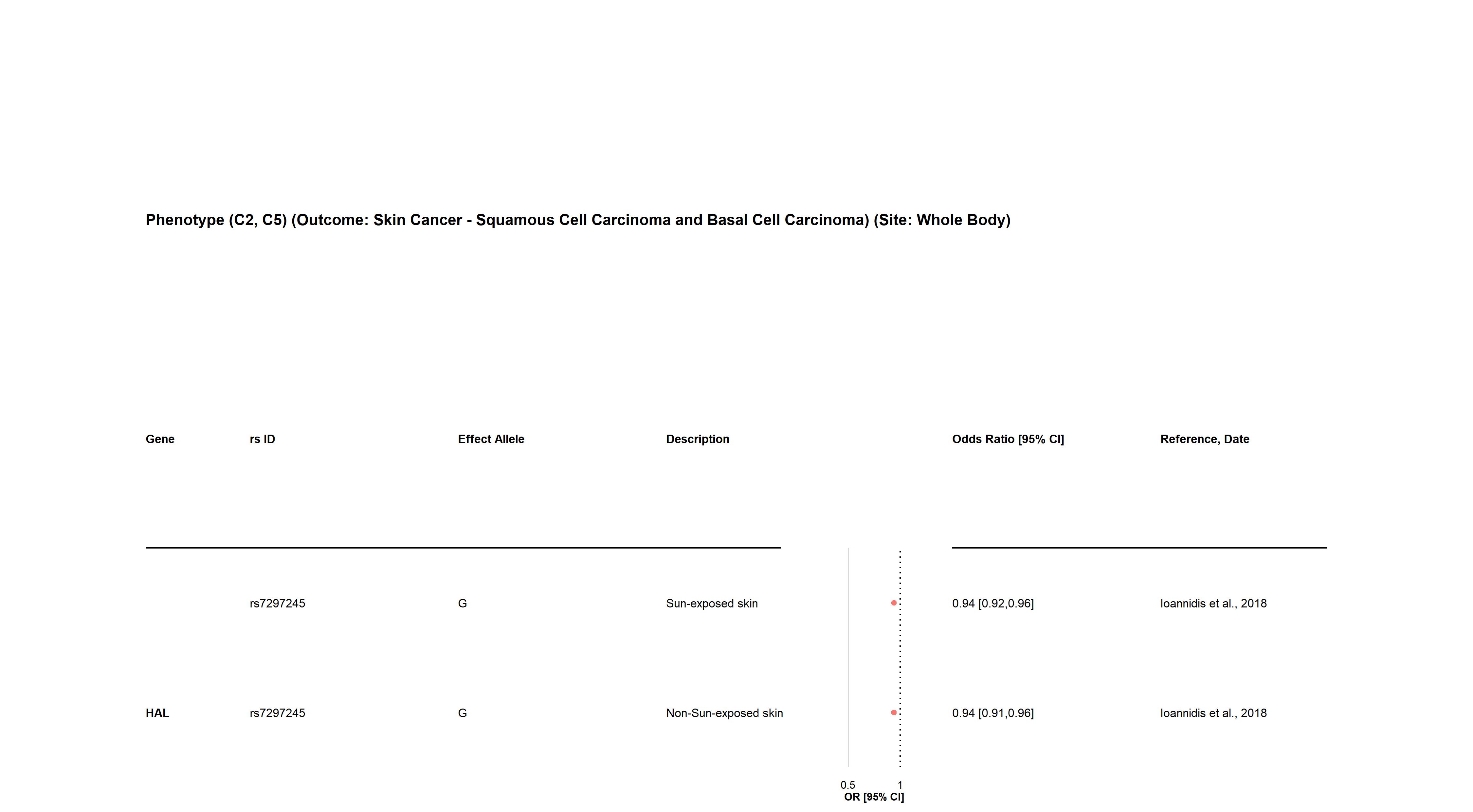

Supplement: Supplementary file 1 — Supplementary Information 1. [file 41598_2022_17443_MOESM1_ESM.zip › Supplementary Datasets/Dataset S2 - SNP-Phenotype Associations with 1 Study 1 Cohort/1 study 1 cohort Phenotype (C2, C5) (Outcome_Skin Cancer - Squamous Cell Carcinoma and Basal Cell Carcinoma) (Site_Whole Body).jpg]

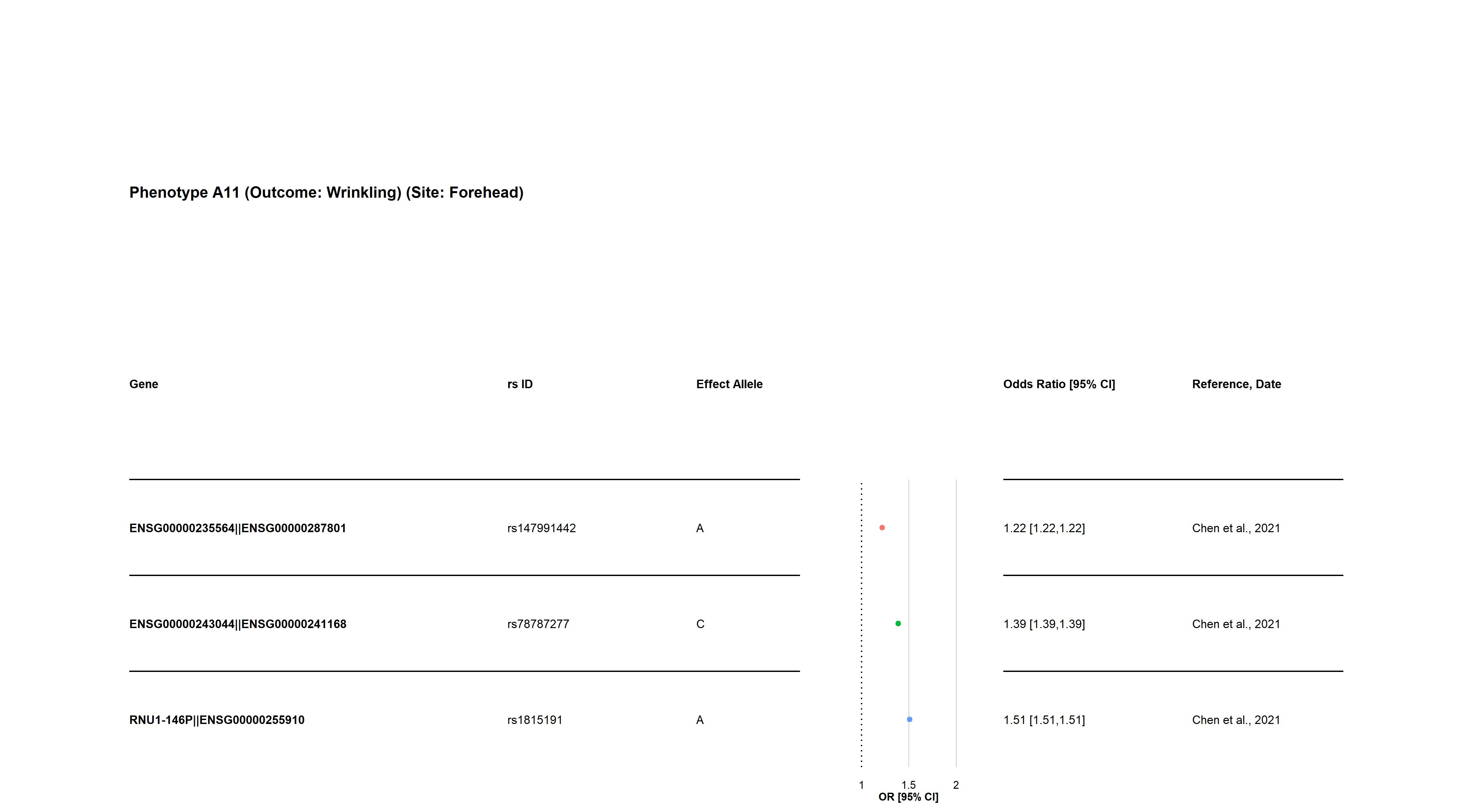

Supplement: Supplementary file 1 — Supplementary Information 1. [file 41598_2022_17443_MOESM1_ESM.zip › Supplementary Datasets/Dataset S2 - SNP-Phenotype Associations with 1 Study 1 Cohort/1 study 1 cohort Phenotype A11 (Outcome_Wrinkling) (Site_Forehead).jpg]

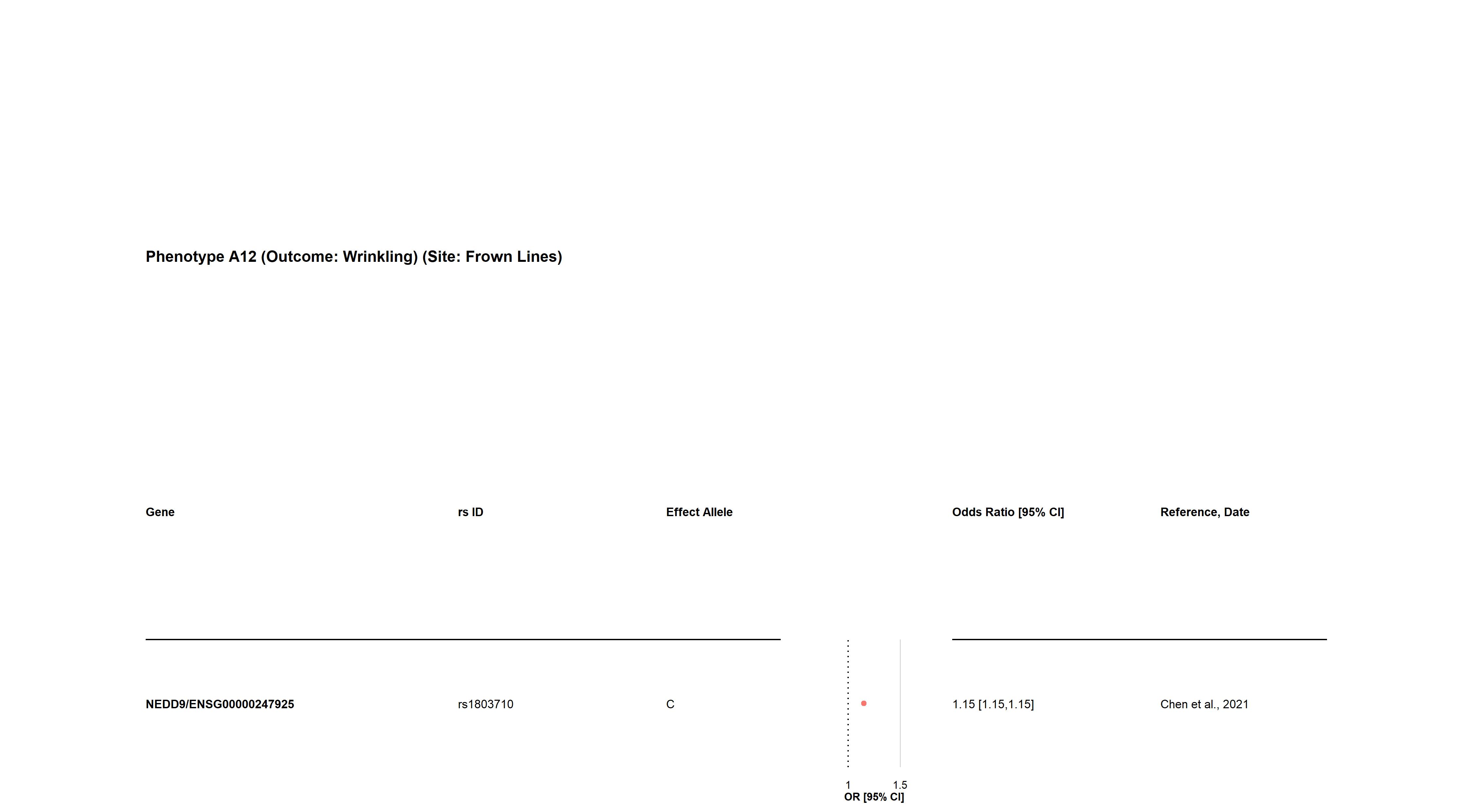

Supplement: Supplementary file 1 — Supplementary Information 1. [file 41598_2022_17443_MOESM1_ESM.zip › Supplementary Datasets/Dataset S2 - SNP-Phenotype Associations with 1 Study 1 Cohort/1 study 1 cohort Phenotype A12 (Outcome_Wrinkling) (Site_Frown Lines).jpg]

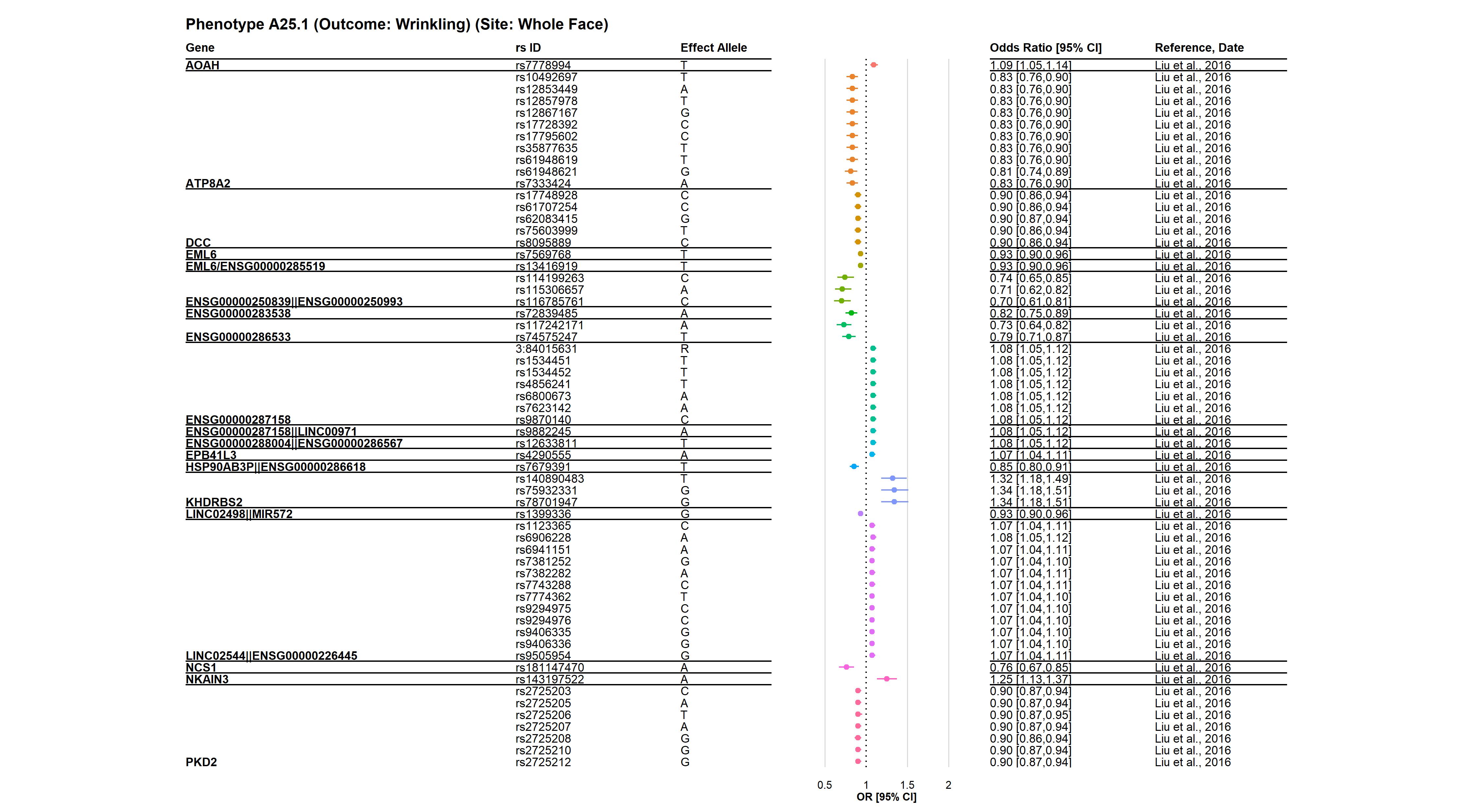

Supplement: Supplementary file 1 — Supplementary Information 1. [file 41598_2022_17443_MOESM1_ESM.zip › Supplementary Datasets/Dataset S2 - SNP-Phenotype Associations with 1 Study 1 Cohort/1 study 1 cohort Phenotype A25.1 (Outcome_Wrinkling) (Site_Whole Face).jpg]

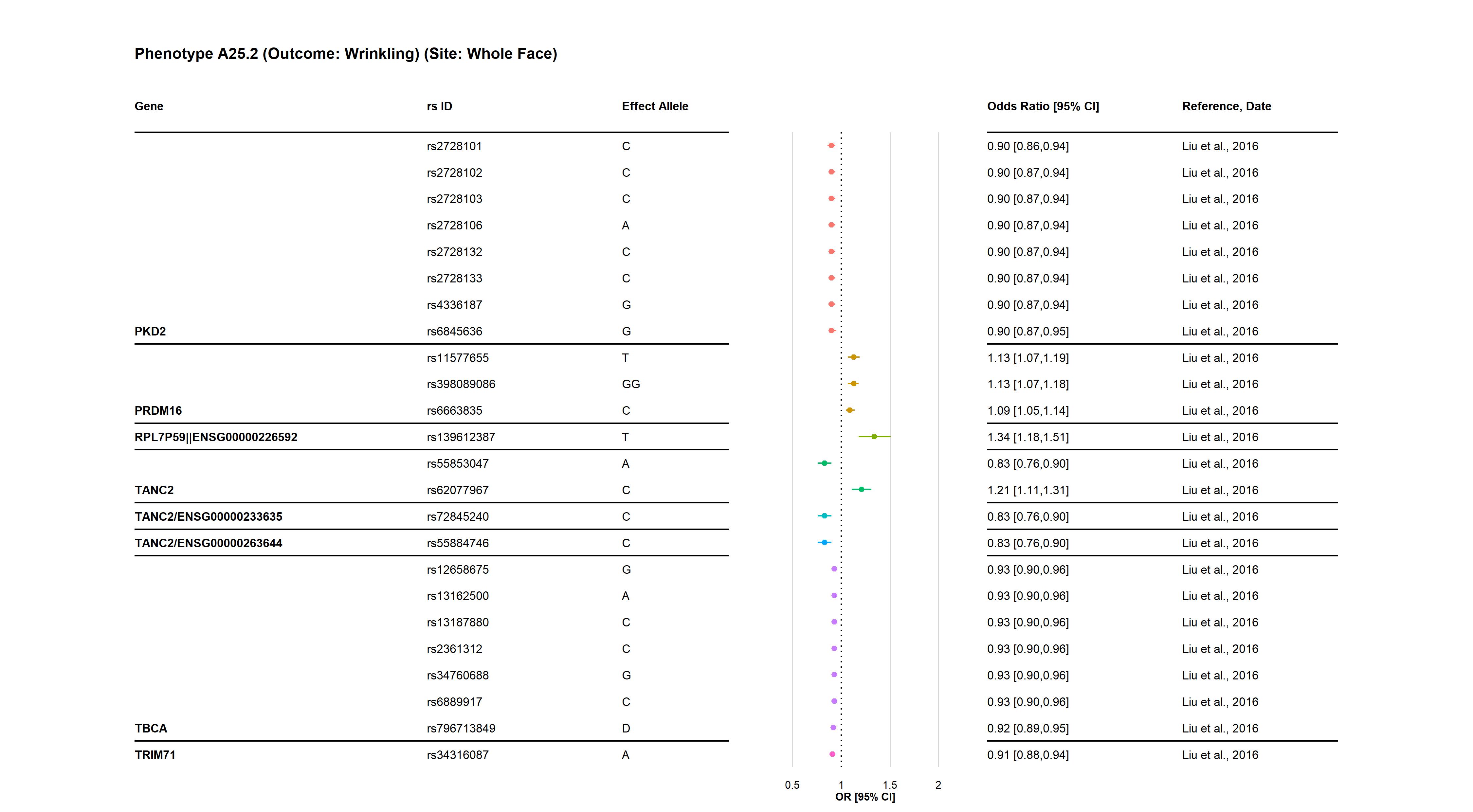

Supplement: Supplementary file 1 — Supplementary Information 1. [file 41598_2022_17443_MOESM1_ESM.zip › Supplementary Datasets/Dataset S2 - SNP-Phenotype Associations with 1 Study 1 Cohort/1 study 1 cohort Phenotype A25.2 (Outcome_Wrinkling) (Site_Whole Face).jpg]

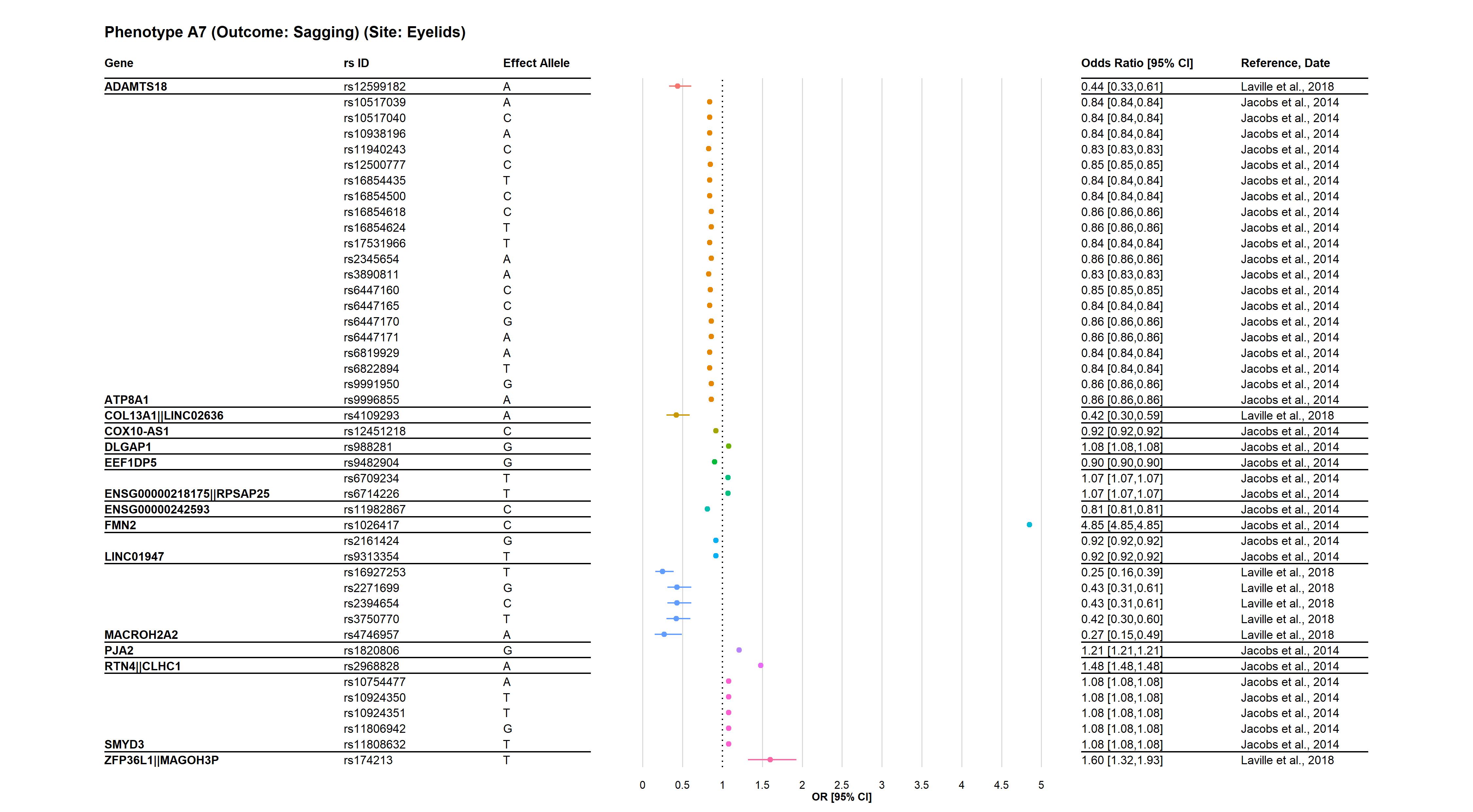

Supplement: Supplementary file 1 — Supplementary Information 1. [file 41598_2022_17443_MOESM1_ESM.zip › Supplementary Datasets/Dataset S2 - SNP-Phenotype Associations with 1 Study 1 Cohort/1 study 1 cohort Phenotype A7.1 (Outcome_Sagging) (Site_Eyelids).jpg]

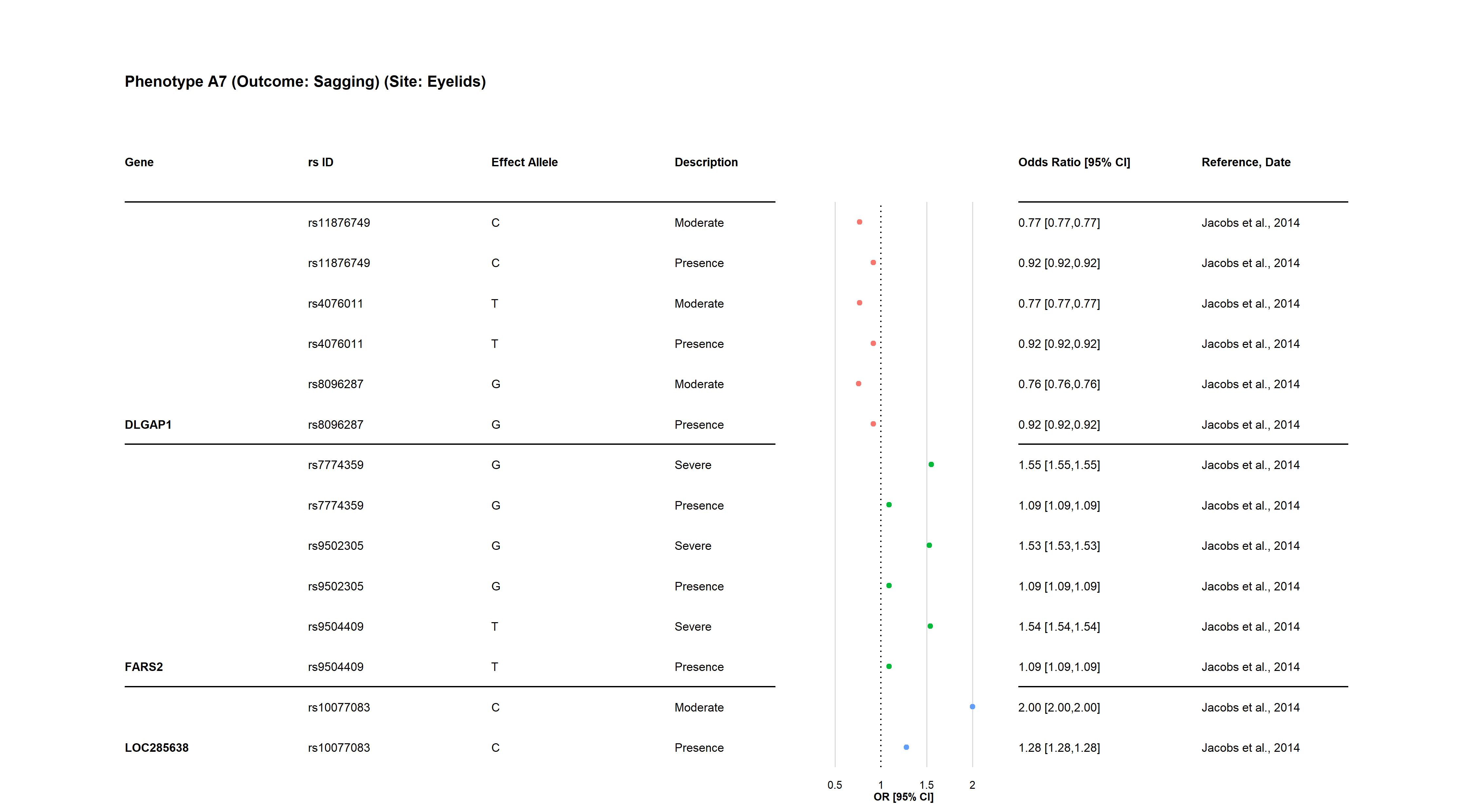

Supplement: Supplementary file 1 — Supplementary Information 1. [file 41598_2022_17443_MOESM1_ESM.zip › Supplementary Datasets/Dataset S2 - SNP-Phenotype Associations with 1 Study 1 Cohort/1 study 1 cohort Phenotype A7.2 (Outcome_Sagging) (Site_Eyelids).jpg]

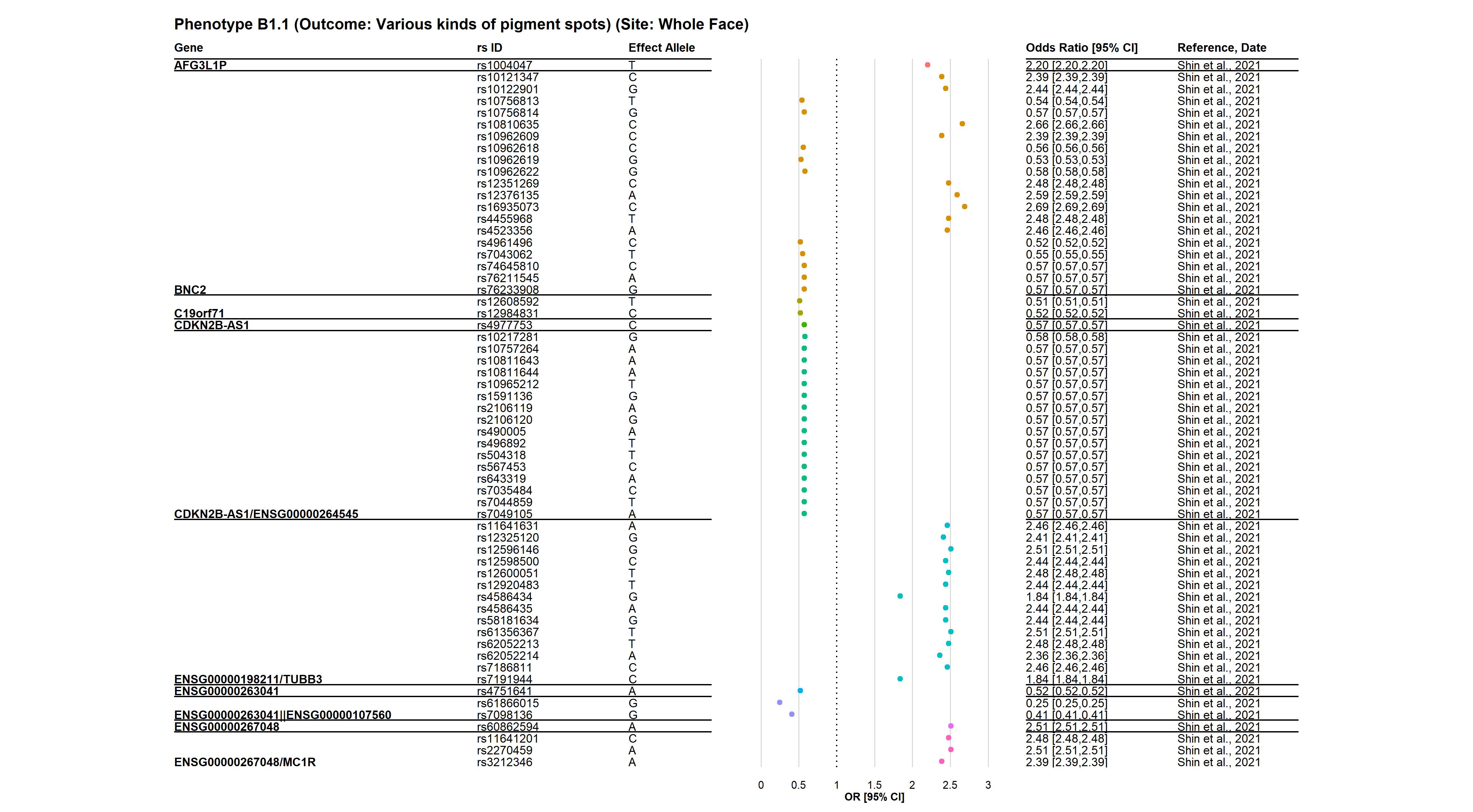

Supplement: Supplementary file 1 — Supplementary Information 1. [file 41598_2022_17443_MOESM1_ESM.zip › Supplementary Datasets/Dataset S2 - SNP-Phenotype Associations with 1 Study 1 Cohort/1 study 1 cohort Phenotype B1.1 (Outcome_Various kinds of pigment spots) (Site_Whole Face).jpg]

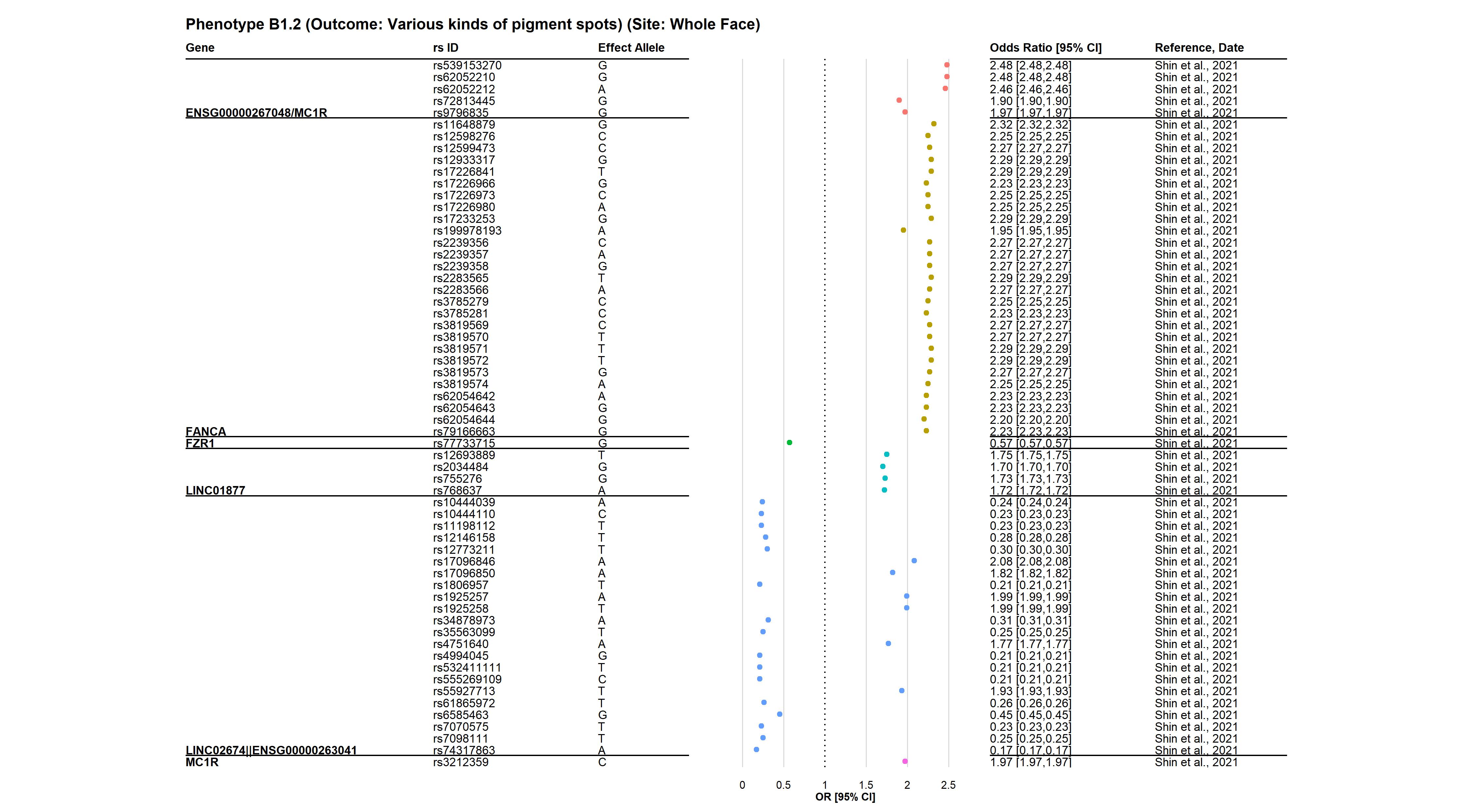

Supplement: Supplementary file 1 — Supplementary Information 1. [file 41598_2022_17443_MOESM1_ESM.zip › Supplementary Datasets/Dataset S2 - SNP-Phenotype Associations with 1 Study 1 Cohort/1 study 1 cohort Phenotype B1.2 (Outcome_Various kinds of pigment spots) (Site_Whole Face).jpg]

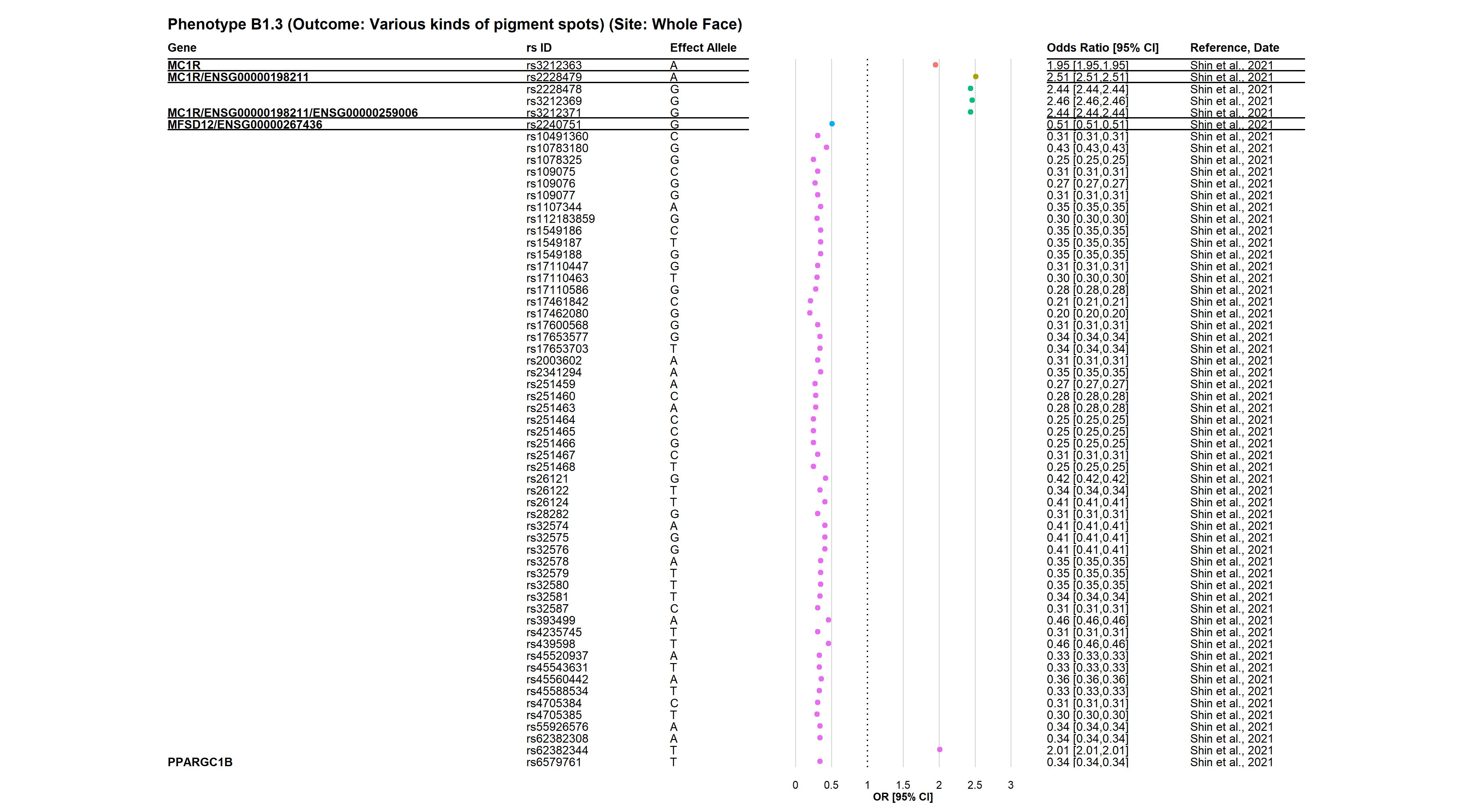

Supplement: Supplementary file 1 — Supplementary Information 1. [file 41598_2022_17443_MOESM1_ESM.zip › Supplementary Datasets/Dataset S2 - SNP-Phenotype Associations with 1 Study 1 Cohort/1 study 1 cohort Phenotype B1.3 (Outcome_Various kinds of pigment spots) (Site_Whole Face).jpg]

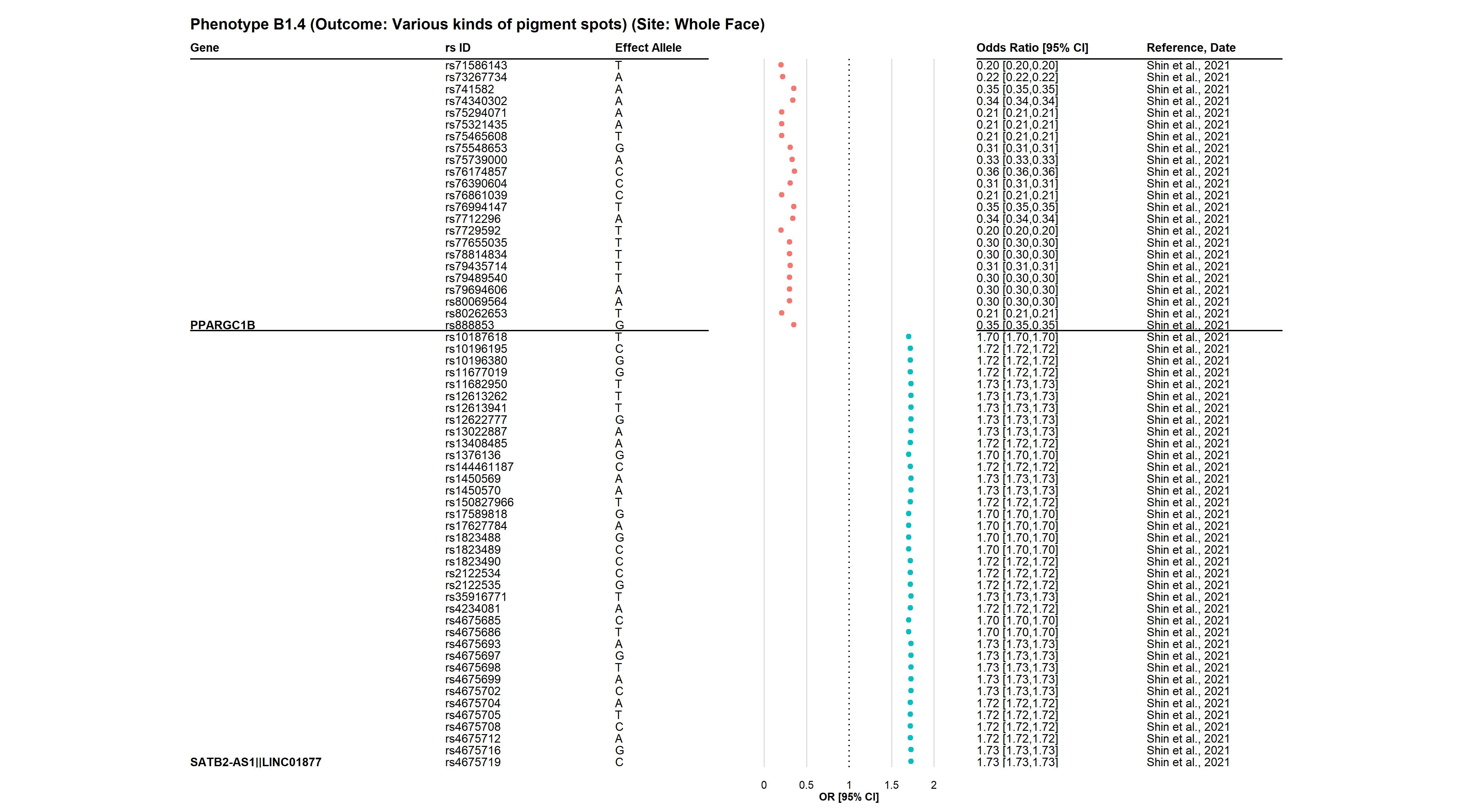

Supplement: Supplementary file 1 — Supplementary Information 1. [file 41598_2022_17443_MOESM1_ESM.zip › Supplementary Datasets/Dataset S2 - SNP-Phenotype Associations with 1 Study 1 Cohort/1 study 1 cohort Phenotype B1.4 (Outcome_Various kinds of pigment spots) (Site_Whole Face).jpg]

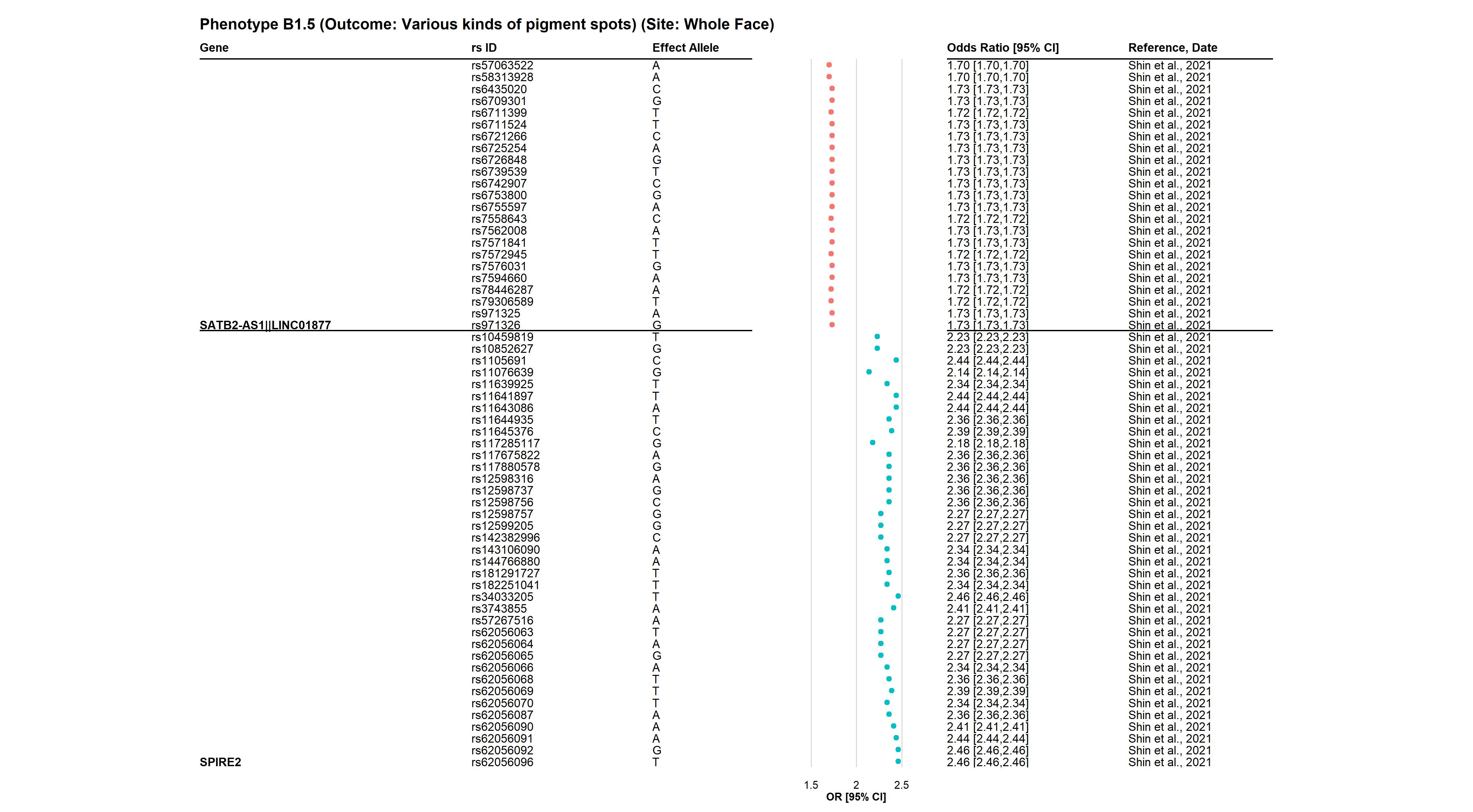

Supplement: Supplementary file 1 — Supplementary Information 1. [file 41598_2022_17443_MOESM1_ESM.zip › Supplementary Datasets/Dataset S2 - SNP-Phenotype Associations with 1 Study 1 Cohort/1 study 1 cohort Phenotype B1.5 (Outcome_Various kinds of pigment spots) (Site_Whole Face).jpg]

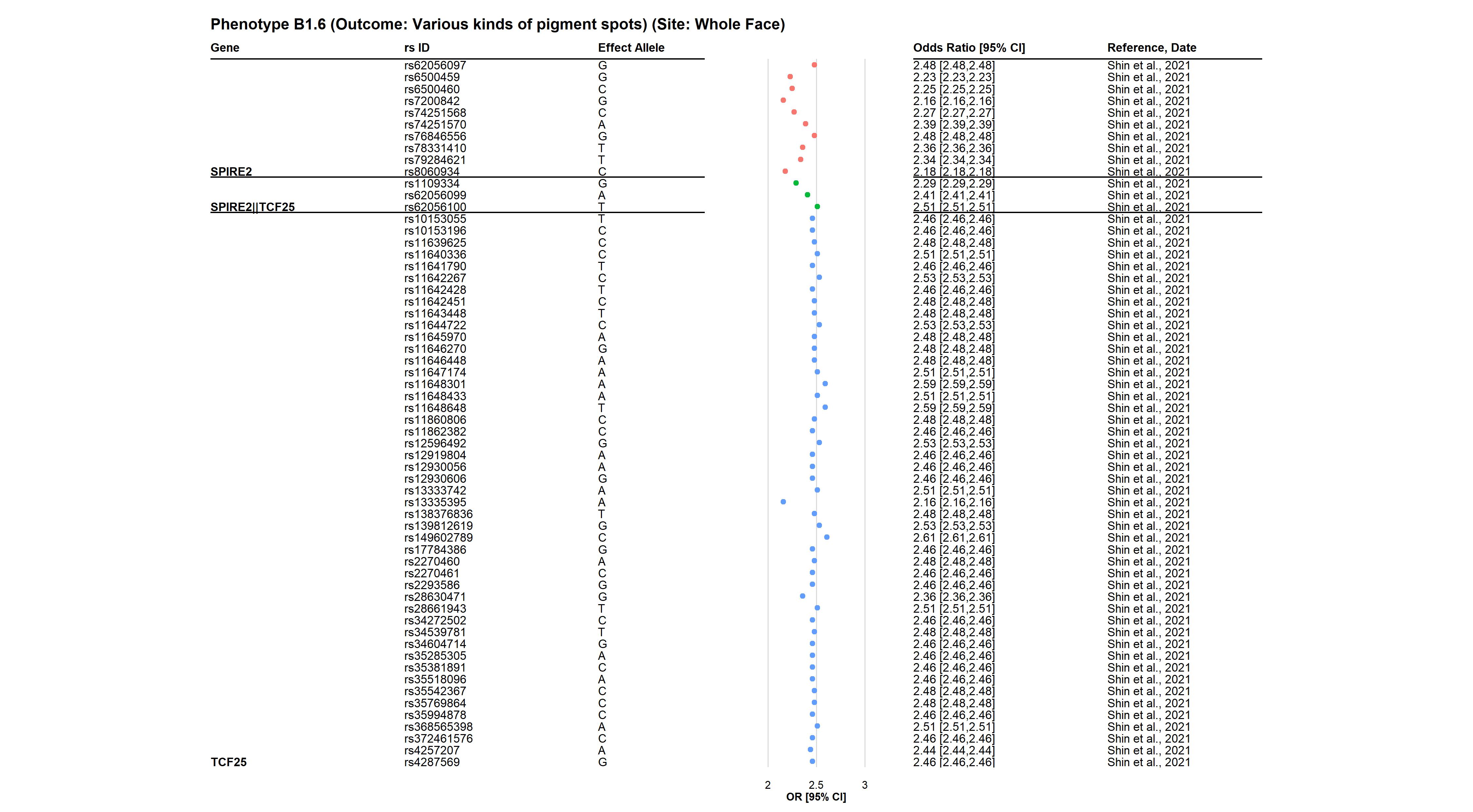

Supplement: Supplementary file 1 — Supplementary Information 1. [file 41598_2022_17443_MOESM1_ESM.zip › Supplementary Datasets/Dataset S2 - SNP-Phenotype Associations with 1 Study 1 Cohort/1 study 1 cohort Phenotype B1.6 (Outcome_Various kinds of pigment spots) (Site_Whole Face).jpg]

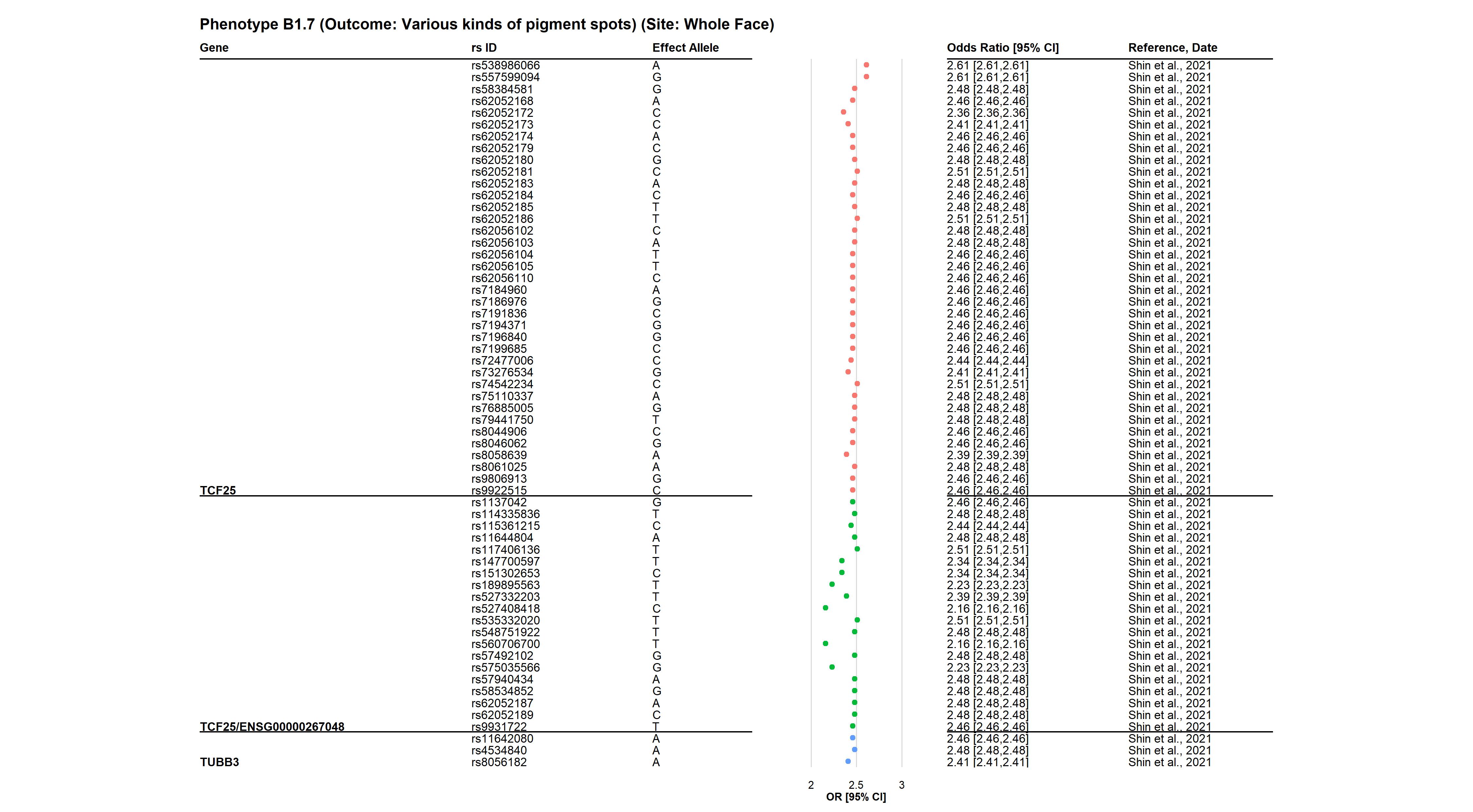

Supplement: Supplementary file 1 — Supplementary Information 1. [file 41598_2022_17443_MOESM1_ESM.zip › Supplementary Datasets/Dataset S2 - SNP-Phenotype Associations with 1 Study 1 Cohort/1 study 1 cohort Phenotype B1.7 (Outcome_Various kinds of pigment spots) (Site_Whole Face).jpg]

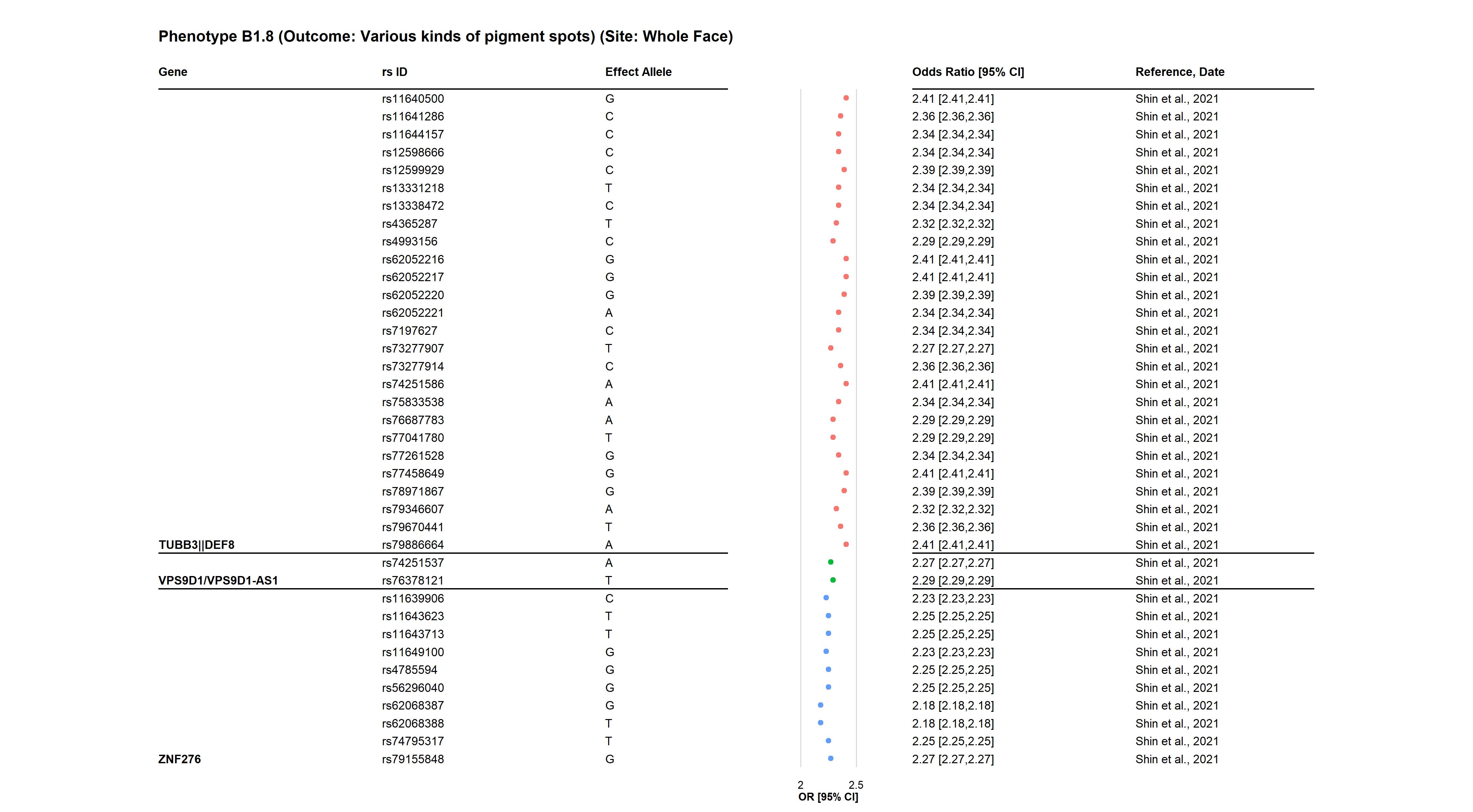

Supplement: Supplementary file 1 — Supplementary Information 1. [file 41598_2022_17443_MOESM1_ESM.zip › Supplementary Datasets/Dataset S2 - SNP-Phenotype Associations with 1 Study 1 Cohort/1 study 1 cohort Phenotype B1.8 (Outcome_Various kinds of pigment spots) (Site_Whole Face).jpg]

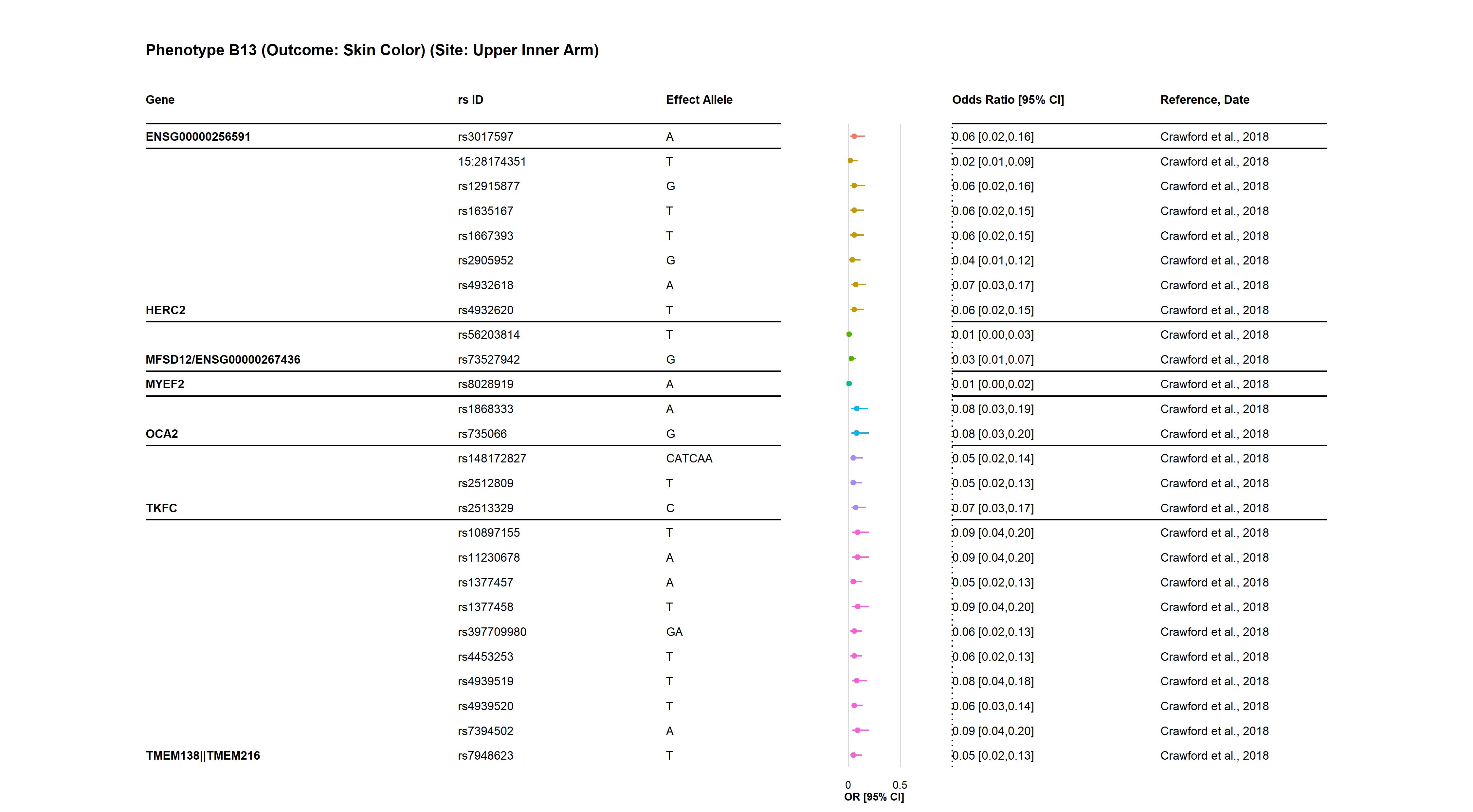

Supplement: Supplementary file 1 — Supplementary Information 1. [file 41598_2022_17443_MOESM1_ESM.zip › Supplementary Datasets/Dataset S2 - SNP-Phenotype Associations with 1 Study 1 Cohort/1 study 1 cohort Phenotype B13.1 (Outcome_Skin Color) (Site_Upper Inner Arm).jpg]

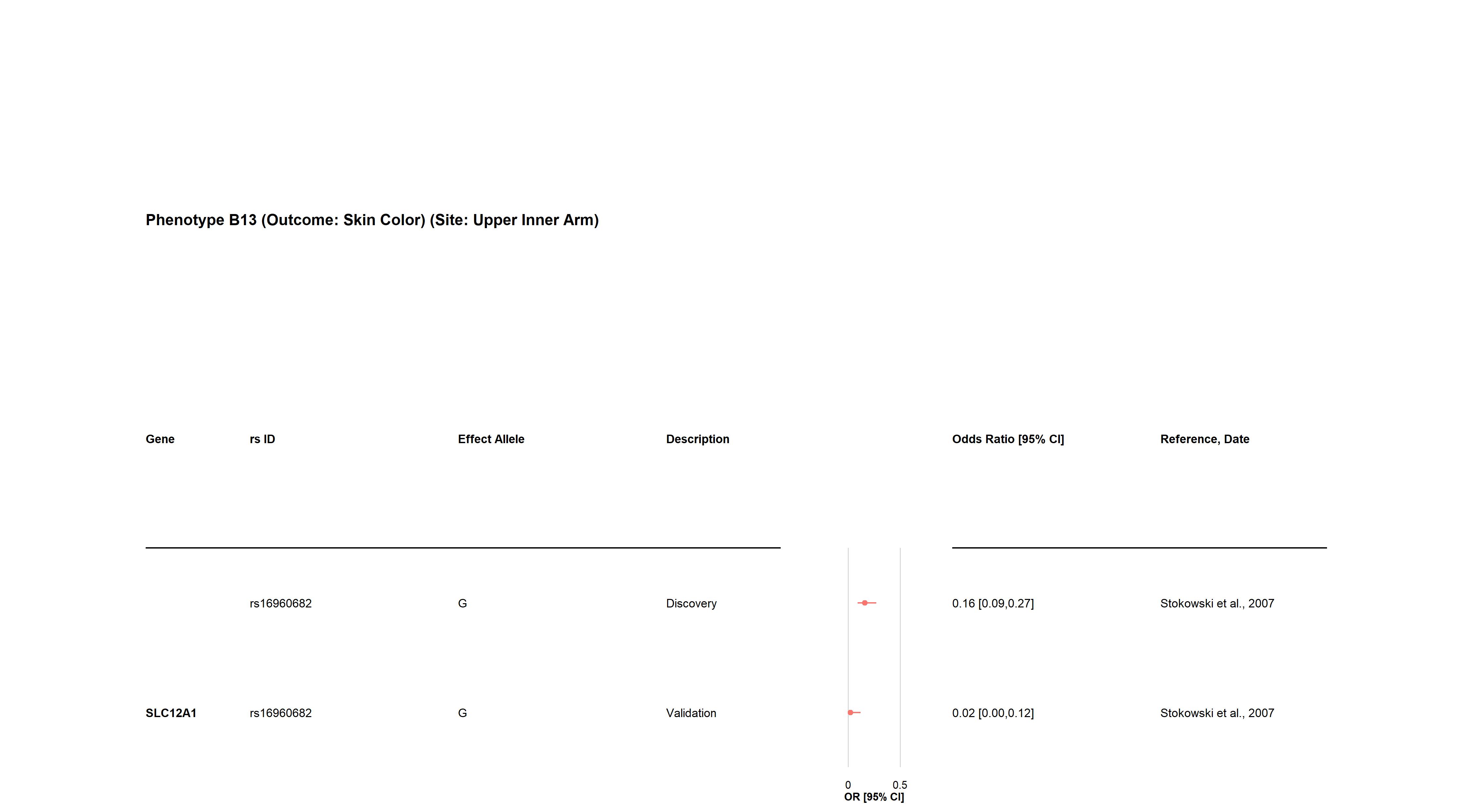

Supplement: Supplementary file 1 — Supplementary Information 1. [file 41598_2022_17443_MOESM1_ESM.zip › Supplementary Datasets/Dataset S2 - SNP-Phenotype Associations with 1 Study 1 Cohort/1 study 1 cohort Phenotype B13.2 (Outcome_Skin Color) (Site_Upper Inner Arm).jpg]

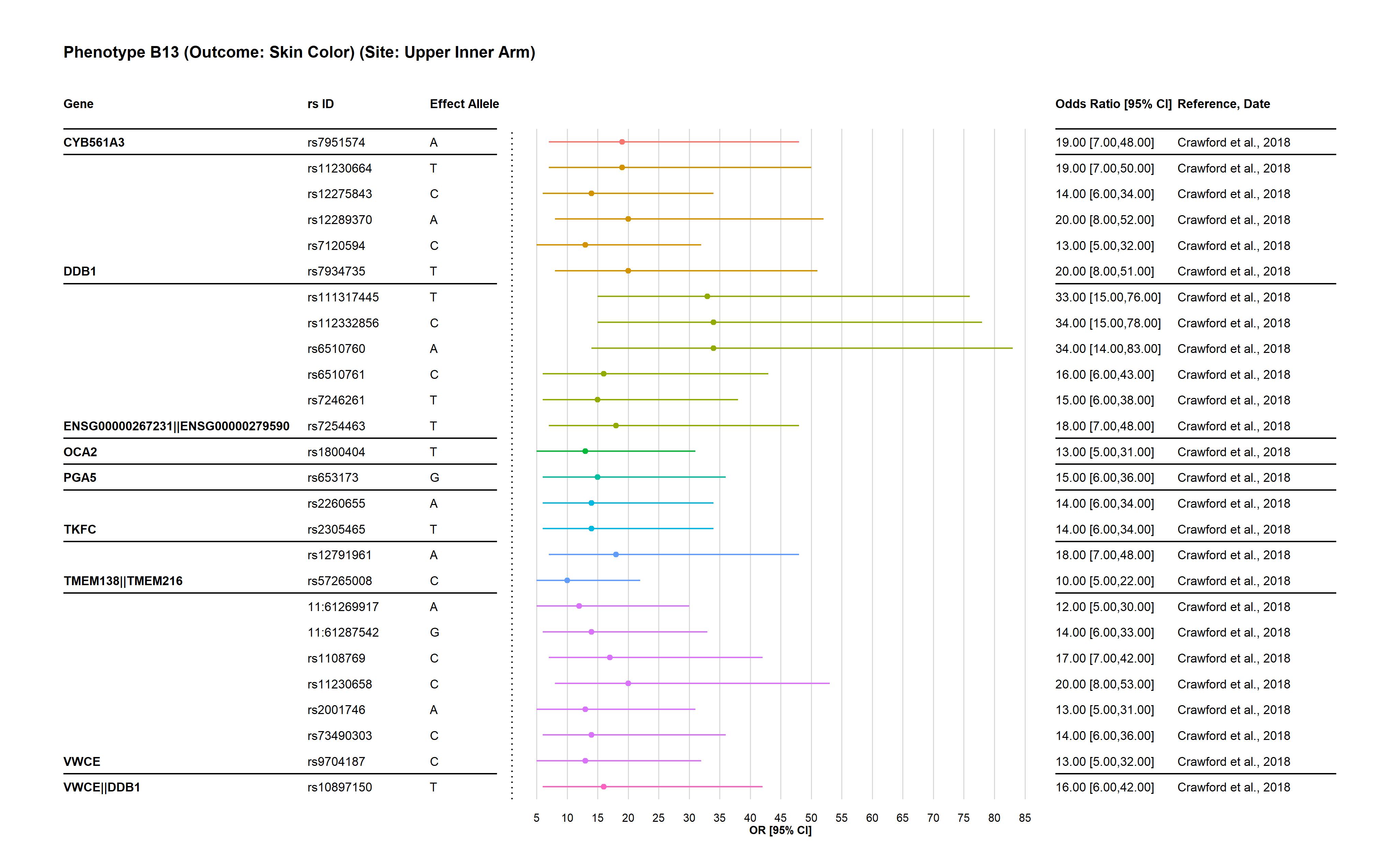

Supplement: Supplementary file 1 — Supplementary Information 1. [file 41598_2022_17443_MOESM1_ESM.zip › Supplementary Datasets/Dataset S2 - SNP-Phenotype Associations with 1 Study 1 Cohort/1 study 1 cohort Phenotype B13.3 (Outcome_Skin Color) (Site_Upper Inner Arm).jpg]

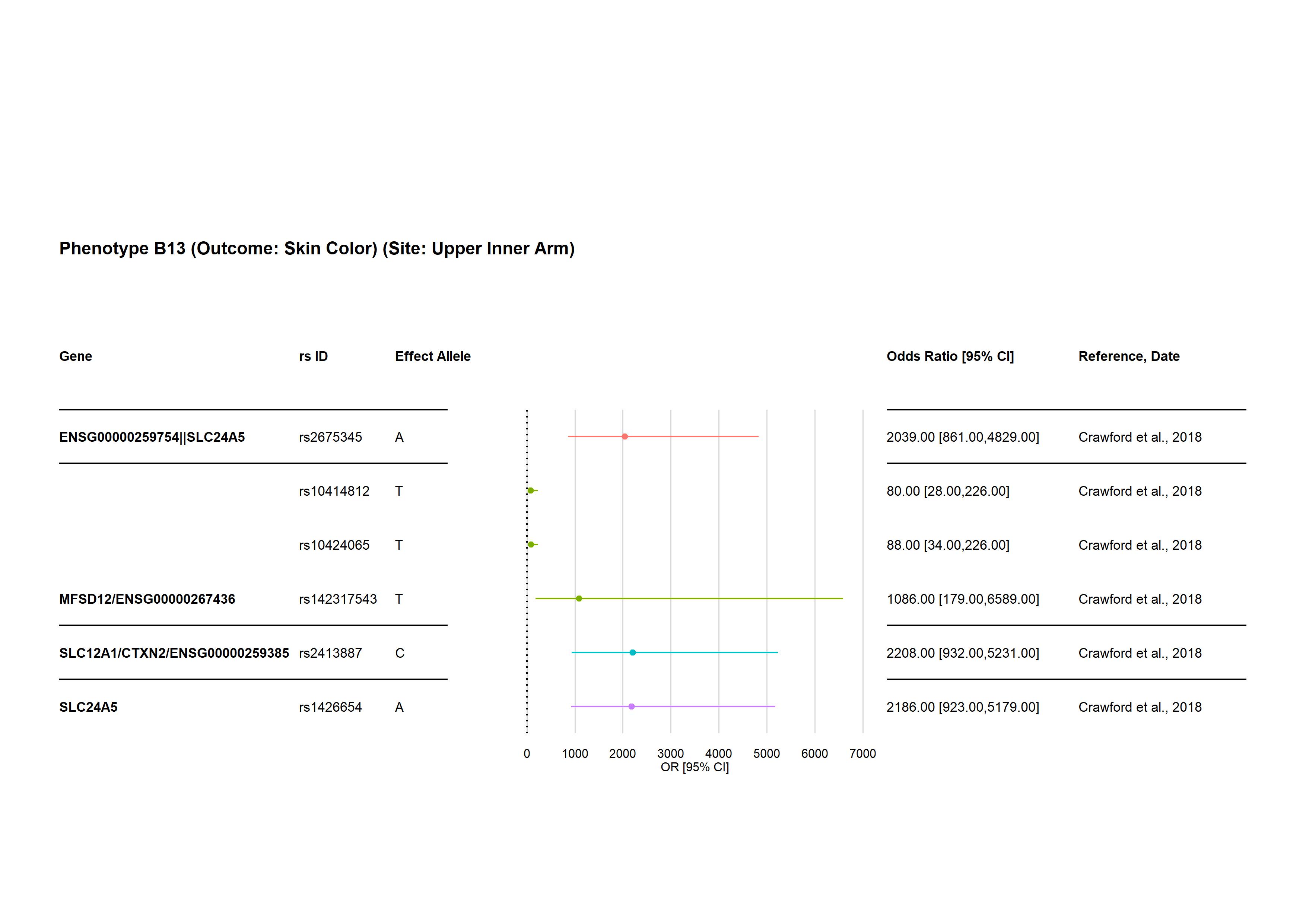

Supplement: Supplementary file 1 — Supplementary Information 1. [file 41598_2022_17443_MOESM1_ESM.zip › Supplementary Datasets/Dataset S2 - SNP-Phenotype Associations with 1 Study 1 Cohort/1 study 1 cohort Phenotype B13.4 (Outcome_Skin Color) (Site_Upper Inner Arm).jpg]

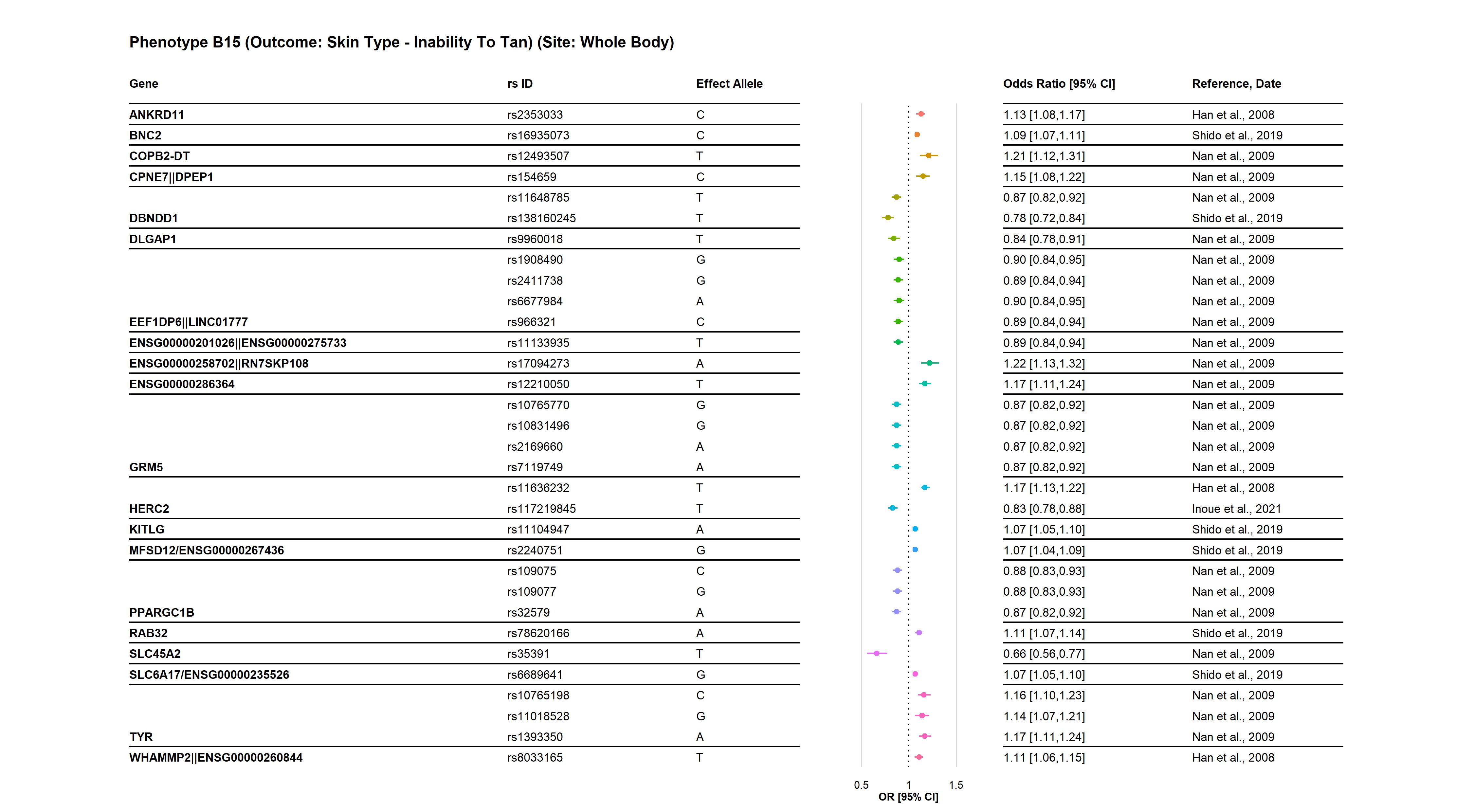

Supplement: Supplementary file 1 — Supplementary Information 1. [file 41598_2022_17443_MOESM1_ESM.zip › Supplementary Datasets/Dataset S2 - SNP-Phenotype Associations with 1 Study 1 Cohort/1 study 1 cohort Phenotype B15 (Outcome_Skin Type - Inability To Tan) (Site_Whole Body).jpg]

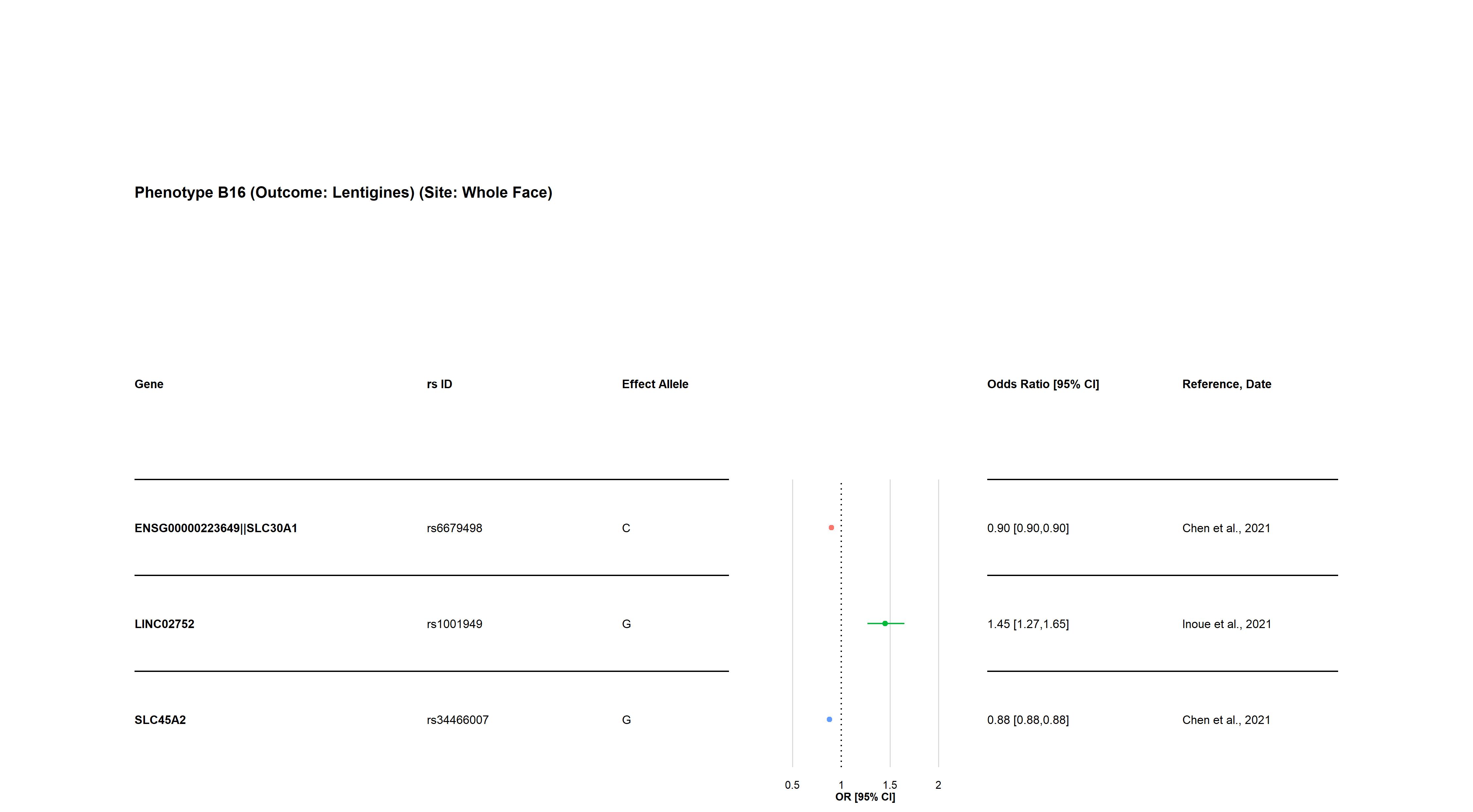

Supplement: Supplementary file 1 — Supplementary Information 1. [file 41598_2022_17443_MOESM1_ESM.zip › Supplementary Datasets/Dataset S2 - SNP-Phenotype Associations with 1 Study 1 Cohort/1 study 1 cohort Phenotype B16 (Outcome_Lentigines) (Site_Whole Face).jpg]

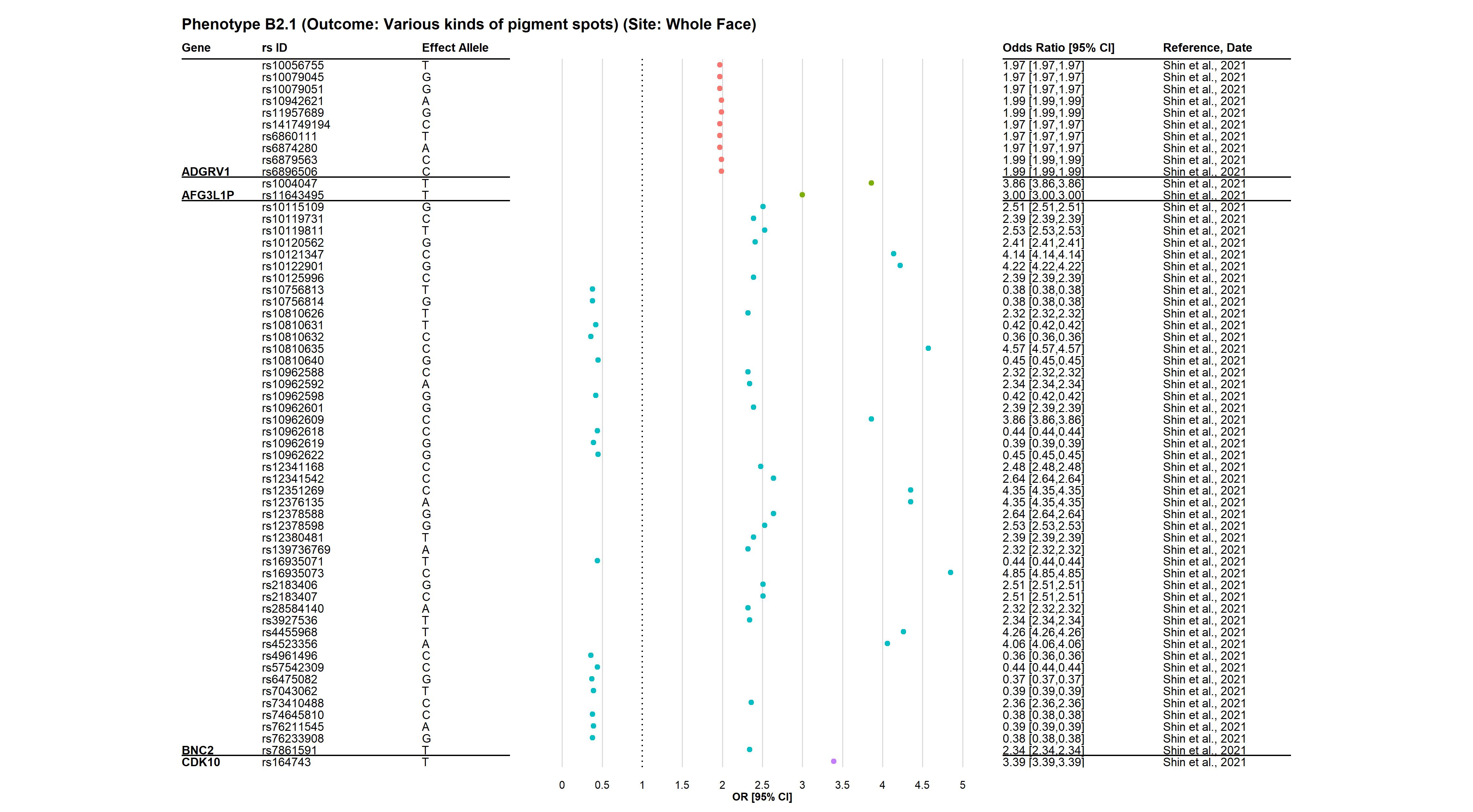

Supplement: Supplementary file 1 — Supplementary Information 1. [file 41598_2022_17443_MOESM1_ESM.zip › Supplementary Datasets/Dataset S2 - SNP-Phenotype Associations with 1 Study 1 Cohort/1 study 1 cohort Phenotype B2.1 (Outcome_Various kinds of pigment spots) (Site_Whole Face).jpg]

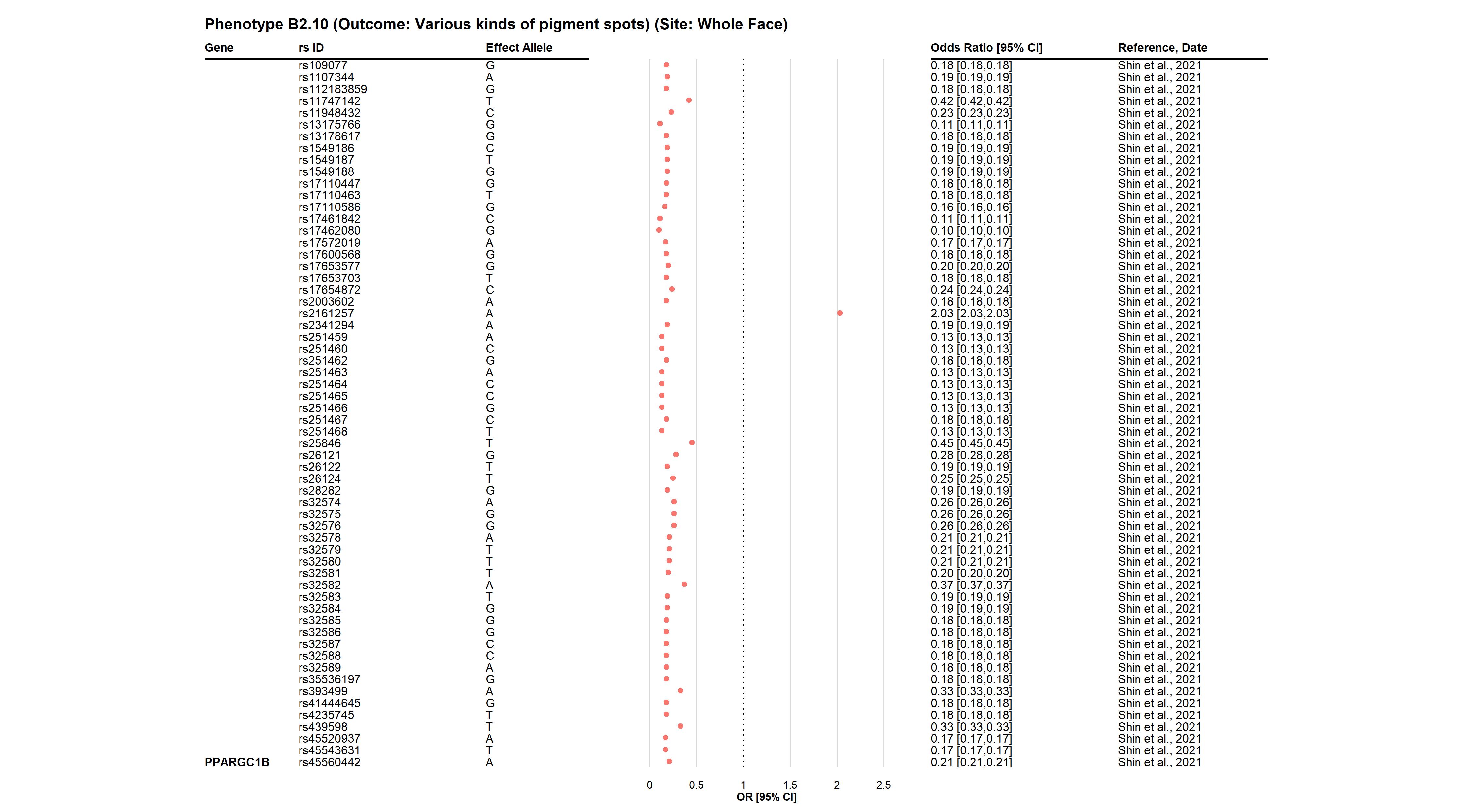

Supplement: Supplementary file 1 — Supplementary Information 1. [file 41598_2022_17443_MOESM1_ESM.zip › Supplementary Datasets/Dataset S2 - SNP-Phenotype Associations with 1 Study 1 Cohort/1 study 1 cohort Phenotype B2.10 (Outcome_Various kinds of pigment spots) (Site_Whole Face).jpg]

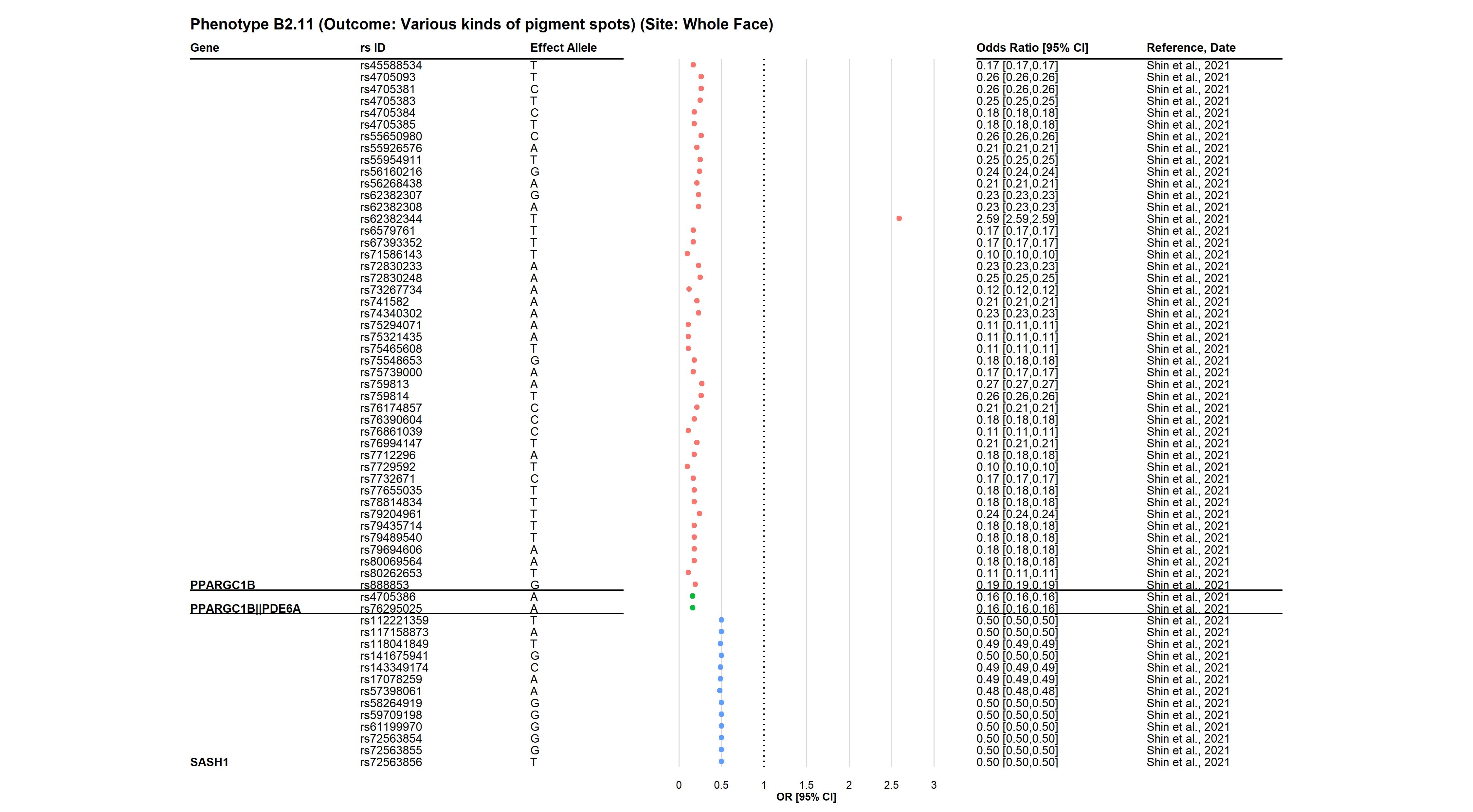

Supplement: Supplementary file 1 — Supplementary Information 1. [file 41598_2022_17443_MOESM1_ESM.zip › Supplementary Datasets/Dataset S2 - SNP-Phenotype Associations with 1 Study 1 Cohort/1 study 1 cohort Phenotype B2.11 (Outcome_Various kinds of pigment spots) (Site_Whole Face).jpg]

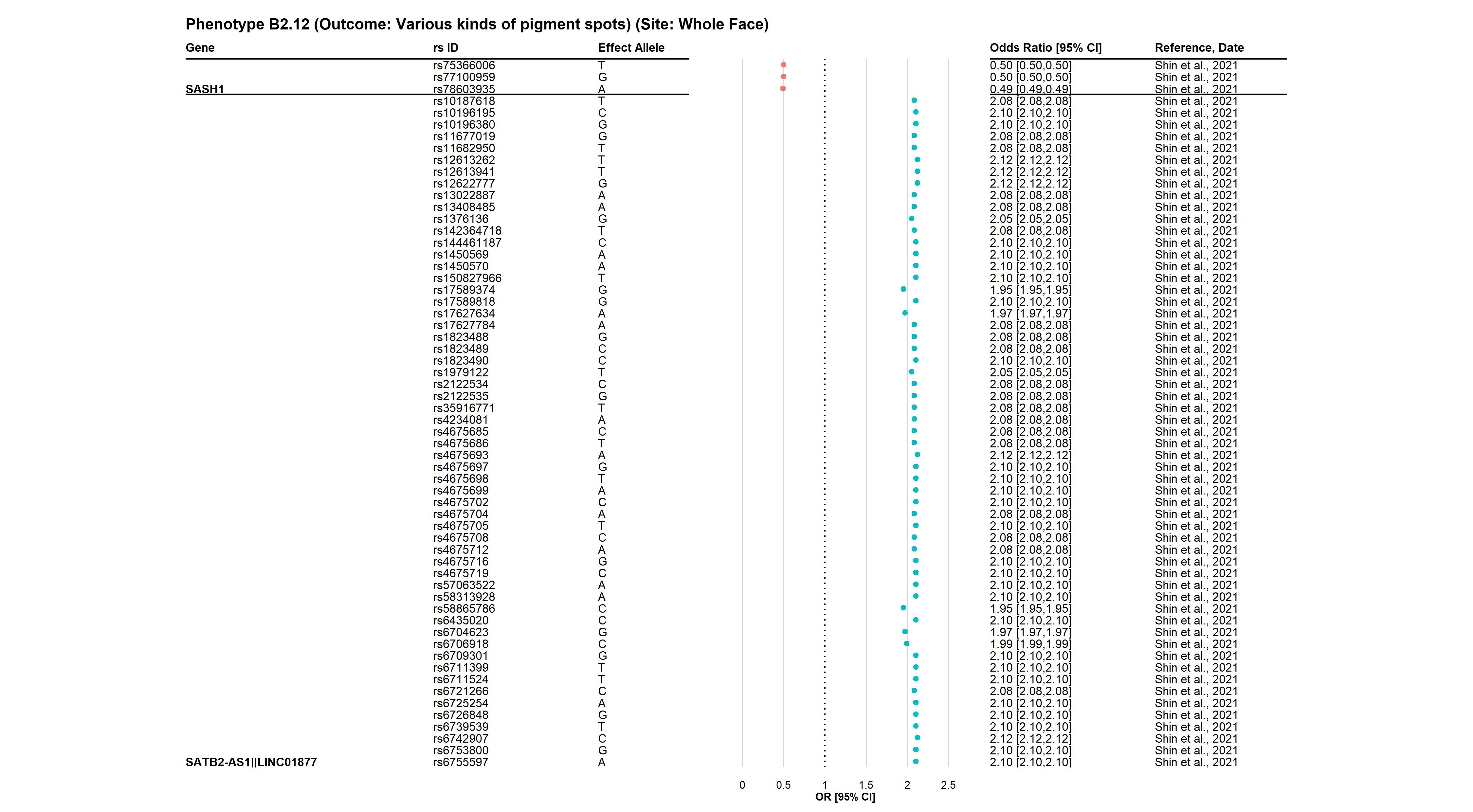

Supplement: Supplementary file 1 — Supplementary Information 1. [file 41598_2022_17443_MOESM1_ESM.zip › Supplementary Datasets/Dataset S2 - SNP-Phenotype Associations with 1 Study 1 Cohort/1 study 1 cohort Phenotype B2.12 (Outcome_Various kinds of pigment spots) (Site_Whole Face).jpg]

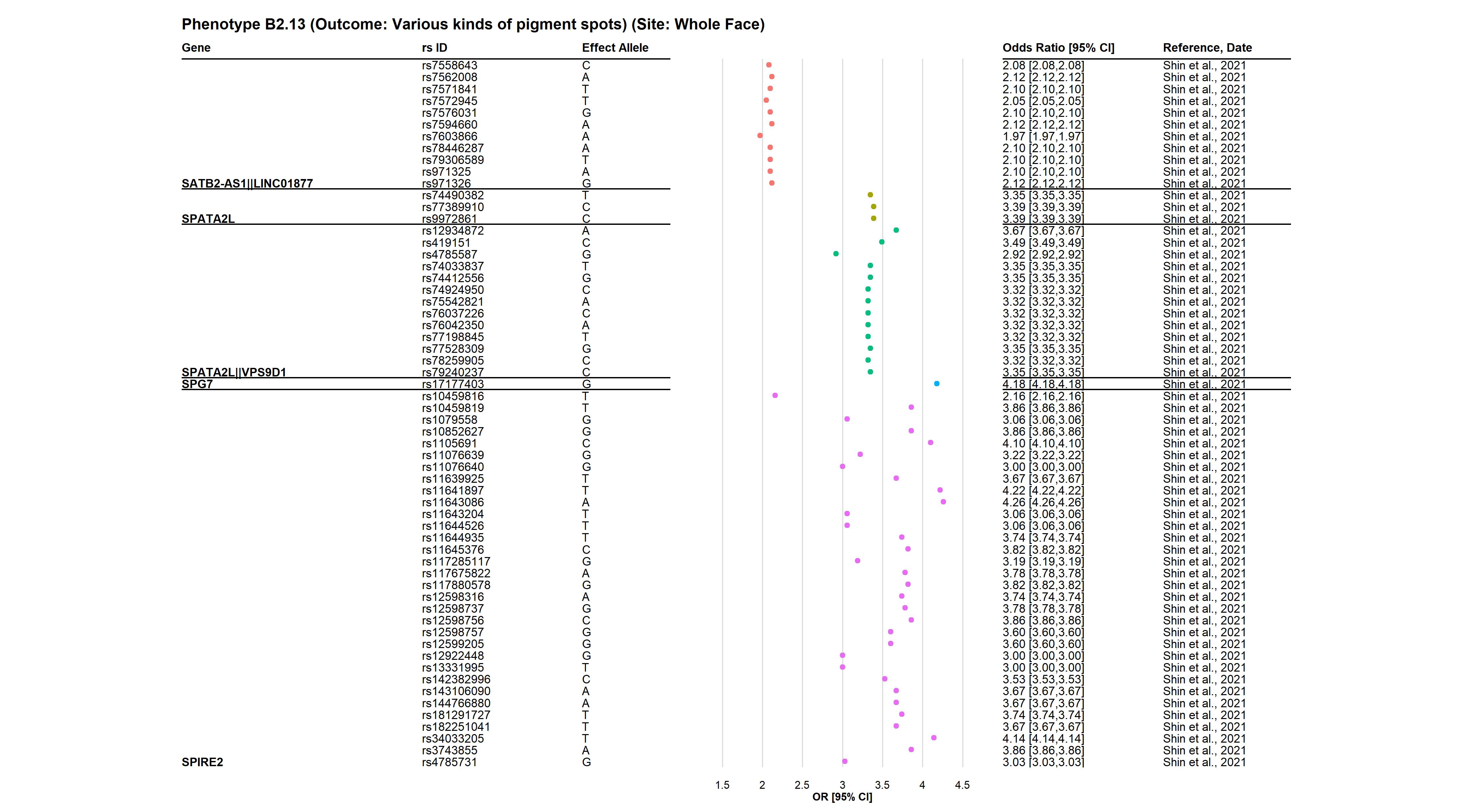

Supplement: Supplementary file 1 — Supplementary Information 1. [file 41598_2022_17443_MOESM1_ESM.zip › Supplementary Datasets/Dataset S2 - SNP-Phenotype Associations with 1 Study 1 Cohort/1 study 1 cohort Phenotype B2.13 (Outcome_Various kinds of pigment spots) (Site_Whole Face).jpg]

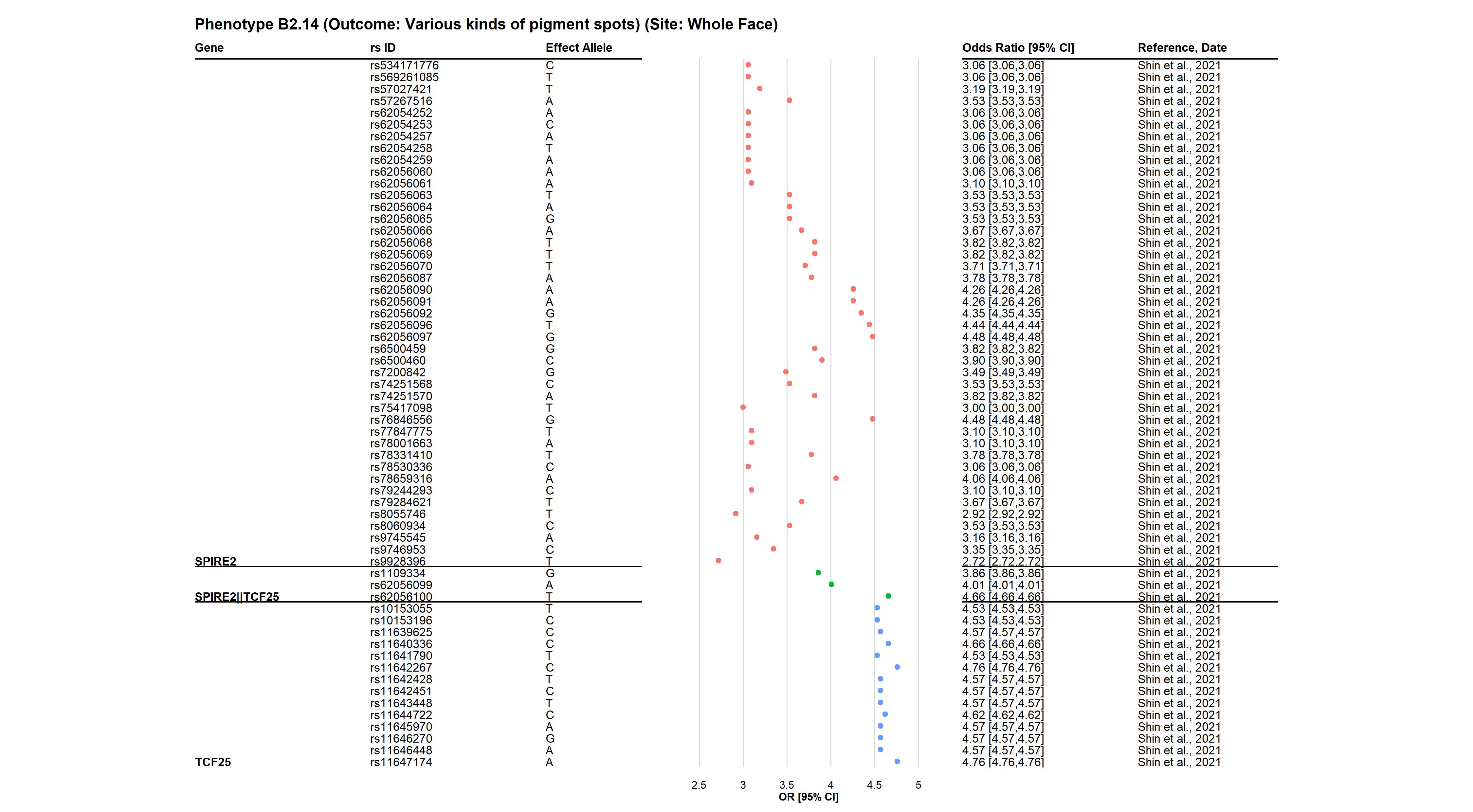

Supplement: Supplementary file 1 — Supplementary Information 1. [file 41598_2022_17443_MOESM1_ESM.zip › Supplementary Datasets/Dataset S2 - SNP-Phenotype Associations with 1 Study 1 Cohort/1 study 1 cohort Phenotype B2.14 (Outcome_Various kinds of pigment spots) (Site_Whole Face).jpg]

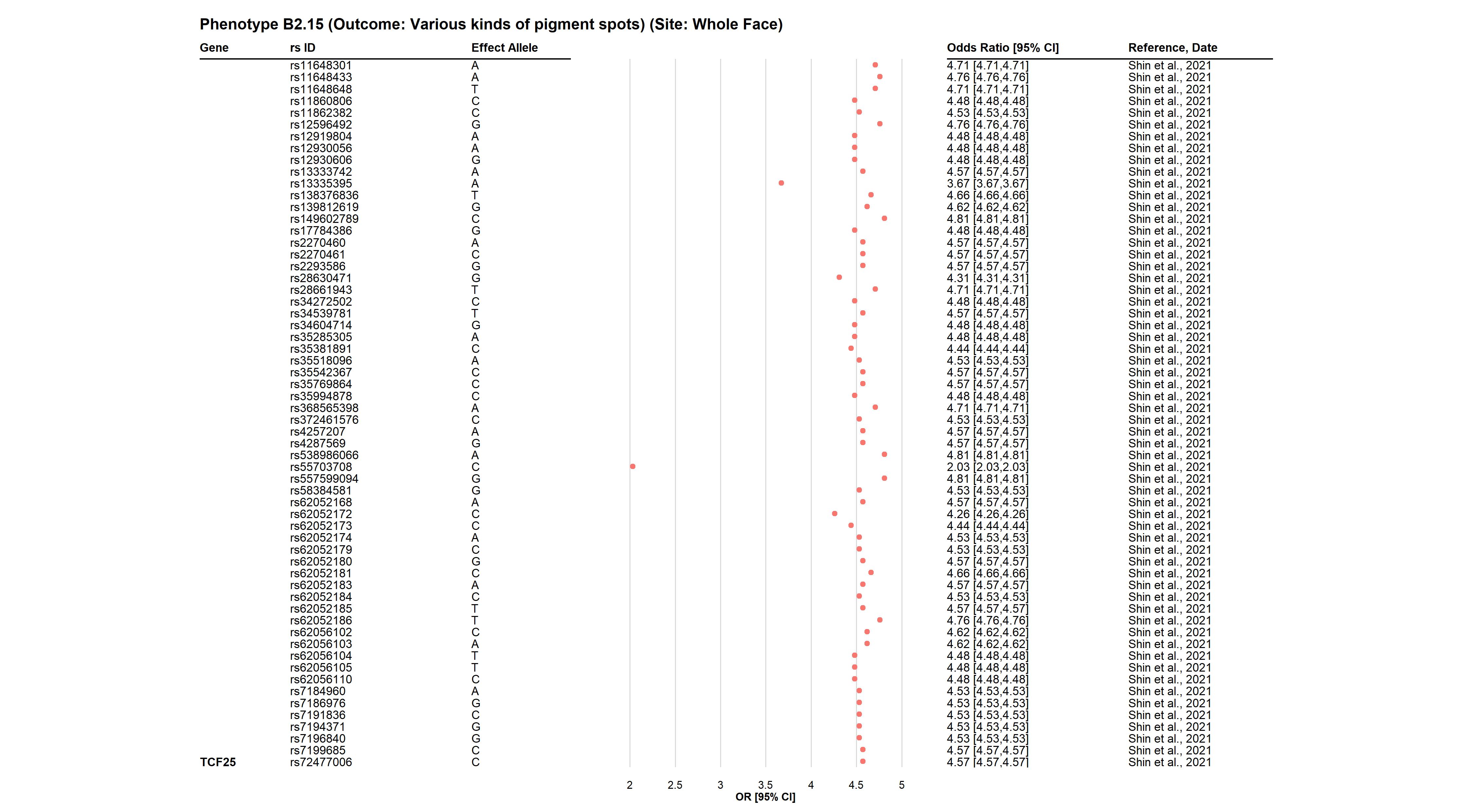

Supplement: Supplementary file 1 — Supplementary Information 1. [file 41598_2022_17443_MOESM1_ESM.zip › Supplementary Datasets/Dataset S2 - SNP-Phenotype Associations with 1 Study 1 Cohort/1 study 1 cohort Phenotype B2.15 (Outcome_Various kinds of pigment spots) (Site_Whole Face).jpg]

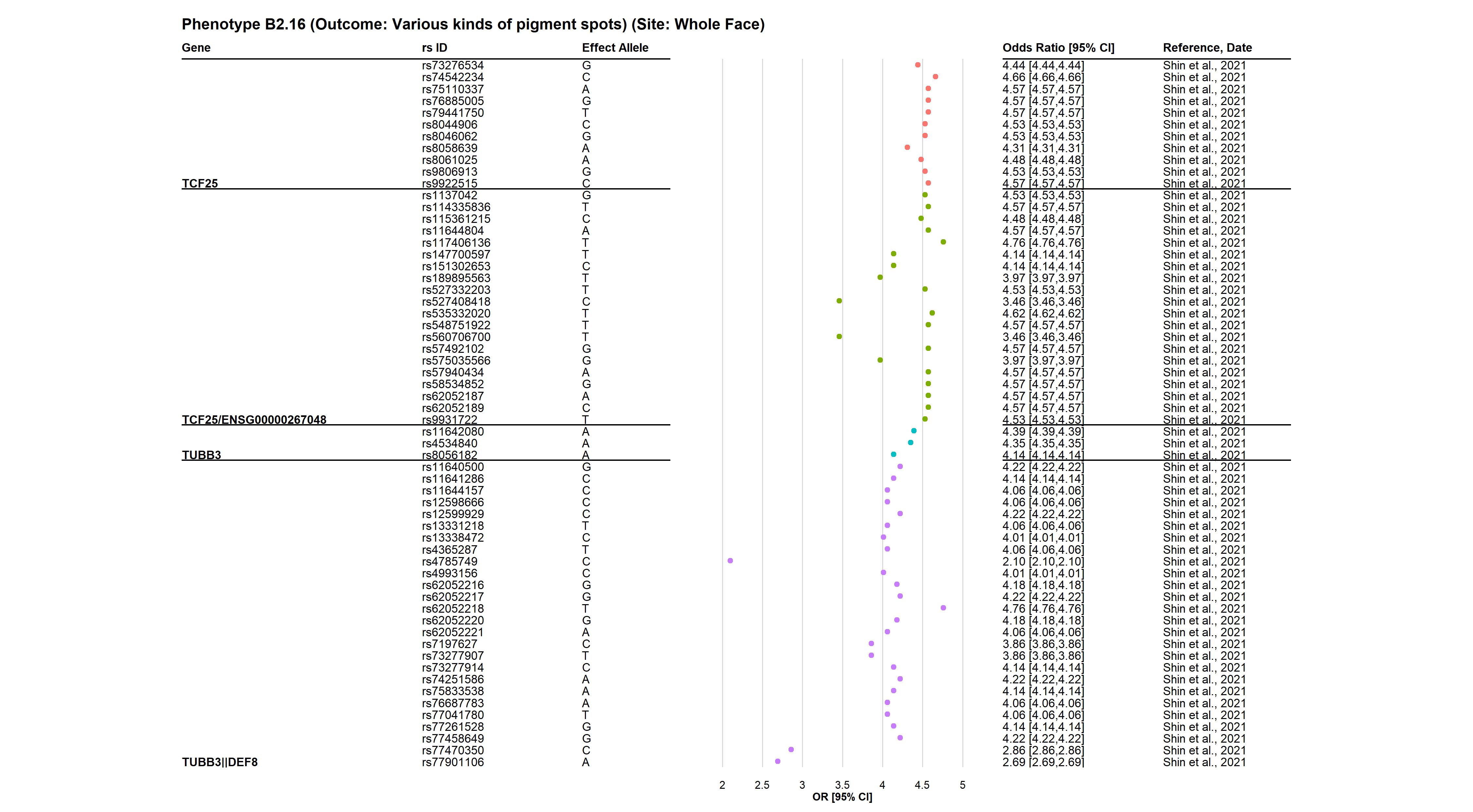

Supplement: Supplementary file 1 — Supplementary Information 1. [file 41598_2022_17443_MOESM1_ESM.zip › Supplementary Datasets/Dataset S2 - SNP-Phenotype Associations with 1 Study 1 Cohort/1 study 1 cohort Phenotype B2.16 (Outcome_Various kinds of pigment spots) (Site_Whole Face).jpg]

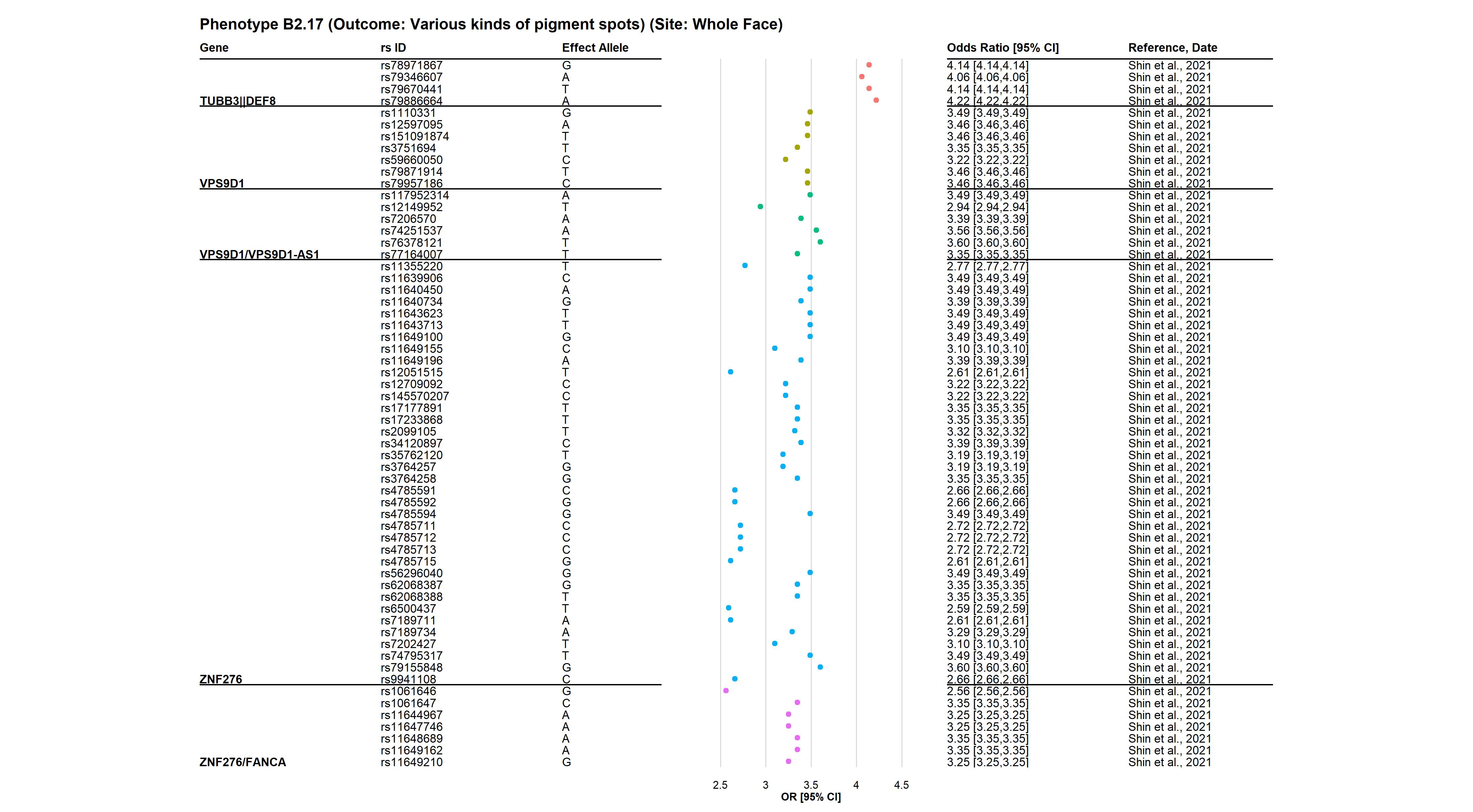

Supplement: Supplementary file 1 — Supplementary Information 1. [file 41598_2022_17443_MOESM1_ESM.zip › Supplementary Datasets/Dataset S2 - SNP-Phenotype Associations with 1 Study 1 Cohort/1 study 1 cohort Phenotype B2.17 (Outcome_Various kinds of pigment spots) (Site_Whole Face).jpg]

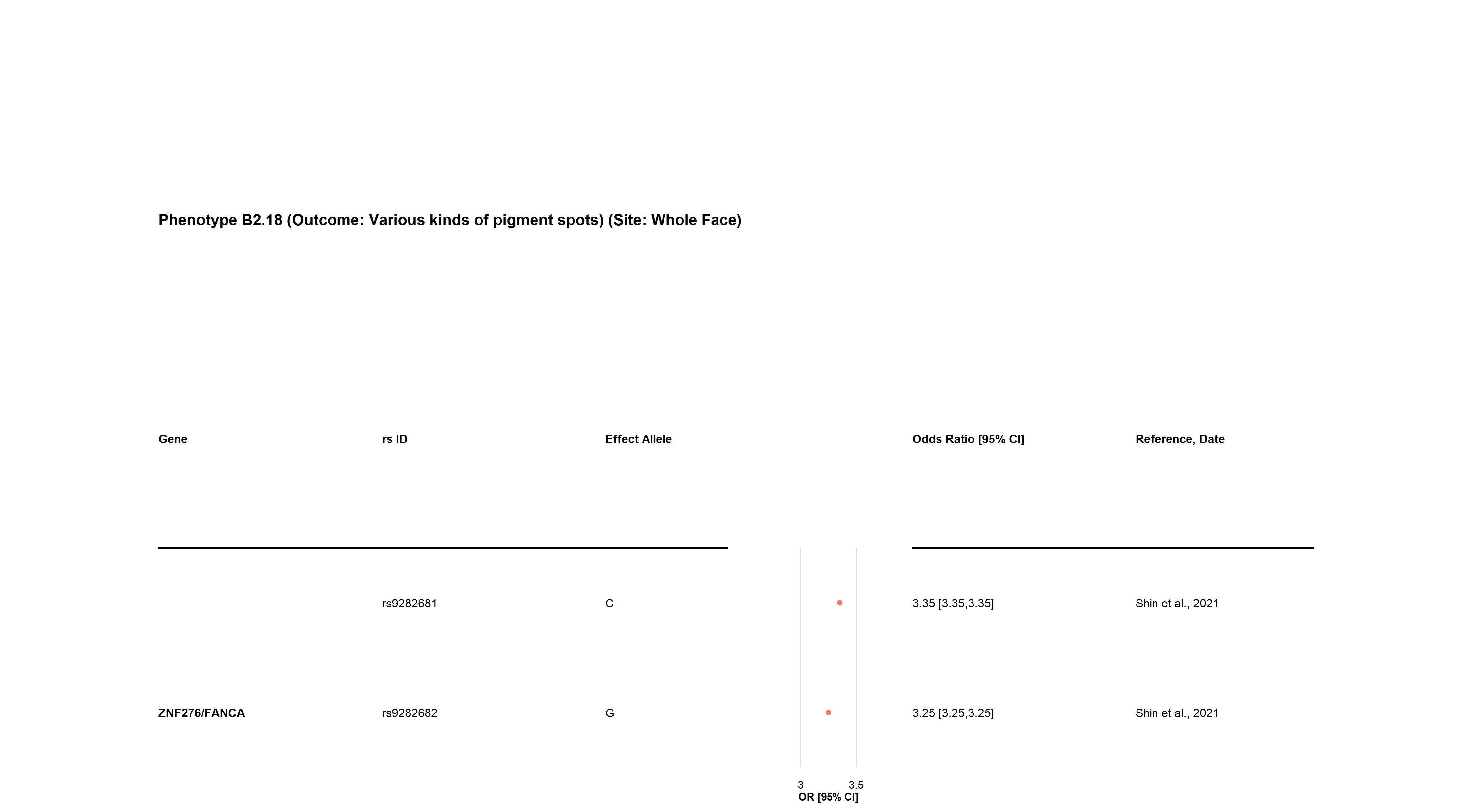

Supplement: Supplementary file 1 — Supplementary Information 1. [file 41598_2022_17443_MOESM1_ESM.zip › Supplementary Datasets/Dataset S2 - SNP-Phenotype Associations with 1 Study 1 Cohort/1 study 1 cohort Phenotype B2.18 (Outcome_Various kinds of pigment spots) (Site_Whole Face).jpg]

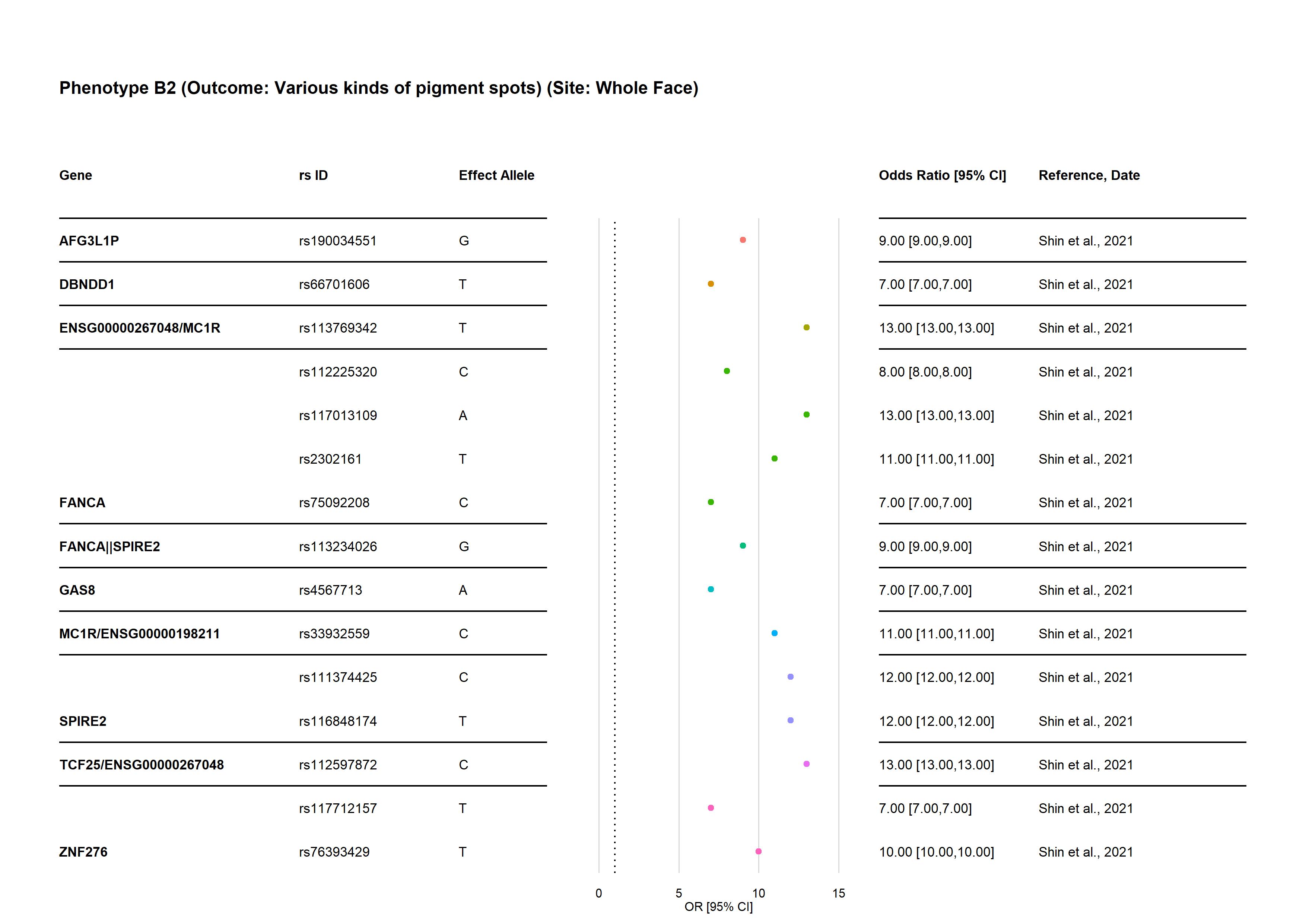

Supplement: Supplementary file 1 — Supplementary Information 1. [file 41598_2022_17443_MOESM1_ESM.zip › Supplementary Datasets/Dataset S2 - SNP-Phenotype Associations with 1 Study 1 Cohort/1 study 1 cohort Phenotype B2.19 (Outcome_Various kinds of pigment spots) (Site_Whole Face).jpg]

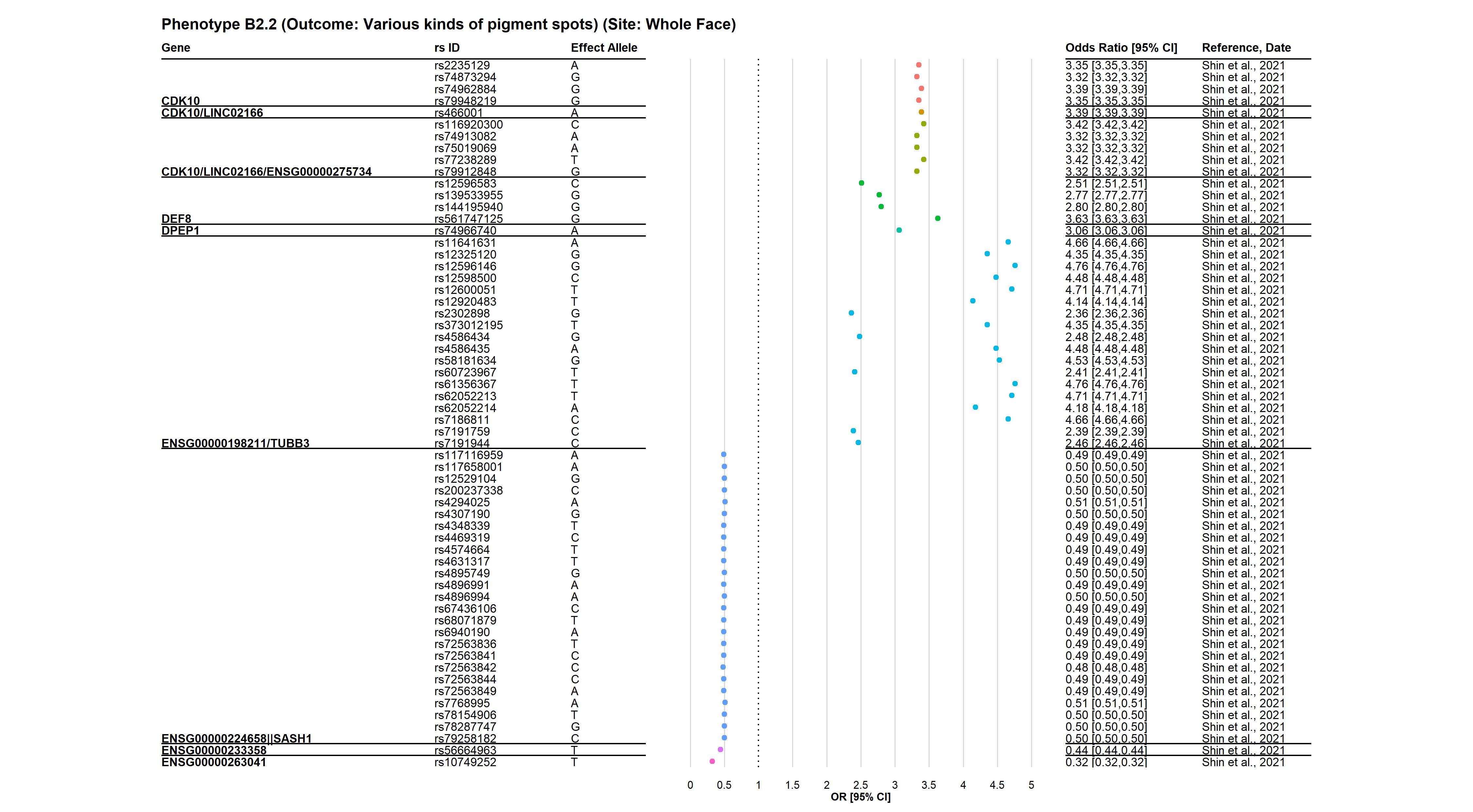

Supplement: Supplementary file 1 — Supplementary Information 1. [file 41598_2022_17443_MOESM1_ESM.zip › Supplementary Datasets/Dataset S2 - SNP-Phenotype Associations with 1 Study 1 Cohort/1 study 1 cohort Phenotype B2.2 (Outcome_Various kinds of pigment spots) (Site_Whole Face).jpg]

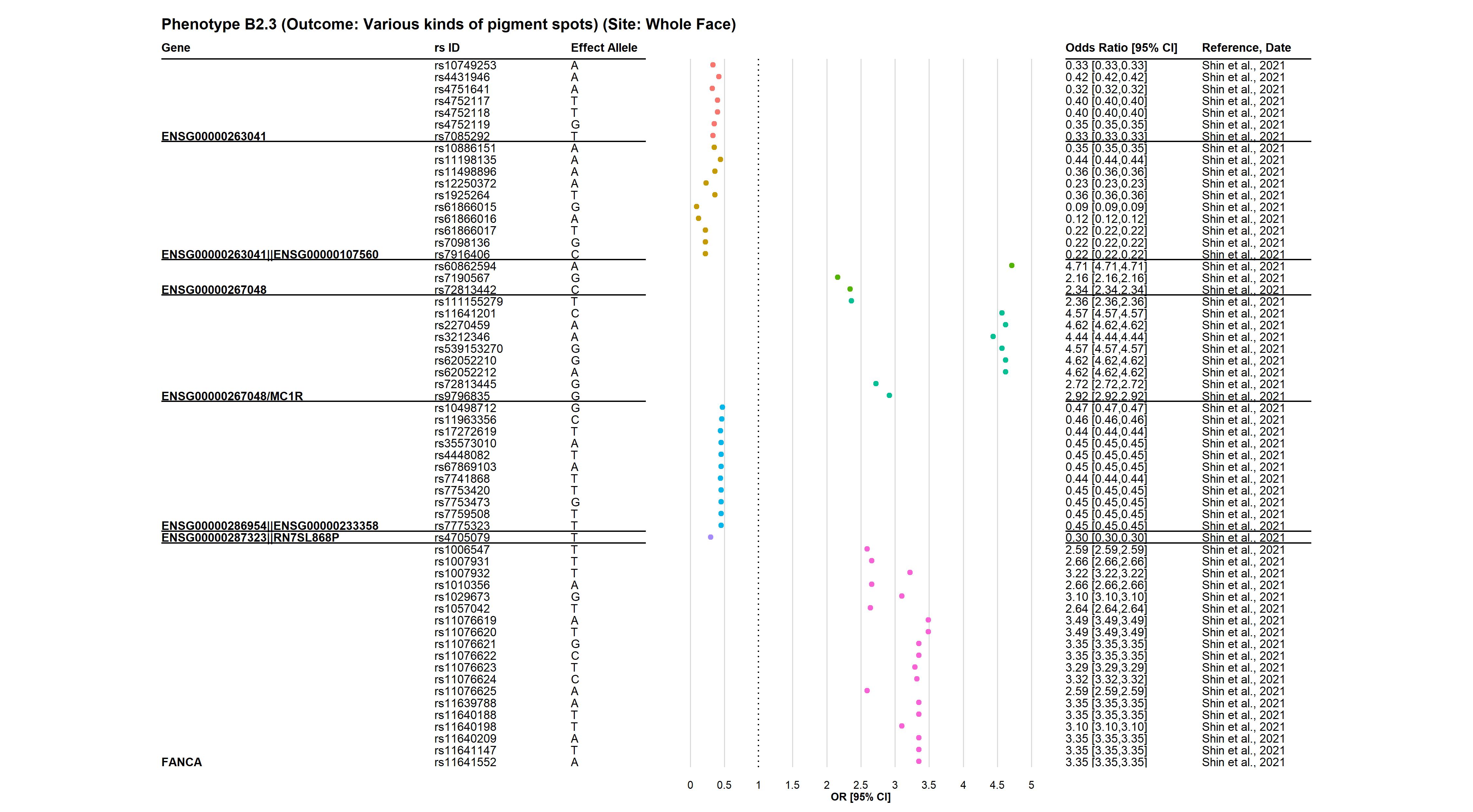

Supplement: Supplementary file 1 — Supplementary Information 1. [file 41598_2022_17443_MOESM1_ESM.zip › Supplementary Datasets/Dataset S2 - SNP-Phenotype Associations with 1 Study 1 Cohort/1 study 1 cohort Phenotype B2.3 (Outcome_Various kinds of pigment spots) (Site_Whole Face).jpg]

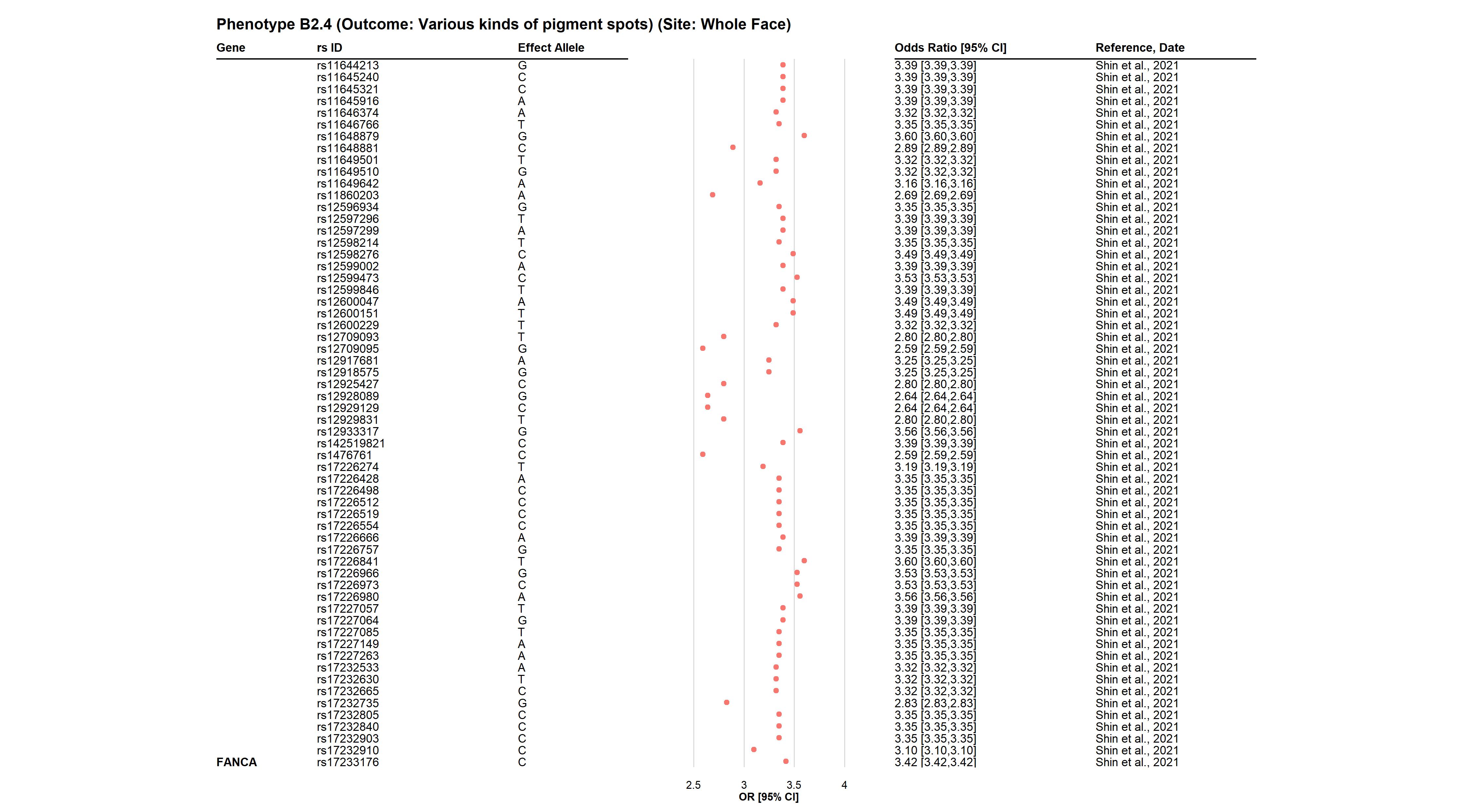

Supplement: Supplementary file 1 — Supplementary Information 1. [file 41598_2022_17443_MOESM1_ESM.zip › Supplementary Datasets/Dataset S2 - SNP-Phenotype Associations with 1 Study 1 Cohort/1 study 1 cohort Phenotype B2.4 (Outcome_Various kinds of pigment spots) (Site_Whole Face).jpg]

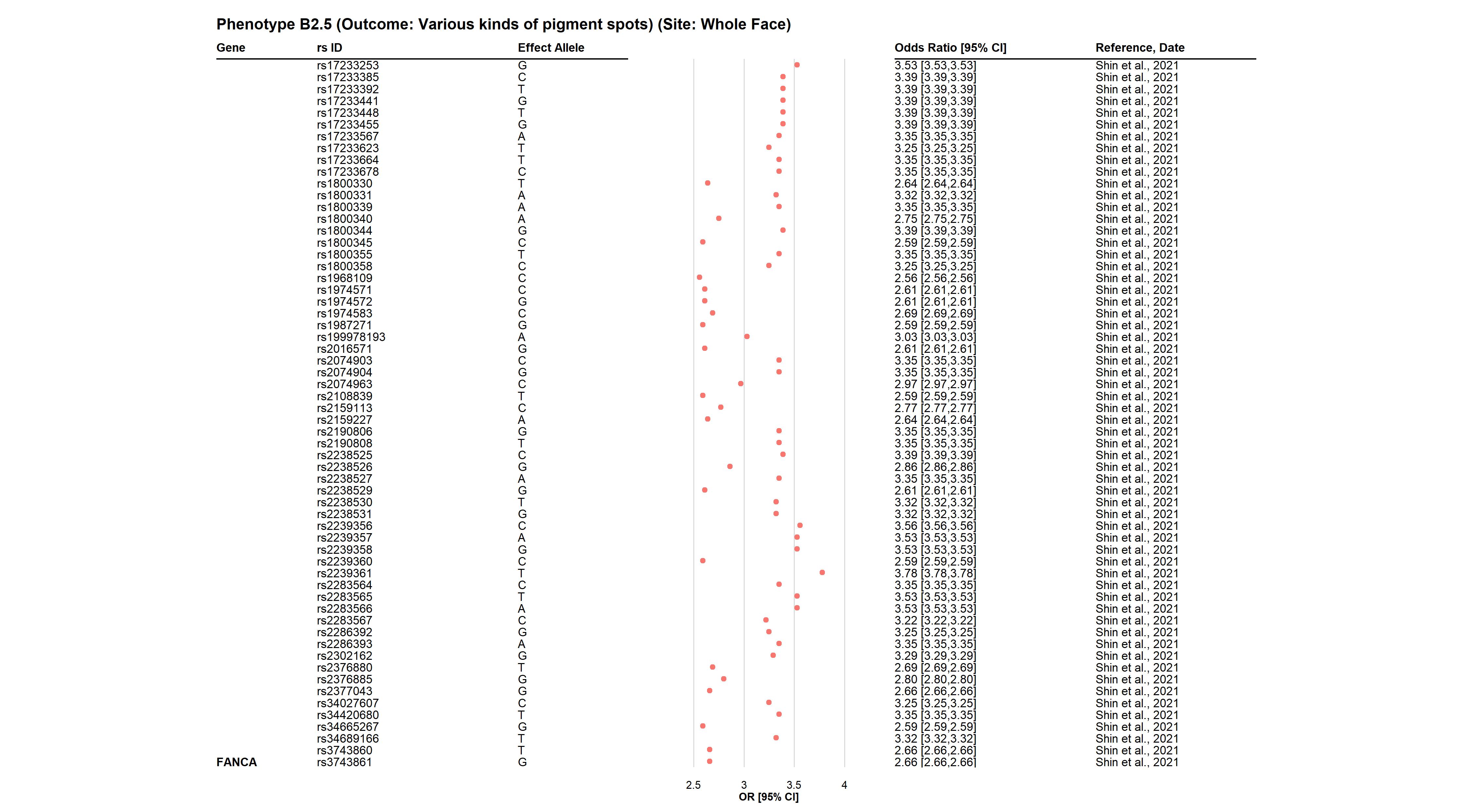

Supplement: Supplementary file 1 — Supplementary Information 1. [file 41598_2022_17443_MOESM1_ESM.zip › Supplementary Datasets/Dataset S2 - SNP-Phenotype Associations with 1 Study 1 Cohort/1 study 1 cohort Phenotype B2.5 (Outcome_Various kinds of pigment spots) (Site_Whole Face).jpg]

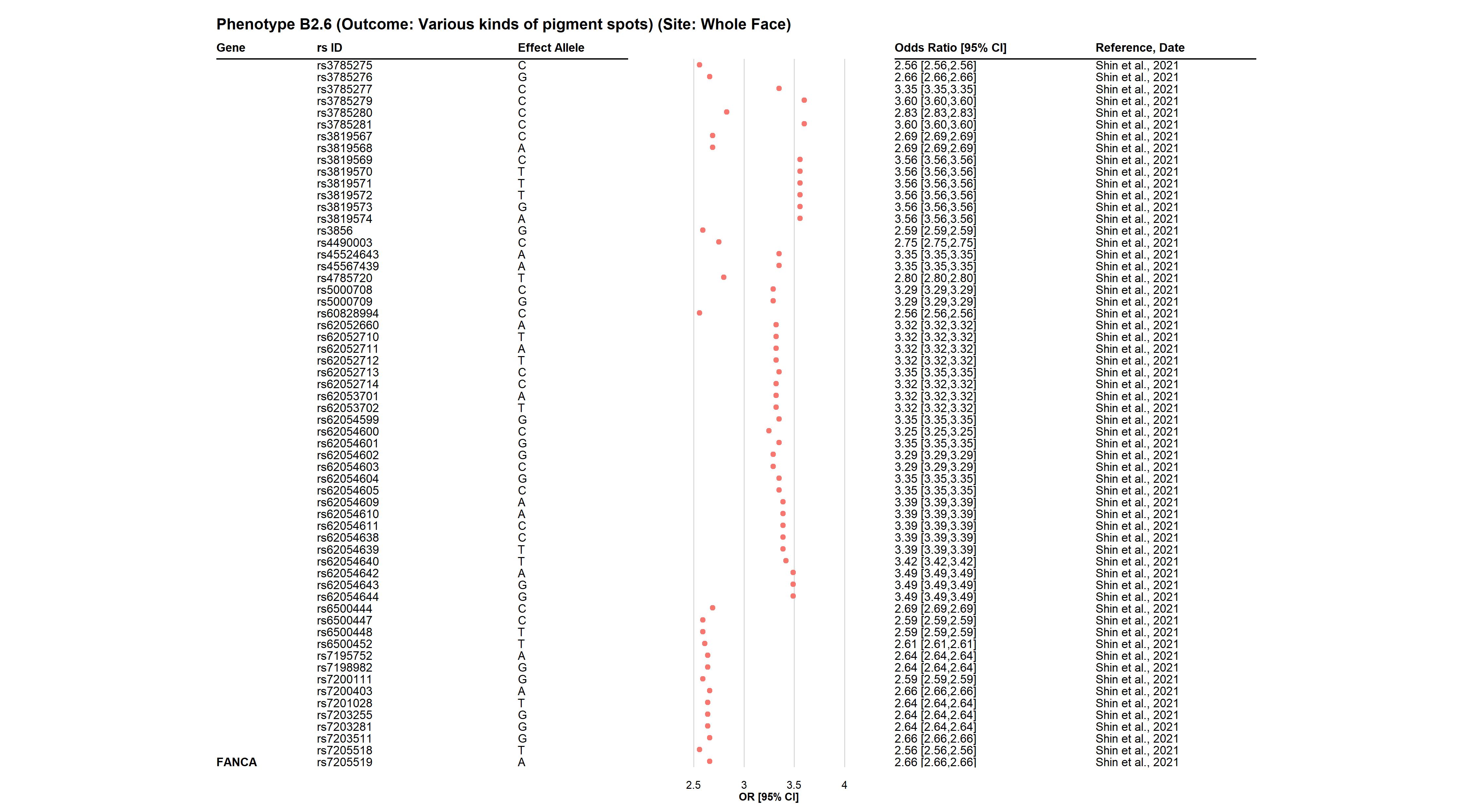

Supplement: Supplementary file 1 — Supplementary Information 1. [file 41598_2022_17443_MOESM1_ESM.zip › Supplementary Datasets/Dataset S2 - SNP-Phenotype Associations with 1 Study 1 Cohort/1 study 1 cohort Phenotype B2.6 (Outcome_Various kinds of pigment spots) (Site_Whole Face).jpg]

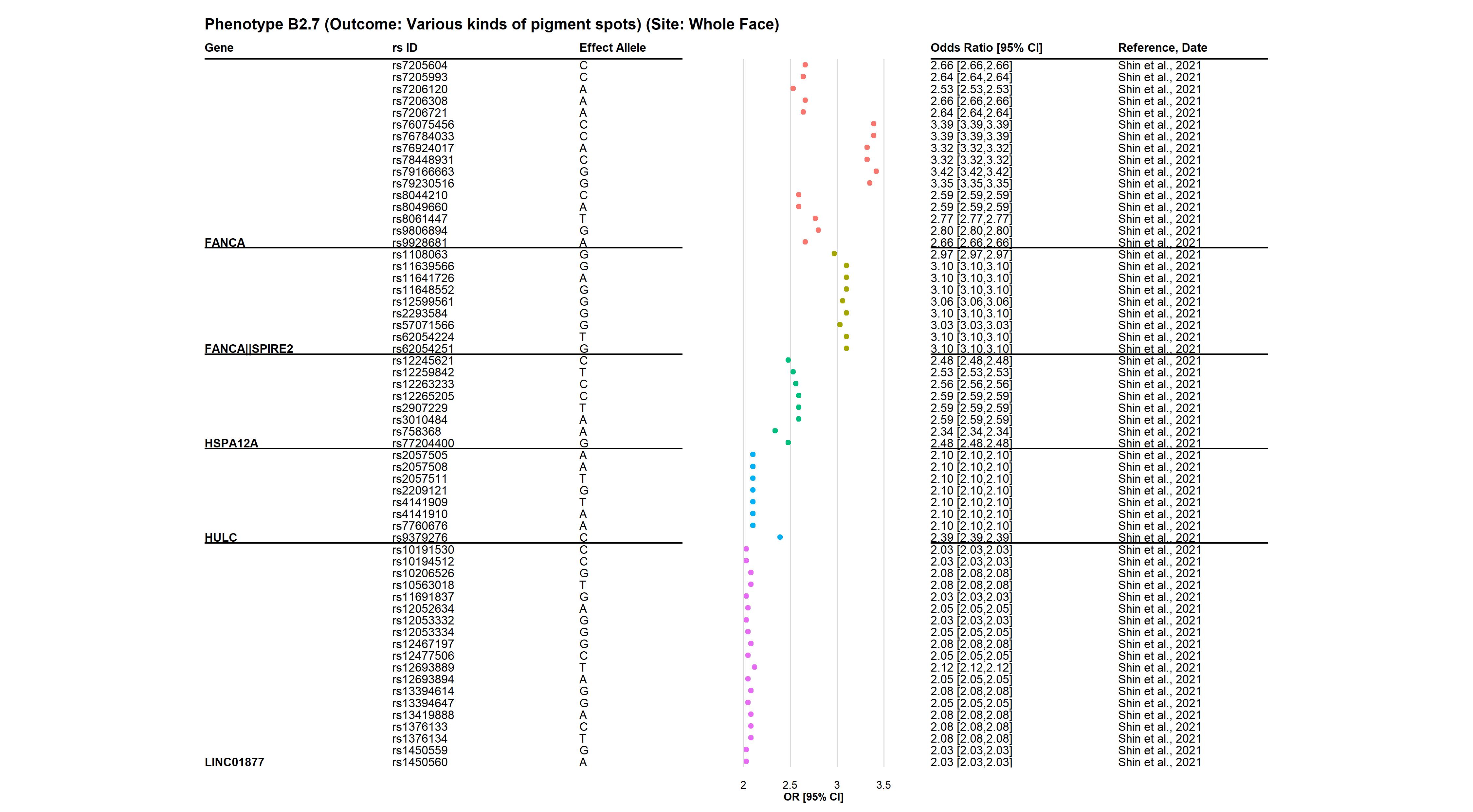

Supplement: Supplementary file 1 — Supplementary Information 1. [file 41598_2022_17443_MOESM1_ESM.zip › Supplementary Datasets/Dataset S2 - SNP-Phenotype Associations with 1 Study 1 Cohort/1 study 1 cohort Phenotype B2.7 (Outcome_Various kinds of pigment spots) (Site_Whole Face).jpg]

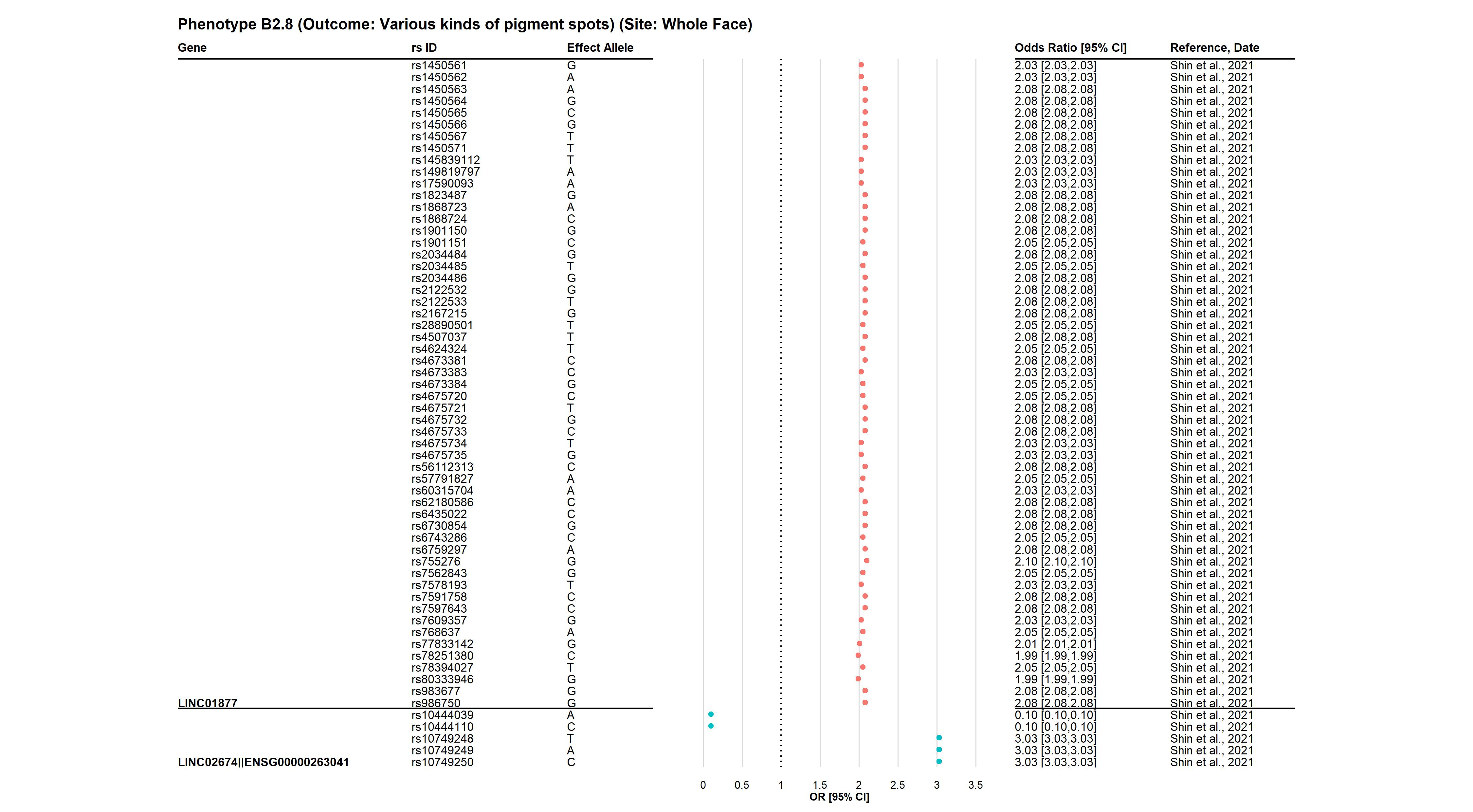

Supplement: Supplementary file 1 — Supplementary Information 1. [file 41598_2022_17443_MOESM1_ESM.zip › Supplementary Datasets/Dataset S2 - SNP-Phenotype Associations with 1 Study 1 Cohort/1 study 1 cohort Phenotype B2.8 (Outcome_Various kinds of pigment spots) (Site_Whole Face).jpg]

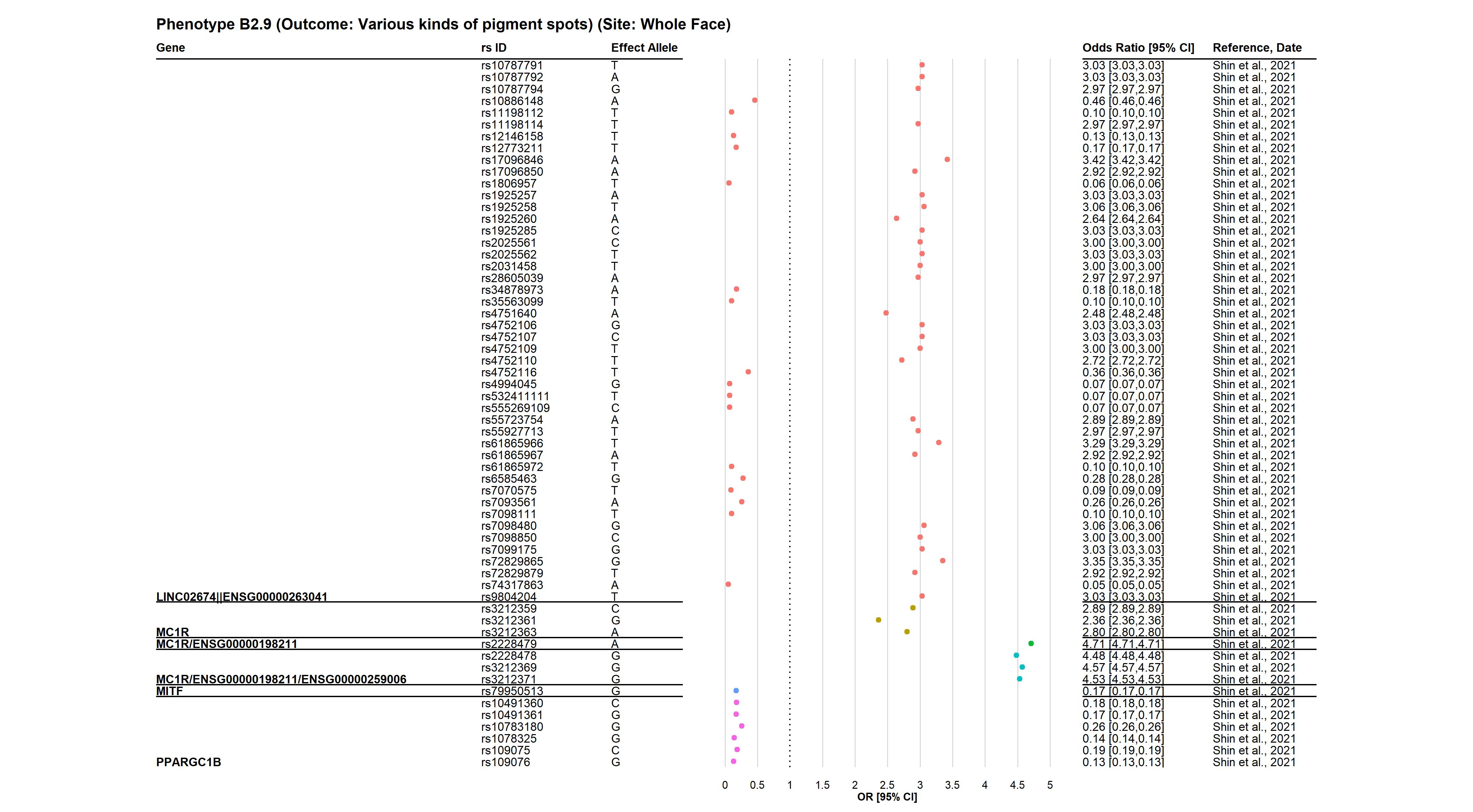

Supplement: Supplementary file 1 — Supplementary Information 1. [file 41598_2022_17443_MOESM1_ESM.zip › Supplementary Datasets/Dataset S2 - SNP-Phenotype Associations with 1 Study 1 Cohort/1 study 1 cohort Phenotype B2.9 (Outcome_Various kinds of pigment spots) (Site_Whole Face).jpg]

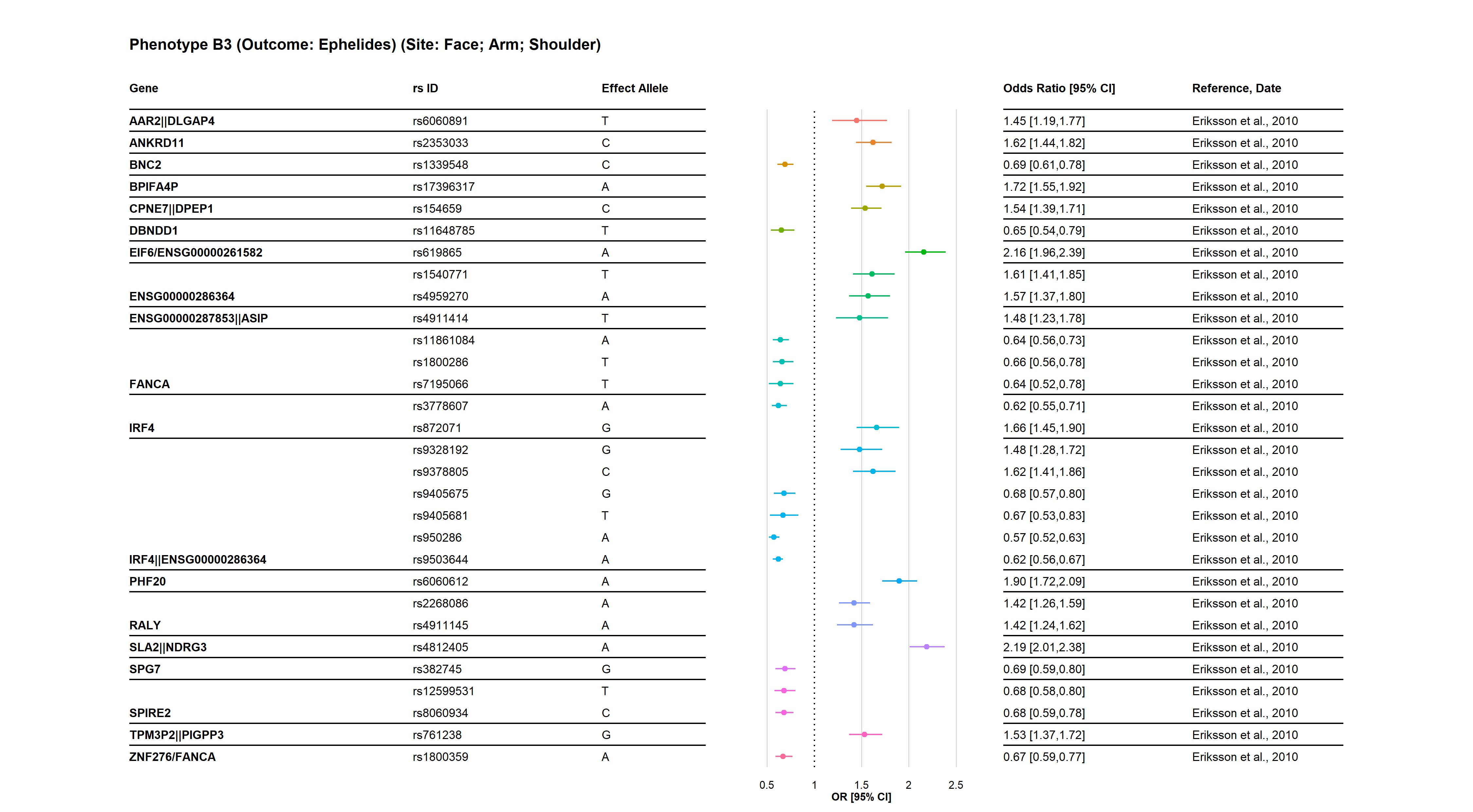

Supplement: Supplementary file 1 — Supplementary Information 1. [file 41598_2022_17443_MOESM1_ESM.zip › Supplementary Datasets/Dataset S2 - SNP-Phenotype Associations with 1 Study 1 Cohort/1 study 1 cohort Phenotype B3.1 (Outcome_Ephelides) (Site_Face_ Arm_ Shoulder).jpg]

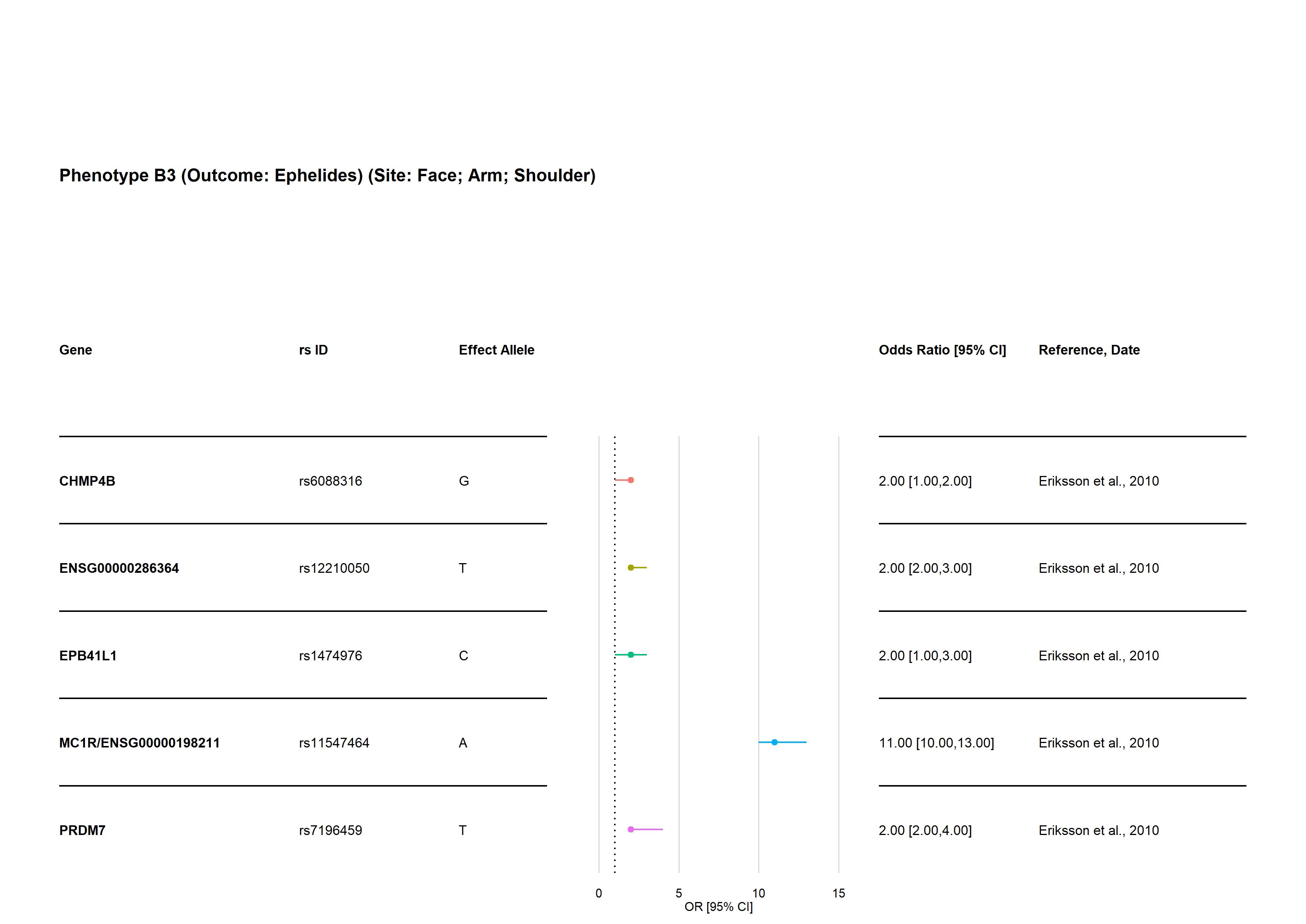

Supplement: Supplementary file 1 — Supplementary Information 1. [file 41598_2022_17443_MOESM1_ESM.zip › Supplementary Datasets/Dataset S2 - SNP-Phenotype Associations with 1 Study 1 Cohort/1 study 1 cohort Phenotype B3.2 (Outcome_Ephelides) (Site_Face_ Arm_ Shoulder).jpg]

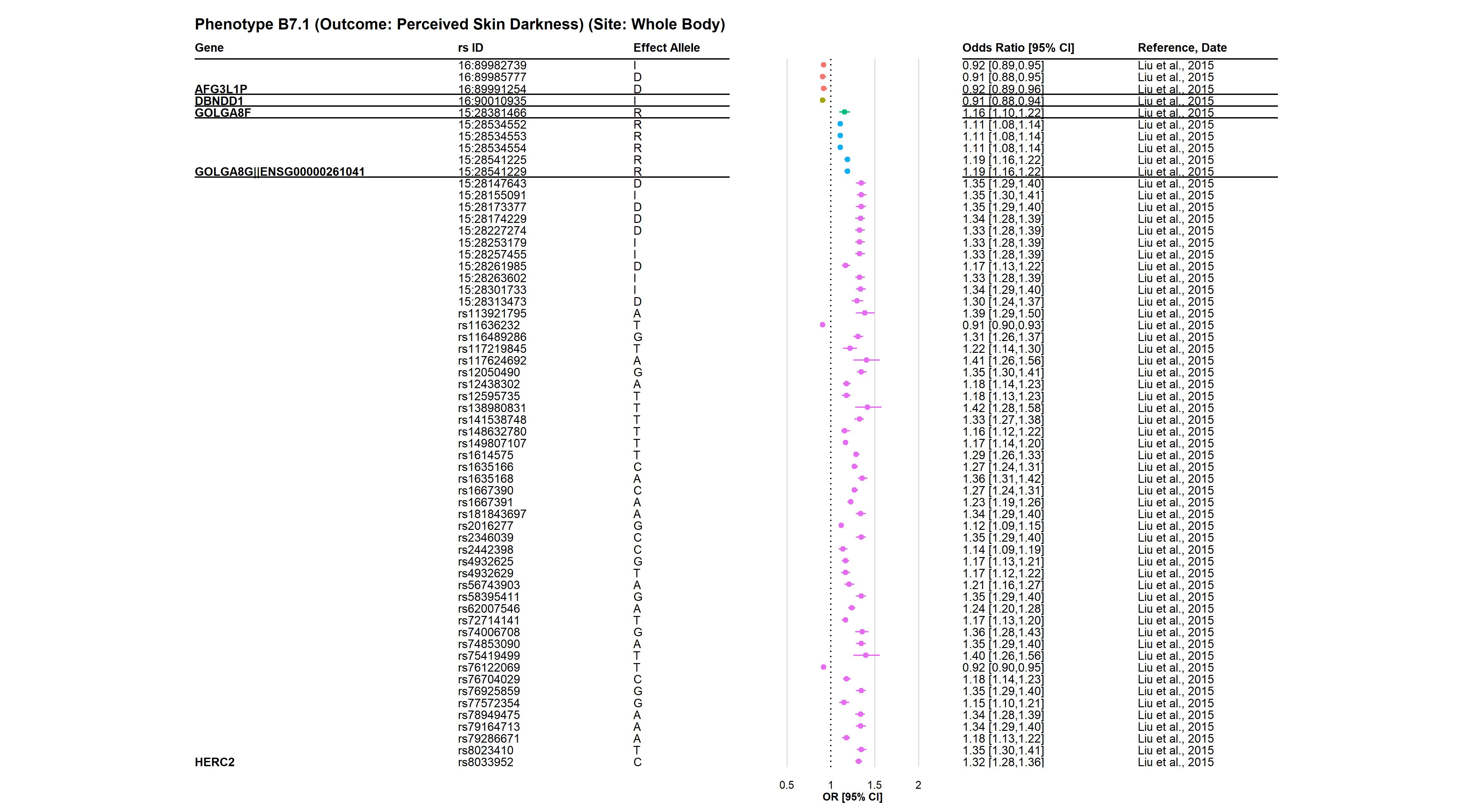

Supplement: Supplementary file 1 — Supplementary Information 1. [file 41598_2022_17443_MOESM1_ESM.zip › Supplementary Datasets/Dataset S2 - SNP-Phenotype Associations with 1 Study 1 Cohort/1 study 1 cohort Phenotype B7.1 (Outcome_Perceived Skin Darkness) (Site_Whole Body).jpg]

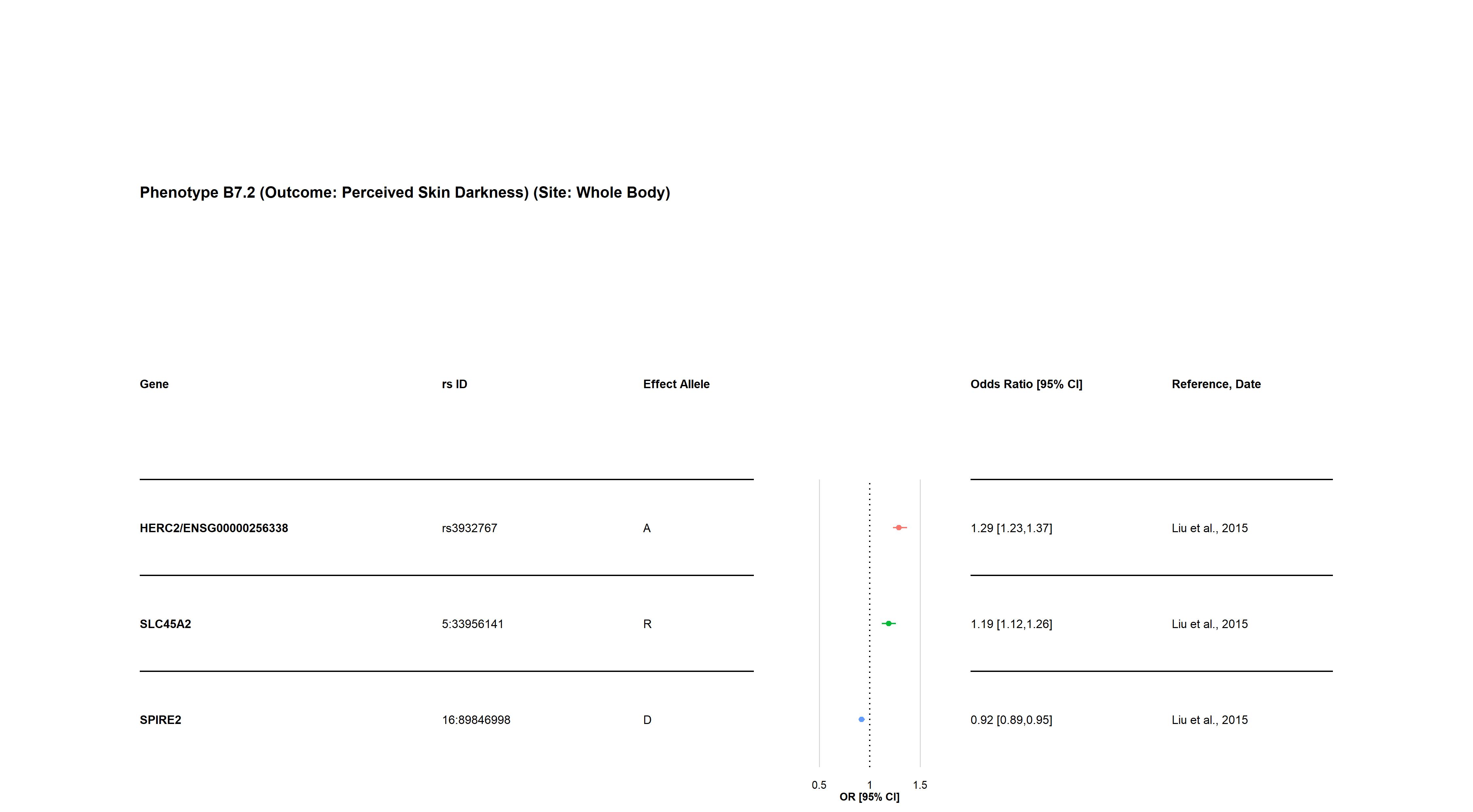

Supplement: Supplementary file 1 — Supplementary Information 1. [file 41598_2022_17443_MOESM1_ESM.zip › Supplementary Datasets/Dataset S2 - SNP-Phenotype Associations with 1 Study 1 Cohort/1 study 1 cohort Phenotype B7.2 (Outcome_Perceived Skin Darkness) (Site_Whole Body).jpg]

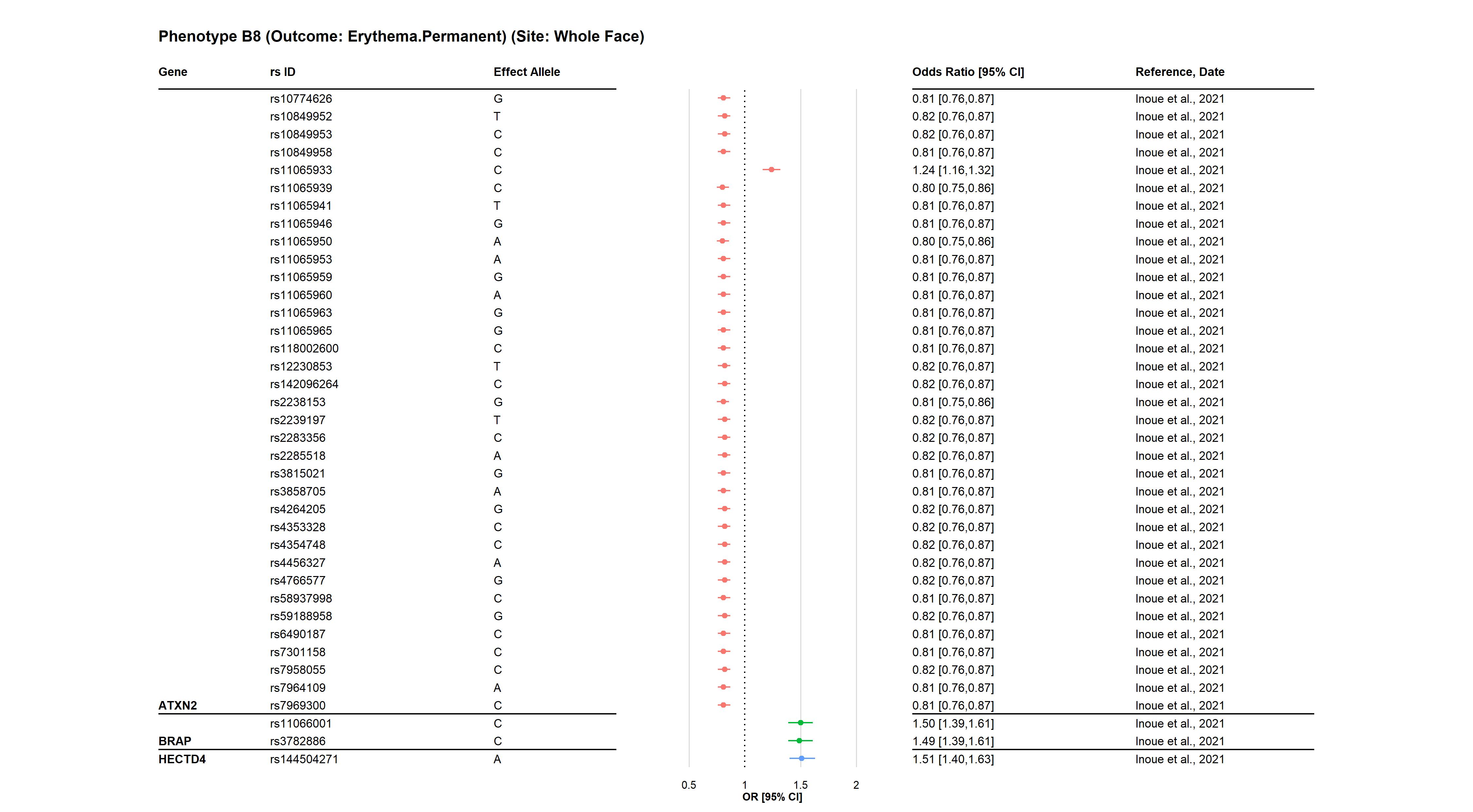

Supplement: Supplementary file 1 — Supplementary Information 1. [file 41598_2022_17443_MOESM1_ESM.zip › Supplementary Datasets/Dataset S2 - SNP-Phenotype Associations with 1 Study 1 Cohort/1 study 1 cohort Phenotype B8 (Outcome_Erythema.Permanent) (Site_Whole Face).jpg]

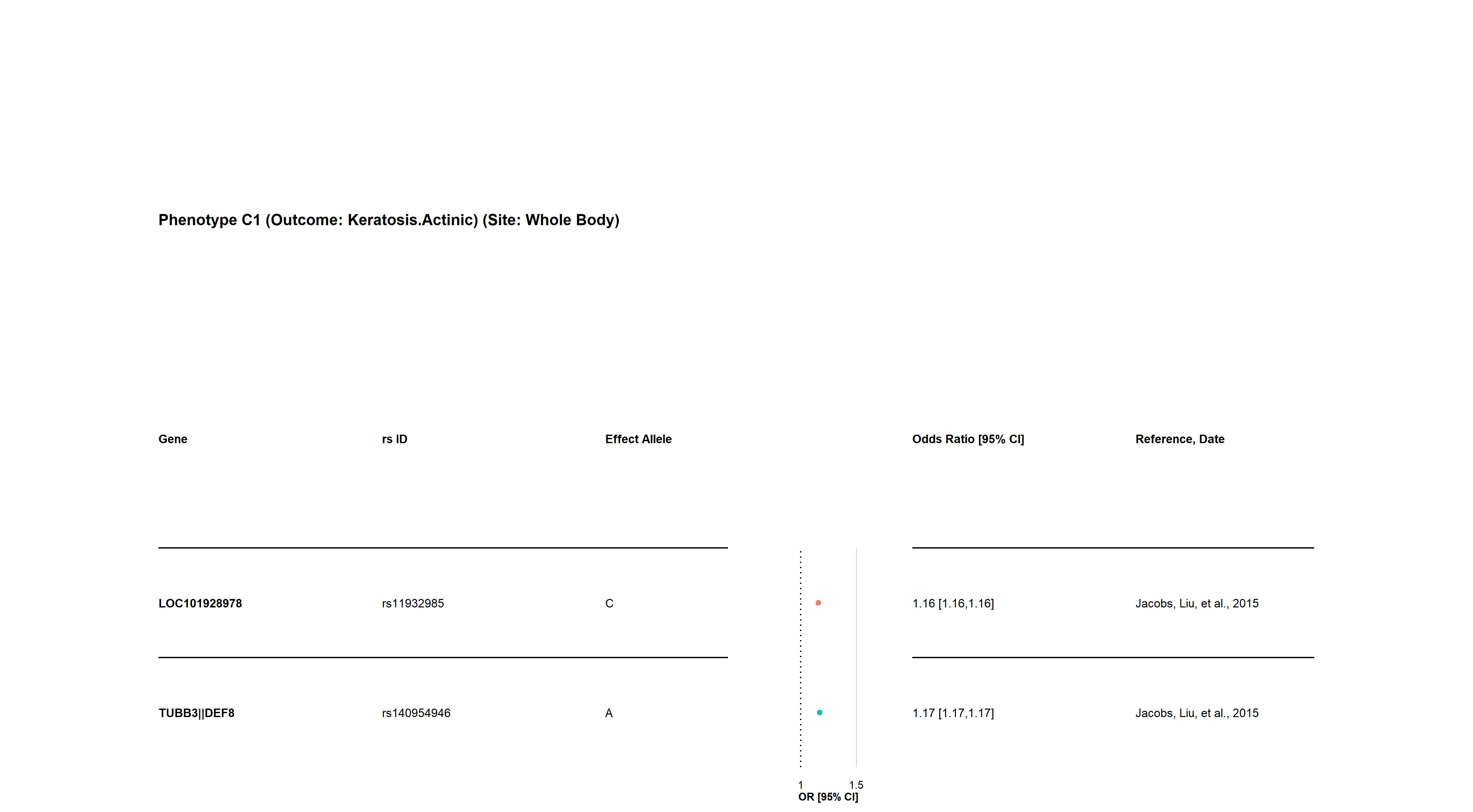

Supplement: Supplementary file 1 — Supplementary Information 1. [file 41598_2022_17443_MOESM1_ESM.zip › Supplementary Datasets/Dataset S2 - SNP-Phenotype Associations with 1 Study 1 Cohort/1 study 1 cohort Phenotype C1 (Outcome_Keratosis.Actinic) (Site_Whole Body).jpg]

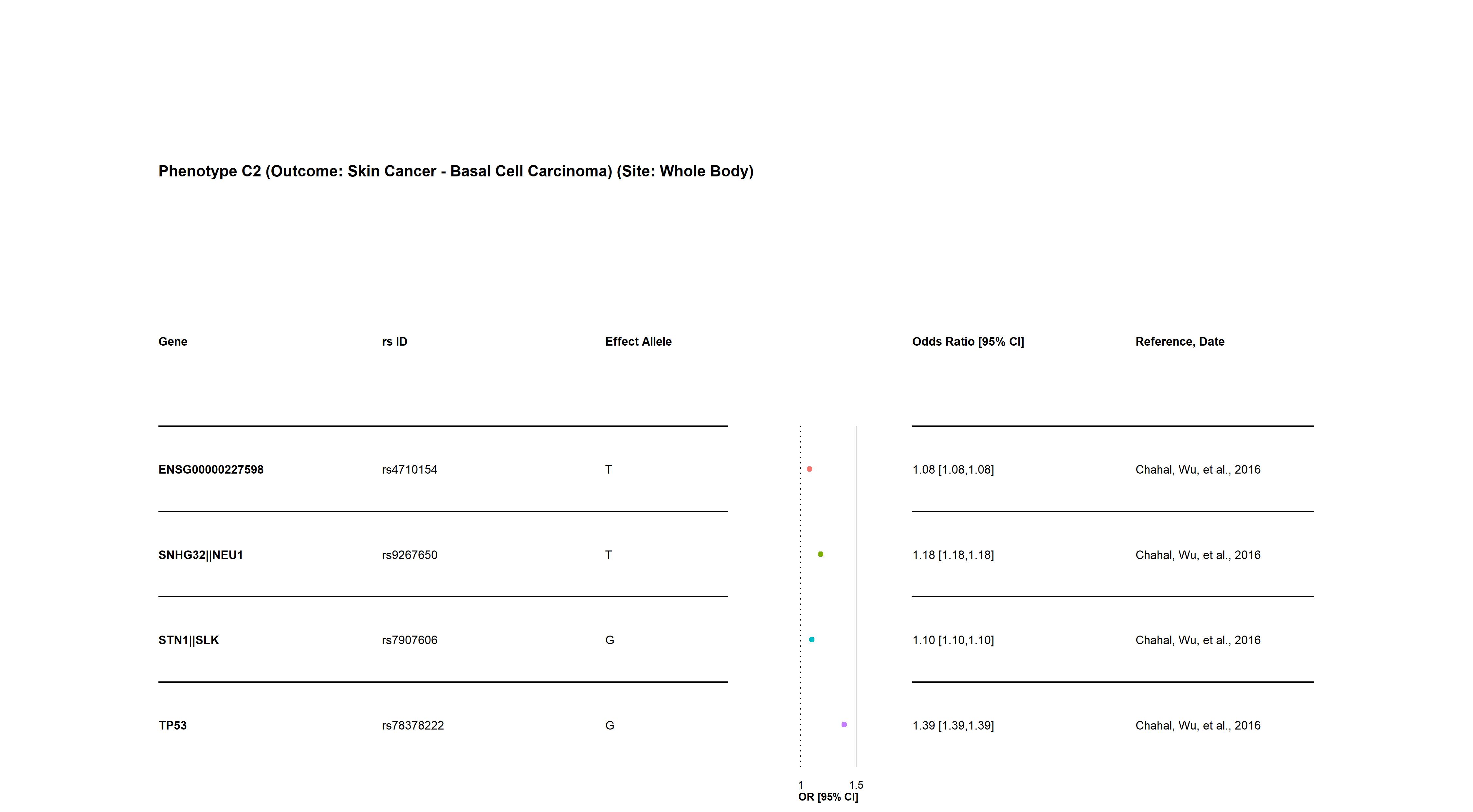

Supplement: Supplementary file 1 — Supplementary Information 1. [file 41598_2022_17443_MOESM1_ESM.zip › Supplementary Datasets/Dataset S2 - SNP-Phenotype Associations with 1 Study 1 Cohort/1 study 1 cohort Phenotype C2.1 (Outcome_Skin Cancer - Basal Cell Carcinoma) (Site_Whole Body).jpg]

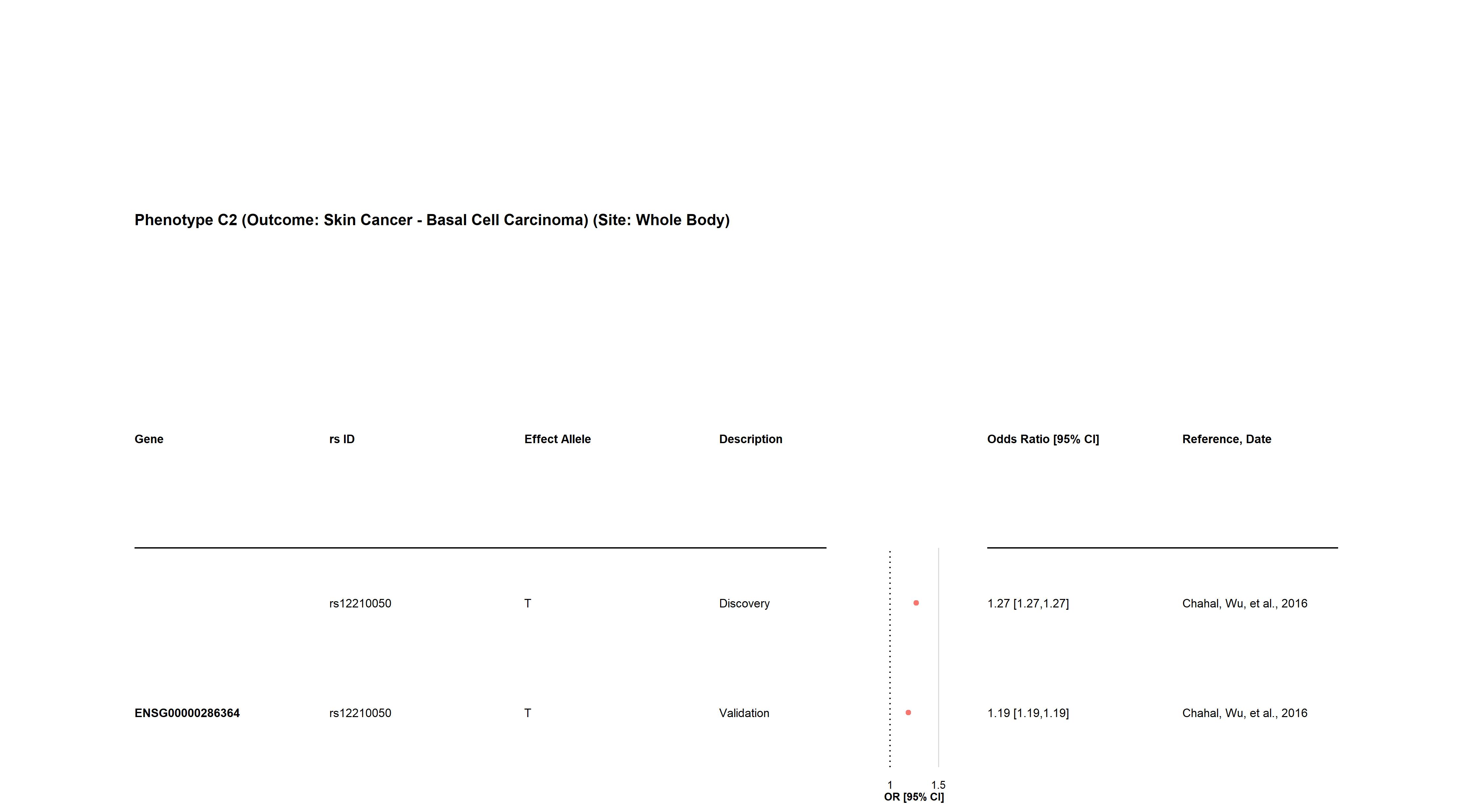

Supplement: Supplementary file 1 — Supplementary Information 1. [file 41598_2022_17443_MOESM1_ESM.zip › Supplementary Datasets/Dataset S2 - SNP-Phenotype Associations with 1 Study 1 Cohort/1 study 1 cohort Phenotype C2.2 (Outcome_Skin Cancer - Basal Cell Carcinoma) (Site_Whole Body).jpg]

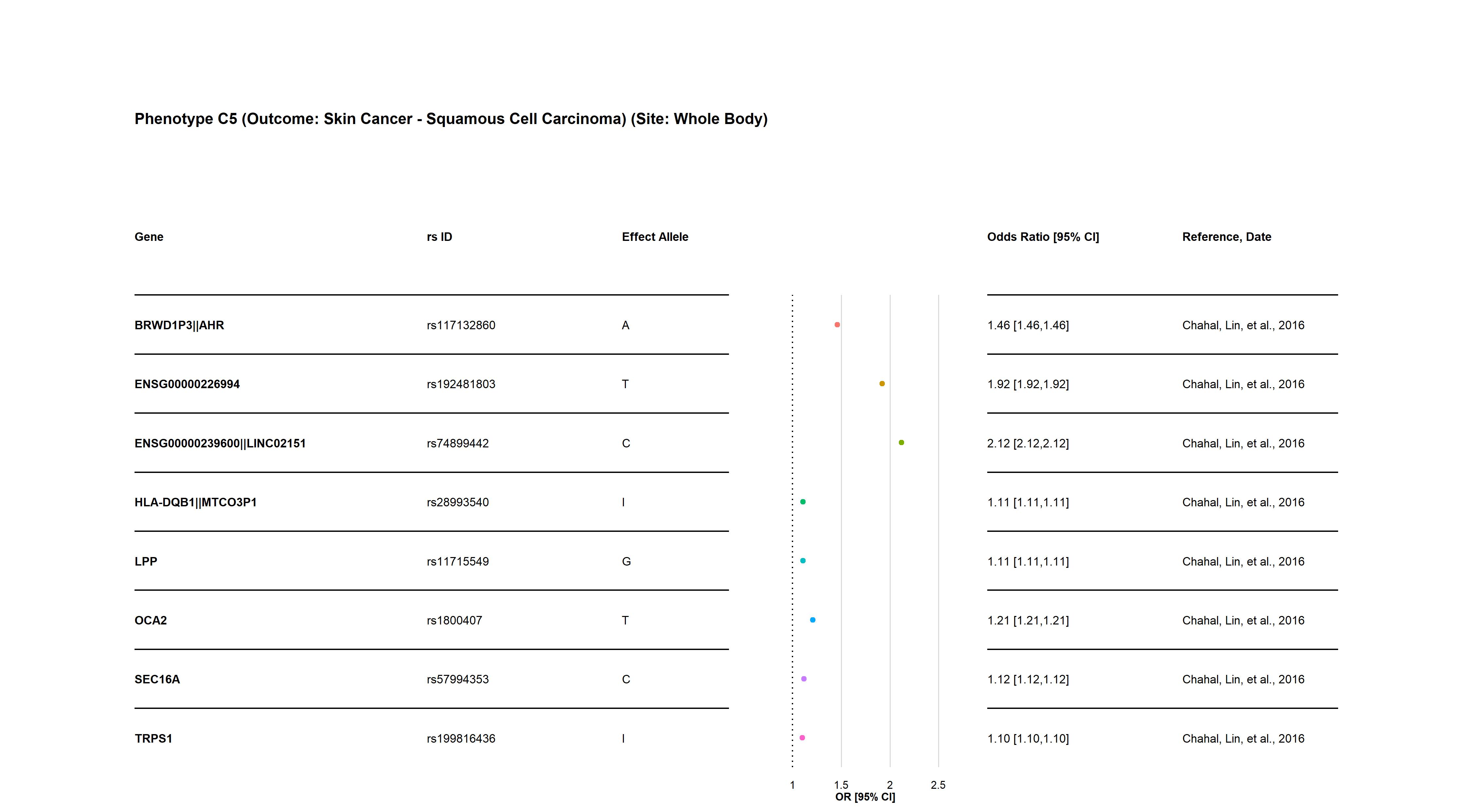

Supplement: Supplementary file 1 — Supplementary Information 1. [file 41598_2022_17443_MOESM1_ESM.zip › Supplementary Datasets/Dataset S2 - SNP-Phenotype Associations with 1 Study 1 Cohort/1 study 1 cohort Phenotype C5 (Outcome_Skin Cancer - Squamous Cell Carcinoma) (Site_Whole Body).jpg]

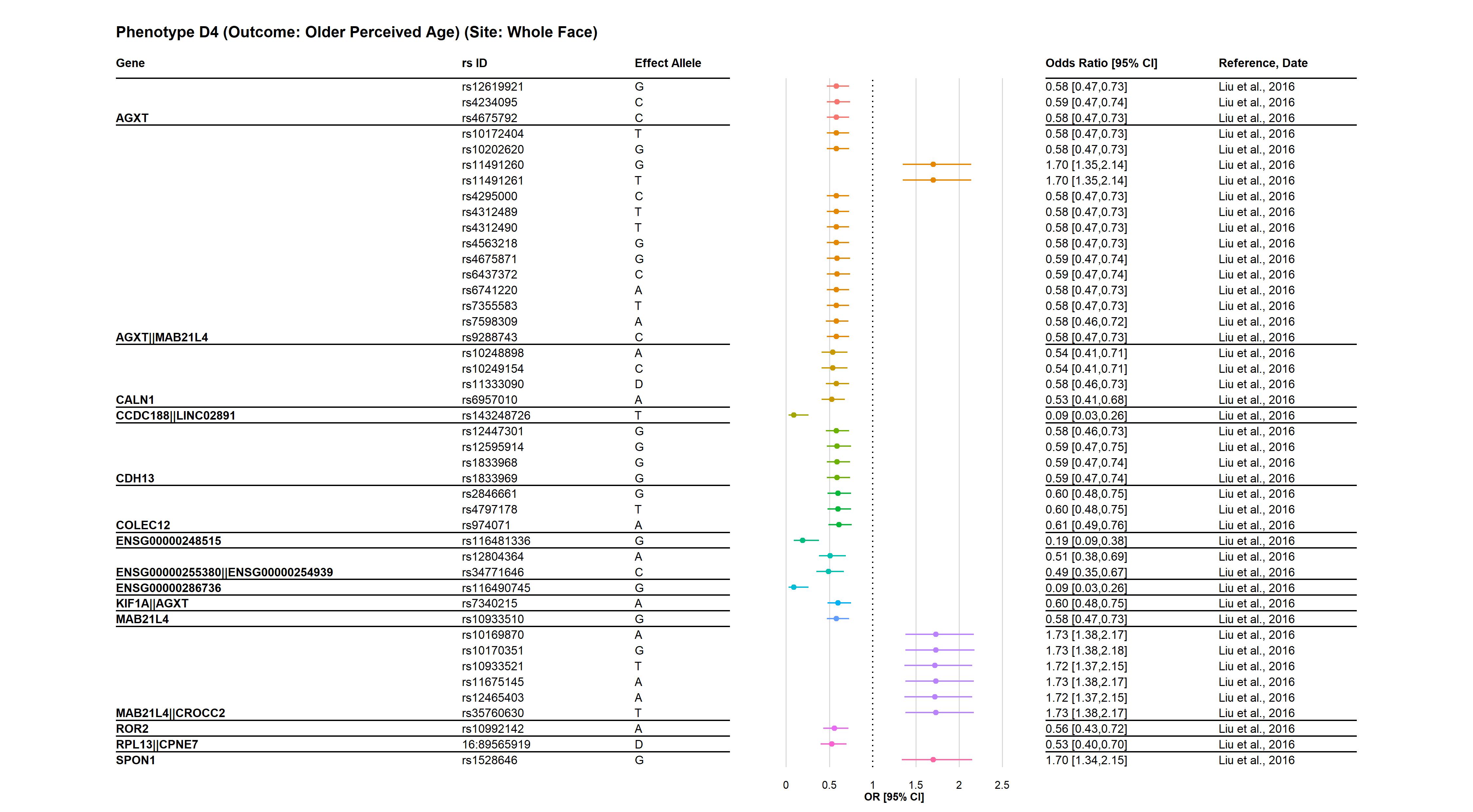

Supplement: Supplementary file 1 — Supplementary Information 1. [file 41598_2022_17443_MOESM1_ESM.zip › Supplementary Datasets/Dataset S2 - SNP-Phenotype Associations with 1 Study 1 Cohort/1 study 1 cohort Phenotype D4.1 (Outcome_Older Perceived Age) (Site_Whole Face).jpg]

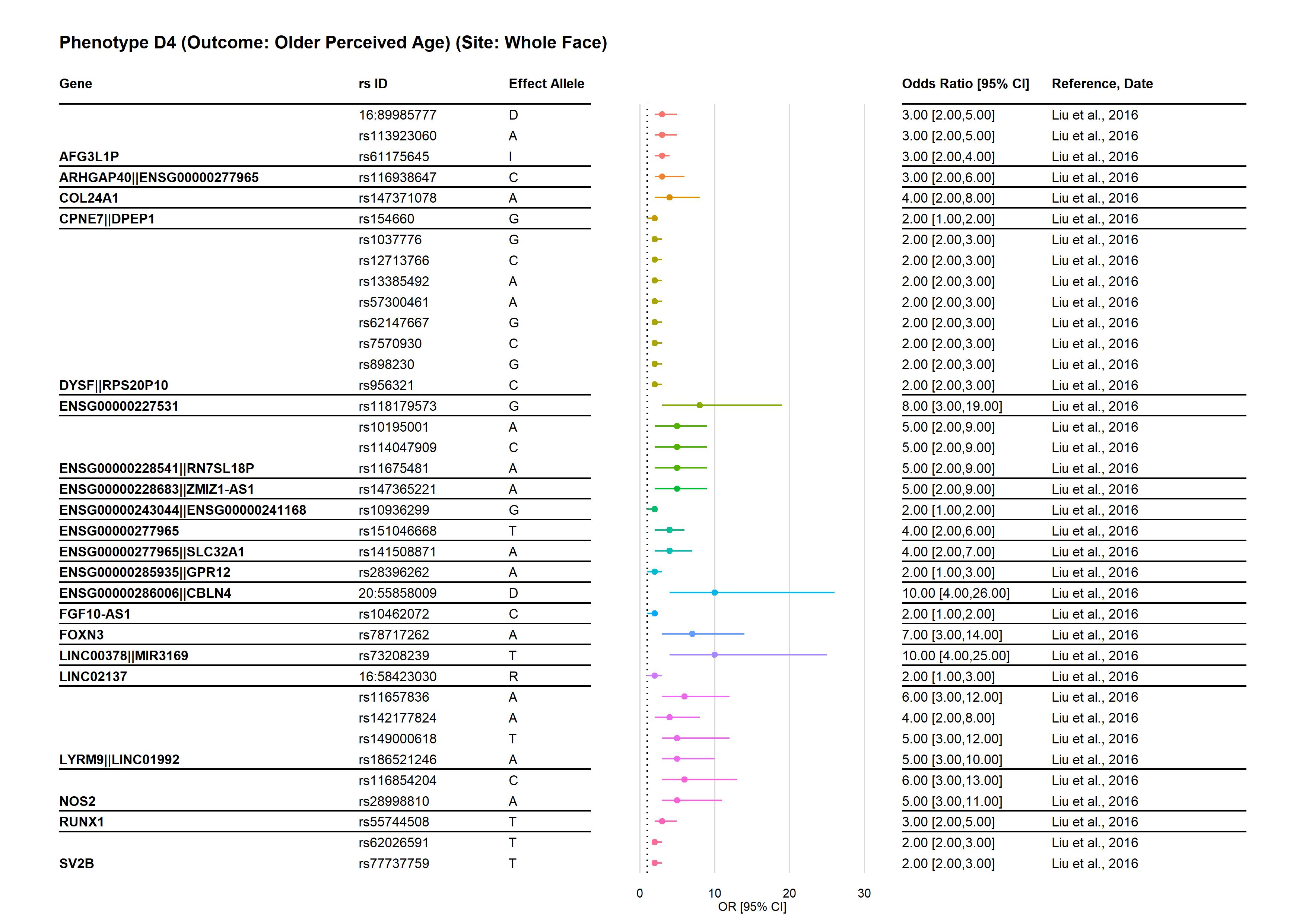

Supplement: Supplementary file 1 — Supplementary Information 1. [file 41598_2022_17443_MOESM1_ESM.zip › Supplementary Datasets/Dataset S2 - SNP-Phenotype Associations with 1 Study 1 Cohort/1 study 1 cohort Phenotype D4.2 (Outcome_Older Perceived Age) (Site_Whole Face).jpg]

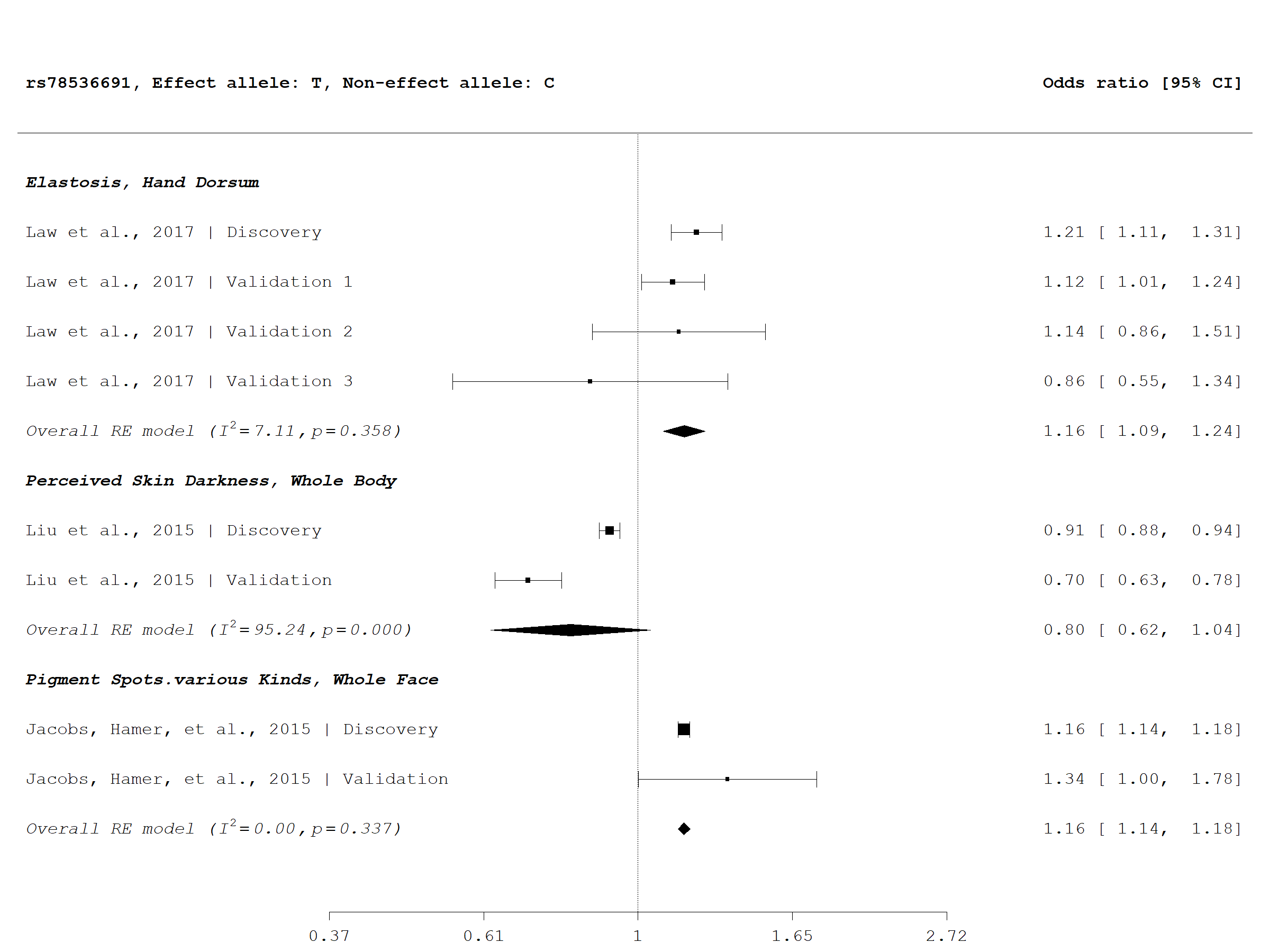

Supplement: Supplementary file 1 — Supplementary Information 1. [file 41598_2022_17443_MOESM1_ESM.zip › Supplementary Datasets/Dataset S3 - Forest Plots/fp100_rs78536691.png]

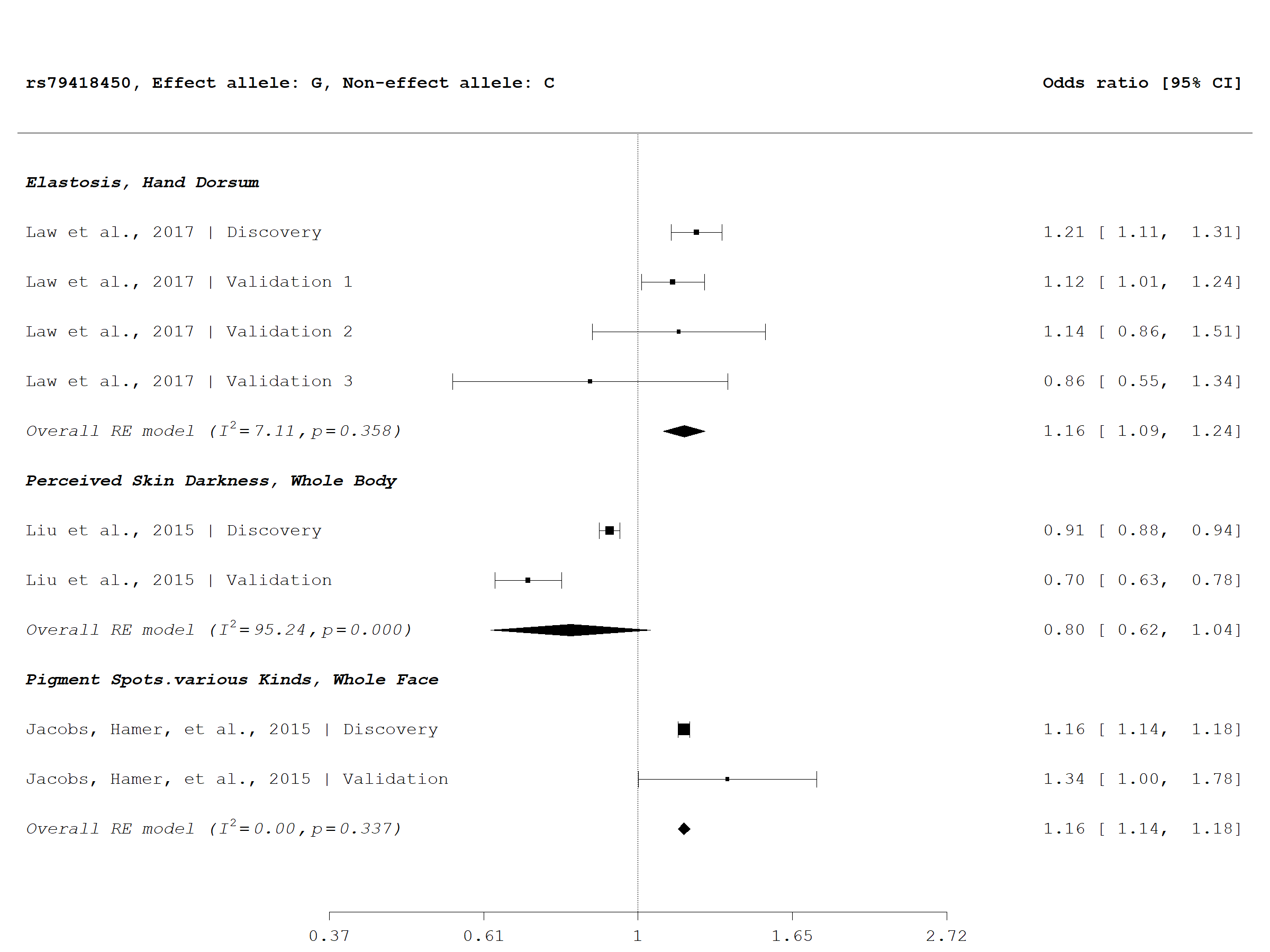

Supplement: Supplementary file 1 — Supplementary Information 1. [file 41598_2022_17443_MOESM1_ESM.zip › Supplementary Datasets/Dataset S3 - Forest Plots/fp101_rs79418450.png]

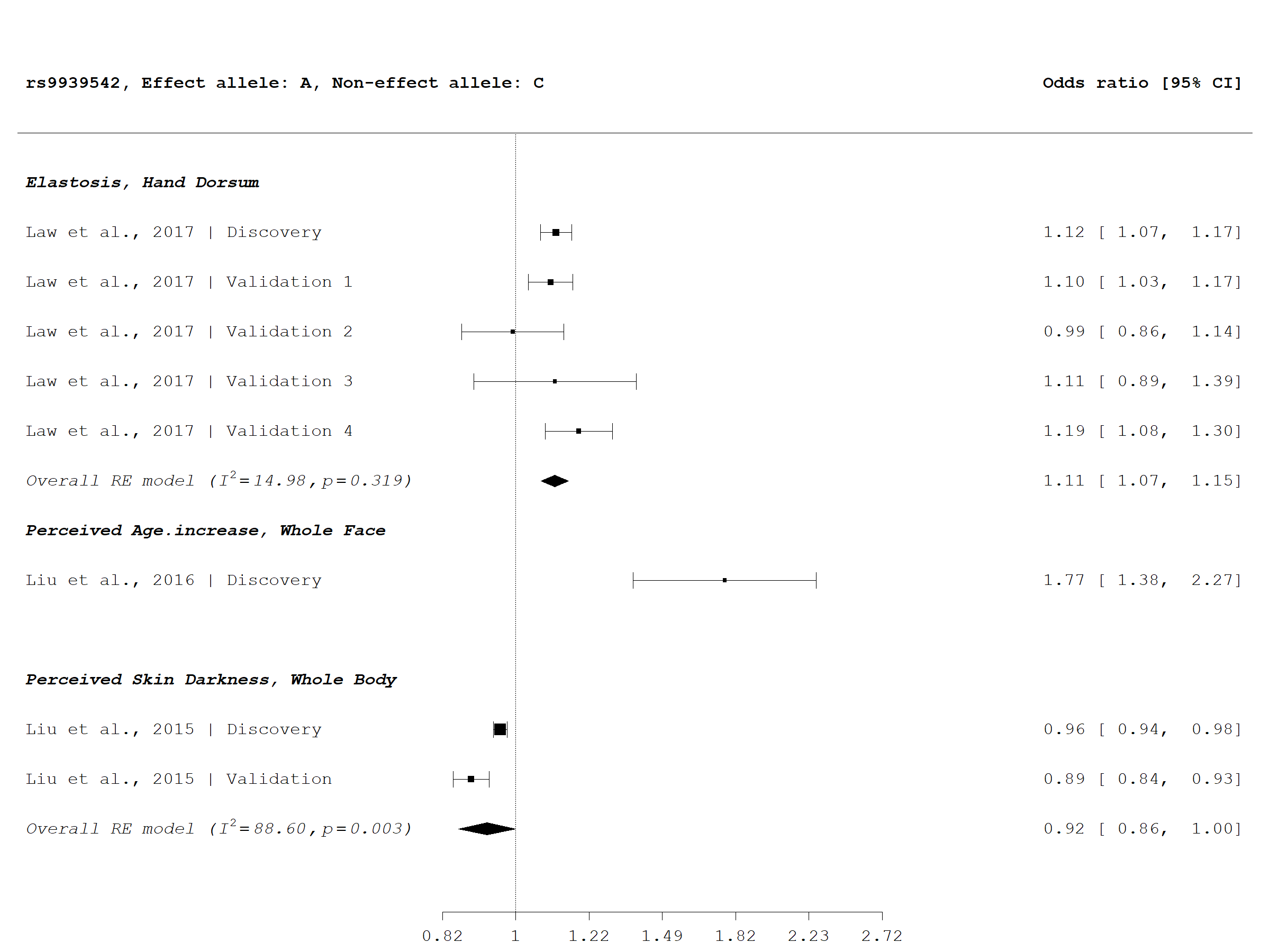

Supplement: Supplementary file 1 — Supplementary Information 1. [file 41598_2022_17443_MOESM1_ESM.zip › Supplementary Datasets/Dataset S3 - Forest Plots/fp102_rs9939542.png]

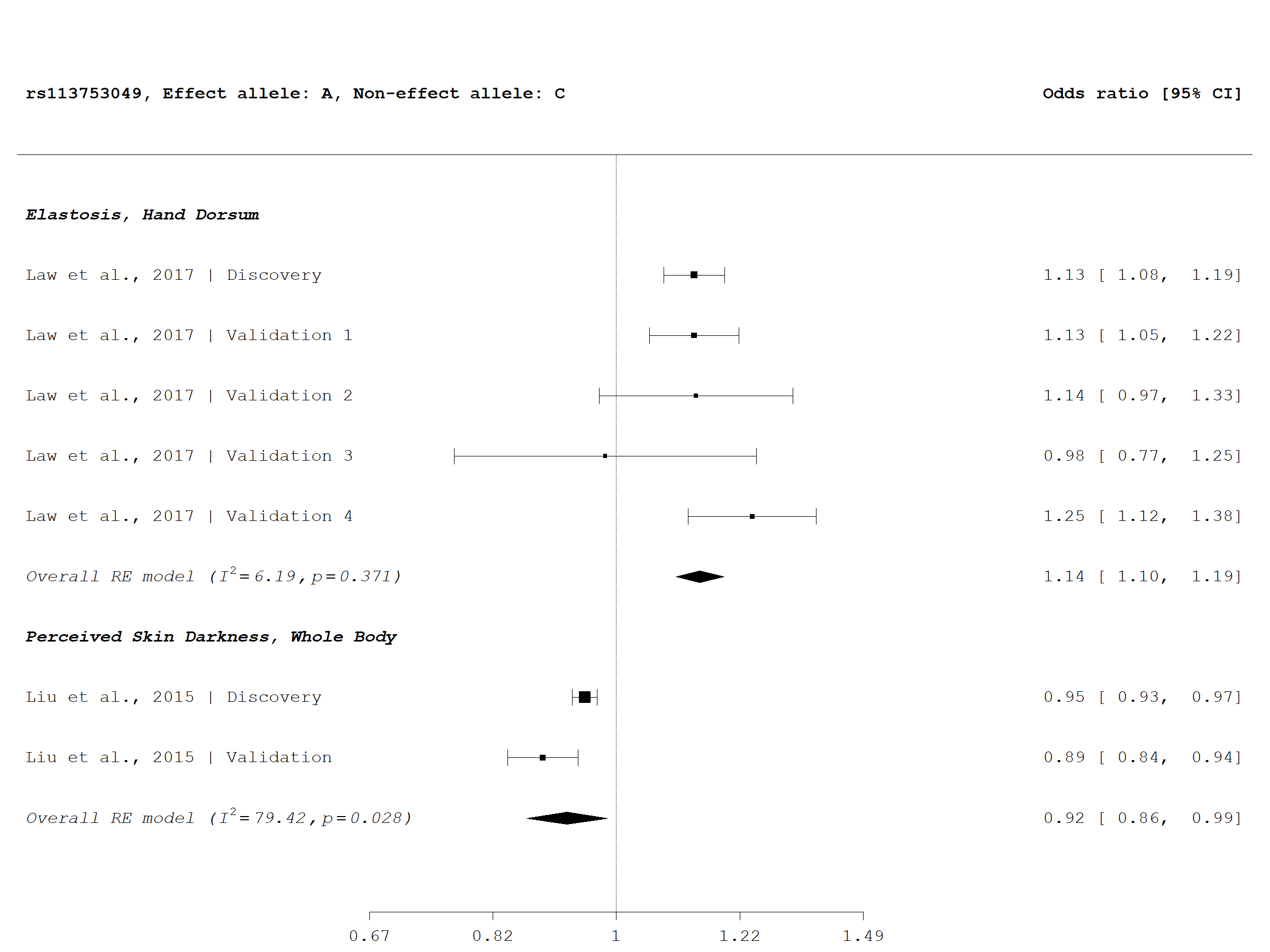

Supplement: Supplementary file 1 — Supplementary Information 1. [file 41598_2022_17443_MOESM1_ESM.zip › Supplementary Datasets/Dataset S3 - Forest Plots/fp103_rs113753049.png]

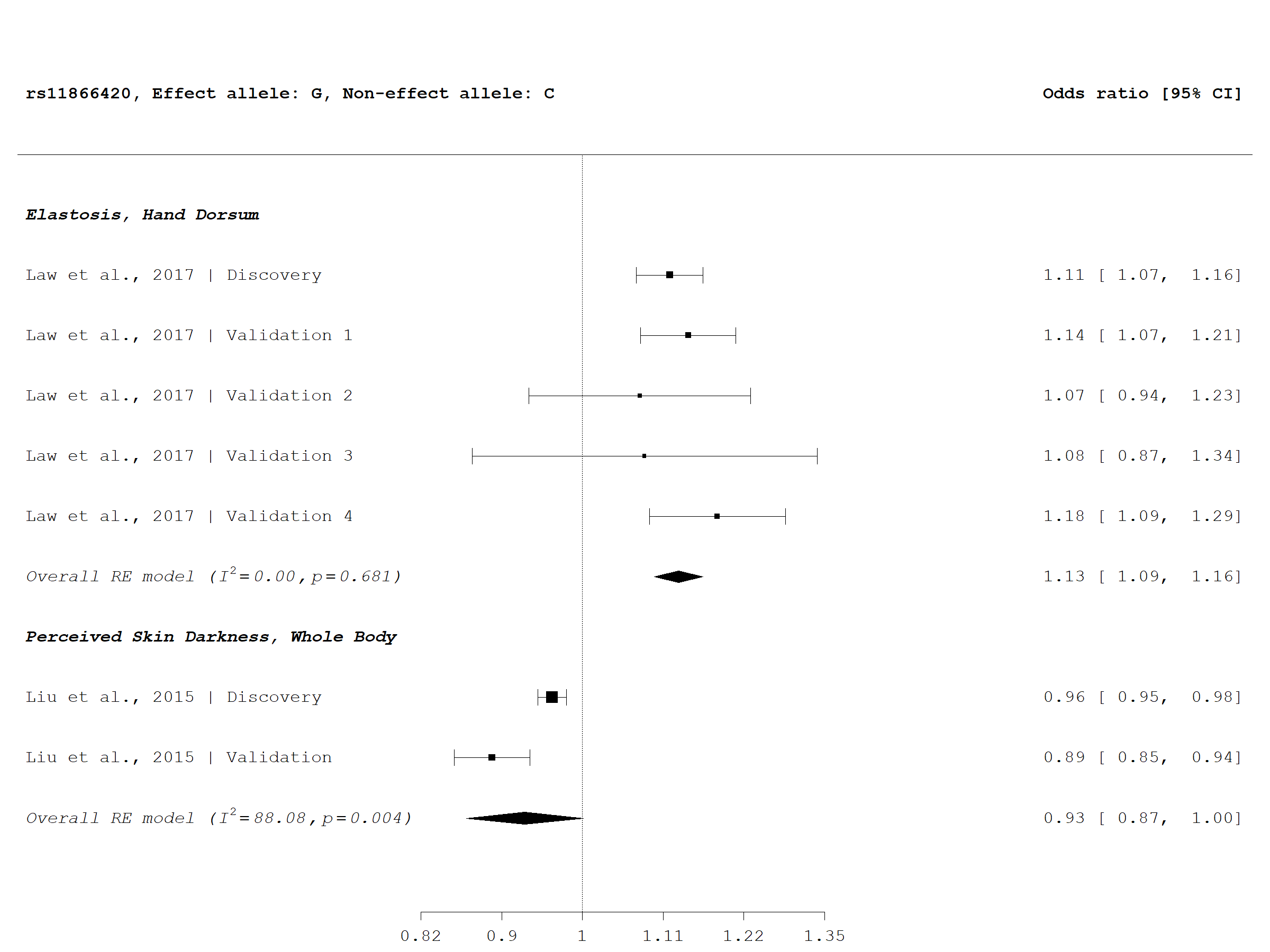

Supplement: Supplementary file 1 — Supplementary Information 1. [file 41598_2022_17443_MOESM1_ESM.zip › Supplementary Datasets/Dataset S3 - Forest Plots/fp104_rs11866420.png]

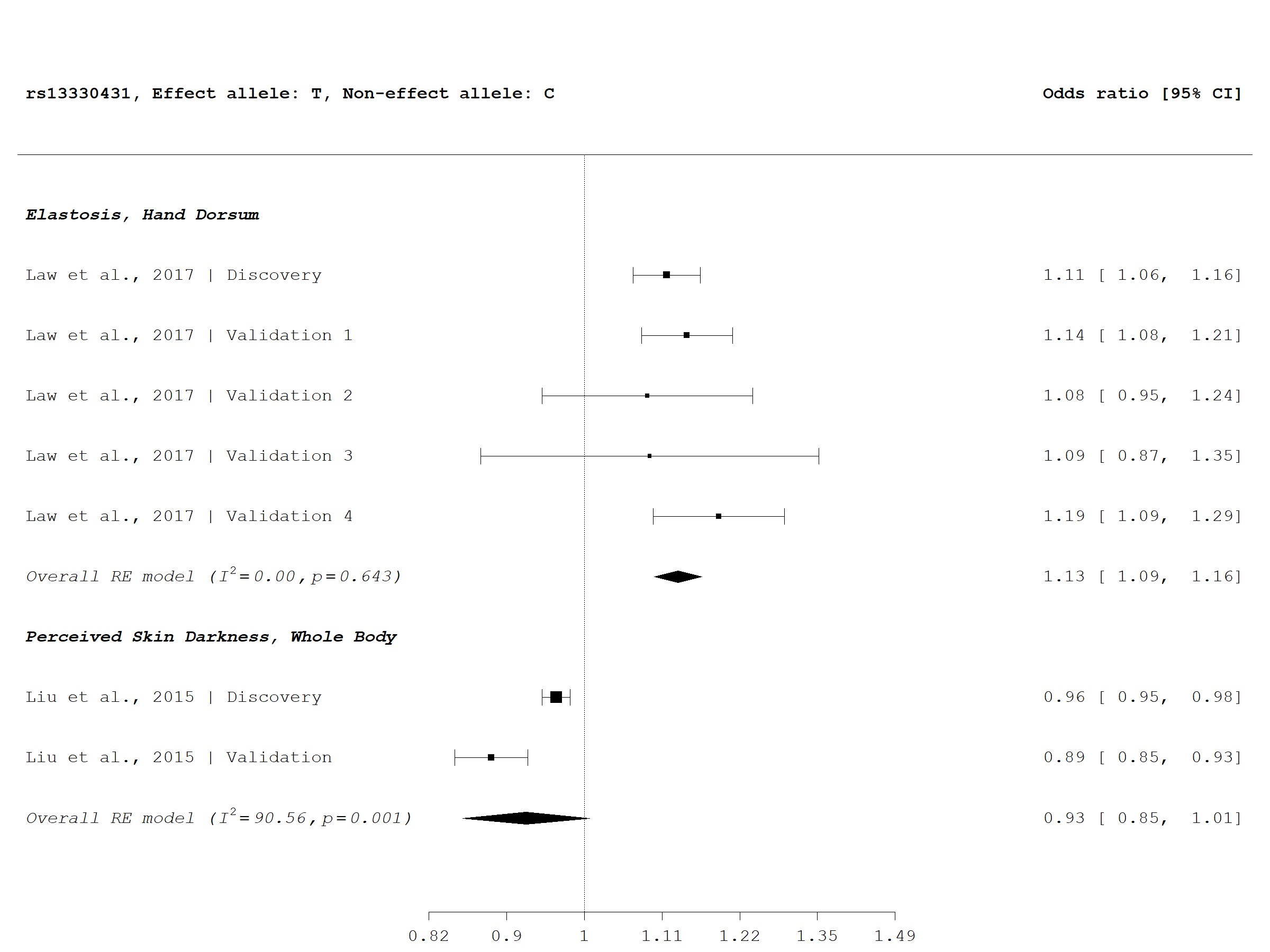

Supplement: Supplementary file 1 — Supplementary Information 1. [file 41598_2022_17443_MOESM1_ESM.zip › Supplementary Datasets/Dataset S3 - Forest Plots/fp105_rs13330431.png]

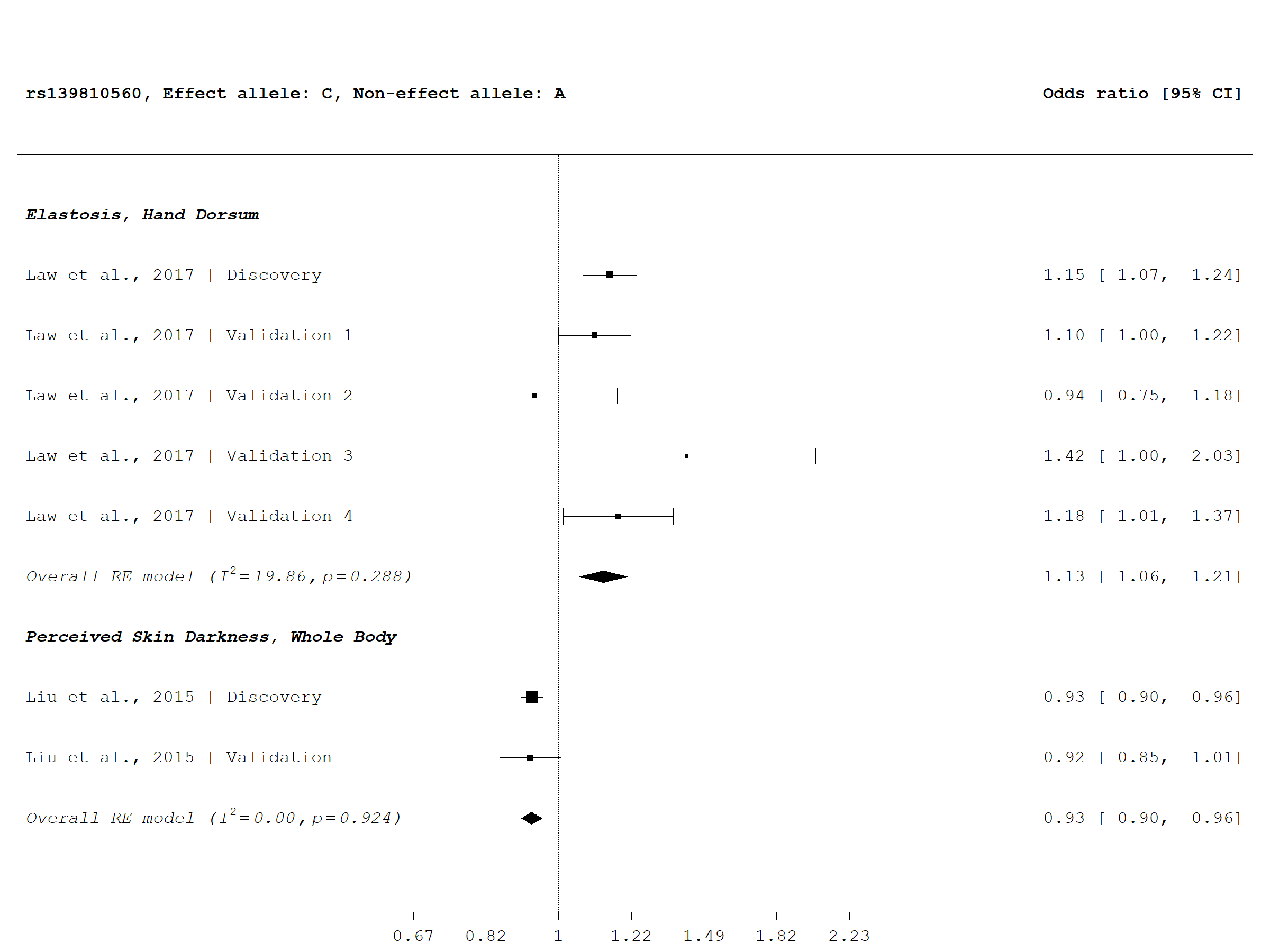

Supplement: Supplementary file 1 — Supplementary Information 1. [file 41598_2022_17443_MOESM1_ESM.zip › Supplementary Datasets/Dataset S3 - Forest Plots/fp106_rs139810560.png]

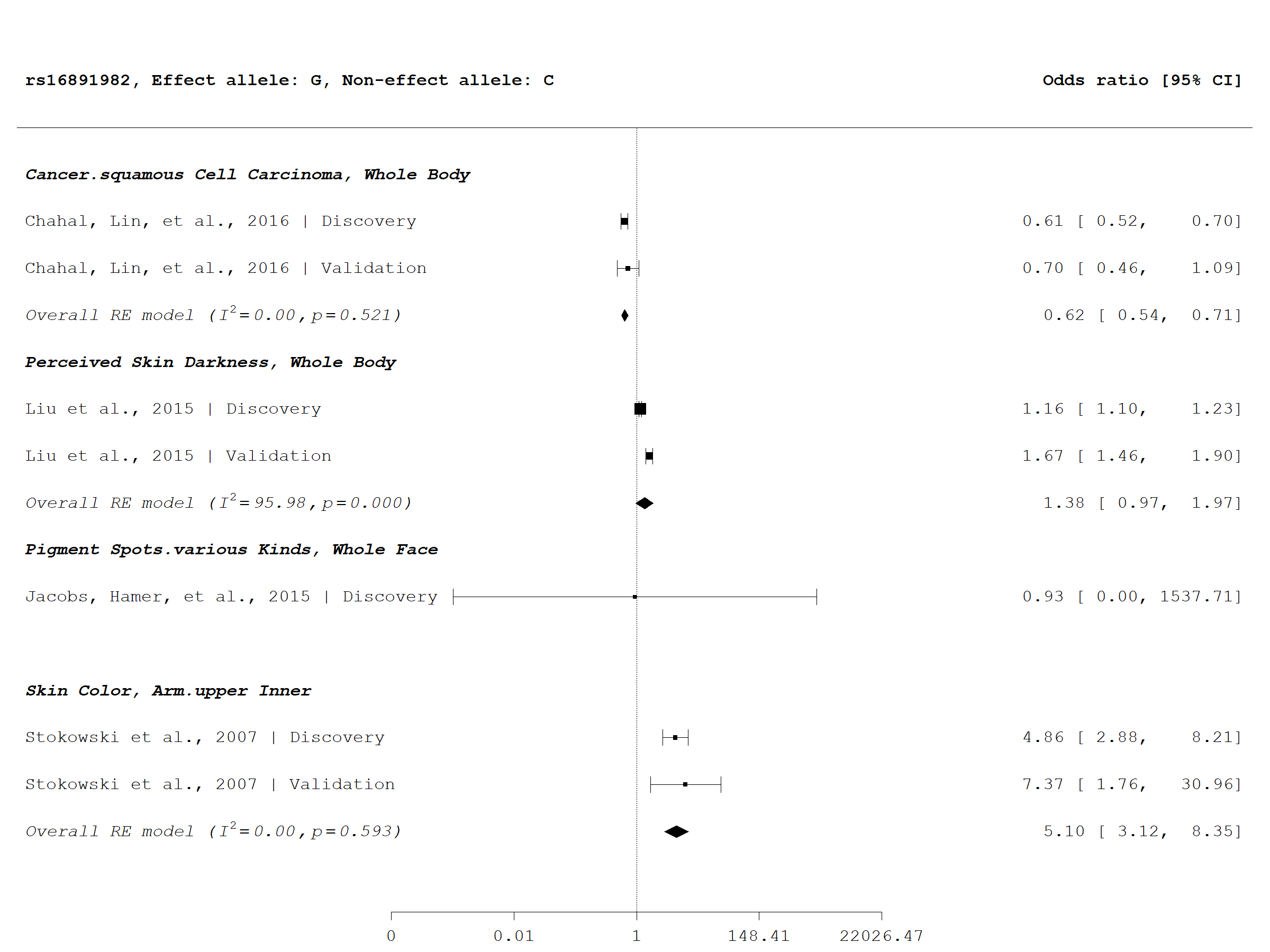

Supplement: Supplementary file 1 — Supplementary Information 1. [file 41598_2022_17443_MOESM1_ESM.zip › Supplementary Datasets/Dataset S3 - Forest Plots/fp107_rs16891982.png]

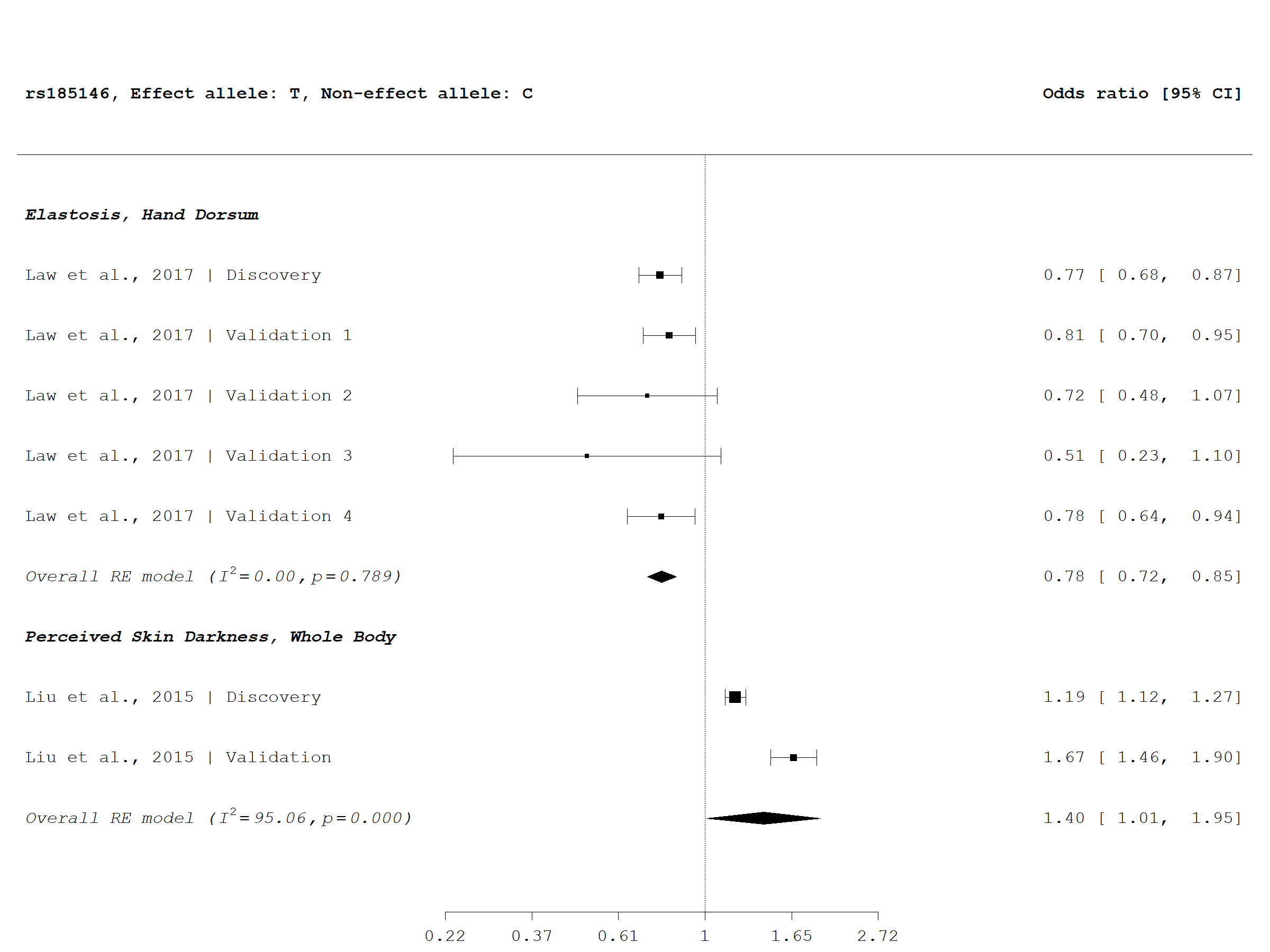

Supplement: Supplementary file 1 — Supplementary Information 1. [file 41598_2022_17443_MOESM1_ESM.zip › Supplementary Datasets/Dataset S3 - Forest Plots/fp108_rs185146.png]

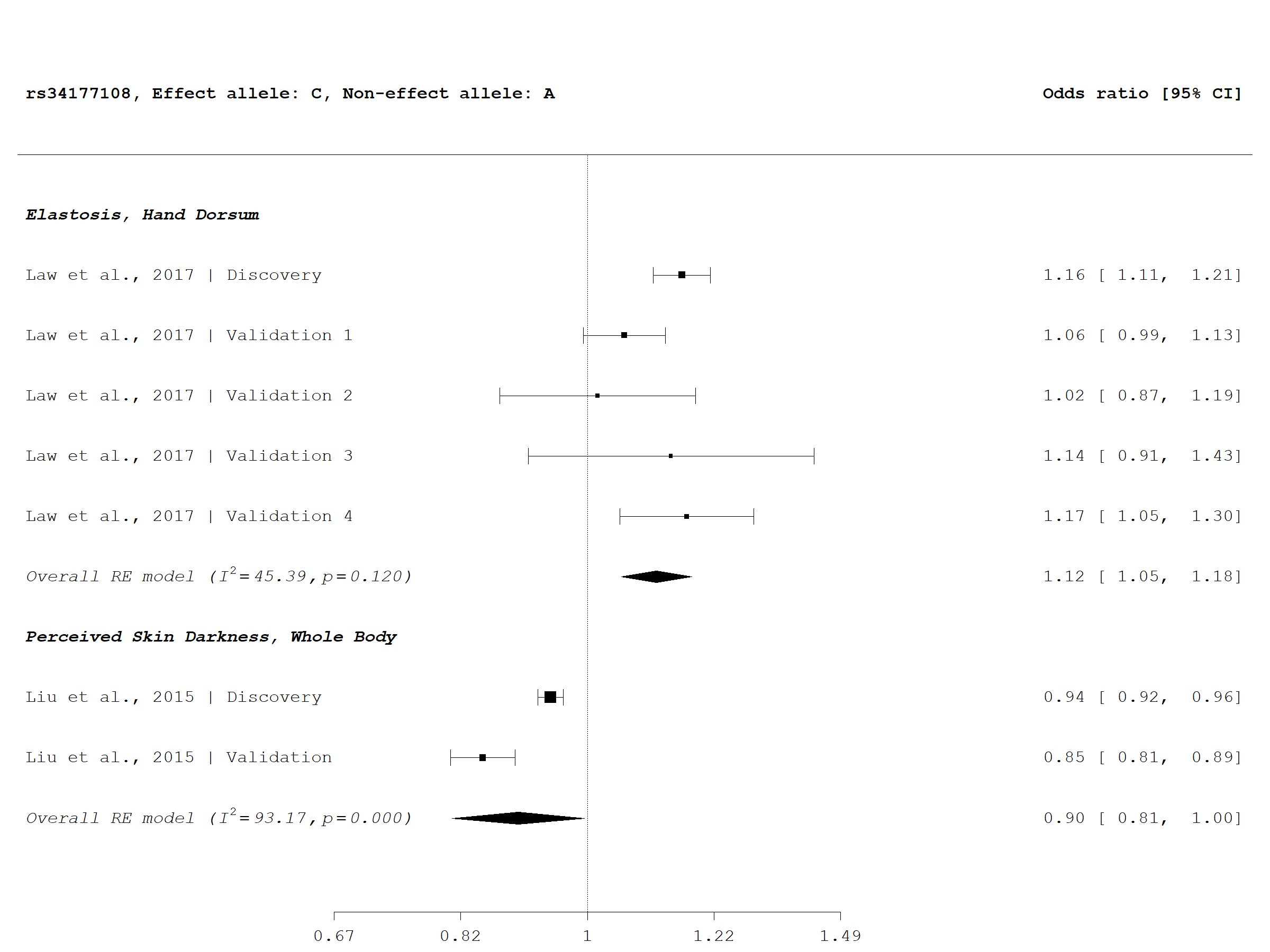

Supplement: Supplementary file 1 — Supplementary Information 1. [file 41598_2022_17443_MOESM1_ESM.zip › Supplementary Datasets/Dataset S3 - Forest Plots/fp109_rs34177108.png]

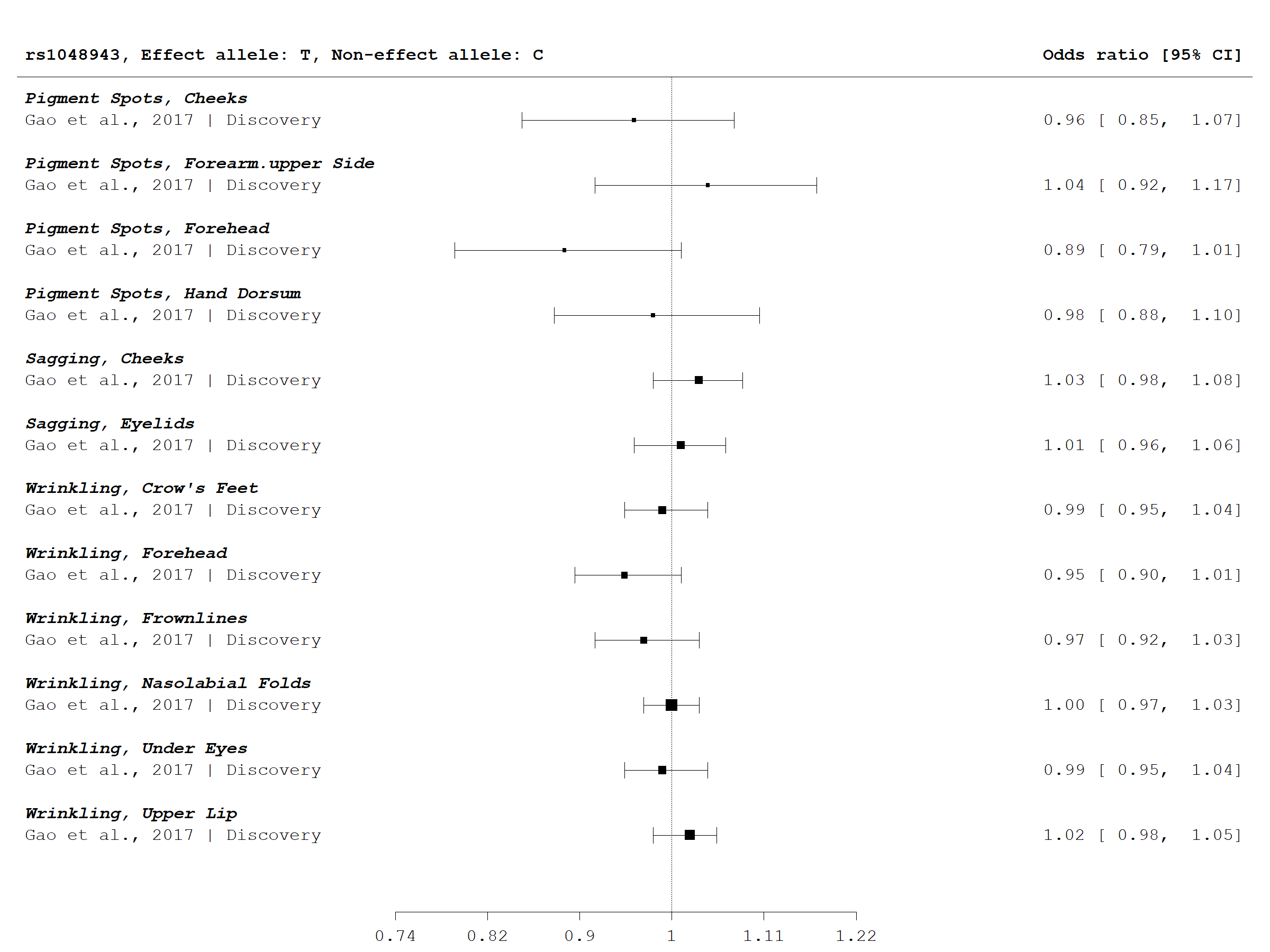

Supplement: Supplementary file 1 — Supplementary Information 1. [file 41598_2022_17443_MOESM1_ESM.zip › Supplementary Datasets/Dataset S3 - Forest Plots/fp10_rs1048943.png]

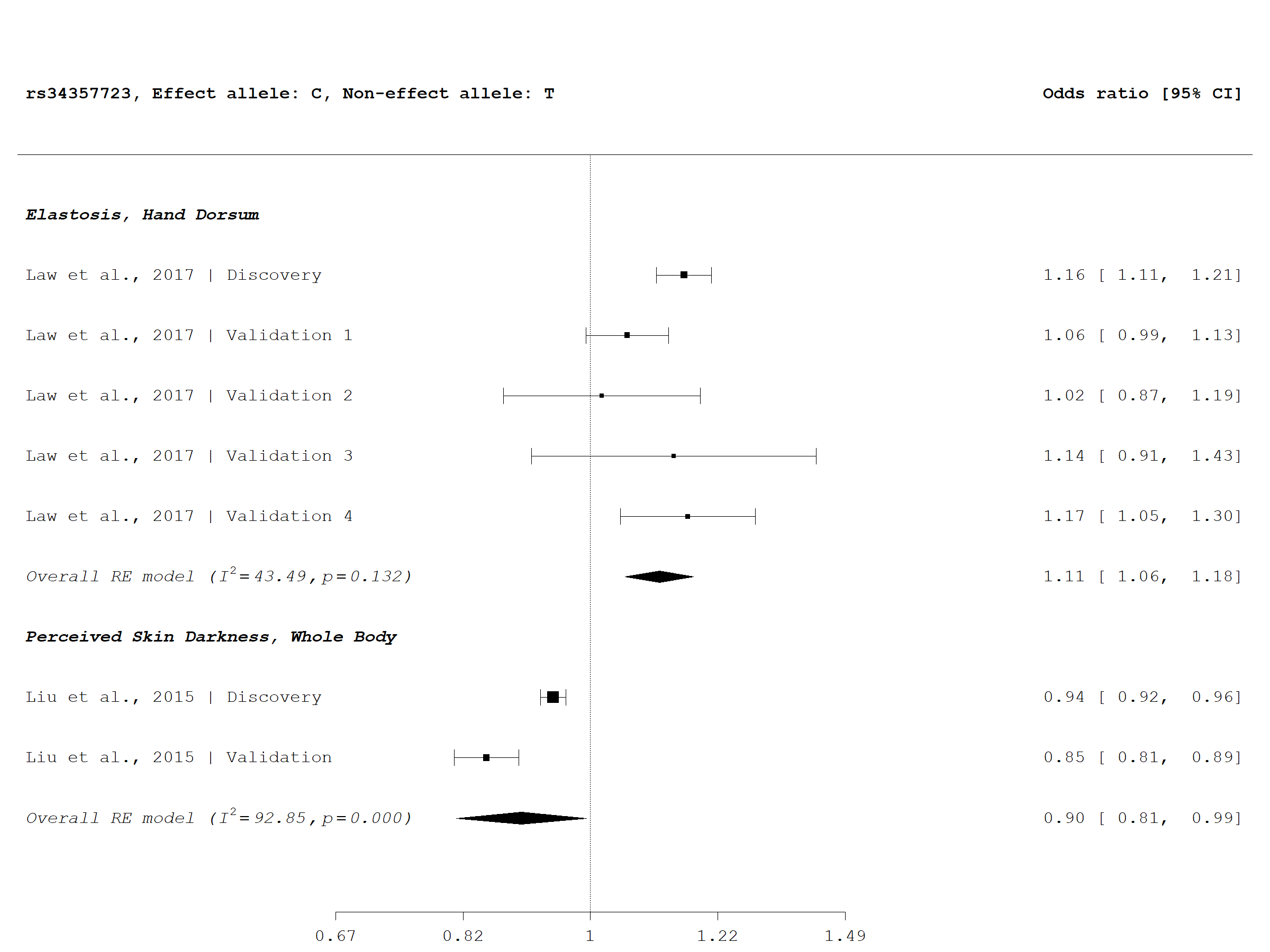

Supplement: Supplementary file 1 — Supplementary Information 1. [file 41598_2022_17443_MOESM1_ESM.zip › Supplementary Datasets/Dataset S3 - Forest Plots/fp110_rs34357723.png]

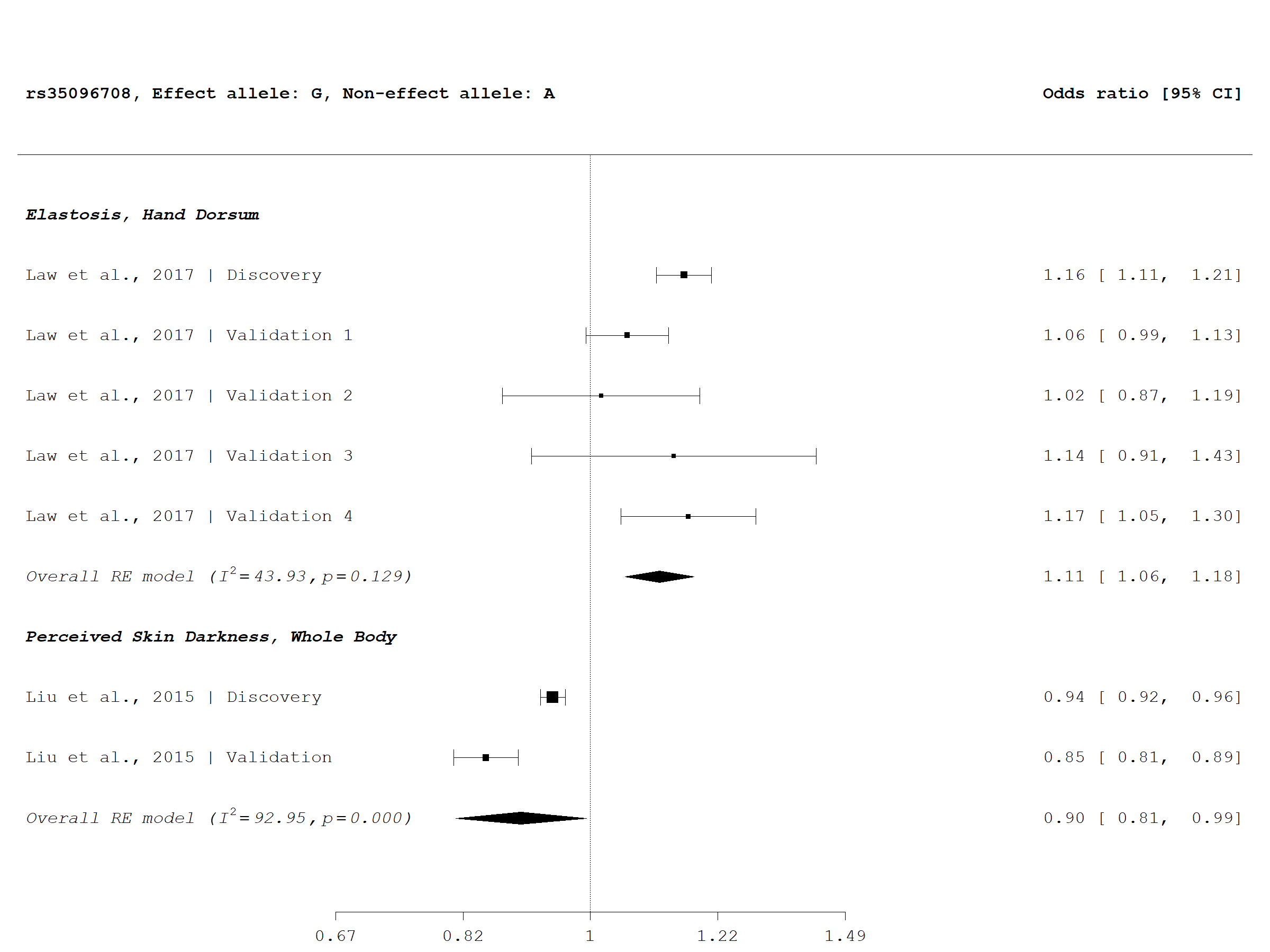

Supplement: Supplementary file 1 — Supplementary Information 1. [file 41598_2022_17443_MOESM1_ESM.zip › Supplementary Datasets/Dataset S3 - Forest Plots/fp111_rs35096708.png]

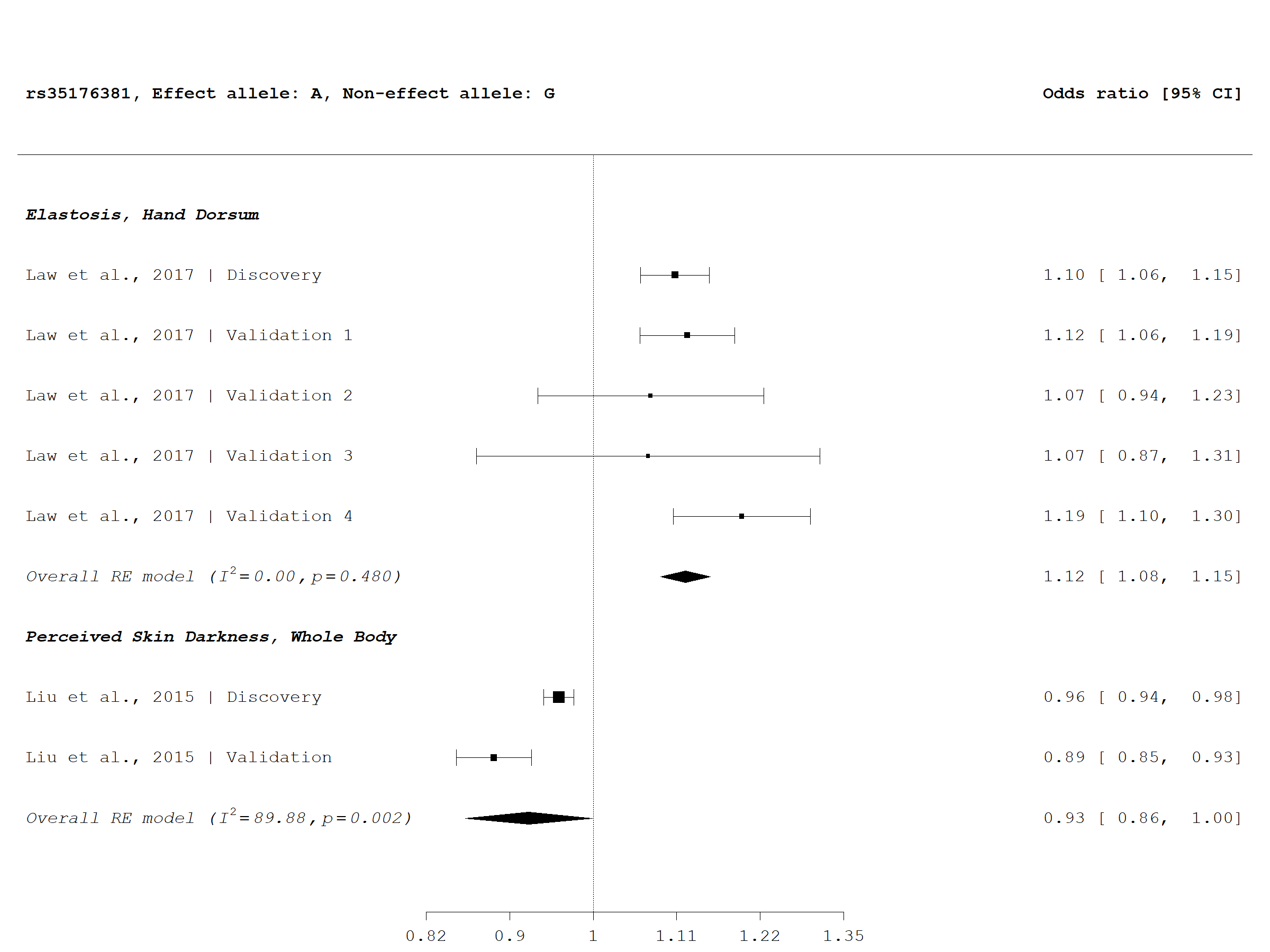

Supplement: Supplementary file 1 — Supplementary Information 1. [file 41598_2022_17443_MOESM1_ESM.zip › Supplementary Datasets/Dataset S3 - Forest Plots/fp112_rs35176381.png]

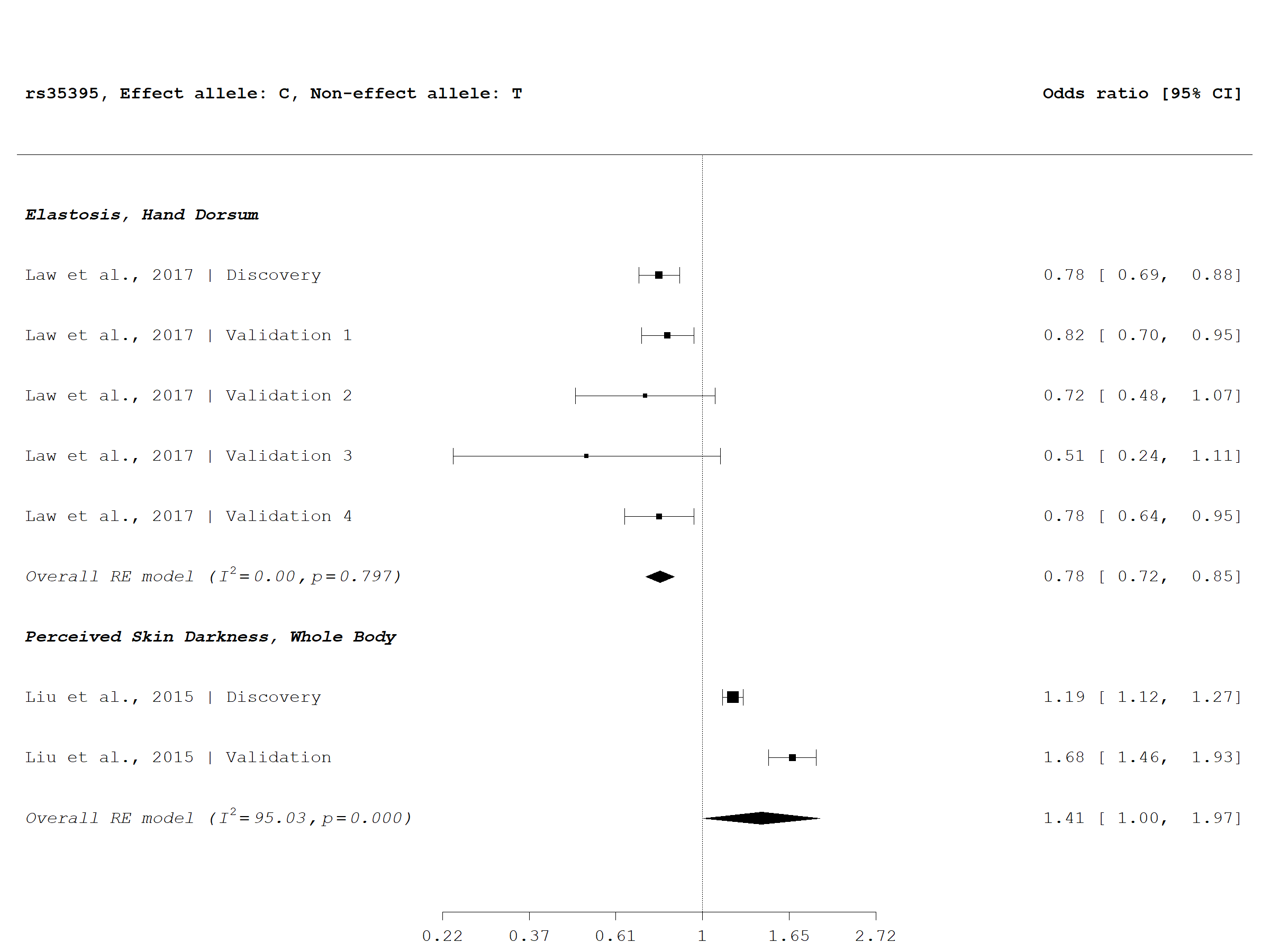

Supplement: Supplementary file 1 — Supplementary Information 1. [file 41598_2022_17443_MOESM1_ESM.zip › Supplementary Datasets/Dataset S3 - Forest Plots/fp113_rs35395.png]

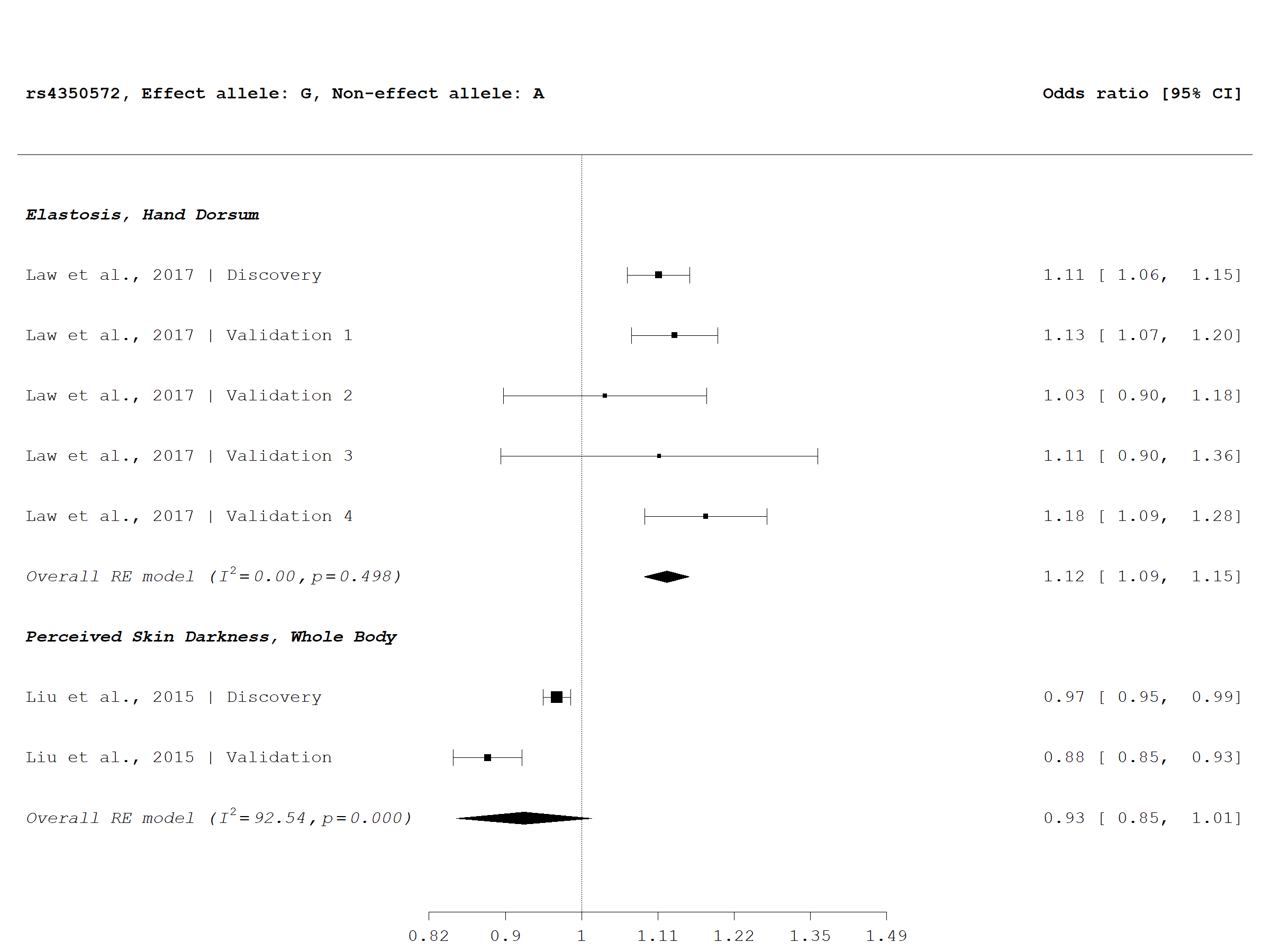

Supplement: Supplementary file 1 — Supplementary Information 1. [file 41598_2022_17443_MOESM1_ESM.zip › Supplementary Datasets/Dataset S3 - Forest Plots/fp114_rs4350572.png]

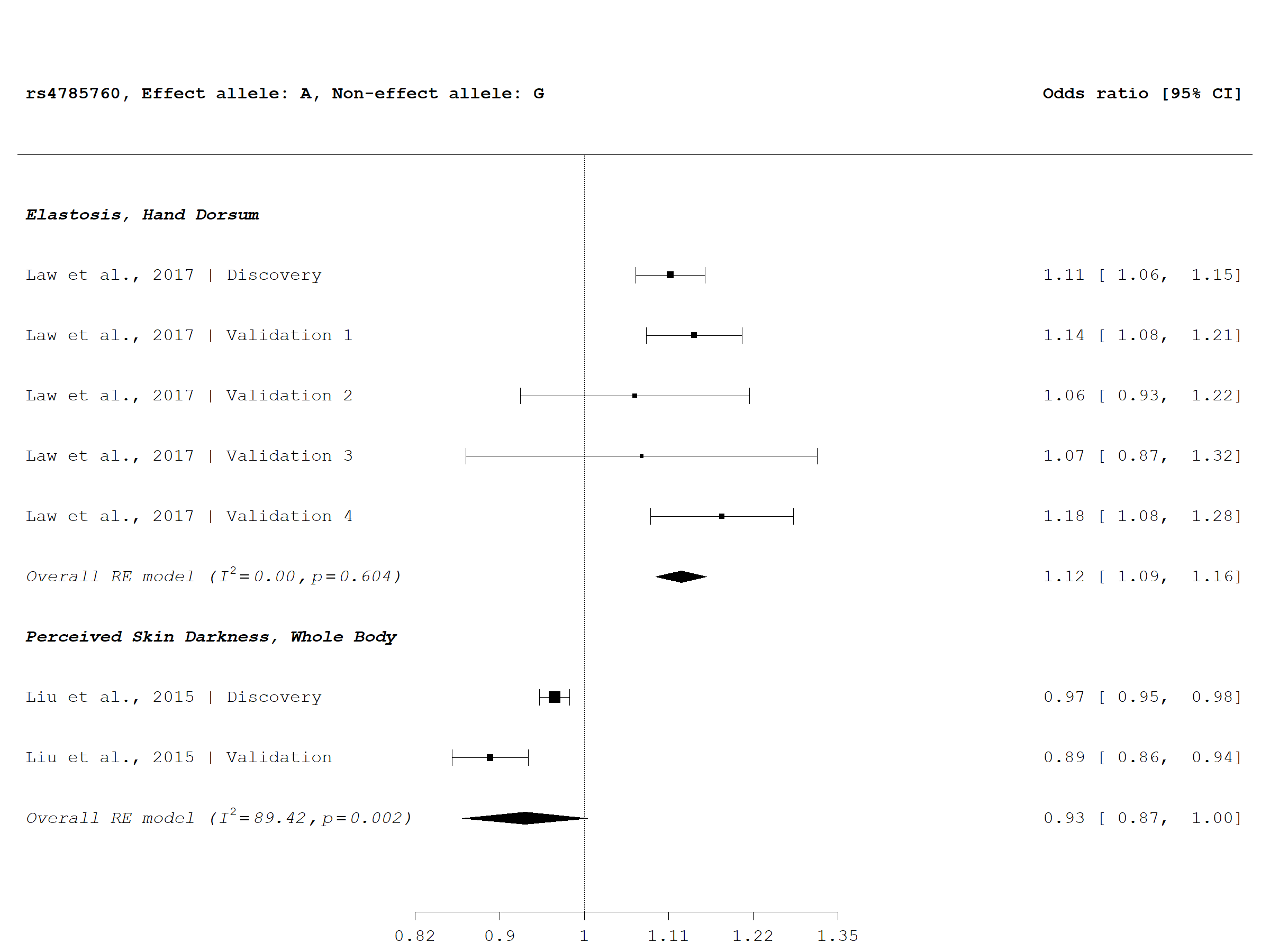

Supplement: Supplementary file 1 — Supplementary Information 1. [file 41598_2022_17443_MOESM1_ESM.zip › Supplementary Datasets/Dataset S3 - Forest Plots/fp115_rs4785760.png]
