# Supplementary material for: A systematic review of skin ageing genes: gene pleiotropy and genes on the chromosomal band 16q24.3 may drive skin ageing
Source: Sci Rep. 2022 Jul 30;12:13099. doi: 10.1038/s41598-022-17443-1 (PMC9338925; doi:10.1038/s41598-022-17443-1)
Supplement: Supplementary file 2 — Supplementary Information 2. [file 41598_2022_17443_MOESM2_ESM.zip › Supplementary Information/Supplementary References.docx]

**Supplementary References**

The 44 eligible publications which were identified during the primary search, secondary search, and the subsequent screening process and used for analysis in this systematic review.

Asgari, M. M. *et al.* Identification of Susceptibility Loci for Cutaneous Squamous Cell Carcinoma. *J. Invest. Dermatol.* **136**, 930–937 (2016).

Bastiaens, M. *et al.* The melanocortin-1-receptor gene is the major freckle gene. *Hum. Mol. Genet.* **10**, 1701–1708 (2001).

Brown, K. M. *et al.* Common sequence variants on 20q11.22 confer melanoma susceptibility. *Nat. Genet.* **40**, 838–840 (2008).

Chang, A. L. S. *et al.* Identification of genes promoting skin youthfulness by genome-wide association study. *J. Invest. Dermatol.* **134**, 651–657 (2014).

Chang, Y. H. *et al.* Use of whole-exome sequencing to determine the genetic basis of signs of skin youthfulness in Korean women. *J. Eur. Acad. Dermatology Venereol.* **31**, e138–e141 (2017).

Chahal, H. S. *et al.* Genome-wide association study identifies 14 novel risk alleles associated with basal cell carcinoma. *Nat. Commun.* **7**, 1–10 (2016).

Chahal, H. S. *et al.* Genome-wide association study identifies novel susceptibility loci for cutaneous squamous cell carcinoma. *Nat. Commun.* **7**, 12048 (2016).

Chen, Y. *et al.* *A GWAS identifies novel gene associations with facial skin wrinkling and mole count in Latin‐Americans*. *British Journal of Dermatology* (2021). doi:10.1111/bjd.20436.

Crawford, N. G. *et al.* Loci associated with skin pigmentation identified in African populations. *Science (80-. ).* **358**, Epub (2018).

Endo, C. *et al.* Genome-wide association study in Japanese females identifies fifteen novel skin-related trait associations. *Sci. Rep.* **8**, (2018).

Eriksson, N. *et al.* Web-Based, Participant-Driven Studies Yield Novel Genetic Associations for Common Traits. *PLoS Genet.* **6**, e1000993 (2010).

Ezzedine, K. *et al.* Freckles and solar lentigines have different risk factors in Caucasian women. *J. Eur. Acad. Dermatology Venereol.* **27**, e345–e356 (2012).

Gao, W. *et al.* Genetic variants associated with skin aging in the Chinese Han population. *J. Dermatol. Sci.* **86**, 21–29 (2017).

Glass, D. *et al.* Gene expression changes with age in skin, adipose tissue, blood and brain. *Genome Biol.* **14**, R75 (2013).

Hamer, M. A. *et al.* Facial Wrinkles in Europeans: A Genome-Wide Association Study. *J. Invest. Dermatol.* **138**, 1877–1880 (2018).

Han, J. *et al.* A Genome-Wide Association Study Identifies Novel Alleles Associated with Hair Color and Skin Pigmentation. *PLoS Genet.* **4**, e1000074 (2008).

Inoue, Y. *et al.* Search for genetic loci involved in the constitution and skin type of a Japanese women using a genome-wide association study. *Exp. Dermatol.* **30**, 1787–1793 (2021).

Ioannidis, N. M. *et al.* Gene expression imputation identifies candidate genes and susceptibility loci associated with cutaneous squamous cell carcinoma. *Nat. Commun.* **9**, (2018).

Jacobs, L. C. *et al.* Intrinsic and extrinsic risk factors for sagging eyelids. *JAMA Dermatology* **150**, 836–843 (2014).

Jacobs, L. C. *et al.* A Genome-Wide Association Study Identifies the Skin Color Genes IRF4 , MC1R , ASIP , and BNC2 Influencing Facial Pigmented Spots. *J. Invest. Dermatol.* **135**, 1735–1742 (2015).

Jacobs, L. C. *et al.* IRF4, MC1R and TYR genes are risk factors for actinic keratosis independent of skin color. *Hum. Mol. Genet.* **24**, 3296–3303 (2015).

Kennedy, C. *et al.* Melanocortin 1 receptor (MC1R) gene variants are associated with an increased risk for cutaneous melanoma which is largely independent of skin type and hair color. *J. Invest. Dermatol.* **117**, 294–300 (2001).

Laville, V. *et al.* A Genome-wide association study in Caucasian women suggests the involvement of HLA genes in the severity of facial solar lentigines. *Pigment Cell Melanoma Res.* **29**, 550–558 (2016).

Laville, V. *et al.* A genome wide association study identifies new genes potentially associated with eyelid sagging. *Exp. Dermatol.* **28**, 892–898 (2018).

Law, M. H. *et al.* Genome-Wide Association Shows that Pigmentation Genes Play a Role in Skin Aging. *J. Invest. Dermatol.* **137**, 1887–1894 (2017).

Le Clerc, S. *et al.* A genome-wide association study in caucasian women points out a putative role of the STXBP5L gene in facial photoaging. *J. Invest. Dermatol.* **133**, 929–935 (2013).

Liu, F. *et al.* Genetics of skin color variation in Europeans: genome-wide association studies with functional follow-up. *Hum. Genet.* **134**, 823–835 (2015).

Liu, F. *et al.* The MC1R Gene and Youthful Looks. *Curr. Biol.* **26**, 1213–1220 (2016).

Liu, Y. *et al.* Genome-wide scan identified genetic variants associated with skin aging in a Chinese female population. *J. Dermatol. Sci.* **96**, 42–49 (2019).

Mekić, S. *et al.* Genetics of facial telangiectasia in the Rotterdam Study: a genome-wide association study and candidate gene approach. *J. Eur. Acad. Dermatology Venereol.* **35**, 749–754 (2021).

Motokawa, T., Kato, T., Hashimoto, Y. & Katagiri, T. Effect of Val92Met and Arg163Gln variants of the MC1R gene on freckles and solar lentigines in Japanese. *Pigment Cell Res.* **20**, 140–143 (2007).

Motokawa, T. *et al.* Polymorphism patterns in the promoter region of the MC1R gene are associated with development of freckles and solar lentigines. *J. Invest. Dermatol.* **128**, 1588–1591 (2008).

Nan, H. *et al.* Genome-Wide Association Study of Tanning Phenotype in a Population of European Ancestry. *J. Invest. Dermatol.* **129**, 2250–2257 (2009).

Shido, K. *et al.* Susceptibility Loci for Tanning Ability in the Japanese Population Identified by a Genome-Wide Association Study from the Tohoku Medical Megabank Project Cohort Study. *J. Invest. Dermatol.* **139**, 1605-1608.e13 (2019).

Shin, J. G. *et al.* GWAS Analysis of 17,019 Korean Women Identifies the Variants Associated with Facial Pigmented Spots. *J. Invest. Dermatol.* **141**, 555–562 (2021).

Stacey, S. N. *et al.* Common variants on 1p36 and 1q42 are associated with cutaneous basal cell carcinoma but not with melanoma or pigmentation traits. *Nat. Genet.* **40**, 1313–1318 (2008).

Stacey, S. N. *et al.* New common variants affecting susceptibility to basal cell carcinoma. *Nat. Genet.* **41**, 909–914 (2009).

Stokowski, R. P. *et al.* A genomewide association study of skin pigmentation in a South Asian population. *Am. J. Hum. Genet.* **81**, 1119–1132 (2007).

Sulem, P. *et al.* Genetic determinants of hair, eye and skin pigmentation in Europeans. *Nat. Genet.* **39**, 1443–1452 (2007).

Suppa, M. *et al.* The determinants of periorbital skin ageing in participants of a melanoma case-control study in the U.K. *Br. J. Dermatol.* **165**, 1011–1021 (2011).

Vierkötter, A. *et al.* Development of Lentigines in German and Japanese Women Correlates with Variants in the SLC45A2 Gene. *J. Invest. Dermatol.* **132**, 733–736 (2012).

Yamaguchi, K. *et al.* Association of melanocortin 1 receptor gene (MC1R) polymorphisms with skin reflectance and freckles in Japanese. *J. Hum. Genet.* **57**, 700–708 (2012).

Zhong, K. *et al.* Pigmentation-Independent Susceptibility Loci for Actinic Keratosis Highlighted by Compound Heterozygosity Analysis. *J. Invest. Dermatol.* **137**, 77–84 (2017).

Zhang, M. *et al.* A Genome-Wide Association Study of Basal Transepidermal Water Loss Finds that Variants at 9q34.3 Are Associated with Skin Barrier Function. *J. Invest. Dermatol.* **137**, 979–982 (2017).
