# Supplementary figures and images for: A systematic review of skin ageing genes: gene pleiotropy and genes on the chromosomal band 16q24.3 may drive skin ageing (part 3 of 3)
Source: Sci Rep. 2022 Jul 30;12:13099. doi: 10.1038/s41598-022-17443-1 (PMC9338925; doi:10.1038/s41598-022-17443-1)

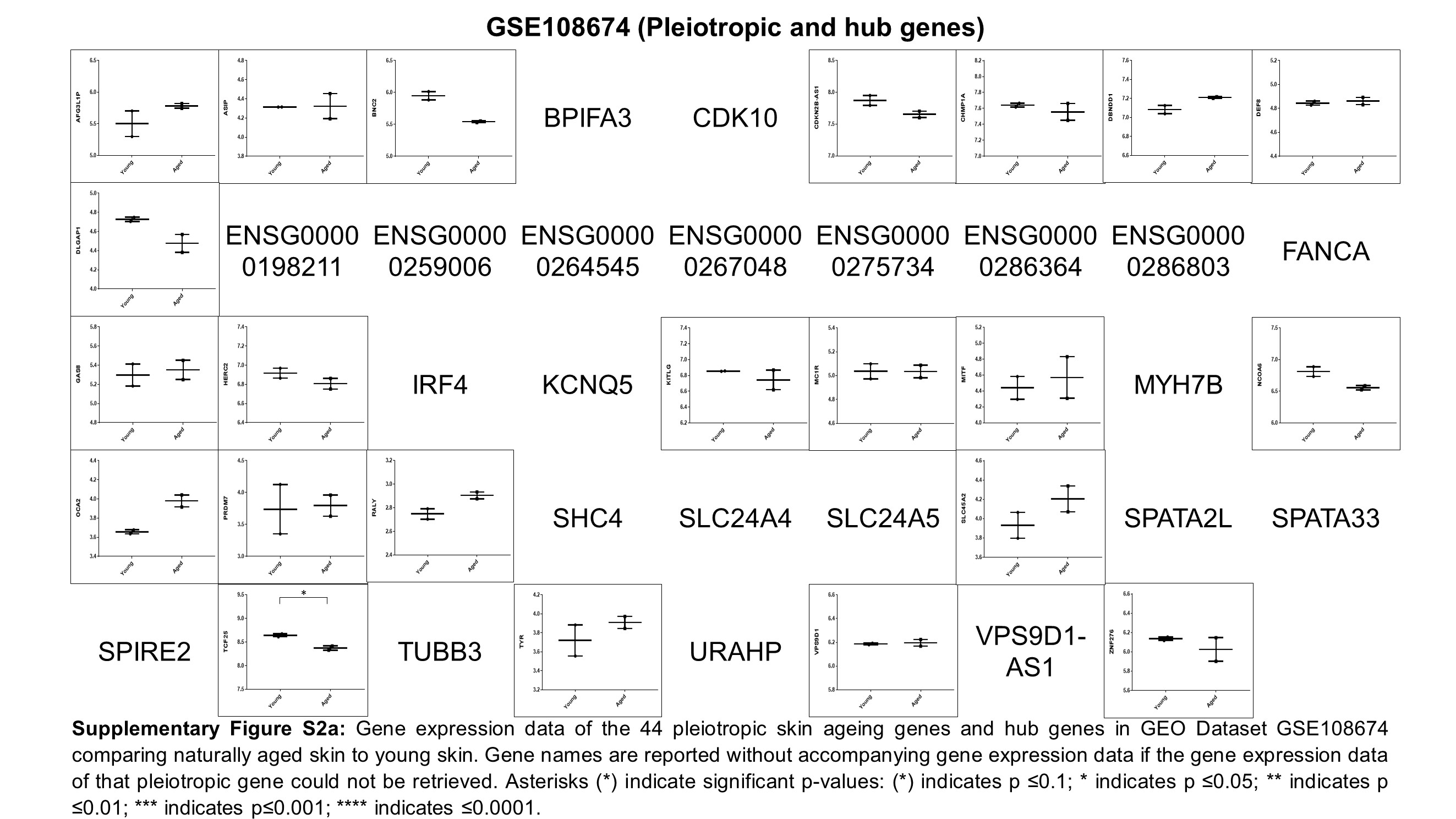

Supplement: Supplementary file 2 — Supplementary Information 2. [file 41598_2022_17443_MOESM2_ESM.zip › Supplementary Information/Figure S2 - GEO Dataset GSE108674/Supplementary Figure S2a.JPG]

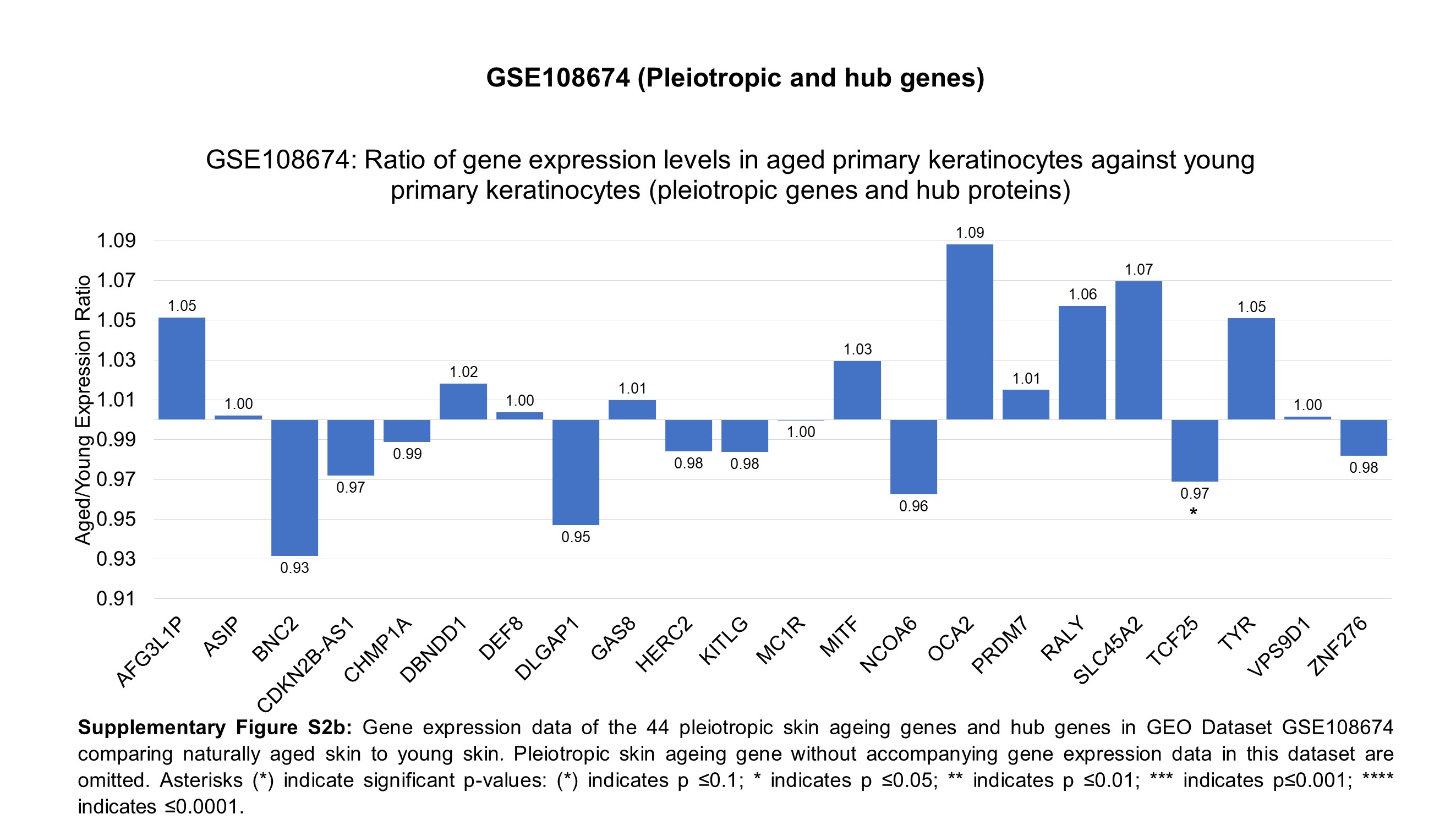

Supplement: Supplementary file 2 — Supplementary Information 2. [file 41598_2022_17443_MOESM2_ESM.zip › Supplementary Information/Figure S2 - GEO Dataset GSE108674/Supplementary Figure S2b.JPG]

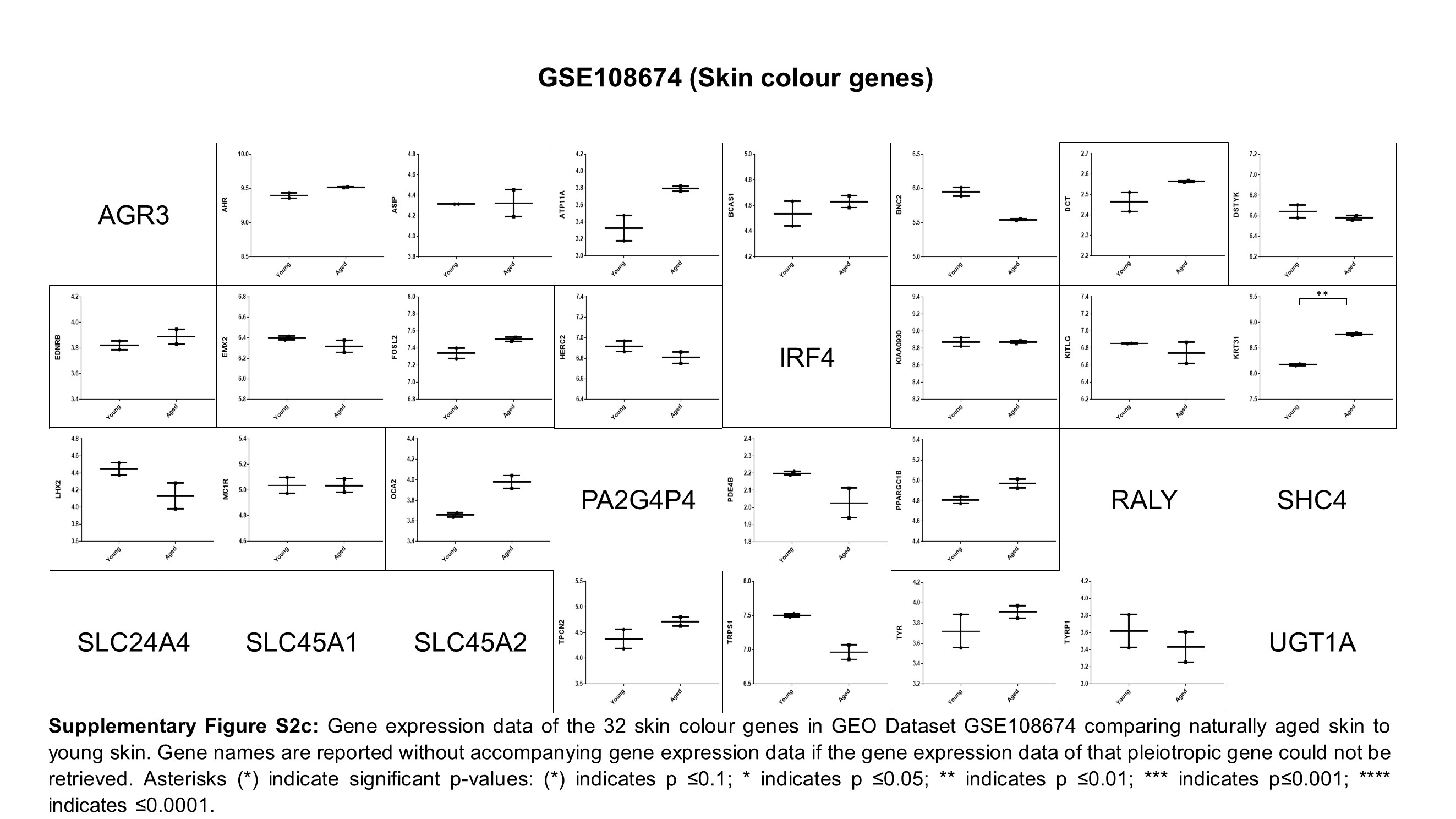

Supplement: Supplementary file 2 — Supplementary Information 2. [file 41598_2022_17443_MOESM2_ESM.zip › Supplementary Information/Figure S2 - GEO Dataset GSE108674/Supplementary Figure S2c.JPG]

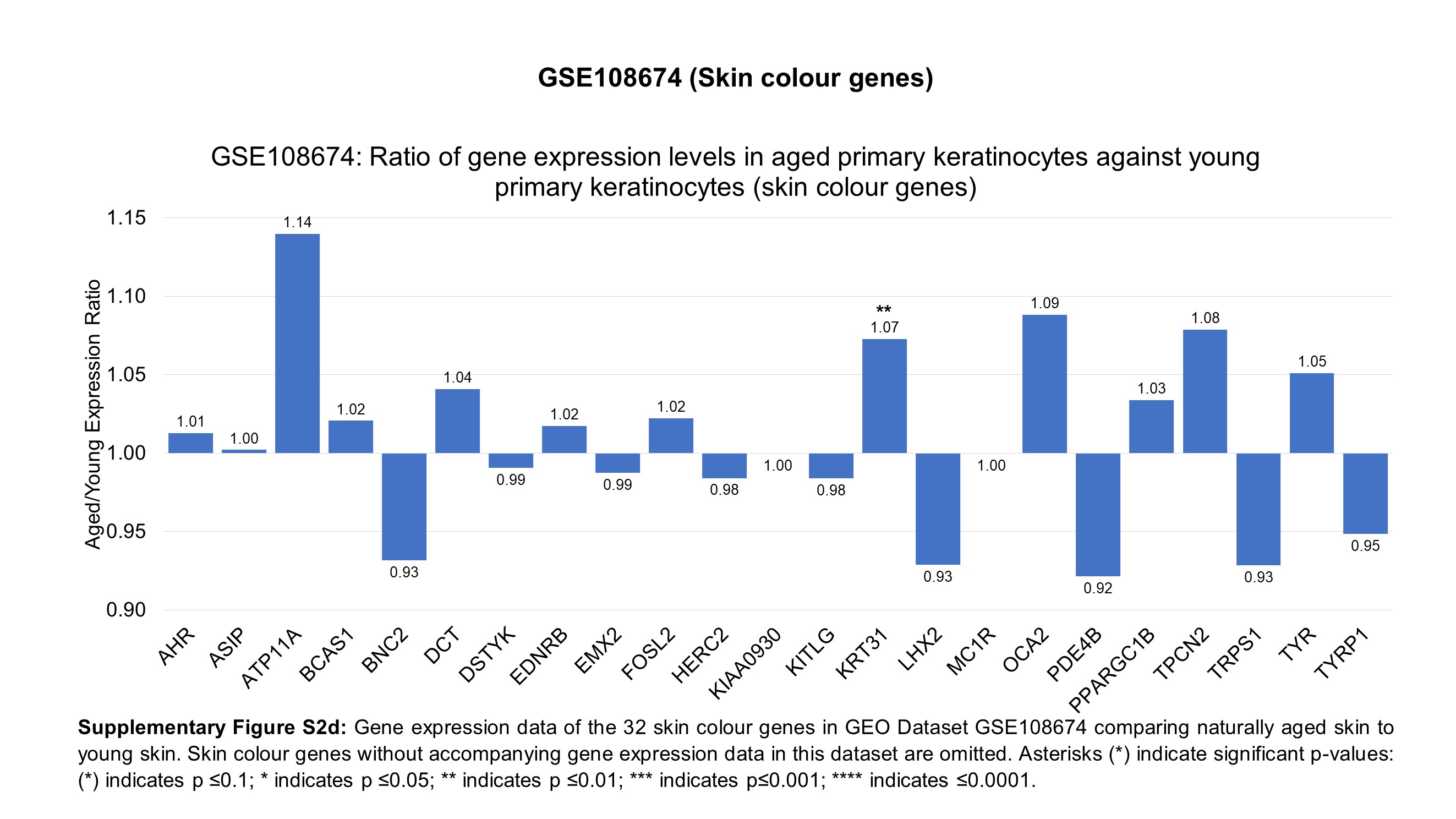

Supplement: Supplementary file 2 — Supplementary Information 2. [file 41598_2022_17443_MOESM2_ESM.zip › Supplementary Information/Figure S2 - GEO Dataset GSE108674/Supplementary Figure S2d.JPG]

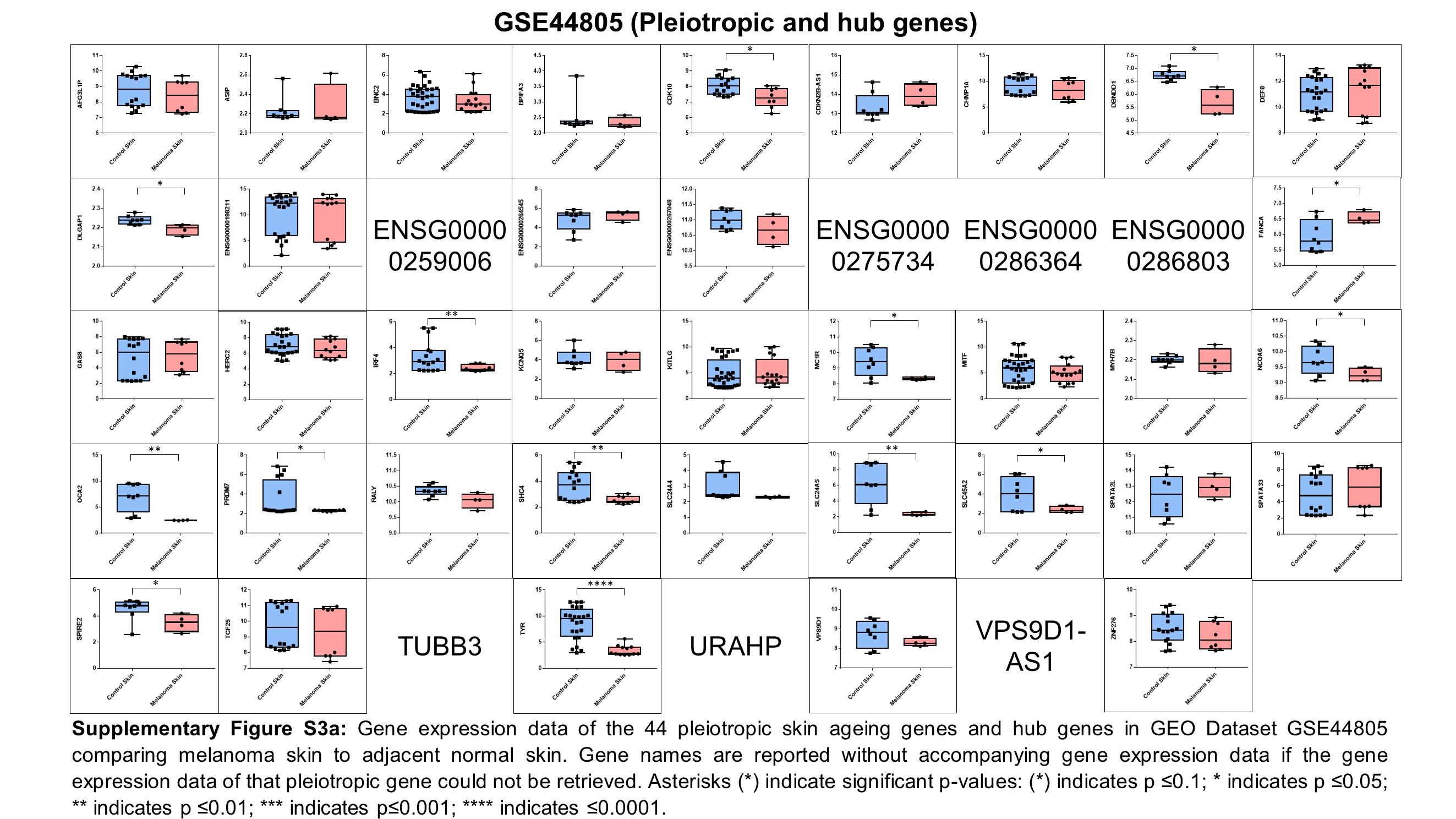

Supplement: Supplementary file 2 — Supplementary Information 2. [file 41598_2022_17443_MOESM2_ESM.zip › Supplementary Information/Figure S3- GEO Dataset GSE44805/Supplementary Figure S3a.JPG]

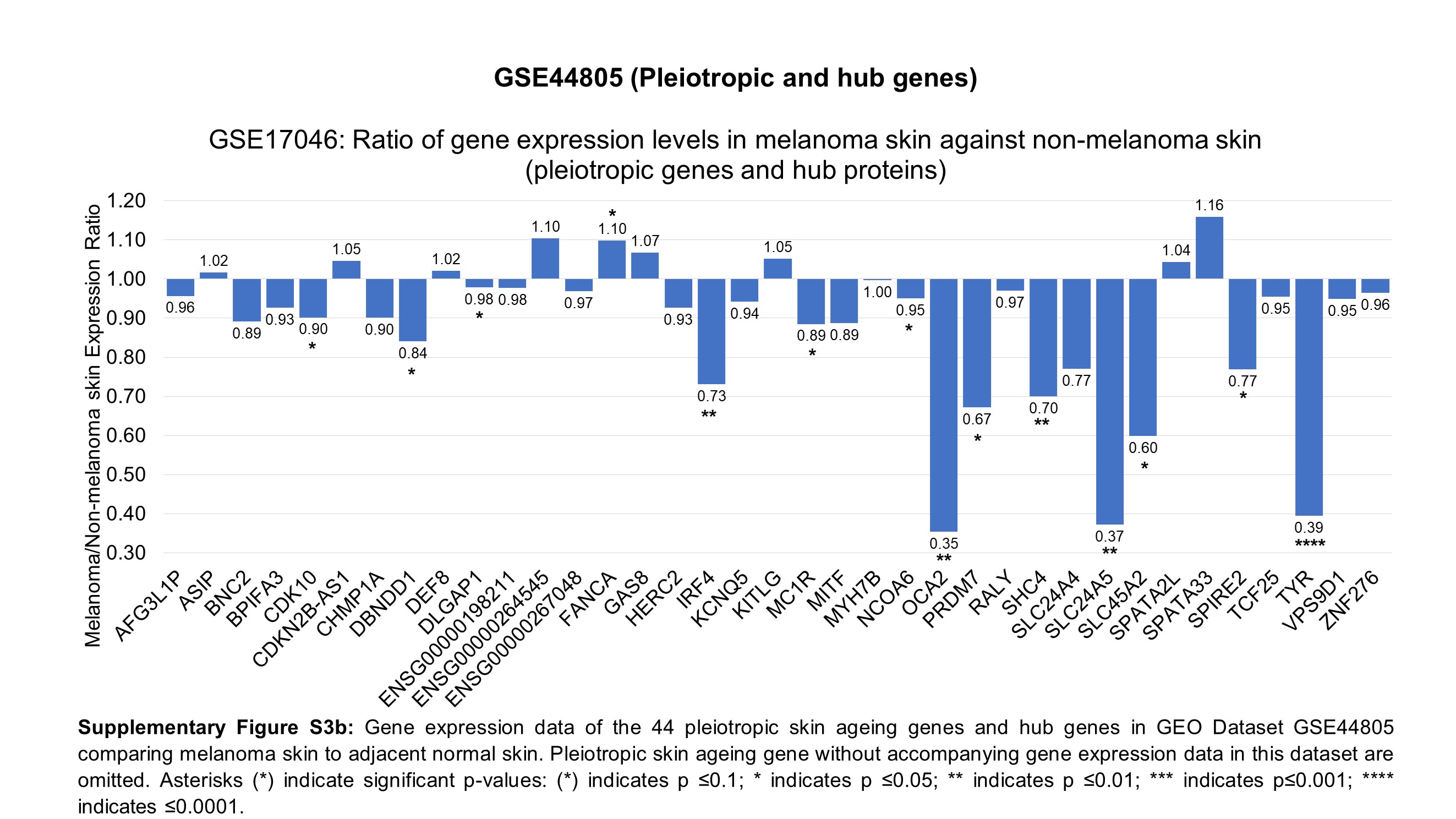

Supplement: Supplementary file 2 — Supplementary Information 2. [file 41598_2022_17443_MOESM2_ESM.zip › Supplementary Information/Figure S3- GEO Dataset GSE44805/Supplementary Figure S3b.JPG]

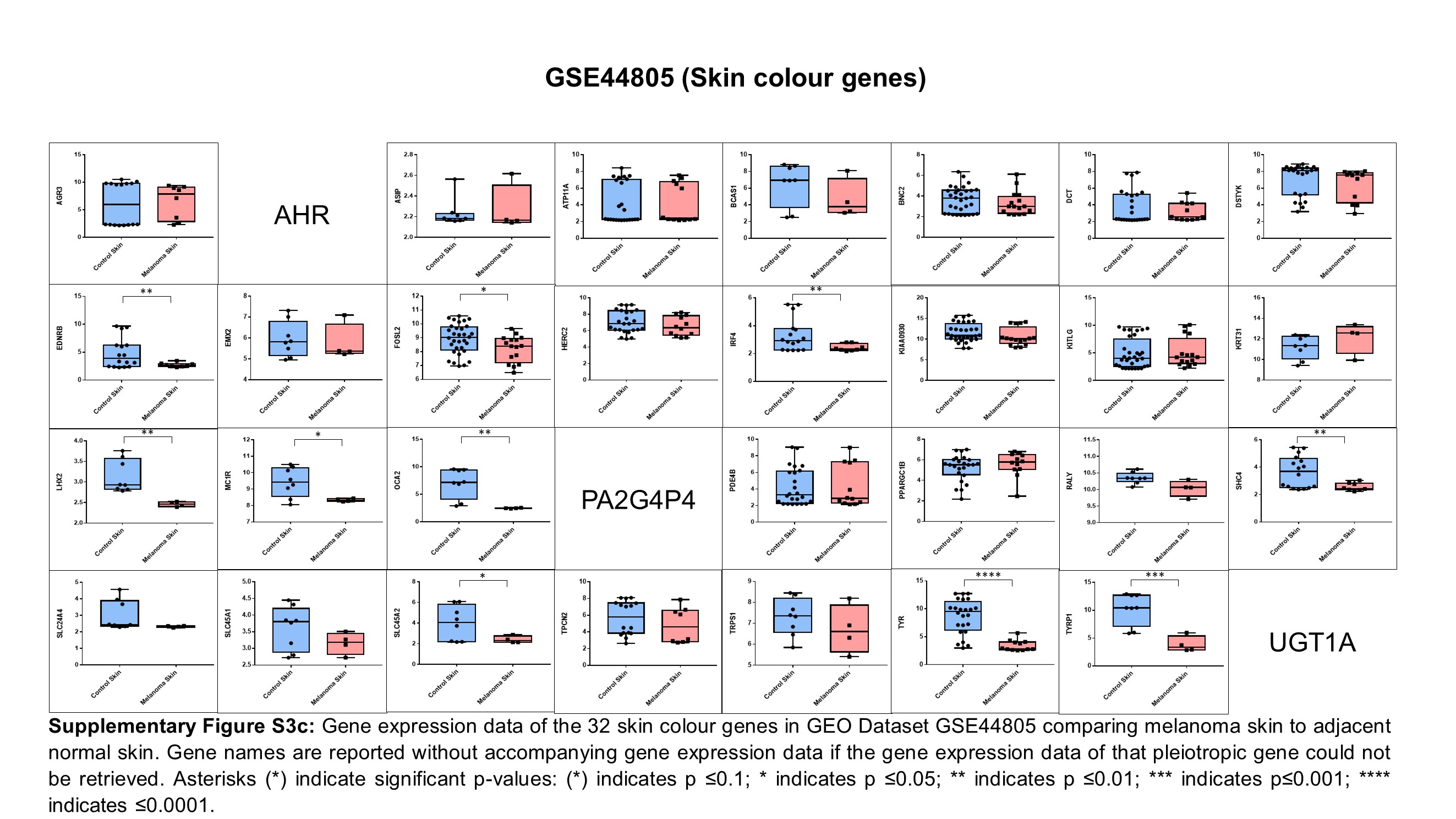

Supplement: Supplementary file 2 — Supplementary Information 2. [file 41598_2022_17443_MOESM2_ESM.zip › Supplementary Information/Figure S3- GEO Dataset GSE44805/Supplementary Figure S3c.JPG]

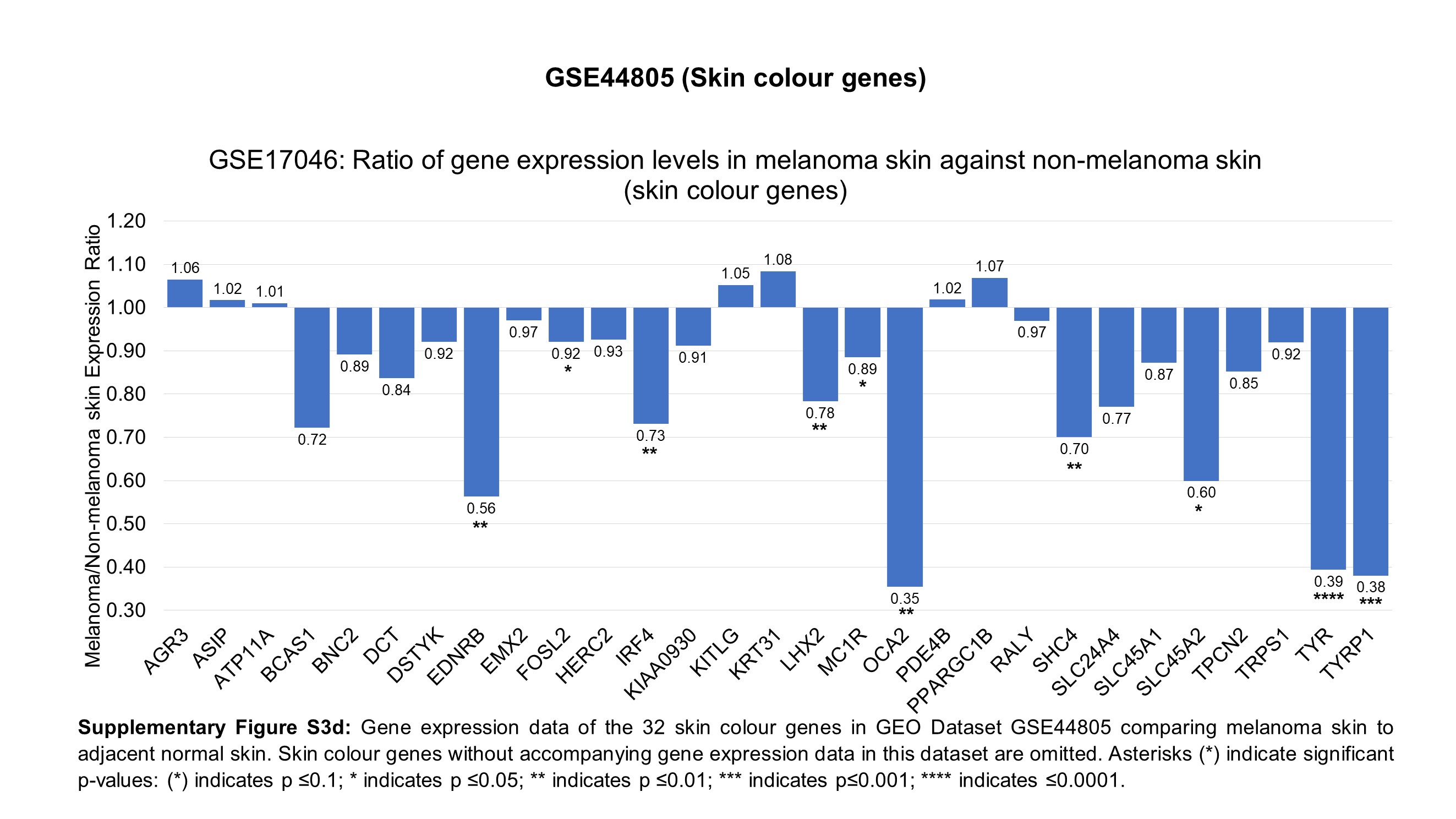

Supplement: Supplementary file 2 — Supplementary Information 2. [file 41598_2022_17443_MOESM2_ESM.zip › Supplementary Information/Figure S3- GEO Dataset GSE44805/Supplementary Figure S3d.JPG]

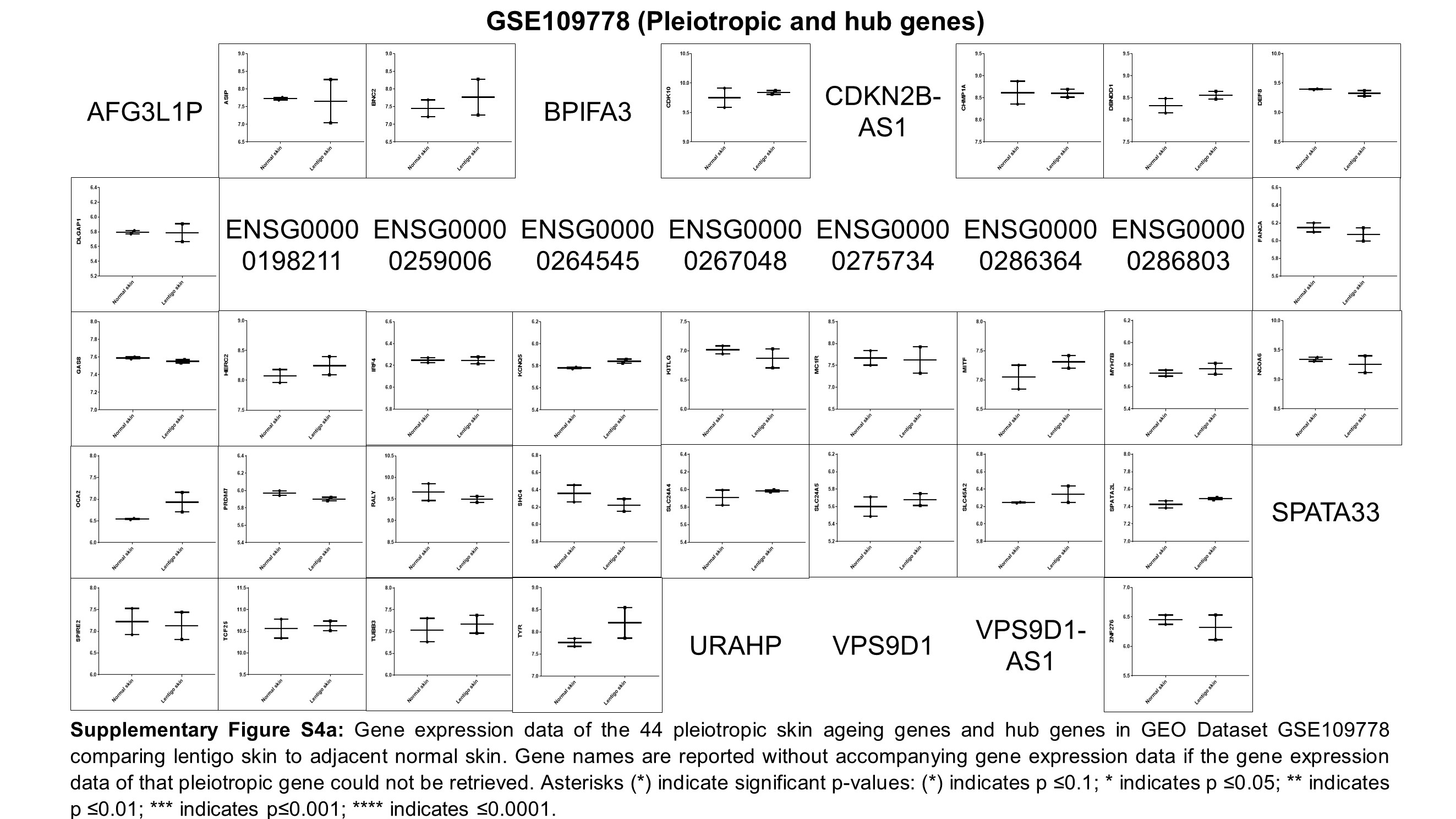

Supplement: Supplementary file 2 — Supplementary Information 2. [file 41598_2022_17443_MOESM2_ESM.zip › Supplementary Information/Figure S4 - GEO Dataset GSE109778/Supplementary Figure S4a.JPG]

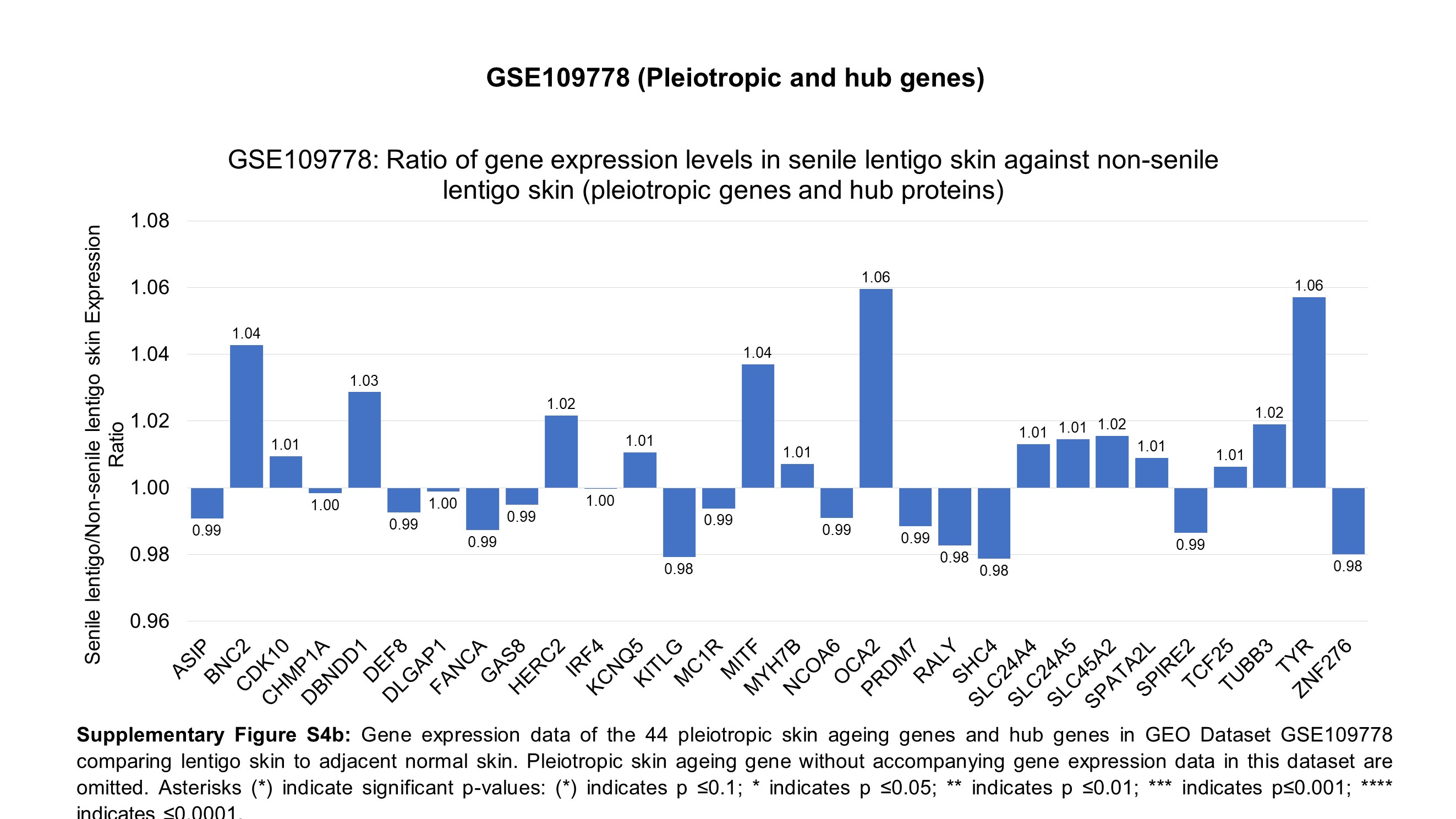

Supplement: Supplementary file 2 — Supplementary Information 2. [file 41598_2022_17443_MOESM2_ESM.zip › Supplementary Information/Figure S4 - GEO Dataset GSE109778/Supplementary Figure S4b.JPG]

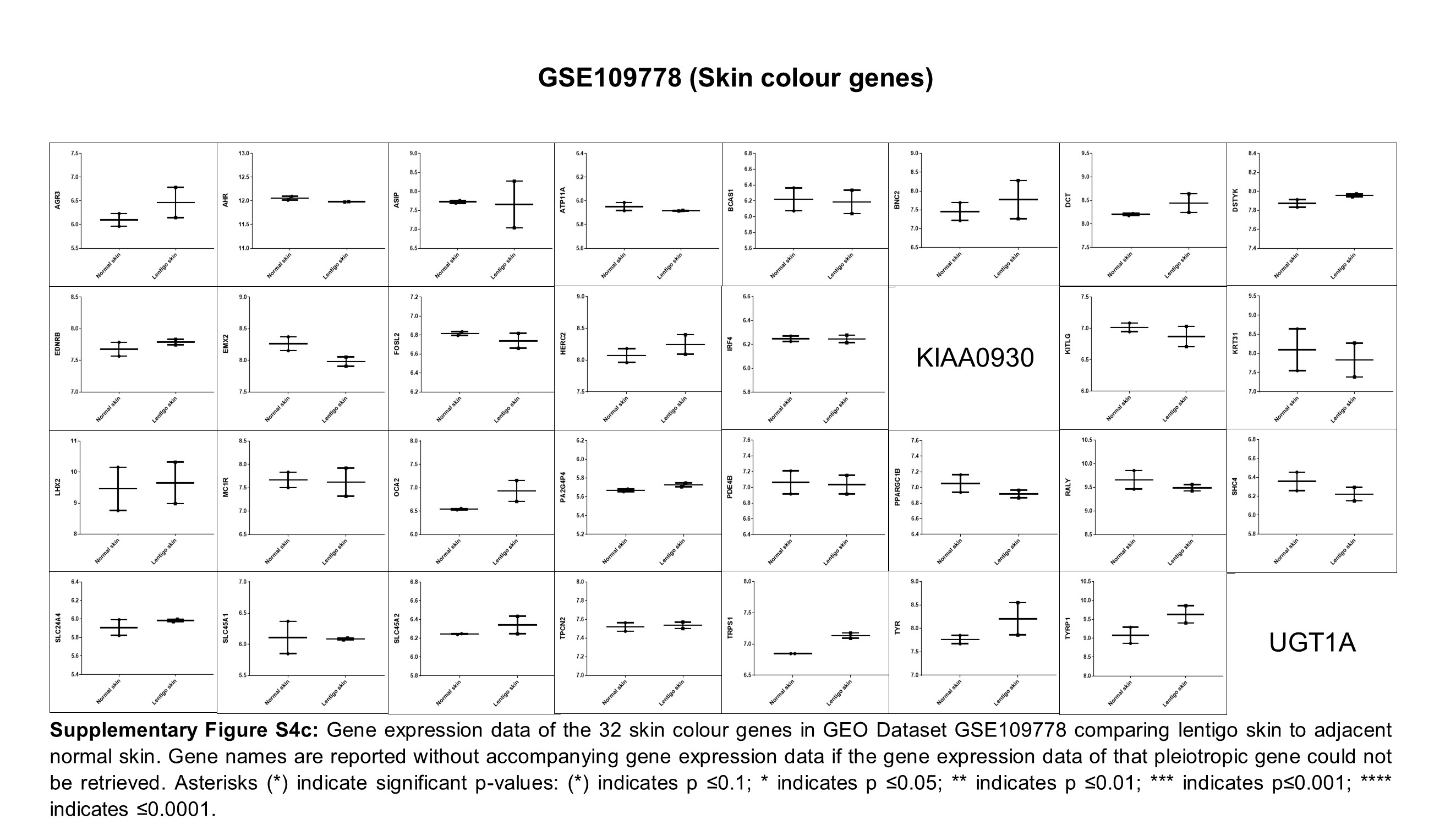

Supplement: Supplementary file 2 — Supplementary Information 2. [file 41598_2022_17443_MOESM2_ESM.zip › Supplementary Information/Figure S4 - GEO Dataset GSE109778/Supplementary Figure S4c.JPG]

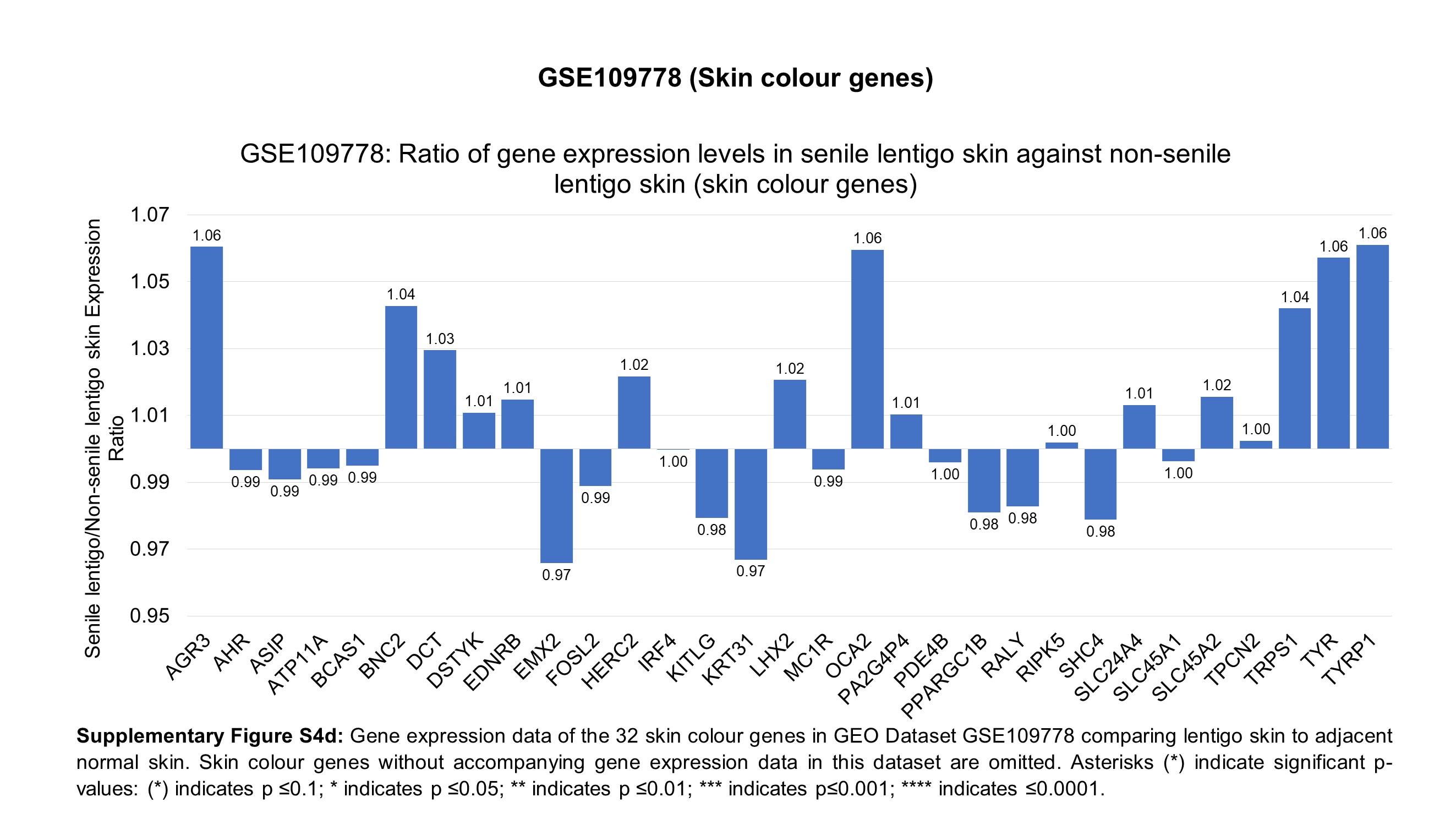

Supplement: Supplementary file 2 — Supplementary Information 2. [file 41598_2022_17443_MOESM2_ESM.zip › Supplementary Information/Figure S4 - GEO Dataset GSE109778/Supplementary Figure S4d.JPG]

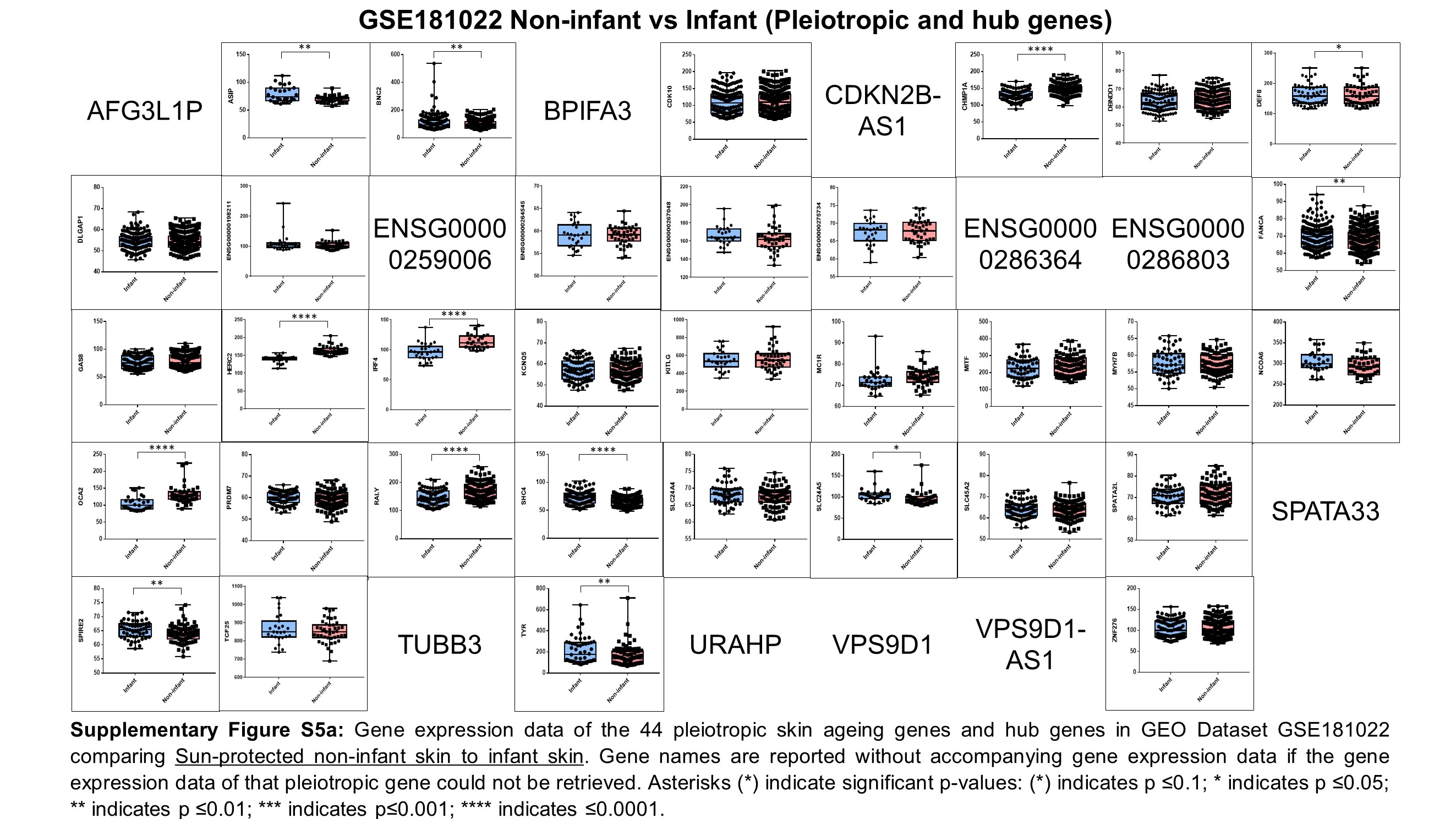

Supplement: Supplementary file 2 — Supplementary Information 2. [file 41598_2022_17443_MOESM2_ESM.zip › Supplementary Information/Figure S5 - GEO Dataset GSE181022/Supplementary Figure S5a.JPG]

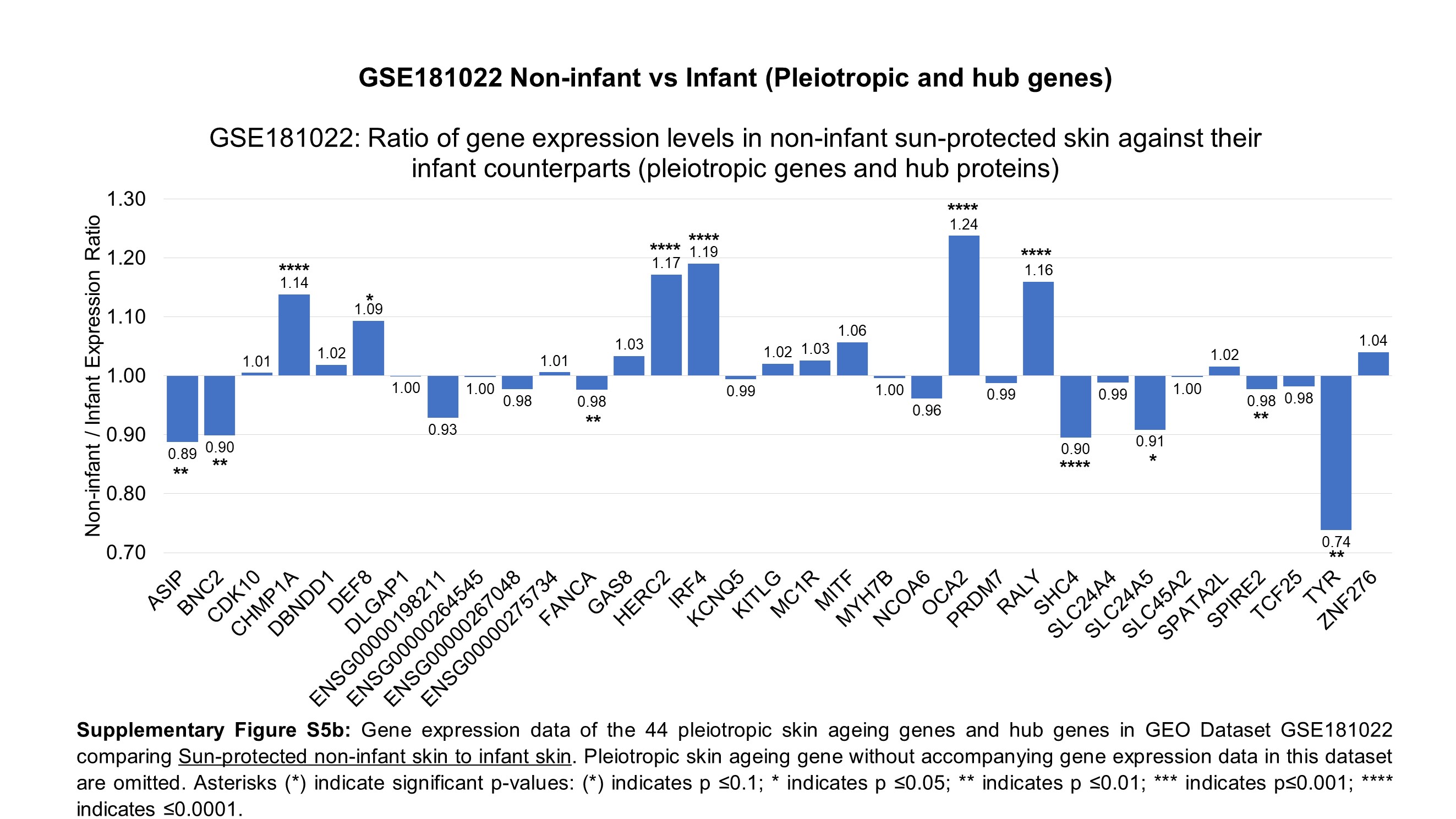

Supplement: Supplementary file 2 — Supplementary Information 2. [file 41598_2022_17443_MOESM2_ESM.zip › Supplementary Information/Figure S5 - GEO Dataset GSE181022/Supplementary Figure S5b.JPG]

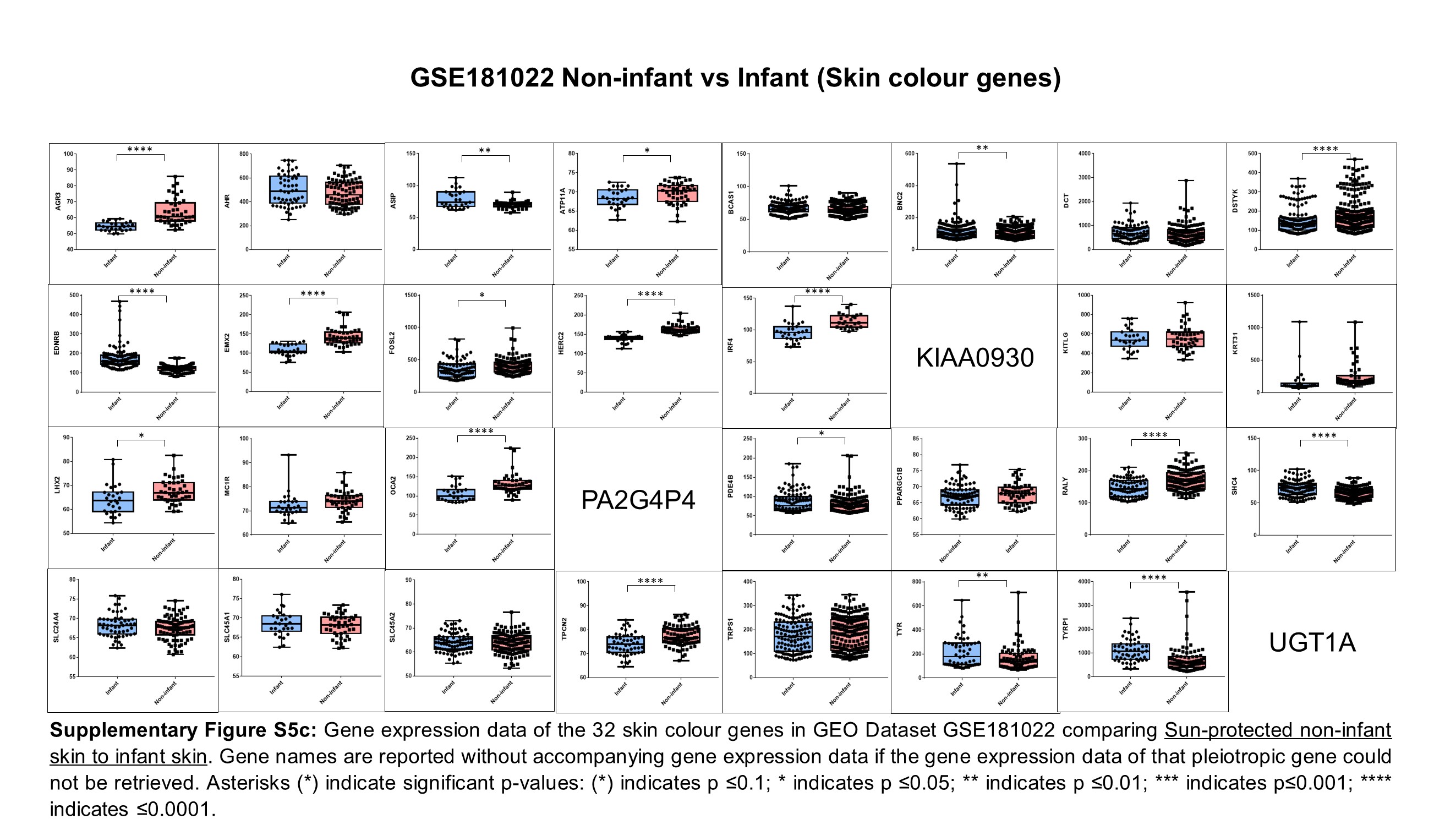

Supplement: Supplementary file 2 — Supplementary Information 2. [file 41598_2022_17443_MOESM2_ESM.zip › Supplementary Information/Figure S5 - GEO Dataset GSE181022/Supplementary Figure S5c.JPG]

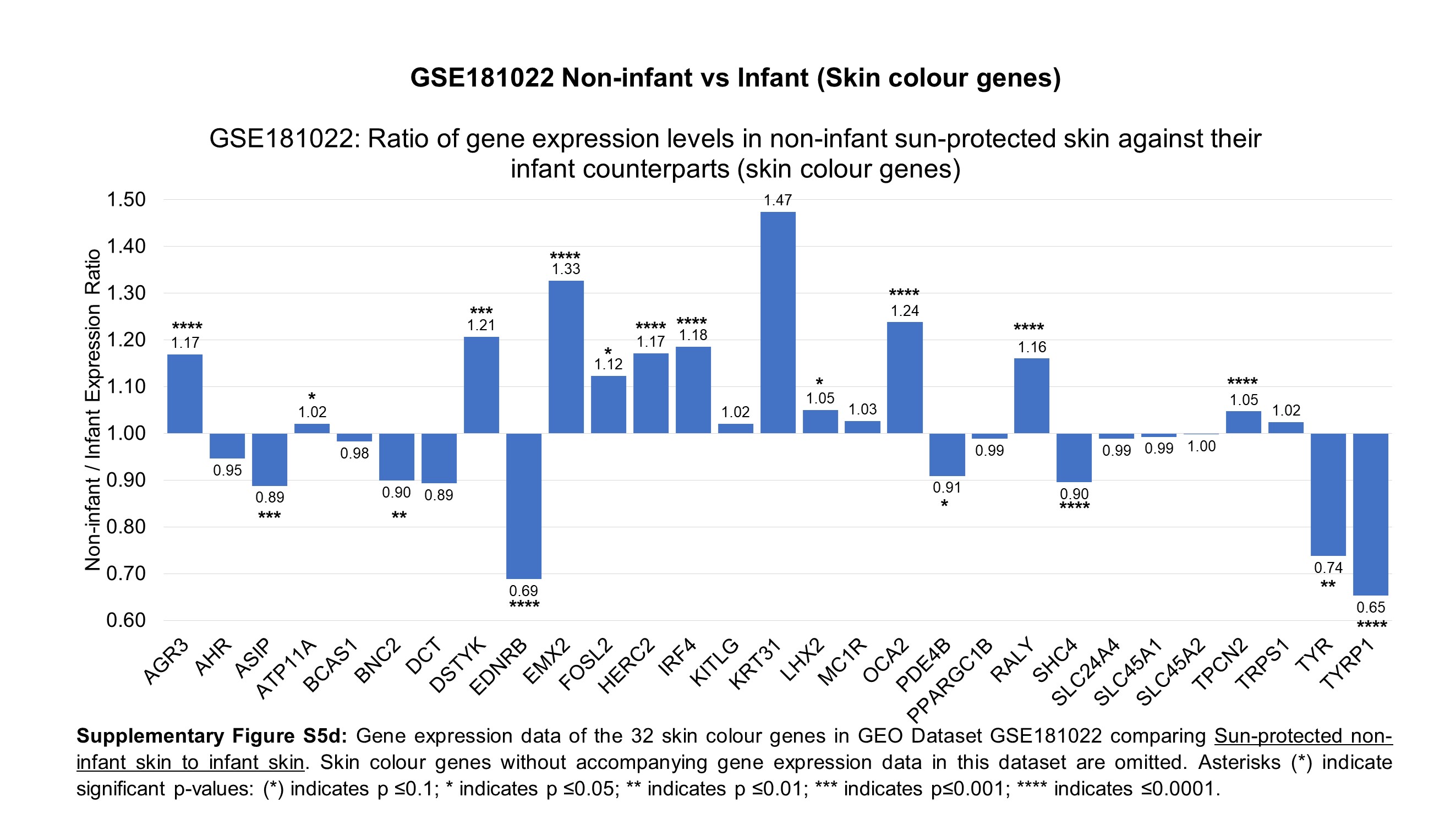

Supplement: Supplementary file 2 — Supplementary Information 2. [file 41598_2022_17443_MOESM2_ESM.zip › Supplementary Information/Figure S5 - GEO Dataset GSE181022/Supplementary Figure S5d.JPG]

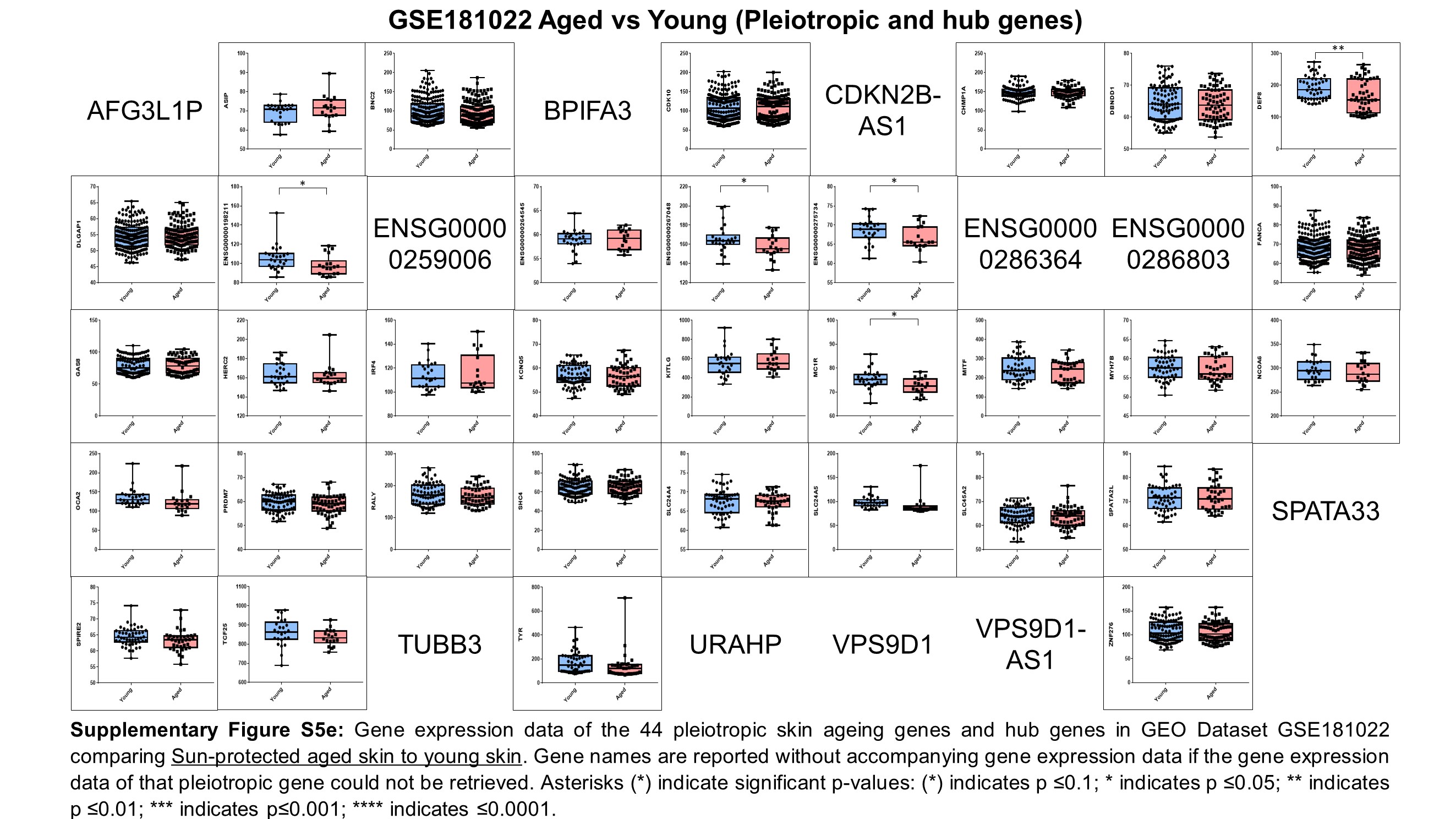

Supplement: Supplementary file 2 — Supplementary Information 2. [file 41598_2022_17443_MOESM2_ESM.zip › Supplementary Information/Figure S5 - GEO Dataset GSE181022/Supplementary Figure S5e.JPG]

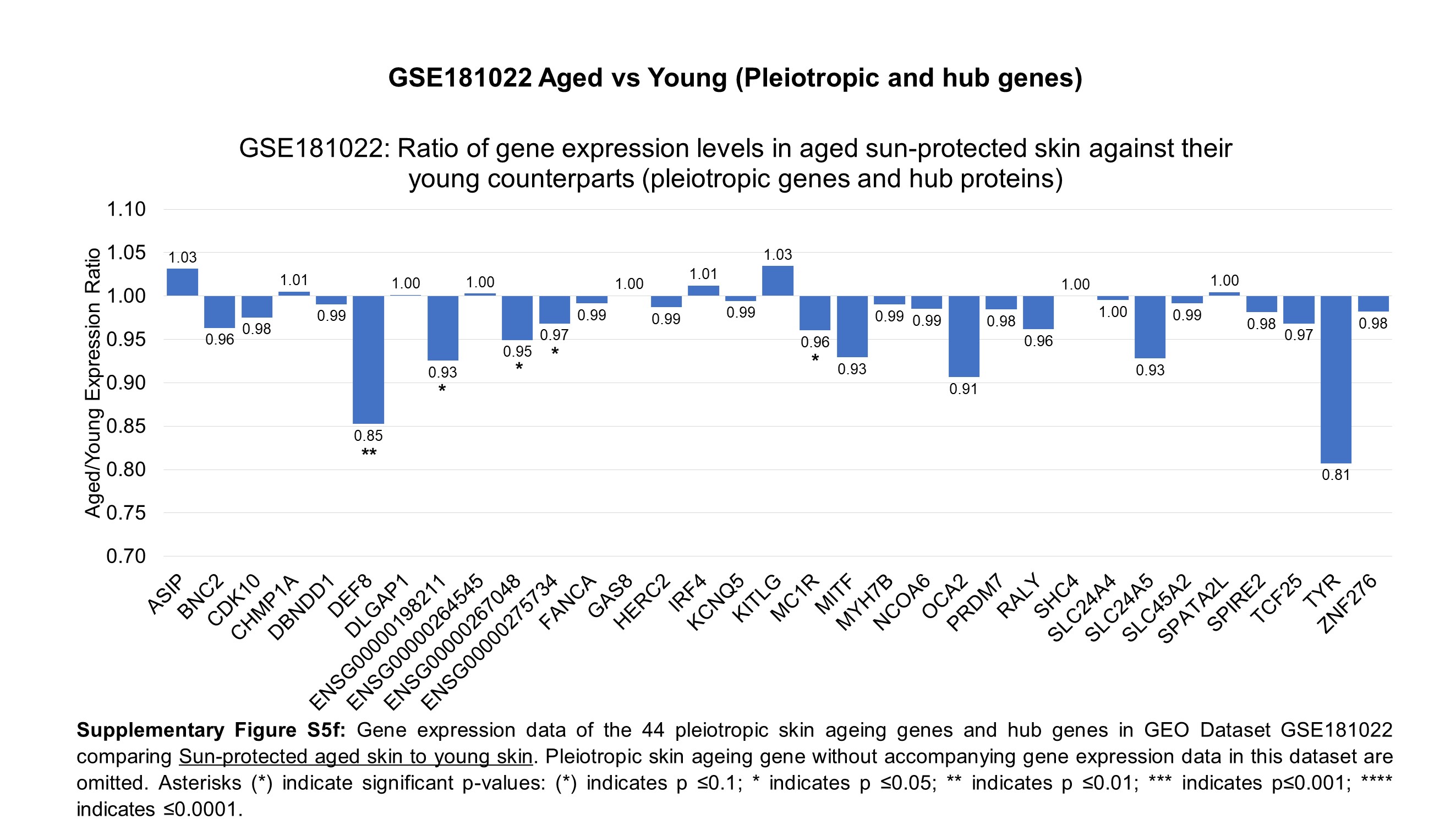

Supplement: Supplementary file 2 — Supplementary Information 2. [file 41598_2022_17443_MOESM2_ESM.zip › Supplementary Information/Figure S5 - GEO Dataset GSE181022/Supplementary Figure S5f.JPG]

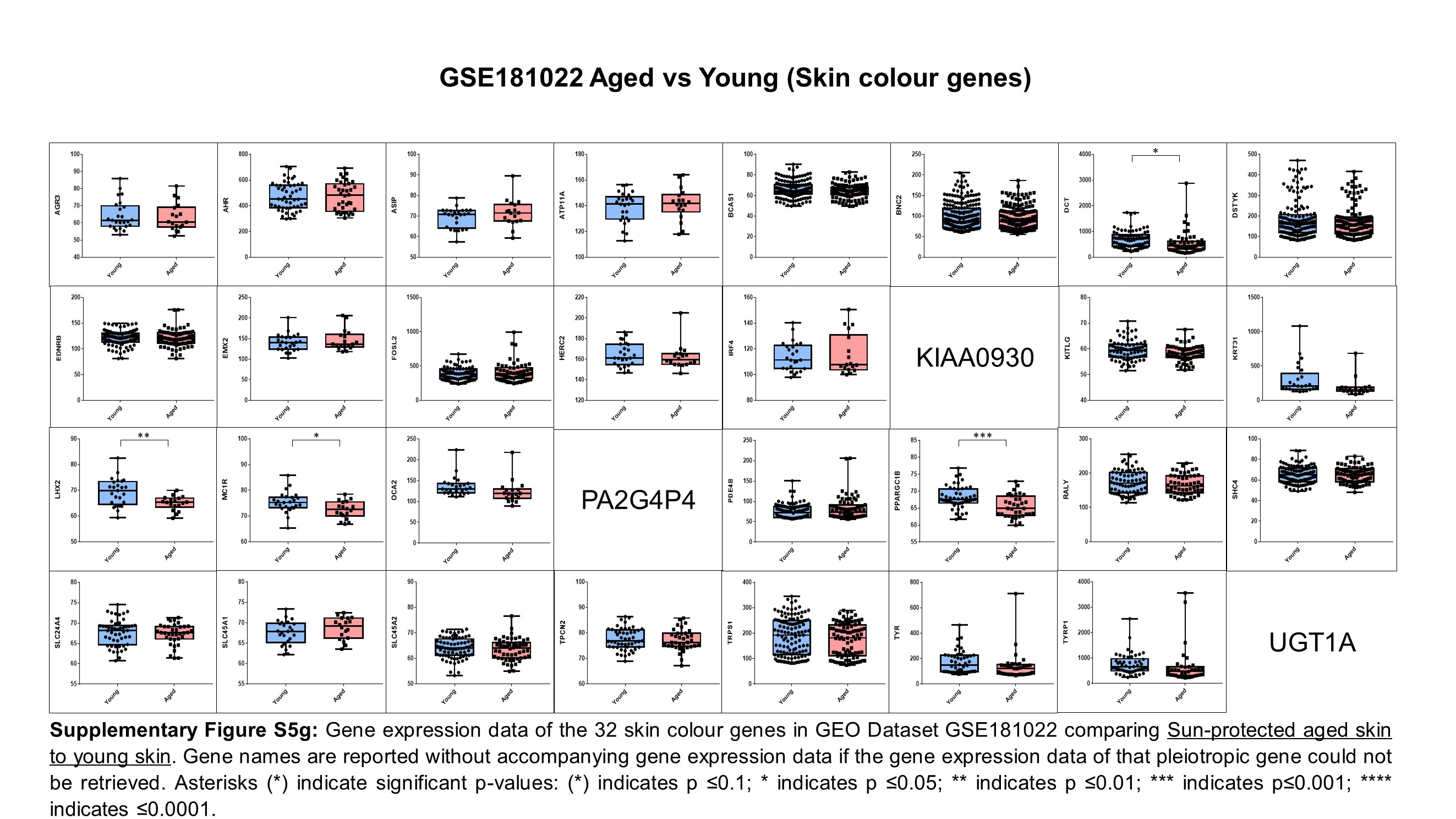

Supplement: Supplementary file 2 — Supplementary Information 2. [file 41598_2022_17443_MOESM2_ESM.zip › Supplementary Information/Figure S5 - GEO Dataset GSE181022/Supplementary Figure S5g.JPG]

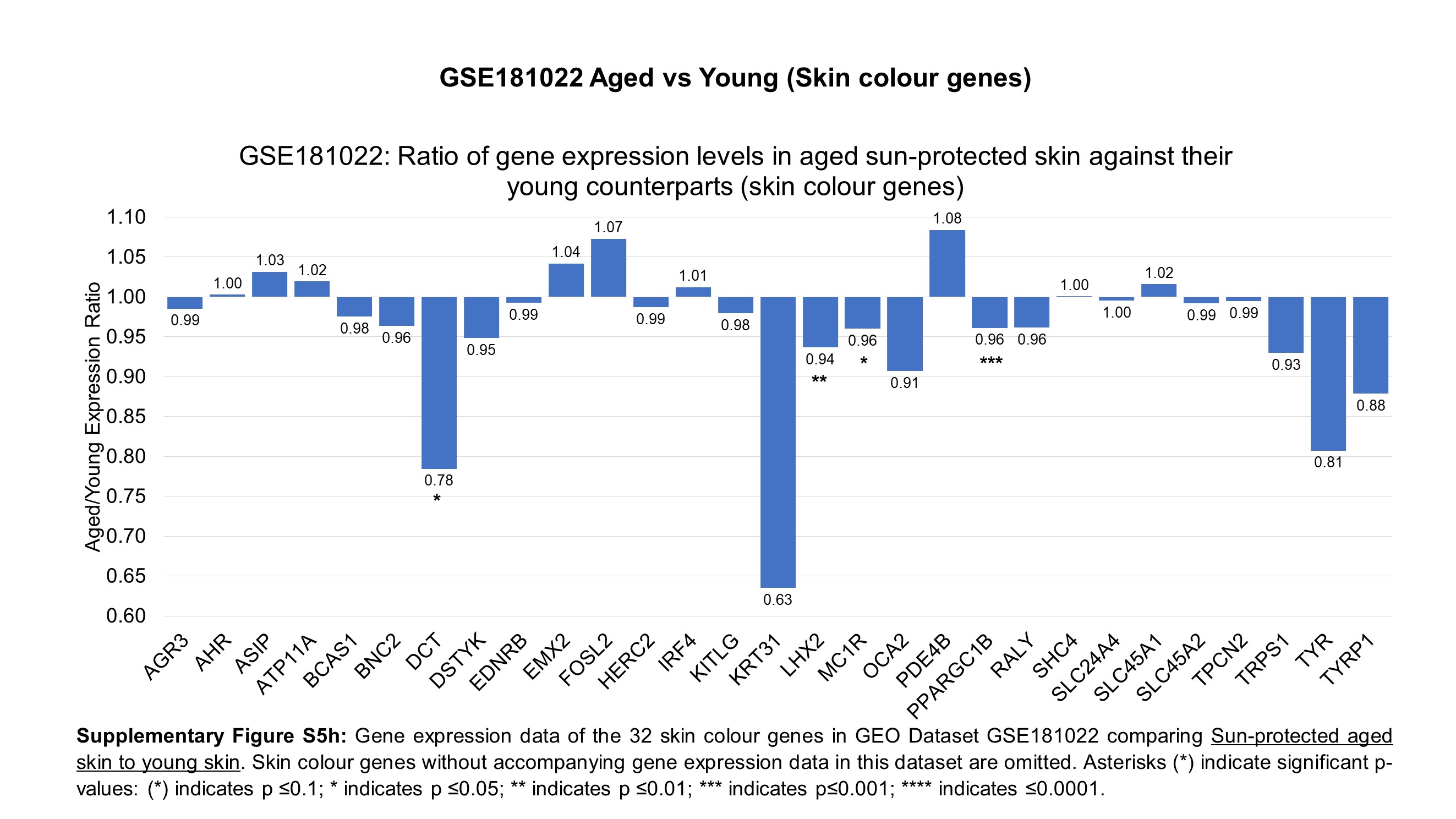

Supplement: Supplementary file 2 — Supplementary Information 2. [file 41598_2022_17443_MOESM2_ESM.zip › Supplementary Information/Figure S5 - GEO Dataset GSE181022/Supplementary Figure S5h.JPG]

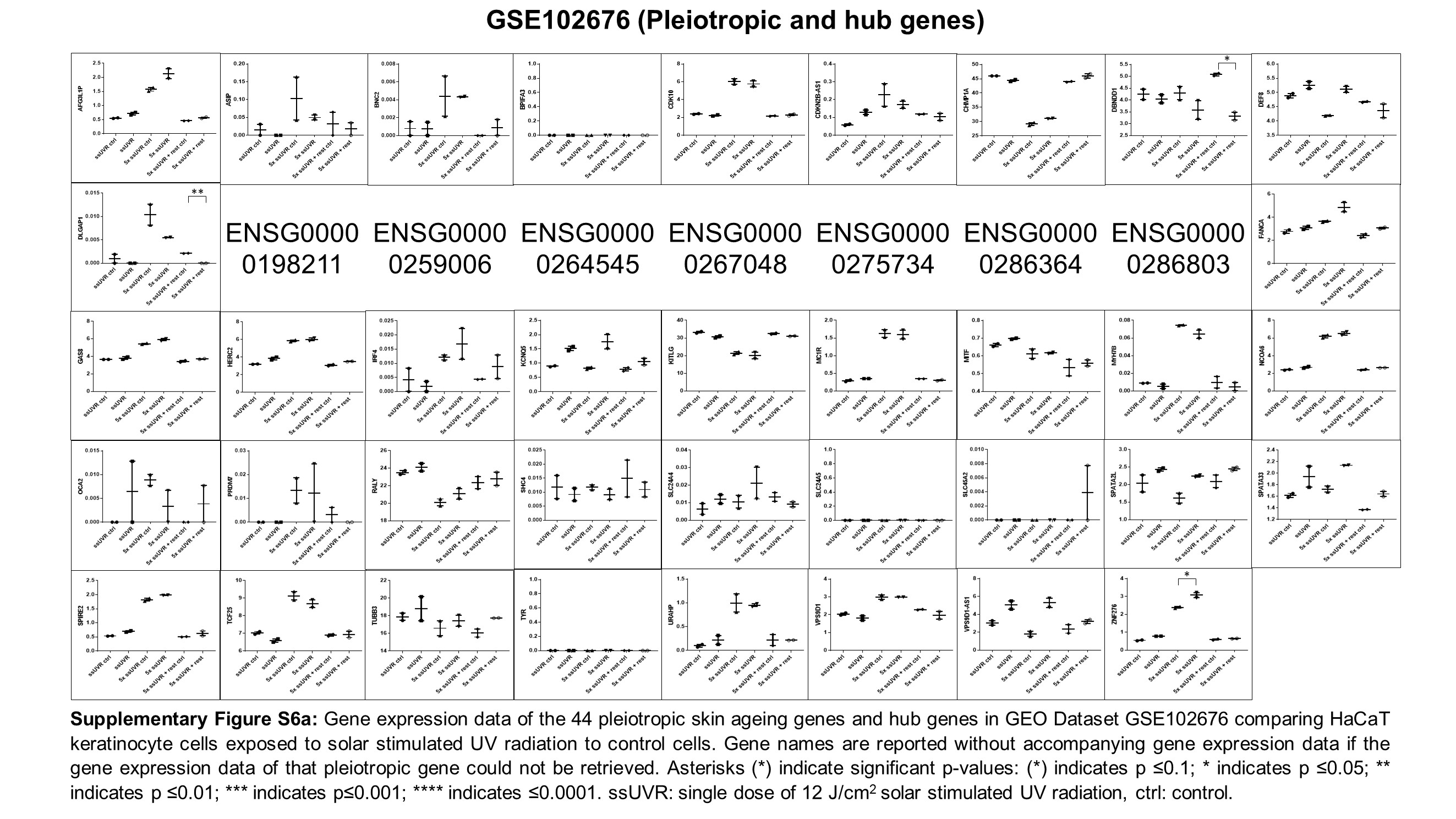

Supplement: Supplementary file 2 — Supplementary Information 2. [file 41598_2022_17443_MOESM2_ESM.zip › Supplementary Information/Figure S6 - GEO Dataset GSE102676/Supplementary Figure S6a.JPG]

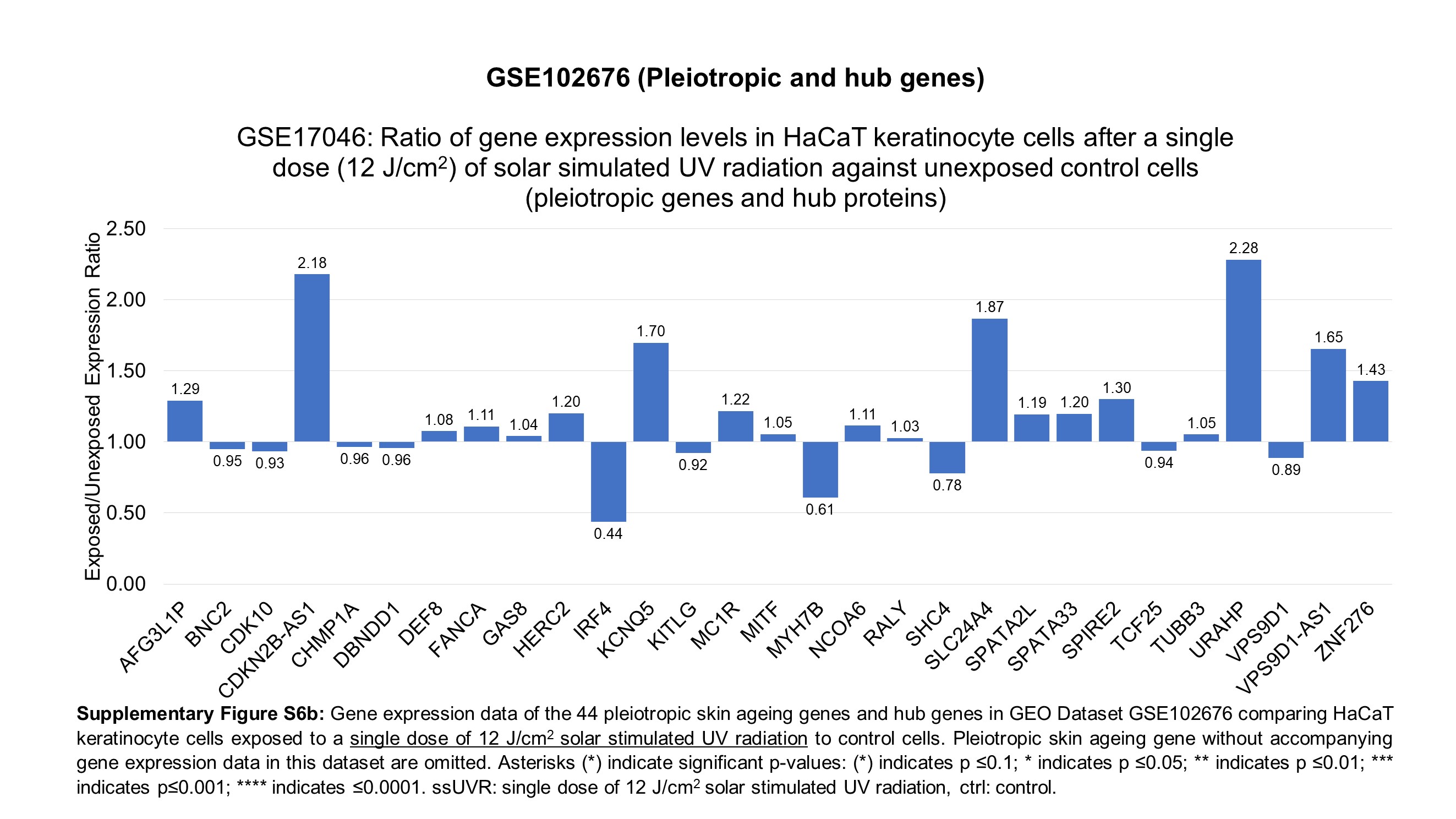

Supplement: Supplementary file 2 — Supplementary Information 2. [file 41598_2022_17443_MOESM2_ESM.zip › Supplementary Information/Figure S6 - GEO Dataset GSE102676/Supplementary Figure S6b.JPG]

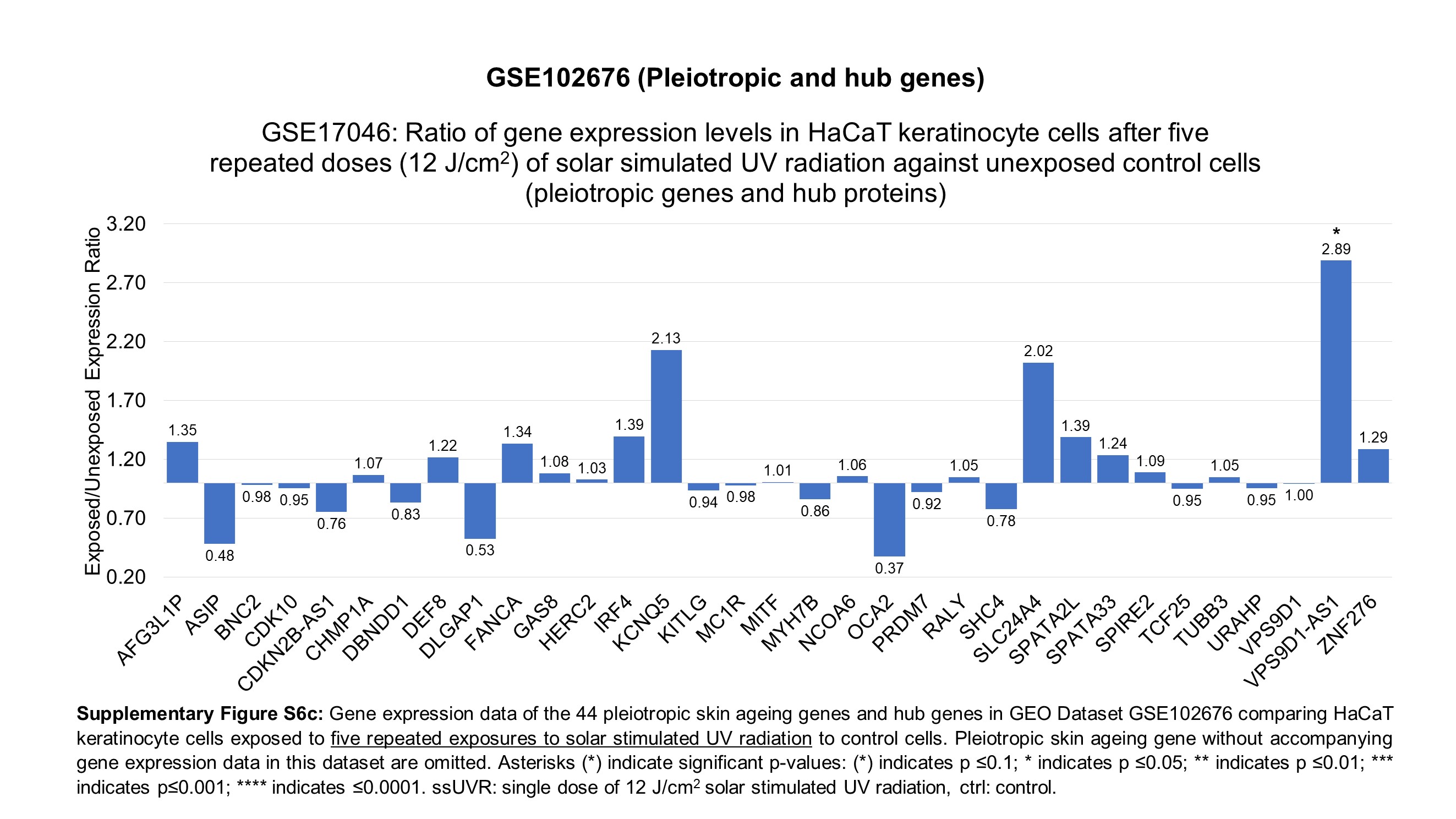

Supplement: Supplementary file 2 — Supplementary Information 2. [file 41598_2022_17443_MOESM2_ESM.zip › Supplementary Information/Figure S6 - GEO Dataset GSE102676/Supplementary Figure S6c.JPG]

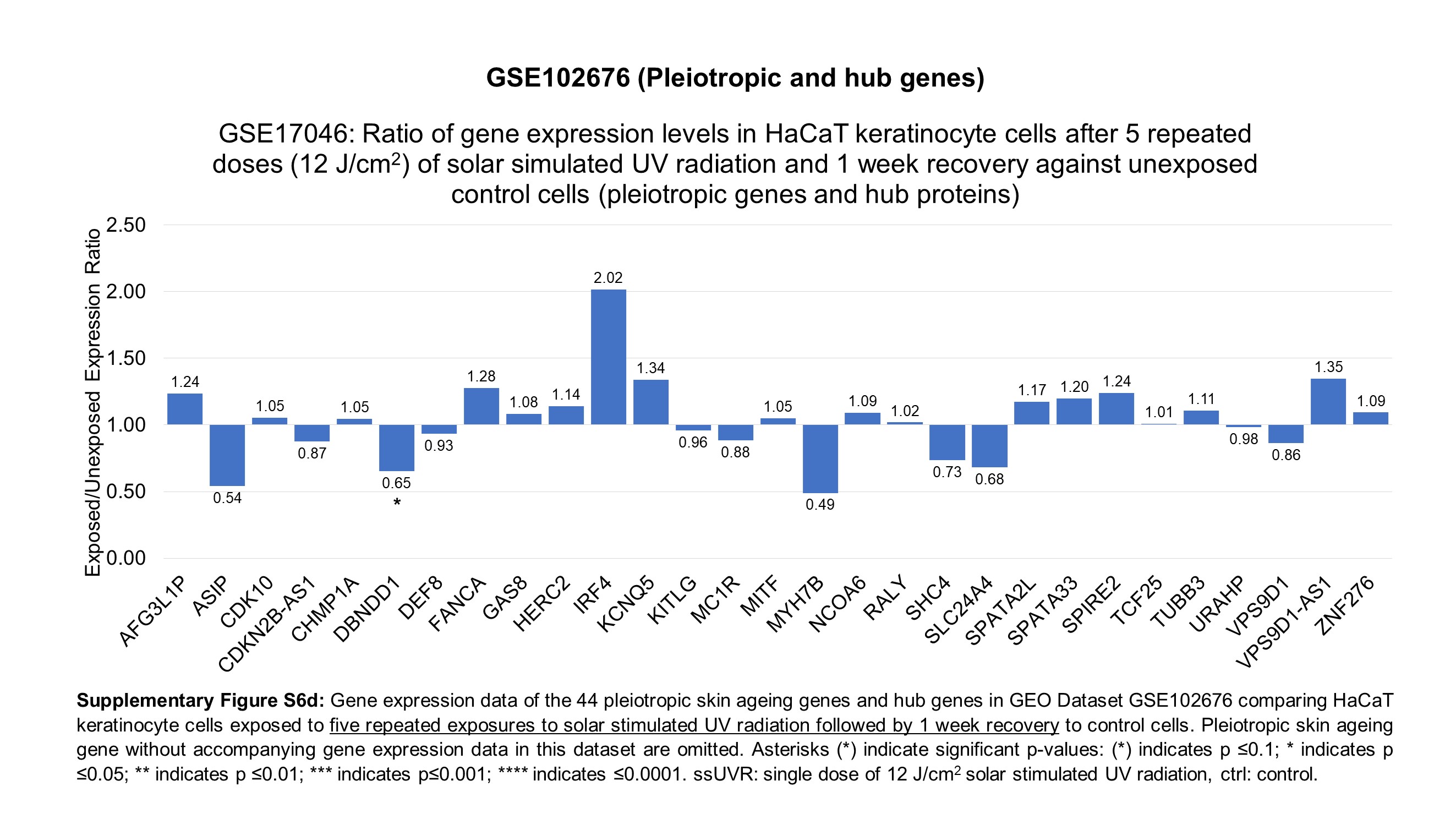

Supplement: Supplementary file 2 — Supplementary Information 2. [file 41598_2022_17443_MOESM2_ESM.zip › Supplementary Information/Figure S6 - GEO Dataset GSE102676/Supplementary Figure S6d.JPG]

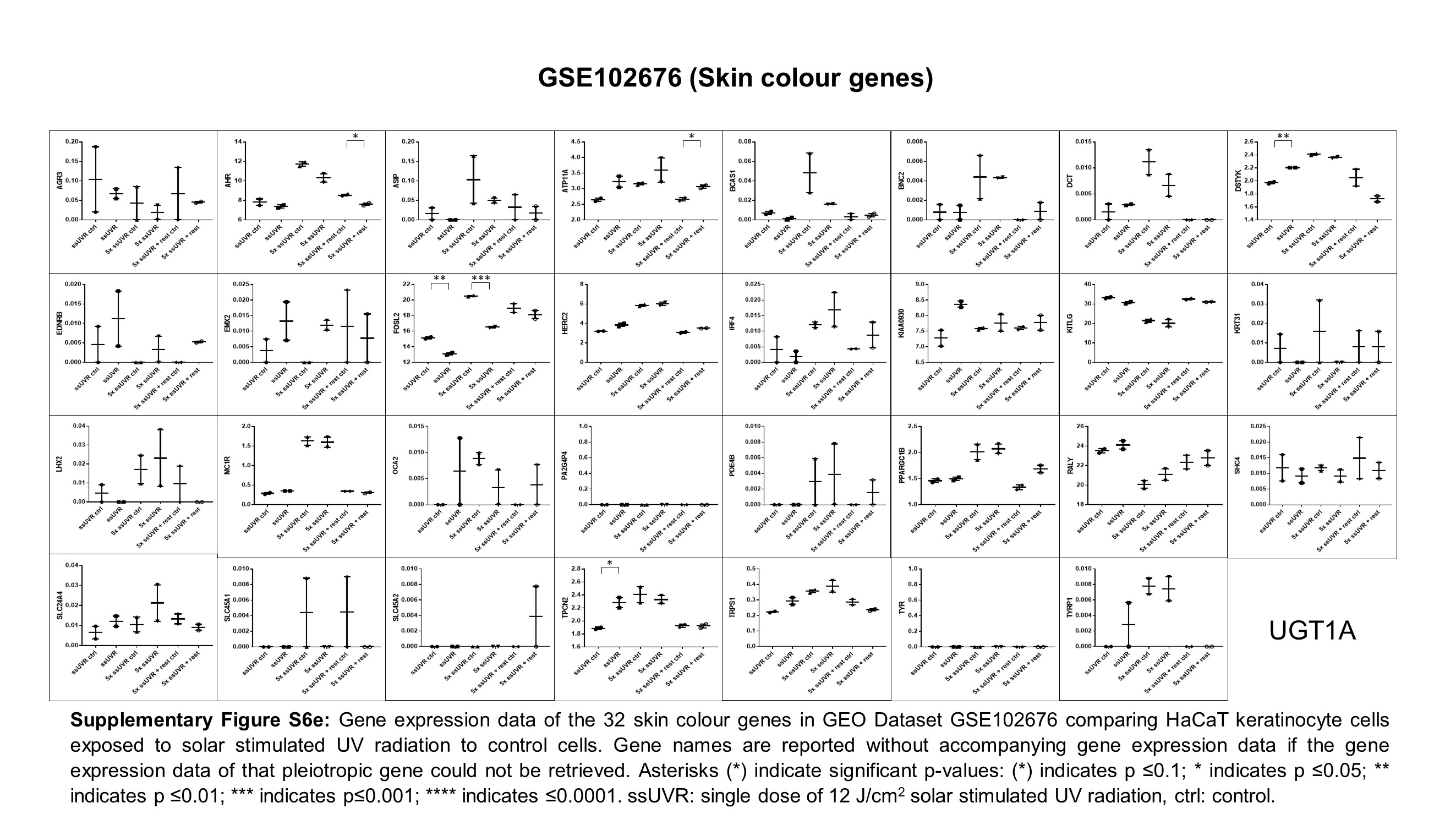

Supplement: Supplementary file 2 — Supplementary Information 2. [file 41598_2022_17443_MOESM2_ESM.zip › Supplementary Information/Figure S6 - GEO Dataset GSE102676/Supplementary Figure S6e.JPG]

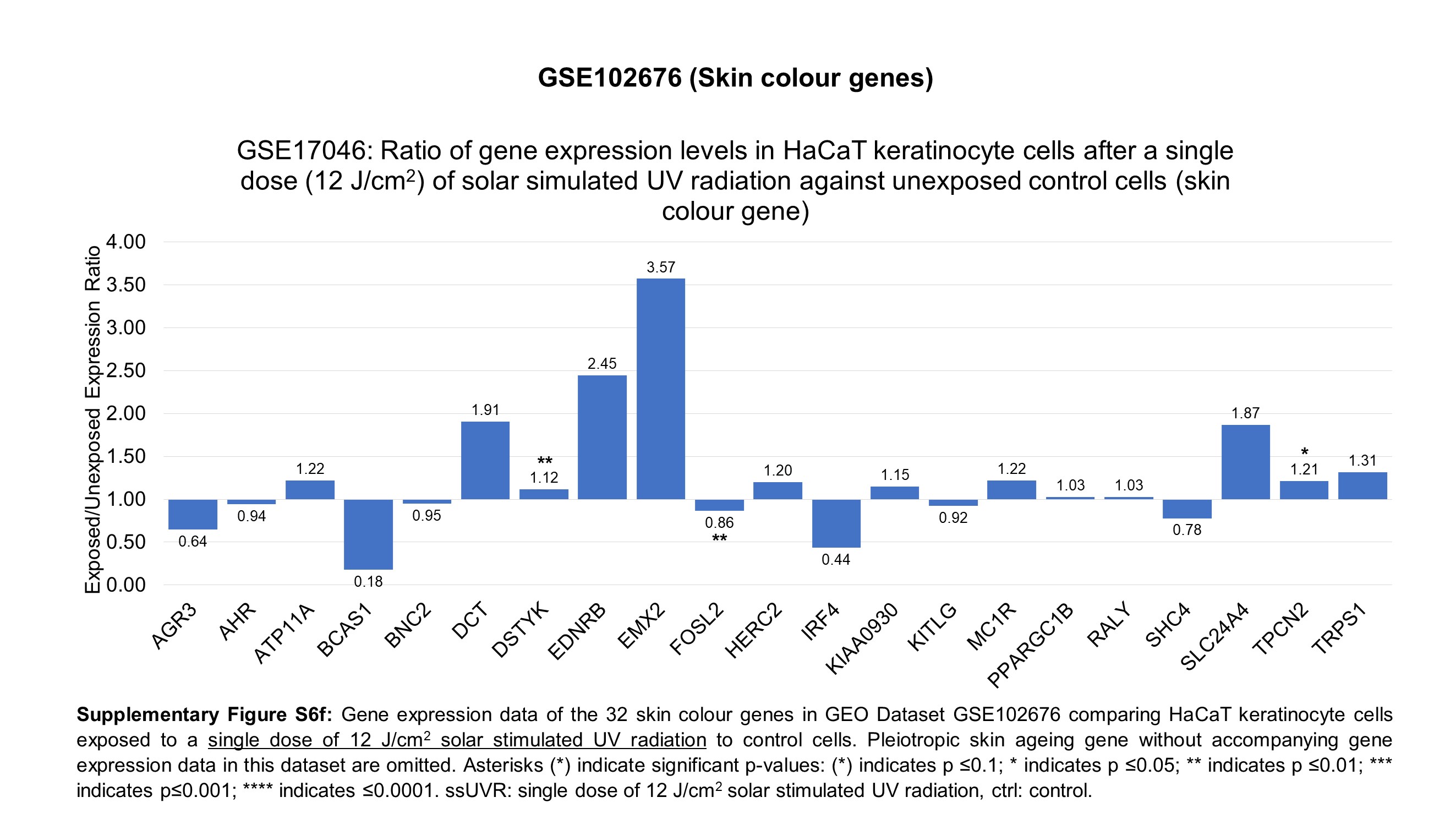

Supplement: Supplementary file 2 — Supplementary Information 2. [file 41598_2022_17443_MOESM2_ESM.zip › Supplementary Information/Figure S6 - GEO Dataset GSE102676/Supplementary Figure S6f.JPG]

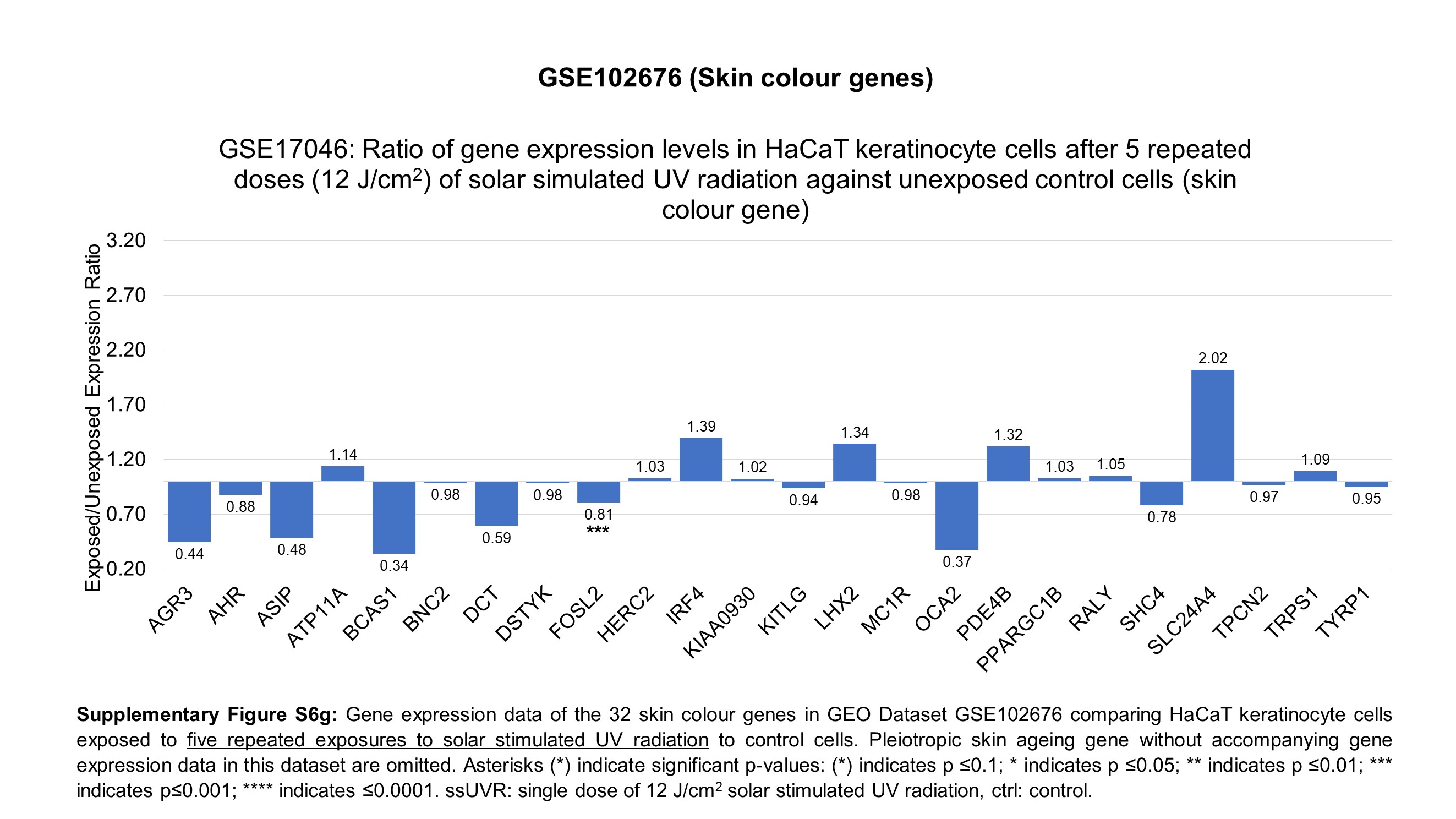

Supplement: Supplementary file 2 — Supplementary Information 2. [file 41598_2022_17443_MOESM2_ESM.zip › Supplementary Information/Figure S6 - GEO Dataset GSE102676/Supplementary Figure S6g.JPG]

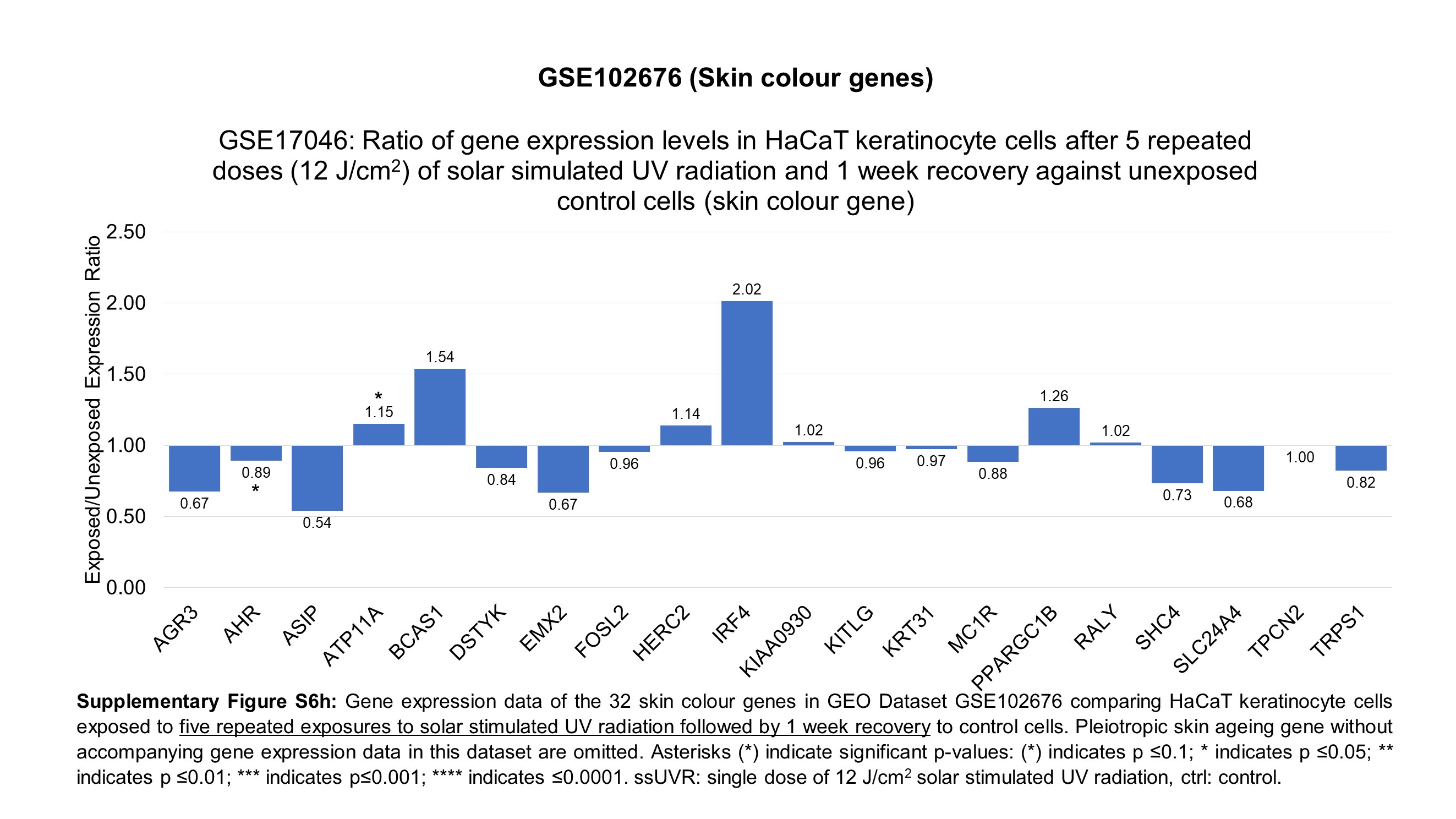

Supplement: Supplementary file 2 — Supplementary Information 2. [file 41598_2022_17443_MOESM2_ESM.zip › Supplementary Information/Figure S6 - GEO Dataset GSE102676/Supplementary Figure S6h.JPG]

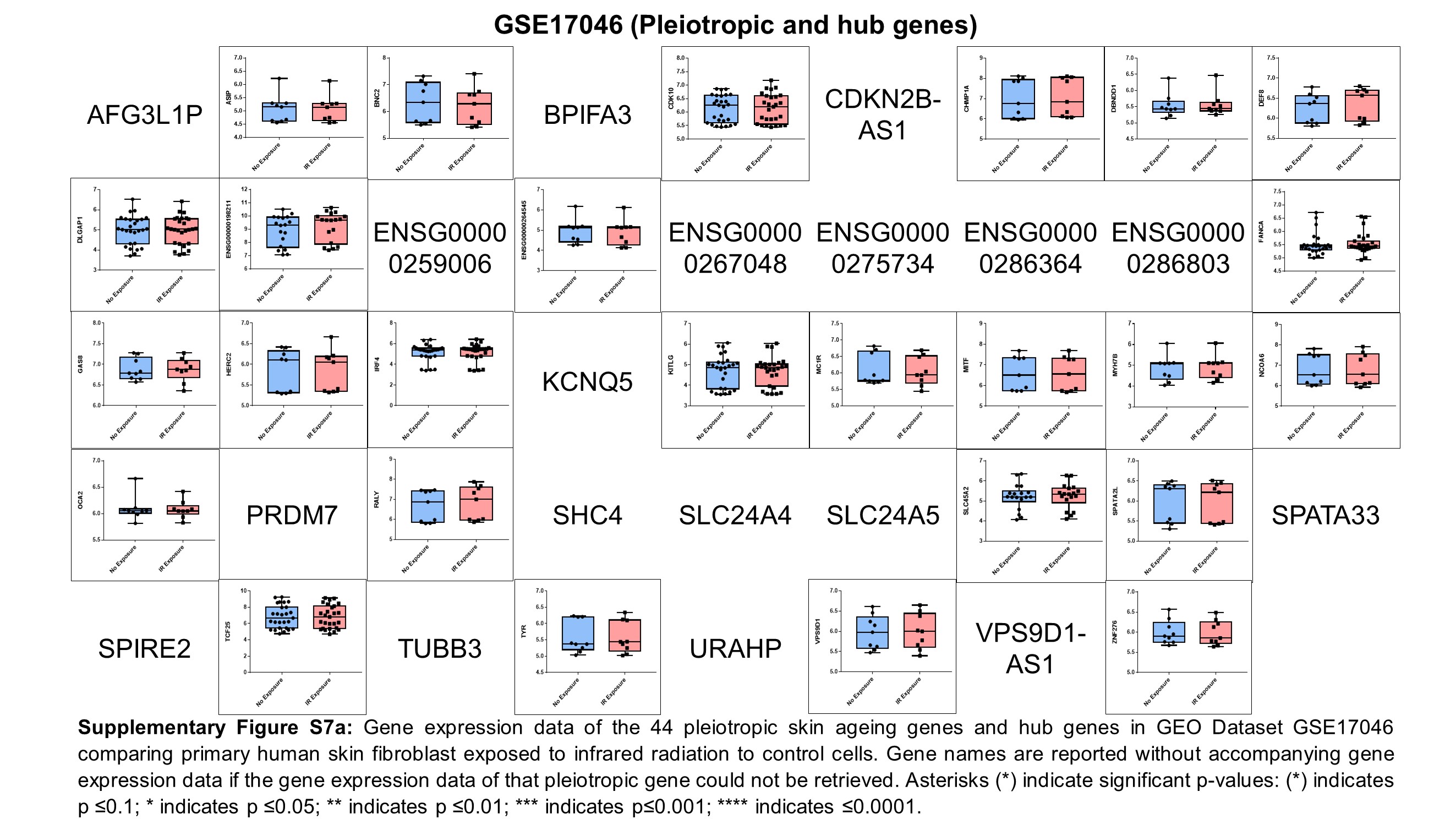

Supplement: Supplementary file 2 — Supplementary Information 2. [file 41598_2022_17443_MOESM2_ESM.zip › Supplementary Information/Figure S7 - GEO Dataset GSE17046/Supplementary Figure S7a.JPG]

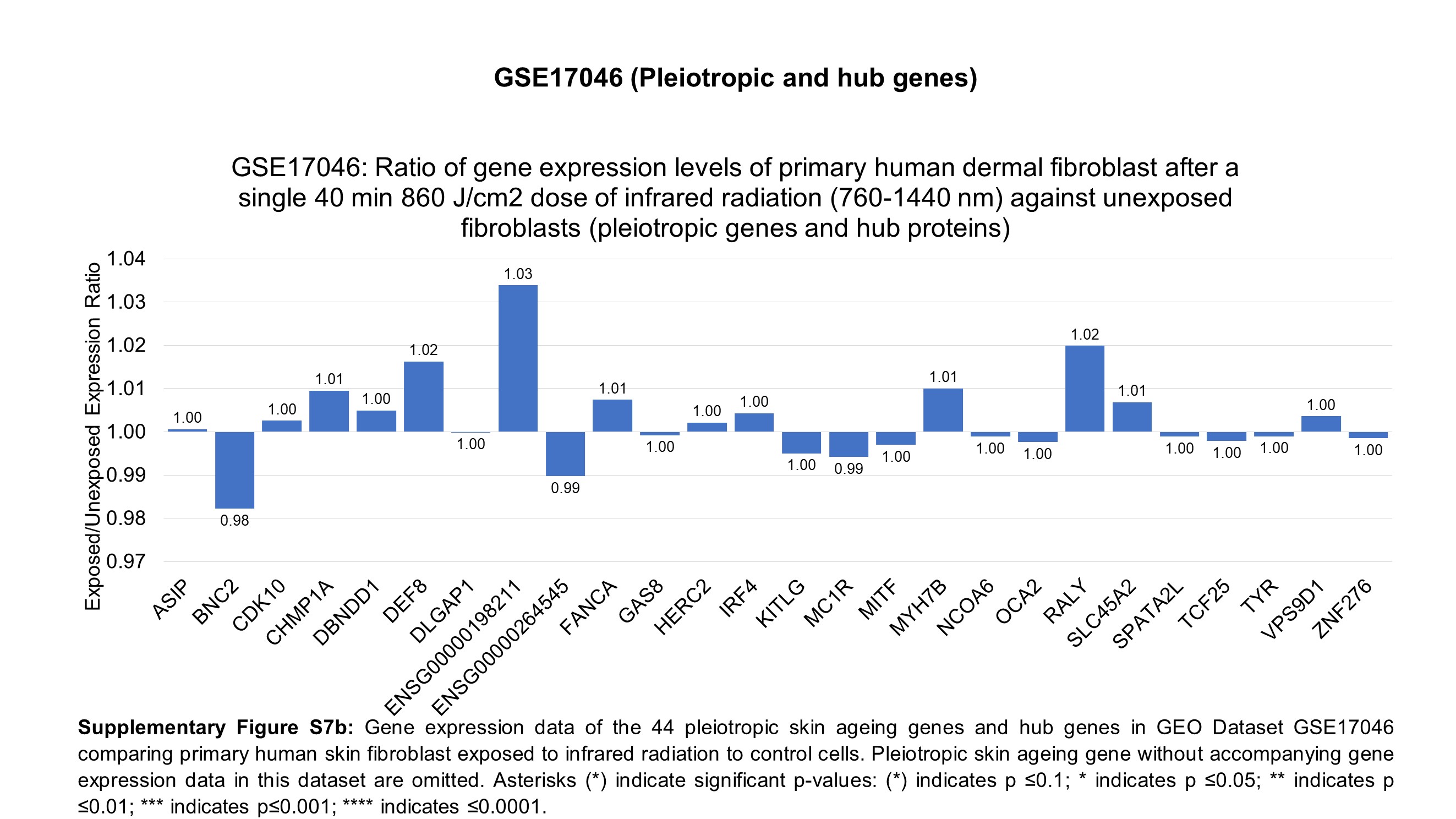

Supplement: Supplementary file 2 — Supplementary Information 2. [file 41598_2022_17443_MOESM2_ESM.zip › Supplementary Information/Figure S7 - GEO Dataset GSE17046/Supplementary Figure S7b.JPG]

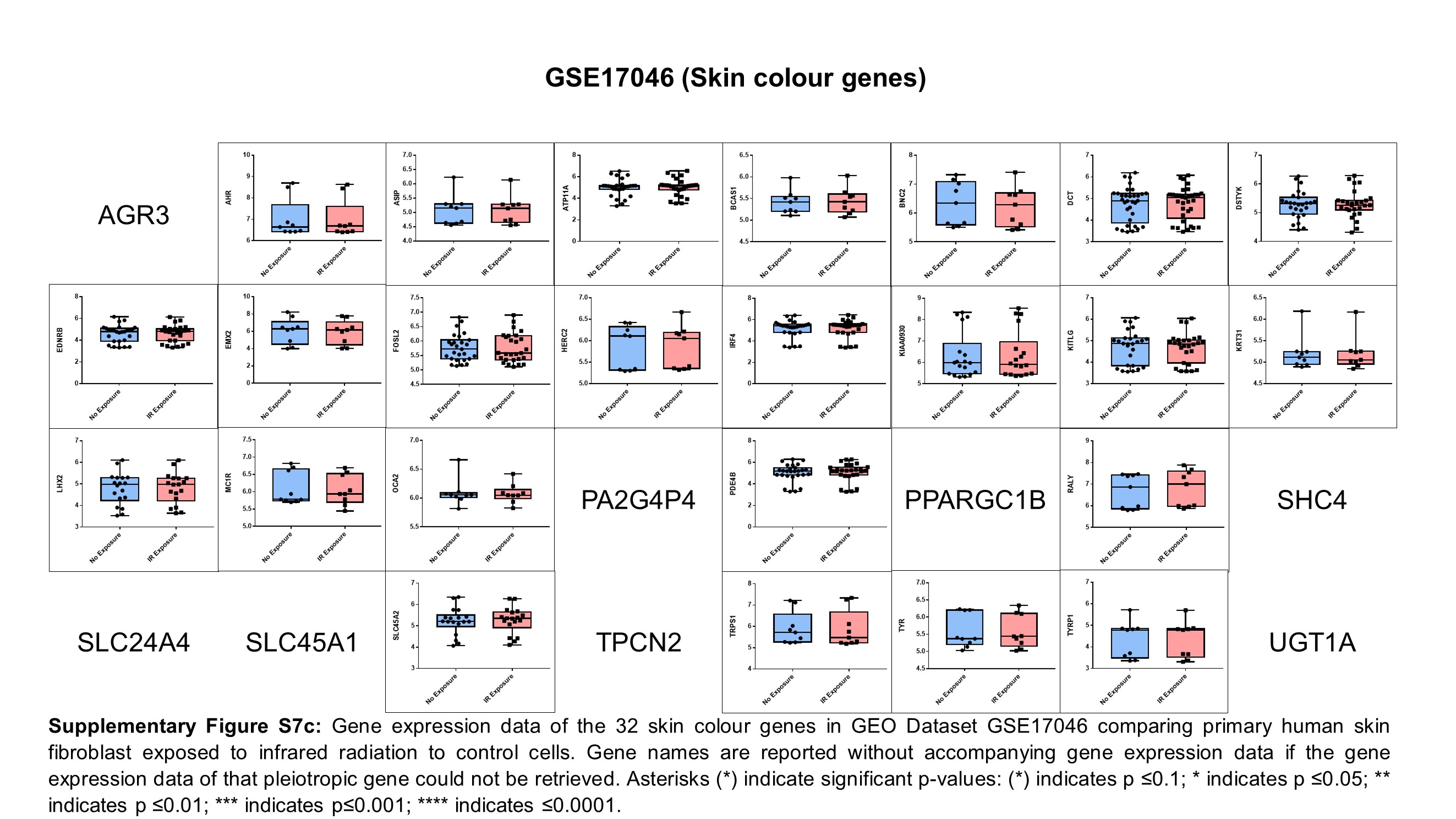

Supplement: Supplementary file 2 — Supplementary Information 2. [file 41598_2022_17443_MOESM2_ESM.zip › Supplementary Information/Figure S7 - GEO Dataset GSE17046/Supplementary Figure S7c.JPG]

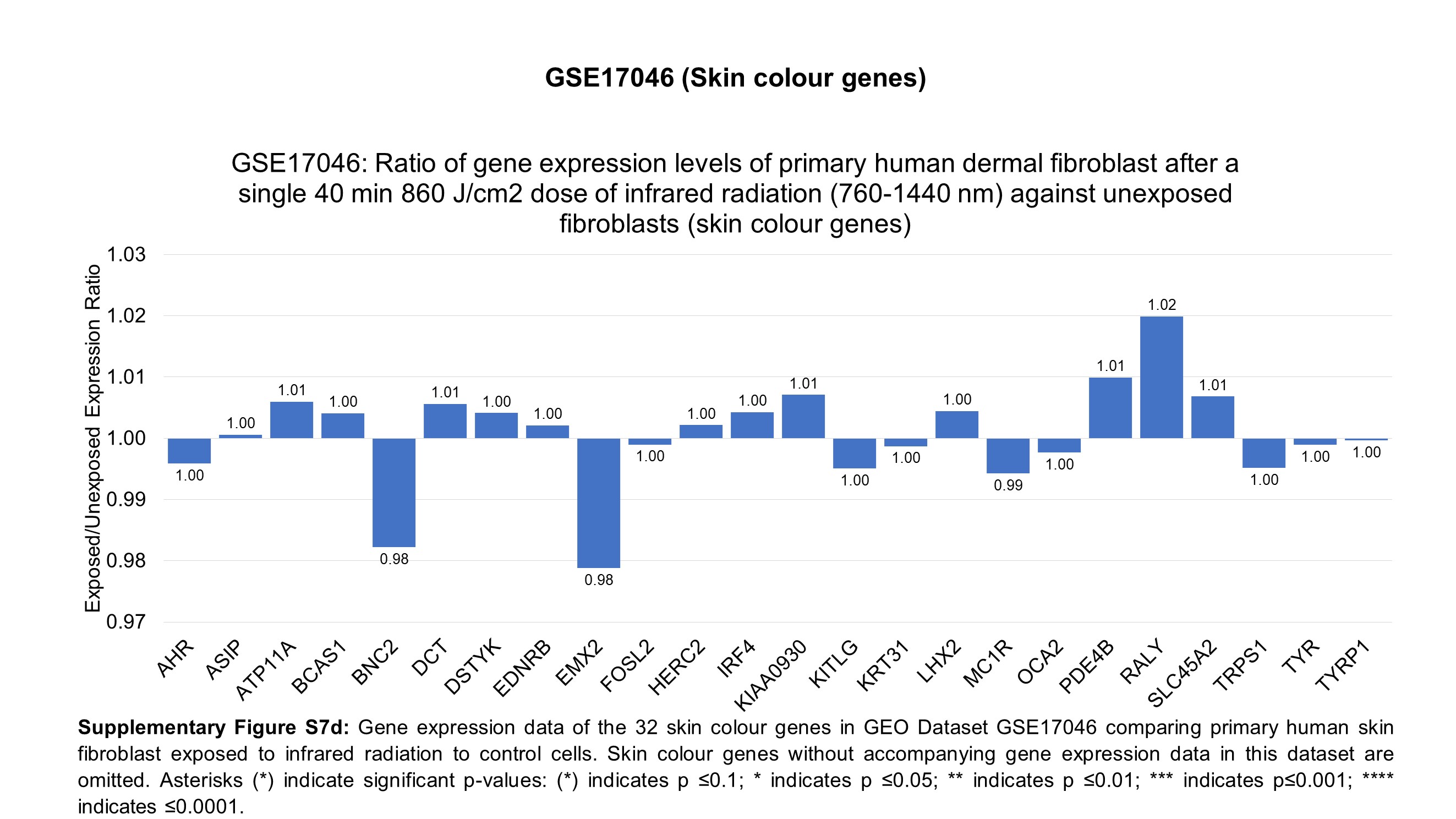

Supplement: Supplementary file 2 — Supplementary Information 2. [file 41598_2022_17443_MOESM2_ESM.zip › Supplementary Information/Figure S7 - GEO Dataset GSE17046/Supplementary Figure S7d.JPG]
